# Identification of candidate genes for prostate cancer-risk SNPs utilizing a normal prostate tissue eQTL data set

**DOI:** 10.1038/ncomms9653

**Published:** 2015-11-27

**Authors:** S. N. Thibodeau, A. J. French, S. K. McDonnell, J. Cheville, S. Middha, L. Tillmans, S. Riska, S. Baheti, M. C. Larson, Z. Fogarty, Y. Zhang, N. Larson, A. Nair, D. O’Brien, L. Wang, D J. Schaid

**Affiliations:** 1 Department of Laboratory Medicine and Pathology, Mayo Clinic College of Medicine, 200 First Street SW, Rochester, Minnesota 55905, USA; 2 Department of Health Sciences Research, Mayo Clinic College of Medicine, 200 First Street SW, Rochester, Minnesota 55905, USA; 3 Department of Epidemiology and Public Health, University of Maryland School of Medicine, 660W Redwood Street, Baltimore, Maryland 21201, USA; 4 Department of Pathology, Medical College of Wisconsin, 8701 Watertown Plank Road, Milwaukee, Wisconsin 53226, USA

## Abstract

Multiple studies have identified loci associated with the risk of developing prostate cancer but the associated genes are not well studied. Here we create a normal prostate tissue-specific eQTL data set and apply this data set to previously identified prostate cancer (PrCa)-risk SNPs in an effort to identify candidate target genes. The eQTL data set is constructed by the genotyping and RNA sequencing of 471 samples. We focus on 146 PrCa-risk SNPs, including all SNPs in linkage disequilibrium with each risk SNP, resulting in 100 unique risk intervals. We analyse *cis*-acting associations where the transcript is located within 2 Mb (±1 Mb) of the risk SNP interval. Of all SNP–gene combinations tested, 41.7% of SNPs demonstrate a significant eQTL signal after adjustment for sample histology and 14 expression principal component covariates. Of the 100 PrCa-risk intervals, 51 have a significant eQTL signal and these are associated with 88 genes. This study provides a rich resource to study biological mechanisms underlying genetic risk to PrCa.

## Abstract (web summary)

Single nucleotide polymorphisms—SNPs—have been identified for prostate cancer but whether these SNPs alter the expression of genes is largely unknown. In this study, the authors search for genes located within 2 Mb of the SNPs and identify SNPs that influence gene expression, so called expression quantitative trait loci.

## Graphical abstract

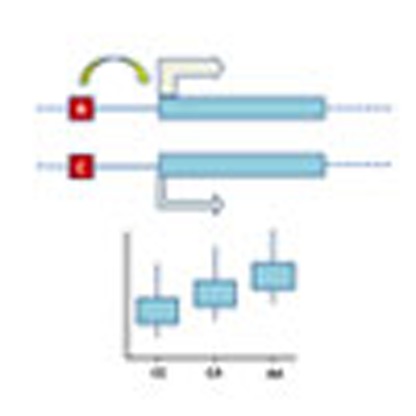

For US men, prostate cancer (PrCa) is the most frequent of all cancers (220,800 newly diagnosed cases annually) and the second most frequent for deaths due to cancer (27,540 deaths annually)^1^. Although the causes of the variation of PrCa incidence are likely to involve differences in screening methods, diet and health-related behaviours, clinical practice patterns and environmental risk factors, there is a large body of literature that also strongly implicates a genetic aetiology. This evidence comes from a variety of study designs, including case–control, cohort, twin and family-based studies^2^,^3^. Both linkage- and association-based strategies have been used to help identify candidate susceptibility loci for PrCa^3^,^4^. Multiple genome-wide association studies (GWAS) have now been performed^5^,^6^,^7^,^8^,^9^,^10^,^11^,^12^,^13^,^14^ yielding numerous single-nucleotide polymorphisms (SNPs) with an increased risk for PrCa. Importantly, a significant number of these have subsequently been validated in well-powered case–control studies^7^,^11^,^13^.

Despite the exceptional progress made for PrCa association-based studies, we are faced with the tremendous challenge of how to interpret these emerging results. There remains a substantial gap between disease–SNP associations derived from GWAS and an understanding of how these risk SNPs contribute to disease. Thus far, the functional role for the majority of risk SNPs for PrCa has not been determined, including knowledge of the target gene. As these risk SNPs have been found in non-coding regions of the genome, with many residing at some distance from any nearby annotated gene, it is believed that many of these (or their closely linked causal SNPs) are located in regulatory domains of the genome that control gene expression rather than in coding regions that directly affect protein function^15^. Thus, the results of GWAS performed for PrCa present with several key problems including: (1) the target gene for the risk SNP (or causal SNP) is most often unknown; (2) the causal SNP is unknown (most likely in linkage disequilibrium (LD) with the measured risk SNP); and (3) the functional consequence of the causal SNP is unknown.

A frequently used strategy to address these problems involves the use of gene expression^16^. There is a great deal of evidence indicating that regulatory SNPs are widespread in the human genome and genetic variations contribute appreciably to differences in gene expression phenotypes^17^,^18^,^19^. Loci that control the expression level of individual mRNA transcripts, referred to as expression quantitative trait loci (eQTL), can be globally identified by comparing genome-wide SNP and genome-wide mRNA expression on a common set of samples. These eQTLs may exist either in *cis* (*cis*-eQTL), probably owing to regulatory sequence polymorphisms, or in *trans* (*trans*-eQTL), presumably representing polymorphisms in transcription factors, miRNAs or pathways that ultimately lead to transcriptional control of a given gene. Publically available eQTL data sets are available, but most of these utilize lymphoblastoid cell lines. Thus, tissue-specific signals may be overlooked. Realizing the importance of eQTL, NIH (National Institutes of Health) established the Genotype Tissue Expression Project (GTEx)^20^,^21^. The goal of GTEx is to unravel the complex patterns of genetic variation and gene expression across diverse human tissue types^20^,^21^. However, large-scale prostate-specific eQTL data sets are not yet available.

In this study, we established and utilized a normal prostate tissue-specific eQTL data set to specifically target and analyse the association of previously reported PrCa-risk SNPs and their neighbouring SNPs with gene expression levels. Our goal was to identify candidate target genes for each of the PrCa-risk SNPs and to fine map the eQTL signal with a dense set of SNPs for each of the target genes identified. Of the 100 PrCa-risk intervals studied, 51 demonstrated a significant eQTL signal and these are associated with 88 genes.

## Results

### PrCa-risk SNP-based eQTL

**Figure 1:**
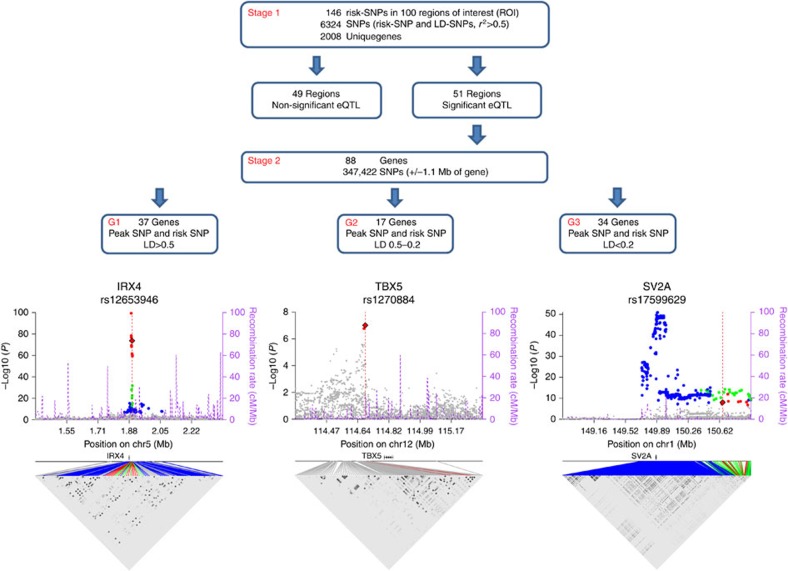
Schematic diagram outlining the eQTL analyses conducted for the primary and second stages. Schematic diagram outlining the analysis conducted for the primary analysis (focused on risk regions defined by PrCa-risk SNPs) and for the second stage (focused on target gene regions defined as all genes meeting a Bonferroni significance threshold in stage 1). Stage 2 results were classified into three groups (G1, G2 and G3) based on the magnitude of LD between the PrCa-risk SNP and the peak eQTL-associated SNP in each region; G1 defined as *r*^2^>0.5 between risk and peak SNPs, G2 having *r*^2^ between 0.2–0.5 and G3 having *r*^2^≤0.2. An example regional association plot is shown for each group. The *x* axis shows the chromosomal position of the SNPs (with analyzed gene in the region displayed below) and the *y* axis is the −log10(*P* value) obtained by regressing normalized expression levels for the gene listed in the panel title on the number of minor alleles of each SNP genotype adjusted for histologic characteristics and 14 expression principal components. The position of the PrCa-risk SNP is indicated by a dotted red vertical line with the eQTL result displayed as diamond. All Bonferroni significant results are coloured, with the colour defined by LD between the SNP and the PrCa-risk SNP listed in the panel title (LD *r*^2^>0.5 red, between 0.2–0.5 green and ≤0.2 blue). The right *y* axis shows the recombination rate (purple dotted lines mark recombination locations). The bottom half of each panel contains an LD heat map of the significant SNPs in the region (if >1,000 significant SNPs, only the top 1,000 SNPs in the region are shown).

Our primary analysis focused on identifying *cis*-eQTLs for 146 reported PrCa-risk SNPs (Supplementary Data 1), including all observed and high-quality imputed SNPs in LD with each risk SNP (*r*^2^>0.5), resulting in a total of 6,324 risk and LD-SNPs to be evaluated in 100 unique risk intervals (several of the risk SNPs were in close proximity to each other and were combined in a single risk interval) (Fig. 1). The risk SNPs and the number of SNPs evaluated for each of the risk intervals is provided in Supplementary Data 2.

A total of 3,229 gene transcripts surrounding the risk-SNP intervals were identified. Of these, 885 were not evaluated due to low expression, leaving 2,344 for further analysis. Some gene transcripts fell within 2 Mb of multiple risk-SNP intervals resulting in 2,008 unique gene transcripts (Fig. 1). The number of genes localized to each risk-SNP interval is provided in Supplementary Data 2, while the expression profile for all genes in each of the 100 regions is shown in Supplementary Fig. 1.

We first evaluated the statistical association for each LD-SNP within our regions of interest with each of the expressed transcripts, for a total of 127,276 association tests. A linear regression model, regressing normalized expression levels on the number of minor alleles of each SNP genotype, adjusted for histologic characteristics and expression principal components (PC) was used to obtain all *P* values reported in this study. Of the 6,324 LD-SNPs located in the 100 risk intervals, 2,638 (41.7%) demonstrated a significant eQTL signal after adjustment for covariates and meeting a Bonferroni-adjusted *P* value threshold of 1.96E-07 (significant *P* values ranged from 1.96E-07 to 6.37E-154) resulting in 4,174 SNP–gene pairs. Of the 100 PrCa-risk intervals, 51 (51%) demonstrated a Bonferroni significant eQTL signal and these were associated with 88 genes (Fig. 1 and Supplementary Data 1 and 2). For each of the significant SNP–gene association tests, the effect size and direction of effect on the mRNA expression is provided by the β-coefficient, obtained from regressing normalized expression levels on the number of minor alleles of each SNP genotype, adjusted for histologic characteristics percent lymphocytic population and percent epithelium present, and 14 principal components (PCs; Methods and Supplementary Data 1).

### Gene-based eQTL

Genes that were statistically significantly associated with one or more PrCa-risk SNPs were considered *cis*-eQTL ‘target genes’ for PrCa risk. To fully identify all *cis*-eQTLs for these target genes, we conducted a second-stage eQTL analysis where we focused on all SNPs surrounding the 88 target genes identified in the primary analysis. On the basis of empirical data using publically available information, we observed that 99% of all eQTLs are located within 1.1 Mb from either the transcription start site (TSS) or transcription end site (TES) of all genes examined (see Methods). Thus, the SNP regions of interest for the second-stage analysis were defined as those localized ±1.1 Mb from the gene TSS or TES positions. Of the 347,442 SNPs surrounding the 88 target gene regions of interest, and excluding those SNPs included in the primary analysis, 36,122 additional SNPs demonstrated a significant eQTL signal after adjustment for covariates and meeting a Bonferroni-adjusted *P* value threshold of 3.02E-08 (significant *P* values ranged from 3.02E-08 to 172E-202). Of the 88 target genes, 82 were found to have significant eQTL signals beyond what was originally detected in the primary analysis. There were no additional statistically significant eQTLs for 6 of the 88 target genes. Examples are provided in Fig. 1 and plots for all genes are provided in Supplementary Figs 2–4,

On the basis of the LD between the PrCa-risk SNP and the peak eQTL signal, considering both primary- and second-stage analyses, the 88 target gene regions of interest were placed into three separate categories (Fig. 1 and Supplementary Figs 2–4). The Pearson correlation was used to measure LD between the PrCa-risk SNP and the SNP most strongly associated with expression level (peak eQTL signal), and then used to create the three groups: *r*^2^ of >0.5 between the two SNPs, *r*^2^ of 0.2–0.5, and *r*^2^ of <0.2 (no LD) for groups 1, 2 and 3, respectively. Of the 88 gene regions of interest, 37 (42%), 17 (19%) and 34 (39%) were placed in groups 1, 2 and 3, respectively. The distance from the risk SNP and the peak eQTL signal varied considerably, with a mean of 98 kb and ranging from 0 to a higher value of ∼760 kb.

To determine whether the target genes had evidence of multiple independent regulatory SNPs, we evaluated the statistical association for each SNP in the target gene region adjusting for the peak eQTL signal SNP. Conditioning on the peak eQTL signal for each of the 88 target genes, no SNPs (including the original risk SNP) demonstrated a Bonferroni significant eQTL signal for 44 (50%) of the target genes. These included 25 (68%) of the 37 genes in group 1, 10 (59%) of the 17 genes in group 2 and 9 (26%) of the 34 genes in group 3. On the other hand, significant residual eQTL signals were found for the remaining 44 genes, including 12 (32%) of the 37 genes in group 1, 7 (41%) of the 17 genes in group 2 and 25 (74%) of the 34 genes in group 3. Among these 44, 13 had a second eQTL signal involving variants in high LD with the PrCa-risk SNP (*r*^2^>0.5); 3 in group 1, 1 in group 2 and 9 in group 3. Adjusted *P* values for some of the SNPs in the risk region, including the PrCa-risk SNP are provided in Supplementary Figs 2–4.

### Characteristics of significant *cis*-eQTL findings

Of the 51 risk intervals demonstrating a significant eQTL signal, 33 comprised significant SNP–gene associations involving a single gene. For the remaining 18 risk intervals, the risk SNPs were each associated with two or more genes, ranging from 2 to 7 genes per region (2 genes in 10 regions, 3 genes in 2 regions, 4 genes in 3 regions, 6 genes in 1 region and 7 genes in 2 regions).

**Figure 2:**
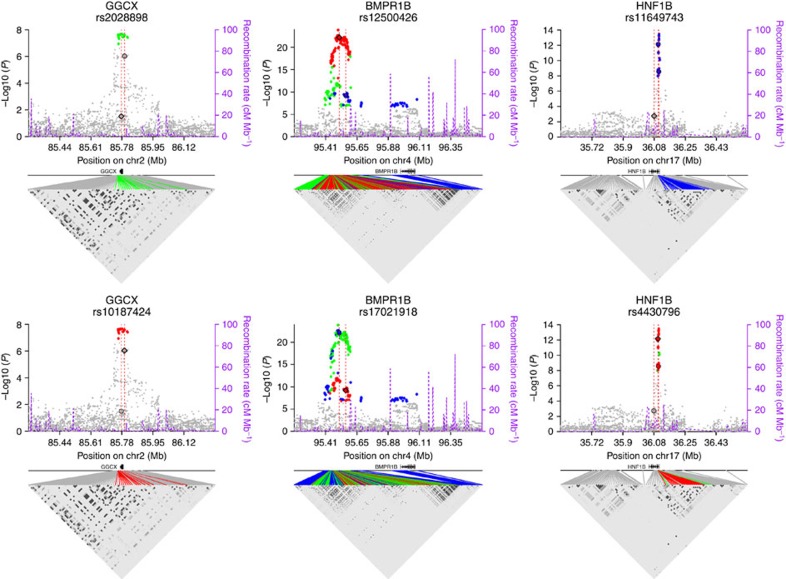
Regional association plots for regions containing multiple risk SNPs. Regional association plots are presented for three gene regions, each of which contain two or more PrCa-risk SNPs with varying degrees of LD between them (*r*^2^=0.54 for *GGCX* region, *r*^2^<0.2 for *BMPR1B* region and *r*^2^=0.67 for *HNF1B* region. The *x* axis shows the chromosomal position of the SNPs (with analyzed gene in the region displayed below) and the *y* axis is the −log10 (*P* value) obtained by regressing normalized expression levels for the gene listed in the panel title on the number of minor alleles of each SNP genotype adjusted for histologic characteristics and 14 expression principal components. The position of the PrCa-risk SNP is indicated by a dotted red vertical line with the eQTL result displayed as diamond. All Bonferroni significant results are coloured, with the colour defined by LD between the SNP and the PrCa-risk SNP listed in the panel title (LD *r*^2^>0.5 red, between 0.2–0.5 green and ≤0.2 blue). The right *y* axis shows the recombination rate (purple dotted lines mark recombination locations). The bottom half of each panel contains an LD heat map of the significant SNPs in the region (if >1,000 significant SNPs, only the top 1,000 SNPs in the region are shown). For each gene region, two or more plots are shown depending on the number of risk SNPs in the region, one for each risk SNP. The points are coloured according to LD with the risk SNP listed in the panel title (*r*^2^>0.5, red; between 0.2–0.5, green; and ≤0.2, blue). The eQTL result for the PrCa-risk SNP listed in the panel title is displayed as diamond, the data points for all of the other PrCa-risk SNPs are displayed as an open circle.

**Figure 3:**
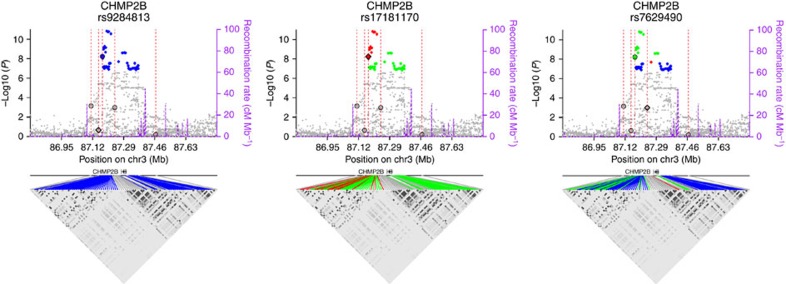
Regional association plots for the *CHMP2B* gene region containing multiple risk SNPs. Regional association plot presented for the *CHMP2B* gene region containing several PrCa-risk SNPs with varying degrees of LD between them. The *x* axis shows the chromosomal position of the SNPs (with analyzed gene in the region displayed below) and the *y* axis is the −log10(*P* value) obtained by regressing normalized expression levels for the gene listed in the panel title on the number of minor alleles of each SNP genotype adjusted for histologic characteristics and 14 expression principal components. The position of the PrCa-risk SNP is indicated by a dotted red vertical line with the eQTL result displayed as diamond. All Bonferroni significant results are coloured, with the colour defined by LD between the SNP and the PrCa-risk SNP listed in the panel title (LD *r*^2^>0.5, red; between 0.2–0.5, green; and ≤0.2, blue). The right *y* axis shows the recombination rate (purple dotted lines mark recombination locations). The bottom half of each panel contains an LD heat map of the significant SNPs in the region (if >1,000 significant SNPs, only the top 1,000 SNPs in the region are shown). For each gene region, two or more plots are shown depending on the number of risk SNPs in the region, one for each risk SNP. The points are coloured according to LD with the risk SNP listed in the panel title (*r*^2^>0.5, red; between 0.2–0.5, green; and ≤0.2, blue). The eQTL result for the PrCa-risk SNP listed in the panel title is displayed as diamond, the data points for all of the other PrCa-risk SNPs are displayed as an open circle.

In addition, 10 of these 51 regions had multiple reported risk SNPs in close proximity, ranging from 2 to 5 risk SNPs per region (2 risk SNPs in 7 regions, 3 risk SNPs in 2 regions and 5 risk SNPs in 1 region). The LD between the risk SNPs varied, ranging from an *r*^2^ of <0.2 to 1 (15 with an *r*^2^ of <0.25, 3 from 0.5 to 0.7 and 4 from 0.7 to 1.0). Examples for 4 of the regions where *r*^2^ was <0.7 among the risk SNPs are shown in Figs 2 and 3 (*r*^2^=0.54 for the risk SNPs in the *GGCX* region, *r*^2^<0.2 for those associated with *BMPR1B*, *r*^2^=0.67 for *HNF1B* and *r*^2^<0.25 within the *CHMP2B* region). For *GGCX* (Fig. 2), the data indicate that the eQTL signal is driven largely by the SNPs in high LD with the risk SNP rs10187424 (lower left panel). The risk SNP rs2028898 (upper left panel) is not likely to be an independent risk factor, as it is in low LD with rs10187424. *BMPR1B* (Fig. 2) demonstrates a more complex region. The peak eQTL signal is in high LD with the risk SNP rs12500426 (upper middle panel). The risk SNP rs17021918 (lower middle panel), which is not in LD with rs12500426 (*r*^2^<0.2), shows another cluster of high-LD-SNPs that have a significant eQTL signal, yet are in moderate to low LD with the peak signal. These data suggest the presence of two independent regulatory domains, each tagged by the two reported risk SNPs. Finally, there is a third cluster of significant eQTL signals that are not in LD with either of the two reported risk SNPs (*r*^2^<0.2 for both, blue points clustered around 96 Mb), suggesting a third independent regulatory domain for *BMPR1B*. Both *HNF1B* (Fig. 2) and *CHMP2B* (Fig. 3) demonstrate the presence of reported risk SNPs that are not in LD with each other and where the eQTL signal is driven by one of the risk-SNP clusters. Thus, in each case, there is no eQTL signal for one (*HNF1B*) or more (*CHMP2B*) of the reported risk SNPs.

**Figure 4:**
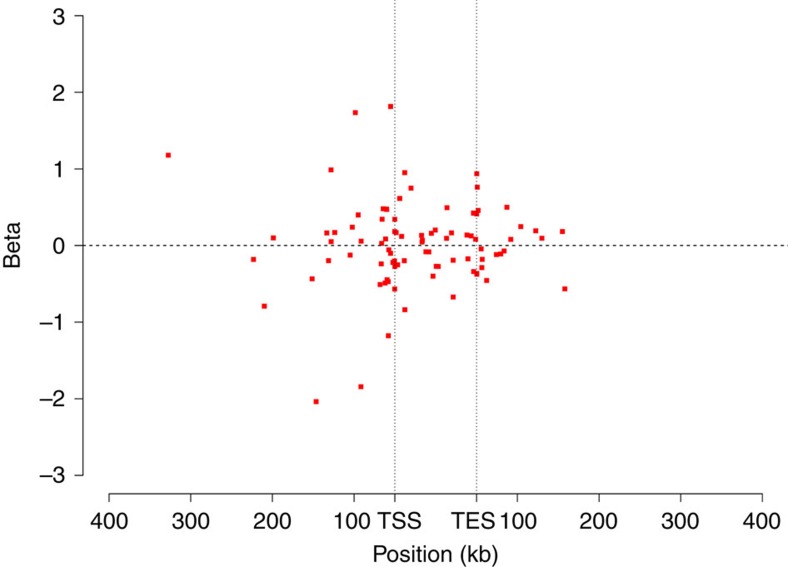
Positional distribution of peak *cis*-eQTLs and their effect size. Each point represents the peak SNP for each significant target gene (*N*=88). The β-coefficient obtained by regressing normalized expression levels for each target gene on the number of minor alleles of each SNP genotype adjusted for histologic characteristics and 14 expression principal components is plotted on the *y* axis and the SNP distance from the TSS or TES on the *x* axis. For display purposes, the distance between the TES and TSS was normalized to be 100 kb.

We then examined the positional distributions and the magnitude of gene dysregulation for each of the peak eQTLs relative to the TSS and TES. In general, we observed a high density of the peak eQTLs in proximity to the TSS and TES of the target genes, with 53 of the 88 (60%) significant peak eQTL signals within 20 kb of at least one of these positions (Fig. 4). However, the distance from the TSS to the peak eQTL signal varied considerably (Fig. 4), ranging from 57 bp to a higher value of ∼1 Mb (not shown). Also shown in Fig. 4 is the magnitude of the eQTL effects (that is, the absolute level of expression differences associated with the SNP genotypes). Generally, those eQTLs associated with larger differences in gene expression clustered near the TSS and TES, while those eQTLs associated with smaller differences were observed further away.

For the purpose of fine-mapping causative SNPs and candidate regulatory elements, we estimated the minimum region within the peak eQTL signal that could contain such elements. The estimated minimal region was based on a visual inspection of the regional association plots (Supplementary Figs 2–4), focusing on the most narrow portion of the peak eQTL signal taking into account the LD structure in that region. The distribution of the estimated minimal size for each of the 88 regions of interest is shown in Supplementary Fig. 5. Overall, 54 (61%) of the peak eQTL signals were <∼50 kb; the remainder ranged from ∼50 to almost 350 kb in size. Examples where the regulatory domains were mapped for two of the target genes (*CTBP2* and *ASCL2*) are shown in Supplementary Figs 6 and 7 and Supplementary Data 3 and 4.

## Discussion

In this study, we identified 88 genes as potential candidates for PrCa risk. The two-stage analysis was critical, as not all PrCa-risk SNPs fell within the peak eQTL signal identified (Supplementary Figs 2–4). In fact, for many of the regions, utilizing the PrCa-risk and LD-SNPs alone would not have been sufficient to map the entire eQTL peak (Supplementary Figs 2–4).

For some of the associations, the overlap between the risk SNP and the eQTL signals may represent a chance overlap, as over two-thirds of transcripts in normal prostate tissue have eQTLs associated with them^22^. If these were the case, then these eQTL analyses may lead to false-positive assignments of target genes as PrCa susceptibility genes. To explore this issue in more detail, we grouped the 88 regions of interest into three separate categories based on the LD between the PrCa-risk SNP and the peak eQTL signal (Supplementary Figs 2–4). The LD between the PrCa-risk SNP and the peak eQTL signal was set at an *r*^2^ of >0.5 for group 1 (high LD), 0.2–0.5 for group 2 (moderate LD) and<0.2 for group 3 (low LD).

For group 1 (37 candidate genes, [Supplementary Fig. 2]), there was a complete overlap between the risk SNP and the eQTL signal. The risk SNP and the LD-SNPs utilized in the primary analysis provided the most significant *P* values, and the eQTL signals were generally categorized by very narrow regional association plots (minimal region). For 25 of the 37 group 1 genes, no SNPs (including the original risk SNP) demonstrated a significant eQTL signal after adjustment for the peak eQTL signal. Overall, the candidate genes identified in this group most likely represent true signals for their association with PrCa risk, assuming the identified PrCa-risk SNP is also a true-positive signal.

For group 3 (34 candidate genes, [Supplementary Fig. 4]), the peak eQTL signals were derived primarily from the second-stage analysis. In this group, the LD between the PrCa-risk SNPs and the peak eQTL signals are low, having an *r*^2^ of <0.2. Unlike group 1, the risk SNPs in this group had minimal overlap with the peak eQTL signal in over half of the regions identified, and in several cases, the risk SNP was localized hundreds of kb from the peak eQTL signal. There are several possible explanations for these findings. First, some of these regions could be the result of a chance overlap between the eQTL signal and the risk SNP. The target gene identified, therefore, may not necessarily be associated with PrCa risk (false positive for PrCa). A second possibility is that there are several independent regulatory regions for the same gene, and a PrCa-risk SNP was identified for only one of them. If this was the case, however, and if the gene was truly associated with PrCa risk, then one might have expected the discovery of PrCa-risk SNPs within the peak eQTL region. Although we are not aware of such risk SNPs in these regions, their discovery is dependent on having adequate SNP coverage in the GWAS performed. To distinguish between the possibility of a chance overlap of the eQTL signal and a true association with PrCa, more detailed association studies ensuring both adequate SNP coverage and adequate statistical power in these regions of interest would be required. A negative finding for a PrCa-risk SNP in the main eQTL peak may suggest that the candidate gene is of less interest, while a positive finding may argue for its importance in PrCa risk. Of note, these eQTL signals may be good candidates for PrCa-risk-SNP discovery.

Group 2 (17 candidate genes, Supplementary Fig. 3) represents an intermediate group, where the LD between the PrCa-risk SNP and the peak eQTL signal is between an *r*^2^ of 0.2 and 0.5. Although these regions share many of the characteristics of group 1, the peak eQTL signals for this group were derived primarily from the second-stage analyses. For over half, the peak eQTL region and risk SNP overlapped. Given that the risk SNP is a tag SNP, the findings in this category are expected. The majority of candidate genes identified in this group most likely represent true signals for their association with PrCa risk.

In the primary analysis, all SNPs in LD with the PrCa-risk SNPs (*r*^2^>0.5) were used to identify potential eQTL signals and candidate genes. If the LD cutoff had been more stringent, several of the genes initially identified would not have met our threshold for detection. Of the 88 target genes initially identified, 12, 16 and 22 genes would not have been selected if the LD cutoff had been set to an *r*^2^ of 0.7, 0.8 and 0.9, respectively. Although using a more stringent LD cutoff might have helped to reduce potential false-positive signals for PrCa susceptibility genes, some true-positive candidate genes might also have been lost. Although generally robust, another potential confounder that has been reported to lead to false-positive eQTL signals for a small number of genes is allelic mapping bias in RNA sequencing^23^. One of the target genes identified in our study, *HLA-DRB5*, has been reported to be affected by this bias and thus may be a false-positive signal^23^. In the end, however, laboratory studies will be required to confirm that the target genes identified by these studies are in fact PrCa susceptibility genes regardless of which group these were placed in.

To explore the possibility of multiple independent regulatory regions, we performed conditional eQTL analysis adjusting for the peak eQTL signal for each target gene. The data suggest the presence of a single regulatory domain for half of the target genes. Importantly, the remaining half of the target genes did show evidence for one or more independent regulatory domains, some of which were observed among SNPs in high LD with the PrCa-risk SNP. It is important to note that the classification of each gene as having a single or multiple regulatory domains is based on the conditional eQTL Bonferroni threshold, and thus is likely conservative. For that reason, we also applied a recently developed fine-mapping method that uses marginal test statistics and correlations among SNPs in a Bayesian framework (CAVIARBF^24^) which may help to interpret the results for some genes. CAVIARBF estimates a posterior inclusion probability for each SNP. When summed, this provides an estimate of the expected number of causal variants in each region. The average expected number of causal variants across the 88 target genes was 2.82 (range 1.72–3.0). Cumulatively, these data provide evidence for the presence of multiple independent regulatory domains for some of the candidate genes, highlighting the complexity of gene regulation and dysregulation at these sites. Unfortunately, our results do not help to identify the causative SNPs themselves, primarily due to LD in the regions. The data do point, however, to the possible number of regulatory domains and their possible general positions.

A number of candidate regions (*n*=10) demonstrating an eQTL signal had several PrCa-risk SNPs in close proximity. The LD for several of the risk-SNP pairs was high (4 with *r*^2^>0.7) and, as a result, the additional SNPs do not add useful information, both appearing to map to the same regulatory domain. For many of the risk-SNP pairs, however, the LD between them was quite low (15 with *r*^2^<0.25). These present an opportunity to examine the more complex nature for several of the regulatory regions. Examples of these are provided in Figs 2 and 3. *BMPR1B* demonstrates a particularly interesting case in point. Overall, the data suggest the presence of three independent regulatory domains. Two of these are each tagged by one reported risk SNP (rs12500426 for one domain, red cluster in the upper panel, and rs17021918 for the other domain, red cluster in the lower panel). A third domain is suggested by the presence of a third cluster of significant eQTL signals that are not in LD with either of the two reported risk SNPs (blue cluster to right of both red domains). This latter region would be ideal for the testing and discovery of additional PrCa-risk SNPs. In another example, both *HNF1B* and *CHMP2B* demonstrate the presence of reported risk SNPs that are not in LD with each other and where the eQTL signal is driven by one of the risk-SNP clusters. Thus, in each case, there are reported risk SNPs that are not associated with an eQTL signal. Assuming that the reported risk SNPs are not a false positive, then these data suggest the presence of other unique mechanisms for PrCa risk that are associated with these risk SNPs.

For each of the eQTL signals in the risk regions, the β-coefficient obtained from the regression analysis provides the direction of the RNA expression relative to the alternative (or minor allele) (Supplementary Data 1). Both up-and down-dysregulation changes were observed, and as such, may provide some insights into the functional significance of regulatory domains as well as the mechanism of action of the various candidate genes. However, future studies will be needed to define the role and mechanism by which these candidate genes are involved in PrCa aetiology and/or progression.

Co-regulation of two or more genes from a common eQTL region was a common finding. Several eQTLs were characterized by association with multiple genes, many with direction and magnitude of regulatory effect similar across the dysregulated genes. The common presence of *cis*-acting co-regulatory modules has been previously described^25^. These data suggest a more complex biology for many of the PrCa-risk SNPs, that is, a one-to-many relationship. This finding also adds an additional layer of complexity in determining which of the co-regulated genes (if not all) is truly associated with PrCa risk.

A number of eQTL studies testing for SNP–gene expression associations for PrCa-risk SNPs have now been published (Supplementary Fig. 8)^26^,^27^,^28^. Grisanzio *et al.*^26^ tested 12 risk SNPs (mass spectrometry) and all expressed genes (NanoString nCounter) within ±0.5 Mb of the risk SNP (all included in our study) with 407 normal prostate tissue samples. They found five significant SNP–gene associations (*P*<0.001) among four risk SNPs, all of which were replicated in our study. These candidate target genes identified include *SLC22A3* (chr6), *NCOA4*, *MSMB* (chr10), *HNF1B* (chr17) and *NUDT11* (chrX). Of the remaining eight risk SNPs without significant signals, they found nine genes among four SNPs that were at least nominally significant (*P*<0.05). Of these, two genes were significantly associated in our study; *BHLHA15* (chr7) and *CTBP2* (chr10). Of the four risk SNPs with no associated genes, we found a significant gene association for two of these, and finally, no associations were identified by either study for the final two risk SNPs.

Xu *et al.*^28^ tested 51 risk SNPs (Affymetrix SNP 6.0 array) and all expressed genes (RNAseq) within ±1.0 Mb of the risk SNP (all included in our study) with 50 PrCa tumour samples. They found 14 significant SNP–gene associations (*P* value <1.41E-02) among seven risk SNPs, five of which were replicated in our study. These candidate target genes include *IRX4* (chr5), *PP1R14A* (chr19), *NUDT11* (chrX), *NCOA4* (chr10) and *FOXP4* (chr6). We found no significant eQTL signals for nine of the genes identified by this group; all nine genes were expressed in our normal tissue samples. Of the remaining 44 risk SNPs with no eQTL signals, we found a significant gene association with 12 of the risk-SNP regions.

Finally, Li *et al.*^27^ tested 69 risk SNPs (Affymetrix SNP 6.0 array) and all expressed genes (RNAseq) within ±0.5 Mb of the SNP with 145 PrCa tumour samples. They found 30 significant (*P* value <0.05) SNP–gene associations, 13 of which we were able to replicate. However, for the remaining 17 signals, we found eQTL associations for 6 of these but with different genes and no signal for 11. All but three of these genes were expressed in our normal tissue samples. Li *et al.* did not provide the list of risk SNPs evaluated, so we are not able to comment on the remaining 39 risk SNPs with no eQTL signals.

In total, 19 candidate genes were identified in two or more studies (Supplementary Fig. 8), only 1 of which was common to all of these (*NCOA4*). If confined to regions common to each of the studies, then we were able to identify 28 additional potential targets, while Xu *et al.* and Li *et al.* identified 9 and 17 additional targets, respectively. If we consider all regions evaluated, then ∼69 additional novel candidate targets have been identified. However, as noted earlier, this very likely represents an overestimate, as some of these are likely to be false-positive signals due to the overlap of PrCa-risk SNPs and eQTL signals by chance (for example, group 3 eQTL signals).

For the purpose of candidate gene discovery by eQTL analysis, the use of tissue relevant to the disease being studied will be important, in this case prostate tissue. However, we recognize that this issue has not been well studied, particularly for genes expressed in prostate tissue. To address this question, we compared the eQTL results from our study with those derived from lymphocytic cell lines, the most common source of cells for the development of eQTL data sets. We first cross-referenced our peak eQTLs with the transcriptome-wide significant (false discovery rate =0.05) findings from the Geuvadis Consortium (GEUV)^29^. In the Geuvadis study, *cis*-eQTL analysis was performed with lymphocytic cell lines from 373 individuals of European descent in the 1000 Genomes Project. This data set is comparable to ours, both in variant density (sequencing data compared with our data imputed to 1000 Genomes) and in sample size. The GEUV results constitute 419,983 significant *cis*-eQTLs for 3,259 genes. Of the 88 genes identified in our peak eQTL analysis, only 23 (26.1%) were present in the GEUV results. Of these, 13 corresponded to an eQTL SNP we identified in our analysis, resulting in only 13/95 (13.7%) SNP–gene eQTL pairs from our analysis reproduced in the lymphocytic cell lines-based eQTL study. These data highlight the importance of testing tissue-specific eQTL data sets for the identification of disease-specific candidate genes. If we had used a lymphocytic cell lines based eQTL data set, we would have missed the majority of eQTL signals detected in our study.

Although this study does not identify the regulatory regions or the causal SNPs associated with the gene dysregulation, our eQTL analysis does provide critical fine mapping information necessary for those studies. Importantly, many of these regions are narrowly defined, enabling the identification of candidate causal regulatory elements. A number of bioinformatics tools and a wealth of publically available data sets now provide a unique opportunity to map and identify candidate regulatory elements, transcription factor binding sites and candidate causal variants within the peak eQTL signals.

An example of a well-studied region is for the PrCa-risk SNP rs4962416 and its associated gene *CTBP2*. In our study, we identified 24 SNPs in the peak eQTL region associated with dysregulation of *CTBP2* (Supplementary Fig. 6 and Supplementary Data 3). Of these, 15 map to a probable prostate-specific enhancer region as defined by both Hazelett *et al.*^30^ and Taberlay *et al.*^31^ One of the top variants (rs12769019), as determined by Regulome, is found in an androgen receptor (AR)-binding site (Chip-seq) and is predicted to possibly disrupt the binding affinities of several transcription factors that could be important for PrCa regulation through interactions with the AR (POU6F1 and POU2F2). Importantly, Takayama *et al.*^32^ found altered enhancer activity using a luciferase assay when the alleles at rs12769019 and rs4962416 were tested in LnCap cells. An example of a novel region identified in the current study is for the PrCa-risk SNP rs7127900 and its associated gene *ASCL2* (Supplementary Fig. 7 and Supplementary Data 4). In this case, the peak eQTL region contained 54 significant eSNPs, many of which are also found in enhancer regions and associated with possible binding motifs. In this case, the SNP with the best Regulome score (rs7123299) was shown to reside within a FOXA1-binding motif. As with *CTBP2*, these data provide the insights necessary to identify target regulatory elements and motifs and to perform subsequent functional studies.

Overall, we believe that the results of our study represent the most exhaustive published to date. We have included all known published PrCa-risk SNPs, we have utilized an extremely dense set of SNPs (2.5M Illumina array plus imputation) to map the regions of interest, and we have used RNAseq to test all possible transcripts in the regions. On the basis of this work, we identified 88 target genes that can now be prioritized for future study as candidate PrCa susceptibility genes. However, additional studies will be needed to identify those genes among the 88 that are truly involved in PrCa risk and to define their mechanism of action. Finally, it is noteworthy to mention that an eQTL signal was not identified for 49 of the 100 risk regions, and for some of the risk SNPs in the 51 positive regions. Clearly, additional work will be required to identify the mechanism by which the risk SNPs in these regions increase risk for PrCa.

## Methods

### Case selection

Informed consent was obtained from all subjects; the study was approved by the Mayo Clinic Institutional Review Board. Normal prostate tissue was acquired from an archive collection of fresh frozen material obtained from patients with either radical prostatectomy or cystoprostatectomy. From this collection, the initial surgical haematoxylin and eosin (H&E) section from over 4,000 cases were reviewed to identify normal tissue samples where prostate tumour was not present on the archived slide and where the Gleason score was ≤7 for the presenting tumour. From 916 cases meeting these criteria, a new H&E slide was prepared from each normal tissue sample and re-examined to select samples with the following characteristics: (1) absence of PrCa; (2) absence of high-grade prostatic intraepithelial neoplasia and benign prostatic hyperplasia; (3) normal prostatic epithelial glands representing ≥40% of all cells; (4) lymphocytic population representing ≤2% of all cells; and (5) the normal epithelium was from the posterior region of the prostate (region most consistent with PrCa). Of these, 565 cases met these inclusion criteria and were eligible for DNA and RNA processing.

### Tissue processing

Frozen tissue samples were processed to obtain sections for RNA and DNA extraction, with an H&E section obtained at the beginning, between the RNA and DNA designated section, and at the end of the sectioning. All H&E sections were re-evaluated by a pathologist utilizing the same inclusion criteria described above. Fifty samples no longer met those criteria and were excluded, resulting in 515 eligible cases. DNA was extracted using the Puregene tissue extraction protocol per the manufacturer’s recommendations. DNA quality was assessed by examining 260/280 ratio and DNA yield. RNA was extracted using the QIAGEN miRNeasy Mini Kit and the QIAcube instrument in accordance with the manufacturer’s instructions. RNA quality was assessed by evaluating the RNA integrity number (≥7) and the 260/280 ratio. Twenty one cases were eliminated due to poor quality, yielding 494 samples eligible for DNA genotyping and RNA sequencing.

To minimize potential batch effects in the final analyses, randomization for sample processing was performed at three stages. Samples were first randomized for tissue cutting with respect to Gleason score (≤6 versus 7), percent epithelium present (<60% versus ≥60%), presence of inflammatory cells (yes/no) and procedure type (cryoprostatectomy versus radical prostatectomy). Sample categories with a larger number of samples were also balanced across tissue cut groups. After tissue cutting, samples were re-randomized for extraction of DNA and RNA considering the tissue cut group in addition to the sample characteristics described above. Finally, after extracting both DNA and RNA, samples were randomized for a final time for RNA library preparation and DNA plating. In this stage, we considered the RNA extraction randomization group, Gleason score (≤6 versus 7) and 260/280 ratio (<1.9 versus ≥1.9) when randomizing RNA samples to 96-well plates. DNA samples were randomly assigned to 96-well plates. After each stage of randomization, we fit one-way analysis of variance models to verify that randomization group was not associated with any sample characteristics or the year of sample collection.

### RNA sequencing

RNA libraries were prepared using the TruSeq RNA Sample Prep Kit v2 (Illumina, San Diego, CA) according to the manufacturer’s instructions. One sample failed library prep and was excluded from the study. The remaining 493 libraries (19 from patients with cryoprostatectomy and 474 from patients with radical prostatectomy) were loaded onto flow cells at concentrations of 8–10 pM to generate cluster densities of 700,000 mm^2^ following Illumina’s standard protocol using the Illumina cBot and cBot Paired-End Cluster Kit Version 3. The flow cells were run as 51 paired-end reads on an Illumina HiSeq 2000 using TruSeq SBS sequencing kit version 3 and HCS v2.0.12 data collection software. Base calling was performed using Illumina’s RTA version 1.17.21.3. A minimum of 50 million total reads per sample was required for analysis. A total of 234 samples with <50 million total reads were re-sequenced and if no quality issues were identified with the individual runs, BAM files were merged.

### RNA-sequencing analysis

RNAseq data were analysed with the use of the MAP-R-Seq pipeline, an integrated suite of open-source bioinformatics tools along with in-house developed methods^33^. Paired-end reads were aligned by TopHat 2.0.6 (ref. 34) against the hg19 genome build using the bowtie1 aligner option^35^. Gene counts were generated using HTseq software (http://www-huber.embl.de/users/anders/HTSeq/doc/overview.html)^36^ and gene annotation files were obtained from Illumina (http://cole-trapnell-lab.github.io/cufflinks/igenome_table/index.html). The MAP-R-Seq pipeline provides detailed quality control data to estimate the distance between paired-end reads, to evaluate the sequencing depth for alternate splicing analysis, to determine the rate of duplicate reads and to evaluate coverage of reads across genes using RSeQC software^37^.

### RNA-sequencing quality control and normalization

Gene counts were quantified for 23,398 genes based on RefSeq gene annotation. Of all genes, 780 (3.3%) had no counts for all samples and were removed from further analysis (genes deemed undetectable) leaving 22,618 for analysis. The remaining genes were distributed across all chromosomes. For genes mapping to both chromosomes X and Y, only the chromosome X version was retained.

Data were assessed for quality using graphical methods for assessing the existence and functional form of bias and the success of normalization^38^. The influence of flowcell, lane, tissue cut group, extraction group and library prep plate on global mRNA abundance shifts in the samples was evaluated by examining per-sample boxplots of log2 gene count across levels of these design factors. The influence of gene size and gene GC content on expression levels was also investigated. Finally, we assessed how individual gene counts differed from the average using residual MA plots.

We filtered out mRNA transcripts that had a median gene count <14, reducing the number of expressed genes to 17,233 for the final data analysis. To remove potential biases such as GC content and to account for differences in sequencing depth, the gene counts were normalized using conditional quantile normalization^39^.

### Genotyping

Samples (200 ng genomic DNA) were genotyped using Illumina Infinium 2.5M bead arrays based on the manufacturer’s protocol (Illumina, San Diego, CA). The DNA samples were plated into 96-well plates, with samples randomized to plates as described above. One Caucasian parent–child Centre d’Etude du Polymorphisme Humain (CEPH) trio from the HapMap project was included as duplicates for quality control.

Extensive quality control analyses were performed to identify any poor-quality samples or SNPs^40^. SNPs were assessed for quality via marker genotyping call rates, duplicate sample concordance and Hardy–Weinberg equilibrium (HWE) *P* values. Samples were assessed via sample genotyping call rates, checks for inconsistency with reported sex, and sample heterozygosity rates. To identify possibly related samples, we used the genome command in PLINK to calculate the proportion of loci where each pair of individuals share zero, one or two alleles identical by descent. We used the Structure software^41^,^42^ to address the issue of population stratification.

A total of 509 samples were genotyped, including 493 normal prostate tissue samples and 16 replicate CEPH samples. The overall concordance rate for each CEPH duplicate was >99.99%. Of the 493 normal prostate tissue samples, 22 were excluded after quality control of the genotypes; 5 samples due to low call rate (<95%), 10 samples were found to have a high proportion of African–American ancestry and 7 samples had low genotype concordance compared with mRNA called variants (concordance<98%). The final data set consisted of 471 normal prostate tissue samples (453 from low Gleason grade PrCa cases and 18 from cryoprostatectomy cases).

From the evaluation of individual called SNPs, a total of 12,588 SNPs (0.005%) were excluded for quality control reasons: 205 failed completely; 4,920 were duplicate SNPs (same physical location); 6,240 SNPs had call rate <95%; and 1,223 with Hardy–Weinberg equilibrium *P* value <1E-5. In addition, we excluded 817,800 SNPs (34.5%) with minor allele frequency<1% because they have low power for association analysis. Thus, the final quality control-passed data set consisted of 1,541,368 observed autosomal and chromosome X SNPs.

Untyped SNPs as well as missing genotypes for typed SNPs were imputed using SHAPEIT^43^ and IMPUTE2 (refs 43, 44) with reference files from the 1000 Genomes Phase I integrated variant set. SNPs on the Y chromosome and mitochondrial SNPs were excluded from imputation. Variants with minor allele frequency >0.5% in the cosmopolitan 1000 Genomes populations were retained in the reference panel. We assessed imputation quality using the allelic *r*-squared metric calculated using BEAGLE^45^ (https://faculty.washington.edu/browning/beagle_utilities/utilities.html) utilities (Version 3). Poorly imputed SNPs, defined as those with allelic *r*-squared ≤0.3, were excluded from further analysis. After imputation and quality filtering, we had a total of 10,856,681 variants available for analysis.

### Covariate identification and adjustment

Before performing eQTL analyses, histologic characteristics and PCs derived from the SNP correlation matrix were evaluated for their association with global transcript abundance using linear (for PC) or logistic (histologic characteristics percent lymphocytic population and percent epithelium present) regression. PC analysis of the SNP correlation matrix identified two eigenvalues with Tracy–Widom *P* values <0.05 (ref. 46). The association of PC1 and PC2 with genome-wide transcript abundance suggested that neither component appears to affect global expression (minimum linear regression *P* value >1E-5). Both histologic characteristics were shown to have large effects on gene expression and were included as covariates in the eQTL analysis. To account for latent sources of non-genetic variation in gene expression, we applied PC analysis to the normalized gene expression matrix, identifying 14 PCs for inclusion as covariates in the eQTL analysis, each having >1% of variation explained which cumulatively explain 57% of the total variation.

### PrCa-risk SNP eQTL—primary analysis

To define the set of SNPs analysed for each risk interval, we calculated the LD between each PrCa-risk SNP and all SNPs in the risk region. All SNPs with an *r*^2^>0.5 with any of the PrCa-risk SNPs (LD-SNP) were included in the analysis. eQTL analyses were conducted using the Matrix eQTL R library^6^. We evaluated the association of each LD-SNP with gene expression using linear regression methods, regressing normalized expression levels on the number of minor alleles of each SNP genotype, adjusted for histologic characteristics percent lymphocytic population and percent epithelium present, and 14 PC. A Bonferroni adjustment was used to determine statistical significance (threshold of 1.96E-07).

Because the variance and the mean of gene expression counts might be related, we fit Negative Binomial (NB) models using a generalized linear model framework to model the gene counts with conditional quantile normalization constants included as an offset term in the generalized linear model. Per-gene estimates of the over-dispersion parameter were computed with the edgeR package in R (ref. 47). Results compared with the linear model were essentially unchanged. Thus, for simplicity, we report only linear models.

### Gene-based eQTL—second stage

To define an evaluation range for the target gene *cis-*eQTL analysis that would capture the vast majority of underlying effects, we downloaded the HapMap lymphocytic cell lines eQTL meta-analysis results from the seeQTL database^48^. We then merged the *cis-*eQTL results with the *trans*-eQTL results where the associated SNP and gene co-occurred on the same chromosome. We characterized the empirical distribution of the minimum SNP distance from either the transcription start or stop site for all findings with a false discovery rate of 0.05 (ref. 49), and identified the 99th percentile to be ∼1.1 Mb. Thus, for each PrCa target gene, we evaluated the statistical association for each SNP within 1.1 Mb of the target gene using the same linear regression model used for the PrCa-risk-SNP analysis described above in the primary analysis. In addition, we excluded all LD-SNPs included in the initial PrCa-risk-SNP analysis. Analyses were conducted using the Matrix eQTL R library^6^. A Bonferroni adjustment was used to determine statistical significance (threshold of 3.02E-08). Regional association plots were generated using a combination of LocusZoom^50^ and locally written R functions with LD estimates obtained from PLINK v1.9 (https://www.cog-genomics.org/plink2) (ref. 51).

### Peak-conditioned eQTL

We evaluated the statistical association for each SNP in the target gene region regressing normalized expression levels on the number of minor alleles for each SNP genotype adjusting for the peak eQTL signal SNP in addition to the histologic characteristics and 14 expression PCs. A Bonferroni adjustment was used to determine conditional statistical significance (threshold of 3.02E-08).

### Mapping of regulatory elements

A variety of bioinformatics tools and publically available data sets were utilized to map functional regulatory elements to the peak eQTL regions identified for two genes of interest, *CTBP2* and *ASCL2* (Supplementary Figs 5 and 6). Prostate-specific mapping information was obtained from a custom track established by Hazelett *et al.*^30^ in the UCSC Genome Browser with the following data from the GEO database (http://www.ncbi.nlm.nih.gov/geo/). For areas of open chromatin, the DNase hypersensitivity tracks used were GSE32970 and GSE29692. The histone modification tracks used were as follows: H3K4me1 and H3K4me3 (GSE27823); and H3K27Ac (GSE51621) and H3K4me3 (GSM686935 and GSM503906). Transcription factors of interest analysed were CTCF (GSE33213), AR (GSE28219), NKX3-1 (GSM699633), TCF7L2 (GSE51621) and FOXA1 (GSM699634 and GSM699635).

Data from Taberlay *et al.*^31^, obtained from prostate-specific cell lines (PrEC and PC3), were utilized to identify tissue-specific chromatin states for the regions of interest. Taberlay *et al.* used Chip-seq to obtain data for H3K4me1, H3K27ac, H3K4me3, H3K27me3, CTCF and RNAPolII, and applied these data to the multivariate hidden Markov model ChromHMM^52^ to annotate the epigenome into nine chromatin states (poised promoter, promoter, promoter+CTCF, insulator, enhancer, enhancer+CTCF, transcribed, repressed and heterochromatin).

Bioinformatics tools used to map the regions of interest include FunciSNP (www.factorbook.org) and Regulome (http://regulome.stanford.edu/). By annotating SNPs with known and predicted regulatory elements, Regulome combines all of its sources to assign a causal score for a specific variant entered^53^. Scores of 1–3 have some evidence for being a causal variant for gene dysregulation while a score of 4–6 does not. Along with the score, the output provides transcriptional factor and histone modification binding sites, PWM, DNase and eQTL information.

## Additional information

**Accession codes:** The microarray and genotyping data have been deposited in the dbGaP database under accession code phs000985.v1.p1.

**How to cite this article:** Thibodeau, S. N. *et al.* Identification of candidate genes for prostate cancer-risk SNPs utilizing a normal prostate tissue eQTL data set. *Nat. Commun.* 6:8653 doi: 10.1038/ncomms9653 (2015).

## Supplementary Material

Supplementary Information Supplementary Figures 1-8 and Supplementary References

Supplementary Data 1 eSNP - gene pairs identified for the primary eQTL analysis in the PrCa risk regions of interest and Supplementary References

Supplementary Data 2 Number of SNPs and genes evaluated for each of the PrCa risk regions for the primary eQTL analysis

Supplementary Data 3 Summary of data on mapping regulatory elements and transcriptional binding sites obtained from the literature1-3 or bioinformatics tools (e.g., Regulome, FunciSNP) and Supplementary References

Supplementary Data 4 Summary of data on mapping regulatory elements and transcriptional binding sites obtained from the literature1-3 or bioinformatics tools (e.g., Regulome, FunciSNP) and Supplementary References

## References

[b1] Siegel R., Miller K. D. & Jemal A. Cancer statistics, 2015. CA Cancer J. Clin. 65, 5–29 (2015). 25559415 10.3322/caac.21254

[b2] Knudsen B. S. & Vasioukhin V. Mechanisms of prostate cancer initiation and progression. Adv. Cancer Res. 109, 1–50 (2010). 21070913 10.1016/B978-0-12-380890-5.00001-6

[b3] Schaid D. J. The complex genetic epidemiology of prostate cancer. Hum. Mol. Genet. 1, R103–R121 (2004). 10.1093/hmg/ddh072 14749351

[b4] Witte J. S. Prostate cancer genomics: towards a new understanding. Nat. Rev. Genet. 10, 77–82 (2009). 19104501 10.1038/nrg2507 PMC2721916

[b5] Duggan D. *et al.* Two genome-wide association studies of aggressive prostate cancer implicate putative prostate tumor suppressor gene DAB2IP. J. Natl Cancer Inst. 99, 1836–1844 (2007). 18073375 10.1093/jnci/djm250

[b6] Easton D. F. & Eeles R. A. Genome-wide association studies in cancer. Hum. Mol. Genet. 17, R109–R115 (2008). 18852198 10.1093/hmg/ddn287

[b7] Eeles R. A. *et al.* Identification of seven new prostate cancer susceptibility loci through a genome-wide association study. Nat. Genet. 41, 1116–1121 (2009). 19767753 10.1038/ng.450 PMC2846760

[b8] Gudmundsson J. *et al.* Genome-wide association study identifies a second prostate cancer susceptibility variant at 8q24. Nat. Genet. 39, 631–637 (2007). 17401366 10.1038/ng1999

[b9] Gudmundsson J. *et al.* Common sequence variants on 2p15 and Xp11.22 confer susceptibility to prostate cancer. Nat. Genet. 40, 281–283 (2008). 18264098 10.1038/ng.89 PMC3598012

[b10] Gudmundsson J. *et al.* Two variants on chromosome 17 confer prostate cancer risk, and the one in TCF2 protects against type 2 diabetes. Nat. Genet. 39, 977–983 (2007). 17603485 10.1038/ng2062

[b11] Kote-Jarai Z. *et al.* Multiple novel prostate cancer predisposition loci confirmed by an international study: the PRACTICAL Consortium. Cancer Epidemiol. Biomarkers Prev. 17, 2052–2061 (2008). 18708398 10.1158/1055-9965.EPI-08-0317 PMC2776652

[b12] Thomas G. *et al.* Multiple loci identified in a genome-wide association study of prostate cancer. Nat. Genet. 40, 310–315 (2008). 18264096 10.1038/ng.91

[b13] Welter D. *et al.* The NHGRI GWAS Catalog, a curated resource of SNP-trait associations. Nucleic Acids Res. 42, D1001–D1006 (2014). 24316577 10.1093/nar/gkt1229 PMC3965119

[b14] Yeager M. *et al.* Genome-wide association study of prostate cancer identifies a second risk locus at 8q24. Nat. Genet. 39, 645–649 (2007). 17401363 10.1038/ng2022

[b15] Nicolae D. L. *et al.* Trait-associated SNPs are more likely to be eQTLs: annotation to enhance discovery from GWAS. PLoS Genet. 6, e1000888 (2010). 20369019 10.1371/journal.pgen.1000888 PMC2848547

[b16] Albert F. W. & Kruglyak L. The role of regulatory variation in complex traits and disease. Nat. Rev. Genet. 16, 197–212 (2015). 25707927 10.1038/nrg3891

[b17] Dixon A. L. *et al.* A genome-wide association study of global gene expression. Nat. Genet. 39, 1202–1207 (2007). 17873877 10.1038/ng2109

[b18] Spielman R. S. *et al.* Common genetic variants account for differences in gene expression among ethnic groups. Nat. Genet. 39, 226–231 (2007). 17206142 10.1038/ng1955 PMC3005333

[b19] Stranger B. E. *et al.* Population genomics of human gene expression. Nat. Genet. 39, 1217–1224 (2007). 17873874 10.1038/ng2142 PMC2683249

[b20] GTEx Consortium. The Genotype-Tissue Expression (GTEx) project. Nat. Genet. 45, 580–585 (2013). 23715323 10.1038/ng.2653 PMC4010069

[b21] Melé M. *et al.* Human genomics. The human transcriptome across tissues and individuals. Science 348, 660–665 (2015). 25954002 10.1126/science.aaa0355 PMC4547472

[b22] Larson N. B. *et al.* Comprehensively evaluating cis-regulatory variation in the human prostate transcriptome by using gene-level allele-specific expression. Am. J. Hum. Genet. 96, 869–882 (2015). 25983244 10.1016/j.ajhg.2015.04.015 PMC4457953

[b23] Panousis N. I., Gutierrez-Arcelus M., Dermitzakis E. T. & Lappalainen T. Allelic mapping bias in RNA-sequencing is not a major confounder in eQTL studies. Genome Biol. 15, 467 (2014). 25239376 10.1186/s13059-014-0467-2 PMC4212091

[b24] Chen W. *et al.* Fine mapping causal variants with an approximate Bayesian method using marginal test statistics. Genetics 200, 719–736 (2015). 25948564 10.1534/genetics.115.176107 PMC4512539

[b25] Battle A. *et al.* Characterizing the genetic basis of transcriptome diversity through RNA-sequencing of 922 individuals. Genome Res. 24, 14–24 (2014). 24092820 10.1101/gr.155192.113 PMC3875855

[b26] Grisanzio C. *et al.* Genetic and functional analyses implicate the NUDT11, HNF1B, and SLC22A3 genes in prostate cancer pathogenesis. Proc. Natl Acad. Sci. USA 109, 11252–11257 (2012). 22730461 10.1073/pnas.1200853109 PMC3396469

[b27] Li Q. *et al.* Expression QTL-based analyses reveal candidate causal genes and loci across five tumor types. Hum. Mol. Genet. 23, 5294–5302 (2014). 24907074 10.1093/hmg/ddu228 PMC4215106

[b28] Xu X. *et al.* Variants at IRX4 as prostate cancer expression quantitative trait loci. Eur. J. Hum. Genet. 22, 558–563 (2014). 24022300 10.1038/ejhg.2013.195 PMC3953920

[b29] Lappalainen T. *et al.* Transcriptome and genome sequencing uncovers functional variation in humans. Nature 501, 506–511 (2013). 24037378 10.1038/nature12531 PMC3918453

[b30] Hazelett D. J. *et al.* Comprehensive functional annotation of 77 prostate cancer risk loci. PLoS Genet. 10, e1004102 (2014). 24497837 10.1371/journal.pgen.1004102 PMC3907334

[b31] Taberlay P. C. *et al.* Reconfiguration of nucleosome-depleted regions at distal regulatory elements accompanies DNA methylation of enhancers and insulators in cancer. Genome Res. 24, 1421–1432 (2014). 24916973 10.1101/gr.163485.113 PMC4158760

[b32] Takayama K. *et al.* CtBP2 modulates the androgen receptor to promote prostate cancer progression. Cancer Res. 74, 6542–6553 (2014). 25228652 10.1158/0008-5472.CAN-14-1030

[b33] Kalari K. R. *et al.* MAP-RSeq: Mayo analysis pipeline for RNA sequencing. BMC Bioinformatics 15, 224 (2014). 24972667 10.1186/1471-2105-15-224 PMC4228501

[b34] Langmead B., Trapnell C., Pop M. & Salzberg S. L. Ultrafast and memory-efficient alignment of short DNA sequences to the human genome. Genome Biol. 10, R25 (2009). 19261174 10.1186/gb-2009-10-3-r25 PMC2690996

[b35] Kim D. & Salzberg S. L. TopHat-Fusion: an algorithm for discovery of novel fusion transcripts. Genome Biol. 12, R72 (2011). 21835007 10.1186/gb-2011-12-8-r72 PMC3245612

[b36] Anders S., Pyl P. T. & Huber W. HTSeq—a Python framework to work with high-throughput sequencing data. Bioinformatics 31, 166–169 (2015). 25260700 10.1093/bioinformatics/btu638 PMC4287950

[b37] Wang L., Wang S. & Li W. RSeQC: quality control of RNA-seq experiments. Bioinformatics 28, 2184–2185 (2012). 22743226 10.1093/bioinformatics/bts356

[b38] Dudoit S., Yang Y. H., Callow M. J. & Speed T. P. Statistical methods for identifying differentially expressed genes in replicated cDNA microarray experiments. Stat. Sinica 12, 111–139 (2002).

[b39] Shabalin A. A. Matrix eQTL: ultra fast eQTL analysis via large matrix operations. Bioinformatics 28, 1353–1358 (2012). 22492648 10.1093/bioinformatics/bts163 PMC3348564

[b40] Turner S. *et al.* Quality control procedures for genome-wide association studies. Curr. Protoc. Hum. Genet. Chapter 1, Unit 1.19 (2011). 10.1002/0471142905.hg0119s68 PMC3066182 21234875

[b41] Patterson N., Price A. L. & Reich D. Population structure and eigenanalysis. PLoS Genet. 2, e190 (2006). 17194218 10.1371/journal.pgen.0020190 PMC1713260

[b42] Price A. L. *et al.* Principal components analysis corrects for stratification in genome-wide association studies. Nat. Genet. 38, 904–909 (2006). 16862161 10.1038/ng1847

[b43] Delaneau O., Zagury J. F. & Marchini J. Improved whole-chromosome phasing for disease and population genetic studies. Nat. Methods 10, 5–6 (2013). 23269371 10.1038/nmeth.2307

[b44] Howie B. *et al.* Fast and accurate genotype imputation in genome-wide association studies through pre-phasing. Nat. Genet. 44, 955–959 (2012). 22820512 10.1038/ng.2354 PMC3696580

[b45] Browning B. L. & Browning S. R. A unified approach to genotype imputation and haplotype-phase inference for large data sets of trios and unrelated individuals. Am. J. Hum. Genet. 84, 210–223 (2009). 19200528 10.1016/j.ajhg.2009.01.005 PMC2668004

[b46] Tracy C. A. & Widom H. in Proceedings of the International Congress of Mathematicians: Beijing August 20–28 2002 (International Congress of Mathematicians//Proceedings) Vol. 1, ed. Tatsien L. I. 587–596Higher Education Press (2002).

[b47] Robinson M. D., McCarthy D. J. & Smyth G. K. edgeR: a Bioconductor package for differential expression analysis of digital gene expression data. Bioinformatics 26, 139–140 (2010). 19910308 10.1093/bioinformatics/btp616 PMC2796818

[b48] Xia K. *et al.* seeQTL: a searchable database for human eQTLs. Bioinformatics 28, 451–452 (2012). 22171328 10.1093/bioinformatics/btr678 PMC3268245

[b49] Benjamini Y. & Hochberg Y. Controlling the false discovery rate—a practical and powerful approach to multiple testing. J. R. Stat. Soc. Ser. B 57, 289–300 (1995).

[b50] Pruim R. J. *et al.* LocusZoom: regional visualization of genome-wide association scan results. Bioinformatics 26, 2336–2337 (2010). 20634204 10.1093/bioinformatics/btq419 PMC2935401

[b51] Chang C. C. *et al.* Second-generation PLINK: rising to the challenge of larger and richer datasets. GigaScience 4, 7 (2015). 25722852 10.1186/s13742-015-0047-8 PMC4342193

[b52] Ernst J. *et al.* Mapping and analysis of chromatin state dynamics in nine human cell types. Nature 473, 43–49 (2011). 21441907 10.1038/nature09906 PMC3088773

[b53] Boyle A. P. *et al.* Annotation of functional variation in personal genomes using RegulomeDB. Genome Res. 22, 1790–1797 (2012). 22955989 10.1101/gr.137323.112 PMC3431494

