# Supplementary material for: Identification of candidate genes for prostate cancer-risk SNPs utilizing a normal prostate tissue eQTL data set
Source: Nat Commun. 2015 Nov 27;6:8653. doi: 10.1038/ncomms9653 (PMC4663677; doi:10.1038/ncomms9653)
Supplement: Supplementary Information — Supplementary Figures 1-8 and Supplementary References [file ncomms9653-s1.pdf]

Supplementary Figure 1 - Distribution of RNA-seq gene counts for each LD risk-region

Each page shows the distribution of RNA-seq gene counts on a log2 scale for all genes in each of the risk-regions [the log2(raw gene count + 1) is plotted to account for samples having 0 reads]. All of the genes are presented in chromosomal order. Genes included in our eQTL analysis are colored in red (median raw gene count $\geq$ 14) while those genes excluded due to low expression are colored in gray. The rs number in the title is the reported risk-SNP from prior GWAS studies in the region examined.

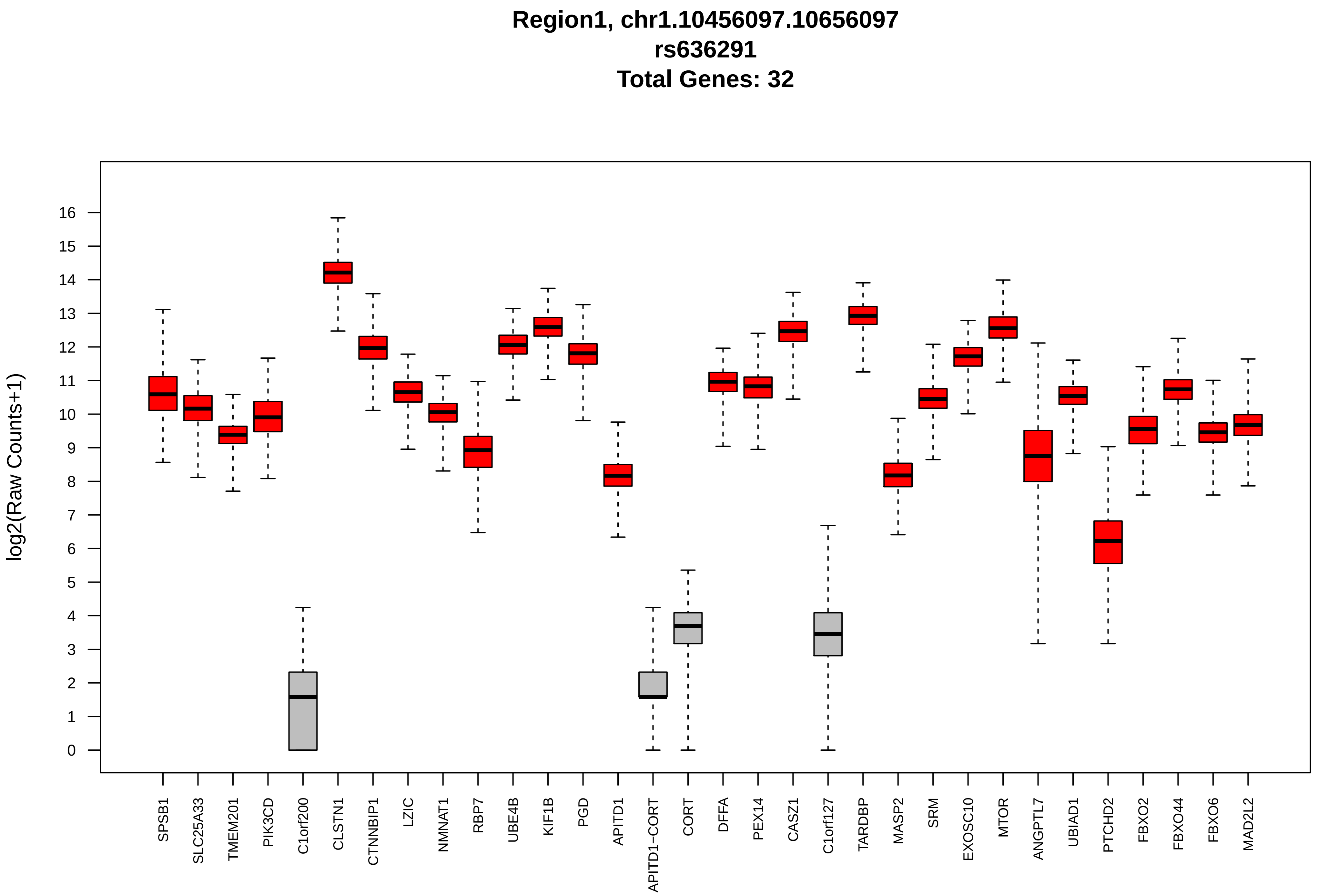
Region1, chr1.10456097.10656097
rs636291
Total Genes: 32
log2(Raw Counts+1)
0
1
2
3
4
5
6
7
8
9
10
11
12
13
14
15
16
SPSB1
SLC25A33
TMEM201
PIK3CD
C1orf200
CLSTN1
CTNNBIP1
LZIC
NMNAT1
RBP7
UBE4B
KIF1B
PGD
APITD1
APITD1−CORT
CORT
DFFA
PEX14
CASZ1
C1orf127
TARDBP
MASP2
SRM
EXOSC10
MTOR
ANGPTL7
UBIAD1
PTCHD2
FBXO2
FBXO44
FBXO6
MAD2L2

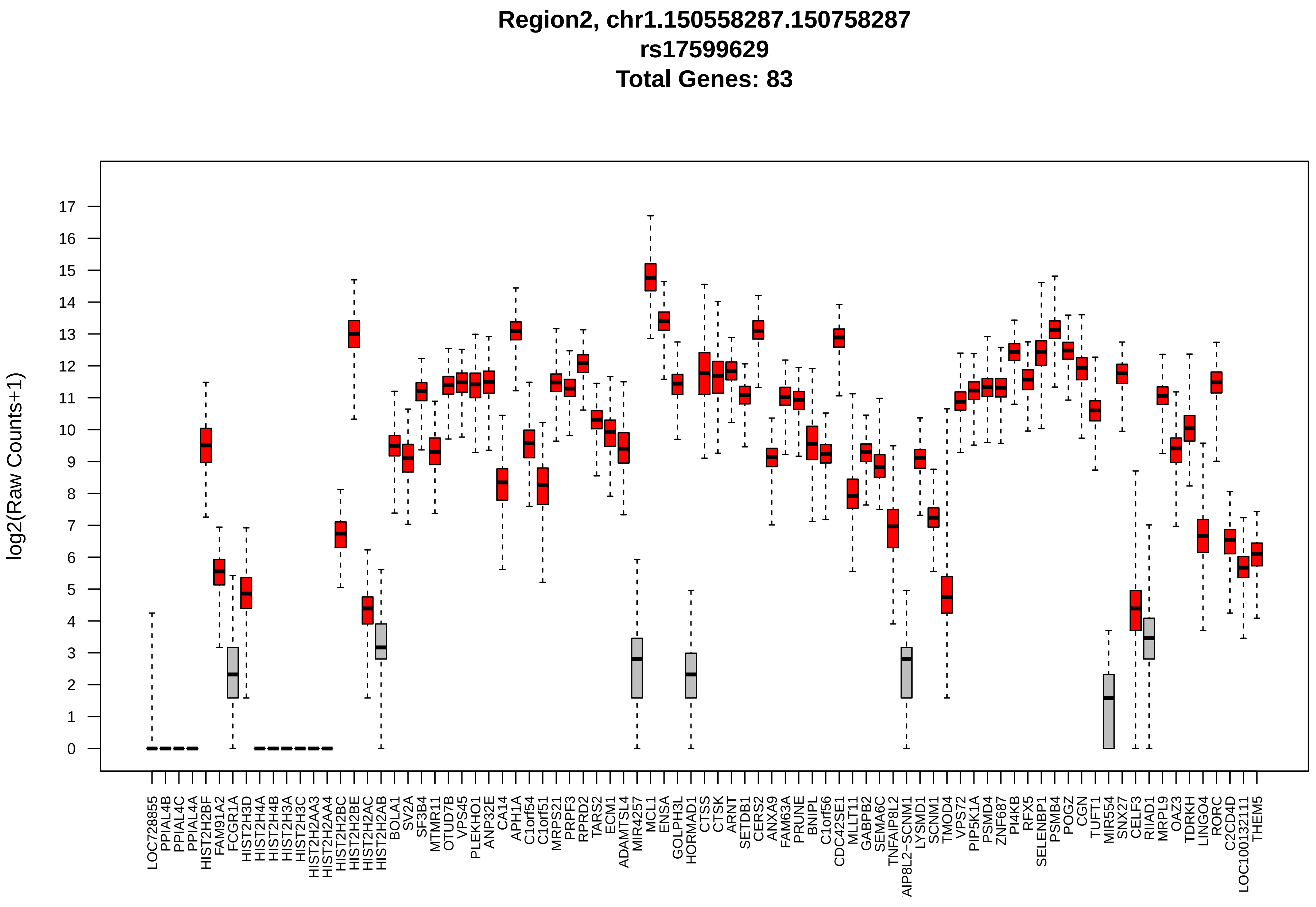
Region2, chr1.150558287.150758287
rs17599629
Total Genes: 83
log2(Raw Counts+1)
0
1
2
3
4
5
6
7
8
9
10
11
12
13
14
15
16
17
LOC728855
PPIAL4B
PPIAL4C
PPIAL4A
HIST2H2BF
FAM91A2
FCGR1A
HIST2H3D
HIST2H4A
HIST2H4B
HIST2H3A
HIST2H3C
HIST2H2AA3
HIST2H2AA4
HIST2H2BC
HIST2H2BE
HIST2H2AC
HIST2H2AB
BOLA1
SV2A
SF3B4
MTMR11
OTUD7B
VPS45
PLEKHO1
ANP32E
CA14
APH1A
C1orf54
C1orf51
MRPS21
PRPF3
RPRD2
TARS2
ECM1
ADAMTSL4
MIR4257
MCL1
ENSA
GOLPH3L
HORMAD1
CTSS
CTSK
ARNT
SETDB1
CERS2
ANXA9
FAM63A
PRUNE
BNIPL
C1orf56
CDC42SE1
MLLT11
GABPB2
SEMA6C
TNFAIP8L2
AIP8L2−SCNM1
LYSMD1
SCNM1
TMOD4
VPS72
PIP5K1A
PSMD4
ZNF687
PI4KB
RFX5
SELENBP1
PSMB4
POGZ
CGN
TUFT1
MIR554
SNX27
CELF3
RIIAD1
MRPL9
OAZ3
TDRKH
LINGO4
RORC
C2CD4D
LOC100132111
THEM5

Region3, chr1.154734183.154934183
rs1218582
Total Genes: 78

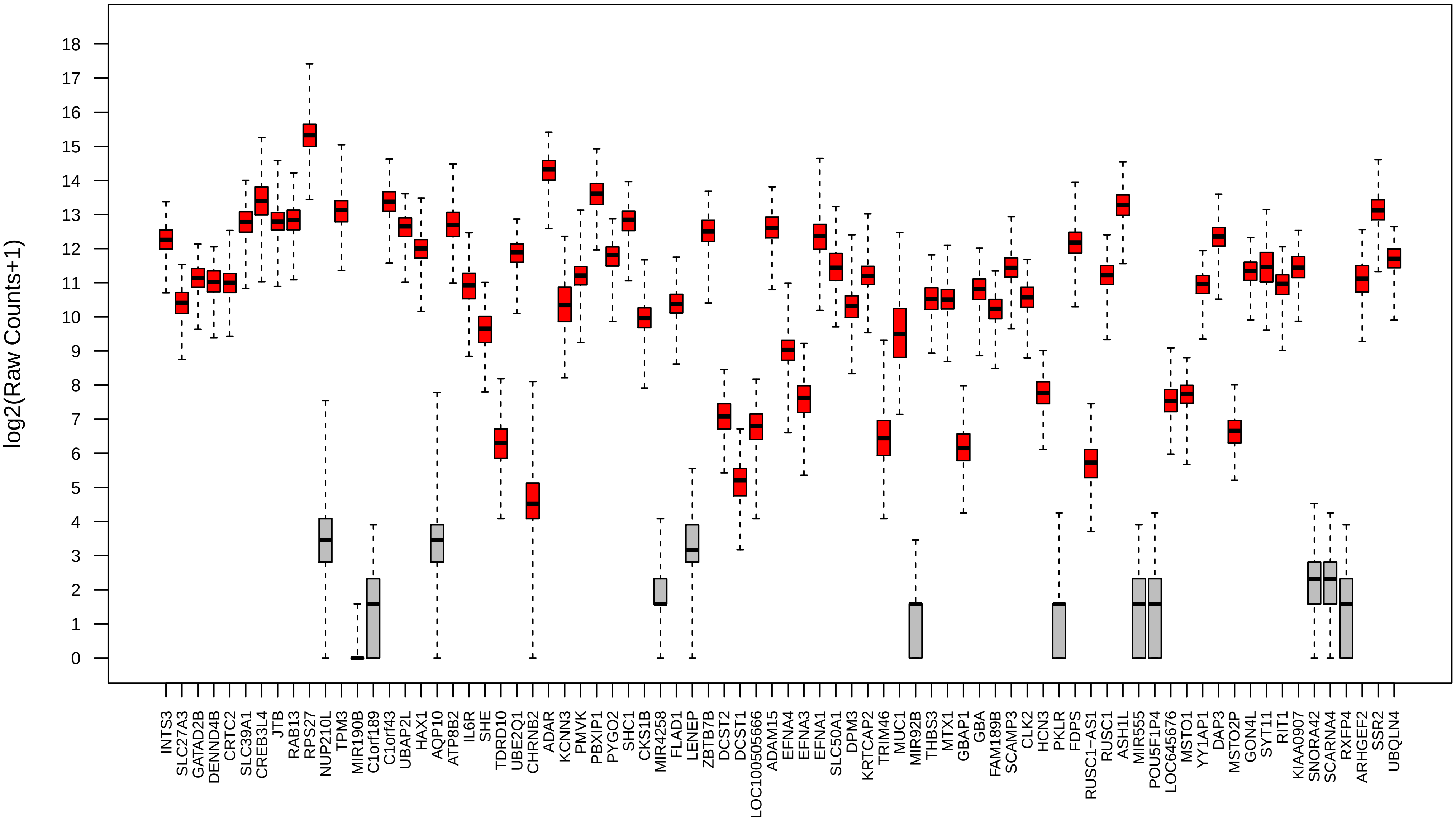

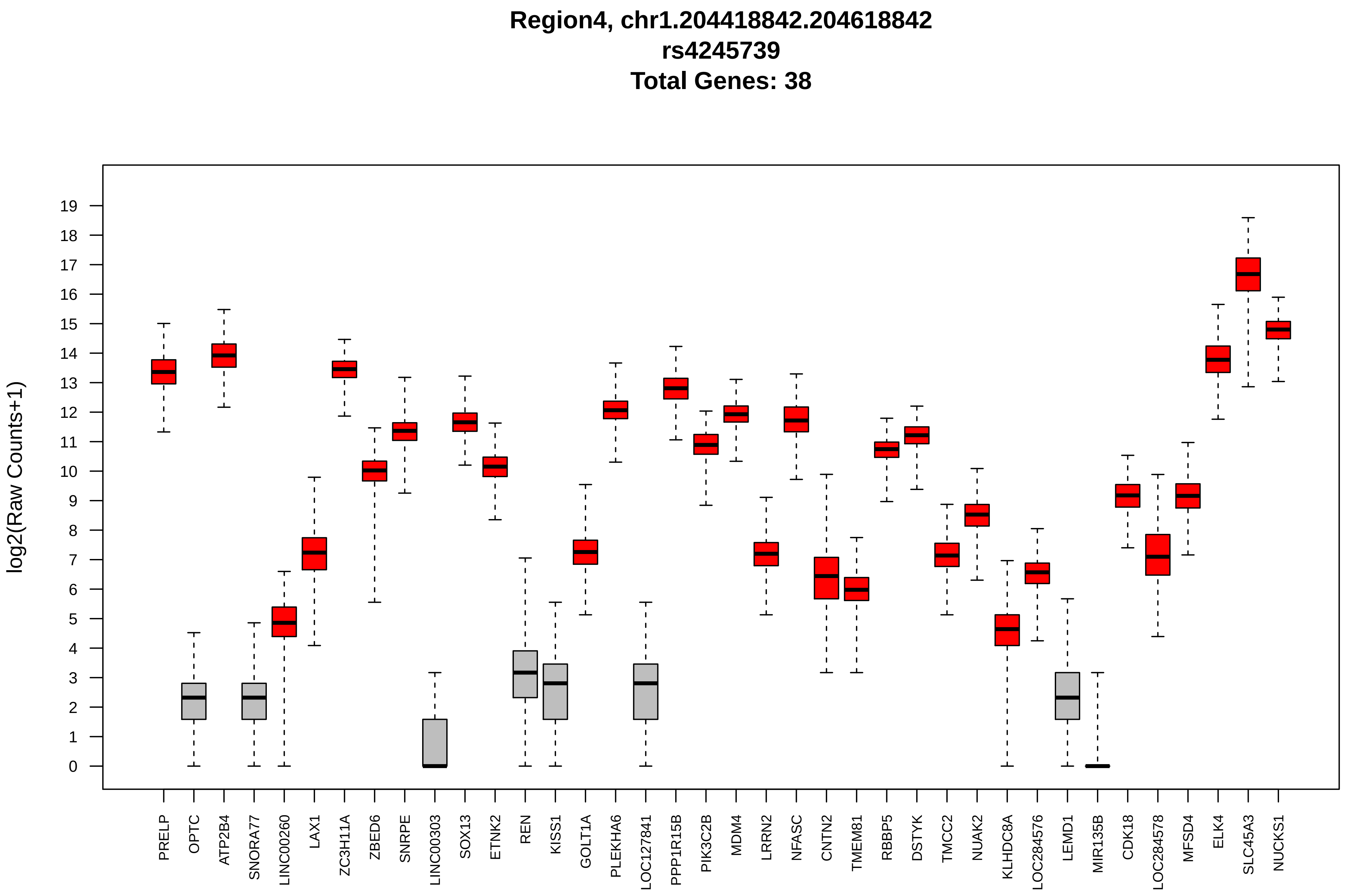

Region4, chr1.204418842.204618842
rs4245739
Total Genes: 38
log2(Raw Counts+1)
0
1
2
3
4
5
6
7
8
9
10
11
12
13
14
15
16
17
18
19
PRELP
OPTC
ATP2B4
SNORA77
LINC00260
LAX1
ZC3H11A
ZBED6
SNRPE
LINC00303
SOX13
ETNK2
REN
KISS1
GOLT1A
PLEKHA6
LOC127841
PPP1R15B
PIK3C2B
MDM4
LRRN2
NFASC
CNTN2
TMEM81
RBBP5
DSTYK
TMCC2
NUAK2
KLHDC8A
LOC284576
LEMD1
MIR135B
CDK18
LOC284578
MFSD4
ELK4
SLC45A3
NUCKS1

Region5, chr1.205657824.205857824
rs1775148
Total Genes: 32

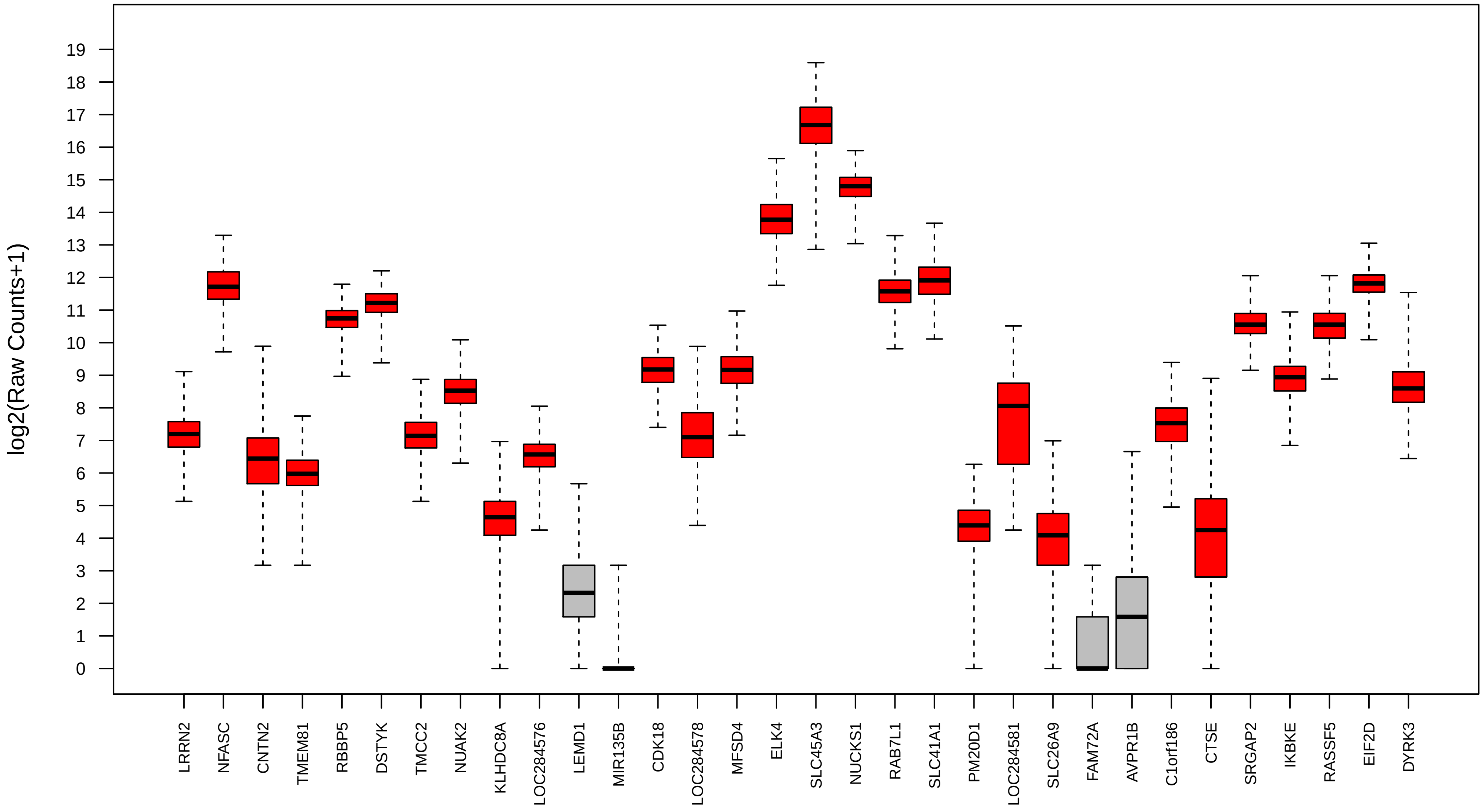

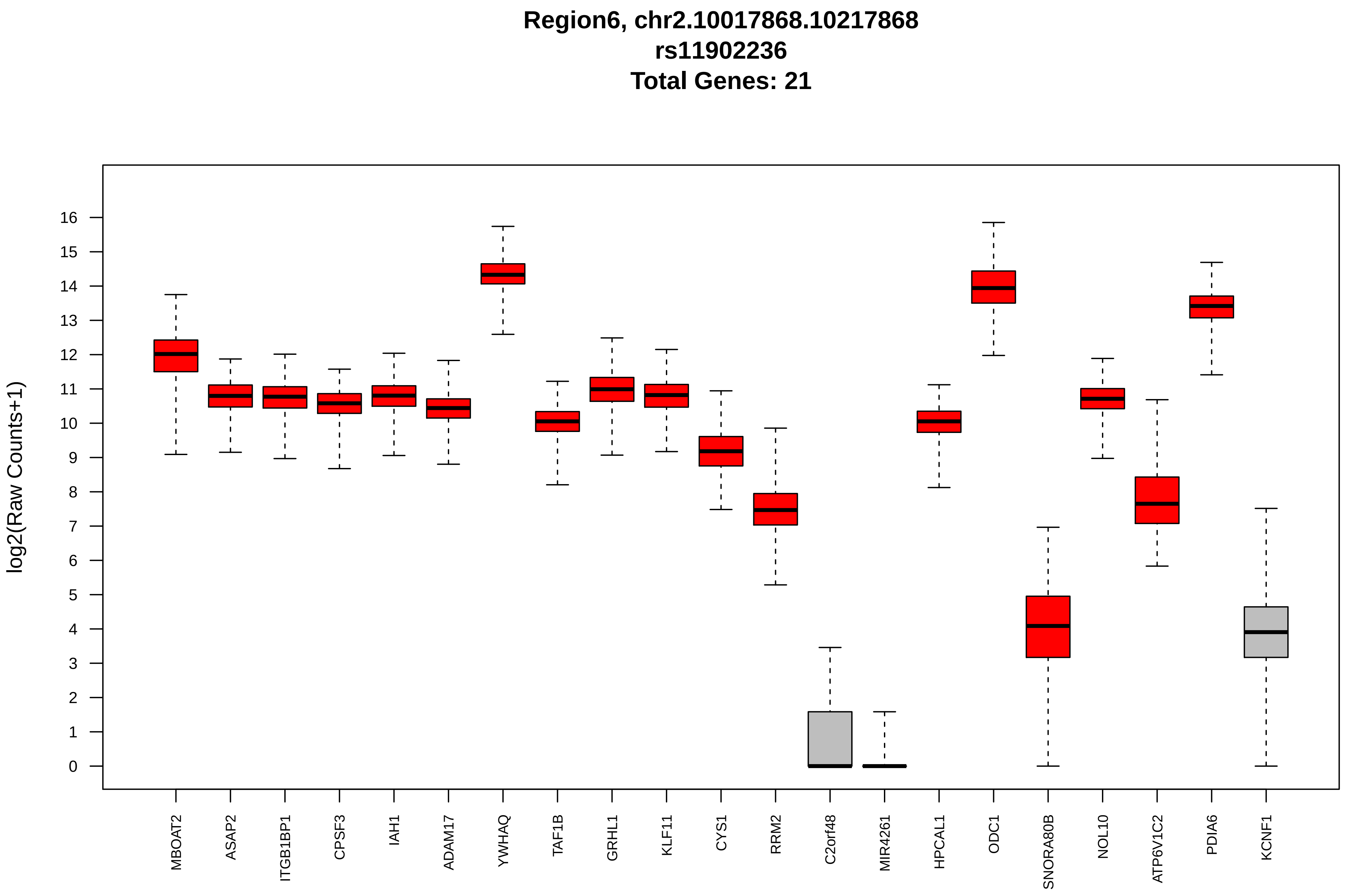

Region6, chr2.10017868.10217868
rs11902236
Total Genes: 21
log2(Raw Counts+1)
0
1
2
3
4
5
6
7
8
9
10
11
12
13
14
15
16
MBOAT2
ASAP2
ITGB1BP1
CPSF3
IAH1
ADAM17
YWHAQ
TAF1B
GRHL1
KLF11
CYS1
RRM2
C2orf48
MIR4261
HPCAL1
ODC1
SNORA80B
NOL10
ATP6V1C2
PDIA6
KCNF1

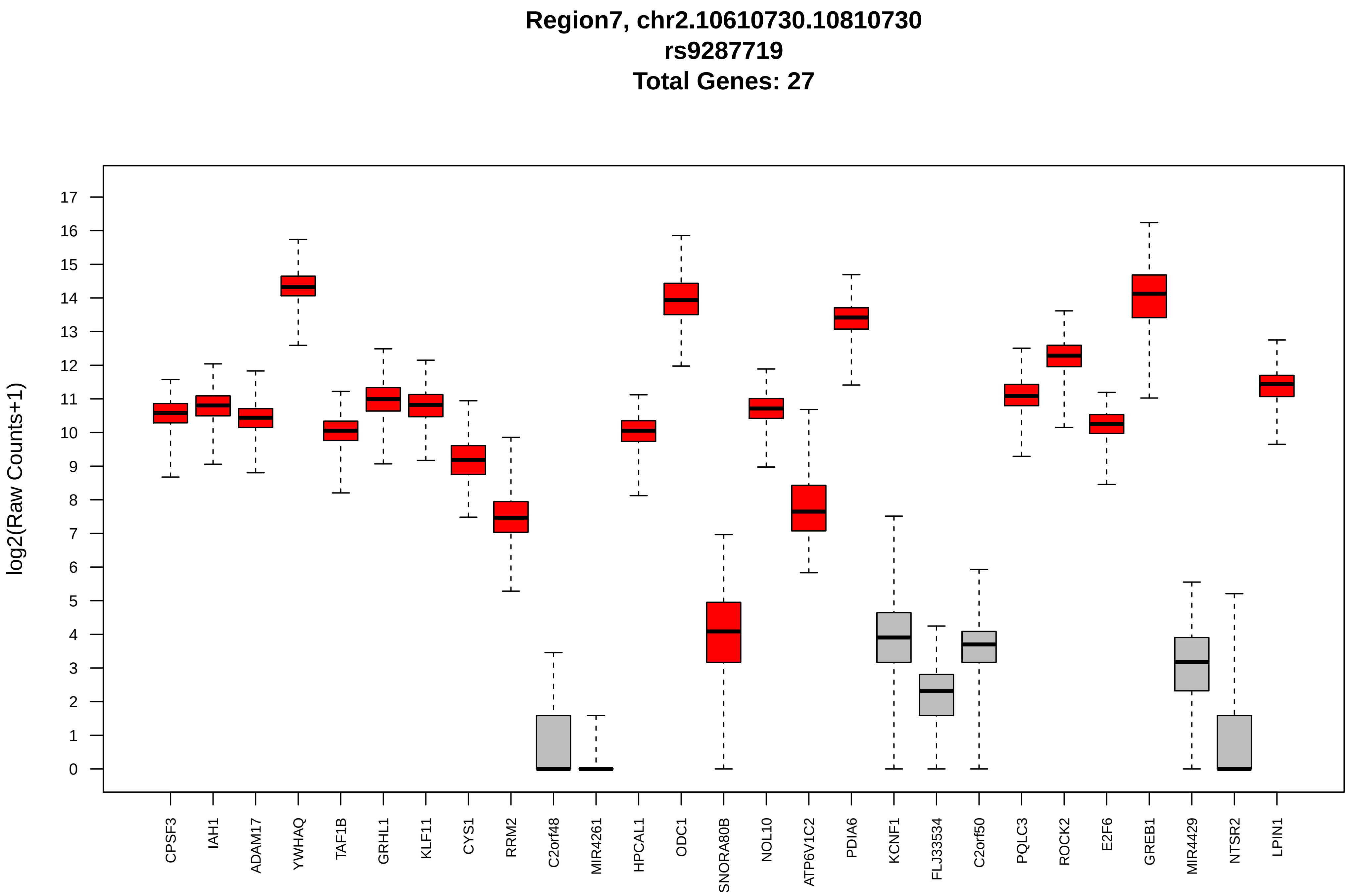

Region7, chr2.10610730.10810730
rs9287719
Total Genes: 27
log2(Raw Counts+1)
0
1
2
3
4
5
6
7
8
9
10
11
12
13
14
15
16
17
CPSF3
IAH1
ADAM17
YWHAQ
TAF1B
GRHL1
KLF11
CYS1
RRM2
C2orf48
MIR4261
HPCAL1
ODC1
SNORA80B
NOL10
ATP6V1C2
PDIA6
KCNF1
FLJ33534
C2orf50
PQLC3
ROCK2
E2F6
GREB1
MIR4429
NTSR2
LPIN1

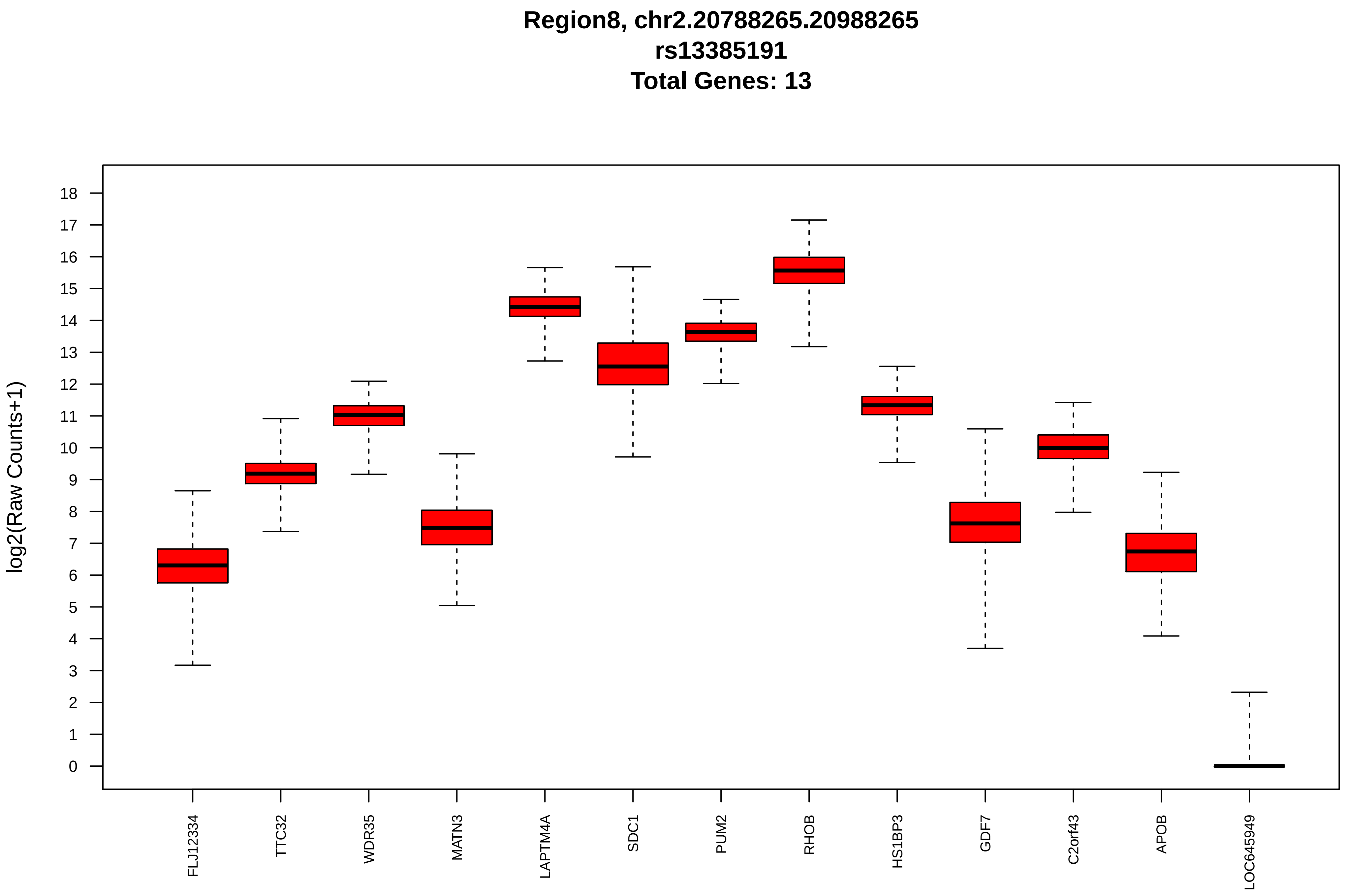
Region8, chr2.20788265.20988265
rs13385191
Total Genes: 13
log2(Raw Counts+1)
0
1
2
3
4
5
6
7
8
9
10
11
12
13
14
15
16
17
18
FLJ12334
TTC32
WDR35
MATN3
LAPTM4A
SDC1
PUM2
RHOB
HS1BP3
GDF7
C2orf43
APOB
LOC645949

Region9, chr2.43453949.43653949
rs1465618
Total Genes: 19

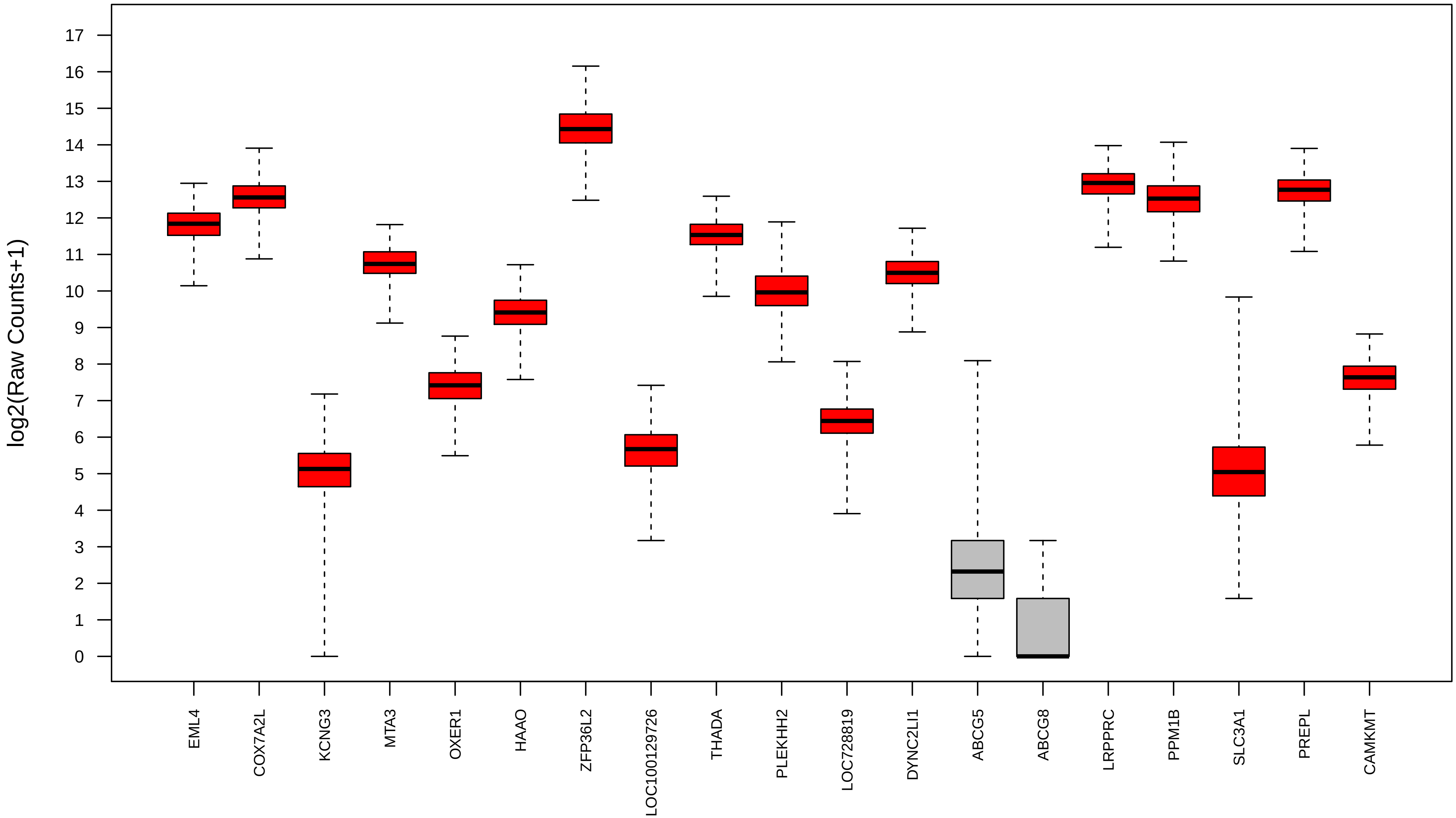

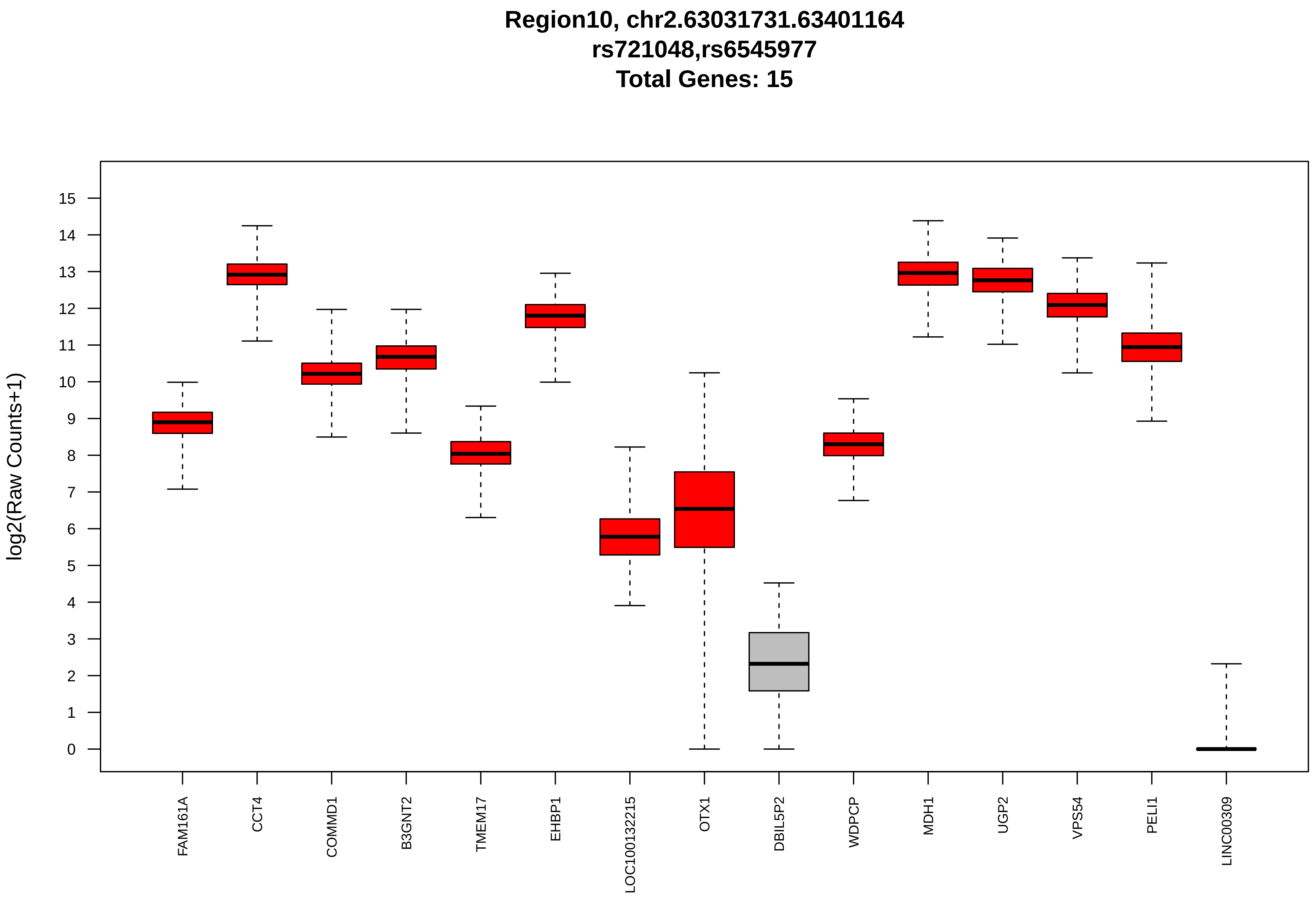

Region10, chr2.63031731.63401164
rs721048,rs6545977
Total Genes: 15
log2(Raw Counts+1)
0
1
2
3
4
5
6
7
8
9
10
11
12
13
14
15
FAM161A
CCT4
COMMD1
B3GNT2
TMEM17
EHBP1
LOC100132215
OTX1
DBIL5P2
WDPCP
MDH1
UGP2
VPS54
PELI1
LINC00309

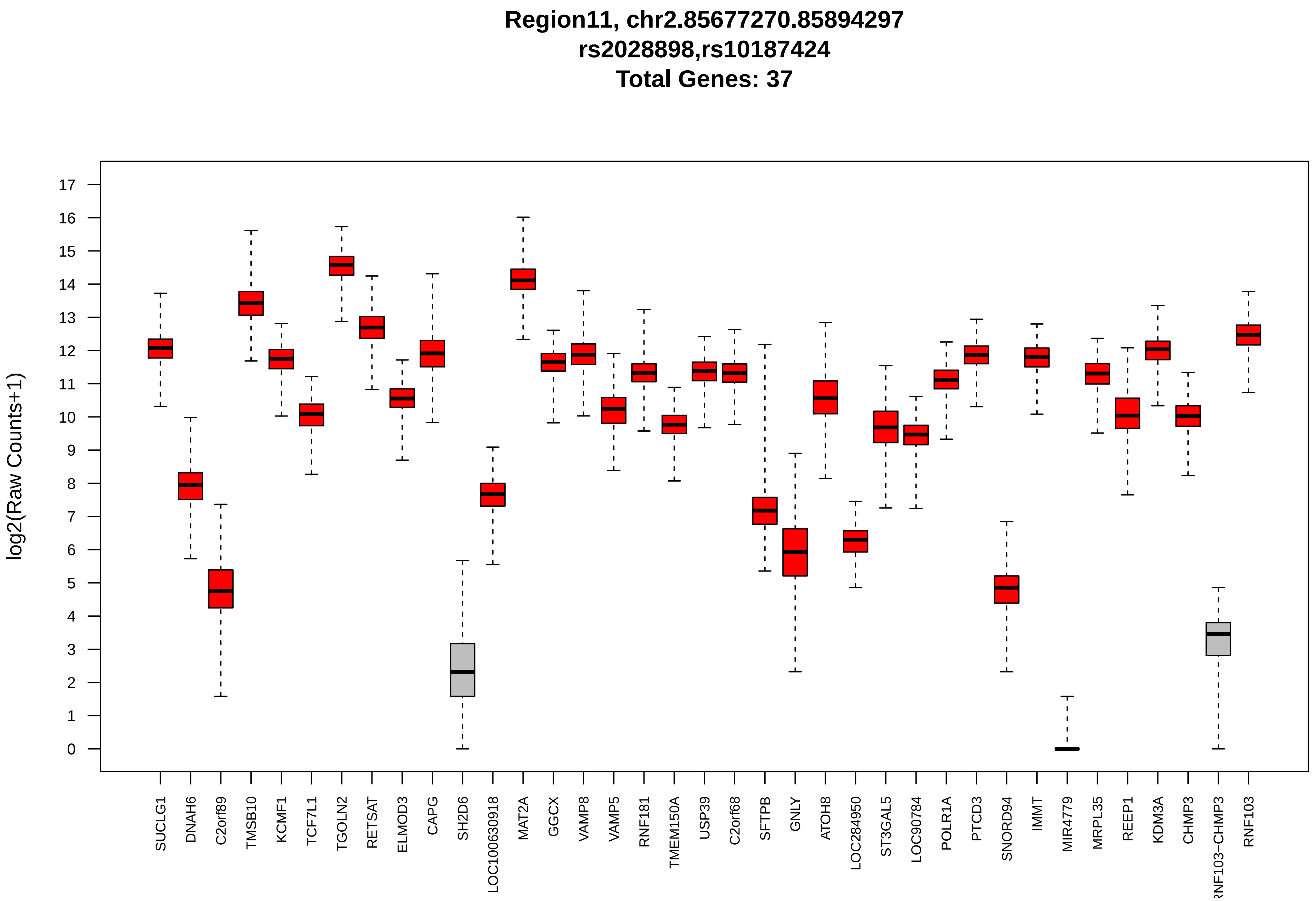

Region11, chr2.85677270.85894297
rs2028898,rs10187424
Total Genes: 37
log2(Raw Counts+1)
0
1
2
3
4
5
6
7
8
9
10
11
12
13
14
15
16
17
SUCLG1
DNAH6
C2orf89
TMSB10
KCMF1
TCF7L1
TGOLN2
RETSAT
ELMOD3
CAPG
SH2D6
LOC100630918
MAT2A
GGCX
VAMP8
VAMP5
RNF181
TMEM150A
USP39
C2orf68
SFTPB
GNLY
ATOH8
LOC284950
ST3GAL5
LOC90784
POLR1A
PTCD3
SNORD94
IMMT
MIR4779
MRPL35
REEP1
KDM3A
CHMP3
RNF103−CHMP3
RNF103

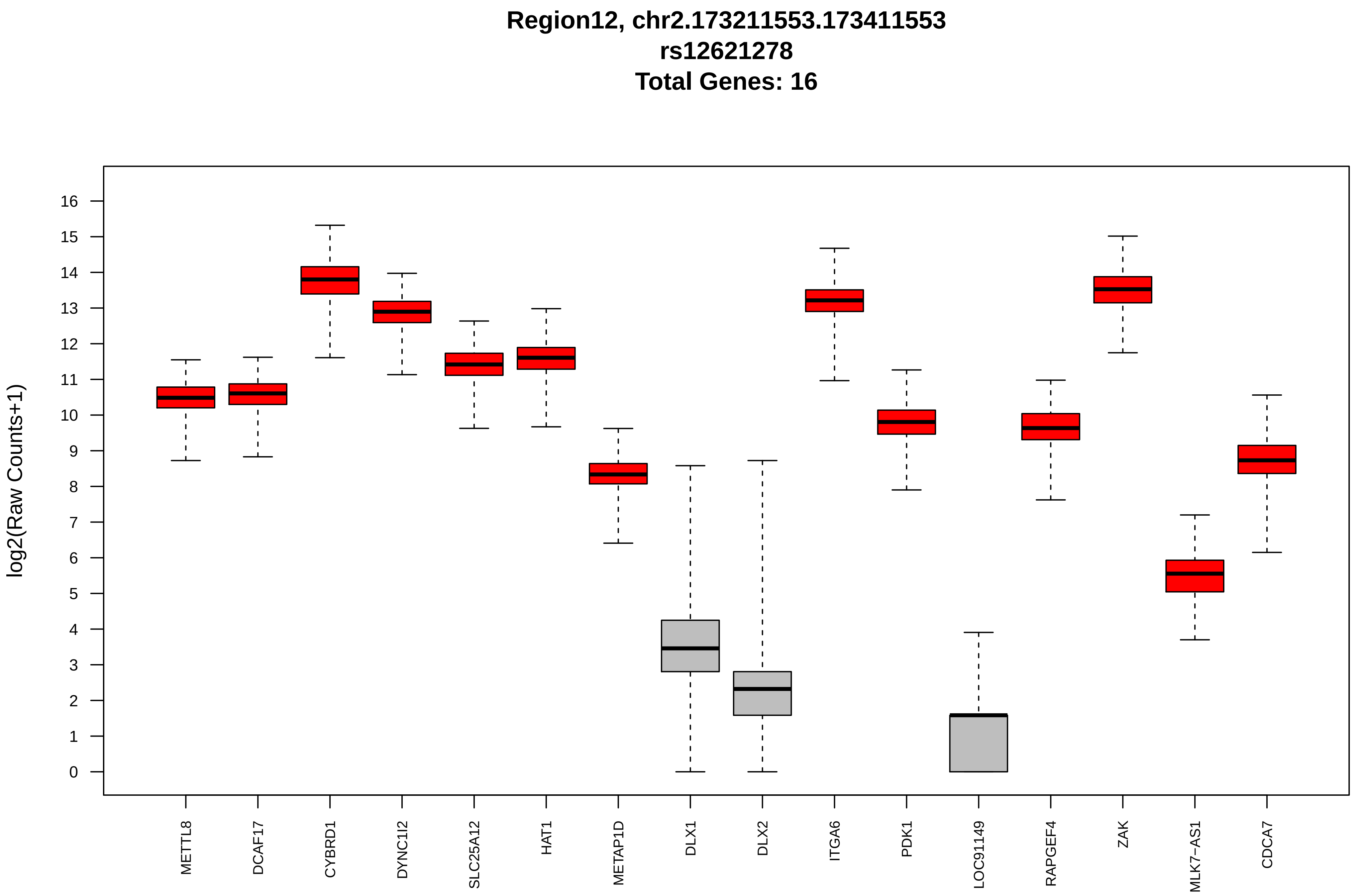
Region12, chr2.173211553.173411553
rs12621278
Total Genes: 16
log2(Raw Counts+1)
0
1
2
3
4
5
6
7
8
9
10
11
12
13
14
15
16
METTL8
DCAF17
CYBRD1
DYNC1I2
SLC25A12
HAT1
METAP1D
DLX1
DLX2
ITGA6
PDK1
LOC91149
RAPGEF4
ZAK
MLK7−AS1
CDCA7

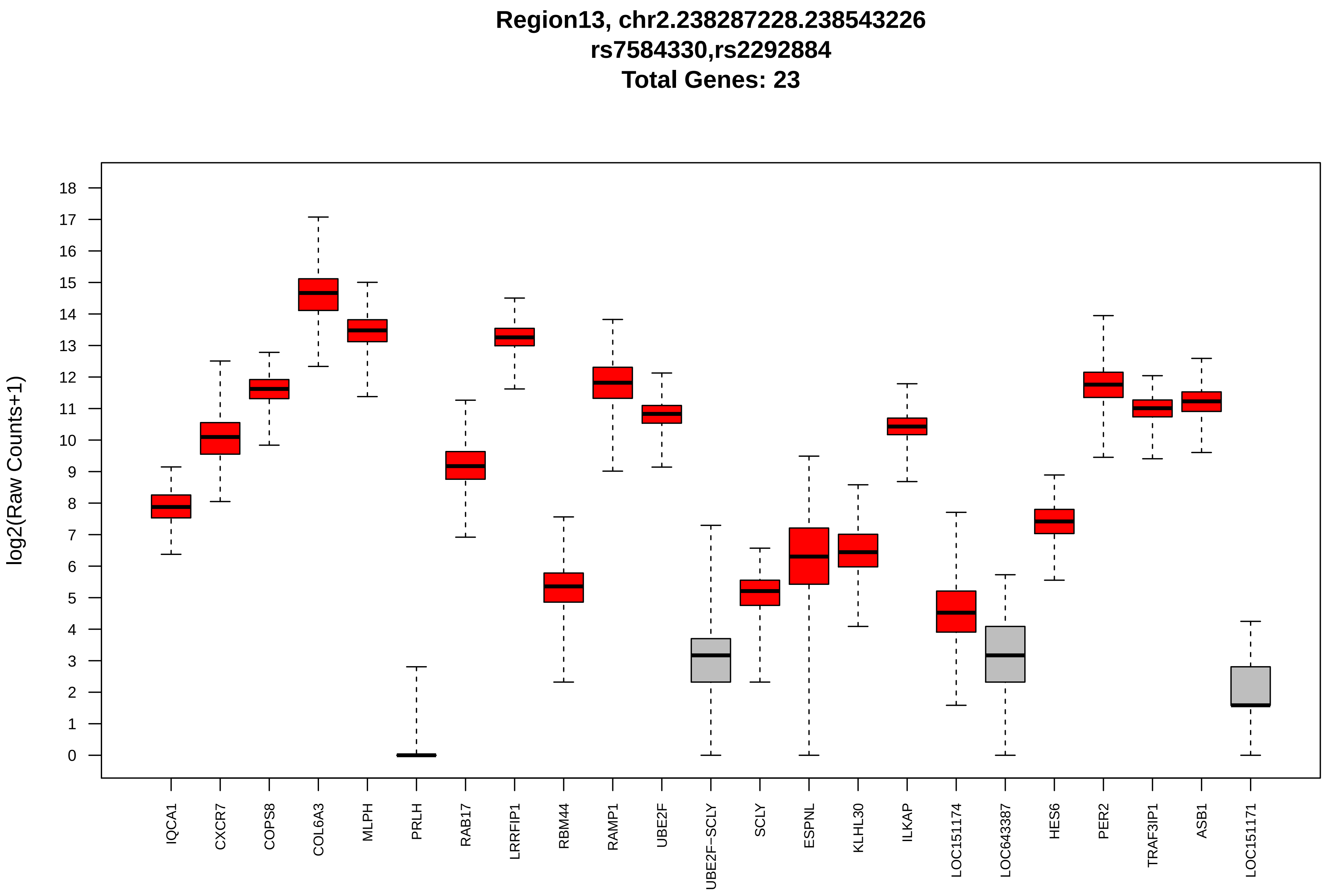

Region13, chr2.238287228.238543226
rs7584330,rs2292884
Total Genes: 23
log2(Raw Counts+1)
0
1
2
3
4
5
6
7
8
9
10
11
12
13
14
15
16
17
18
IQCA1
CXCR7
COPS8
COL6A3
MLPH
PRLH
RAB17
LRRFIP1
RBM44
RAMP1
UBE2F
UBE2F−SCLY
SCLY
ESPNL
KLHL30
ILKAP
LOC151174
LOC643387
HES6
PER2
TRAF3IP1
ASB1
LOC151171

Region14, chr2.242282864.242482864
rs3771570
Total Genes: 35

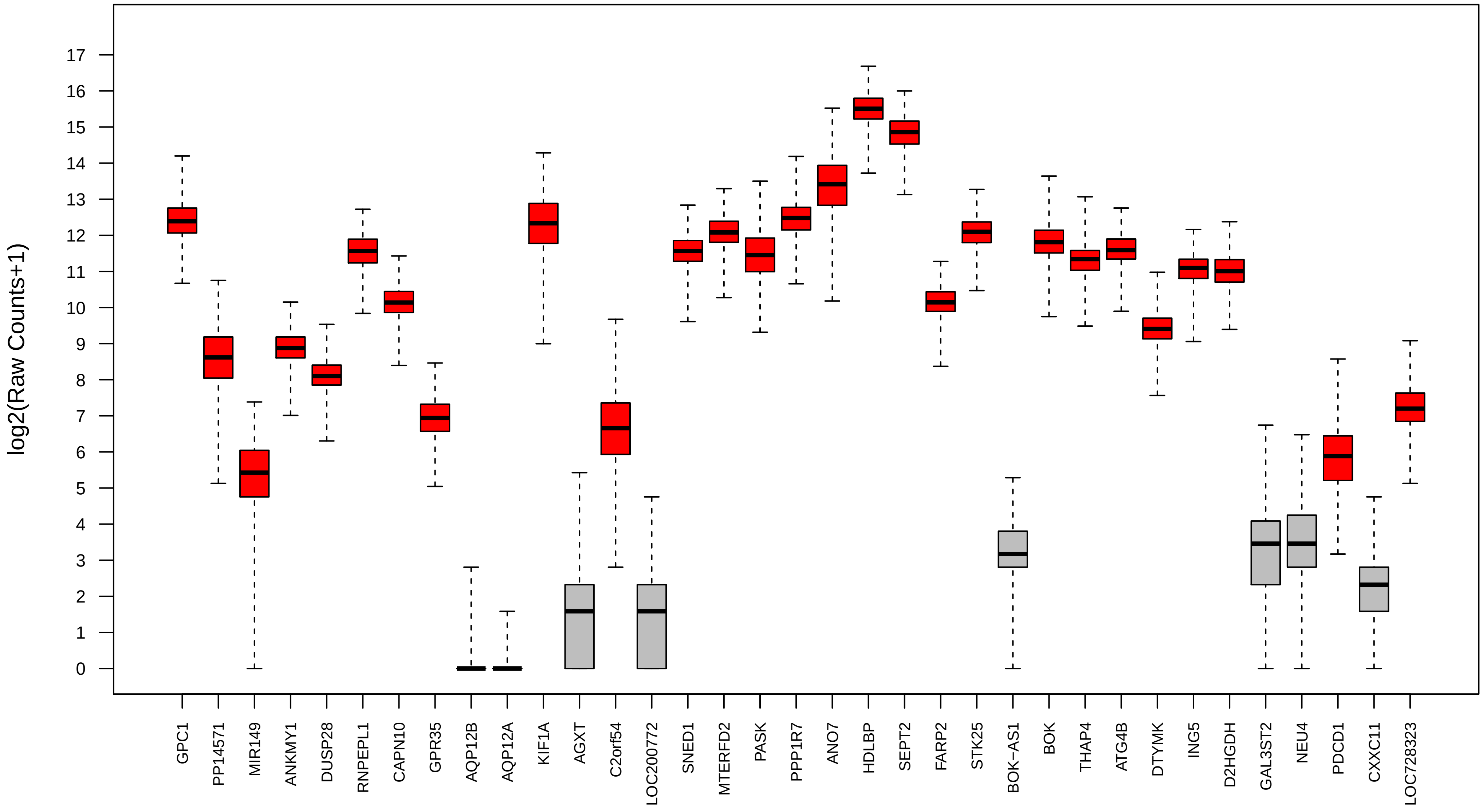

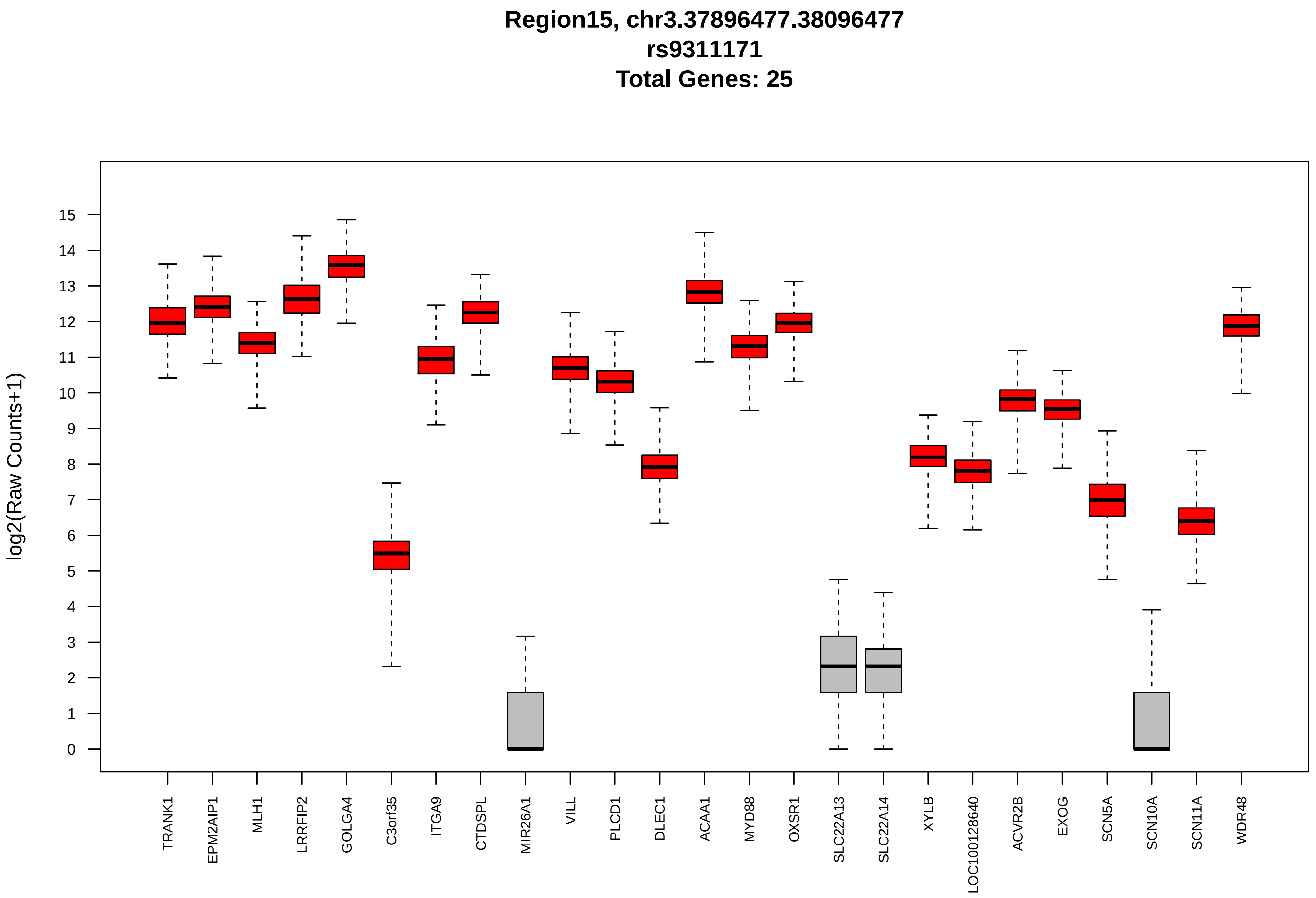

Region15, chr3.37896477.38096477
rs9311171
Total Genes: 25
log2(Raw Counts+1)
0
1
2
3
4
5
6
7
8
9
10
11
12
13
14
15
TRANK1
EPM2AIP1
MLH1
LRRFIP2
GOLGA4
C3orf35
ITGA9
CTDSPL
MIR26A1
VILL
PLCD1
DLEC1
ACAA1
MYD88
OXSR1
SLC22A13
SLC22A14
XYLB
LOC100128640
ACVR2B
EXOG
SCN5A
SCN10A
SCN11A
WDR48

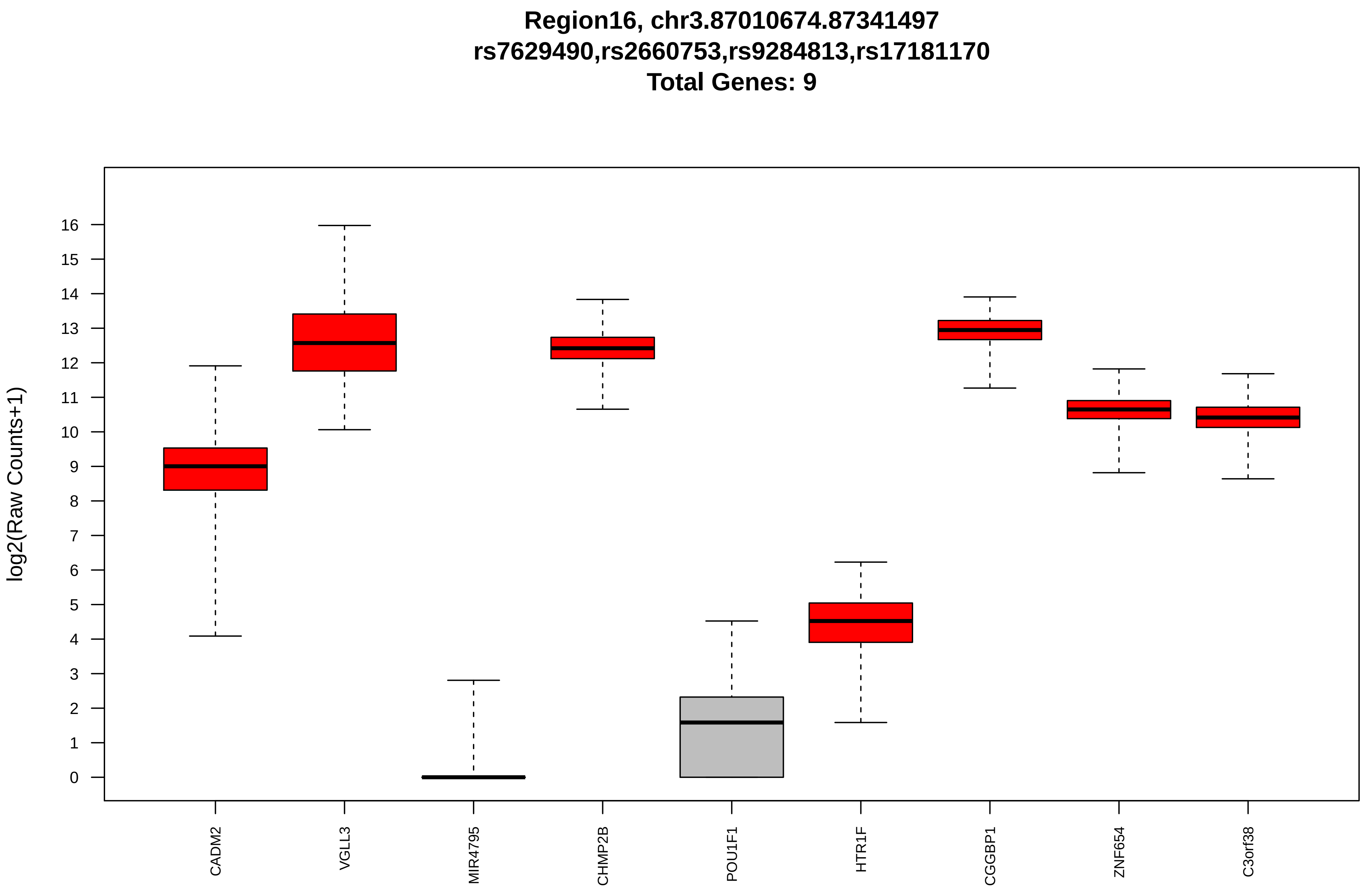
Region16, chr3.87010674.87341497
rs7629490,rs2660753,rs9284813,rs17181170
Total Genes: 9
log2(Raw Counts+1)
0
1
2
3
4
5
6
7
8
9
10
11
12
13
14
15
16
CADM2
VGLL3
MIR4795
CHMP2B
POU1F1
HTR1F
CGGBP1
ZNF654
C3orf38

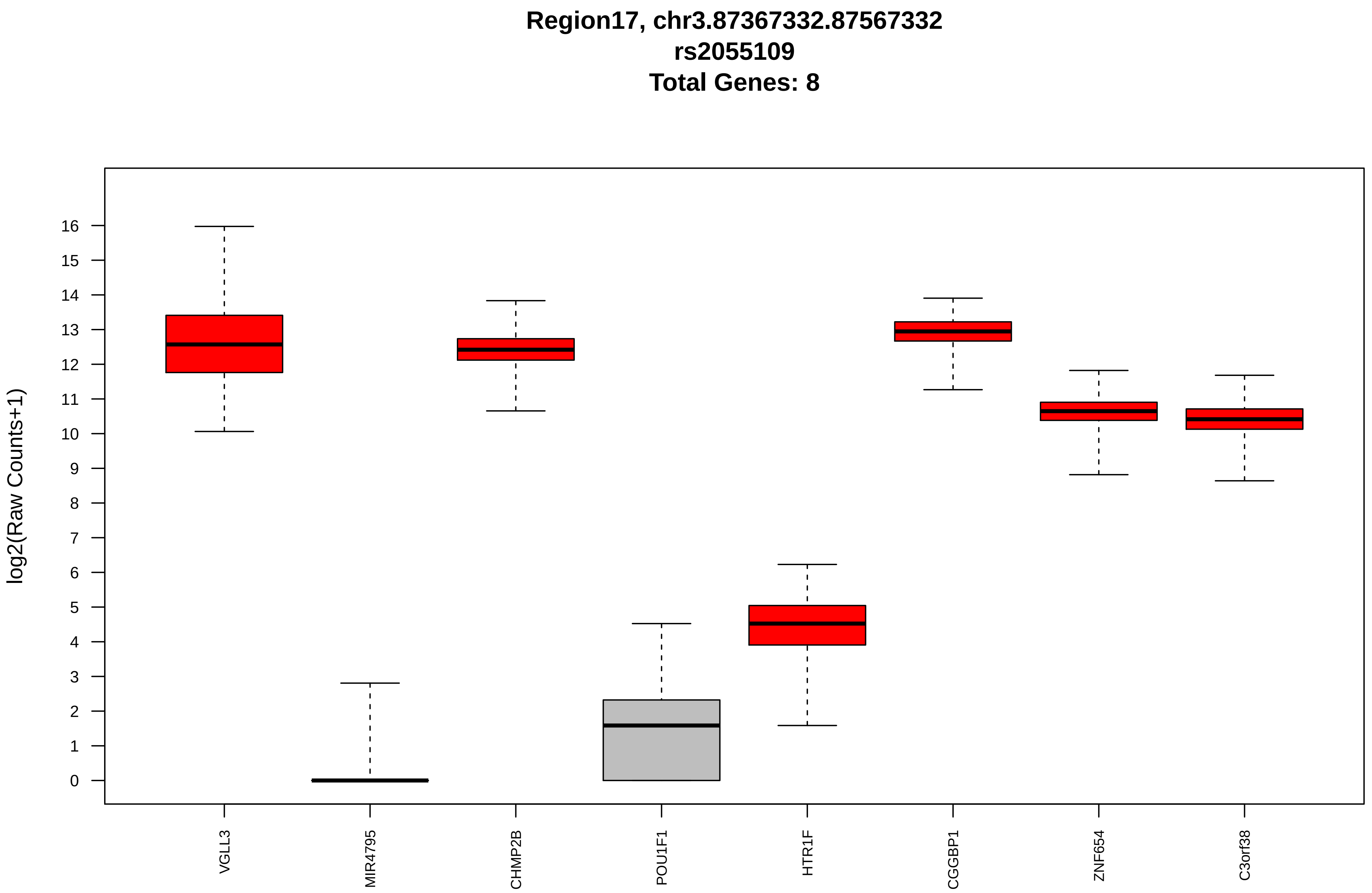

Region17, chr3.87367332.87567332
rs2055109
Total Genes: 8
log2(Raw Counts+1)
0
1
2
3
4
5
6
7
8
9
10
11
12
13
14
15
16
VGLL3
MIR4795
CHMP2B
POU1F1
HTR1F
CGGBP1
ZNF654
C3orf38

Region18, chr3.113175624.113375624
rs7611694
Total Genes: 26

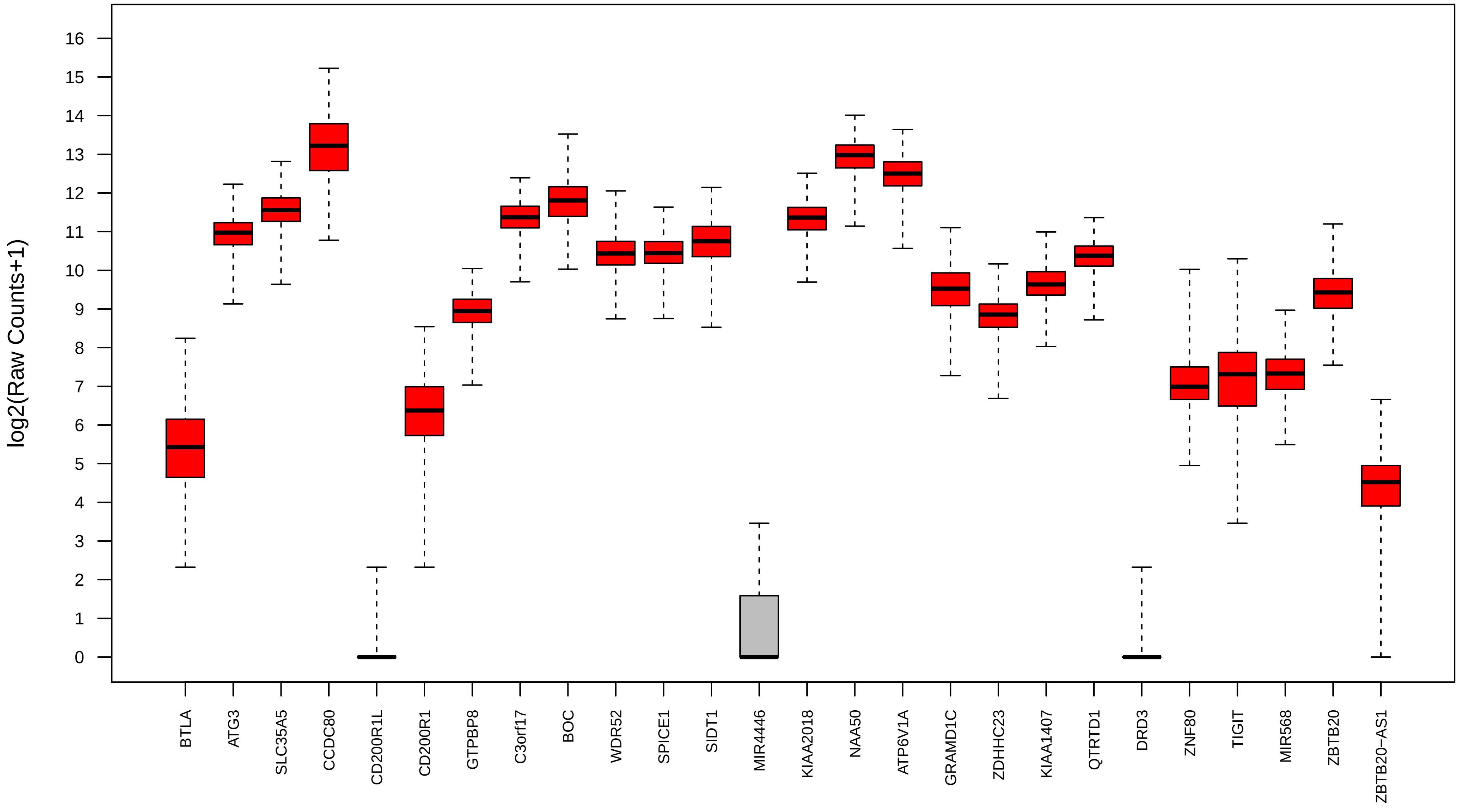

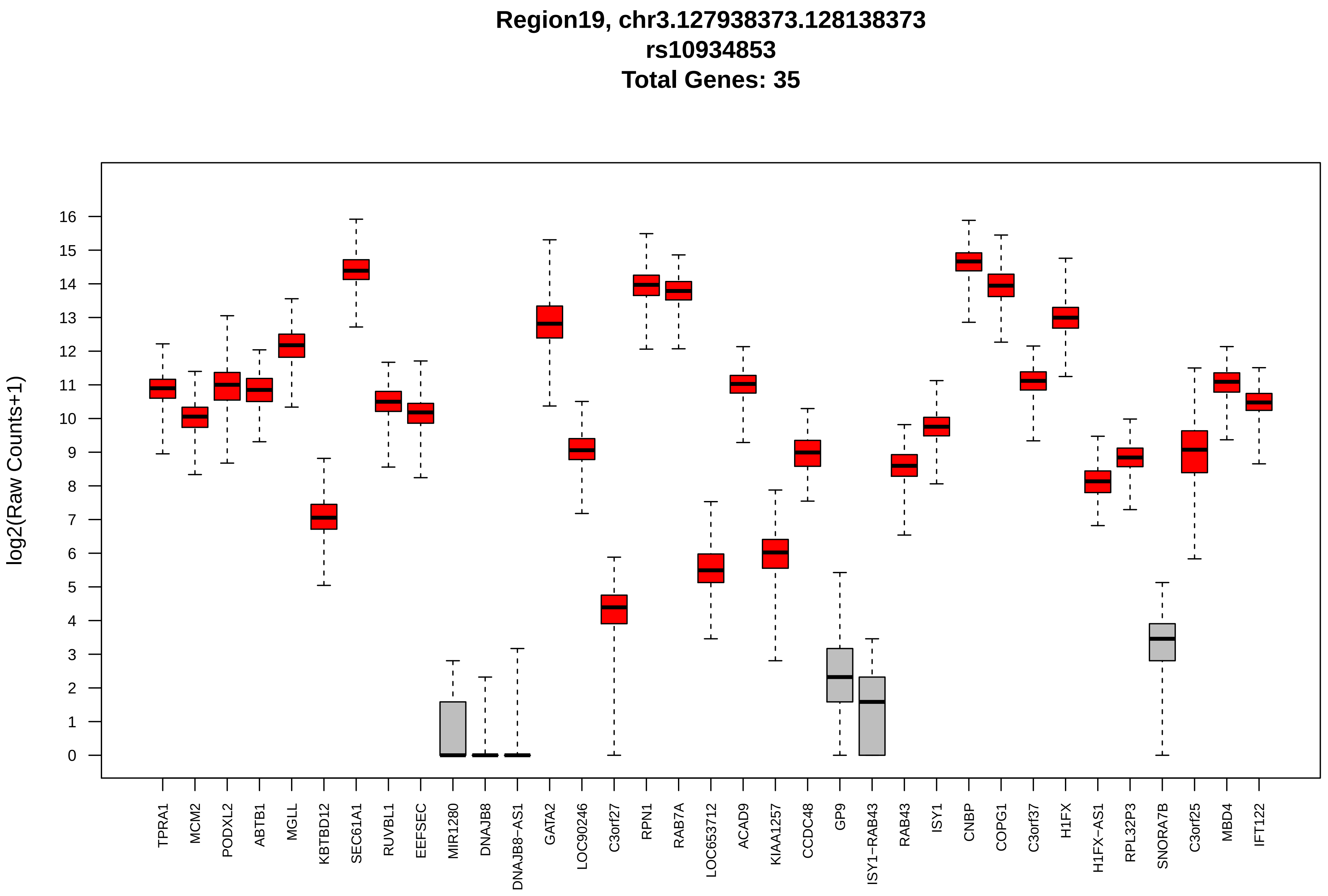
Region19, chr3.127938373.128138373
rs10934853
Total Genes: 35
log2(Raw Counts+1)
0
1
2
3
4
5
6
7
8
9
10
11
12
13
14
15
16
TPRA1
MCM2
PODXL2
ABTB1
MGLL
KBTBD12
SEC61A1
RUVBL1
EEFSEC
MIR1280
DNAJB8
DNAJB8−AS1
GATA2
LOC90246
C3orf27
RPN1
RAB7A
LOC653712
ACAD9
KIAA1257
CCDC48
GP9
ISY1−RAB43
RAB43
ISY1
CNBP
COPG1
C3orf37
H1FX
H1FX−AS1
RPL32P3
SNORA7B
C3orf25
MBD4
IFT122

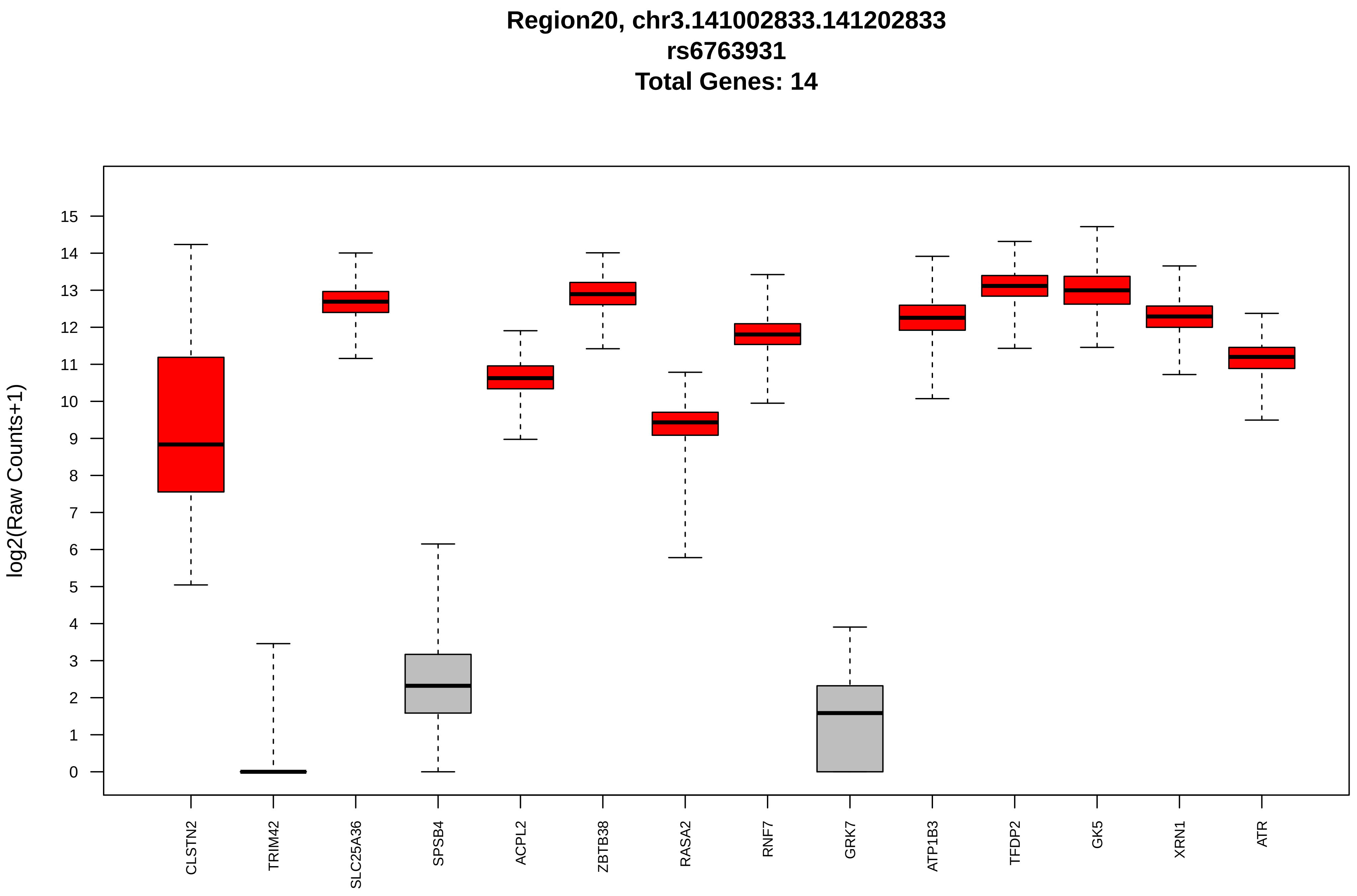
Region20, chr3.141002833.141202833
rs6763931
Total Genes: 14
log2(Raw Counts+1)
0
1
2
3
4
5
6
7
8
9
10
11
12
13
14
15
CLSTN2
TRIM42
SLC25A36
SPSB4
ACPL2
ZBTB38
RASA2
RNF7
GRK7
ATP1B3
TFDP2
GK5
XRN1
ATR

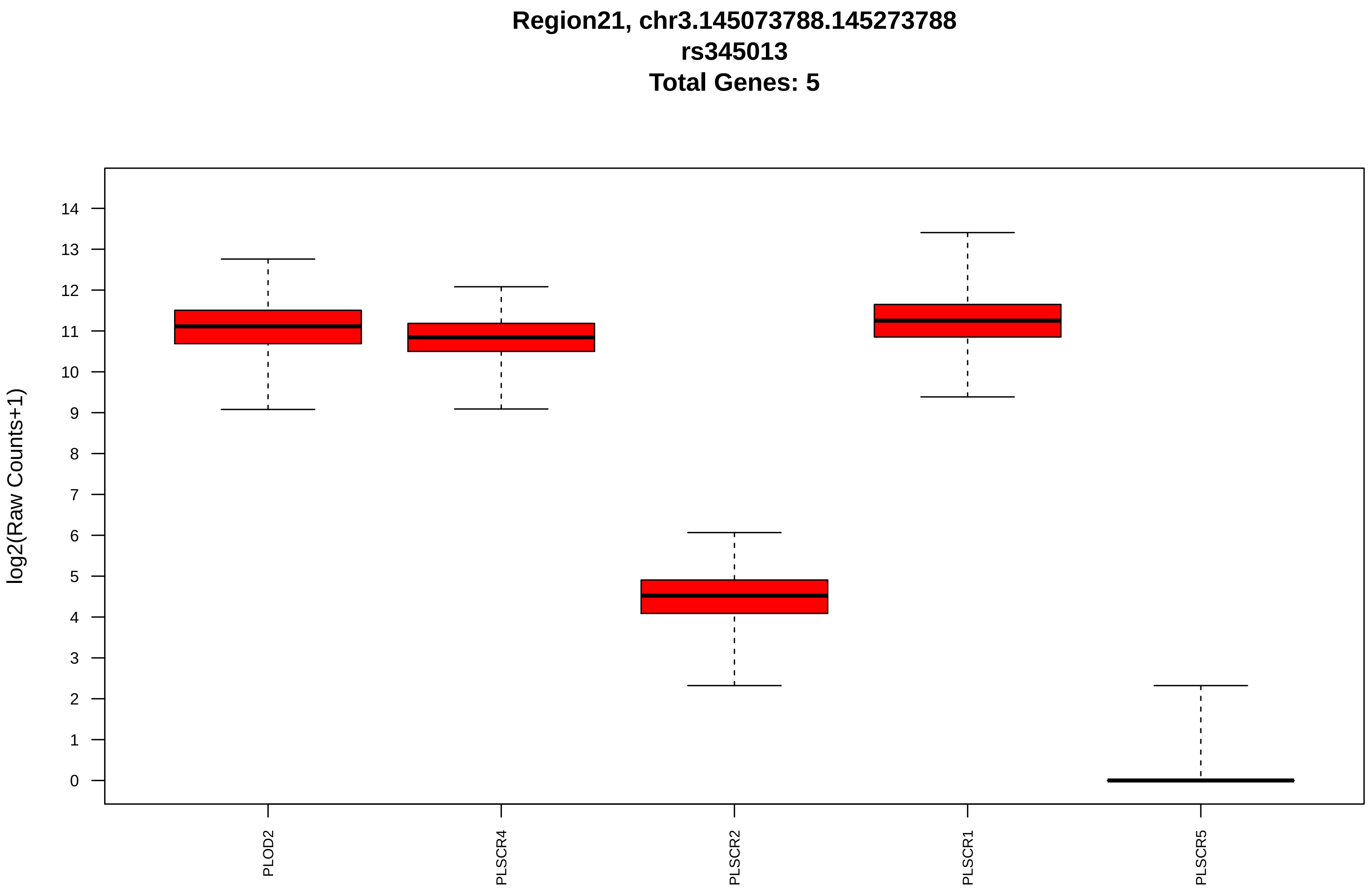
Region21, chr3.145073788.145273788
rs345013
Total Genes: 5
log2(Raw Counts+1)
0
1
2
3
4
5
6
7
8
9
10
11
12
13
14
PLOD2
PLSCR4
PLSCR2
PLSCR1
PLSCR5

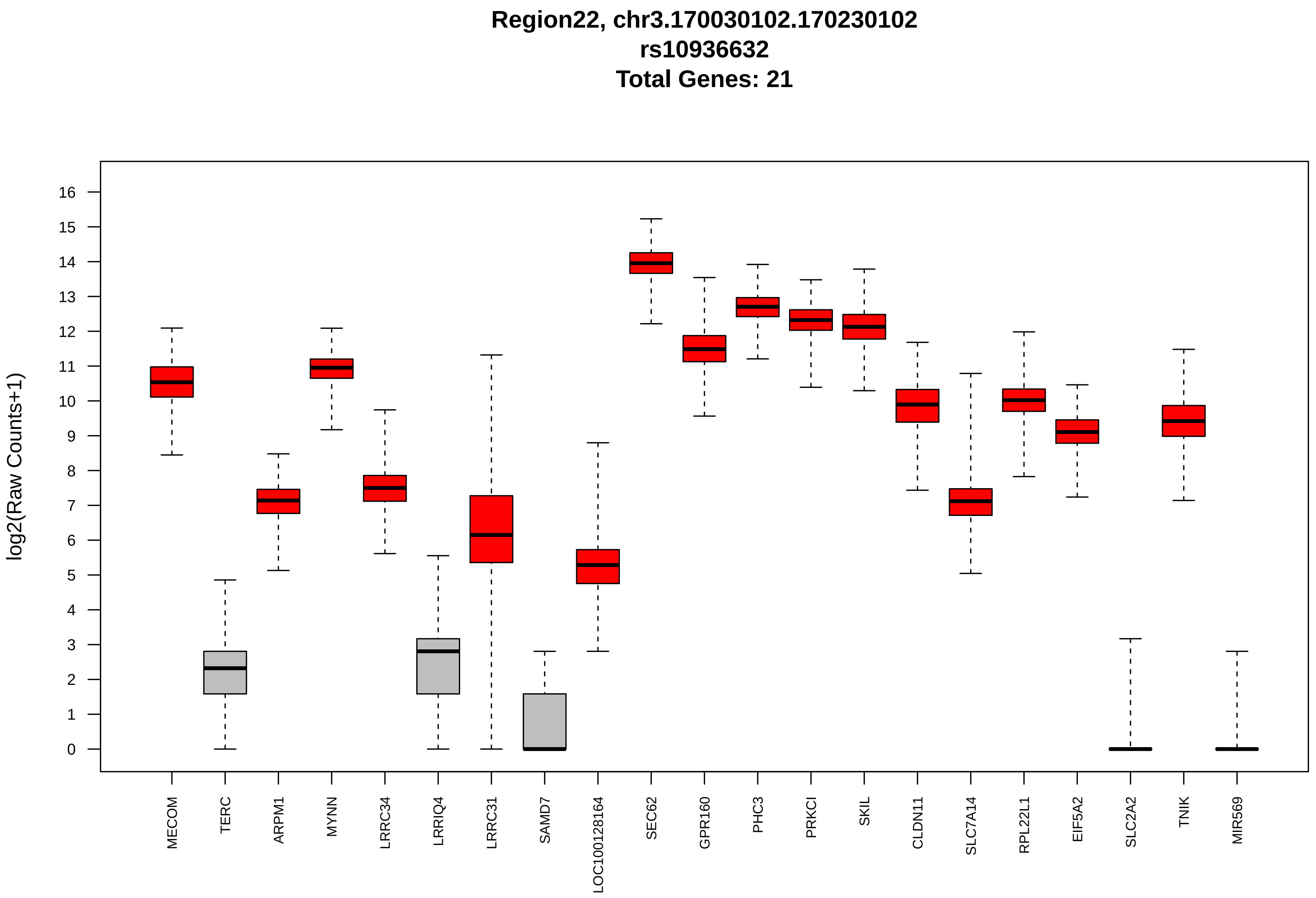
Region22, chr3.170030102.170230102
rs10936632
Total Genes: 21
log2(Raw Counts+1)
0
1
2
3
4
5
6
7
8
9
10
11
12
13
14
15
16
MECOM
TERC
ARPM1
MYNN
LRRC34
LRRIQ4
LRRC31
SAMD7
LOC100128164
SEC62
GPR160
PHC3
PRKCI
SKIL
CLDN11
SLC7A14
RPL22L1
EIF5A2
SLC2A2
TNIK
MIR569

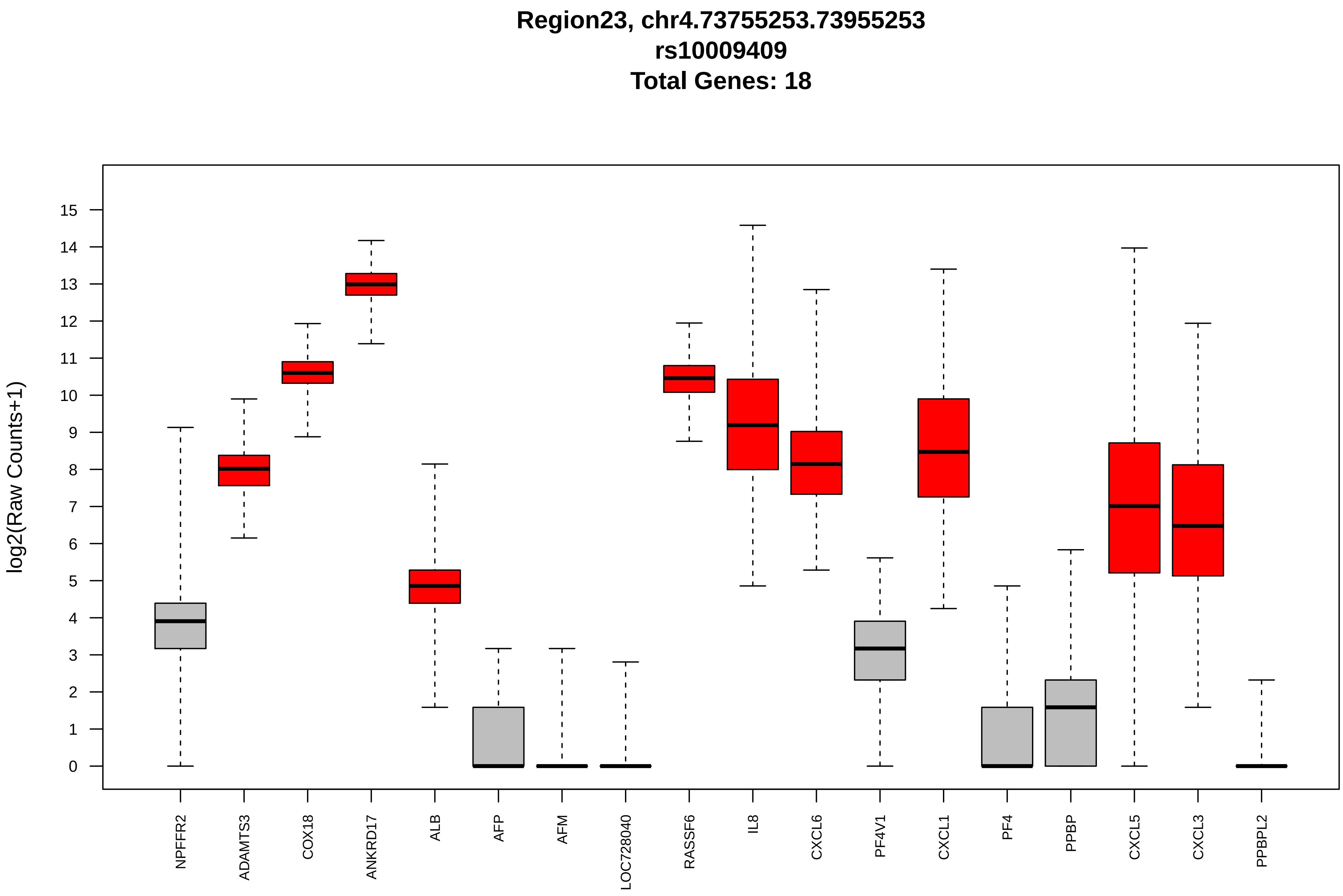
Region23, chr4.73755253.73955253
rs10009409
Total Genes: 18
log2(Raw Counts+1)
0
1
2
3
4
5
6
7
8
9
10
11
12
13
14
15
NPFFR2
ADAMTS3
COX18
ANKRD17
ALB
AFP
AFM
LOC728040
RASSF6
IL8
CXCL6
PF4V1
CXCL1
PF4
PPBP
CXCL5
CXCL3
PPBPL2

Region24, chr4.74249158.74449158
rs1894292
Total Genes: 22

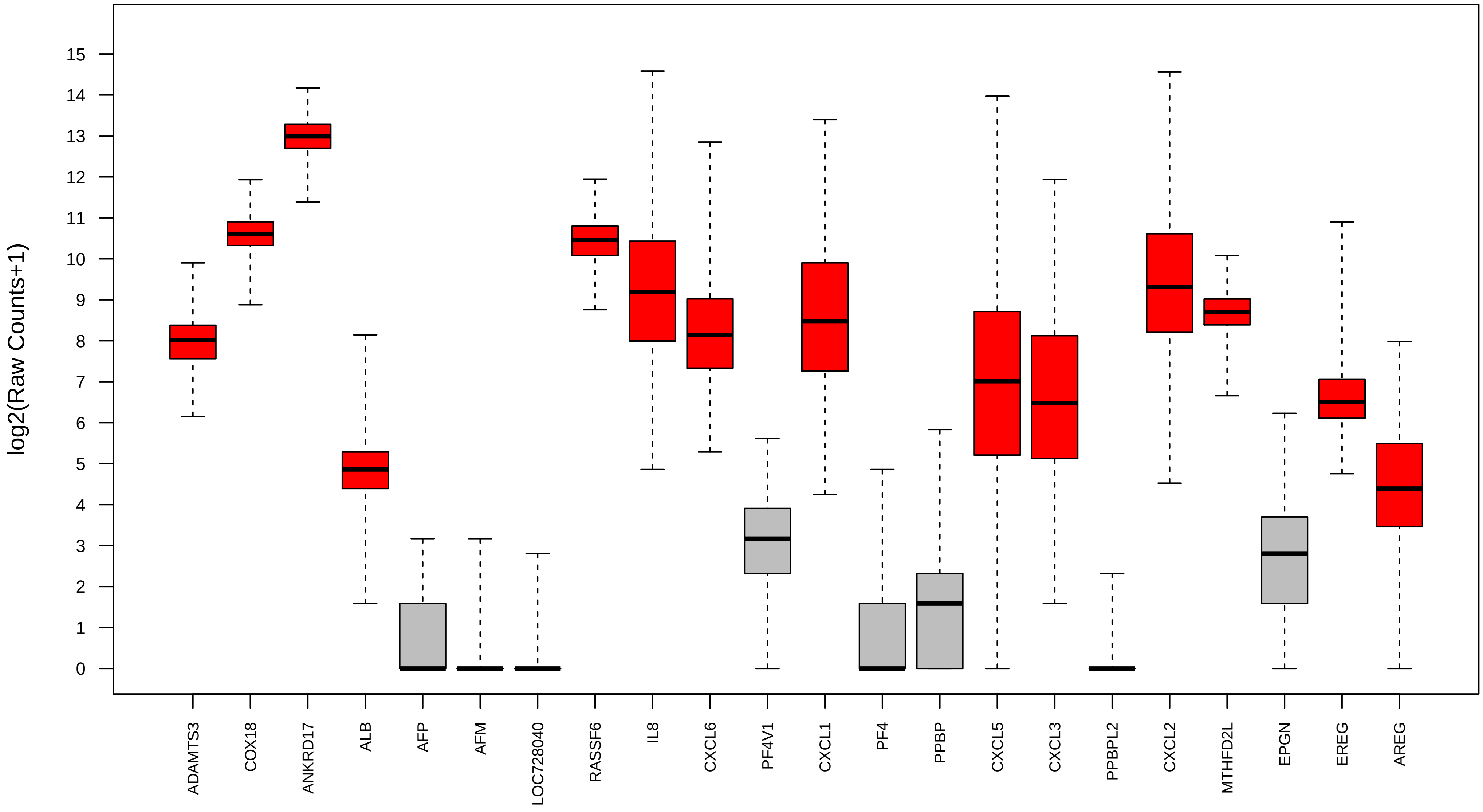

Region25, chr4.95414609.95662877
rs12500426,rs17021918
Total Genes: 7

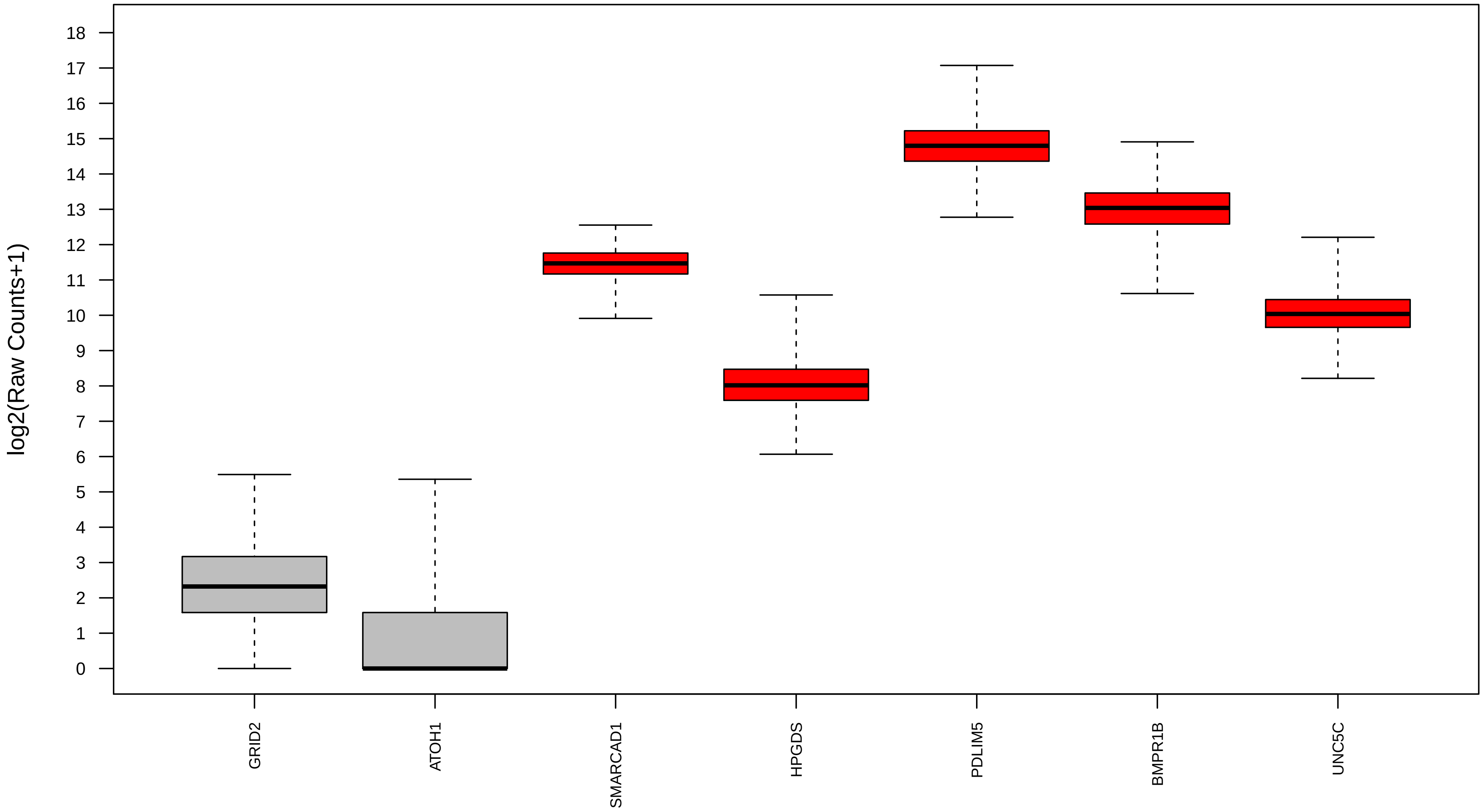

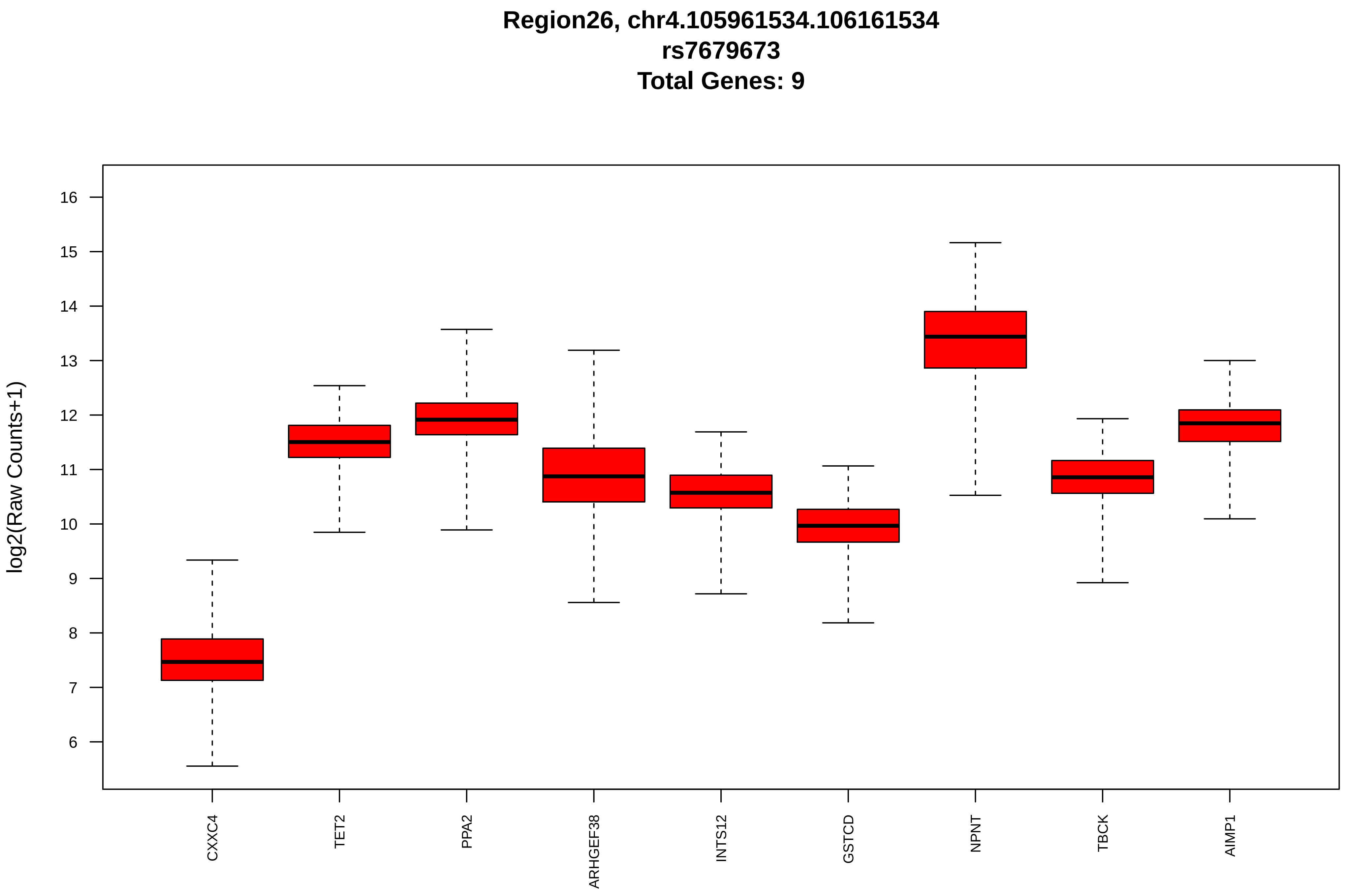
Region26, chr4.105961534.106161534
rs7679673
Total Genes: 9
log2(Raw Counts+1)
16
15
14
13
12
11
10
9
8
7
6
CXXC4
TET2
PPA2
ARHGEF38
INTS12
GSTCD
NPNT
TBCK
AIMP1

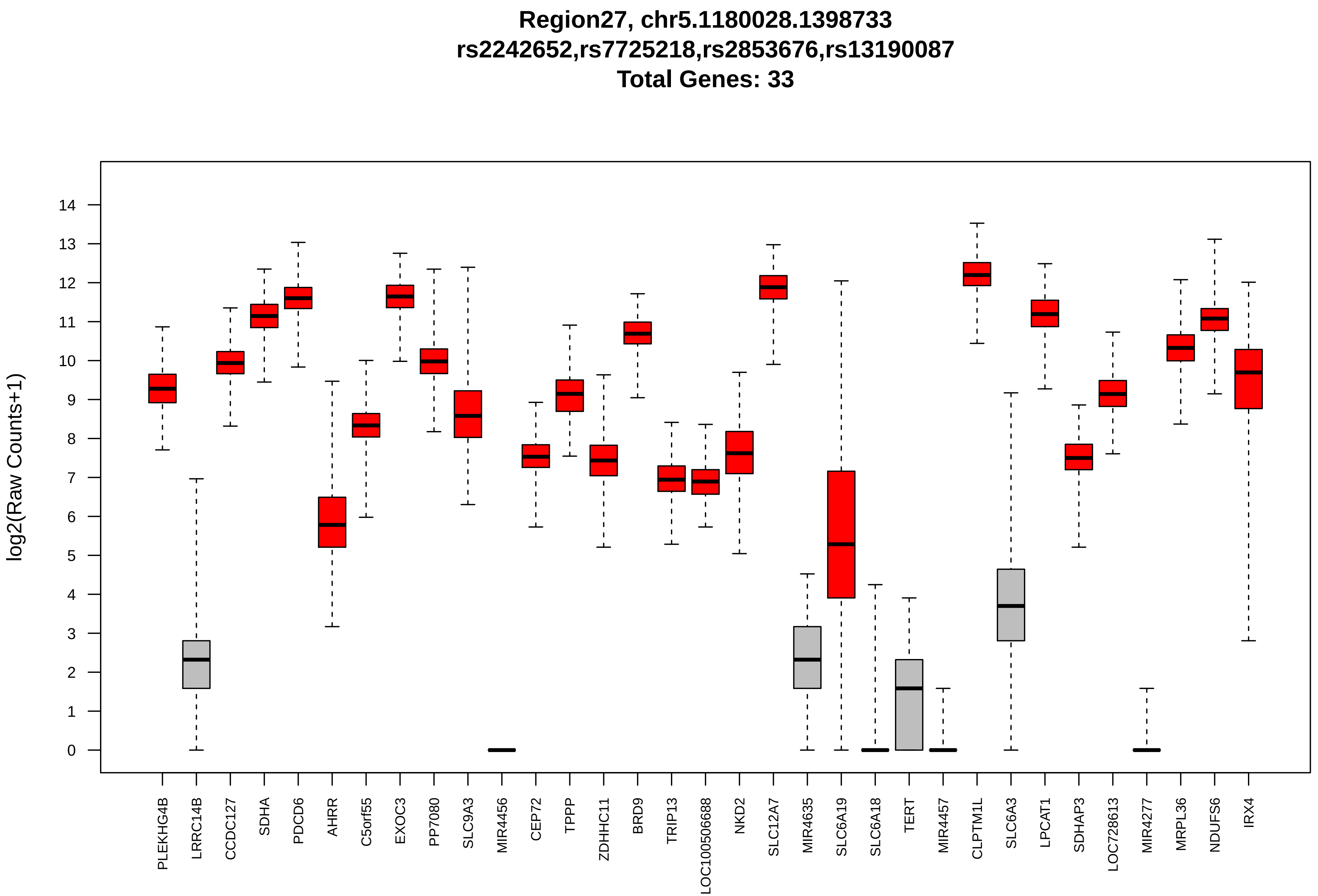

Region27, chr5.1180028.1398733
rs2242652,rs7725218,rs2853676,rs13190087
Total Genes: 33
log2(Raw Counts+1)
0
1
2
3
4
5
6
7
8
9
10
11
12
13
14
PLEKHG4B
LRRC14B
CCDC127
SDHA
PDCD6
AHRR
C5orf55
EXOC3
PP7080
SLC9A3
MIR4456
CEP72
TPPP
ZDHHC11
BRD9
TRIP13
LOC100506688
NKD2
SLC12A7
MIR4635
SLC6A19
SLC6A18
TERT
MIR4457
CLPTM1L
SLC6A3
LPCAT1
SDHAP3
LOC728613
MIR4277
MRPL36
NDUFS6
IRX4

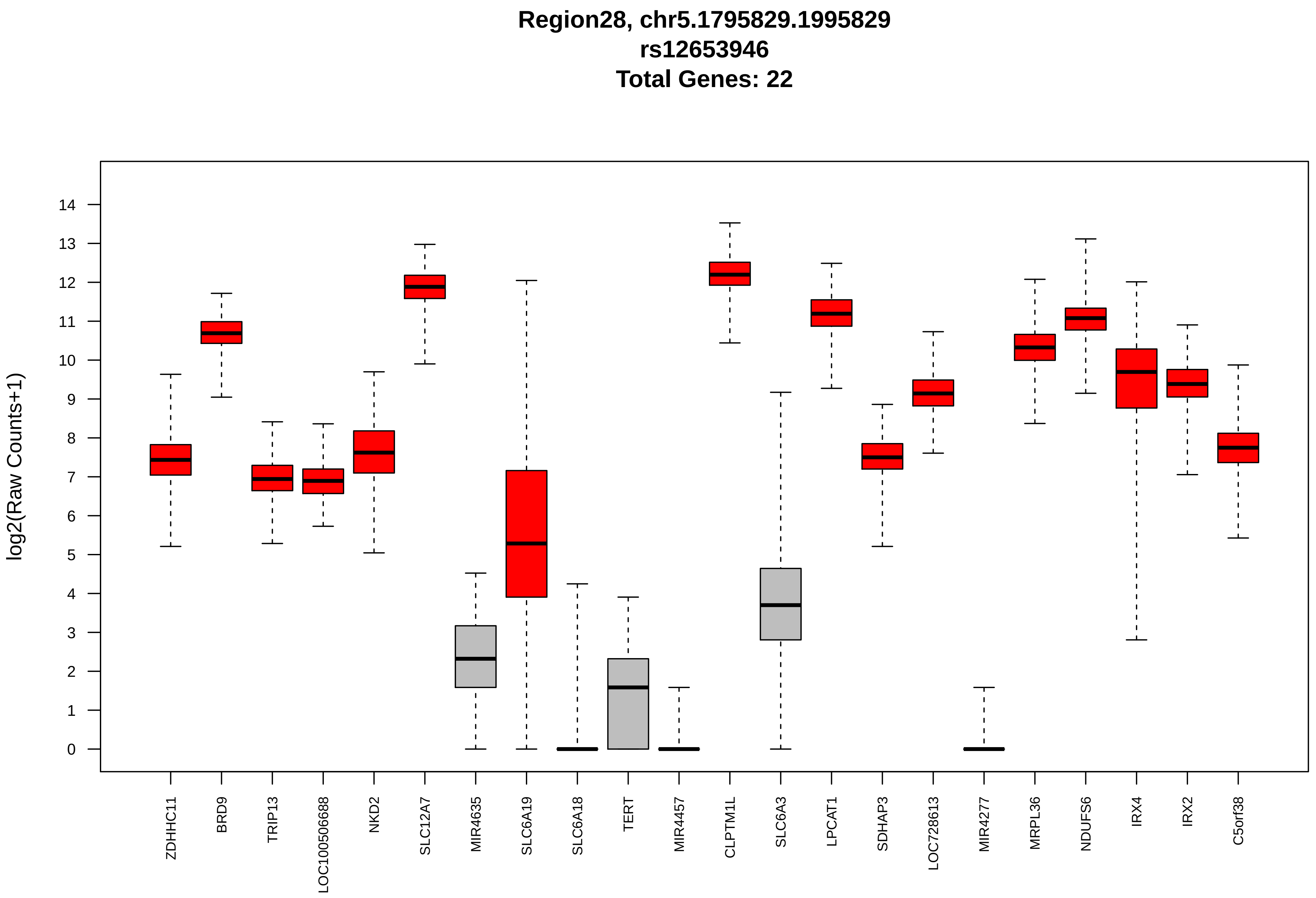

Region28, chr5.1795829.1995829
rs12653946
Total Genes: 22
log2(Raw Counts+1)
0
1
2
3
4
5
6
7
8
9
10
11
12
13
14
ZDHHC11
BRD9
TRIP13
LOC100506688
NKD2
SLC12A7
MIR4635
SLC6A19
SLC6A18
TERT
MIR4457
CLPTM1L
SLC6A3
LPCAT1
SDHAP3
LOC728613
MIR4277
MRPL36
NDUFS6
IRX4
IRX2
C5orf38

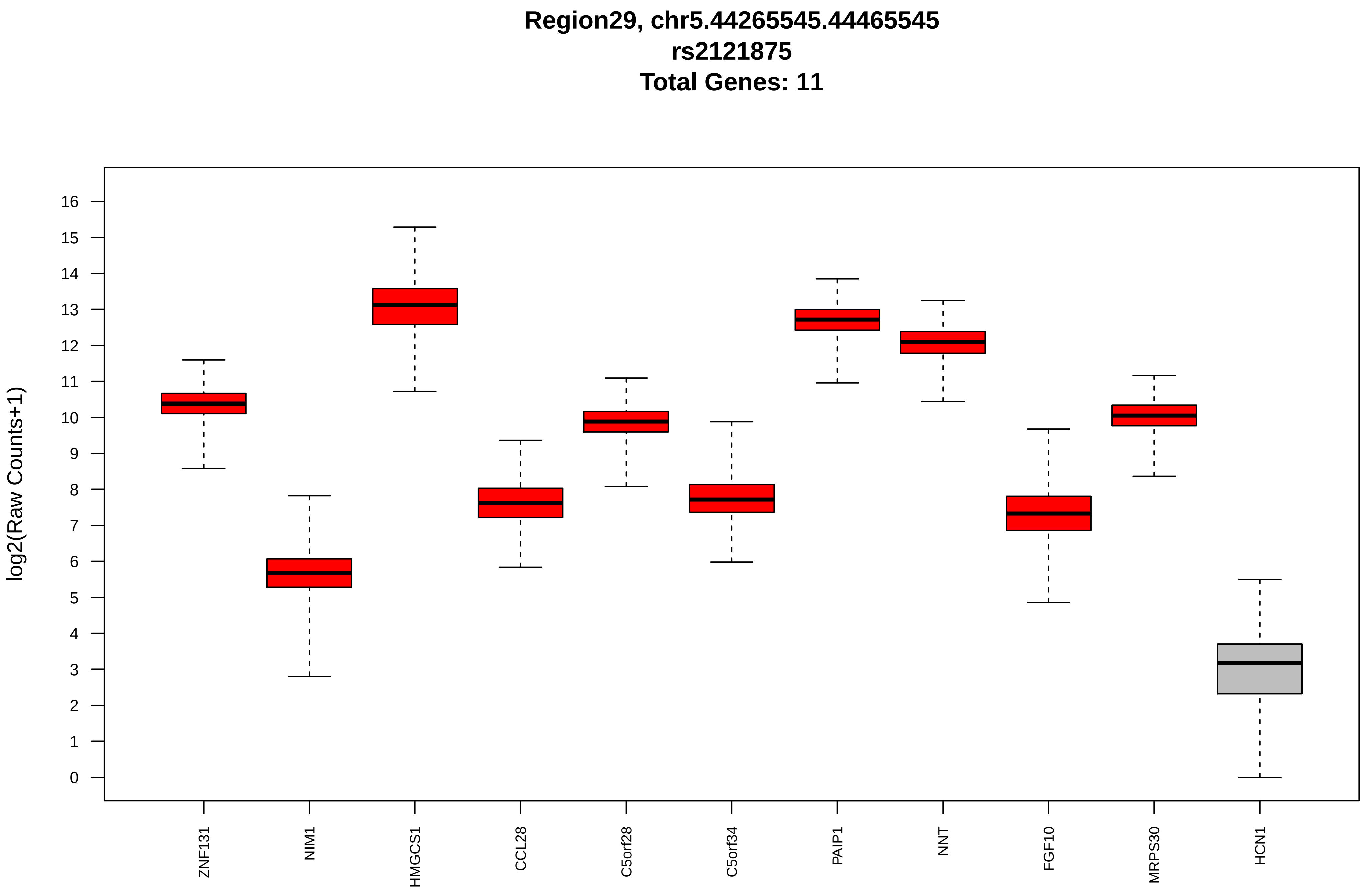
Region29, chr5.44265545.44465545
rs2121875
Total Genes: 11
log2(Raw Counts+1)
0
1
2
3
4
5
6
7
8
9
10
11
12
13
14
15
16
ZNF131
NIM1
HMGCS1
CCL28
C5orf28
C5orf34
PAIP1
NNT
FGF10
MRPS30
HCN1

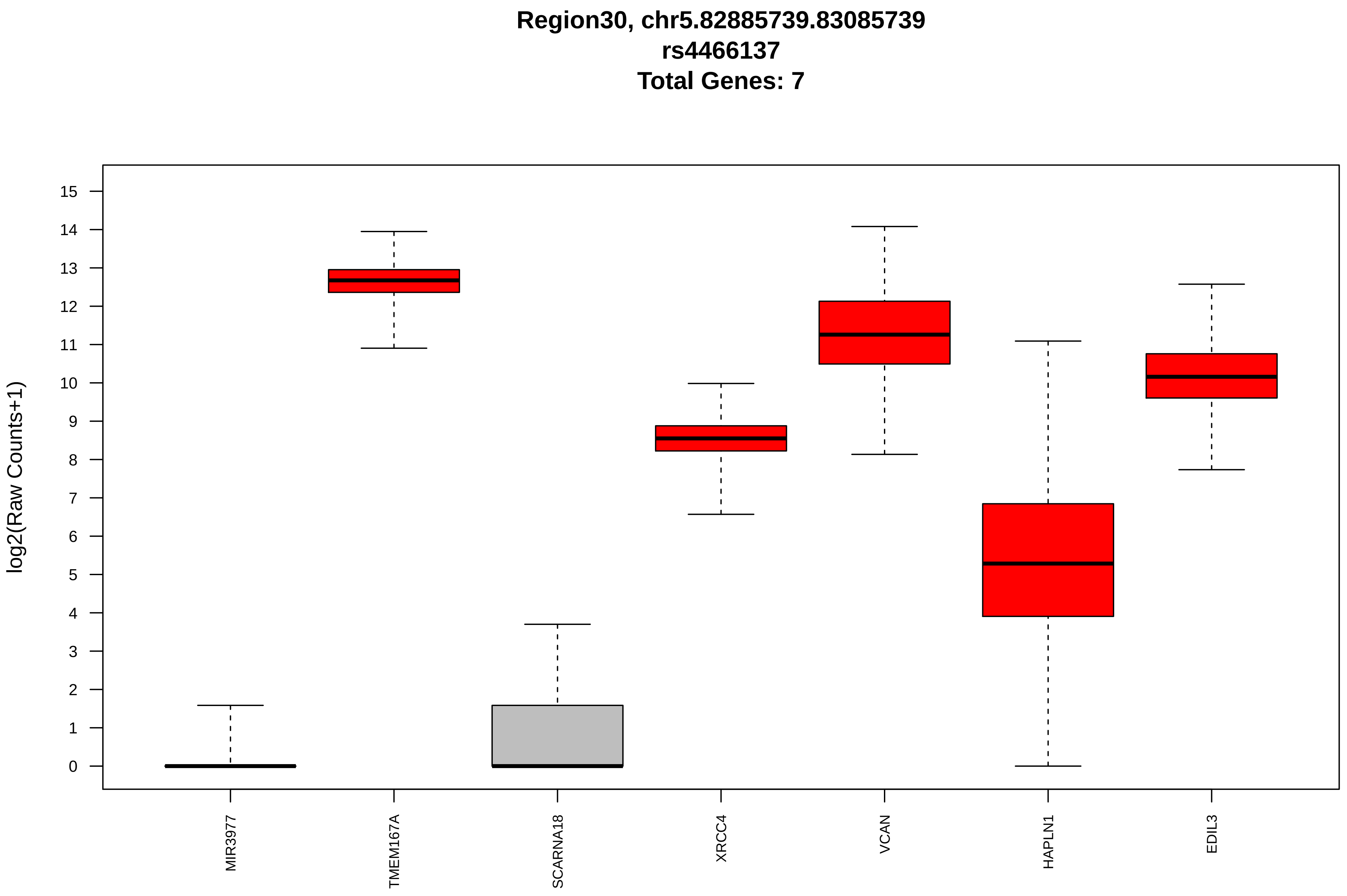

Region30, chr5.82885739.83085739
rs4466137
Total Genes: 7
log2(Raw Counts+1)
0
1
2
3
4
5
6
7
8
9
10
11
12
13
14
15
MIR3977
TMEM167A
SCARNA18
XRCC4
VCAN
HAPLN1
EDIL3

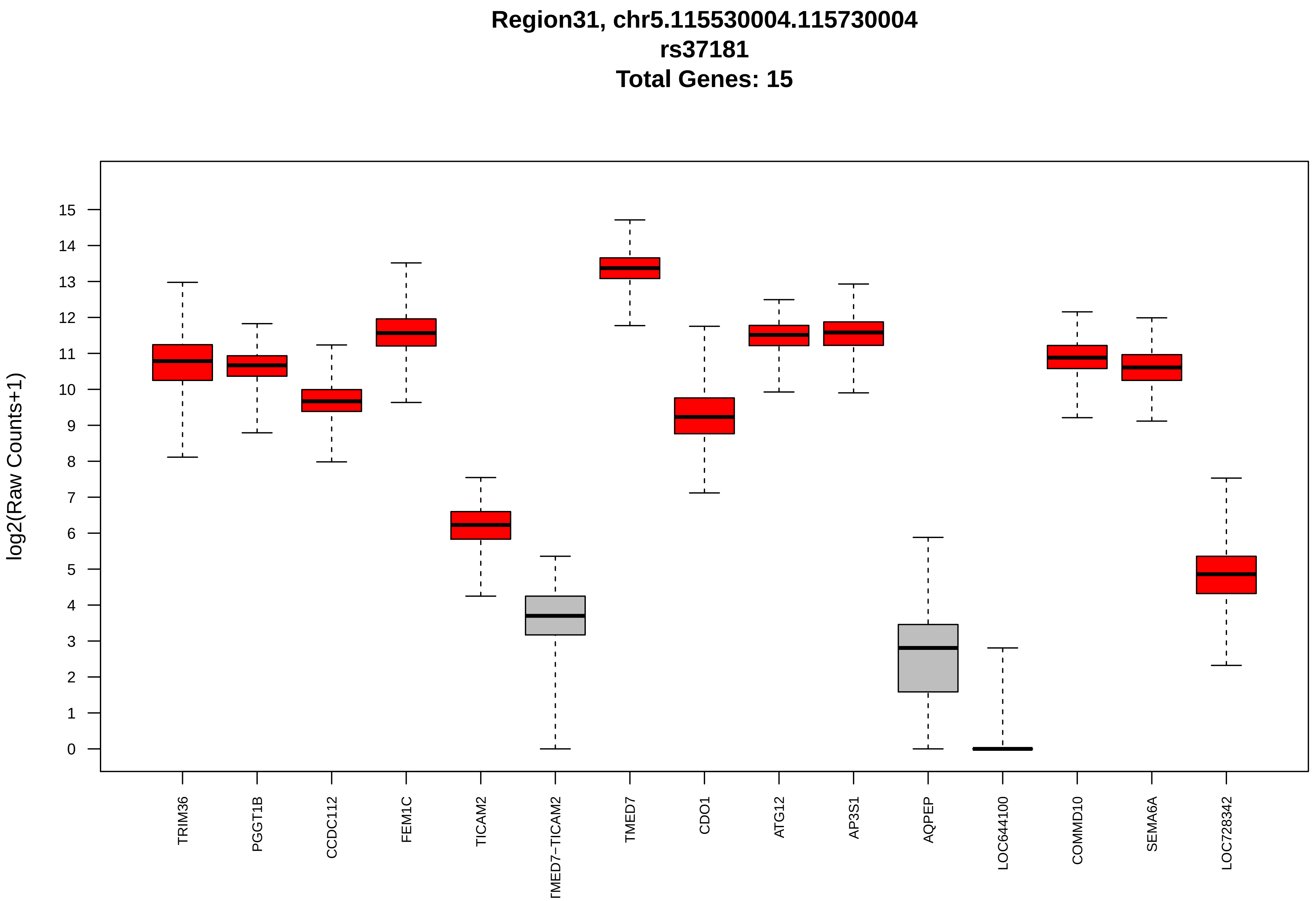
Region31, chr5.115530004.115730004
rs37181
Total Genes: 15
log2(Raw Counts+1)
0
1
2
3
4
5
6
7
8
9
10
11
12
13
14
15
TRIM36
PGGT1B
CCDC112
FEM1C
TICAM2
TMED7–TICAM2
TMED7
CDO1
ATG12
AP3S1
AQPEP
LOC644100
COMMD10
SEMA6A
LOC728342

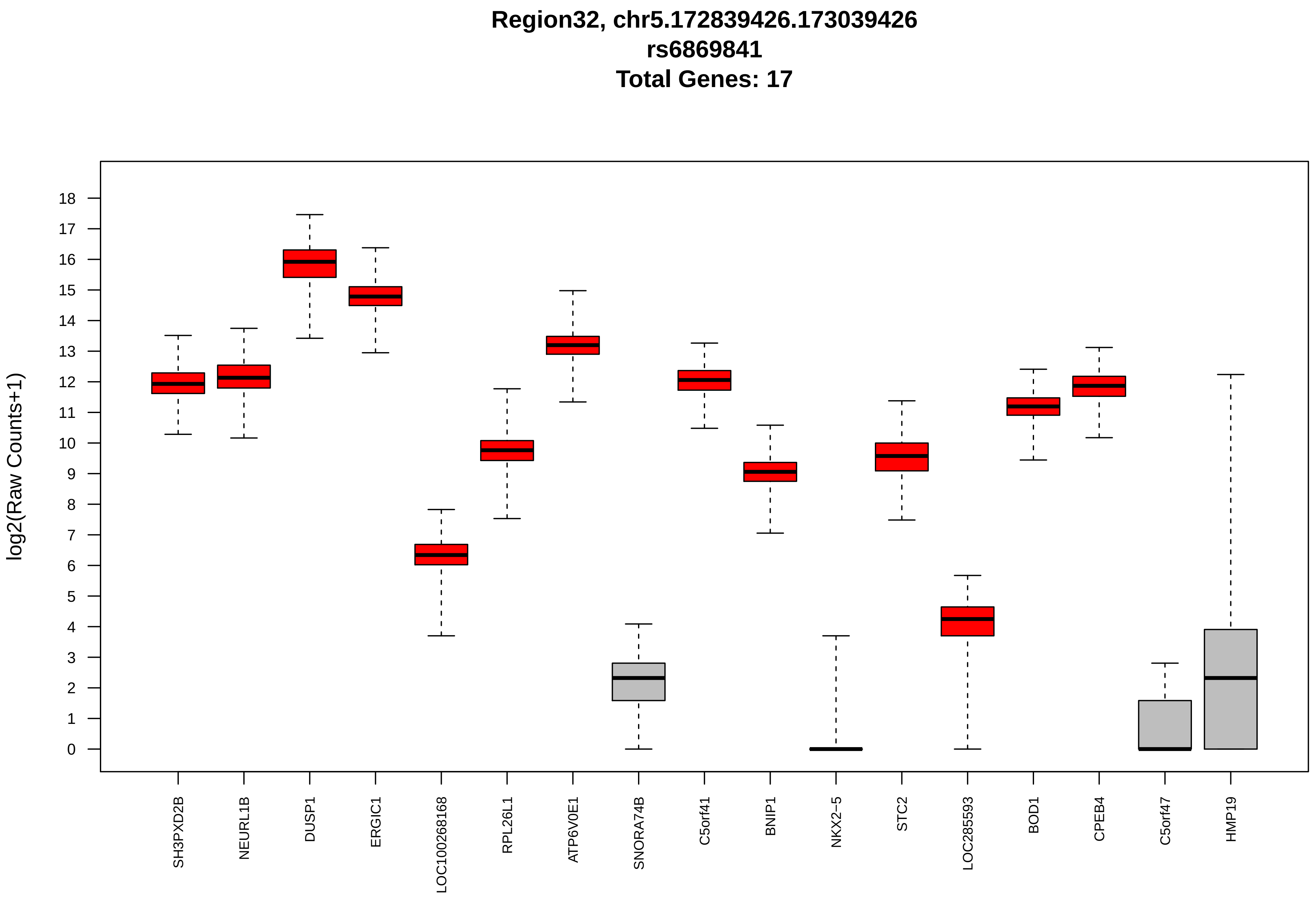

Region32, chr5.172839426.173039426
rs6869841
Total Genes: 17
log2(Raw Counts+1)
0
1
2
3
4
5
6
7
8
9
10
11
12
13
14
15
16
17
18
SH3PXD2B
NEURL1B
DUSP1
ERGIC1
LOC100268168
RPL26L1
ATP6V0E1
SNORA74B
C5orf41
BNIP1
NKX2−5
STC2
LOC285593
BOD1
CPEB4
C5orf47
HMP19

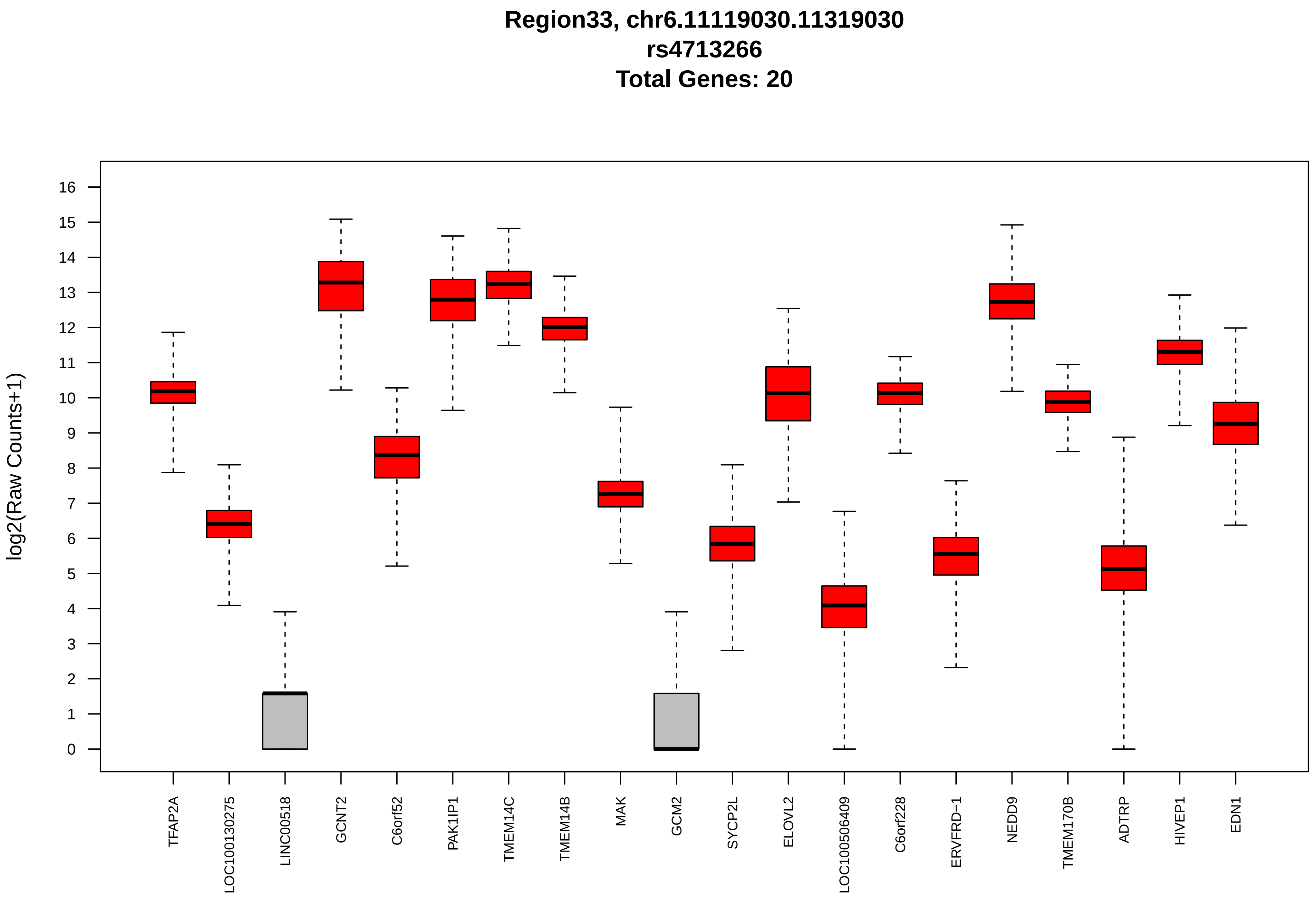
Region33, chr6.11119030.11319030
rs4713266
Total Genes: 20
log2(Raw Counts+1)
0
1
2
3
4
5
6
7
8
9
10
11
12
13
14
15
16
TFAP2A
LOC100130275
LINC00518
GCNT2
C6orf52
PAK1IP1
TMEM14C
TMEM14B
MAK
GCM2
SYCP2L
ELOVL2
LOC100506409
C6orf228
ERVFRD−1
NEDD9
TMEM170B
ADTRP
HIVEP1
EDN1

Region34, chr6.29973776.30173776
rs7767188
Total Genes: 82

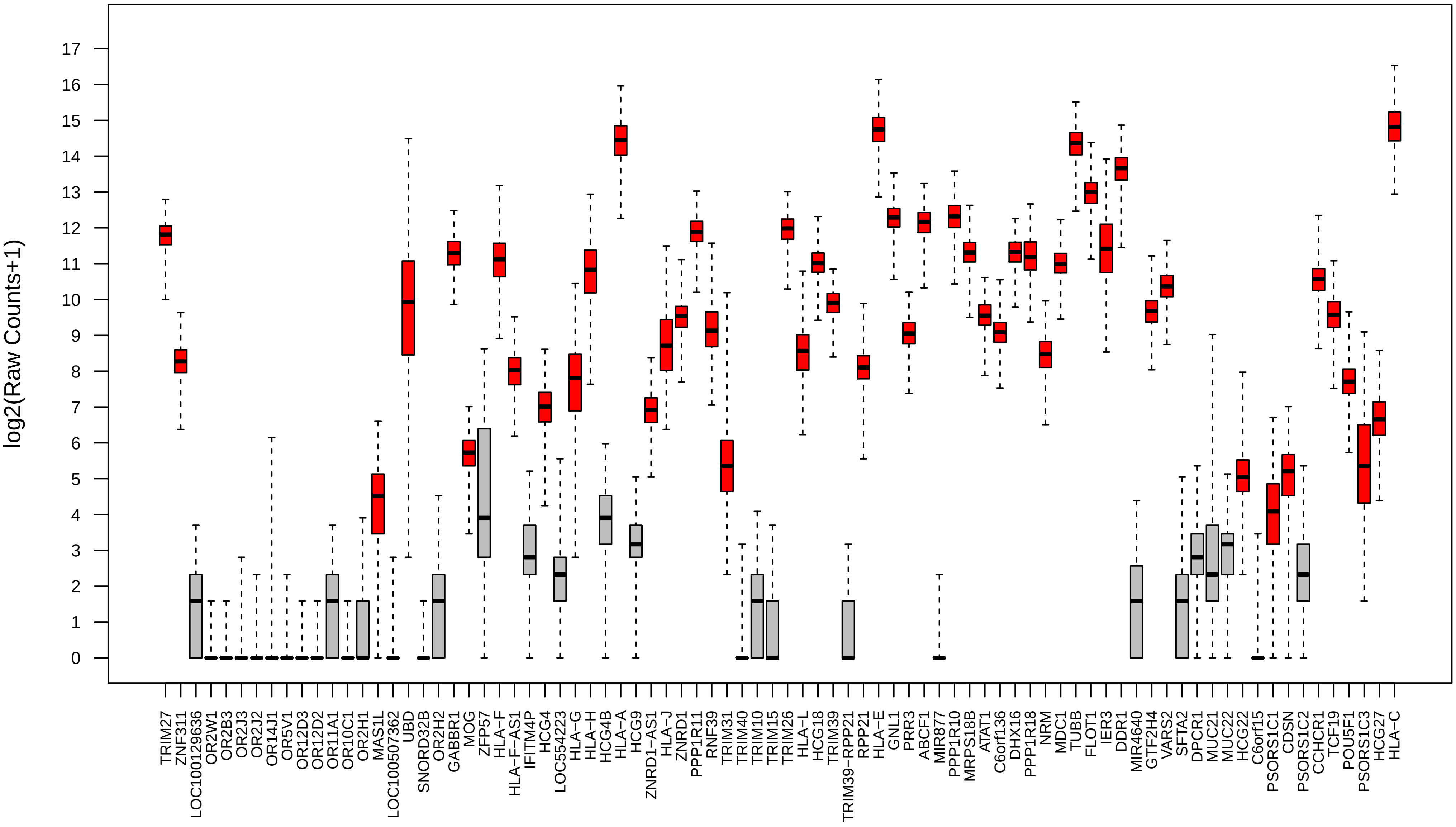

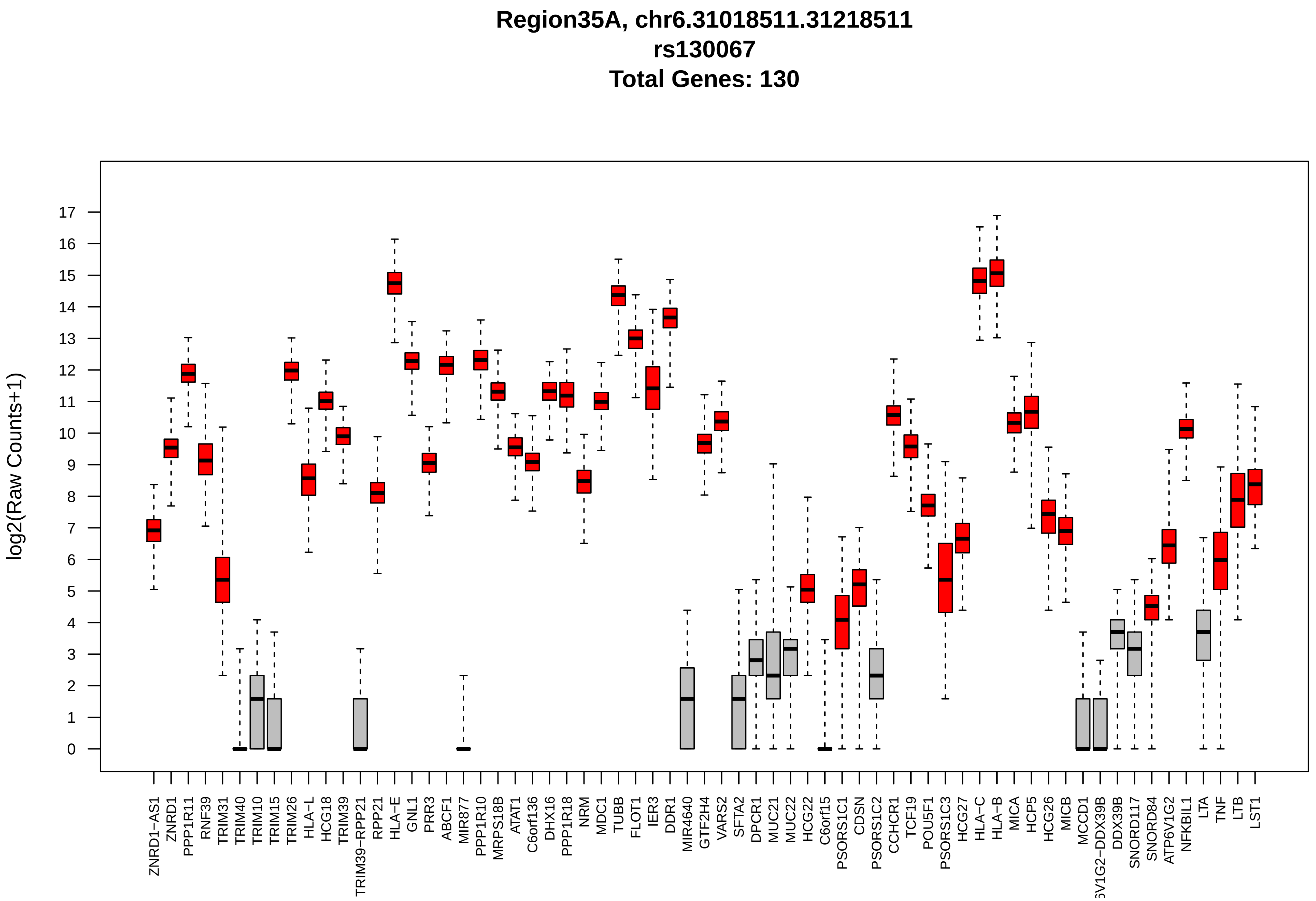

Region35A, chr6.31018511.31218511
rs130067
Total Genes: 130
log2(Raw Counts+1)
0
1
2
3
4
5
6
7
8
9
10
11
12
13
14
15
16
17
ZNRD1−AS1
ZNRD1
PPP1R11
RNF39
TRIM31
TRIM40
TRIM10
TRIM15
TRIM26
HLA−L
HCG18
TRIM39
TRIM39−RPP21
RPP21
HLA−E
GNL1
PRR3
ABCF1
MIR877
PPP1R10
MRPS18B
ATAT1
C6orf136
DHX16
PPP1R18
NRM
MDC1
TUBB
FLOT1
IER3
DDR1
MIR4640
GTF2H4
VARS2
SFTA2
DPCR1
MUC21
MUC22
HCG22
C6orf15
PSORS1C1
CDSN
PSORS1C2
CCHCR1
TCF19
POU5F1
PSORS1C3
HCG27
HLA−C
HLA−B
MICA
HCP5
HCG26
MICB
MCCD1
DDX39B
SNORD117
SNORD84
ATP6V1G2
NFKBIL1
LTA
TNF
LTB
LST1

Region35B, chr6.31018511.31218511
rs130067
Total Genes: 130

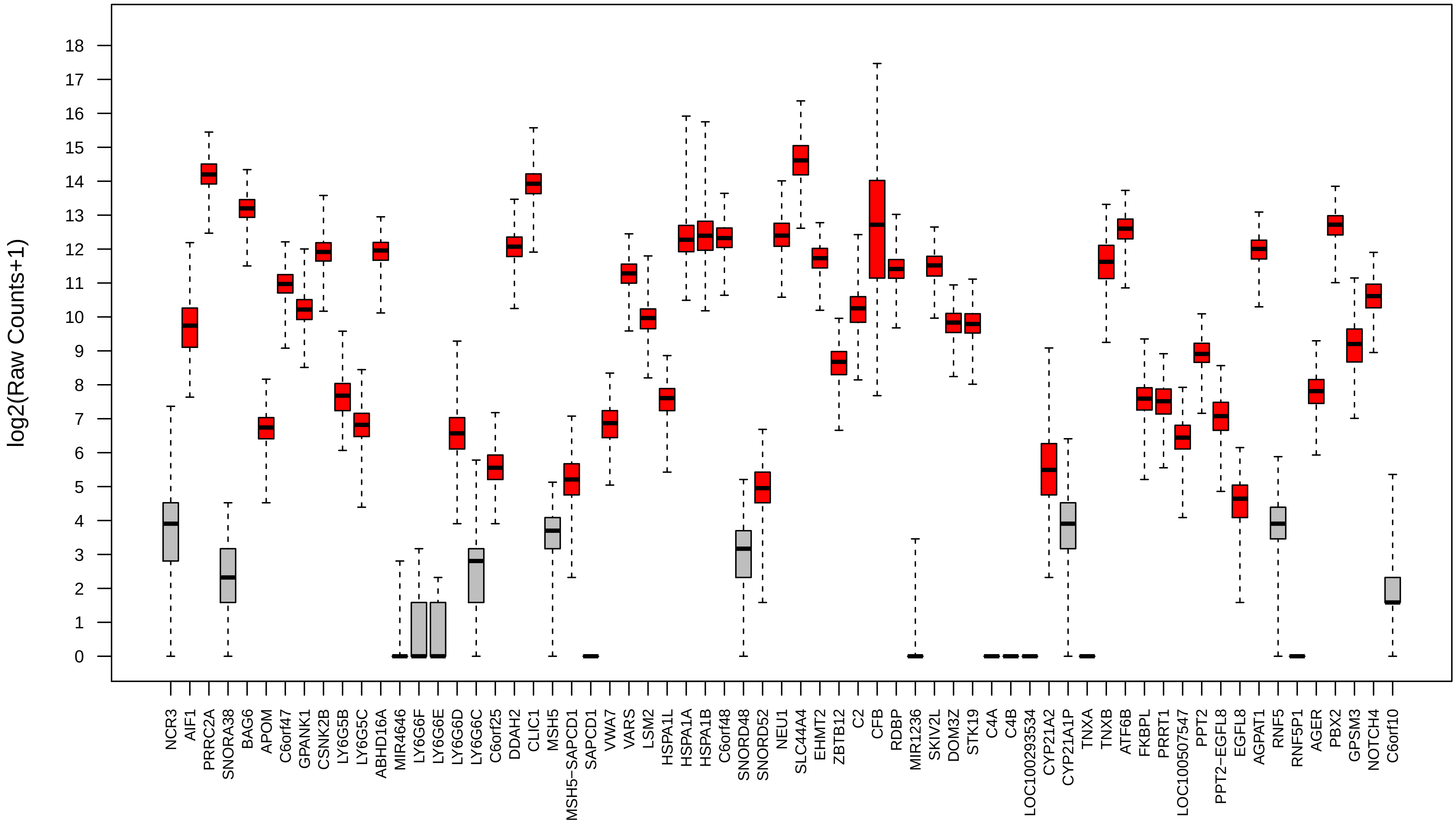

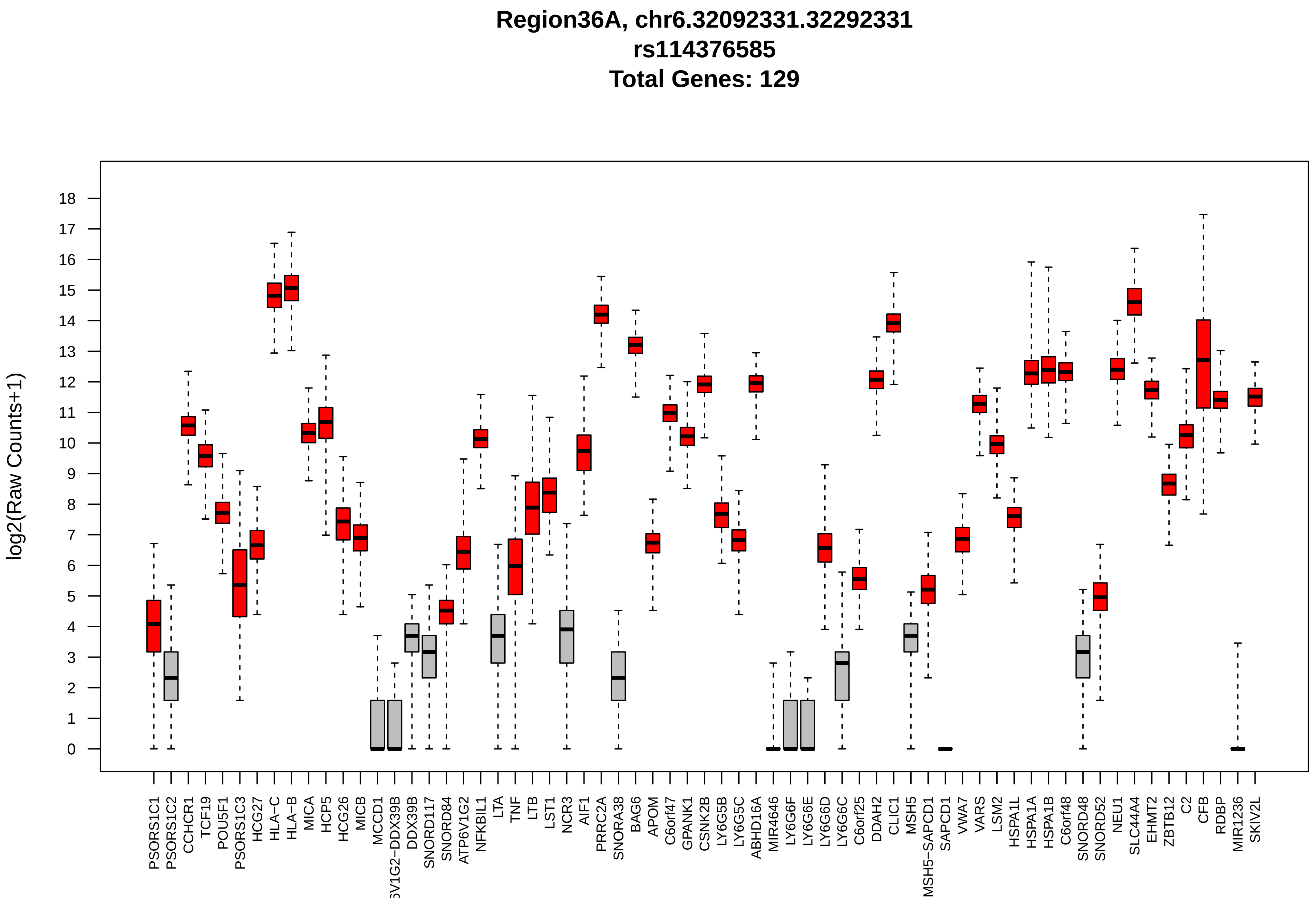
Region36A, chr6.32092331.32292331
rs114376585
Total Genes: 129
log2(Raw Counts+1)
0
1
2
3
4
5
6
7
8
9
10
11
12
13
14
15
16
17
18
PSORS1C1
PSORS1C2
CCHCR1
TCF19
POU5F1
PSORS1C3
HCG27
HLA−C
HLA−B
MICA
HCP5
HCG26
MICB
MCCD1
DDX39B
SNORD117
SNORD84
ATP6V1G2
NFKBIL1
LTA
TNF
LTB
LST1
NCR3
AIF1
PRRC2A
SNORA38
BAG6
APOM
C6orf47
GPANK1
CSNK2B
LY6G5B
LY6G5C
ABHD16A
MIR4646
LY6G6F
LY6G6E
LY6G6D
LY6G6C
C6orf25
DDAH2
CLIC1
MSH5
MSH5−SAPCD1
SAPCD1
VWA7
VARS
LSM2
HSPA1L
HSPA1A
HSPA1B
C6orf48
SNORD48
SNORD52
NEU1
SLC44A4
EHMT2
ZBTB12
C2
CFB
RDBP
MIR1236
SKIV2L

Region36B, chr6.32092331.32292331
rs114376585
Total Genes: 129

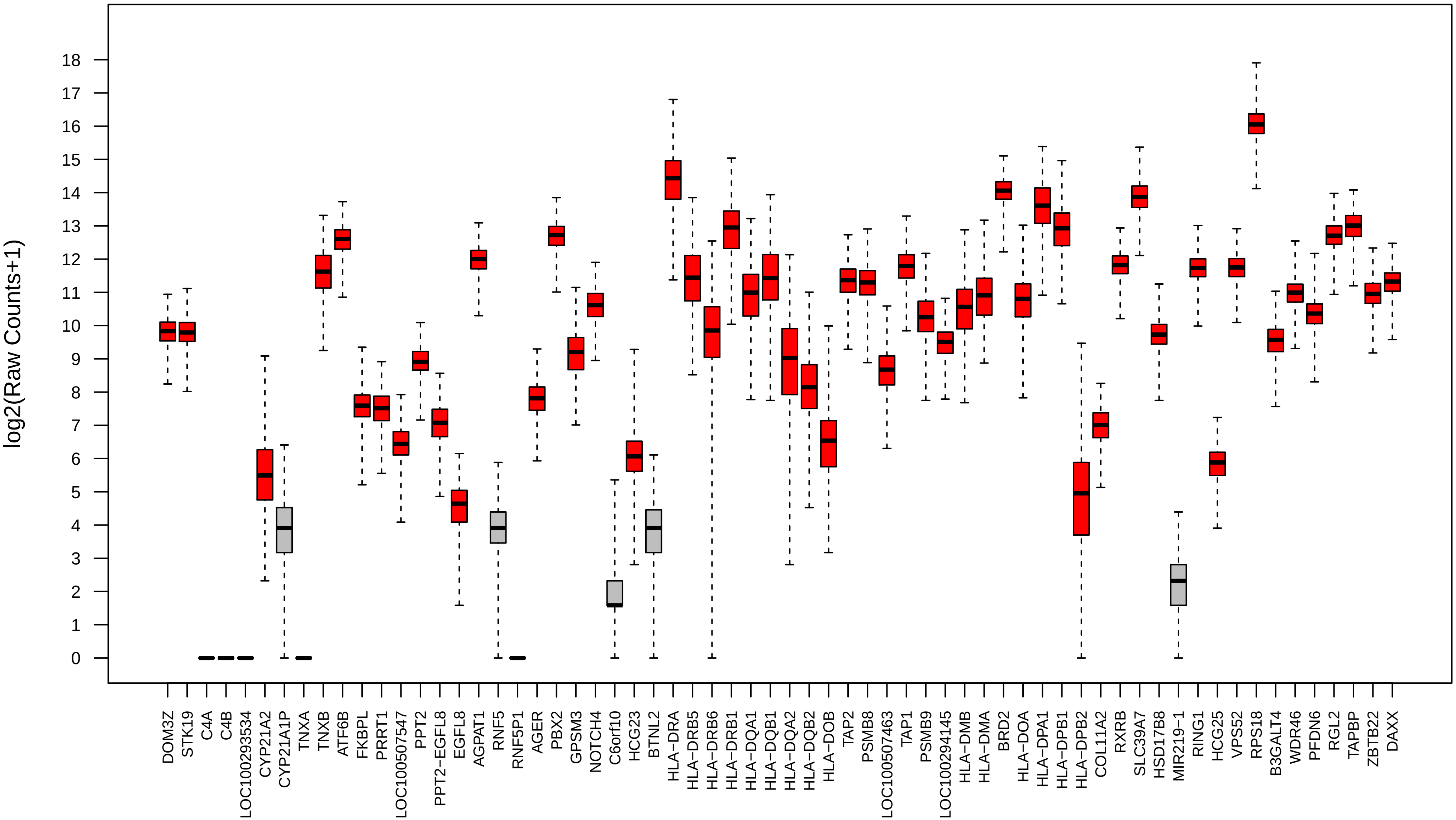

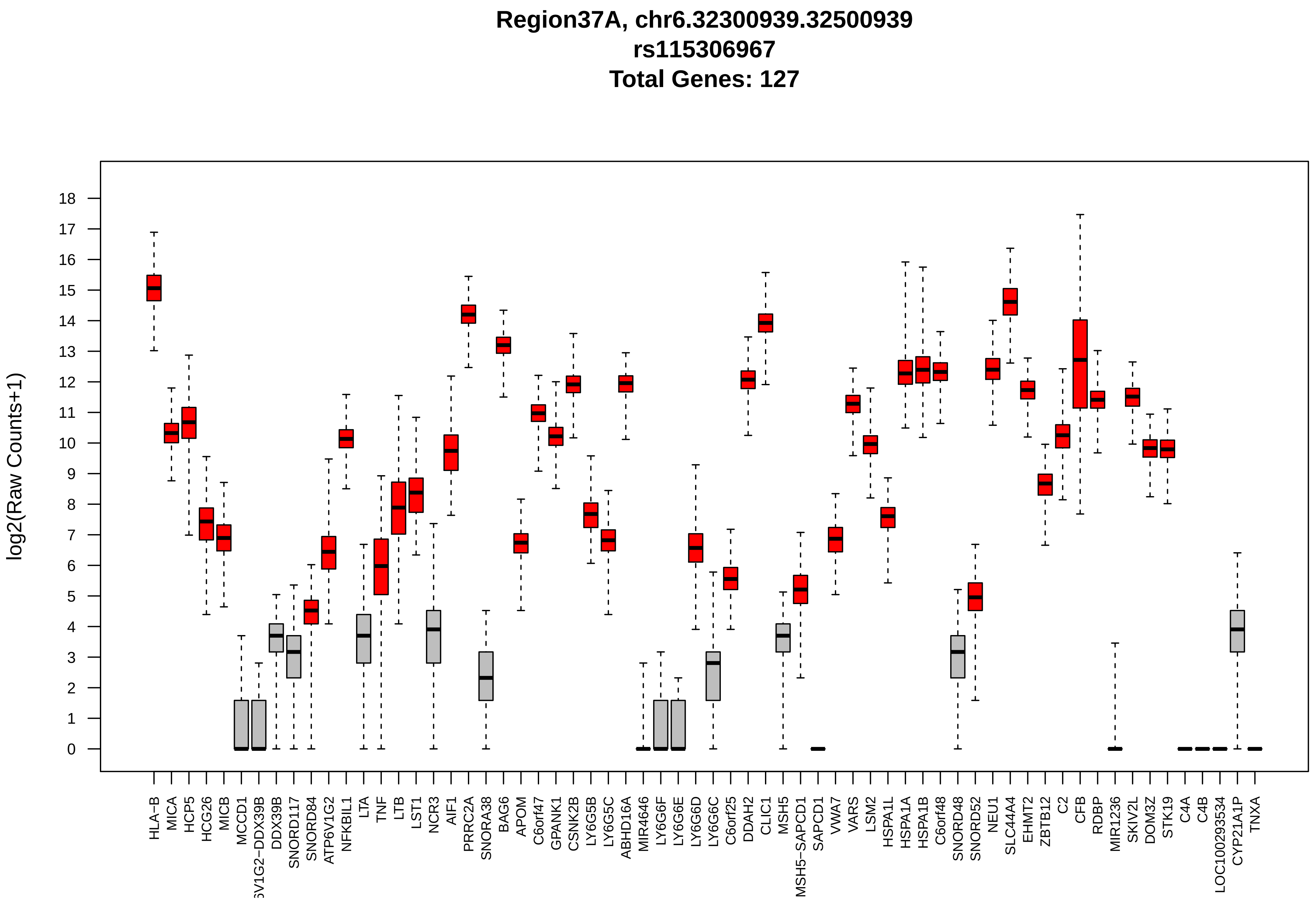
Region37A, chr6.32300939.32500939
rs115306967
Total Genes: 127
log2(Raw Counts+1)
0
1
2
3
4
5
6
7
8
9
10
11
12
13
14
15
16
17
18
HLA−B
MICA
HCP5
HCG26
MICB
MCCD1
DDX39B
SNORD117
SNORD84
ATP6V1G2
NFKBIL1
LTA
TNF
LTB
LST1
NCR3
AIF1
PRRC2A
SNORA38
BAG6
APOM
C6orf47
GPANK1
CSNK2B
LY6G5B
LY6G5C
ABHD16A
MIR4646
LY6G6F
LY6G6E
LY6G6D
LY6G6C
C6orf25
DDAH2
CLIC1
MSH5
MSH5−SAPCD1
SAPCD1
VWA7
VARS
LSM2
HSPA1L
HSPA1A
HSPA1B
C6orf48
SNORD48
SNORD52
NEU1
SLC44A4
EHMT2
ZBTB12
C2
CFB
RDBP
MIR1236
SKIV2L
DOM3Z
STK19
C4A
C4B
LOC100293534
CYP21A1P
TNXA

Region37B, chr6.32300939.32500939
rs115306967
Total Genes: 127

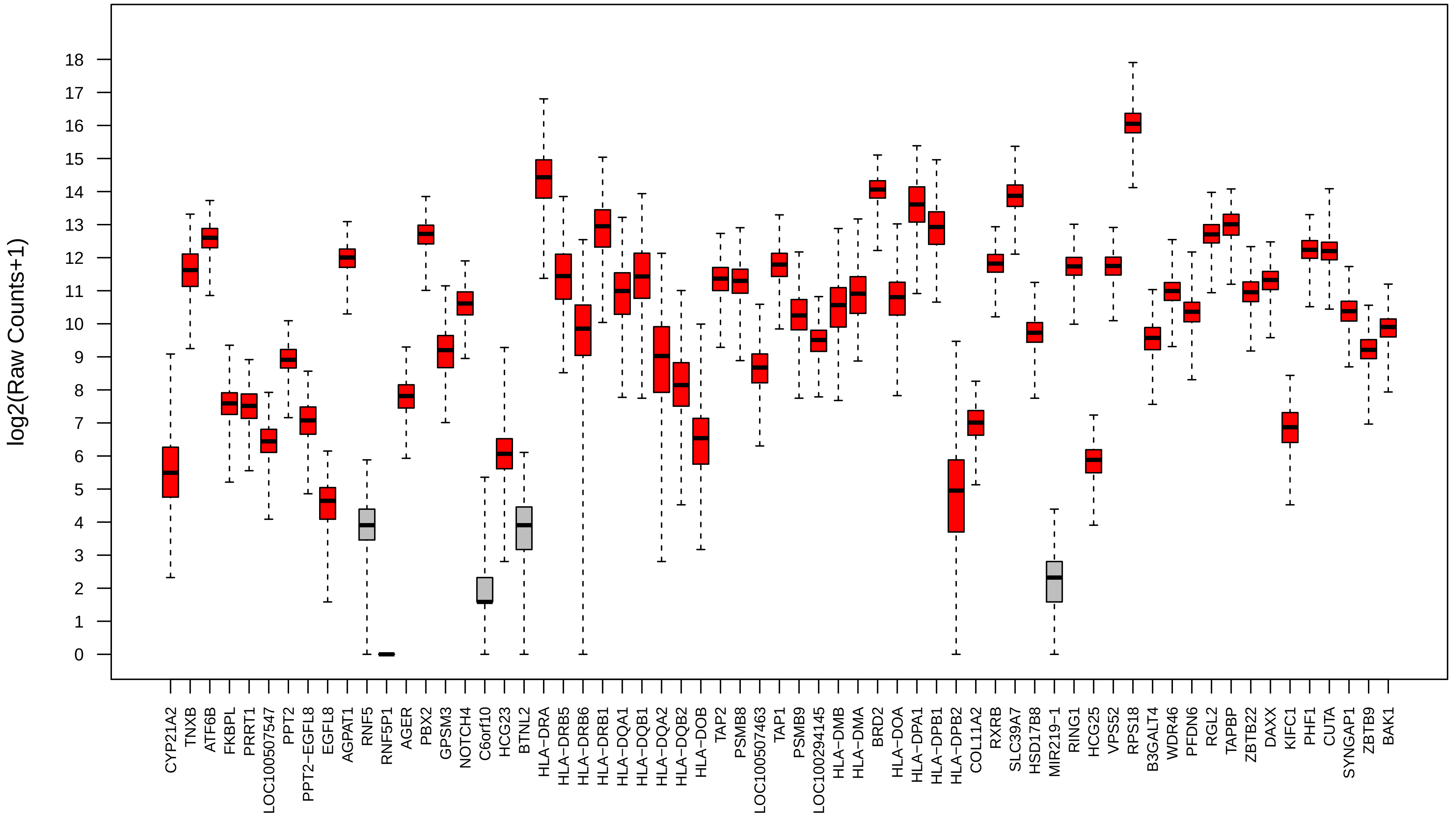

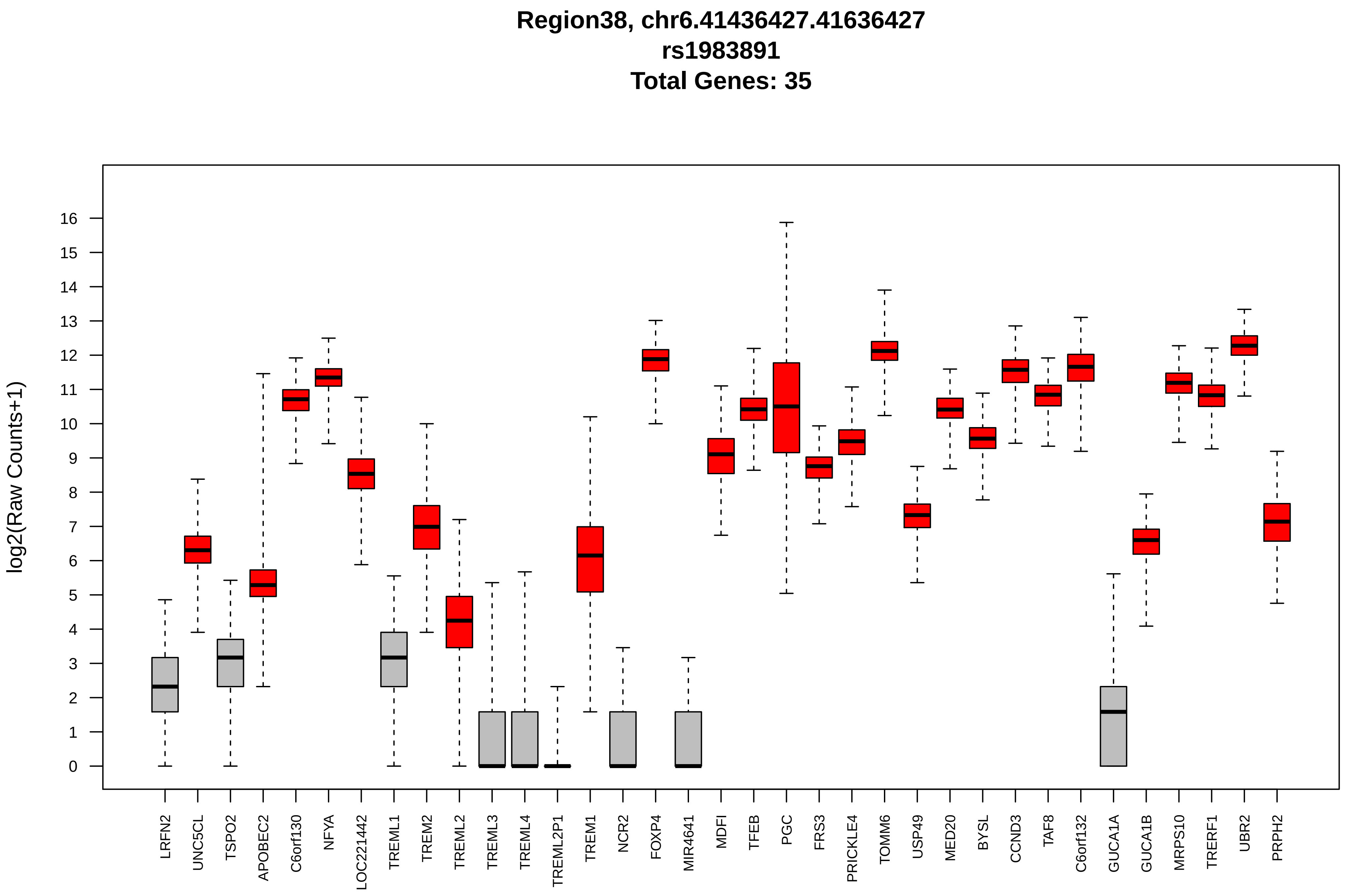
Region38, chr6.41436427.41636427
rs1983891
Total Genes: 35
log2(Raw Counts+1)
0
1
2
3
4
5
6
7
8
9
10
11
12
13
14
15
16
LRFN2
UNC5CL
TSPO2
APOBEC2
C6orf130
NFYA
LOC221442
TREML1
TREM2
TREML2
TREML3
TREML4
TREML2P1
TREM1
NCR2
FOXP4
MIR4641
MDFI
TFEB
PGC
FRS3
PRICKLE4
TOMM6
USP49
MED20
BYSL
CCND3
TAF8
C6orf132
GUCA1A
GUCA1B
MRPS10
TRERF1
UBR2
PRPH2

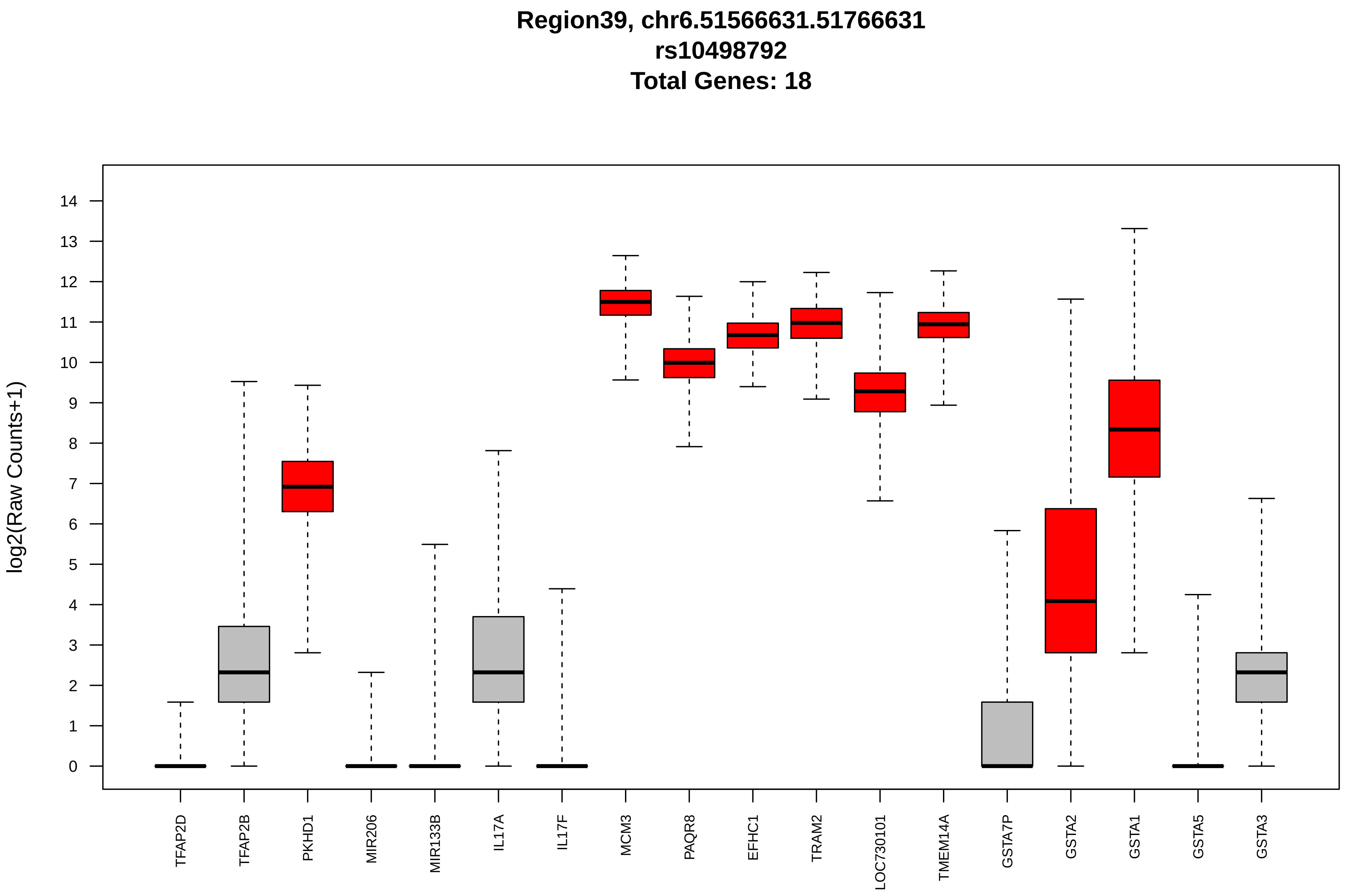
Region39, chr6.51566631.51766631
rs10498792
Total Genes: 18
log2(Raw Counts+1)
0
1
2
3
4
5
6
7
8
9
10
11
12
13
14
TFAP2D
TFAP2B
PKHD1
MIR206
MIR133B
IL17A
IL17F
MCM3
PAQR8
EFHC1
TRAM2
LOC730101
TMEM14A
GSTA7P
GSTA2
GSTA1
GSTA5
GSTA3

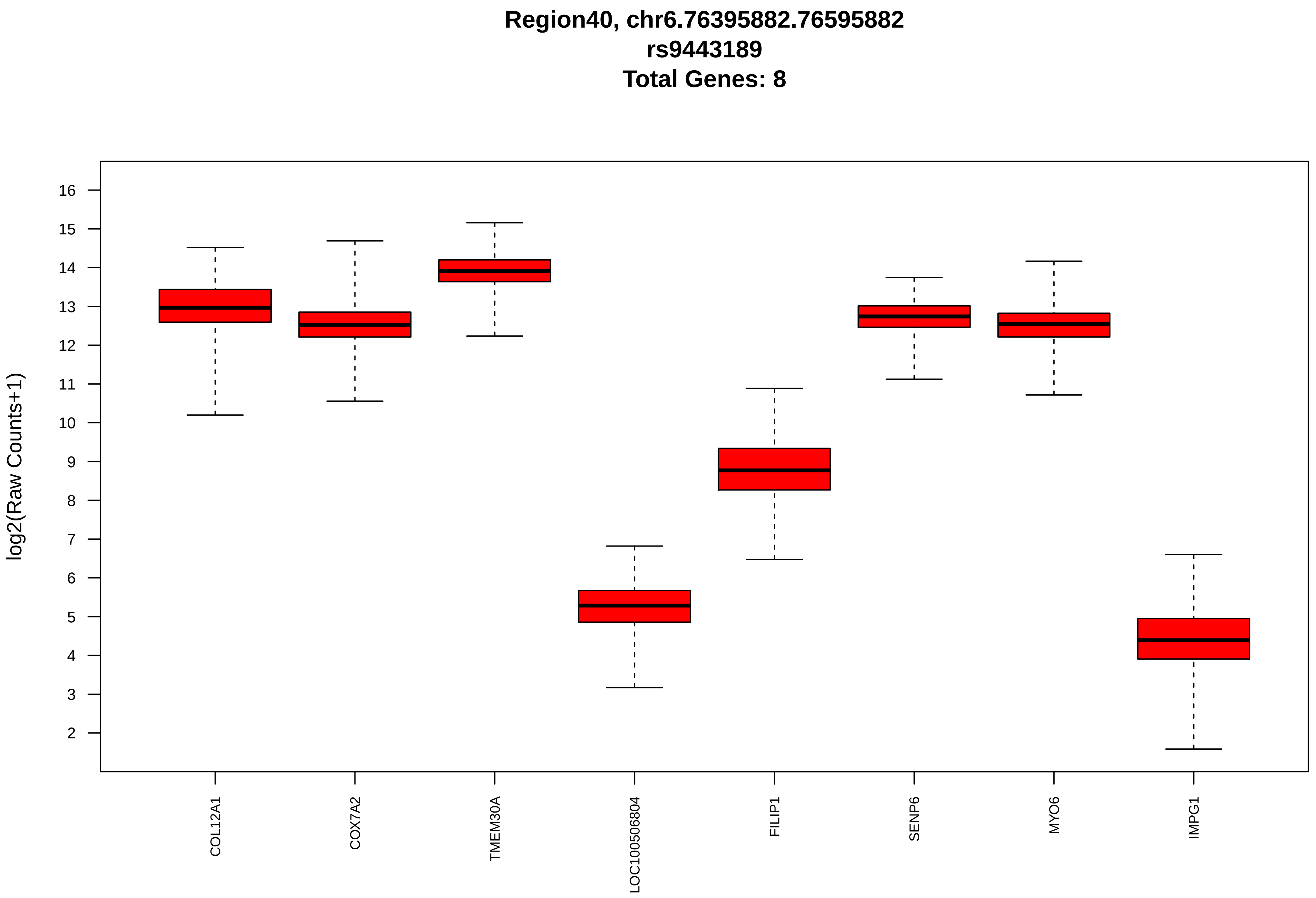

Region40, chr6.76395882.76595882
rs9443189
Total Genes: 8
log2(Raw Counts+1)
16
15
14
13
12
11
10
9
8
7
6
5
4
3
2
COL12A1
COX7A2
TMEM30A
LOC100506804
FILIP1
SENP6
MYO6
IMPG1

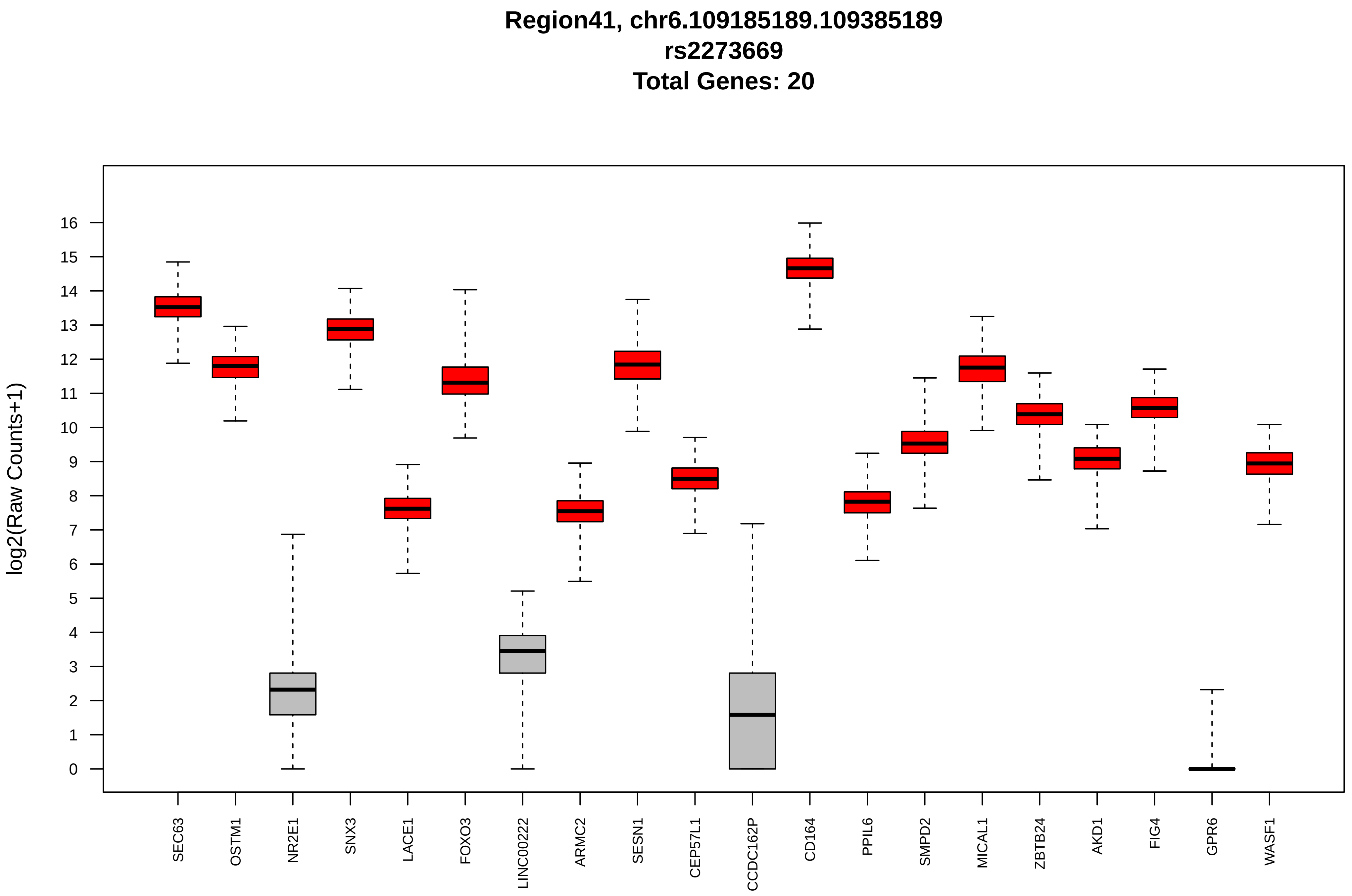

Region41, chr6.109185189.109385189
rs2273669
Total Genes: 20
log2(Raw Counts+1)
0
1
2
3
4
5
6
7
8
9
10
11
12
13
14
15
16
SEC63
OSTM1
NR2E1
SNX3
LACE1
FOXO3
LINC00222
ARMC2
SESN1
CEP57L1
CCDC162P
CD164
PPIL6
SMPD2
MICAL1
ZBTB24
AKD1
FIG4
GPR6
WASF1

Region42, chr6.117110052.117310052
rs339331
Total Genes: 24

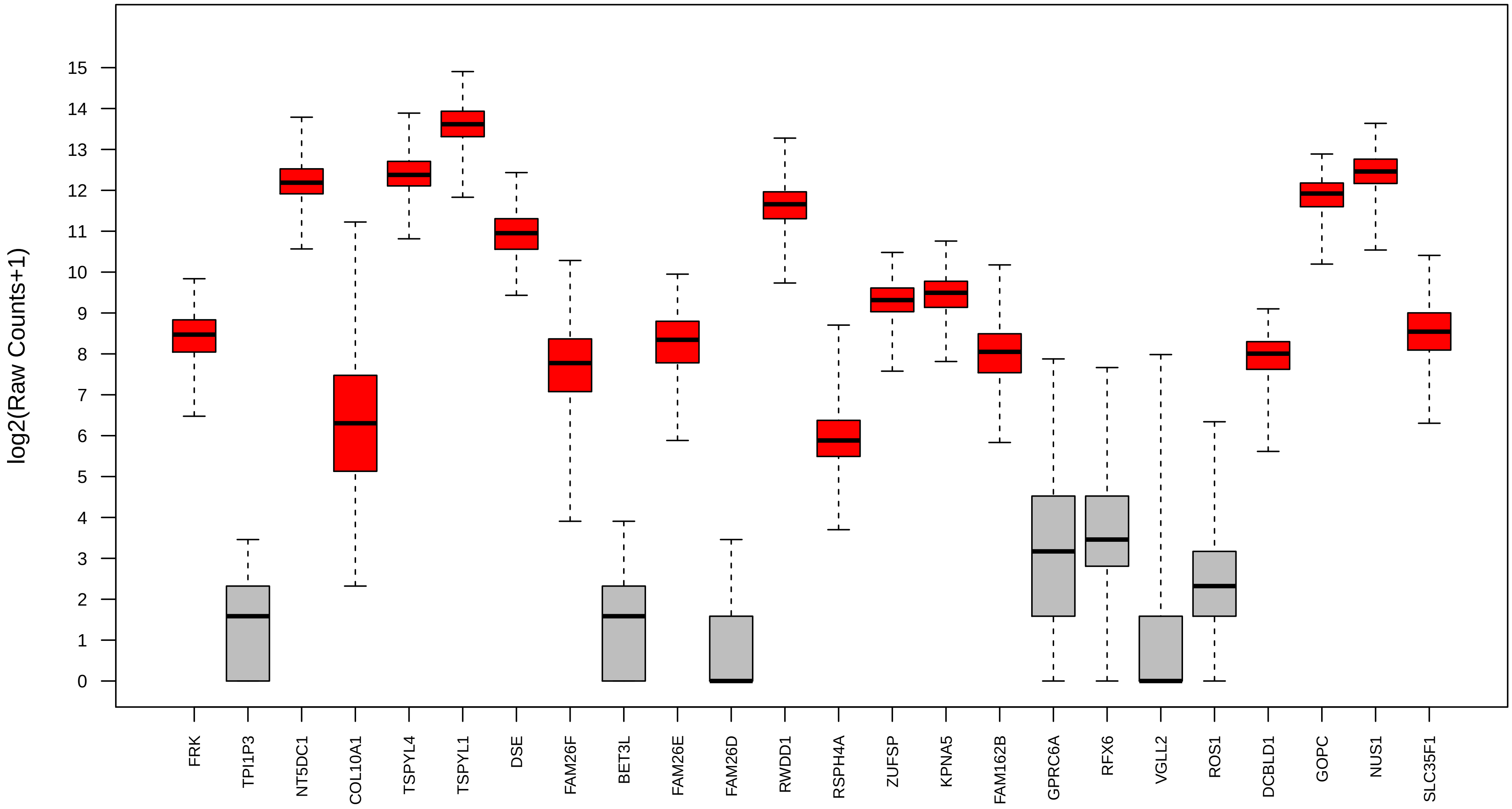

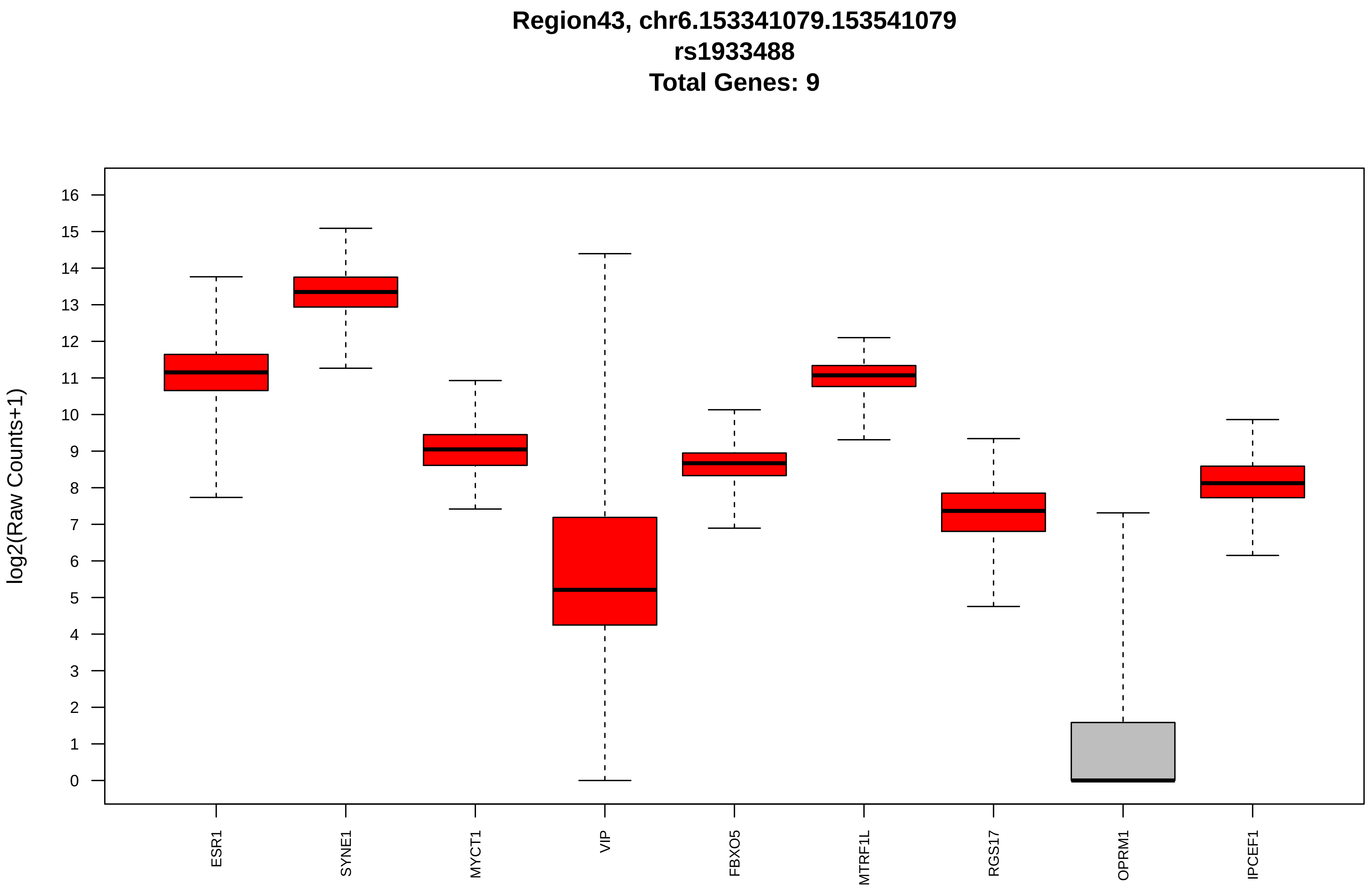

Region43, chr6.153341079.153541079
rs1933488
Total Genes: 9
log2(Raw Counts+1)
0
1
2
3
4
5
6
7
8
9
10
11
12
13
14
15
16
ESR1
SYNE1
MYCT1
VIP
FBXO5
MTRF1L
RGS17
OPRM1
IPCEF1

Region44, chr6.160481374.160681374
rs651164
Total Genes: 22

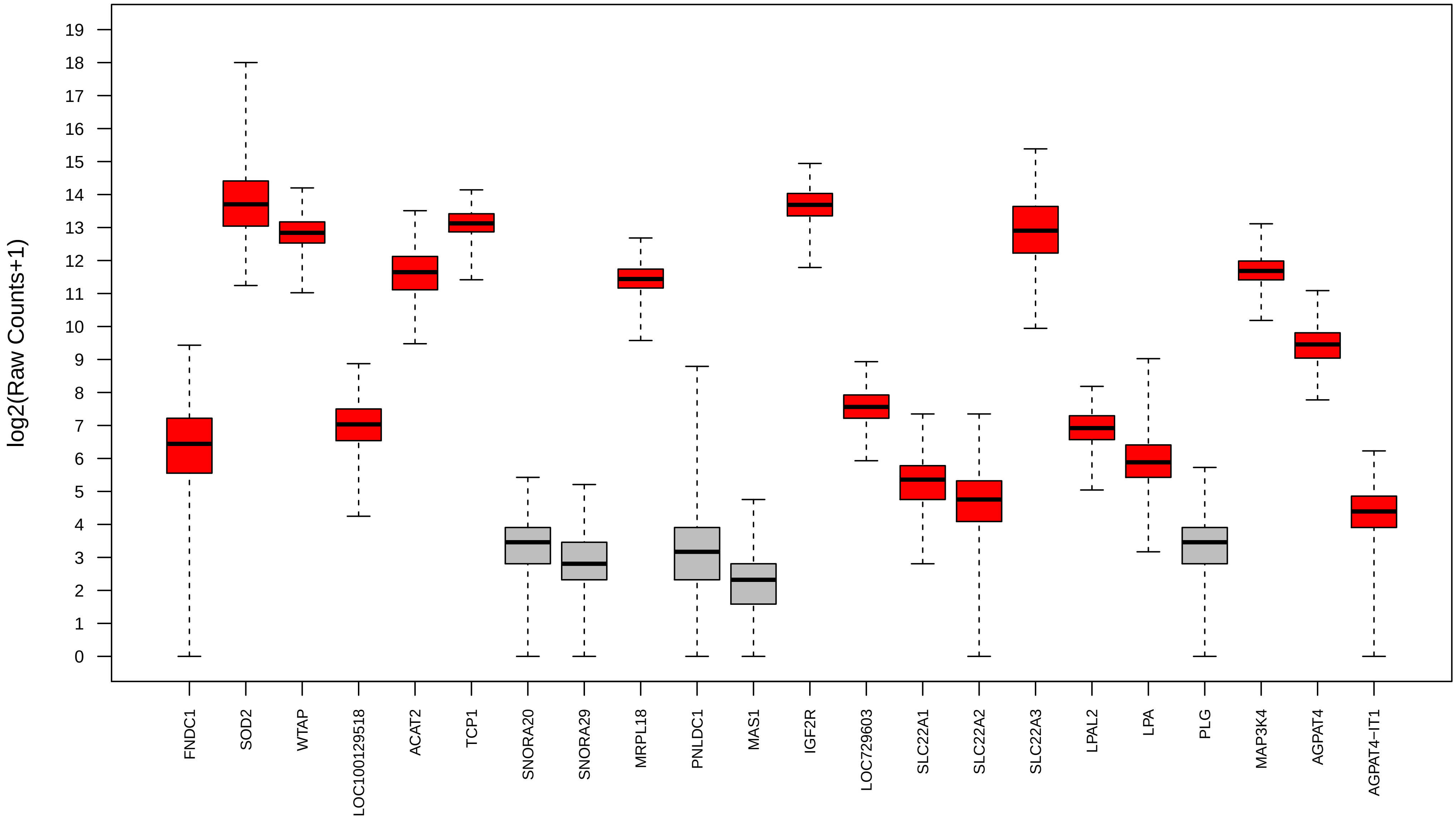

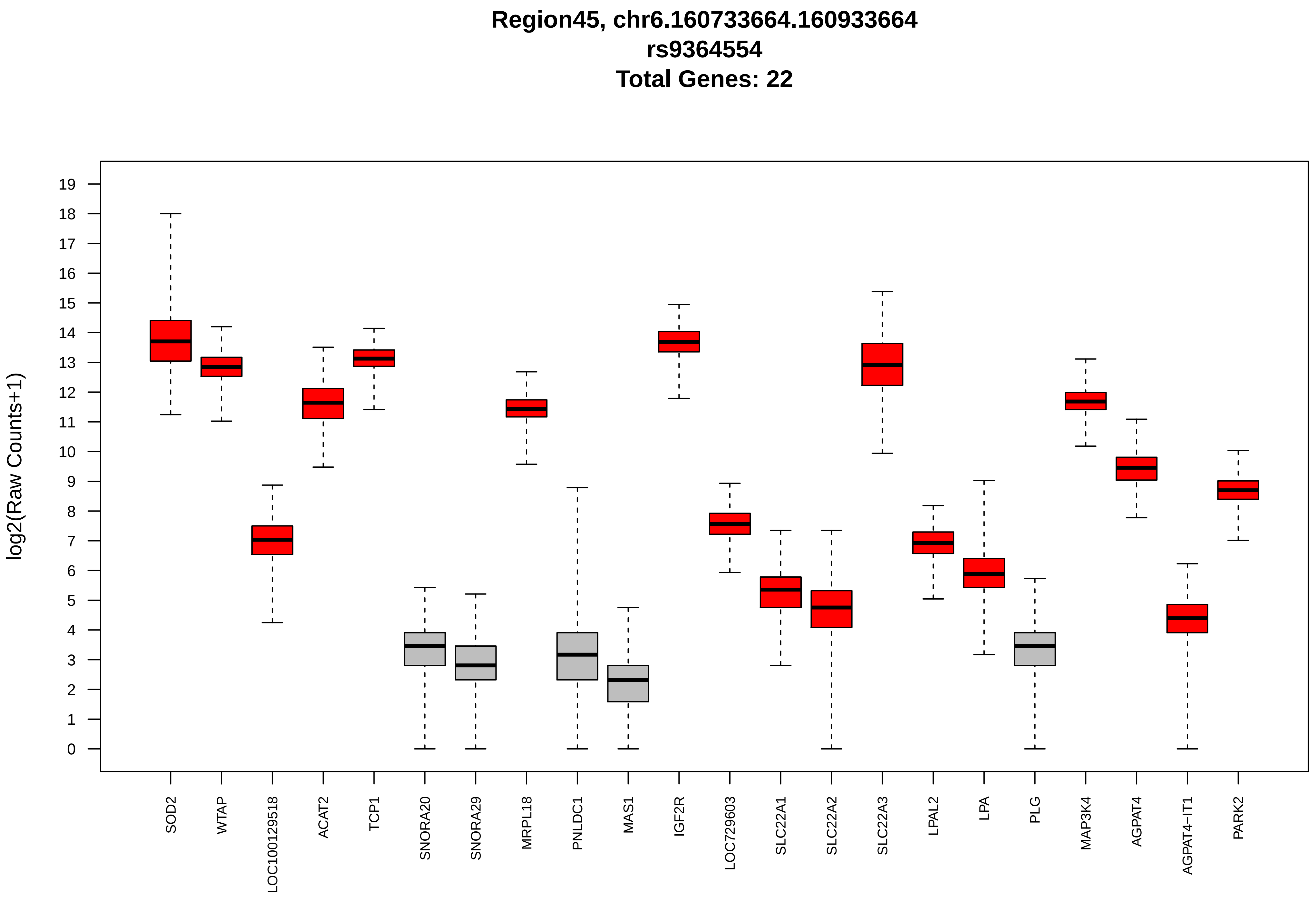
Region45, chr6.160733664.160933664
rs9364554
Total Genes: 22
log2(Raw Counts+1)
0
1
2
3
4
5
6
7
8
9
10
11
12
13
14
15
16
17
18
19
SOD2
WTAP
LOC100129518
ACAT2
TCP1
SNORA20
SNORA29
MRPL18
PNLDC1
MAS1
IGF2R
LOC729603
SLC22A1
SLC22A2
SLC22A3
LPAL2
LPA
PLG
MAP3K4
AGPAT4
AGPAT4−IT1
PARK2

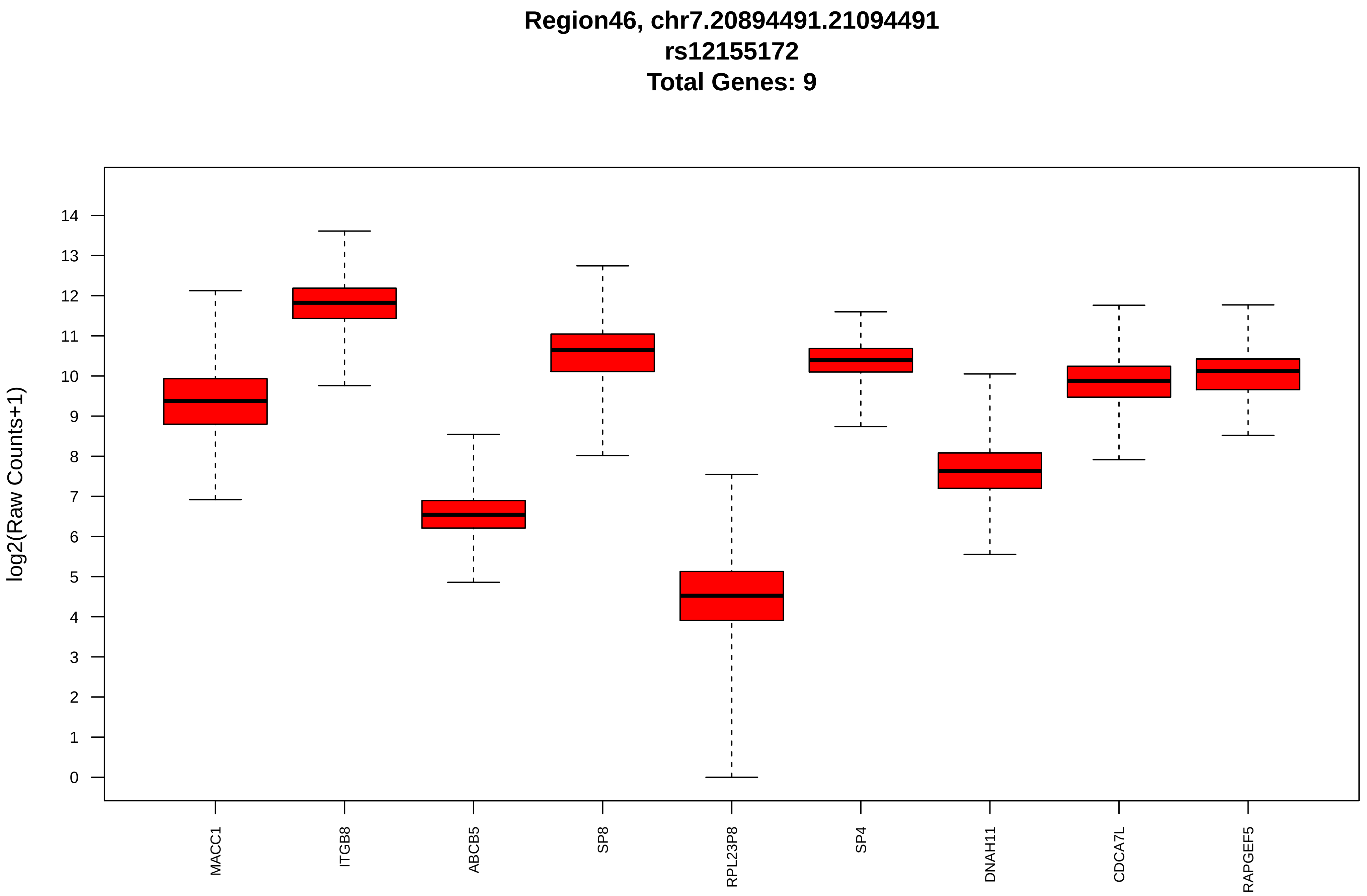

Region46, chr7.20894491.21094491
rs12155172
Total Genes: 9
log2(Raw Counts+1)
0
1
2
3
4
5
6
7
8
9
10
11
12
13
14
MACC1
ITGB8
ABCB5
SP8
RPL23P8
SP4
DNAH11
CDCA7L
RAPGEF5

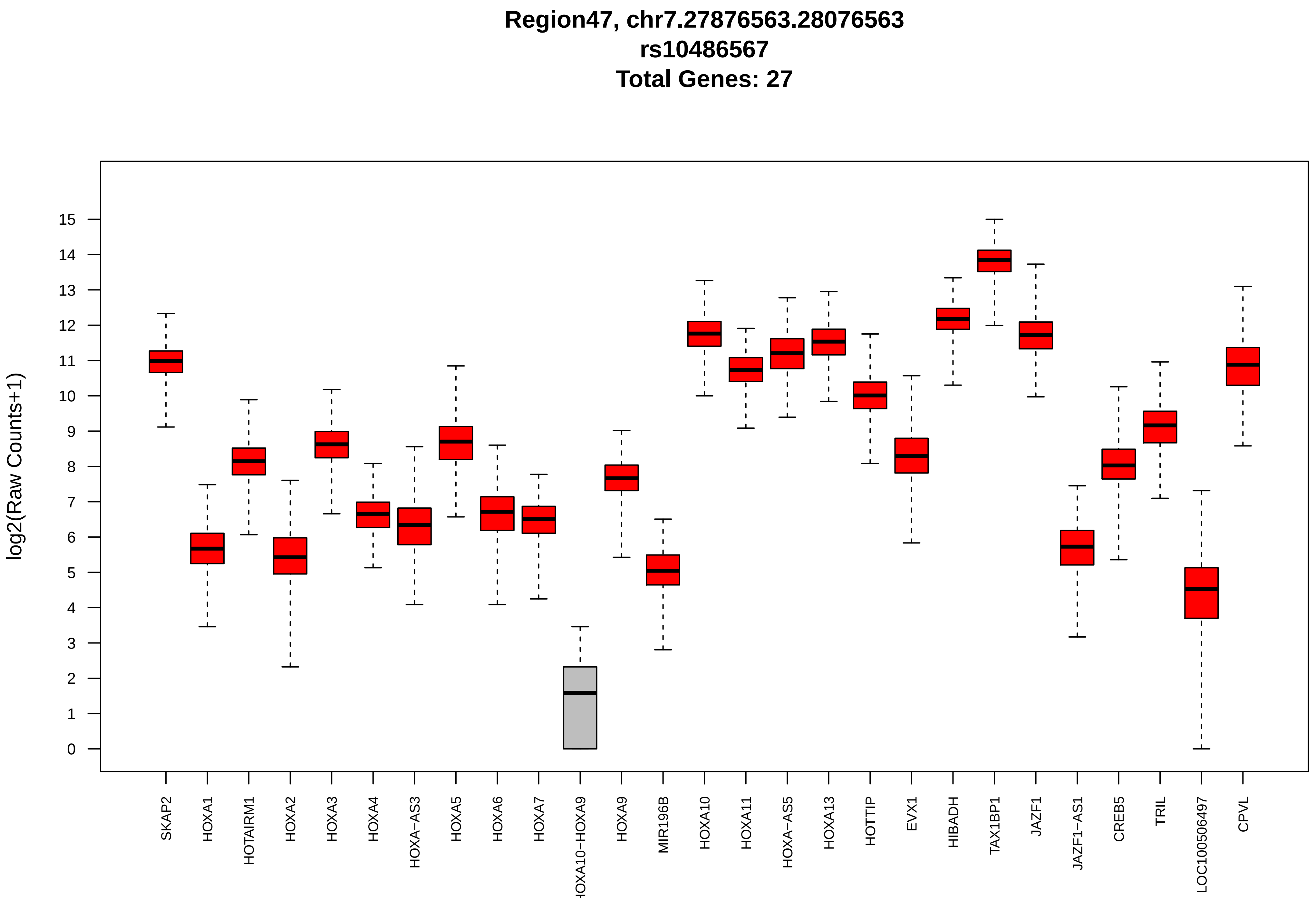

Region47, chr7.27876563.28076563
rs10486567
Total Genes: 27
log2(Raw Counts+1)
0
1
2
3
4
5
6
7
8
9
10
11
12
13
14
15
SKAP2
HOXA1
HOTAIRM1
HOXA2
HOXA3
HOXA4
HOXA−AS3
HOXA5
HOXA6
HOXA7
HOXA9
MIR196B
HOXA10
HOXA11
HOXA−AS5
HOXA13
HOTTIP
EVX1
HIBADH
TAX1BP1
JAZF1
JAZF1−AS1
CREB5
TRIL
LOC100506497
CPVL

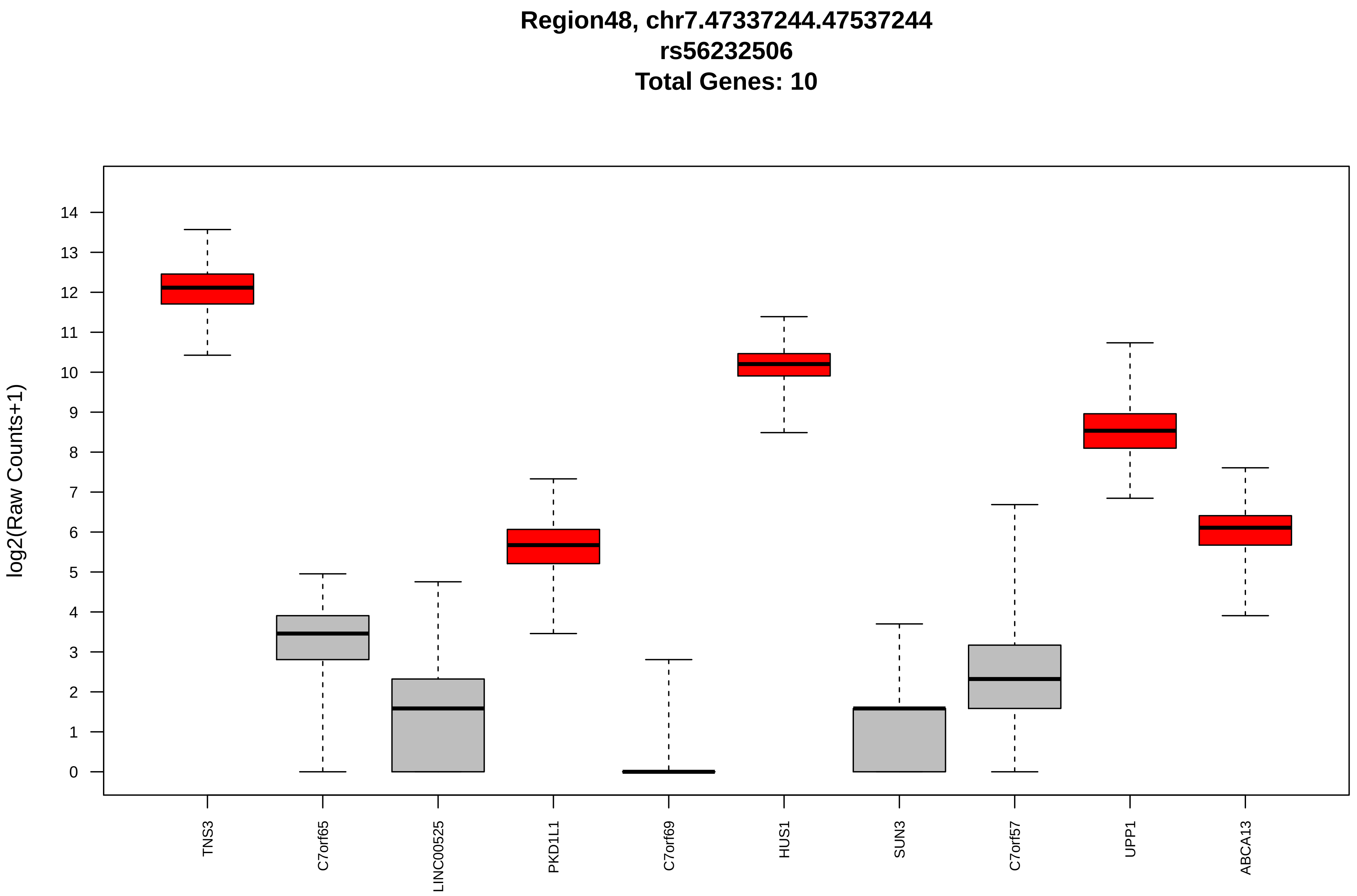

Region48, chr7.47337244.47537244
rs56232506
Total Genes: 10
log2(Raw Counts+1)
0
1
2
3
4
5
6
7
8
9
10
11
12
13
14
TNS3
C7orf65
LINC00525
PKD1L1
C7orf69
HUS1
SUN3
C7orf57
UPP1
ABCA13

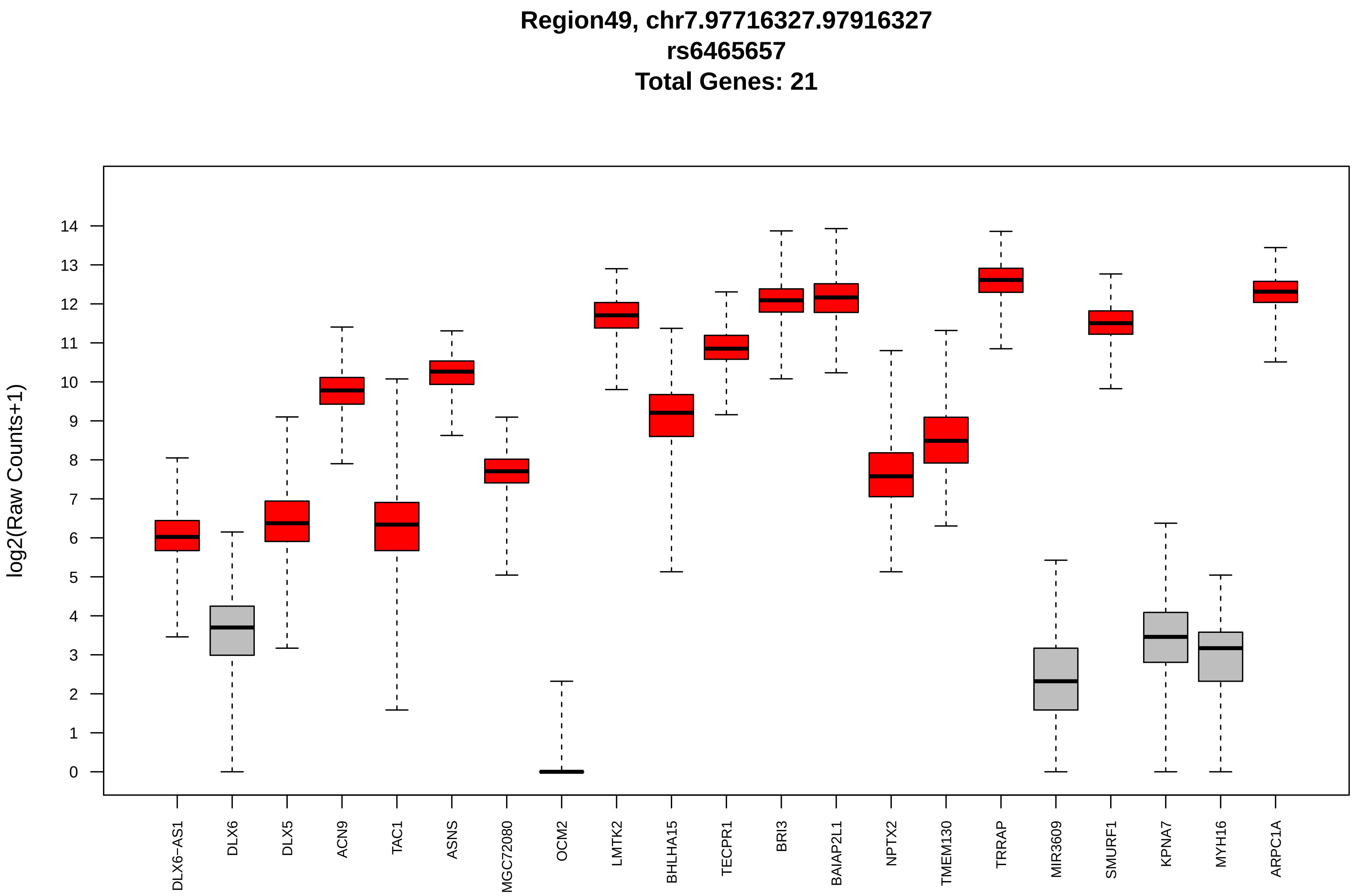

Region49, chr7.97716327.97916327
rs6465657
Total Genes: 21
log2(Raw Counts+1)
0
1
2
3
4
5
6
7
8
9
10
11
12
13
14
DLX6−AS1
DLX6
DLX5
ACN9
TAC1
ASNS
MGC72080
OCM2
LMTK2
BHLHA15
TECPR1
BRI3
BAIAP2L1
NPTX2
TMEM130
TRRAP
MIR3609
SMURF1
KPNA7
MYH16
ARPC1A

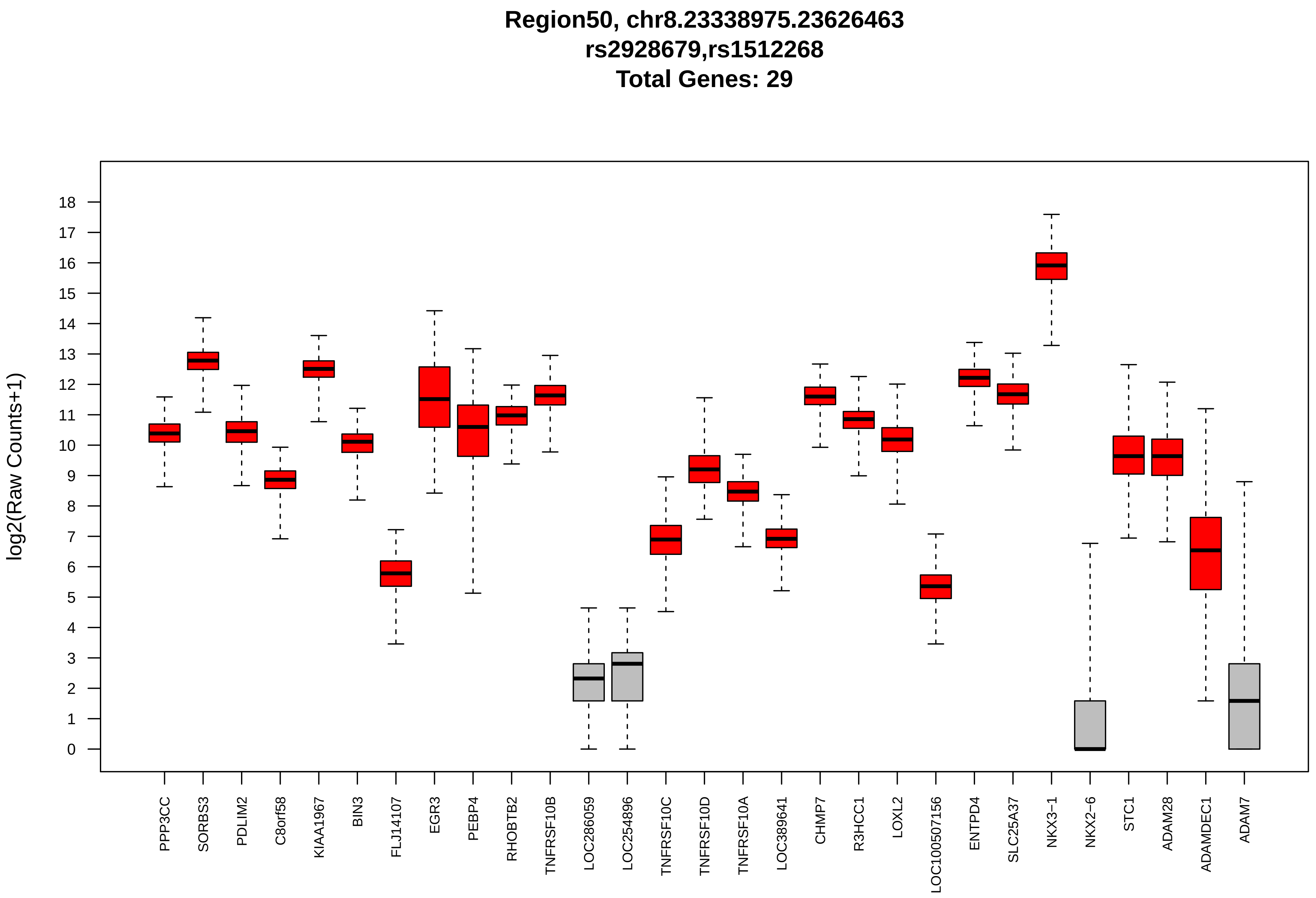
Region50, chr8.23338975.23626463
rs2928679,rs1512268
Total Genes: 29
log2(Raw Counts+1)
0
1
2
3
4
5
6
7
8
9
10
11
12
13
14
15
16
17
18
PPP3CC
SORBS3
PDLIM2
C8orf58
KIAA1967
BIN3
FLJ14107
EGR3
PEBP4
RHOBTB2
TNFRSF10B
LOC286059
LOC254896
TNFRSF10C
TNFRSF10D
TNFRSF10A
LOC389641
CHMP7
R3HCC1
LOXL2
LOC100507156
ENTPD4
SLC25A37
NKX3−1
NKX2−6
STC1
ADAM28
ADAMDEC1
ADAM7

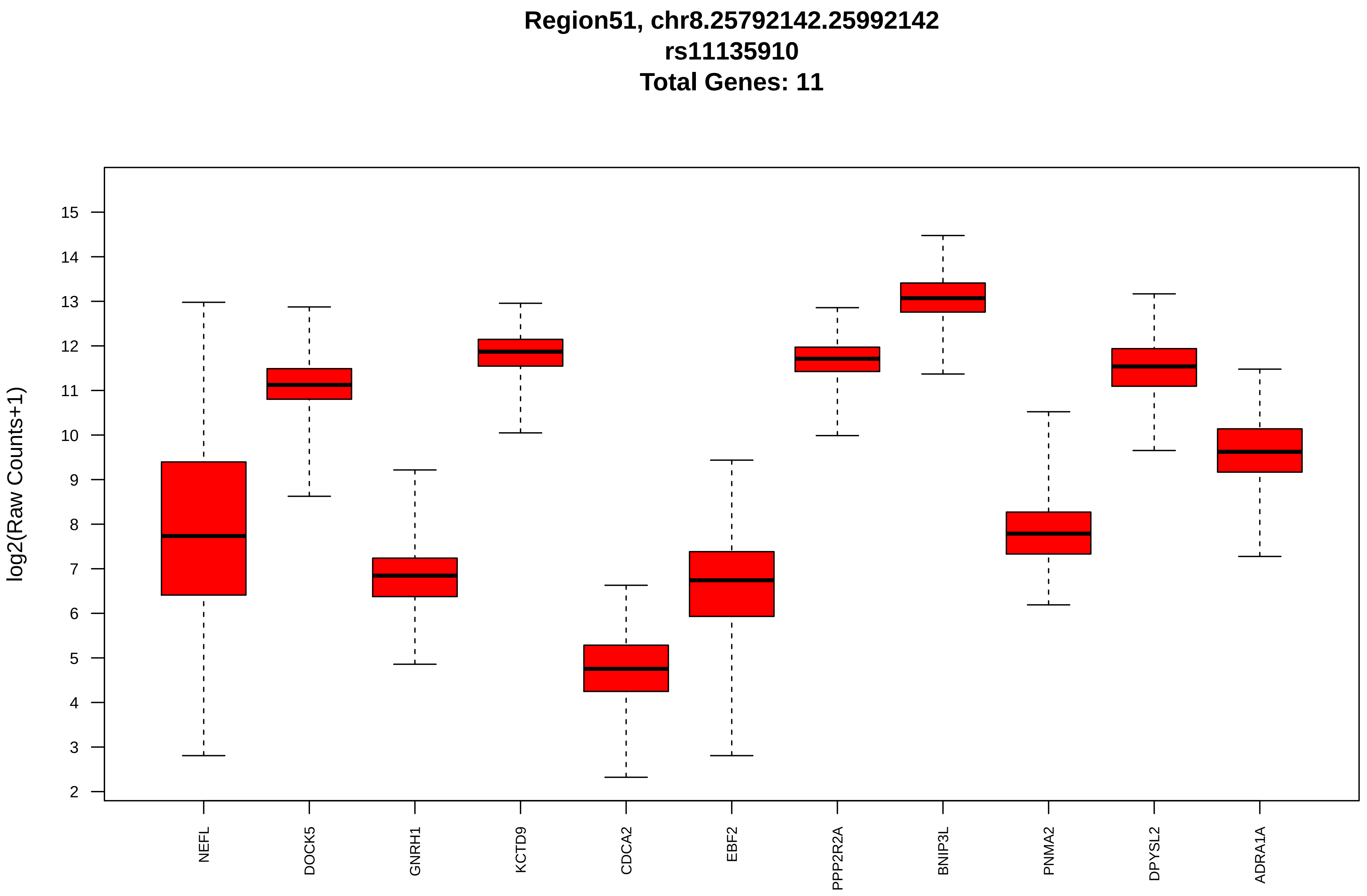

Region51, chr8.25792142.25992142
rs11135910
Total Genes: 11
log2(Raw Counts+1)
15
14
13
12
11
10
9
8
7
6
5
4
3
2
NEFL
DOCK5
GNRH1
KCTD9
CDCA2
EBF2
PPP2R2A
BNIP3L
PNMA2
DPYSL2
ADRA1A

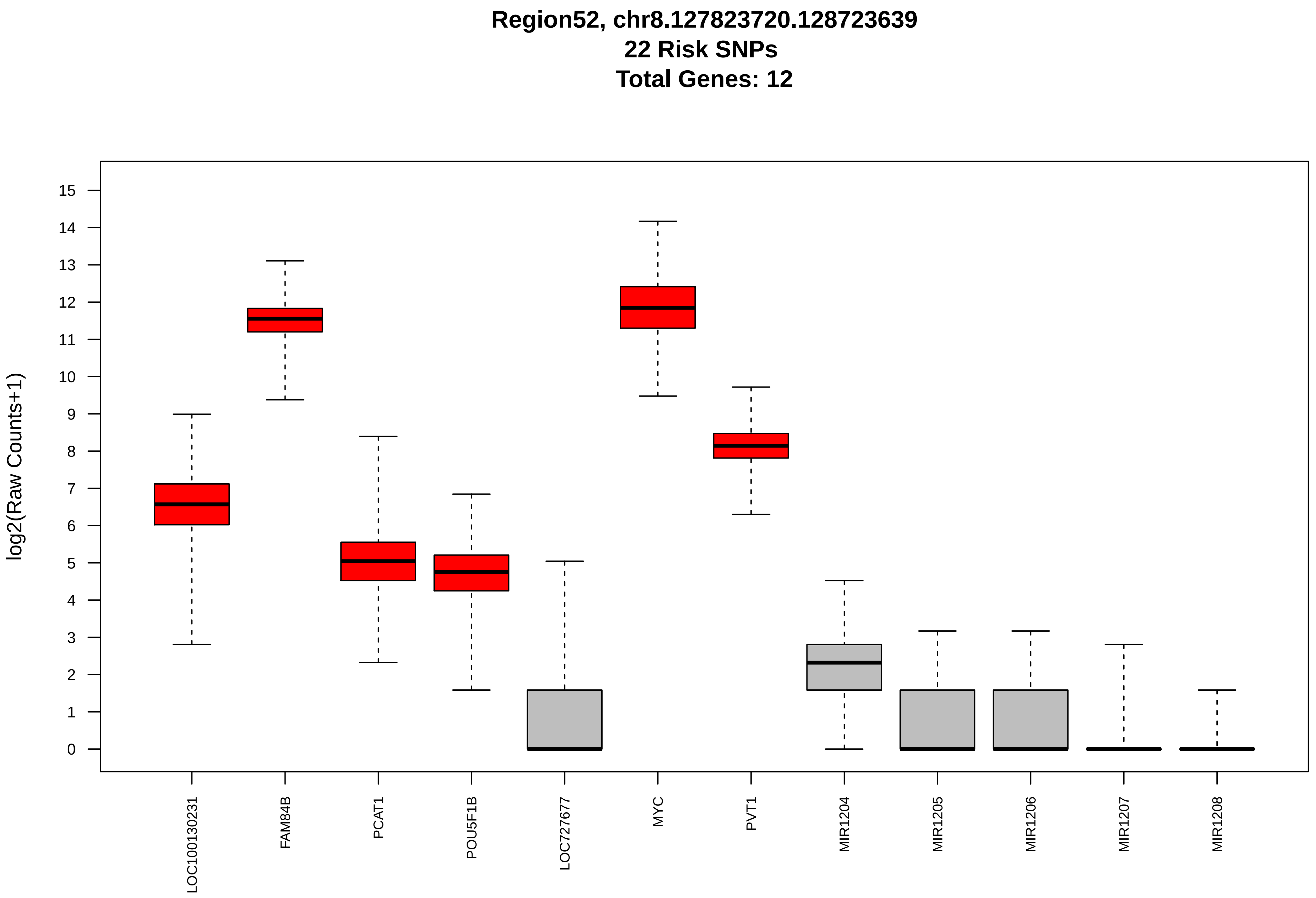

Region52, chr8.127823720.128723639
22 Risk SNPs
Total Genes: 12
log2(Raw Counts+1)
0
1
2
3
4
5
6
7
8
9
10
11
12
13
14
15
LOC100130231
FAM84B
PCAT1
POU5F1B
LOC727677
MYC
PVT1
MIR1204
MIR1205
MIR1206
MIR1207
MIR1208

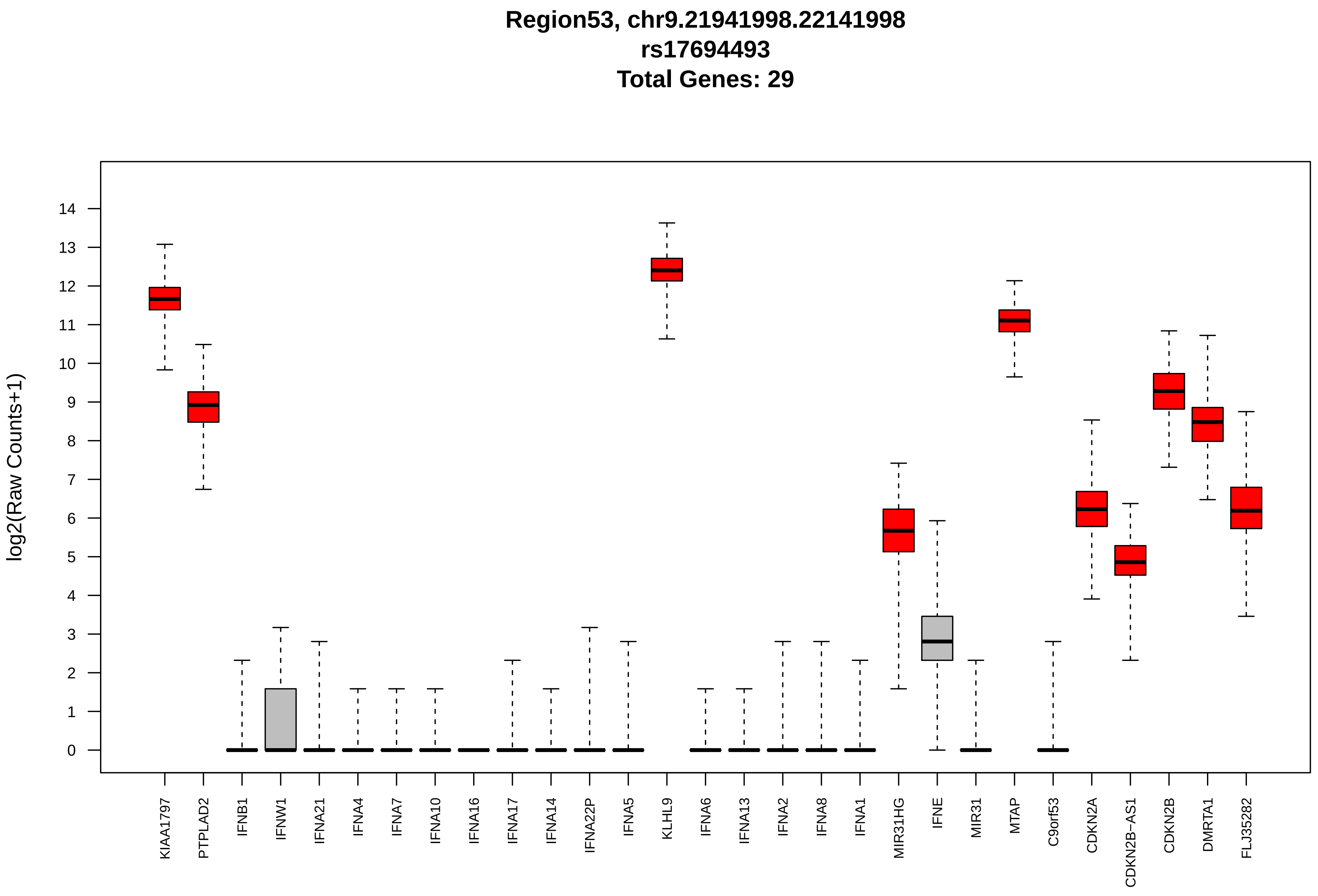
Region53, chr9.21941998.22141998
rs17694493
Total Genes: 29
log2(Raw Counts+1)
0
1
2
3
4
5
6
7
8
9
10
11
12
13
14
KIAA1797
PTPLAD2
IFNB1
IFNW1
IFNA21
IFNA4
IFNA7
IFNA10
IFNA16
IFNA17
IFNA14
IFNA22P
IFNA5
KLHL9
IFNA6
IFNA13
IFNA2
IFNA8
IFNA1
MIR31HG
IFNE
MIR31
MTAP
C9orf53
CDKN2A
CDKN2B−AS1
CDKN2B
DMRTA1
FLJ35282

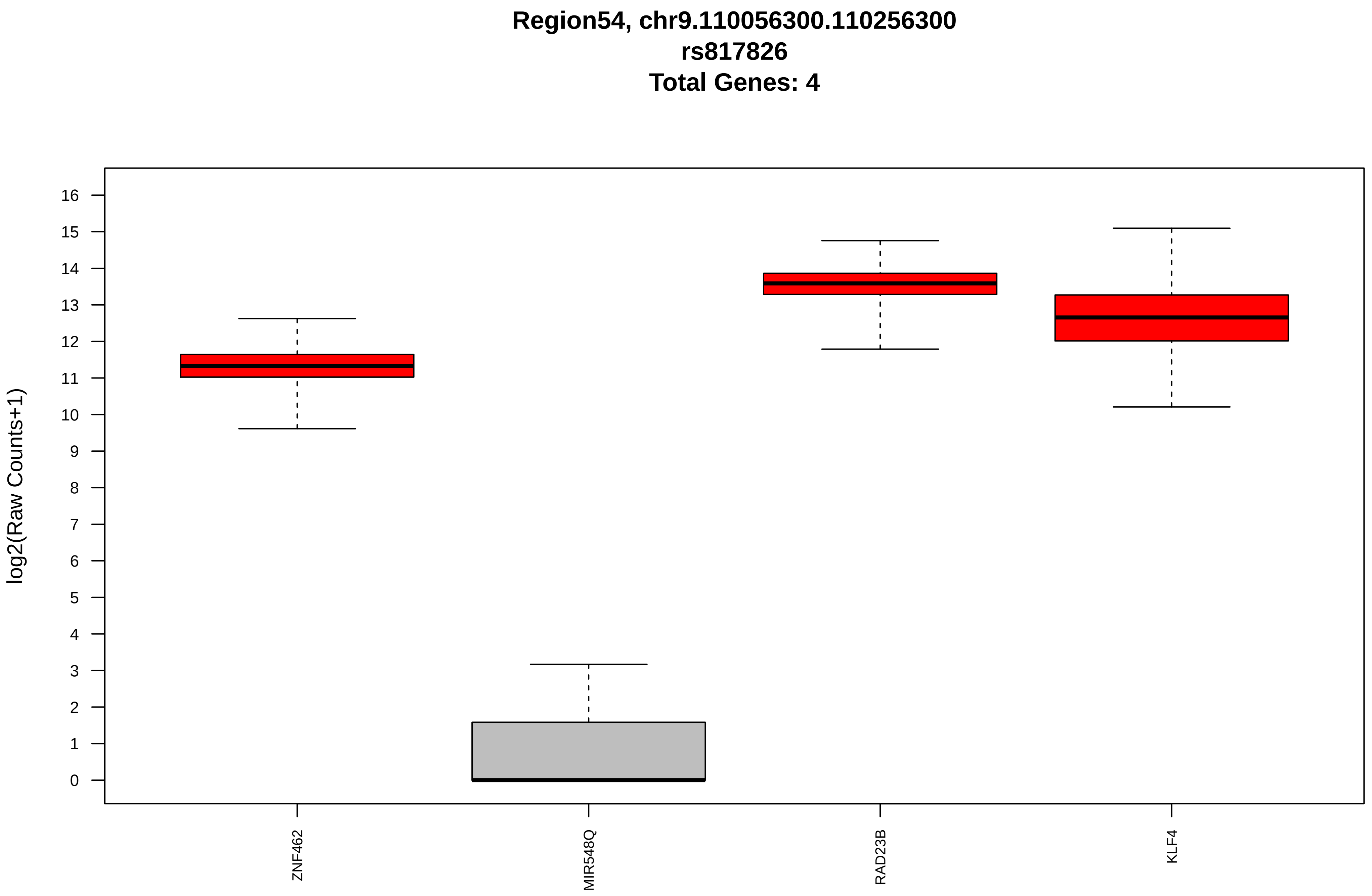

Region54, chr9.110056300.110256300
rs817826
Total Genes: 4
log2(Raw Counts+1)
0
1
2
3
4
5
6
7
8
9
10
11
12
13
14
15
16
ZNF462
MIR548Q
RAD23B
KLF4

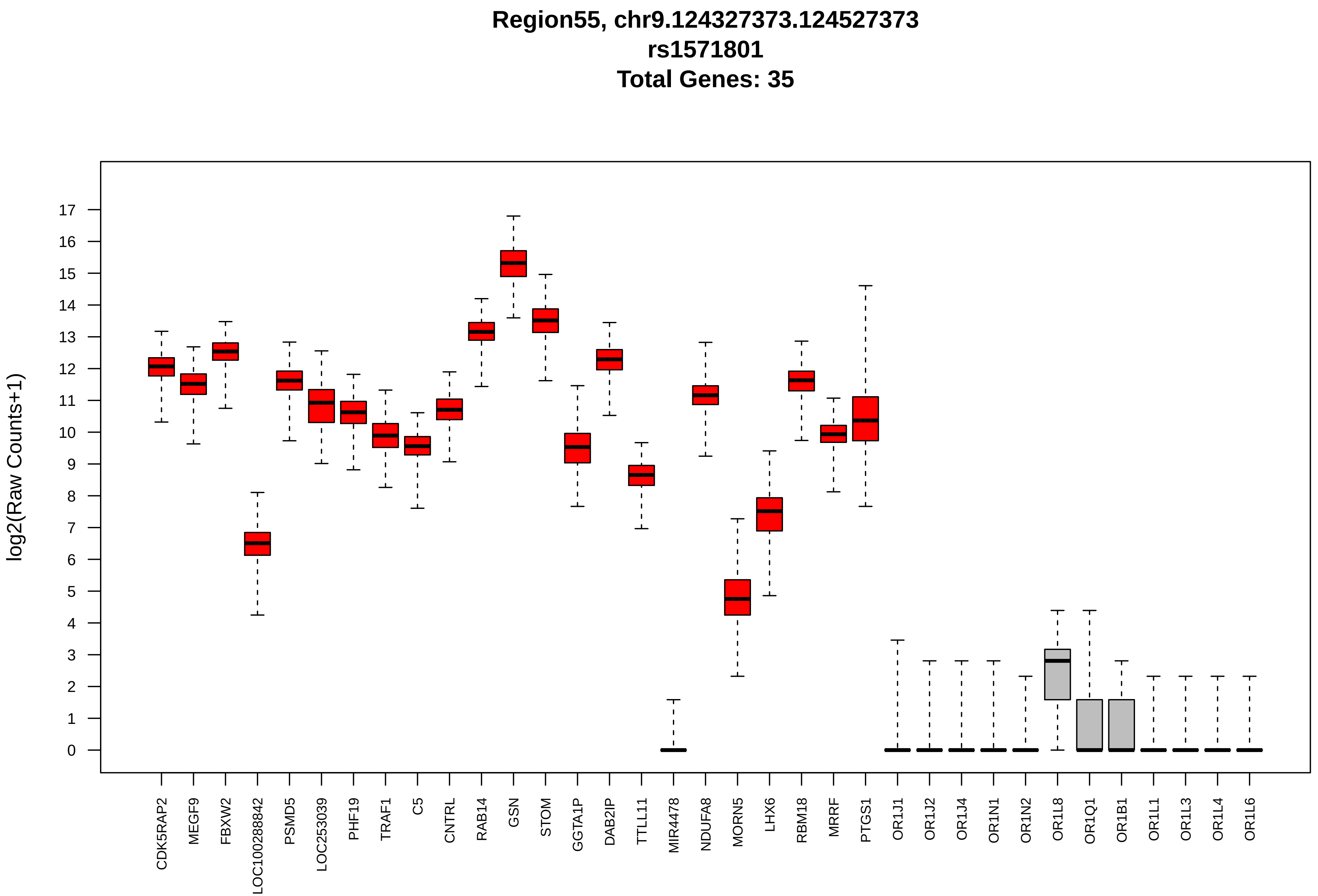
Region55, chr9.124327373.124527373
rs1571801
Total Genes: 35
log2(Raw Counts+1)
0
1
2
3
4
5
6
7
8
9
10
11
12
13
14
15
16
17
CDK5RAP2
MEGF9
FBXW2
LOC100288842
PSMD5
LOC253039
PHF19
TRAF1
C5
CNTRL
RAB14
GSN
STOM
GGTA1P
DAB2IP
TTLL11
MIR4478
NDUFA8
MORN5
LHX6
RBM18
MRRF
PTGS1
OR1J1
OR1J2
OR1J4
OR1N1
OR1N2
OR1L8
OR1Q1
OR1B1
OR1L1
OR1L3
OR1L4
OR1L6

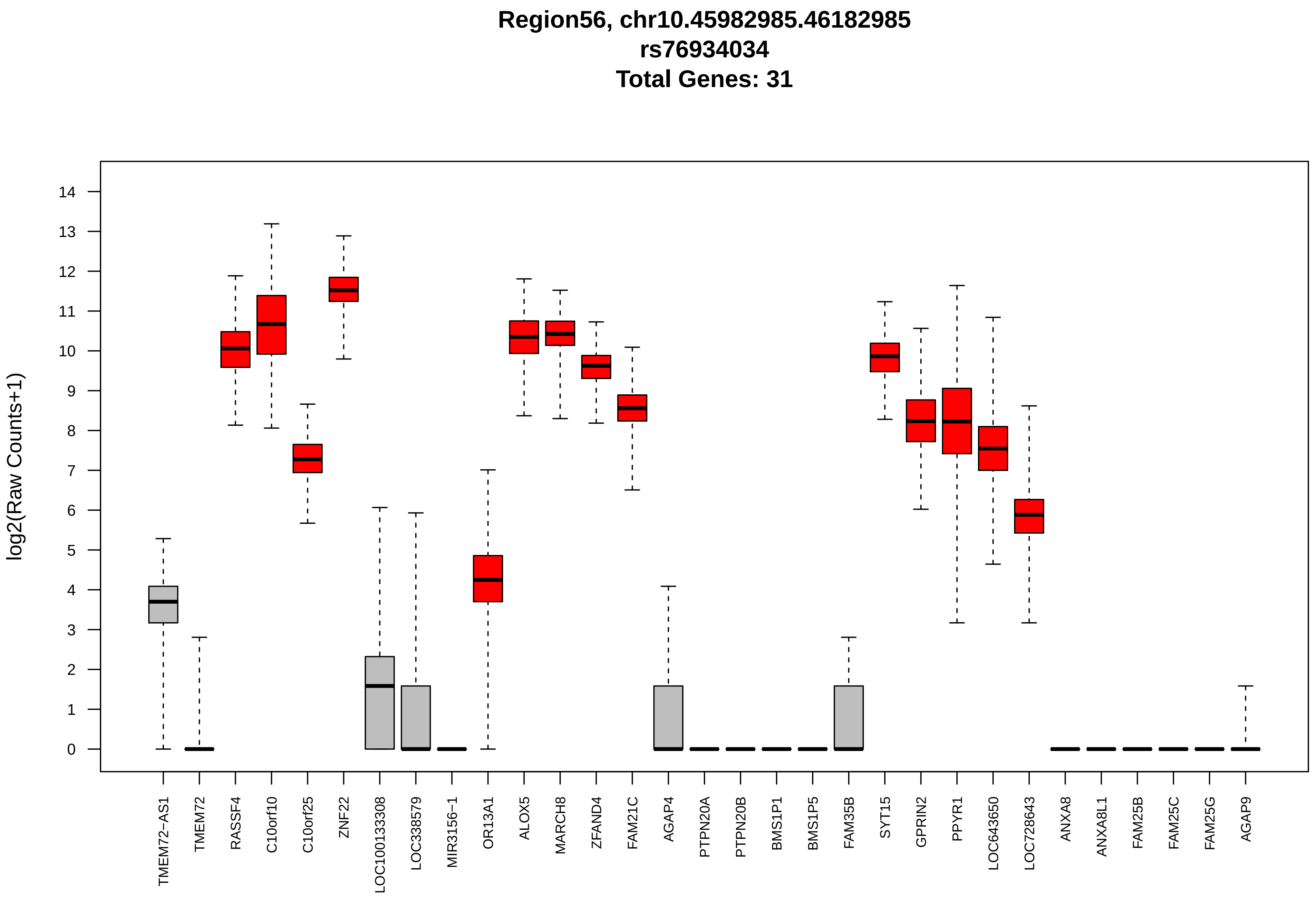

Region56, chr10.45982985.46182985
rs76934034
Total Genes: 31
log2(Raw Counts+1)
0
1
2
3
4
5
6
7
8
9
10
11
12
13
14
TMEM72−AS1
TMEM72
RASSF4
C10orf10
C10orf25
ZNF22
LOC100133308
LOC338579
MIR3156−1
OR13A1
ALOX5
MARCH8
ZFAND4
FAM21C
AGAP4
PTPN20A
PTPN20B
BMS1P1
BMS1P5
FAM35B
SYT15
GPRIN2
PPYR1
LOC643650
LOC728643
ANXA8
ANXA8L1
FAM25B
FAM25C
FAM25G
AGAP9

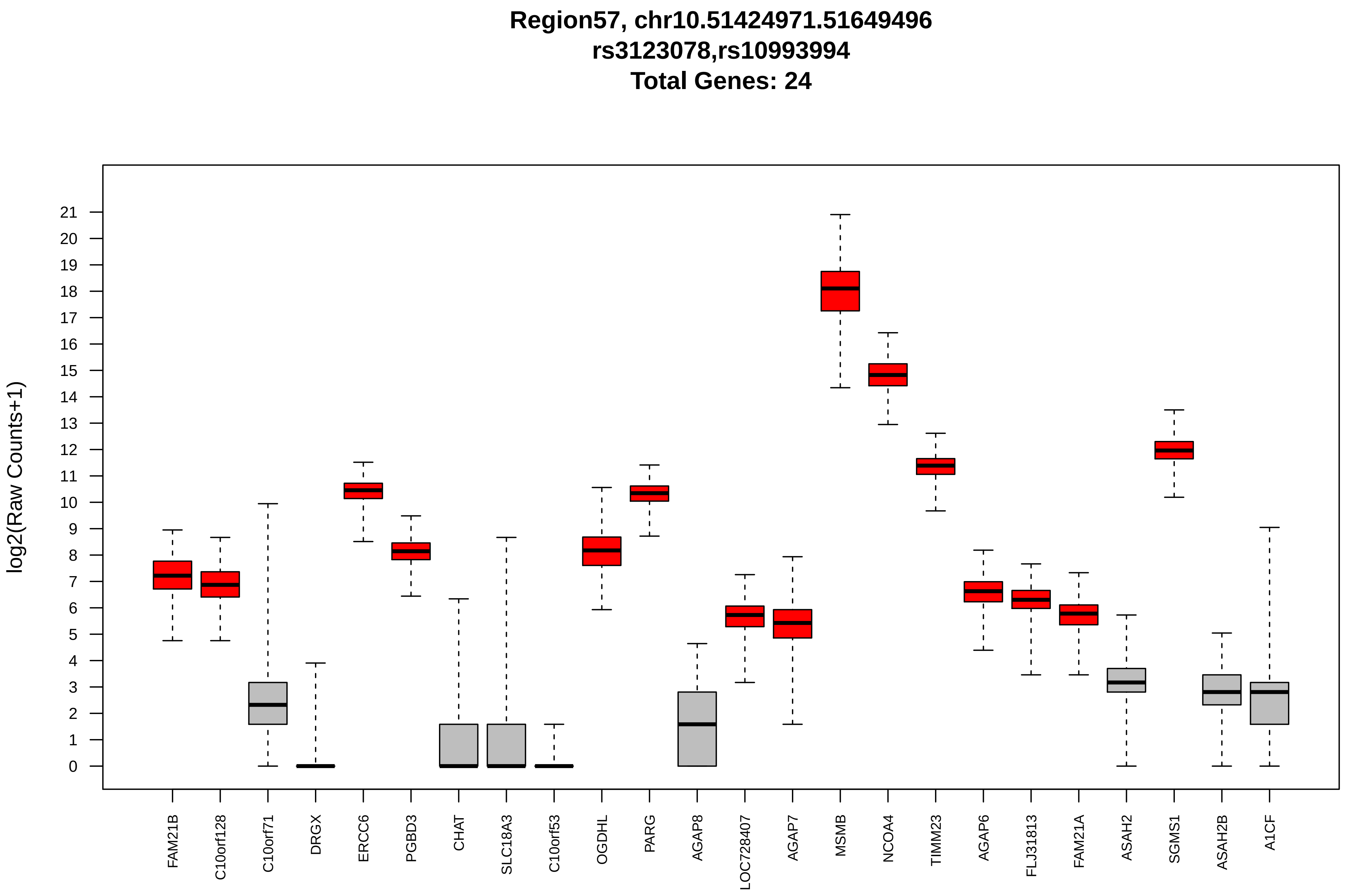

Region57, chr10.51424971.51649496
rs3123078,rs10993994
Total Genes: 24
log2(Raw Counts+1)
0
1
2
3
4
5
6
7
8
9
10
11
12
13
14
15
16
17
18
19
20
21
FAM21B
C10orf128
C10orf71
DRGX
ERCC6
PGBD3
CHAT
SLC18A3
C10orf53
OGDHL
PARG
AGAP8
LOC728407
AGAP7
MSMB
NCOA4
TIMM23
AGAP6
FLJ31813
FAM21A
ASAH2
SGMS1
ASAH2B
A1CF

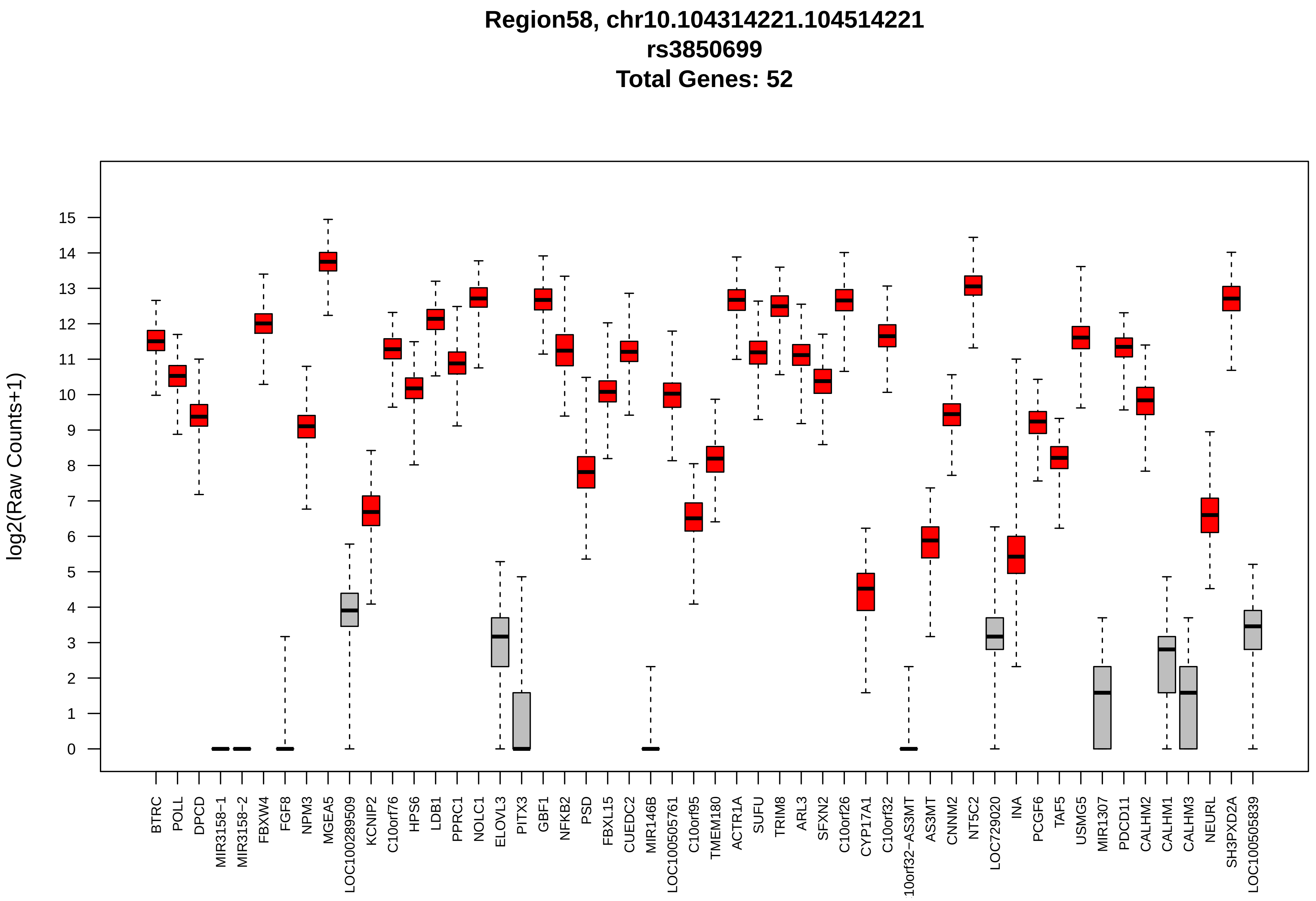
Region58, chr10.104314221.104514221
rs3850699
Total Genes: 52
log2(Raw Counts+1)
0
1
2
3
4
5
6
7
8
9
10
11
12
13
14
15
BTRC
POLL
DPCD
MIR3158−1
MIR3158−2
FBXW4
FGF8
NPM3
MGEA5
LOC100289509
KCNIP2
C10orf76
HPS6
LDB1
PPRC1
NOLC1
ELOVL3
PITX3
GBF1
NFKB2
PSD
FBXL15
CUEDC2
MIR146B
LOC100505761
C10orf95
TMEM180
ACTR1A
SUFU
TRIM8
ARL3
SFXN2
C10orf26
CYP17A1
C10orf32
10orf32−AS3MT
AS3MT
CNNM2
NT5C2
LOC729020
INA
PCGF6
TAF5
USMG5
MIR1307
PDCD11
CALHM2
CALHM1
CALHM3
NEURL
SH3PXD2A
LOC100505839

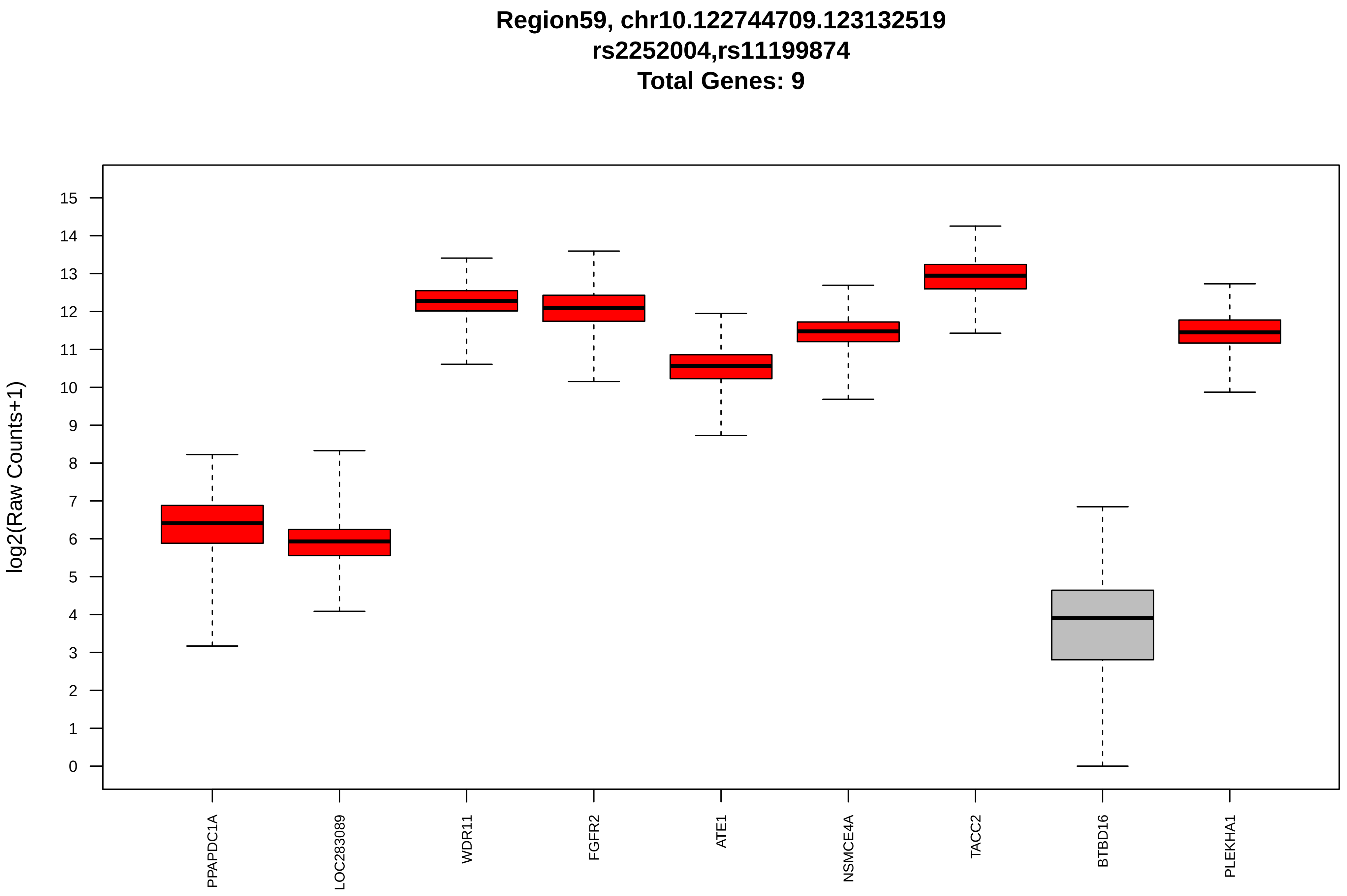

Region59, chr10.122744709.123132519
rs2252004,rs11199874
Total Genes: 9
log2(Raw Counts+1)
0
1
2
3
4
5
6
7
8
9
10
11
12
13
14
15
PPAPDC1A
LOC283089
WDR11
FGFR2
ATE1
NSMCE4A
TACC2
BTBD16
PLEKHA1

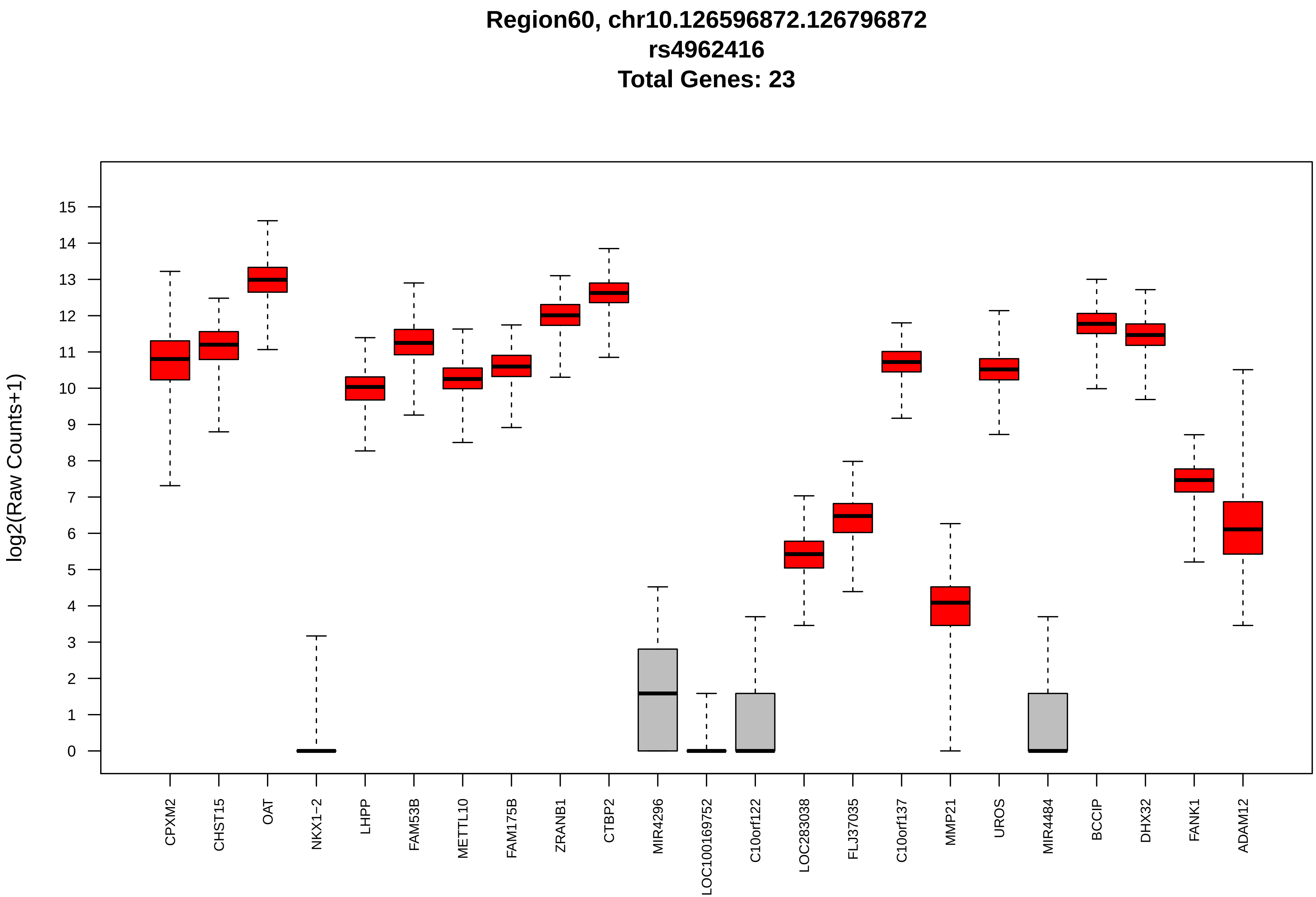
Region60, chr10.126596872.126796872
rs4962416
Total Genes: 23
log2(Raw Counts+1)
0
1
2
3
4
5
6
7
8
9
10
11
12
13
14
15
CPXM2
CHST15
OAT
NKX1−2
LHPP
FAM53B
METTL10
FAM175B
ZRANB1
CTBP2
MIR4296
LOC100169752
C10orf122
LOC283038
FLJ37035
C10orf137
MMP21
UROS
MIR4484
BCCIP
DHX32
FANK1
ADAM12

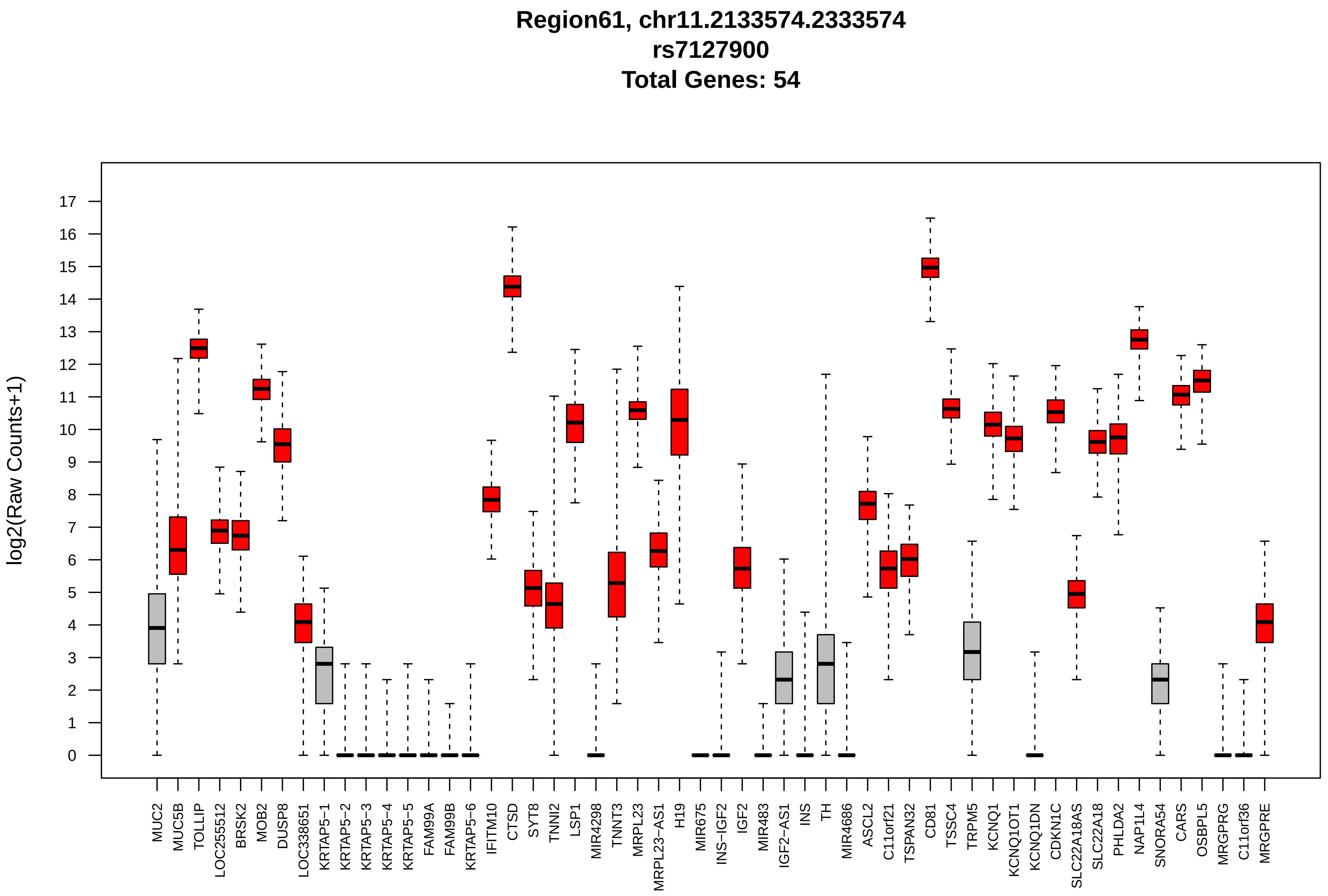

Region61, chr11.2133574.2333574
rs7127900
Total Genes: 54
log2(Raw Counts+1)
0
1
2
3
4
5
6
7
8
9
10
11
12
13
14
15
16
17
MUC2
MUC5B
TOLLIP
LOC255512
BRSK2
MOB2
DUSP8
LOC338651
KRTAP5−1
KRTAP5−2
KRTAP5−3
KRTAP5−4
KRTAP5−5
FAM99A
FAM99B
KRTAP5−6
IFITM10
CTSD
SYT8
TNNI2
LSP1
MIR4298
TNNT3
MRPL23
MRPL23−AS1
H19
MIR675
INS−IGF2
IGF2
MIR483
IGF2−AS1
INS
TH
MIR4686
ASCL2
C11orf21
TSPAN32
CD81
TSSC4
TRPM5
KCNQ1
KCNQ1OT1
KCNQ1DN
CDKN1C
SLC22A18AS
SLC22A18
PHLDA2
NAP1L4
SNORA54
CARS
OSBPL5
MRGPRG
C11orf36
MRGPRE

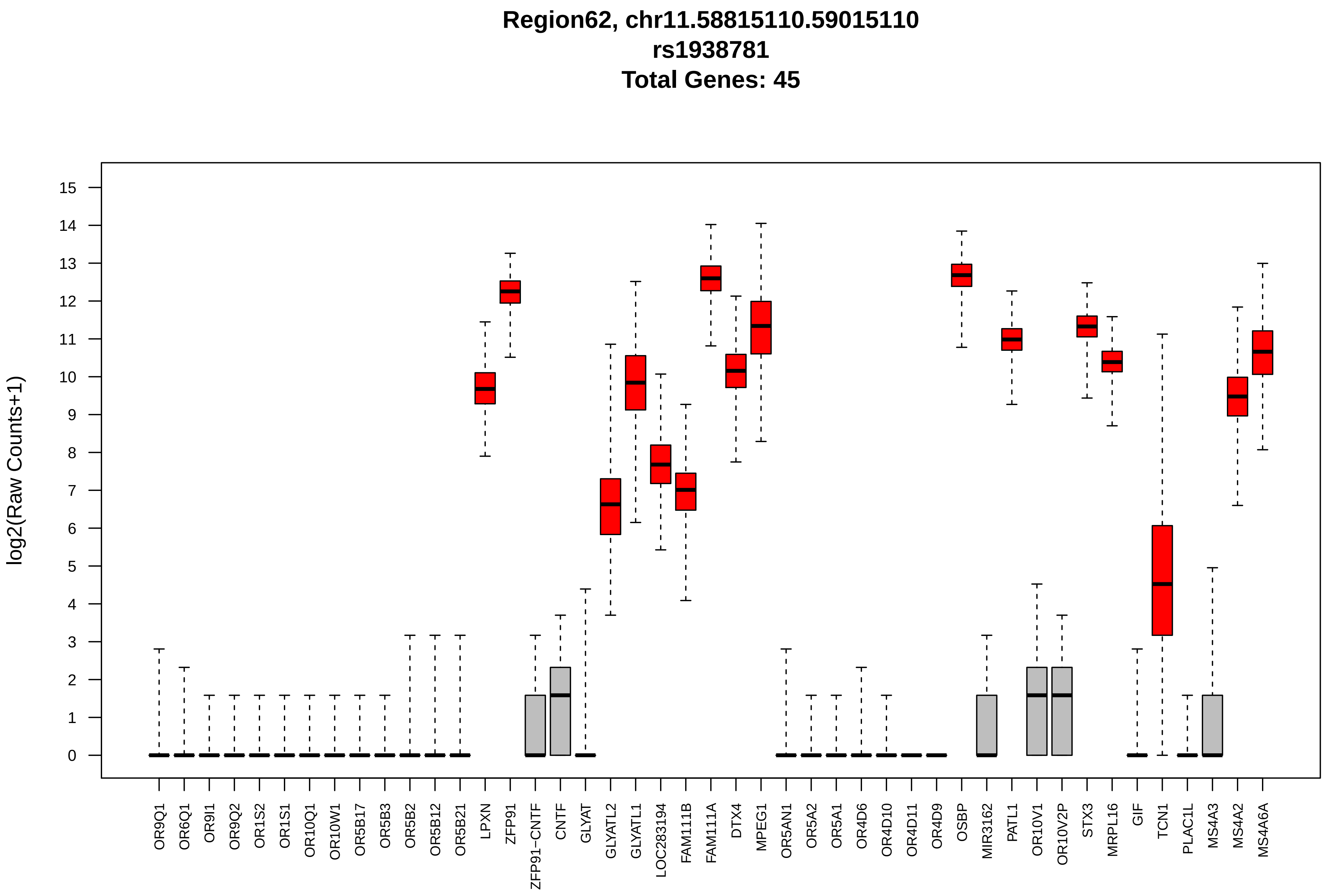

Region62, chr11.58815110.59015110
rs1938781
Total Genes: 45
log2(Raw Counts+1)
0
1
2
3
4
5
6
7
8
9
10
11
12
13
14
15
OR9Q1
OR6Q1
OR9I1
OR9Q2
OR1S2
OR1S1
OR10Q1
OR10W1
OR5B17
OR5B3
OR5B2
OR5B12
OR5B21
LPXN
ZFP91
ZFP91−CNTF
CNTF
GLYAT
GLYATL2
GLYATL1
LOC283194
FAM111B
FAM111A
DTX4
MPEG1
OR5AN1
OR5A2
OR5A1
OR4D6
OR4D10
OR4D11
OR4D9
OSBP
MIR3162
PATL1
OR10V1
OR10V2P
STX3
MRPL16
GIF
TCN1
PLAC1L
MS4A3
MS4A2
MS4A6A

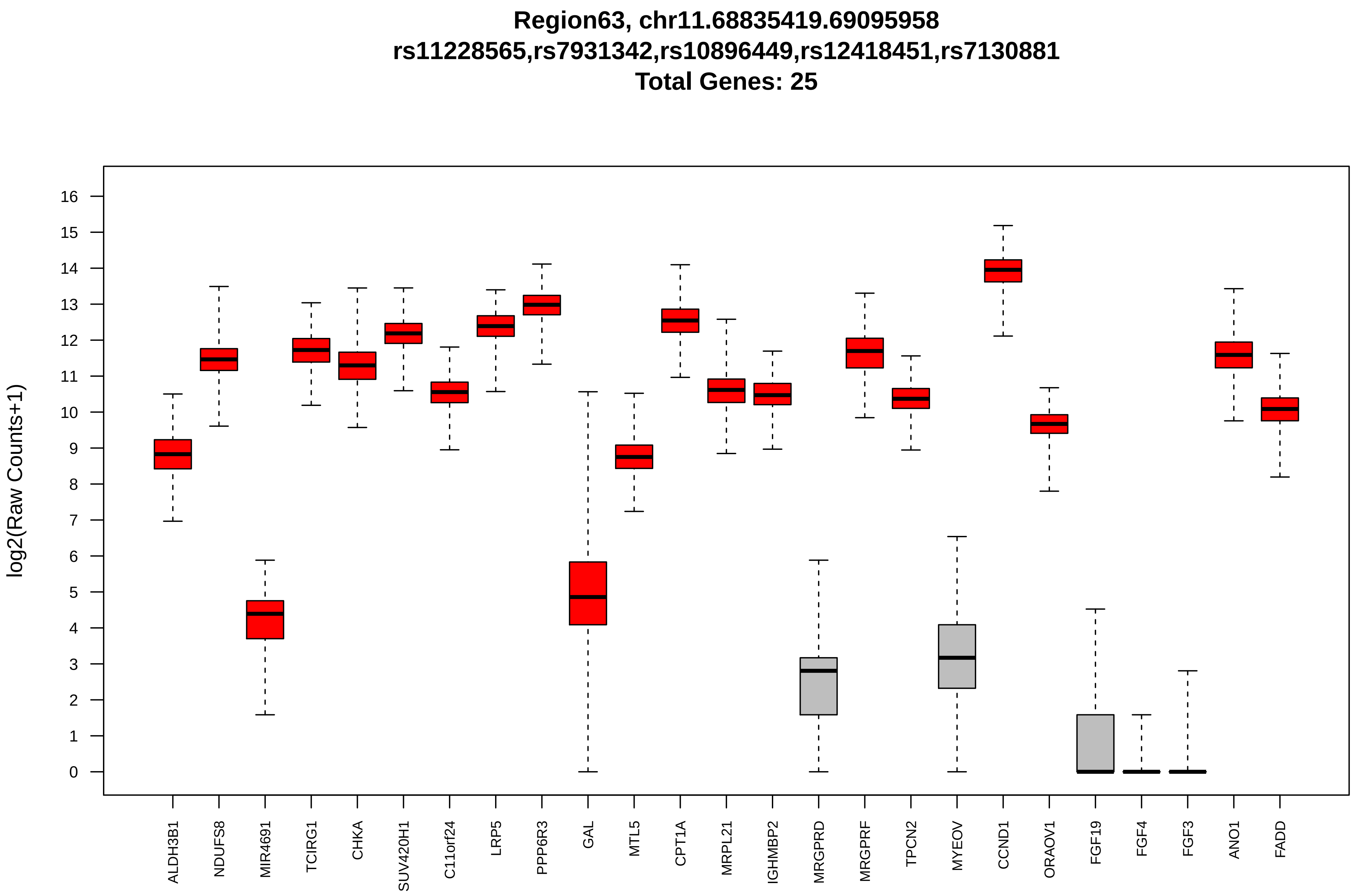

Region63, chr11.68835419.69095958
rs11228565,rs7931342,rs10896449,rs12418451,rs7130881
Total Genes: 25
log2(Raw Counts+1)
0
1
2
3
4
5
6
7
8
9
10
11
12
13
14
15
16
ALDH3B1
NDUFS8
MIR4691
TCIRG1
CHKA
SUV420H1
C11orf24
LRP5
PPP6R3
GAL
MTL5
CPT1A
MRPL21
IGHMBP2
MRGPRD
MRGPRF
TPCN2
MYEOV
CCND1
ORAOV1
FGF19
FGF4
FGF3
ANO1
FADD

Region64, chr11.102301661.102501661
rs11568818
Total Genes: 21

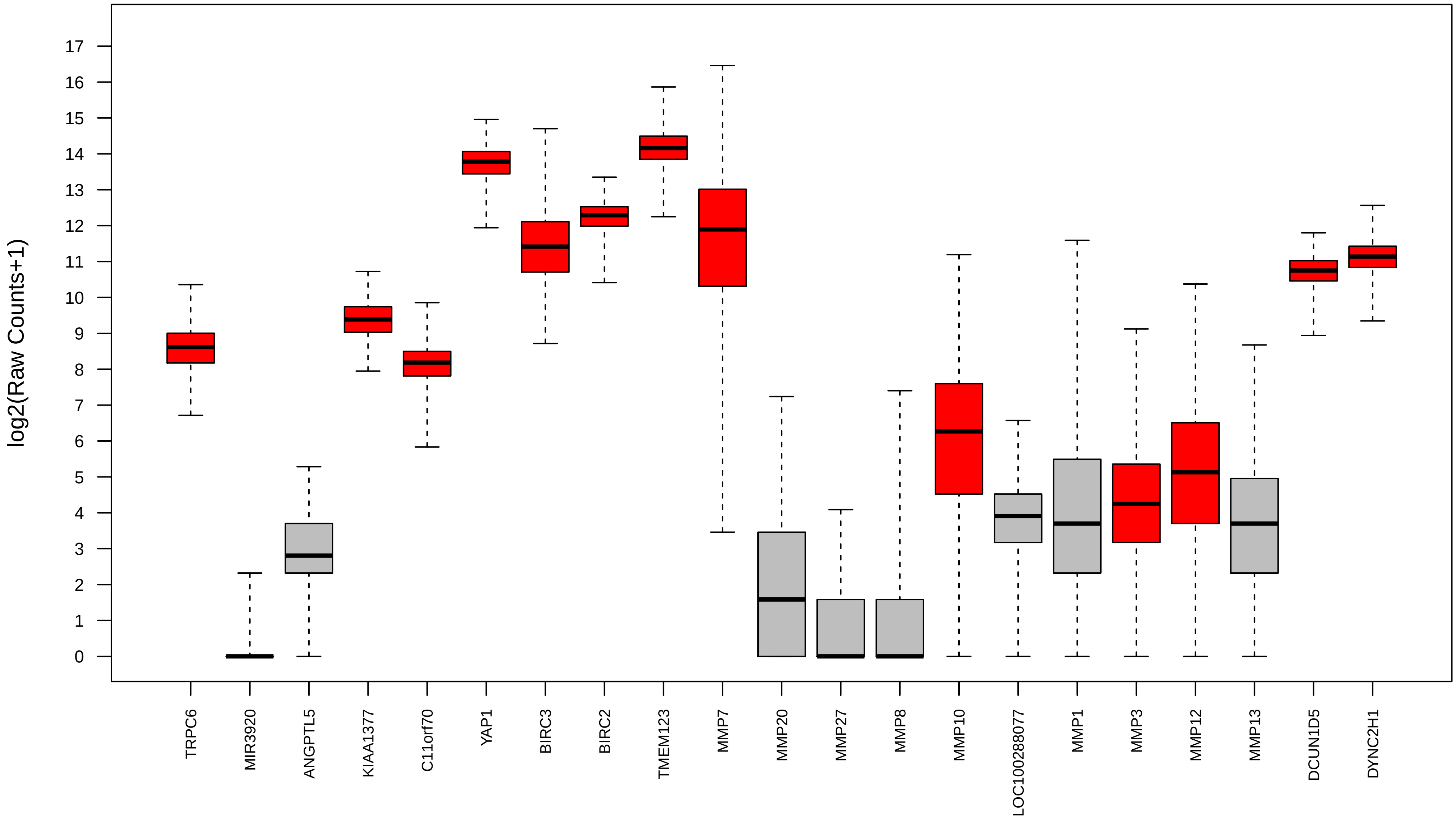

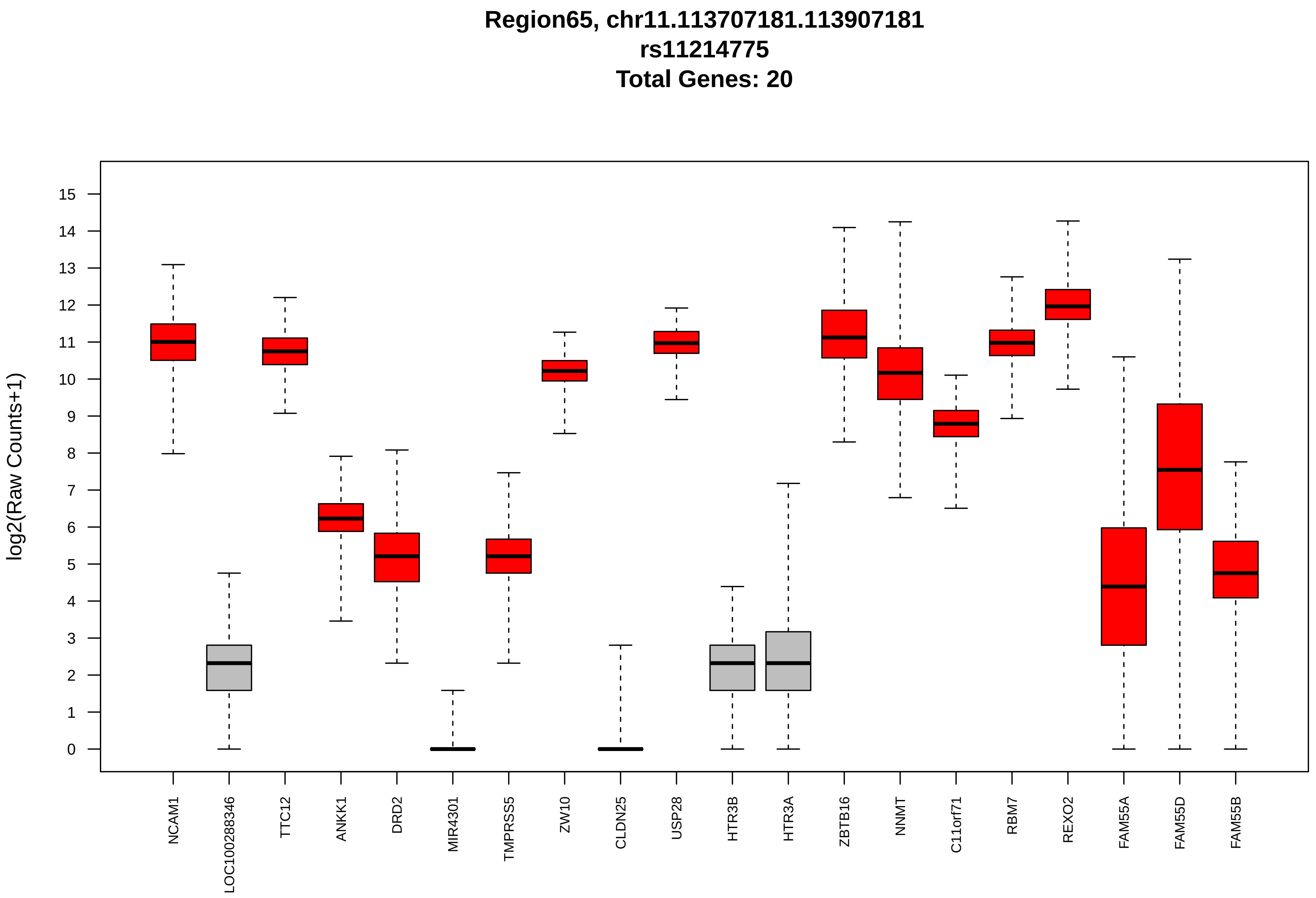

Region65, chr11.113707181.113907181
rs11214775
Total Genes: 20
log2(Raw Counts+1)
0
1
2
3
4
5
6
7
8
9
10
11
12
13
14
15
NCAM1
LOC100288346
TTC12
ANKK1
DRD2
MIR4301
TMPRSS5
ZW10
CLDN25
USP28
HTR3B
HTR3A
ZBTB16
NNMT
C11orf71
RBM7
REXO2
FAM55A
FAM55D
FAM55B

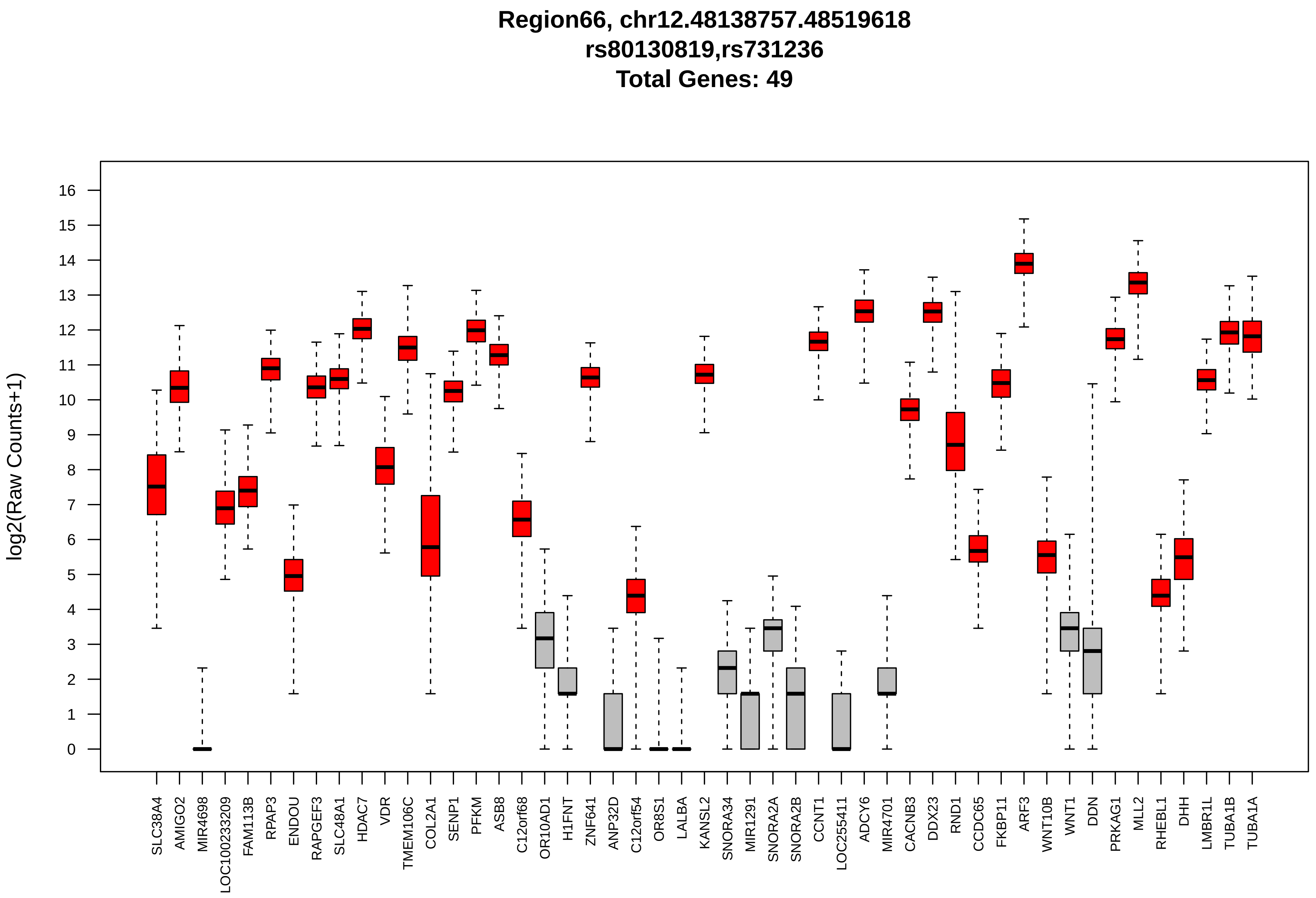
Region66, chr12.48138757.48519618
rs80130819,rs731236
Total Genes: 49
log2(Raw Counts+1)
0
1
2
3
4
5
6
7
8
9
10
11
12
13
14
15
16
SLC38A4
AMIGO2
MIR4698
LOC100233209
FAM113B
RPAP3
ENDOU
RAPGEF3
SLC48A1
HDAC7
VDR
TMEM106C
COL2A1
SENP1
PFKM
ASB8
C12orf68
OR10AD1
H1FNT
ZNF641
ANP32D
C12orf54
OR8S1
LALBA
KANSL2
SNORA34
MIR1291
SNORA2A
SNORA2B
CCNT1
LOC255411
ADCY6
MIR4701
CACNB3
DDX23
RND1
CCDC65
FKBP11
ARF3
WNT10B
WNT1
DDN
PRKAG1
MLL2
RHEBL1
DHH
LMBR1L
TUBA1B
TUBA1A

Region67, chr12.49576010.49776010
rs10875943
Total Genes: 65

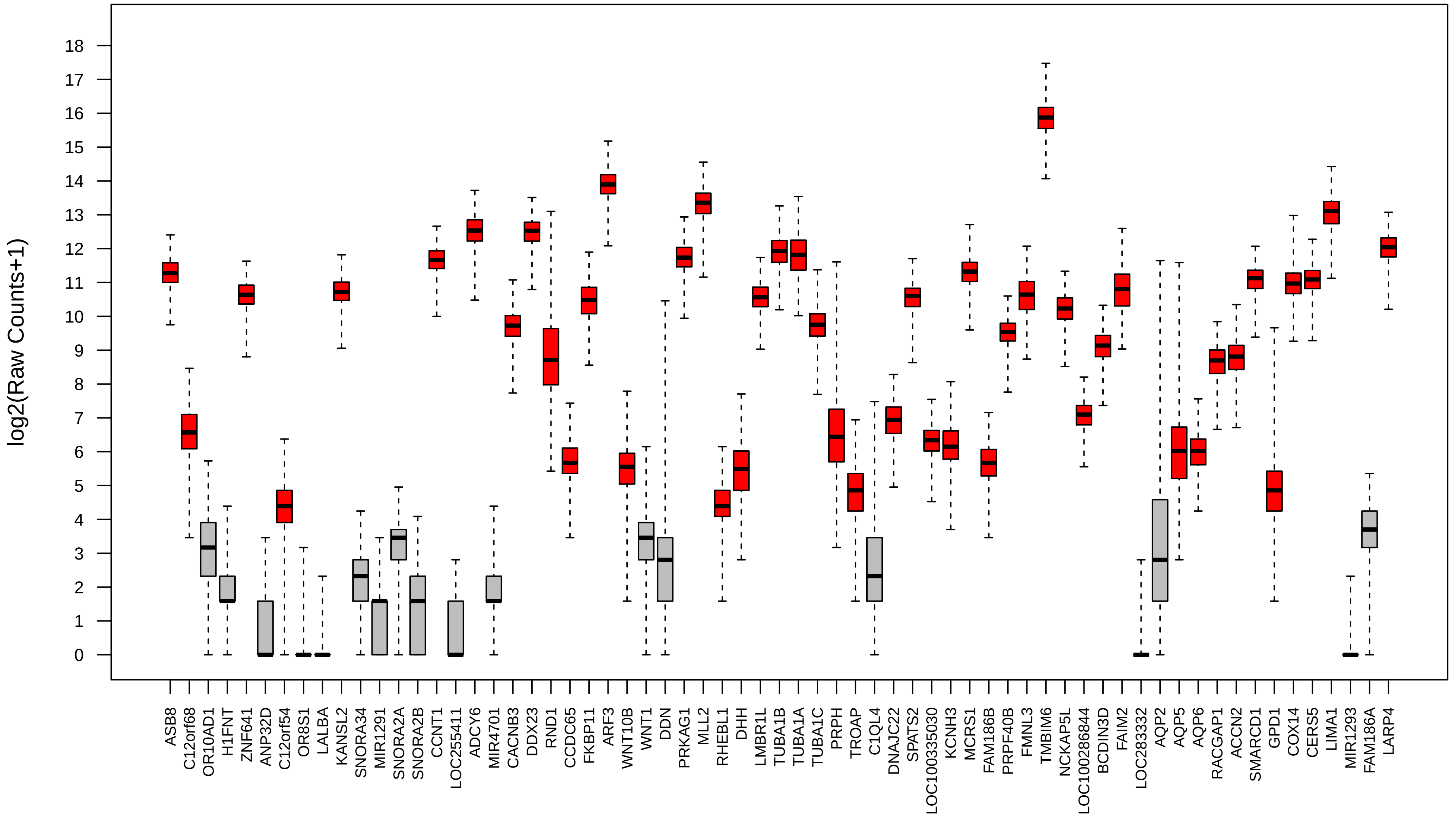

Region68, chr12.53173904.53373904
rs902774
Total Genes: 76

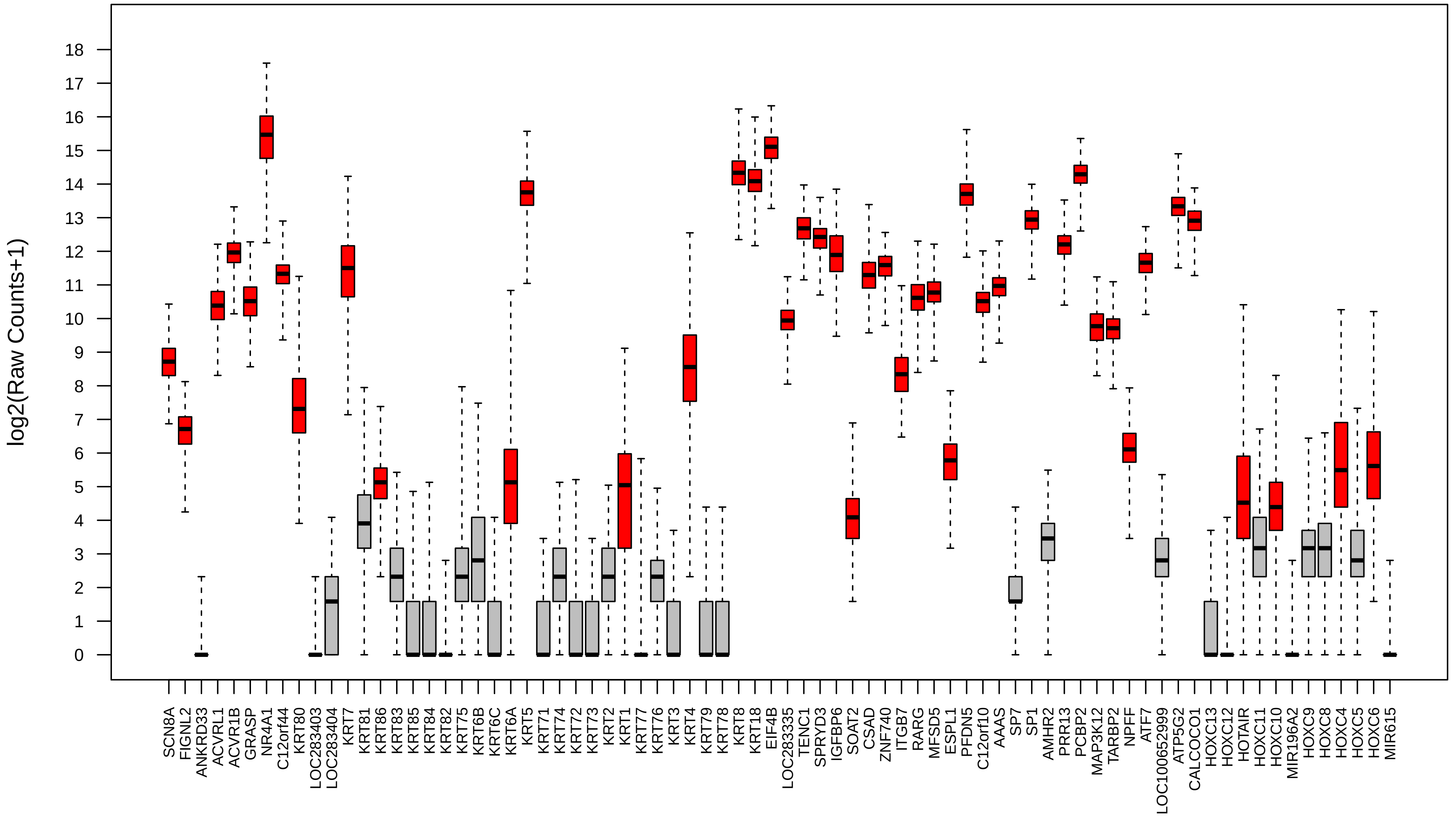

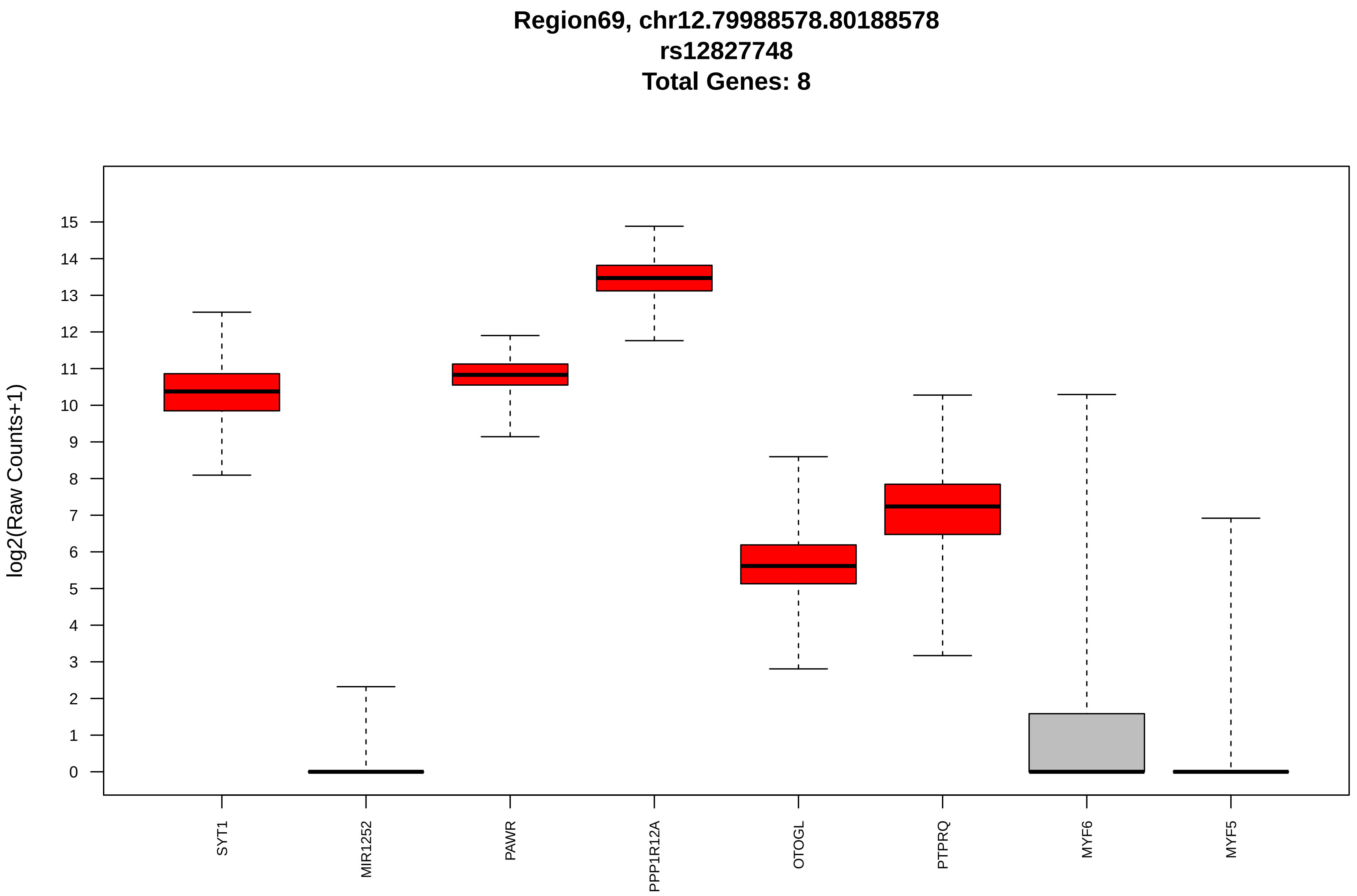
Region69, chr12.79988578.80188578
rs12827748
Total Genes: 8
log2(Raw Counts+1)
0
1
2
3
4
5
6
7
8
9
10
11
12
13
14
15
SYT1
MIR1252
PAWR
PPP1R12A
OTOGL
PTPRQ
MYF6
MYF5

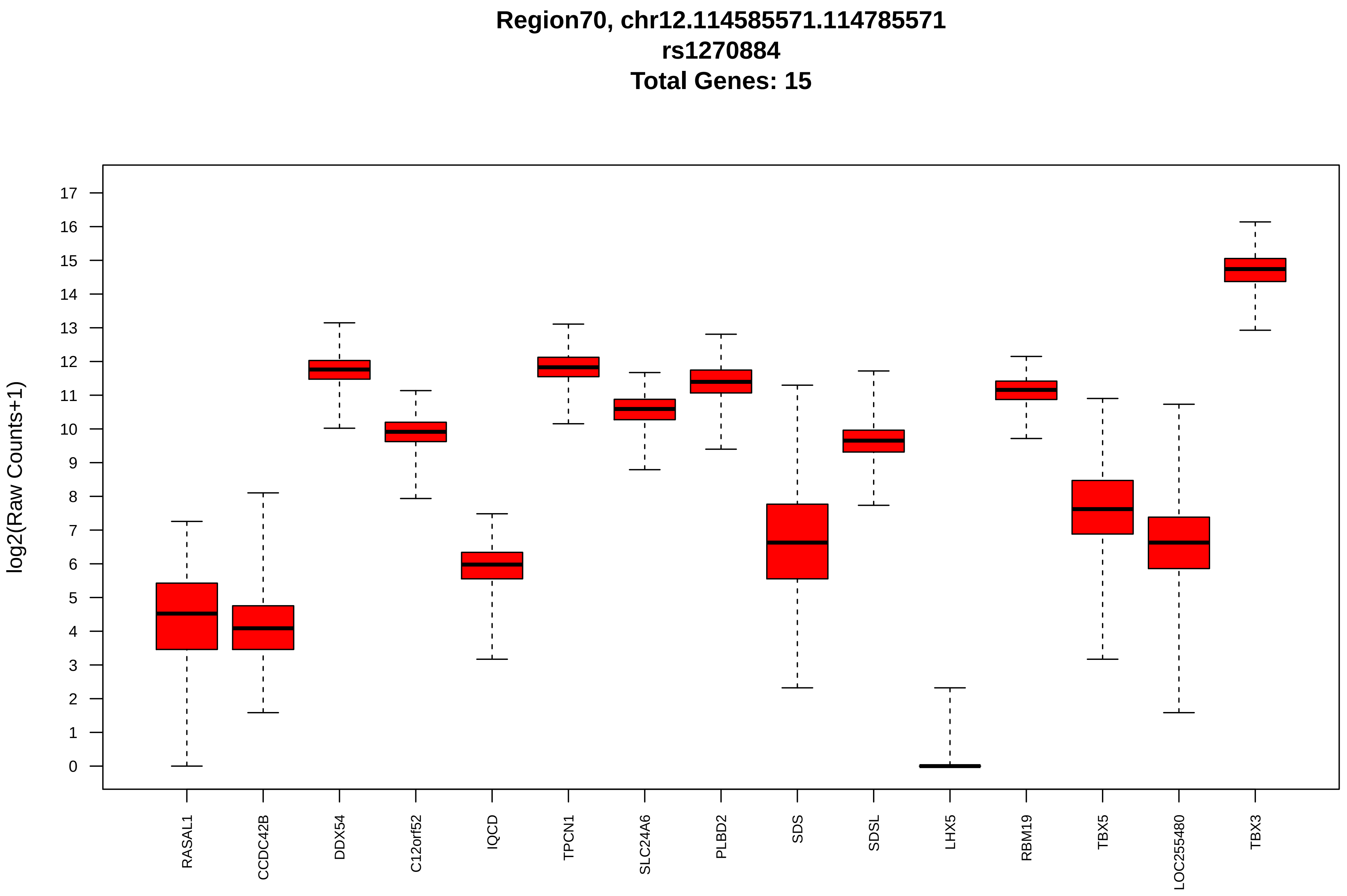
Region70, chr12.114585571.114785571
rs1270884
Total Genes: 15
log2(Raw Counts+1)
0
1
2
3
4
5
6
7
8
9
10
11
12
13
14
15
16
17
RASAL1
CCDC42B
DDX54
C12orf52
IQCD
TPCN1
SLC24A6
PLBD2
SDS
SDSL
LHX5
RBM19
TBX5
LOC255480
TBX3

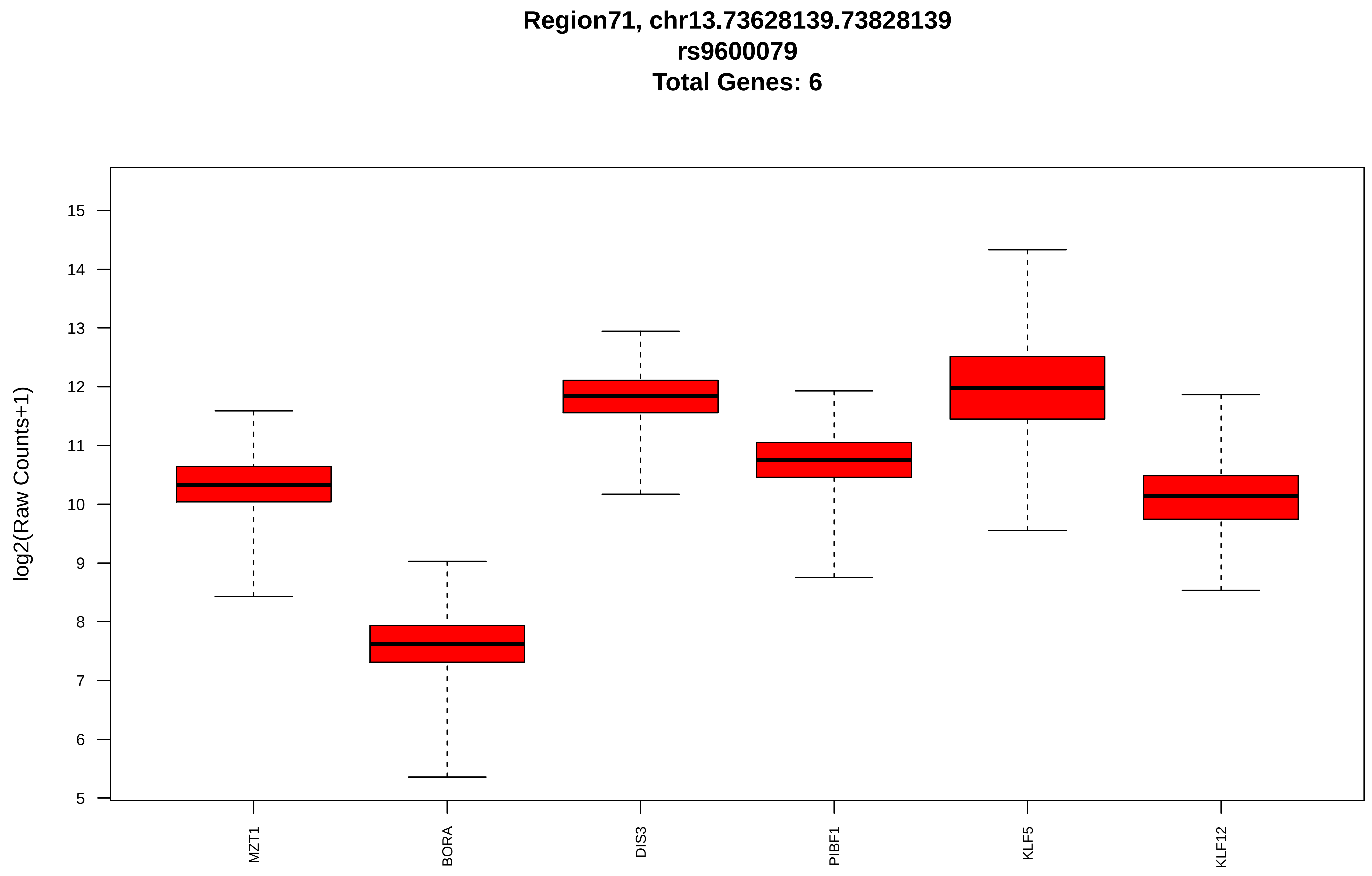
Region71, chr13.73628139.73828139
rs9600079
Total Genes: 6
log2(Raw Counts+1)
15
14
13
12
11
10
9
8
7
6
5
MZT1
BORA
DIS3
PIBF1
KLF5
KLF12

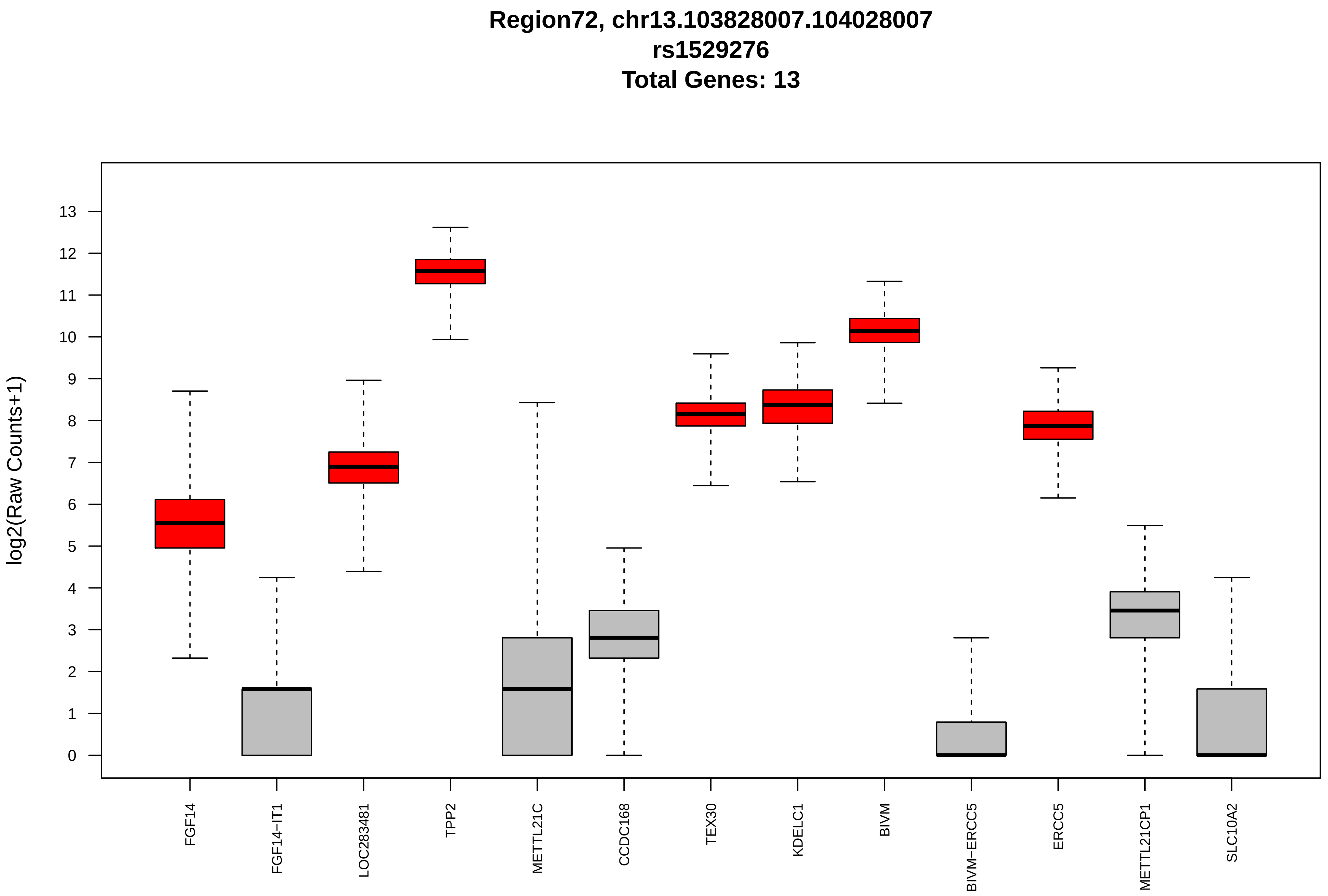

Region72, chr13.103828007.104028007
rs1529276
Total Genes: 13
log2(Raw Counts+1)
0
1
2
3
4
5
6
7
8
9
10
11
12
13
FGF14
FGF14−IT1
LOC283481
TPP2
METTL21C
CCDC168
TEX30
KDELC1
BIVM
BIVM−ERCC5
ERCC5
METTL21CP1
SLC10A2

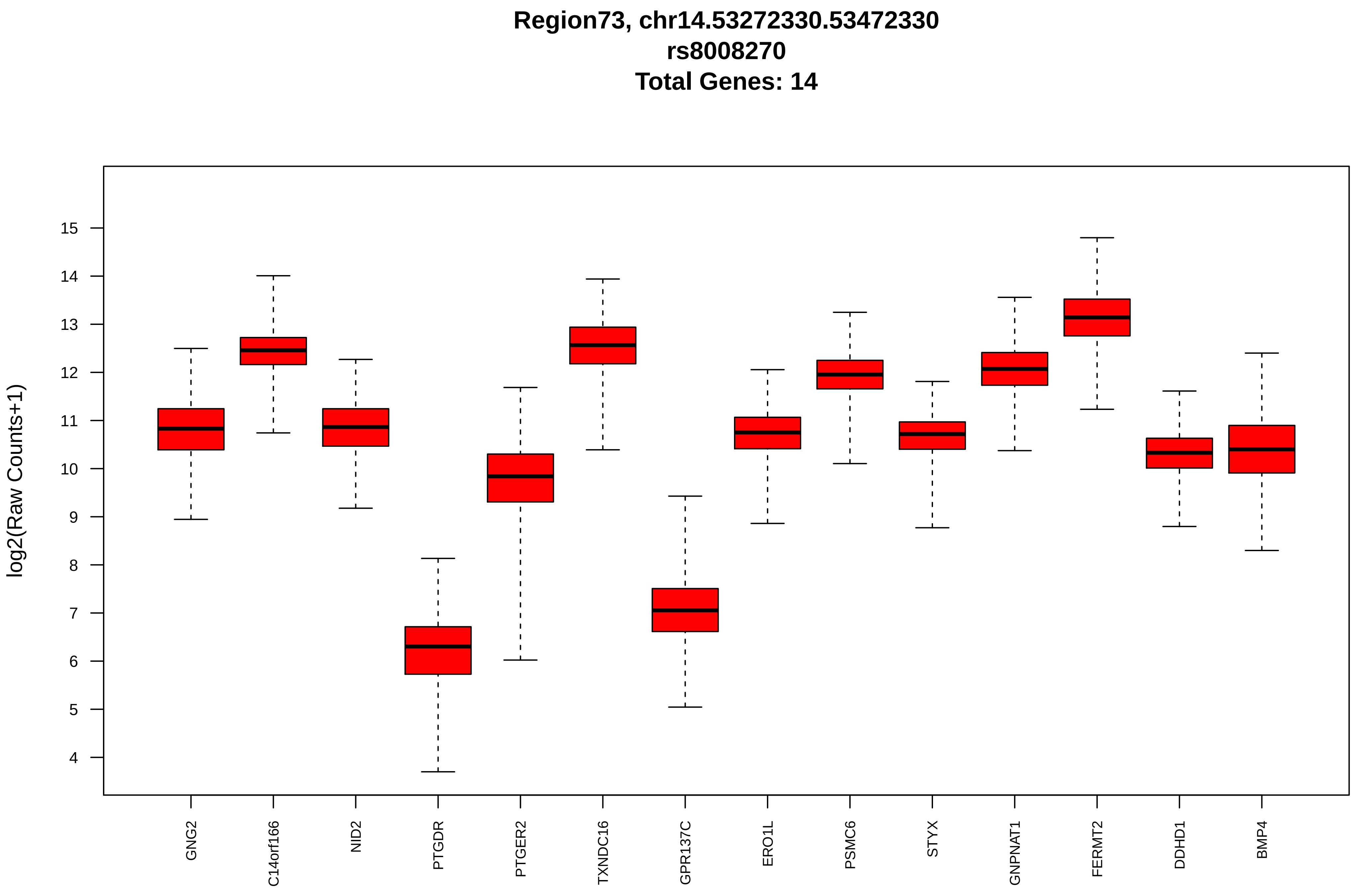

Region73, chr14.53272330.53472330
rs8008270
Total Genes: 14
log2(Raw Counts+1)
4
5
6
7
8
9
10
11
12
13
14
15
GNG2
C14orf166
NID2
PTGDR
PTGER2
TXNDC16
GPR137C
ERO1L
PSMC6
STYX
GNPNAT1
FERMT2
DDHD1
BMP4

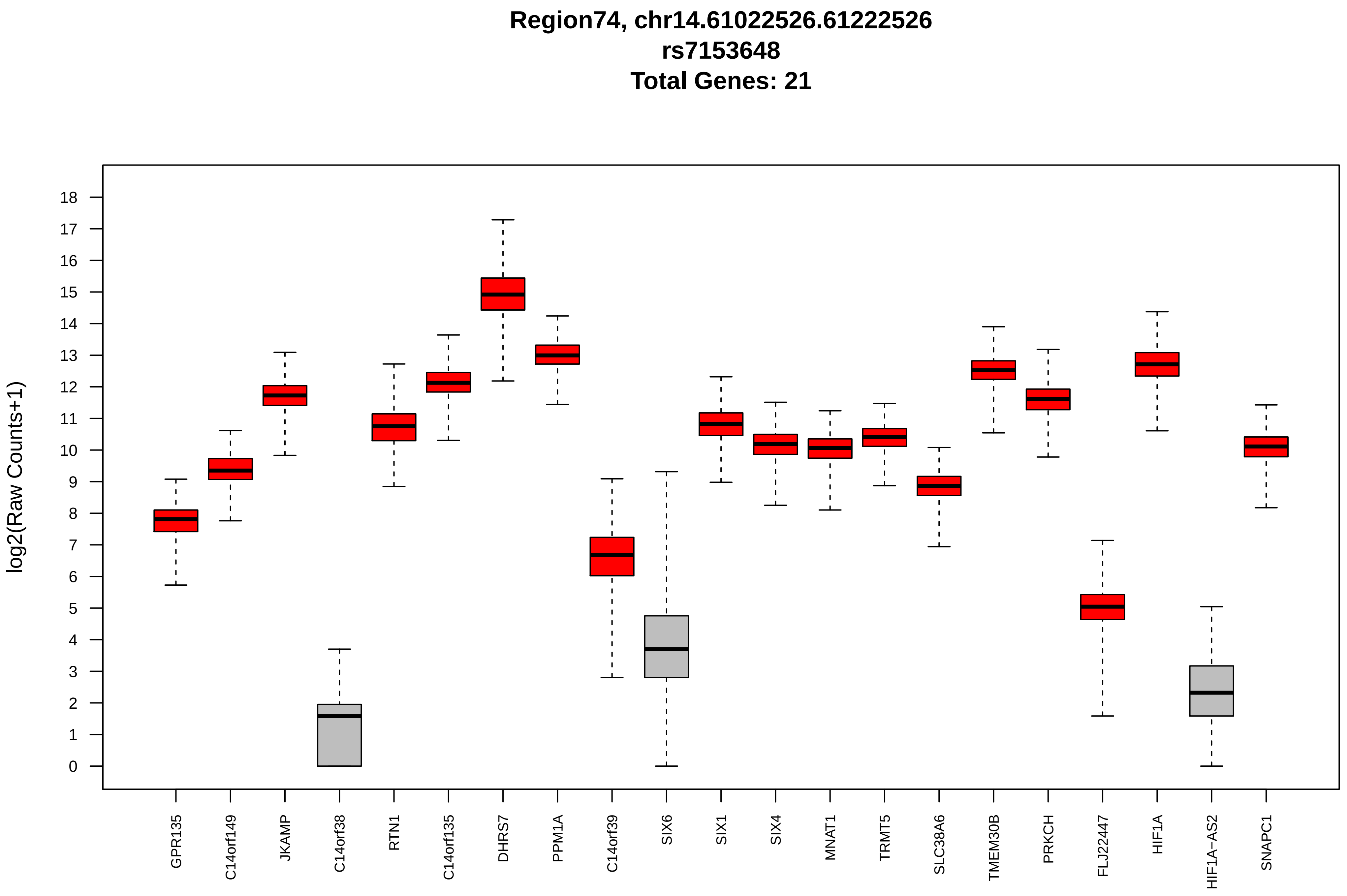
Region74, chr14.61022526.61222526
rs7153648
Total Genes: 21
log2(Raw Counts+1)
0
1
2
3
4
5
6
7
8
9
10
11
12
13
14
15
16
17
18
GPR135
C14orf149
JKAMP
C14orf38
RTN1
C14orf135
DHRS7
PPM1A
C14orf39
SIX6
SIX1
SIX4
MNAT1
TRMT5
SLC38A6
TMEM30B
PRKCH
FLJ22447
HIF1A
HIF1A−AS2
SNAPC1

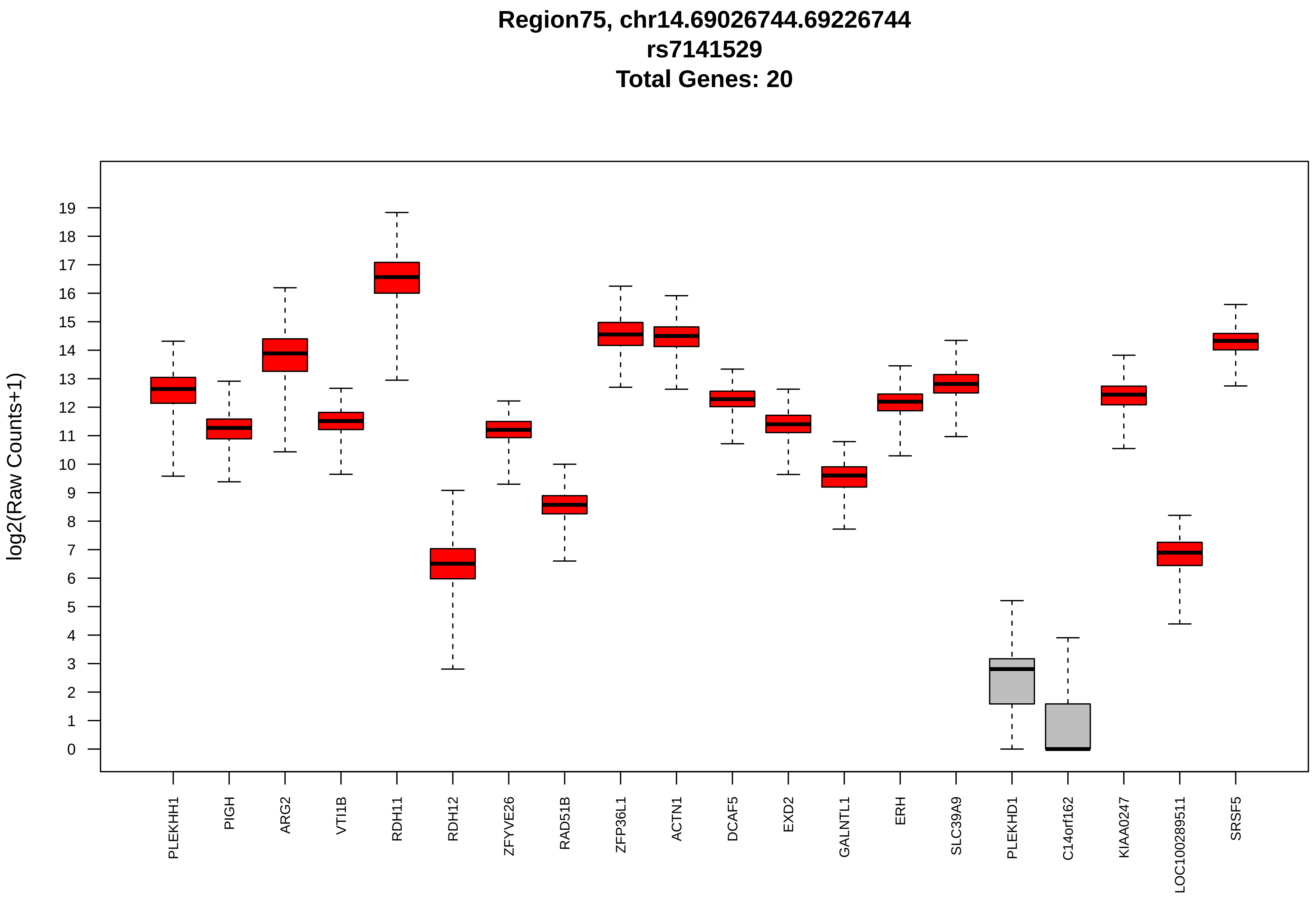
Region75, chr14.69026744.69226744
rs7141529
Total Genes: 20
log2(Raw Counts+1)
0
1
2
3
4
5
6
7
8
9
10
11
12
13
14
15
16
17
18
19
PLEKHH1
PIGH
ARG2
VTI1B
RDH11
RDH12
ZFYVE26
RAD51B
ZFP36L1
ACTN1
DCAF5
EXD2
GALNTL1
ERH
SLC39A9
PLEKHD1
C14orf162
KIAA0247
LOC100289511
SRSF5

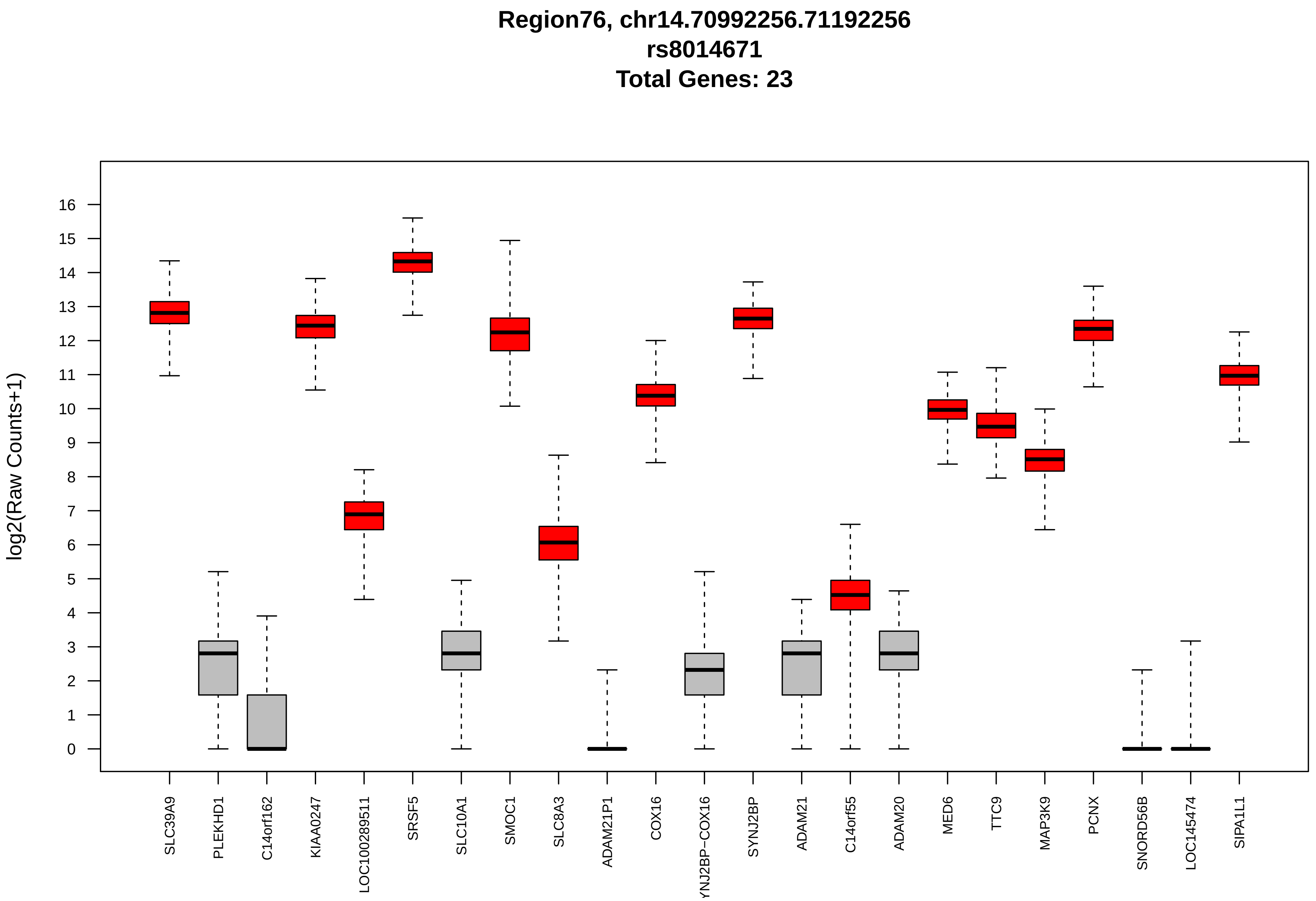

Region76, chr14.70992256.71192256
rs8014671
Total Genes: 23
log2(Raw Counts+1)
0
1
2
3
4
5
6
7
8
9
10
11
12
13
14
15
16
SLC39A9
PLEKHD1
C14orf162
KIAA0247
LOC100289511
SRSF5
SLC10A1
SMOC1
SLC8A3
ADAM21P1
COX16
SYNJ2BP−COX16
SYNJ2BP
ADAM21
C14orf55
ADAM20
MED6
TTC9
MAP3K9
PCNX
SNORD56B
LOC145474
SIPA1L1

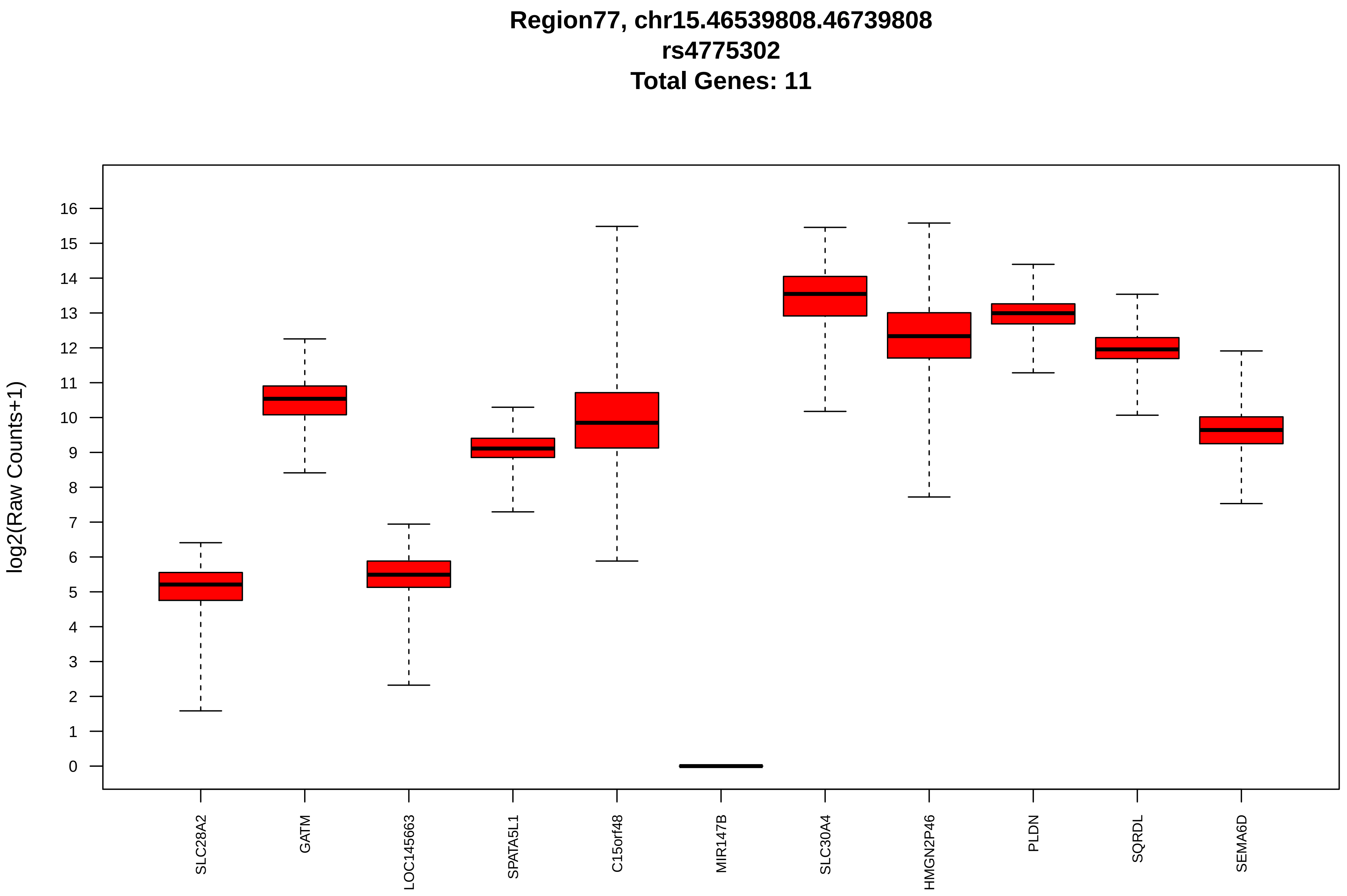
Region77, chr15.46539808.46739808
rs4775302
Total Genes: 11
log2(Raw Counts+1)
0
1
2
3
4
5
6
7
8
9
10
11
12
13
14
15
16
SLC28A2
GATM
LOC145663
SPATA5L1
C15orf48
MIR147B
SLC30A4
HMGN2P46
PLDN
SQRDL
SEMA6D

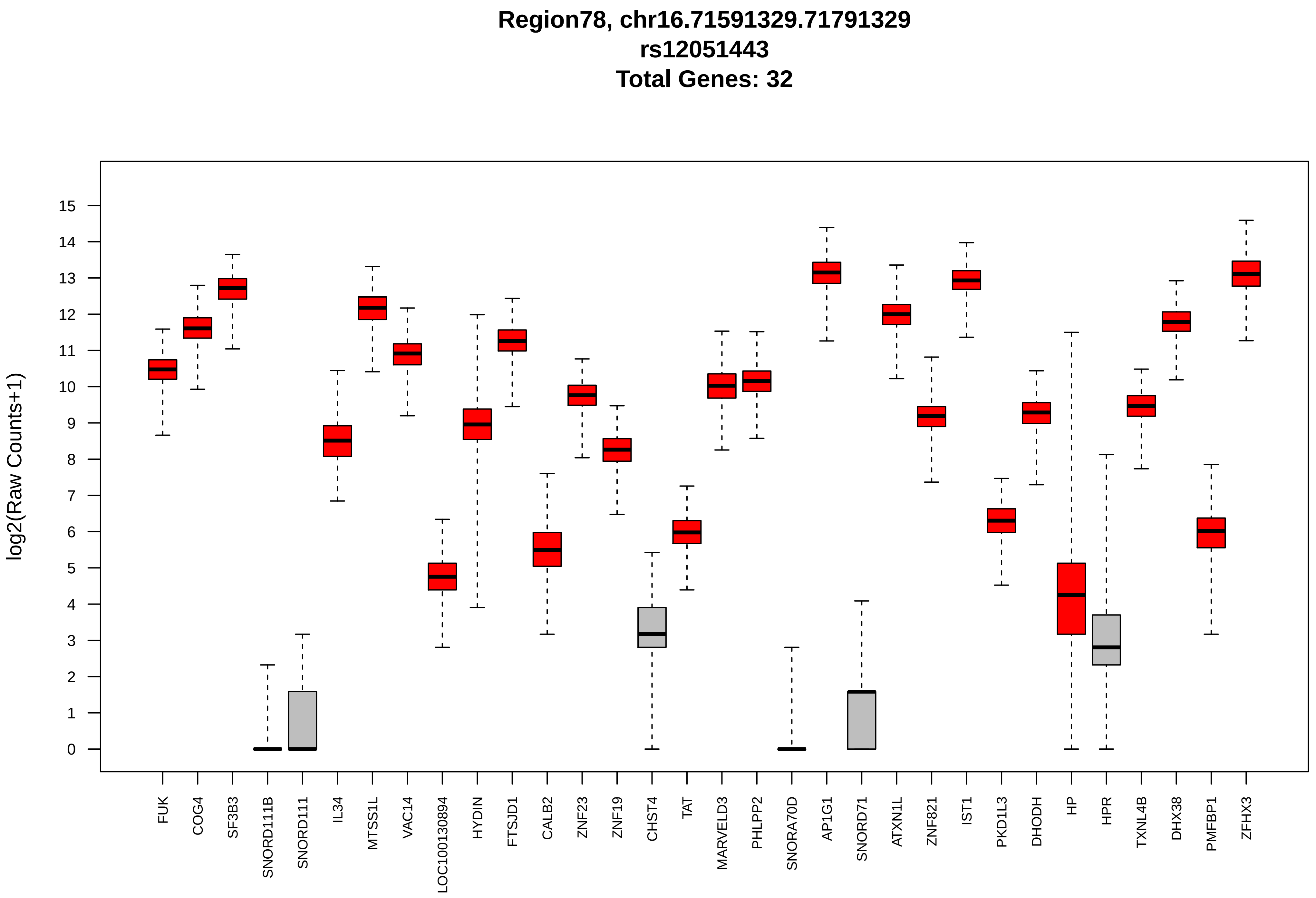

Region78, chr16.71591329.71791329
rs12051443
Total Genes: 32
log2(Raw Counts+1)
0
1
2
3
4
5
6
7
8
9
10
11
12
13
14
15
FUK
COG4
SF3B3
SNORD111B
SNORD111
IL34
MTSS1L
VAC14
LOC100130894
HYDIN
FTSJD1
CALB2
ZNF23
ZNF19
CHST4
TAT
MARVELD3
PHLPP2
SNORA70D
AP1G1
SNORD71
ATXN1L
ZNF821
IST1
PKD1L3
DHODH
HP
HPR
TXNL4B
DHX38
PMFBP1
ZFHX3

Region79, chr17.518965.718965
rs684232
Total Genes: 34

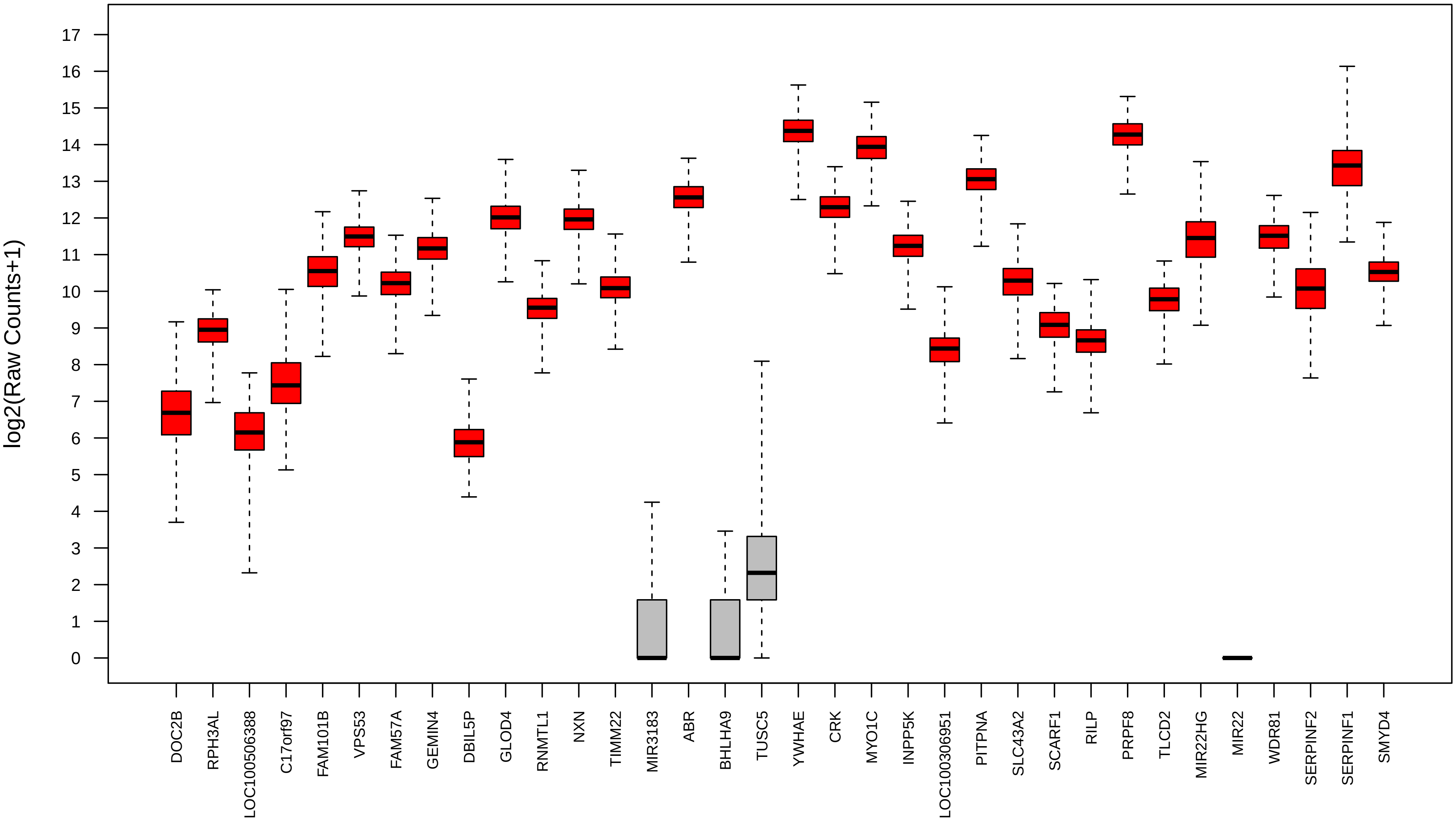

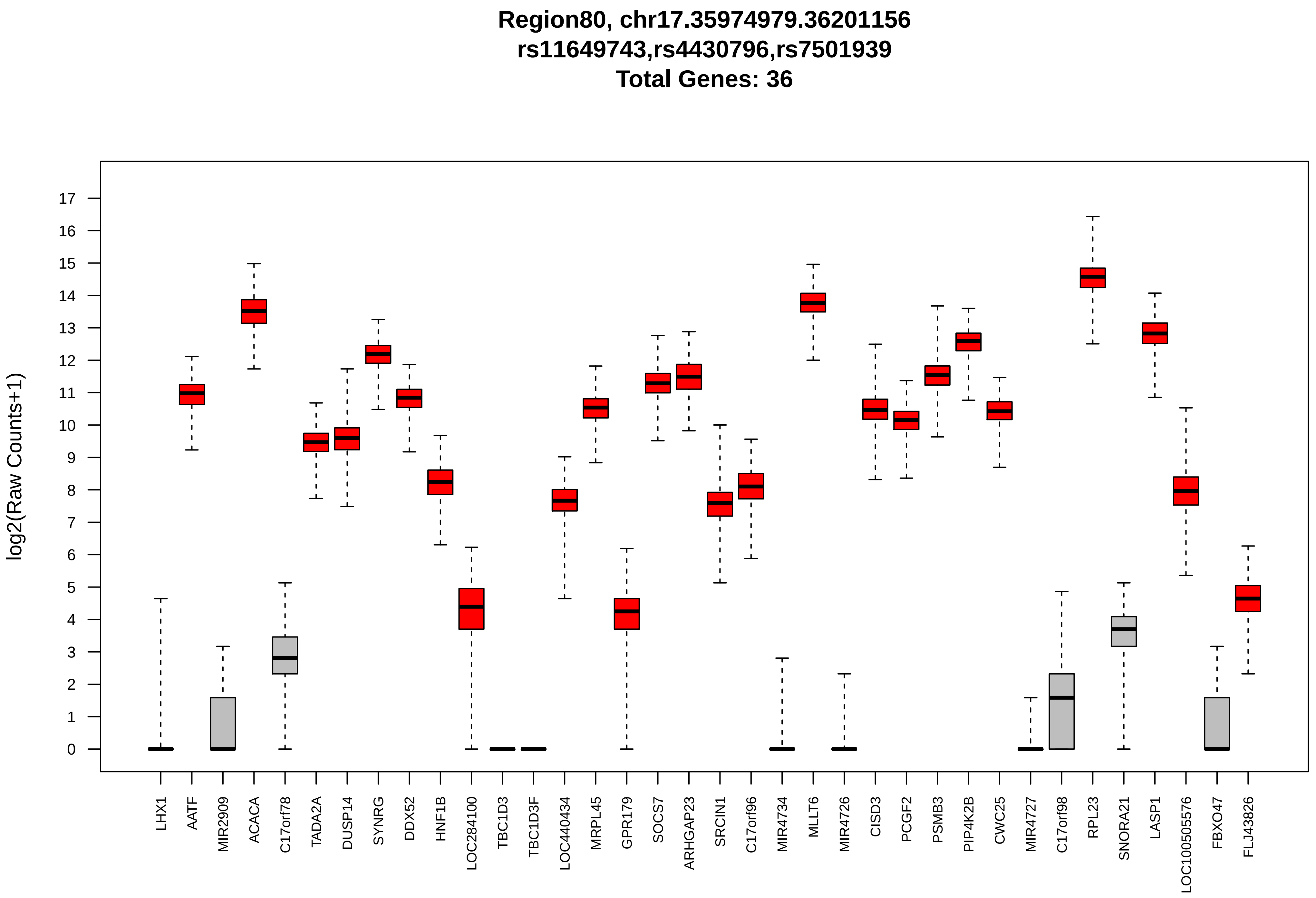

Region80, chr17.35974979.36201156
rs11649743,rs4430796,rs7501939
Total Genes: 36
log2(Raw Counts+1)
0
1
2
3
4
5
6
7
8
9
10
11
12
13
14
15
16
17
LHX1
AATF
MIR2909
ACACA
C17orf78
TADA2A
DUSP14
SYNRG
DDX52
HNF1B
LOC284100
TBC1D3
TBC1D3F
LOC440434
MRPL45
GPR179
SOCS7
ARHGAP23
SRCIN1
C17orf96
MIR4734
MLLT6
MIR4726
CISD3
PCGF2
PSMB3
PIP4K2B
CWC25
MIR4727
C17orf98
RPL23
SNORA21
LASP1
LOC100505576
FBXO47
FLJ43826

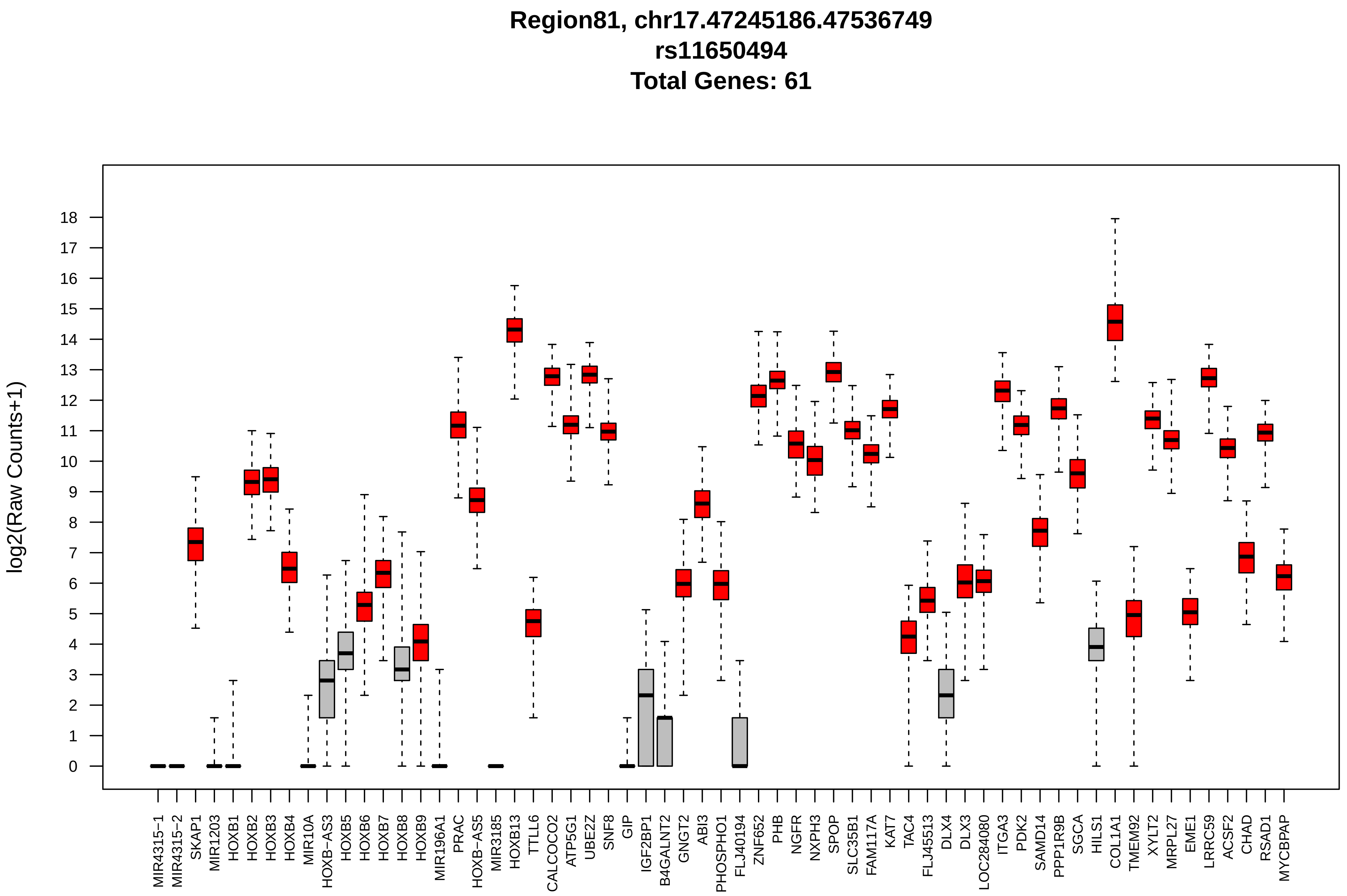
Region81, chr17.47245186.47536749
rs11650494
Total Genes: 61
log2(Raw Counts+1)
0
1
2
3
4
5
6
7
8
9
10
11
12
13
14
15
16
17
18
MIR4315−1
MIR4315−2
SKAP1
MIR1203
HOXB1
HOXB2
HOXB3
HOXB4
MIR10A
HOXB−AS3
HOXB5
HOXB6
HOXB7
HOXB8
HOXB9
MIR196A1
PRAC
HOXB−AS5
MIR3185
HOXB13
TTLL6
CALCOCO2
ATP5G1
UBE2Z
SNF8
GIP
IGF2BP1
B4GALNT2
GNGT2
ABI3
PHOSPHO1
FLJ40194
ZNF652
PHB
NGFR
NXPH3
SPOP
SLC35B1
FAM117A
KAT7
TAC4
FLJ45513
DLX4
DLX3
LOC284080
ITGA3
PDK2
SAMD14
PPP1R9B
SGCA
HILS1
COL1A1
TMEM92
XYLT2
MRPL27
EME1
LRRC59
ACSF2
CHAD
RSAD1
MYCBPAP

Region82, chr17.69008753.69208753
rs1859962
Total Genes: 4

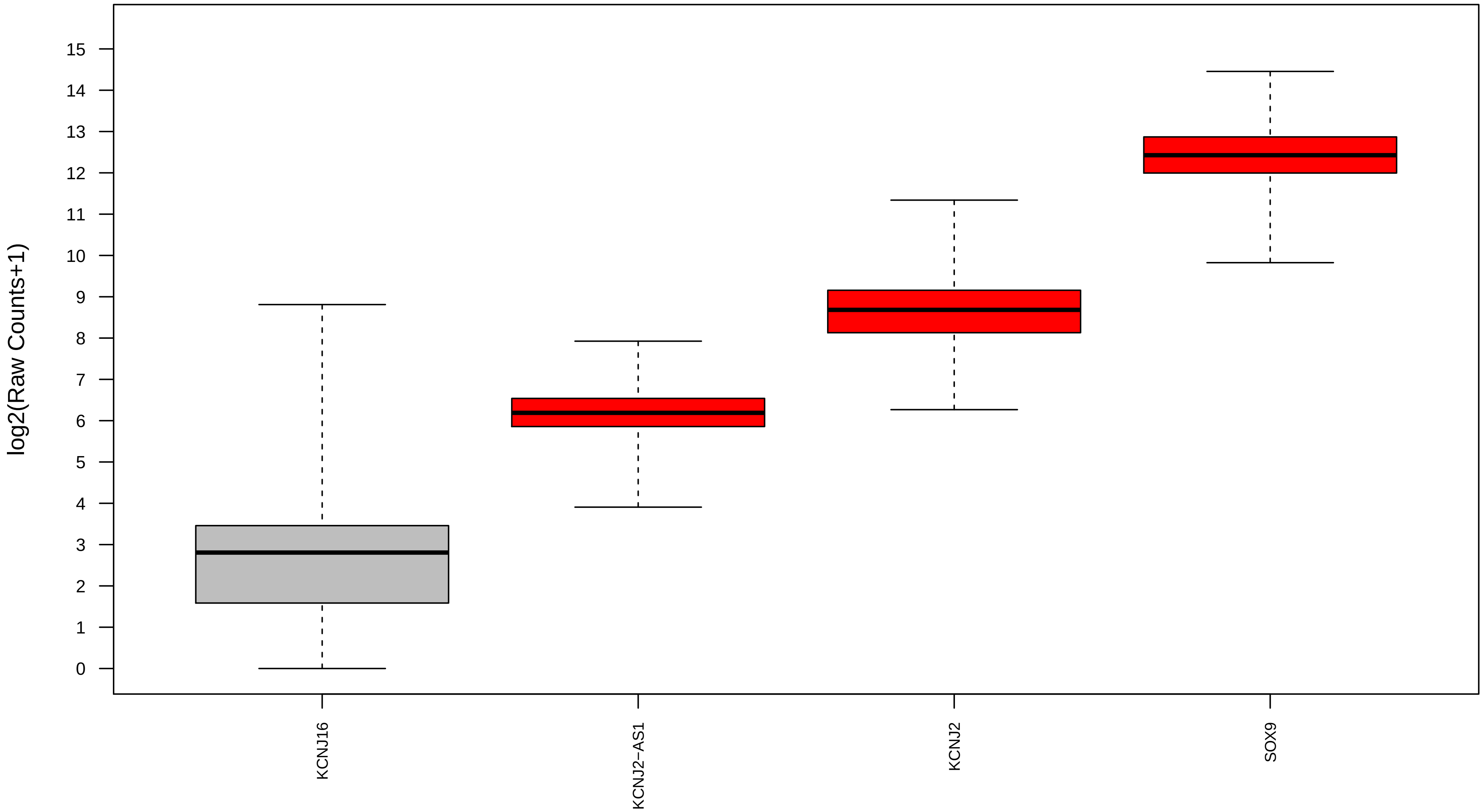

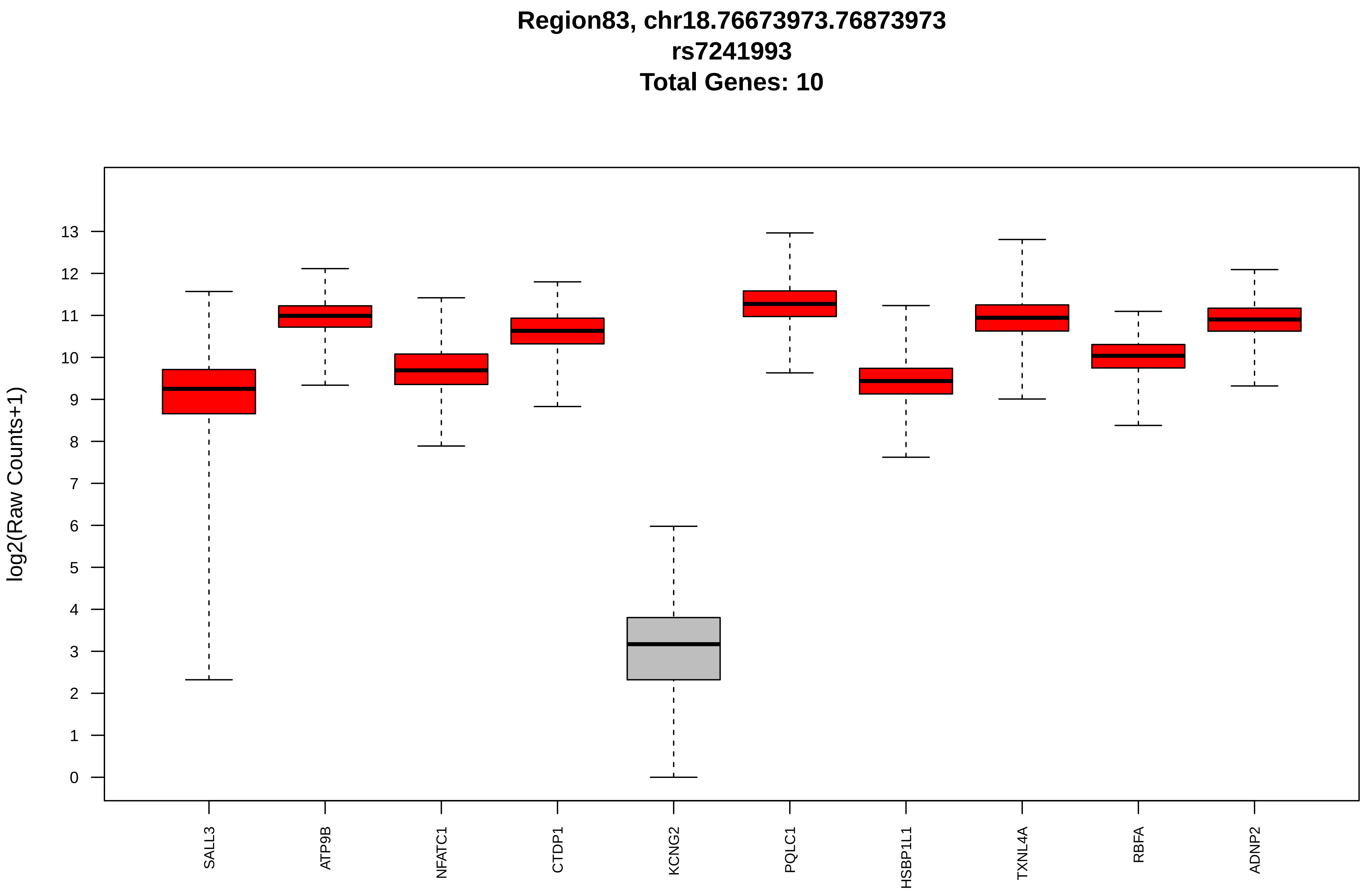

Region83, chr18.76673973.76873973
rs7241993
Total Genes: 10
log2(Raw Counts+1)
0
1
2
3
4
5
6
7
8
9
10
11
12
13
SALL3
ATP9B
NFATC1
CTDP1
KCNG2
PQLC1
HSBP1L1
TXNL4A
RBFA
ADNP2

Region84, chr19.38635613.38835613
rs8102476
Total Genes: 60

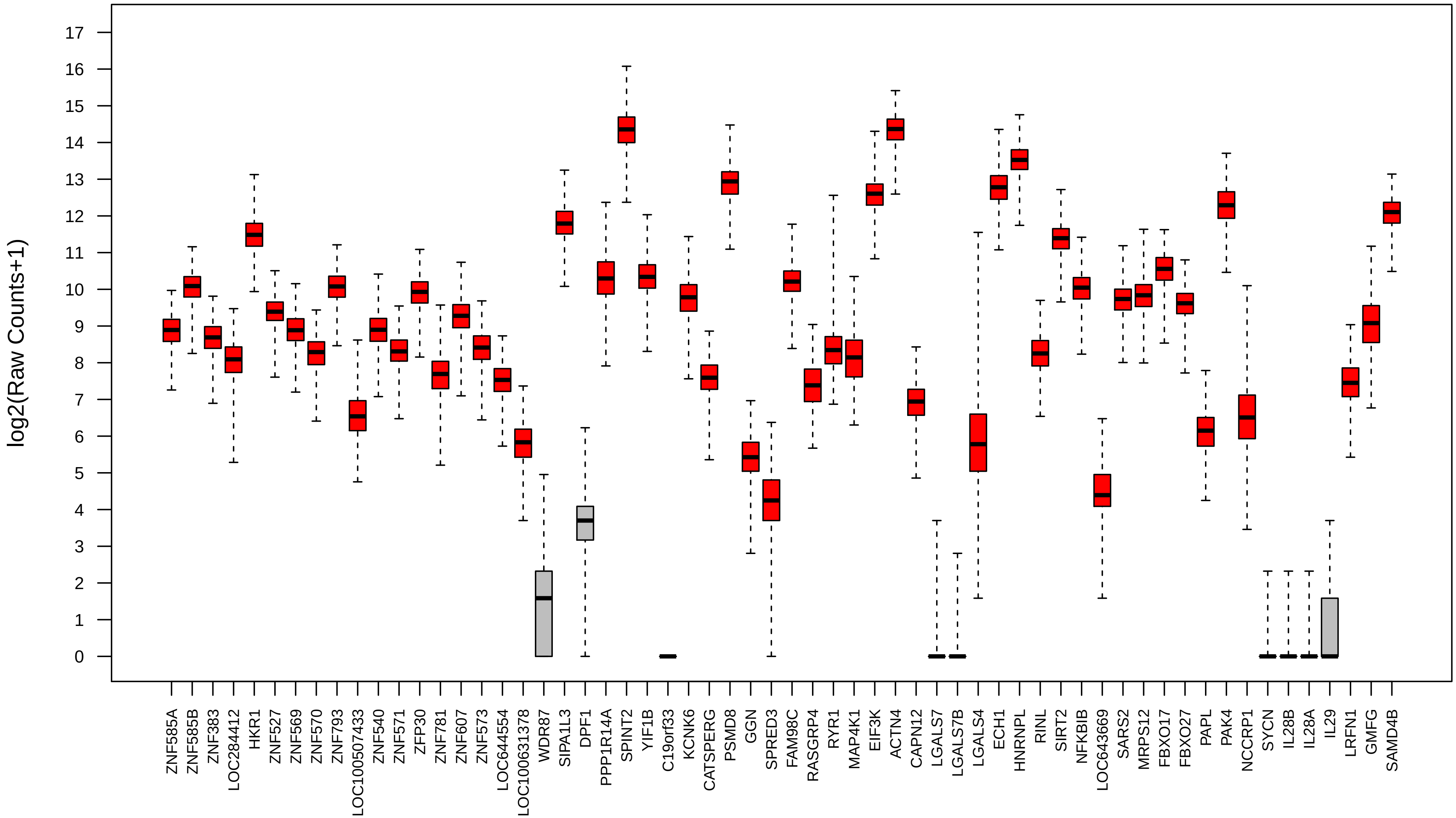

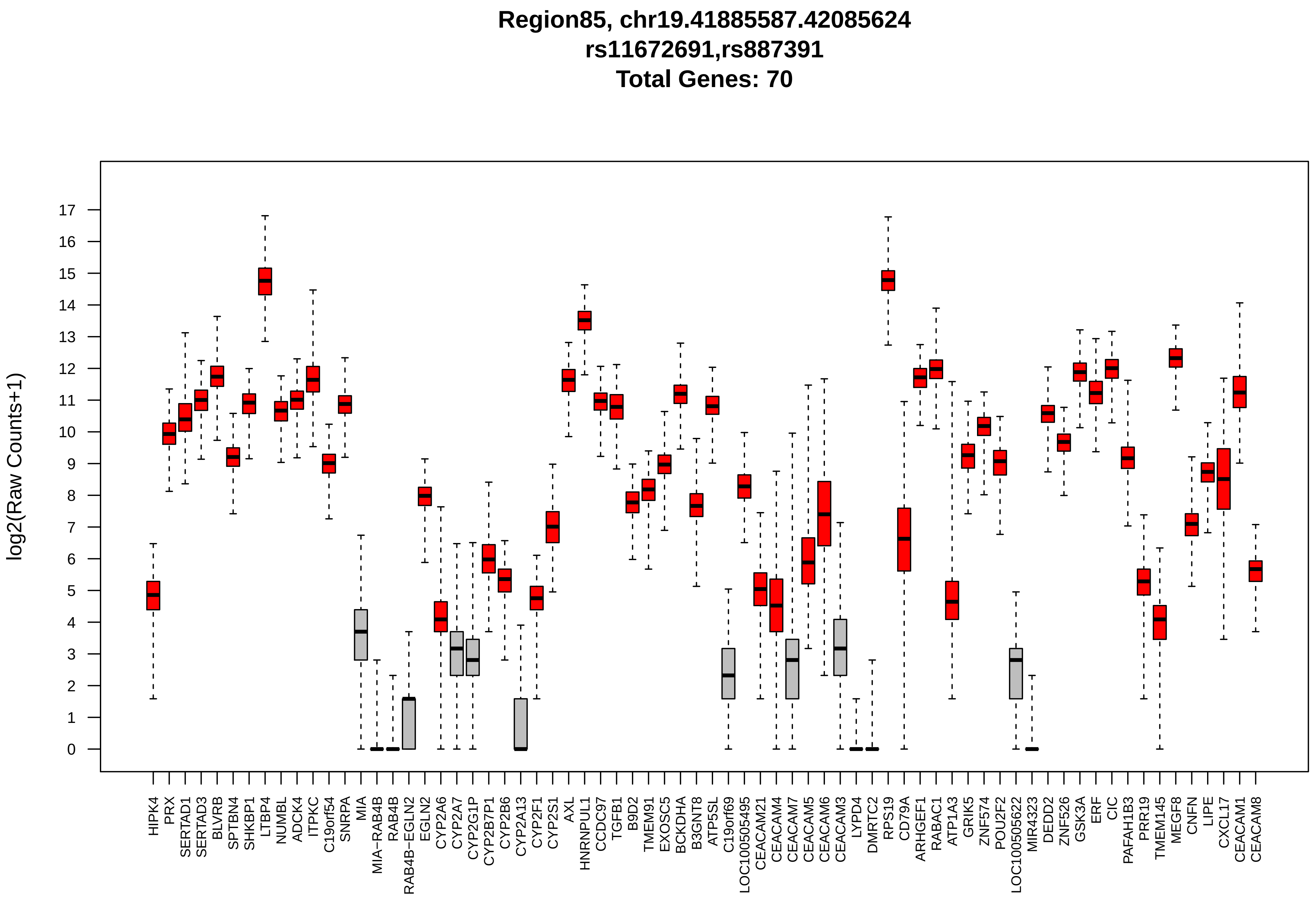

Region85, chr19.41885587.42085624
rs11672691,rs887391
Total Genes: 70
log2(Raw Counts+1)
0
1
2
3
4
5
6
7
8
9
10
11
12
13
14
15
16
17
HIPK4
PRX
SERTAD1
SERTAD3
BLVRB
SPTBN4
SHKBP1
LTBP4
NUMBL
ADCK4
ITPKC
C19orf54
SNRPA
MIA
MIA−RAB4B
RAB4B
RAB4B−EGLN2
EGLN2
CYP2A6
CYP2A7
CYP2G1P
CYP2B7P1
CYP2B6
CYP2A13
CYP2F1
CYP2S1
AXL
HNRNPUL1
CCDC97
TGFB1
B9D2
TMEM91
EXOSC5
BCKDHA
B3GNT8
ATP5SL
C19orf69
LOC100505495
CEACAM21
CEACAM4
CEACAM7
CEACAM5
CEACAM6
CEACAM3
LYPD4
DMRTC2
RPS19
CD79A
ARHGEF1
RABAC1
ATP1A3
GRIK5
ZNF574
POU2F2
LOC100505622
MIR4323
DEDD2
ZNF526
GSK3A
ERF
CIC
PAFAH1B3
PRR19
TMEM145
MEGF8
CNFN
LIPE
CXCL17
CEACAM1
CEACAM8

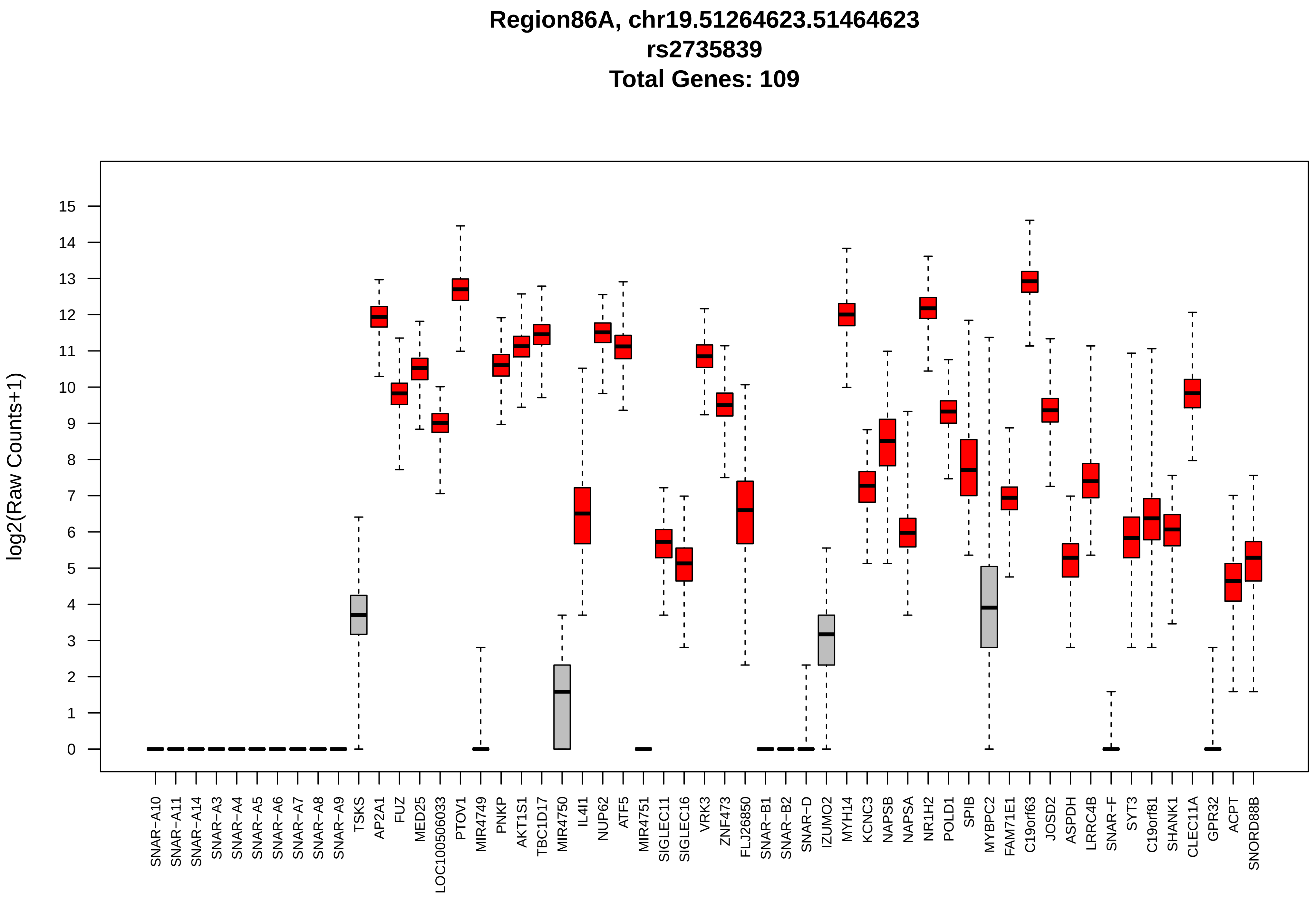

Region86A, chr19.51264623.51464623
rs2735839
Total Genes: 109
log2(Raw Counts+1)
0
1
2
3
4
5
6
7
8
9
10
11
12
13
14
15
SNAR−A10
SNAR−A11
SNAR−A14
SNAR−A3
SNAR−A4
SNAR−A5
SNAR−A6
SNAR−A7
SNAR−A8
SNAR−A9
TSKS
AP2A1
FUZ
MED25
LOC100506033
PTOV1
MIR4749
PNKP
AKT1S1
TBC1D17
MIR4750
IL4I1
NUP62
ATF5
MIR4751
SIGLEC11
SIGLEC16
VRK3
ZNF473
FLJ26850
SNAR−B1
SNAR−B2
SNAR−D
IZUMO2
MYH14
KCNC3
NAPSB
NAPSA
NR1H2
POLD1
SPIB
MYBPC2
FAM71E1
C19orf63
JOSD2
ASPDH
LRRC4B
SNAR−F
SYT3
C19orf81
SHANK1
CLEC11A
GPR32
ACPT
SNORD88B

Region86B, chr19.51264623.51464623
rs2735839
Total Genes: 109

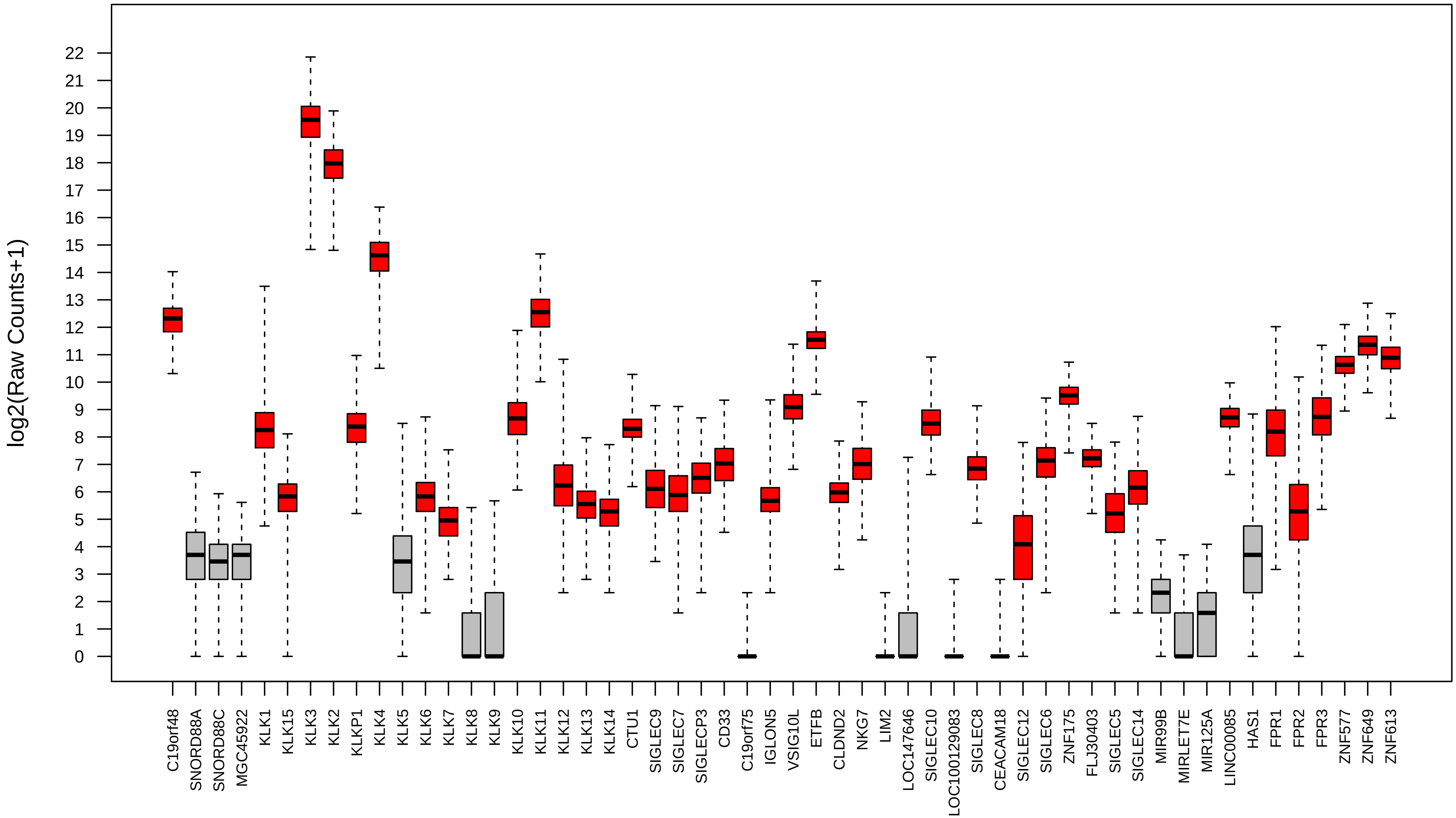

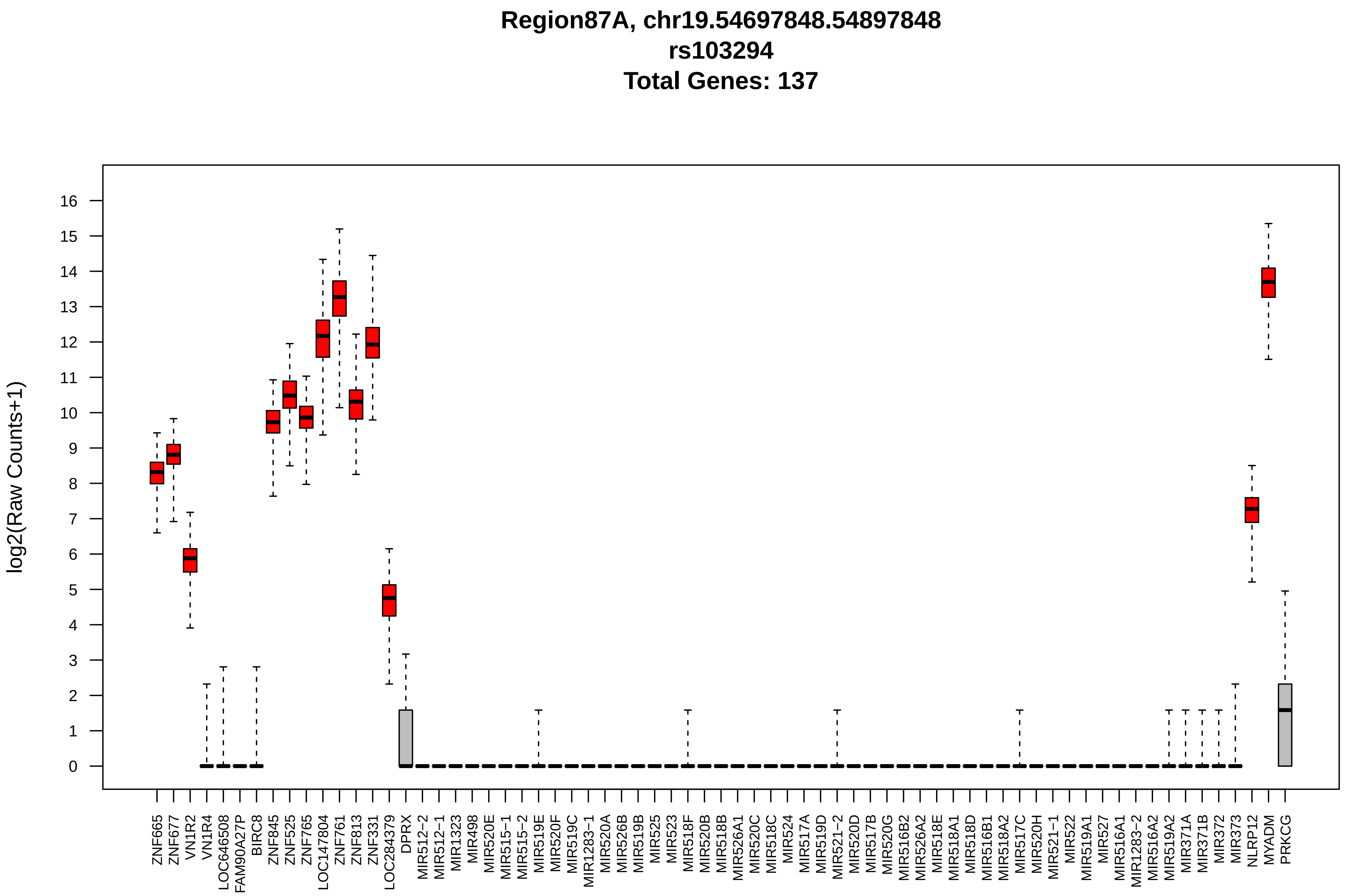

Region87A, chr19.54697848.54897848
rs103294
Total Genes: 137
log2(Raw Counts+1)
0
1
2
3
4
5
6
7
8
9
10
11
12
13
14
15
16
ZNF665
ZNF677
VN1R2
VN1R4
LOC646508
FAM90A27P
BIRC8
ZNF845
ZNF525
ZNF765
LOC147804
ZNF761
ZNF813
ZNF331
LOC284379
DPRX
MIR512−2
MIR512−1
MIR1323
MIR498
MIR520E
MIR515−1
MIR515−2
MIR519E
MIR520F
MIR519C
MIR1283−1
MIR520A
MIR526B
MIR519B
MIR525
MIR523
MIR518F
MIR520B
MIR518B
MIR526A1
MIR520C
MIR518C
MIR524
MIR517A
MIR519D
MIR521−2
MIR520D
MIR517B
MIR520G
MIR516B2
MIR526A2
MIR518E
MIR518A1
MIR518D
MIR516B1
MIR518A2
MIR517C
MIR520H
MIR521−1
MIR522
MIR519A1
MIR527
MIR516A1
MIR1283−2
MIR516A2
MIR519A2
MIR371A
MIR371B
MIR372
MIR373
NLRP12
MYADM
PRKCG

Region87B, chr19.54697848.54897848
rs103294
Total Genes: 137

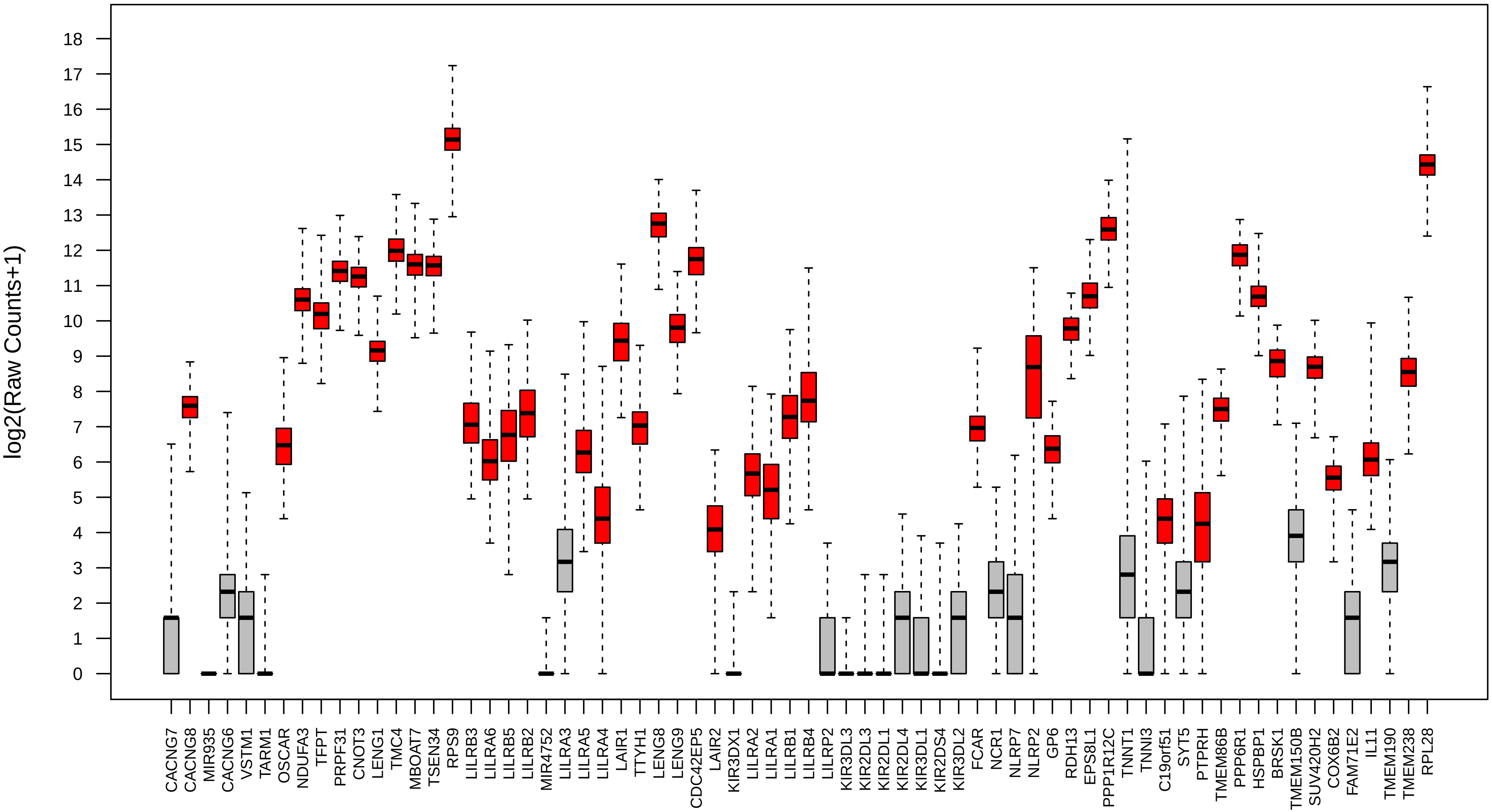

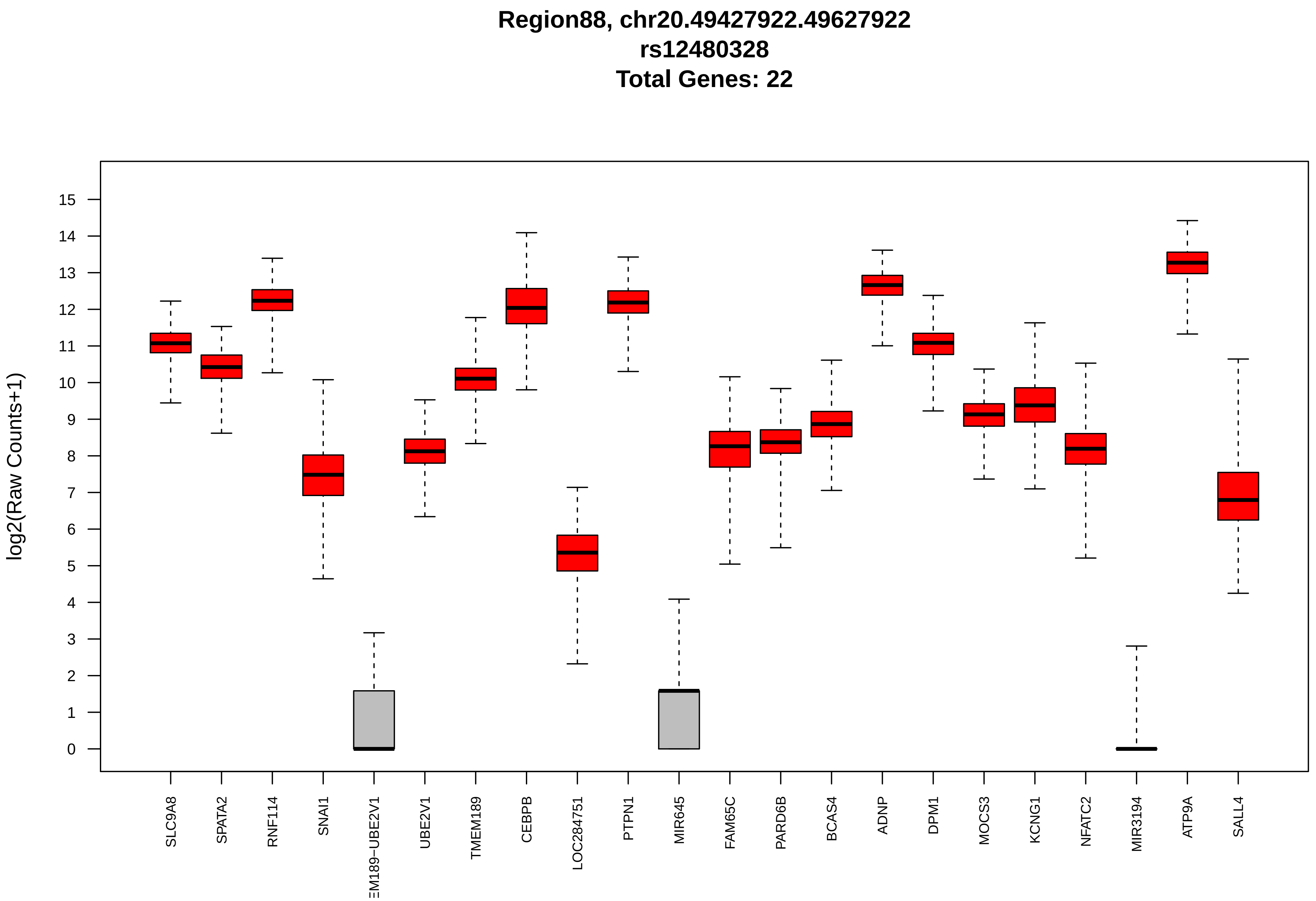
Region88, chr20.49427922.49627922
rs12480328
Total Genes: 22
log2(Raw Counts+1)
0
1
2
3
4
5
6
7
8
9
10
11
12
13
14
15
SLC9A8
SPATA2
RNF114
SNAI1
EM189−UBE2V1
UBE2V1
TMEM189
CEBPB
LOC284751
PTPN1
MIR645
FAM65C
PARD6B
BCAS4
ADNP
DPM1
MOCS3
KCNG1
NFATC2
MIR3194
ATP9A
SALL4

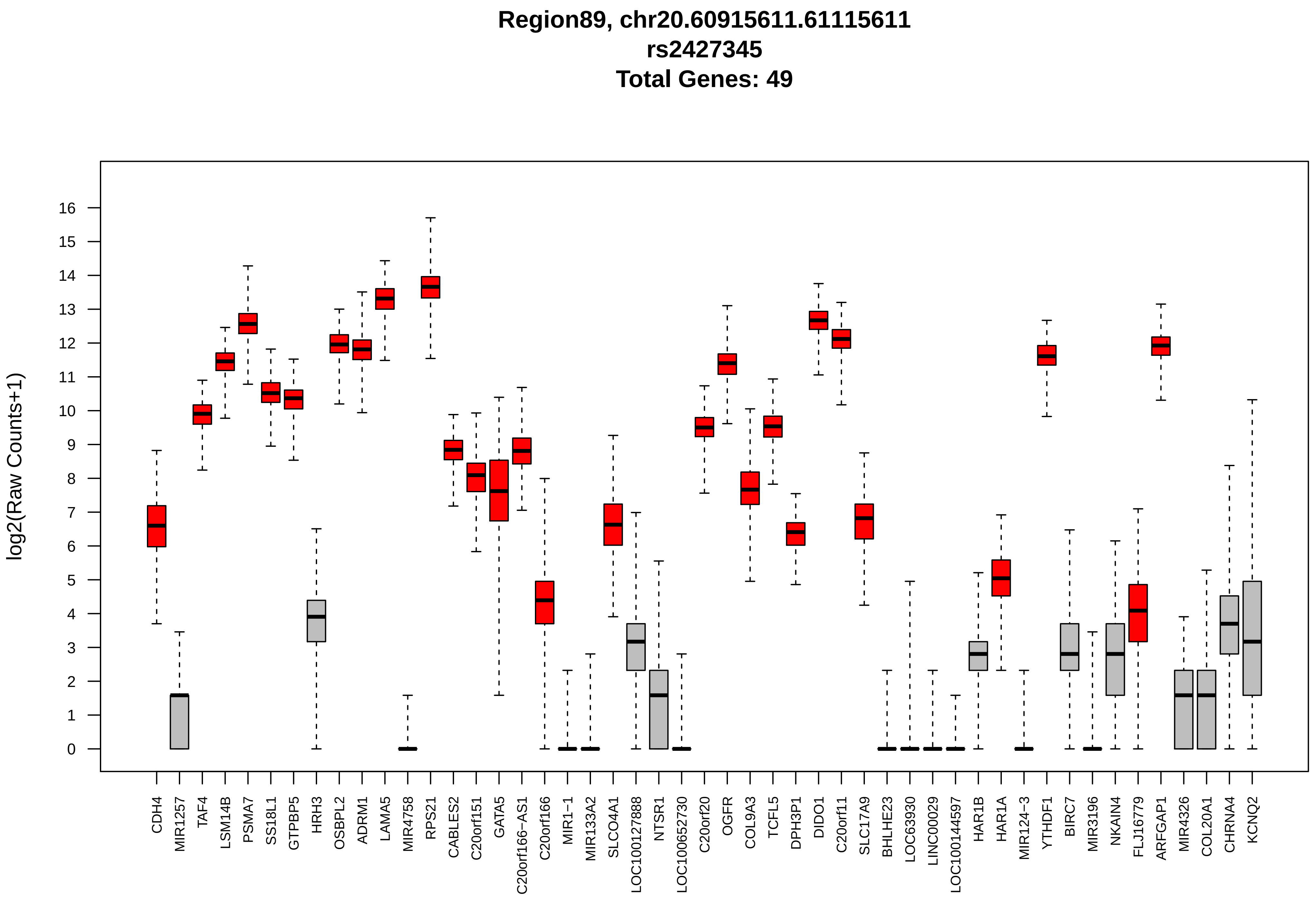
Region89, chr20.60915611.61115611
rs2427345
Total Genes: 49
log2(Raw Counts+1)
0
1
2
3
4
5
6
7
8
9
10
11
12
13
14
15
16
CDH4
MIR1257
TAF4
LSM14B
PSMA7
SS18L1
GTPBP5
HRH3
OSBPL2
ADRM1
LAMA5
MIR4758
RPS21
CABLES2
C20orf151
GATA5
C20orf166−AS1
C20orf166
MIR1−1
MIR133A2
SLCO4A1
LOC100127888
NTSR1
LOC100652730
C20orf20
OGFR
COL9A3
TCFL5
DPH3P1
DIDO1
C20orf11
SLC17A9
BHLHE23
LOC63930
LINC00029
LOC100144597
HAR1B
HAR1A
MIR124−3
YTHDF1
BIRC7
MIR3196
NKAIN4
FLJ16779
ARFGAP1
MIR4326
COL20A1
CHRNA4
KCNQ2

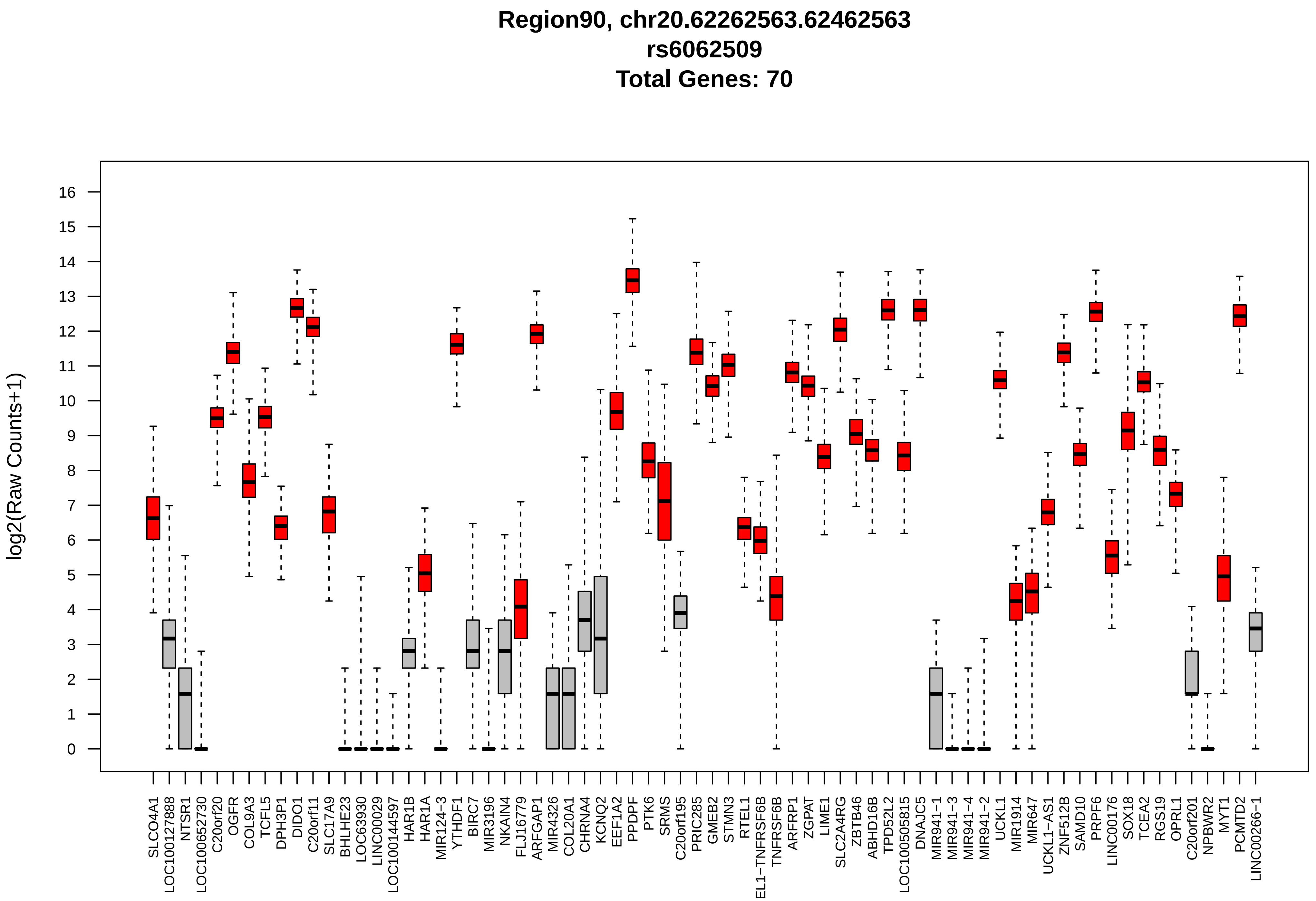
Region90, chr20.62262563.62462563
rs6062509
Total Genes: 70
log2(Raw Counts+1)
SLCO4A1
LOC100127888
NTSR1
LOC100652730
C20orf20
OGFR
COL9A3
TCFL5
DPH3P1
DIDO1
C20orf11
SLC17A9
BHLHE23
LOC63930
LINC00029
LOC100144597
HAR1B
HAR1A
MIR124−3
YTHDF1
BIRC7
MIR3196
NKAIN4
FLJ16779
ARFGAP1
MIR4326
COL20A1
CHRNA4
KCNQ2
EEF1A2
PPDPF
PTK6
SRMS
C20orf195
PRIC285
GMEB2
STMN3
RTEL1
TNFRSF6B
ARFRP1
ZGPAT
LIME1
SLC2A4RG
ZBTB46
ABHD16B
TPD52L2
LOC100505815
DNAJC5
MIR941−1
MIR941−3
MIR941−4
MIR941−2
UCKL1
MIR1914
MIR647
UCKL1−AS1
ZNF512B
SAMD10
PRPF6
LINC00176
SOX18
TCEA2
RGS19
OPRL1
C20orf201
NPBWR2
MYT1
PCMTD2
LINC00266−1

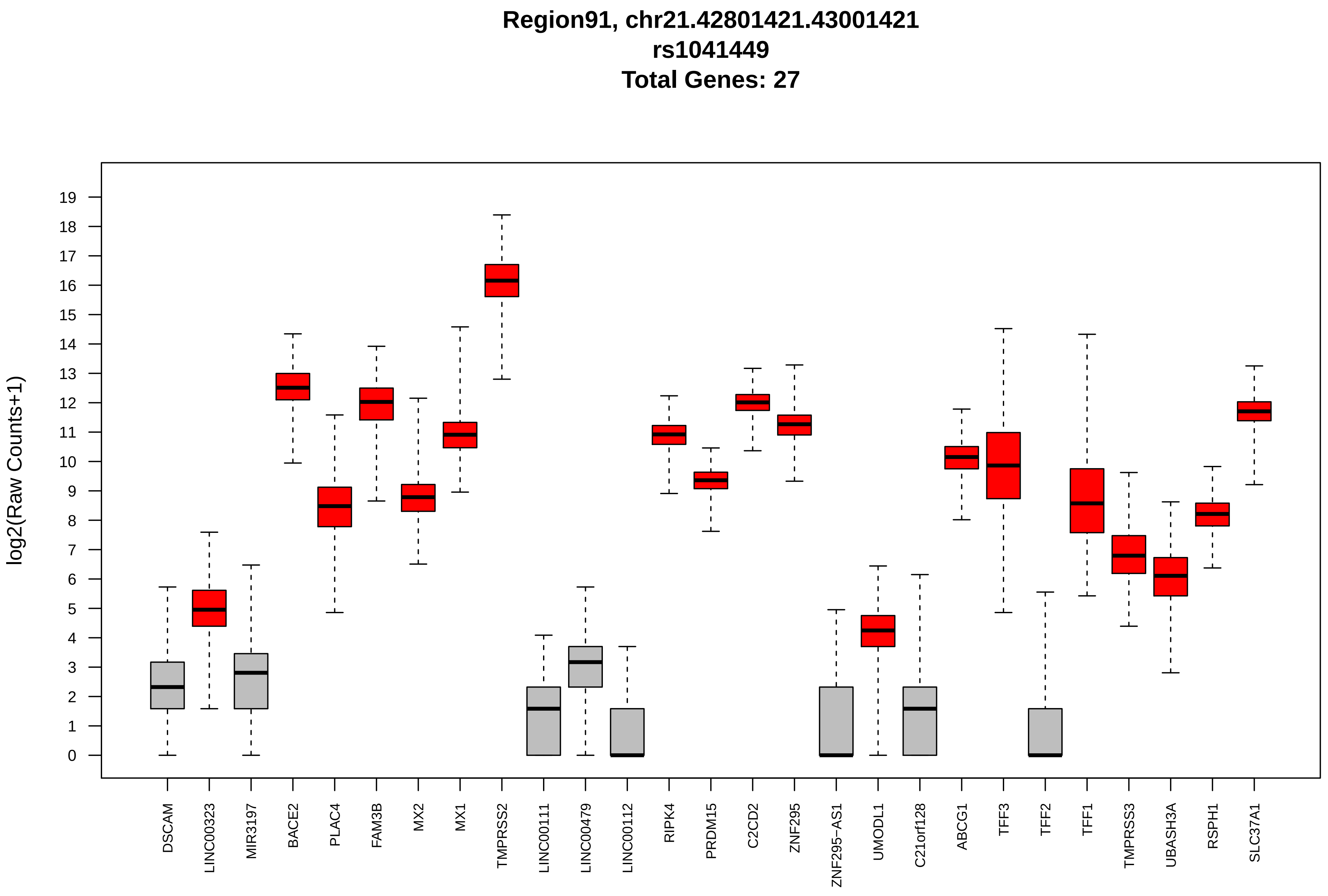

Region91, chr21.42801421.43001421
rs1041449
Total Genes: 27
log2(Raw Counts+1)
0
1
2
3
4
5
6
7
8
9
10
11
12
13
14
15
16
17
18
19
DSCAM
LINC00323
MIR3197
BACE2
PLAC4
FAM3B
MX2
MX1
TMPRSS2
LINC00111
LINC00479
LINC00112
RIPK4
PRDM15
C2CD2
ZNF295
ZNF295−AS1
UMODL1
C21orf128
ABCG1
TFF3
TFF2
TFF1
TMPRSS3
UBASH3A
RSPH1
SLC37A1

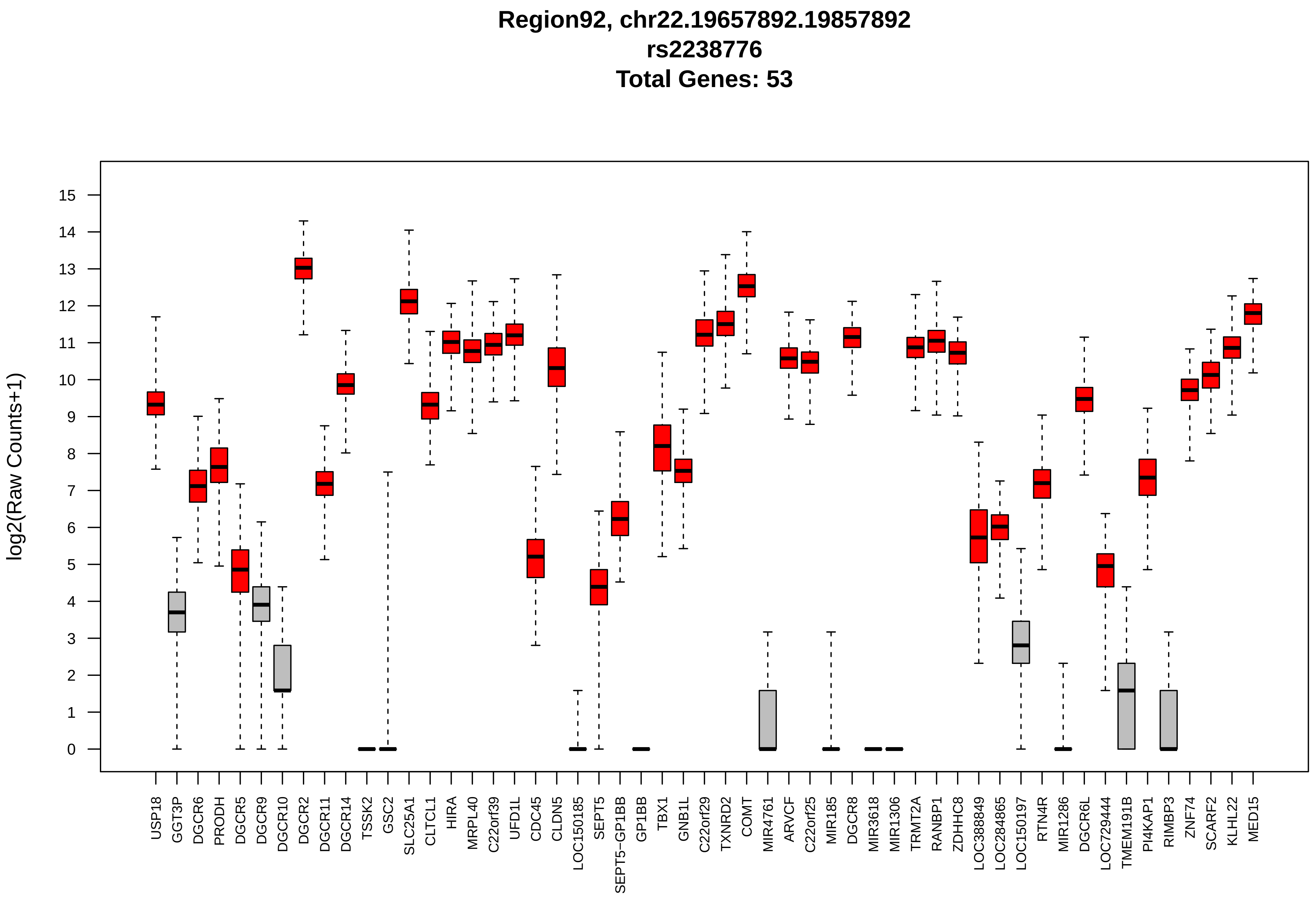
Region92, chr22.19657892.19857892
rs2238776
Total Genes: 53
log2(Raw Counts+1)
0
1
2
3
4
5
6
7
8
9
10
11
12
13
14
15
USP18
GGT3P
DGCR6
PRODH
DGCR5
DGCR9
DGCR10
DGCR2
DGCR11
DGCR14
TSSK2
GSC2
SLC25A1
CLTCL1
HIRA
MRPL40
C22orf39
UFD1L
CDC45
CLDN5
LOC150185
SEPT5
SEPT5−GP1BB
GP1BB
TBX1
GNB1L
C22orf29
TXNRD2
COMT
MIR4761
ARVCF
C22orf25
MIR185
DGCR8
MIR3618
MIR1306
TRMT2A
RANBP1
ZDHHC8
LOC388849
LOC284865
LOC150197
RTN4R
MIR1286
DGCR6L
LOC729444
TMEM191B
PI4KAP1
RIMBP3
ZNF74
SCARF2
KLHL22
MED15

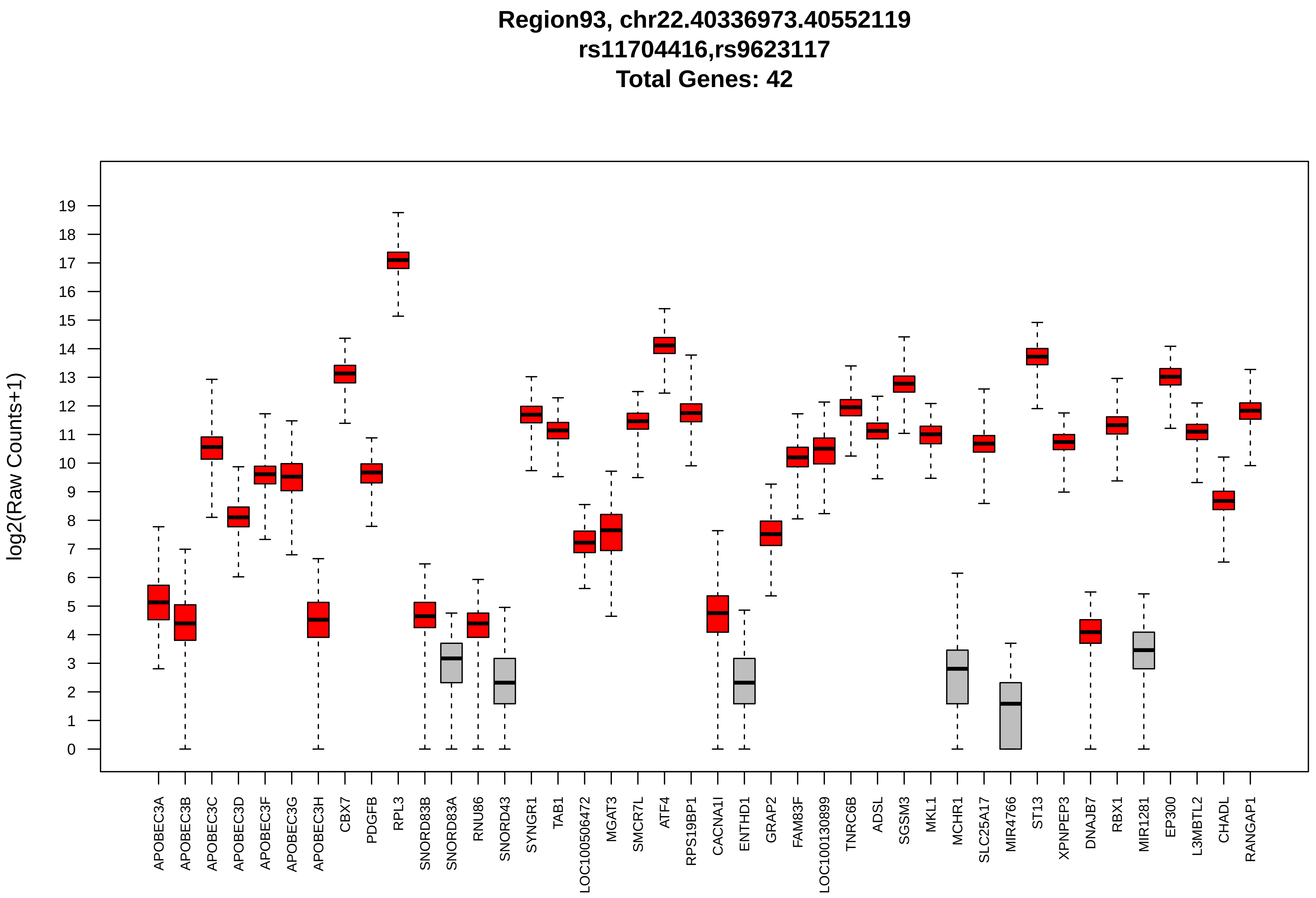
Region93, chr22.40336973.40552119
rs11704416,rs9623117
Total Genes: 42
log2(Raw Counts+1)
0
1
2
3
4
5
6
7
8
9
10
11
12
13
14
15
16
17
18
19
APOBEC3A
APOBEC3B
APOBEC3C
APOBEC3D
APOBEC3F
APOBEC3G
APOBEC3H
CBX7
PDGFB
RPL3
SNORD83B
SNORD83A
RNU86
SNORD43
SYNGR1
TAB1
LOC100506472
MGAT3
SMCR7L
ATF4
RPS19BP1
CACNA1I
ENTHD1
GRAP2
FAM83F
LOC100130899
TNRC6B
ADSL
SGSM3
MKL1
MCHR1
SLC25A17
MIR4766
ST13
XPNPEP3
DNAJB7
RBX1
MIR1281
EP300
L3MBTL2
CHADL
RANGAP1

Region94, chr22.43400212.43618275
rs5759167,rs742134
Total Genes: 37

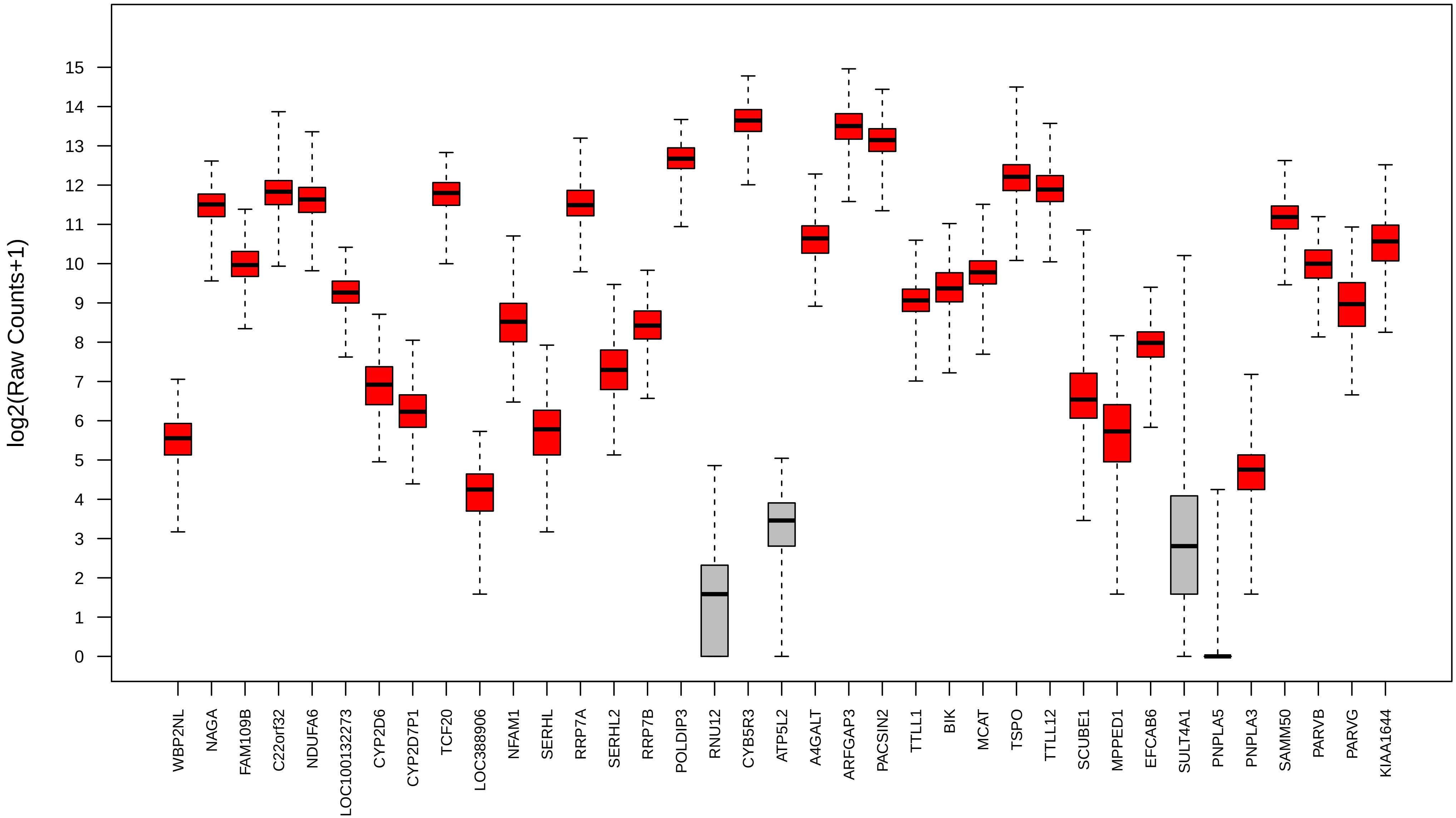

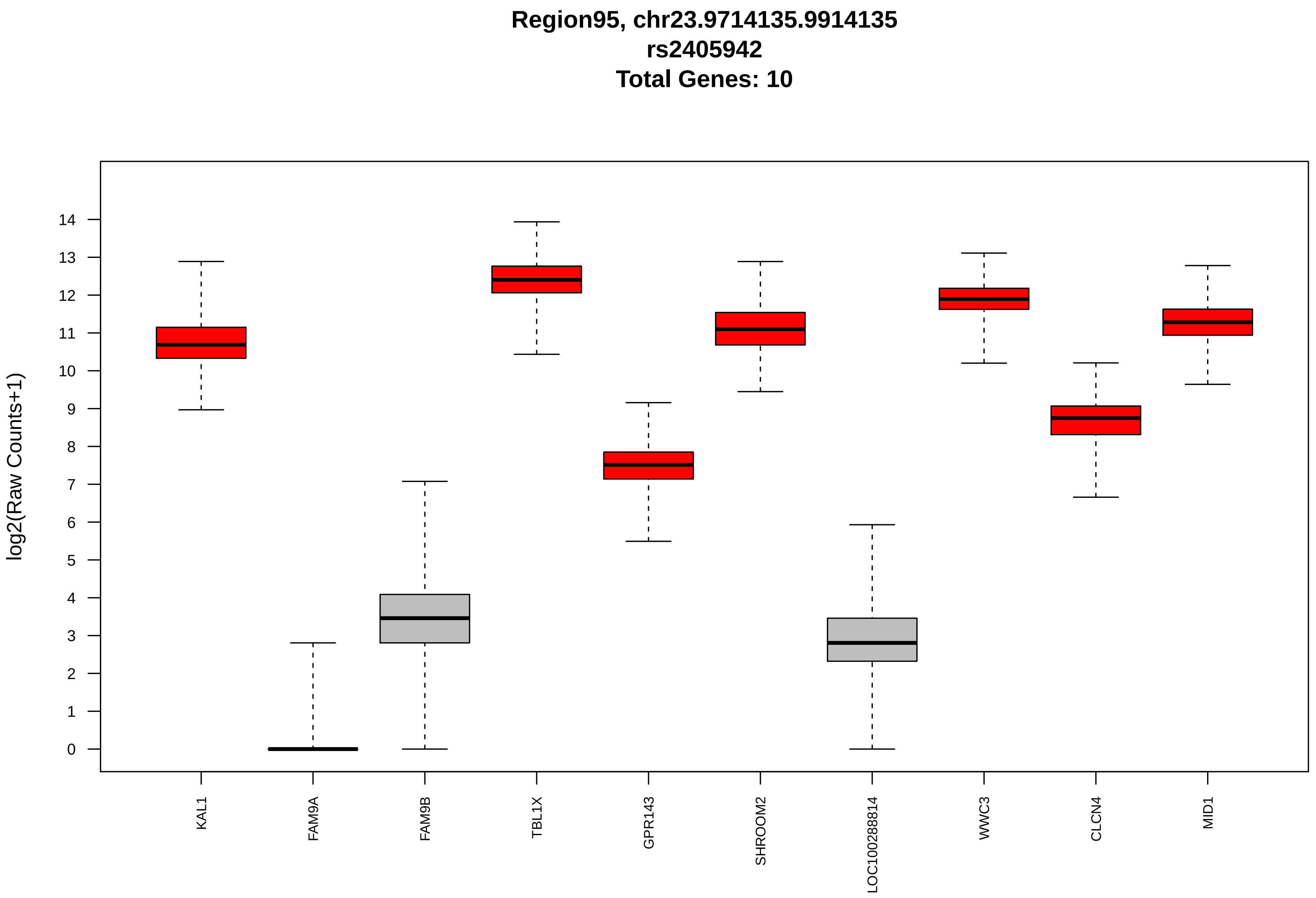
Region95, chr23.9714135.9914135
rs2405942
Total Genes: 10
log2(Raw Counts+1)
14
13
12
11
10
9
8
7
6
5
4
3
2
1
0
KAL1
FAM9A
FAM9B
TBL1X
GPR143
SHROOM2
LOC100288814
WWC3
CLCN4
MID1

Region96, chr23.51110057.51341672
rs1327301,rs5945572,rs5945619
Total Genes: 21

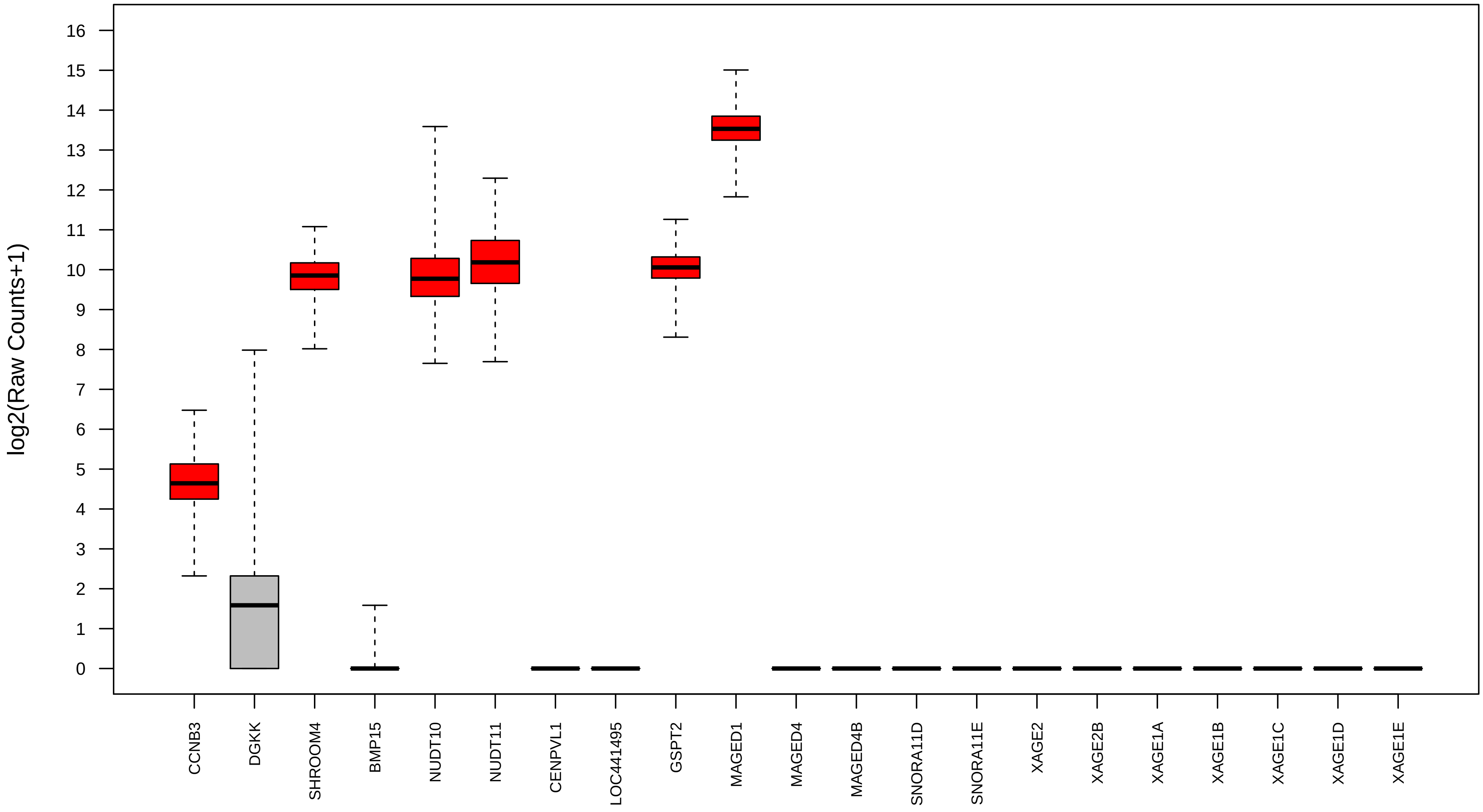

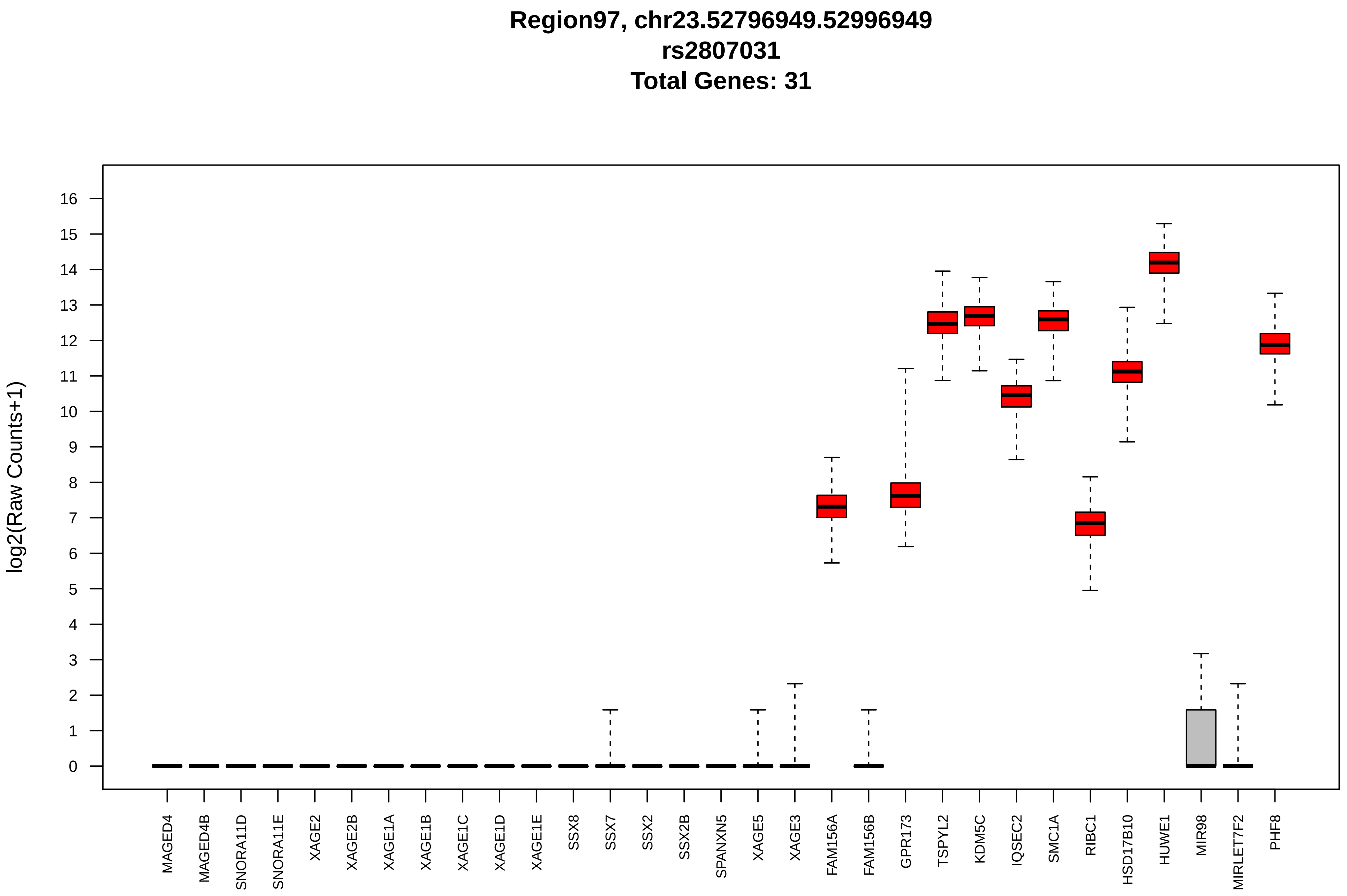
Region97, chr23.52796949.52996949
rs2807031
Total Genes: 31
log2(Raw Counts+1)
0
1
2
3
4
5
6
7
8
9
10
11
12
13
14
15
16
MAGED4
MAGED4B
SNORA11D
SNORA11E
XAGE2
XAGE2B
XAGE1A
XAGE1B
XAGE1C
XAGE1D
XAGE1E
SSX8
SSX7
SSX2
SSX2B
SPANXN5
XAGE5
XAGE3
FAM156A
FAM156B
GPR173
TSPYL2
KDM5C
IQSEC2
SMC1A
RIBC1
HSD17B10
HUWE1
MIR98
MIRLET7F2
PHF8

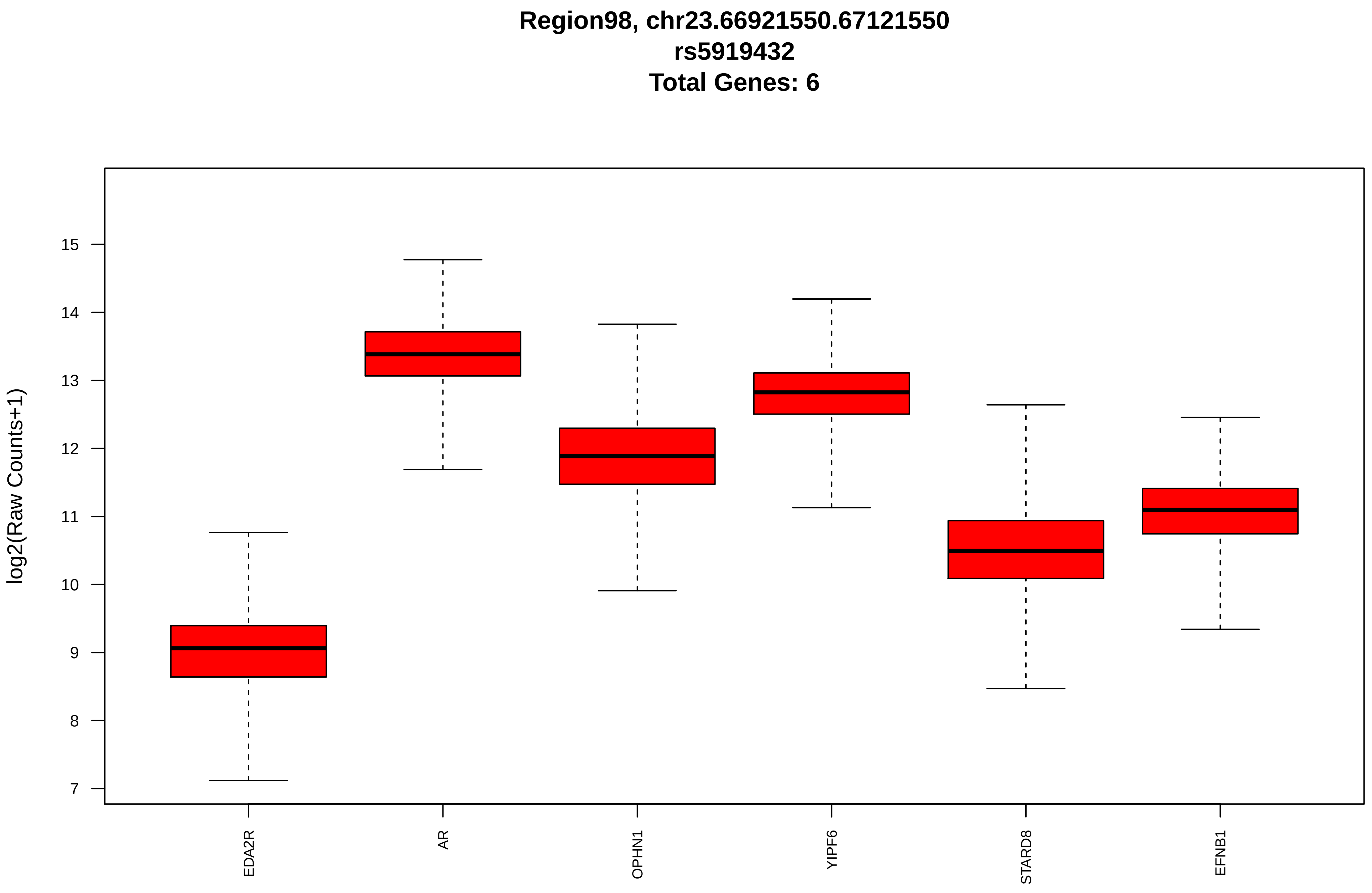
Region98, chr23.66921550.67121550
rs5919432
Total Genes: 6
log2(Raw Counts+1)
15
14
13
12
11
10
9
8
7
EDA2R
AR
OPHN1
YIPF6
STARD8
EFNB1

Region99, chr23.70039850.70239850
rs6625711
Total Genes: 37

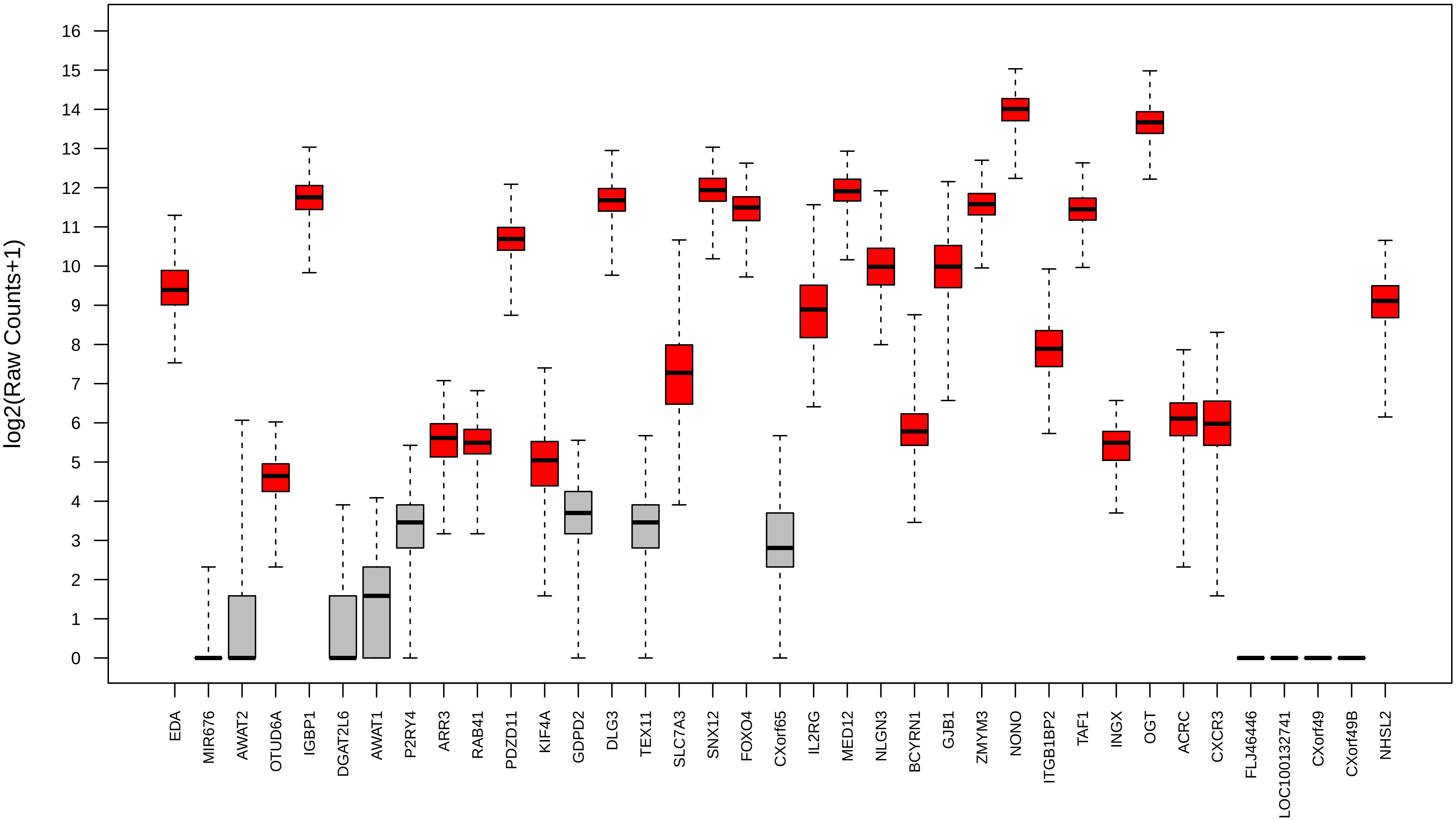

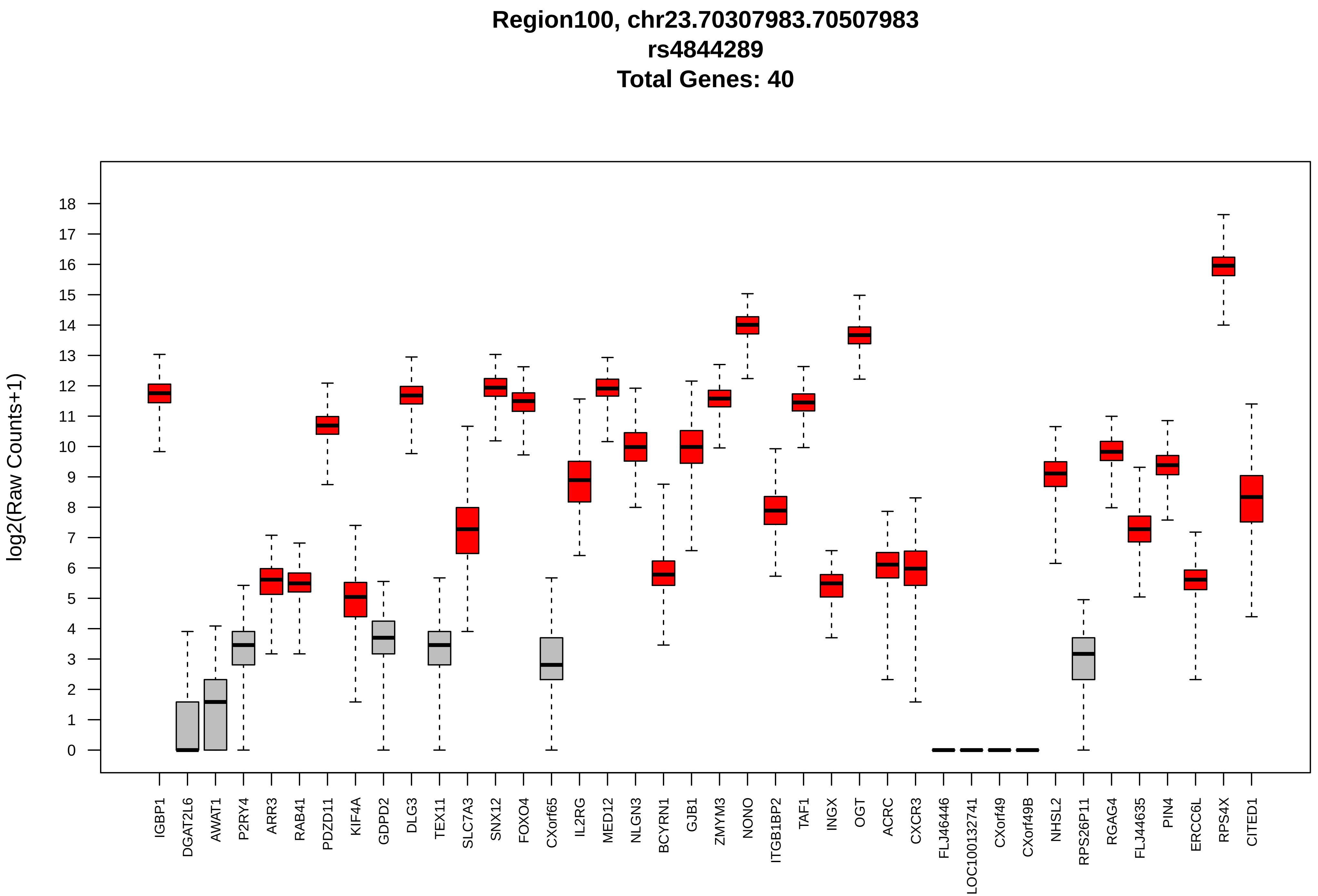

Region100, chr23.70307983.70507983
rs4844289
Total Genes: 40
log2(Raw Counts+1)
0
1
2
3
4
5
6
7
8
9
10
11
12
13
14
15
16
17
18
IGBP1
DGAT2L6
AWAT1
P2RY4
ARR3
RAB41
PDZD11
KIF4A
GDPD2
DLG3
TEX11
SLC7A3
SNX12
FOXO4
CXorf65
IL2RG
MED12
NLGN3
BCYRN1
GJB1
ZMYM3
NONO
ITGB1BP2
TAF1
INGX
OGT
ACRC
CXCR3
FLJ46446
LOC100132741
CXorf49
CXorf49B
NHSL2
RPS26P11
RGAG4
FLJ44635
PIN4
ERCC6L
RPS4X
CITED1

Supplementary Figure 2 - Regional association plots for each of the genes placed in Group 1

Regional association plots display eQTL results for the target gene listed in the title and all SNPs in the target gene region. All PC risk-SNPs in the region are listed in the second line of the title. The x-axis shows the chromosomal position of the SNPs (with expressed genes in the region displayed below) and the y-axis is the –log10(p-value) obtained by regressing normalized expression levels for the target gene on the number of minor alleles of each SNP genotype adjusted for histologic characteristics and 14 expression principal components. The PC risk-SNP position is indicated by a dotted red vertical line with the eQTL result displayed as a diamond. Colored points show all Bonferroni significant results with the color defined by LD between the SNP and the PC risk-SNP (LD r^2>0.5 red, between 0.2 – 0.5 green, and ≤0.2 blue). If the region contains multiple PC risk-SNPs, the points are colored based on LD with the PC risk-SNP that is in highest LD with the eQTL peak associated SNP. The eQTL result for this PC risk-SNP is displayed as diamond, the data points for all of the other PC risk-SNPs are displayed as an open circle.

The table below the LD plot shows results for the peak-SNP (Result Type = Peak) and the most upstream and downstream SNPs (Result Type = Flanking) surpassing the Bonferroni significance threshold (P < 1.96E-07 for primary analysis and P < 3.02E-08 for second stage) as well as the top result for each PC risk SNP (Result Type = PC Risk) and, if a SNP in LD (r^2 > 0.5) with the PC risk SNP has a stronger association than the PC risk SNP (i.e., lower p-value), then the LD SNP with the strongest association is also presented (Result Type = MaxLD). Finally, if all of these SNPs have a single direction of effect, e.g., all have positive beta coefficients thus increasing gene expression, and if any Bonferroni significant results were

observed in the opposite direction, then the strongest result among the opposite direction SNPs is also presented (Result Type = ReverseBeta).

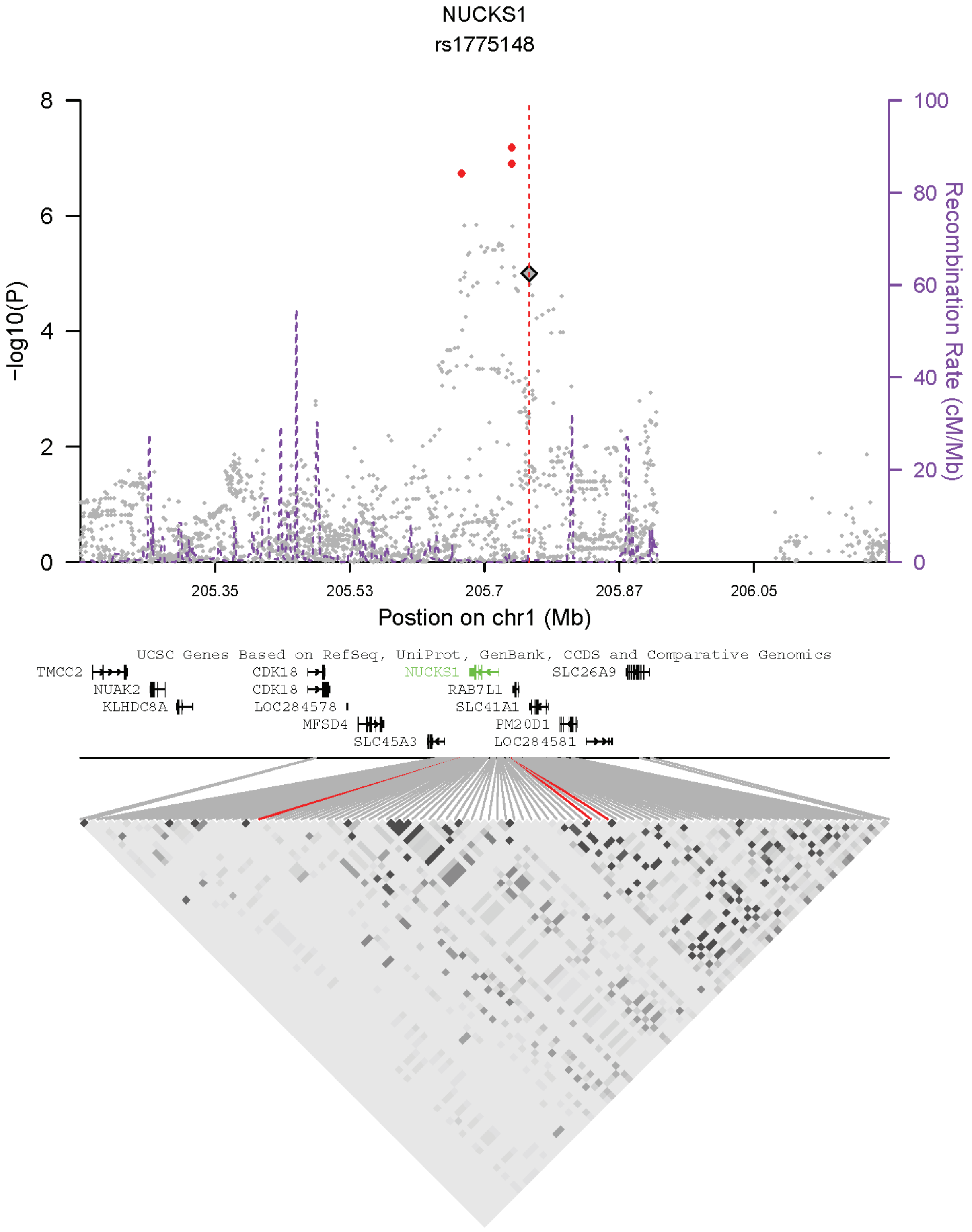

rsID	pos	minor	major	MAF	Beta	p.value	adj.pvalue	rs1775148	imputed	Result Type
rs11240554	205671644	T	C	0.34	0.027	1.84e-07	0.425	0.627	1	Flanking
rs2014202	205735612	A	G	0.38	0.027	6.73e-08	–	0.773	1	Peak
rs7522056	205735891	A	G	0.38	0.027	1.27e-07	0.422	0.782	0	Flanking
rs1775148	205757824	C	T	0.43	0.023	1.00e-05	0.632	1.000	1	PC Risk

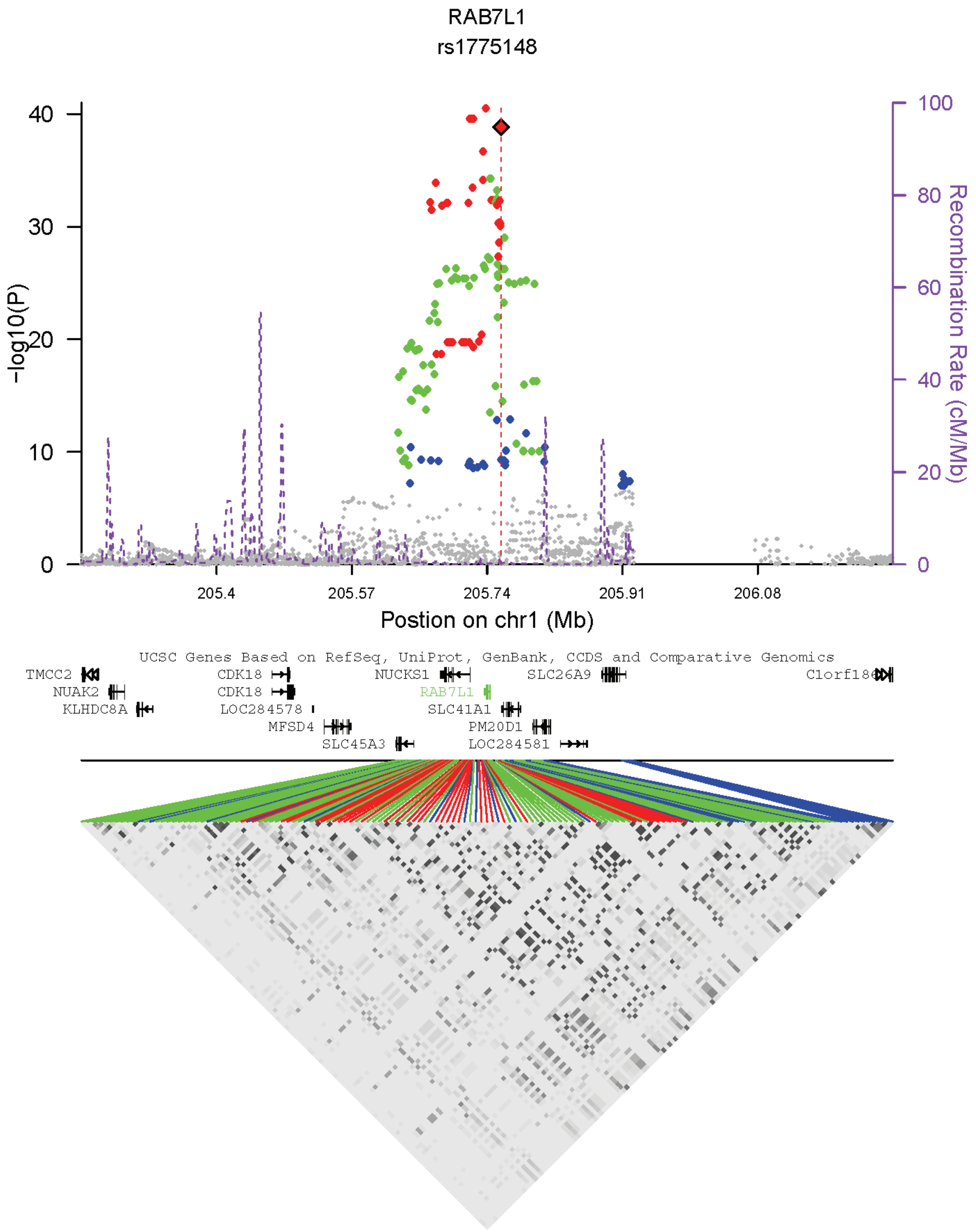

rsID	pos	minor	major	MAF	Beta	p.value	adj.pvalue	rs1775148	imputed	Result Type
rs3761916	205630893	G	A	0.29	−0.123	2.14e−12	9.33e−02	0.287	0	Flanking
rs708723	205739266	C	T	0.49	−0.193	3.29e−41	−	0.678	0	Peak
rs1775148	205757824	C	T	0.43	−0.196	1.49e−39	2.33e−05	1.000	1	PC Risk
rs7415921	205910883	G	T	0.46	0.087	2.77e−08	8.20e−01	<0.2	0	Flanking

NOL10
rs9287719, rs11902236

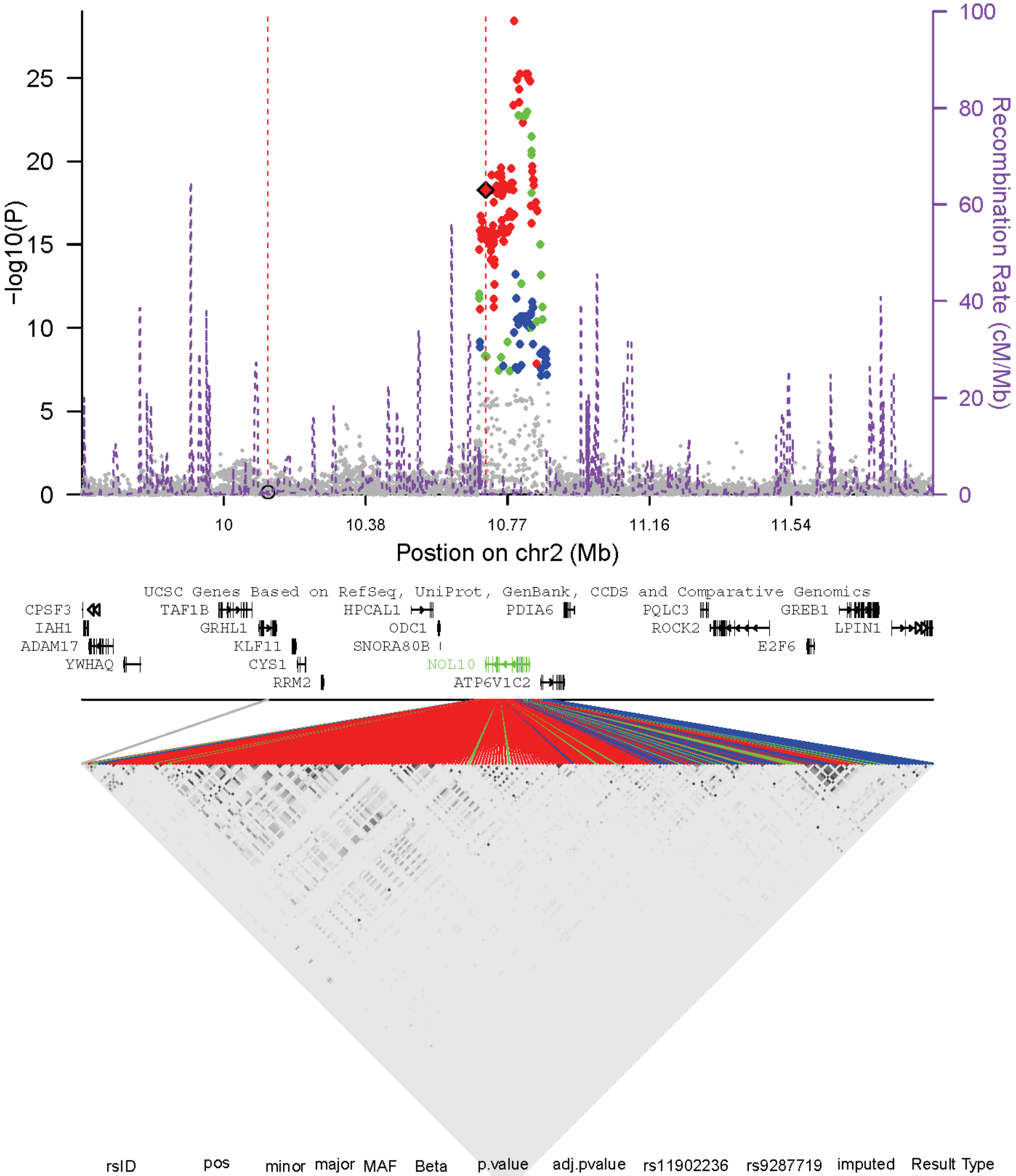

rsID	pos	minor	major	MAF	Beta	p.value	adj.pvalue	rs11902236	rs9287719	imputed	Result Type
rs7581701	10093908	T	C	0.42	0.005	3.91e−01	0.413	0.520	<0.2	0	MaxLD
rs11902236	10117868	T	C	0.29	−0.003	6.70e−01	0.918	1.000	<0.2	0	PC Risk
rs3929613	10694242	A	G	0.40	0.052	2.17e−15	0.703	<0.2	0.551	1	Flanking
rs9287719	10710730	C	T	0.48	−0.053	5.32e−19	0.958	<0.2	1.000	1	PC Risk
rs7567304	10790029	T	C	0.42	0.067	3.70e−29	–	<0.2	0.629	1	Peak
rs4669615	10878511	T	G	0.21	−0.045	1.57e−08	0.153	<0.2	<0.2	0	Flanking

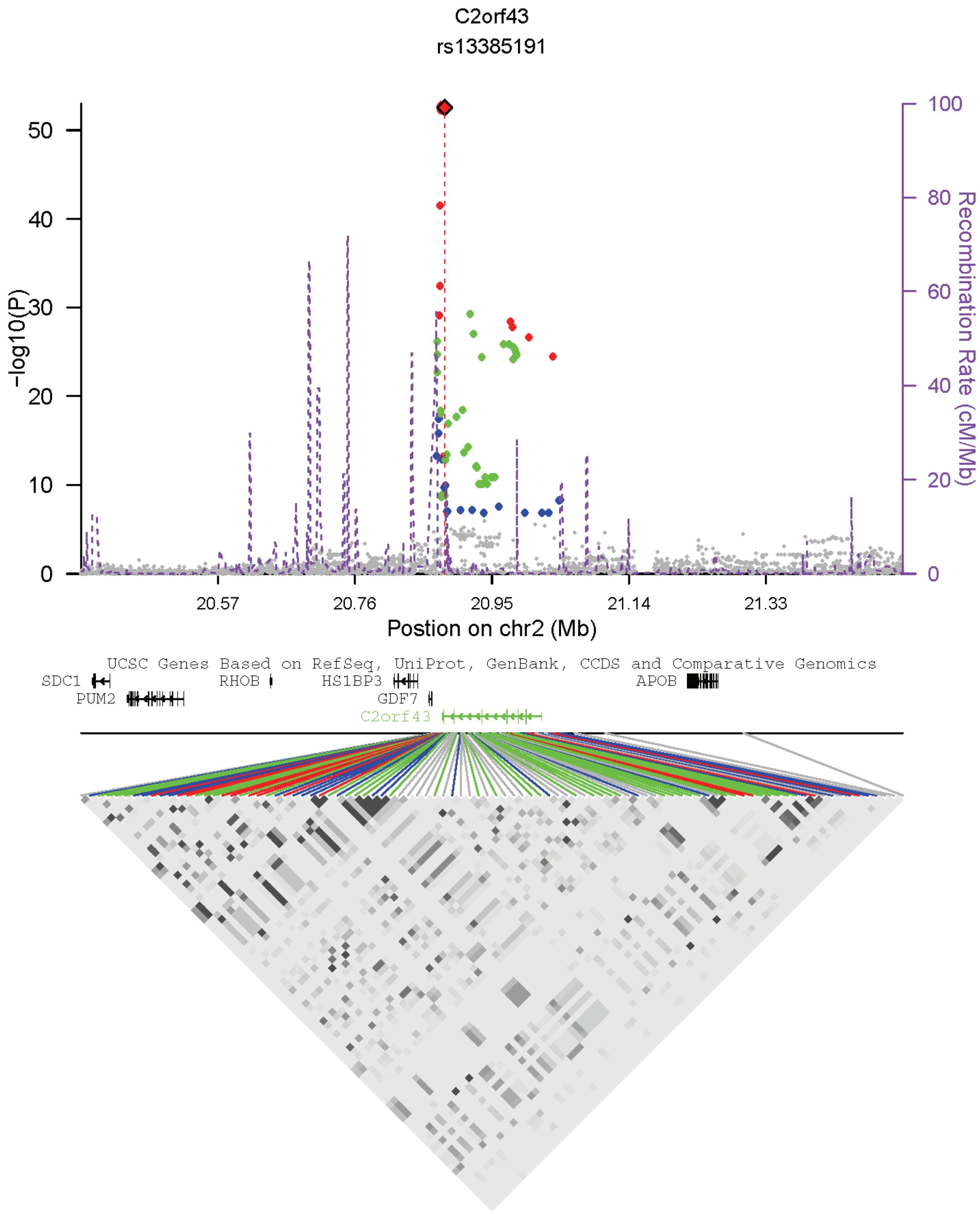

rsID	pos	minor	major	MAF	Beta	p.value	adj.pvalue	rs13385191	imputed	Result Type
rs72784331	20877436	A	C	0.14	0.223	5.95e−14	1.32e−09	<0.2	0	Flanking
rs10182643	20884586	C	G	0.24	−0.364	1.54e−53	–	0.992	1	Peak
rs13385191	20888265	G	A	0.24	−0.363	2.84e−53	9.08e−01	1.000	1	PC Risk
rs12476956	21049129	T	C	0.43	0.130	4.87e−09	9.60e−01	<0.2	1	Flanking

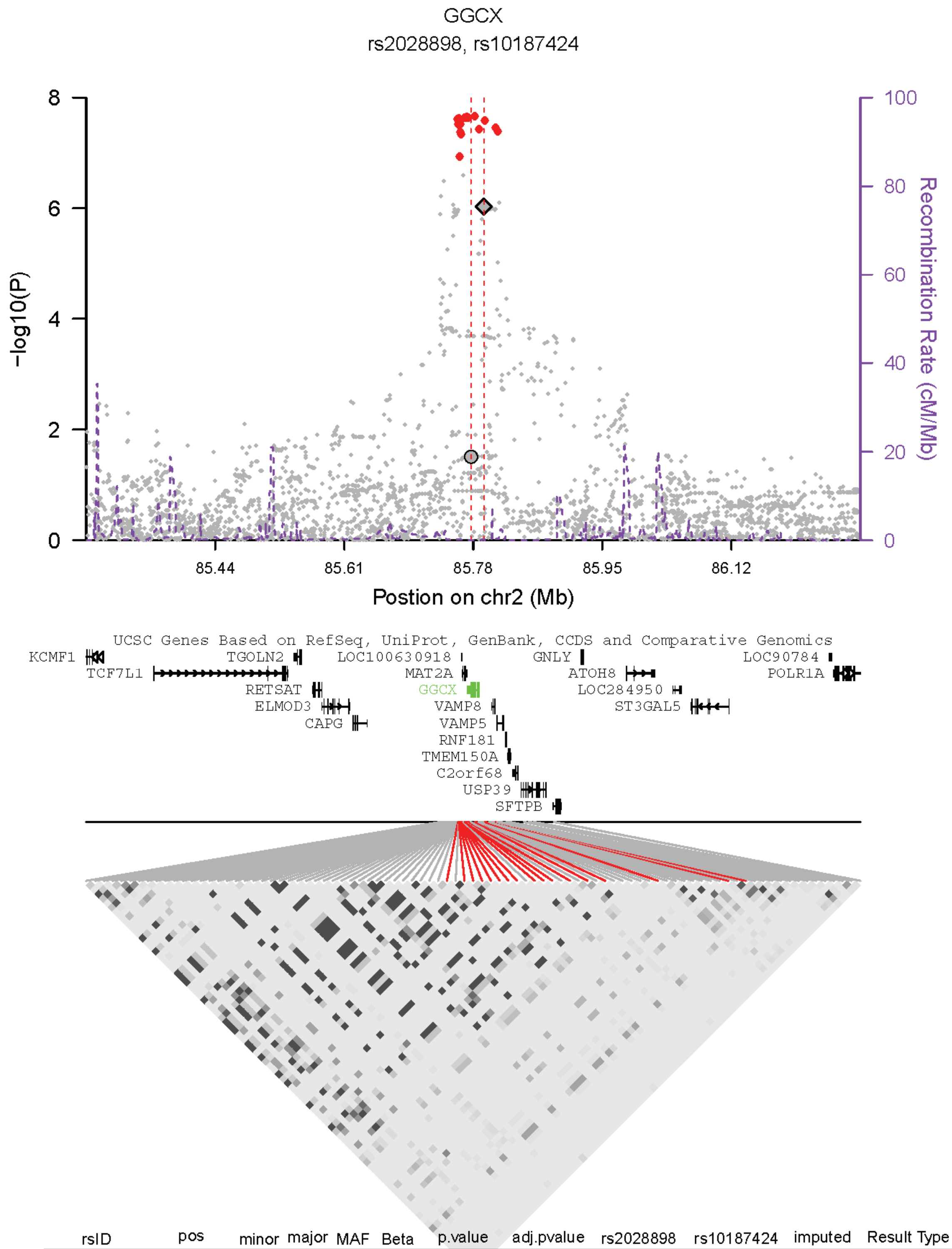

rsID	pos	minor	major	MAF	Beta	p.value	adj.pvalue	rs2028898	rs10187424	imputed	Result Type
rs6735152	85754450	C	A	0.41	0.040	8.76e−07	0.7092	0.513	0.964	1	MaxLD
rs6731005	85760395	T	C	0.45	0.045	2.47e−08	0.7690	0.444	0.836	1	Flanking
rs2028898	85777270	A	G	0.27	0.020	3.06e−02	0.0284	1.000	0.536	1	PC Risk
rs6738645	85783128	G	T	0.45	0.045	2.20e−08	−	0.461	0.855	1	Peak
rs10187424	85794297	C	T	0.41	0.040	9.24e−07	0.5112	0.536	1.000	0	PC Risk
rs55971080	85812746	C	T	0.45	0.044	4.08e−08	0.9761	0.454	0.836	1	Flanking

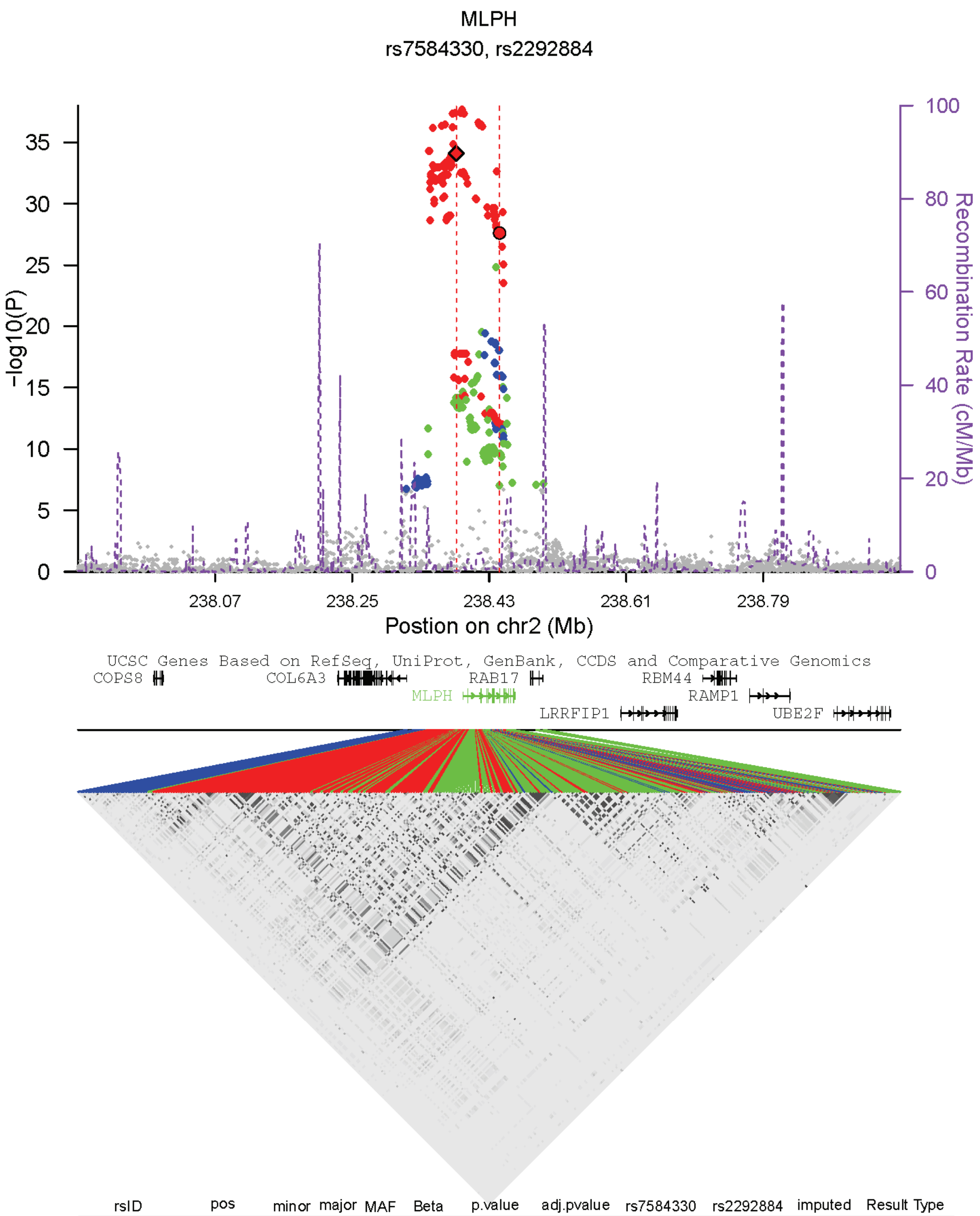

rsID	pos	minor	major	MAF	Beta	p.value	adj.pvalue	rs7584330	rs2292884	imputed	Result Type
rs57027839	238337593	G	A	0.07	−0.159	2.80e−08	0.05746	<0.2	<0.2	0	Flanking
rs7584330	238387228	G	A	0.28	−0.188	8.10e−35	0.50733	1.000	0.690	0	PC Risk
rs58057291	238395479	A	T	0.25	−0.203	2.22e−38	−	0.890	0.736	1	Peak
rs2292884	238443226	G	A	0.27	−0.167	2.24e−28	0.80923	0.690	1.000	0	PC Risk
rs867306	238454154	A	C	0.15	−0.131	4.66e−11	0.00992	0.402	0.432	0	Flanking

CHMP2B

rs7629490, rs2055109, rs2660753, rs9284813, rs17181170

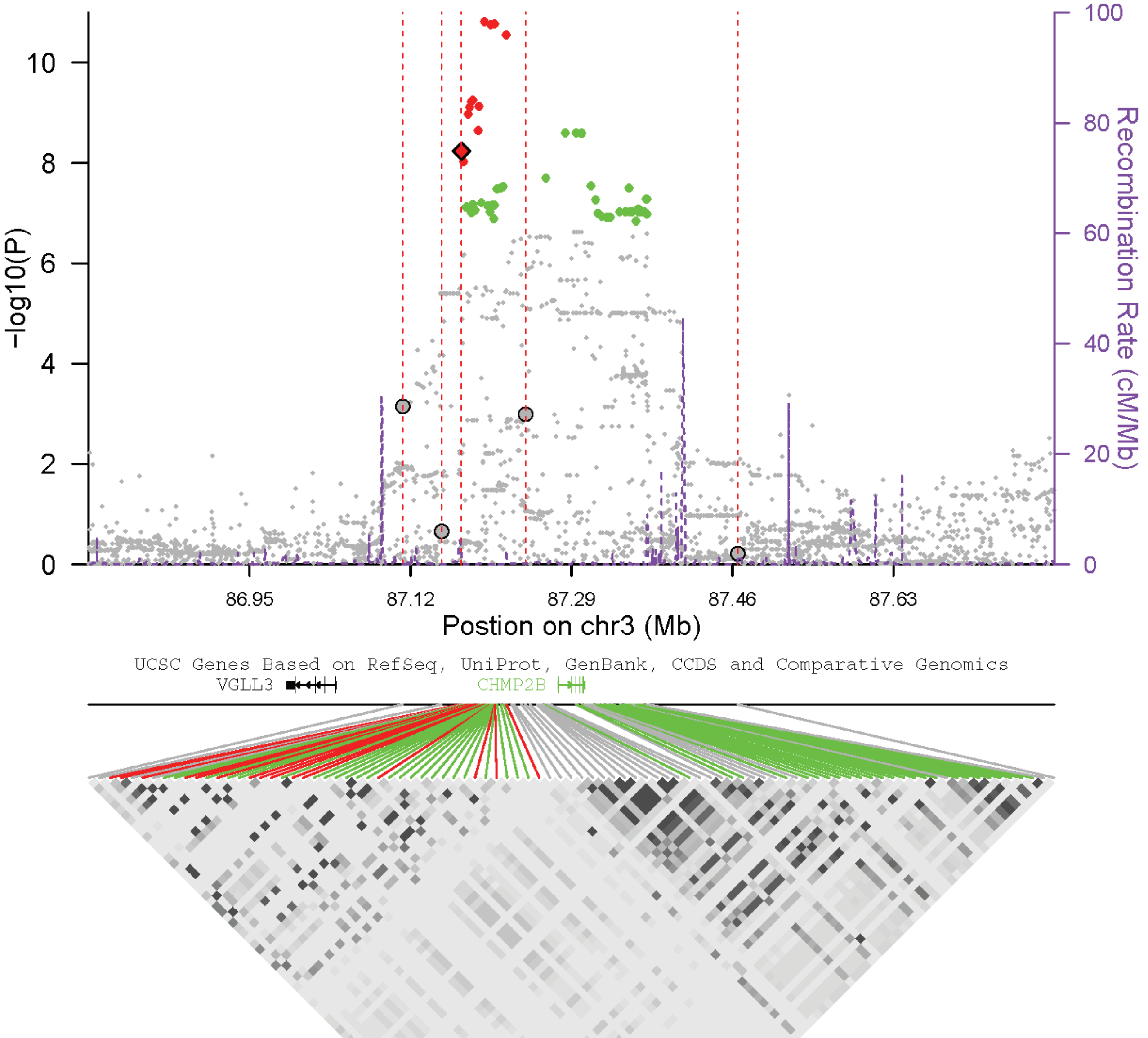

rsID	pos	minor	major	MAF	Beta	p.value	adj.pvalue	rs2660753	rs9284813	rs17181170	rs7629490	rs2055109	imputed	Result Type
rs2575748	87109709	G	A	0.17	0.026	9.99e−03	0.0240	0.614	0.569	<0.2	<0.2	<0.2	1	MaxLD
rs2660753	87110674	T	C	0.11	0.040	7.03e−04	0.0463	1.000	0.243	<0.2	<0.2	<0.2	0	PC Risk
rs1436642	87118806	T	C	0.11	0.041	5.95e−04	0.0468	0.992	0.247	<0.2	<0.2	<0.2	1	MaxLD
rs1865866	87119543	A	G	0.11	0.041	5.95e−04	0.0468	0.992	0.247	<0.2	<0.2	<0.2	0	MaxLD
rs9284813	87152169	G	A	0.14	0.013	2.16e−01	0.4088	0.243	1.000	<0.2	<0.2	<0.2	0	PC Risk
rs7642887	87172632	C	T	0.49	−0.044	5.55e−09	0.6900	<0.2	<0.2	1.000	0.204	<0.2	1	Flanking
rs17181170	87173324	A	G	0.49	−0.044	5.80e−09	0.7005	<0.2	<0.2	1.000	0.204	<0.2	0	PC Risk
rs9856752	87198389	A	G	0.45	0.051	1.52e−11	–	<0.2	<0.2	0.702	0.347	<0.2	1	Peak
rs7629490	87241497	T	C	0.36	0.025	1.01e−03	0.4748	<0.2	<0.2	0.204	1.000	<0.2	0	PC Risk
rs17024046	87263497	T	C	0.37	0.042	2.02e−08	0.1583	<0.2	<0.2	0.324	0.714	<0.2	0	MaxLD
rs11127975	87311731	C	T	0.40	0.042	2.88e−08	0.3773	<0.2	<0.2	0.371	<0.2	<0.2	1	Flanking
rs2055109	87467332	G	A	0.25	−0.005	6.03e−01	0.7888	<0.2	<0.2	<0.2	<0.2	1.000	0	PC Risk
rs9840394	87582728	T	G	0.30	−0.018	3.44e−02	0.4246	<0.2	<0.2	<0.2	<0.2	0.611	0	MaxLD
rs9858431	87583001	A	G	0.30	−0.018	3.44e−02	0.4246	<0.2	<0.2	<0.2	<0.2	0.611	1	MaxLD

BMPR1B

rs12500426, rs17021918

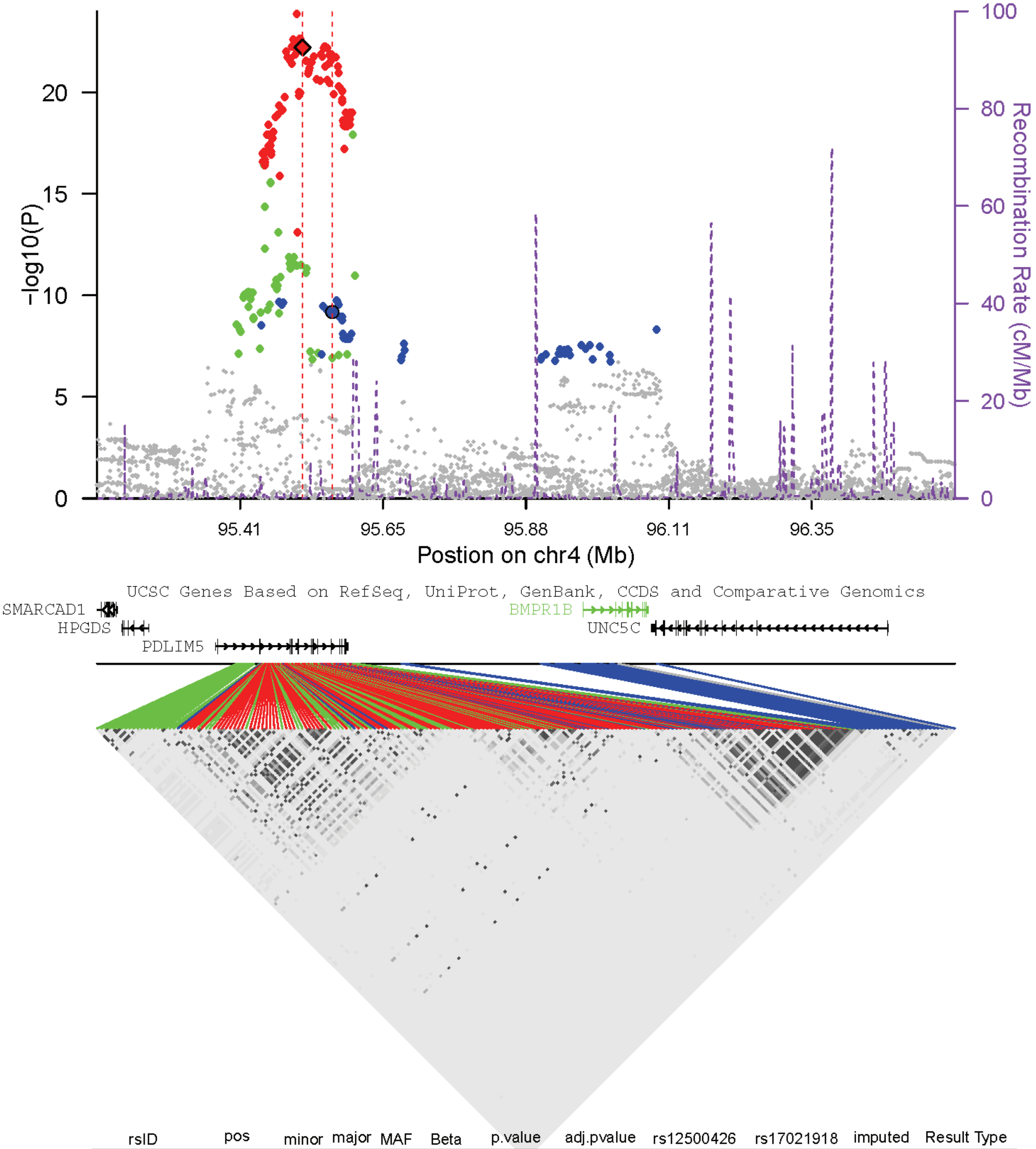

rsID	pos	minor	major	MAF	Beta	p.value	adj.pvalue	rs12500426	rs17021918	imputed	Result Type
rs2438140	95407821	C	T	0.49	−0.109	2.75e−09	5.98e−01	0.240	<0.2	0	Flanking
rs2452597	95493246	G	A	0.28	0.144	1.35e−12	2.09e−02	0.354	0.713	1	MaxLD
rs12639887	95505975	T	C	0.43	−0.182	1.38e−24	–	0.769	0.319	0	Peak
rs12500426	95514609	A	C	0.47	−0.178	6.19e−23	7.59e−02	1.000	<0.2	1	PC Risk
rs17021918	95562877	T	C	0.33	0.120	6.36e−10	2.83e−01	<0.2	1.000	1	PC Risk
rs7693093	96093552	T	C	0.44	0.116	5.05e−09	1.85e−09	<0.2	<0.2	1	Flanking

IRX4

rs2242652, rs7725218, rs2853676, rs13190087, rs12653946

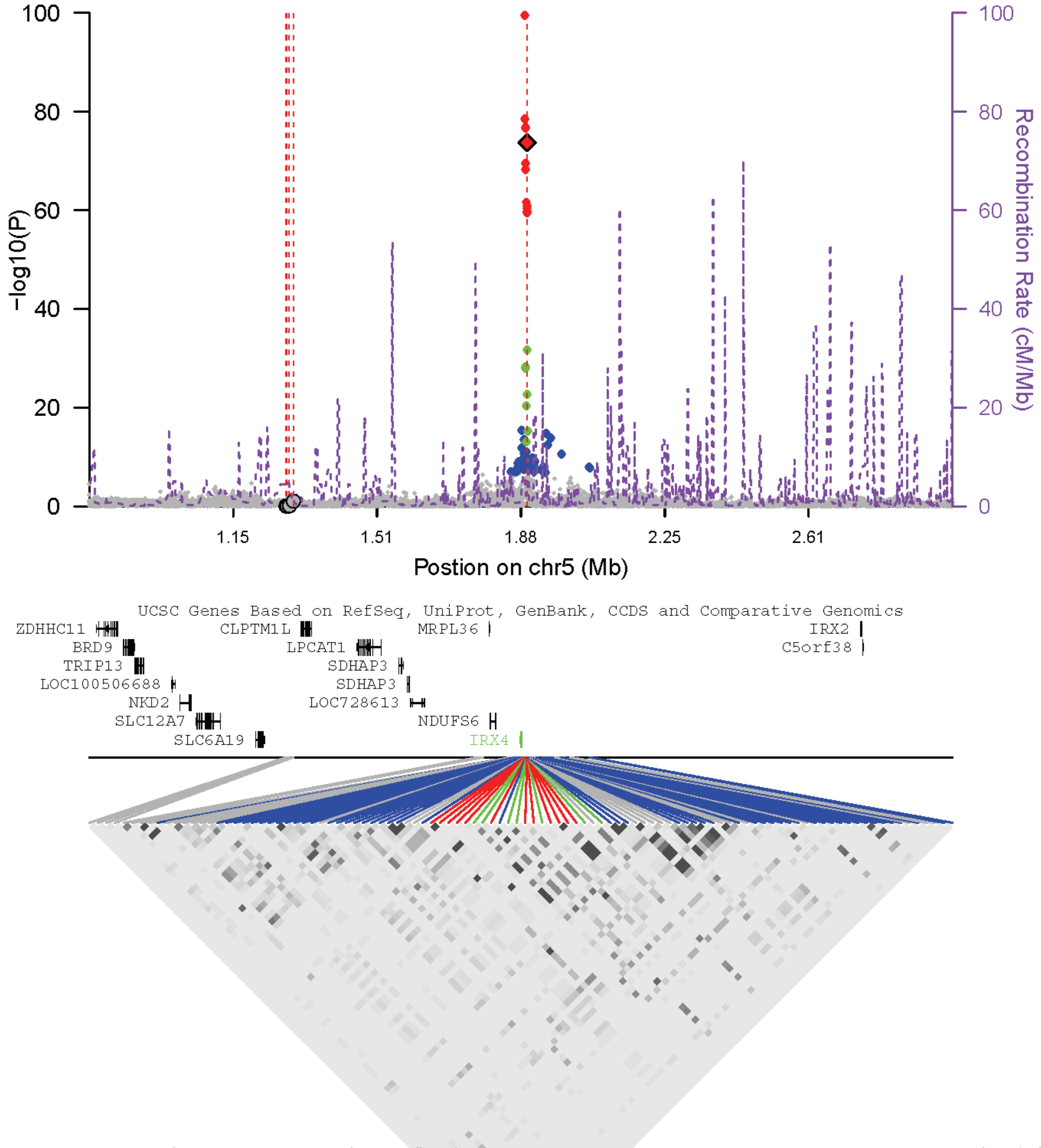

rsID	pos	minor	major	MAF	Beta	p.value	adj.pvalue	rs2242652	rs7725218	rs2853676	rs13190087	rs12653946	imputed	Result Type
rs2242652	1280028	A	G	0.17	−0.033	7.23e−01	5.37e−01	1.000	0.364	<0.2	<0.2	<0.2	1	PC Risk
rs4975538	1280830	C	G	0.35	−0.048	5.08e−01	8.44e−01	0.373	0.668	<0.2	<0.2	<0.2	1	MaxLD
rs7725218	1282414	A	G	0.32	−0.014	8.48e−01	8.62e−01	0.364	1.000	<0.2	<0.2	<0.2	0	PC Risk
rs2853676	1288547	T	C	0.24	−0.036	6.45e−01	9.80e−01	<0.2	<0.2	1.000	<0.2	<0.2	0	PC Risk
rs13190087	1298733	C	A	0.04	−0.317	7.44e−02	1.51e−01	<0.2	<0.2	<0.2	1.000	<0.2	1	PC Risk
rs263202	1872594	A	G	0.13	0.562	2.06e−08	1.09e−02	<0.2	<0.2	<0.2	<0.2	<0.2	1	Flanking
rs12655062	1890877	A	G	0.34	−1.177	4.00e−100	–	<0.2	<0.2	<0.2	<0.2	0.566	0	Peak
rs12653946	1895829	T	C	0.42	−1.020	1.88e−74	1.35e−09	<0.2	<0.2	<0.2	<0.2	1.000	0	PC Risk
rs113339853	2056345	A	G	0.09	−0.680	2.18e−08	6.40e−06	<0.2	<0.2	<0.2	<0.2	<0.2	0	Flanking

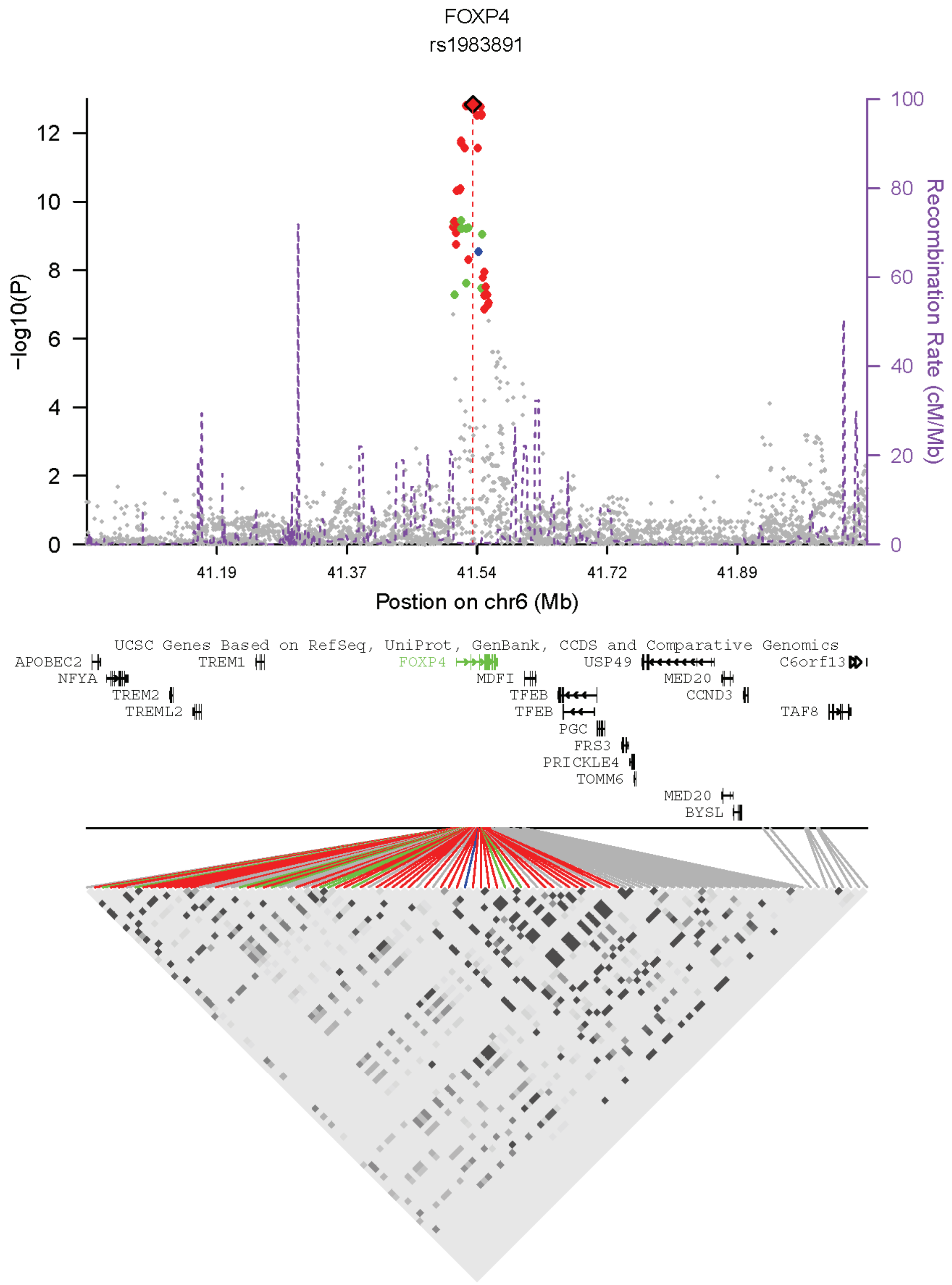

rsID	pos	minor	major	MAF	Beta	p.value	adj.pvalue	rs1983891	imputed	Result Type
rs12203518	41510291	C	G	0.25	−0.069	5.45e−10	0.0803	0.873	1	Flanking
rs9296365	41535379	G	A	0.24	−0.082	1.44e−13	–	1.000	1	Peak
rs1983891	41536427	T	C	0.24	−0.082	1.45e−13	0.9249	1.000	1	PC Risk
rs3800285	41558443	G	A	0.28	−0.057	9.10e−08	0.5740	0.605	1	Flanking

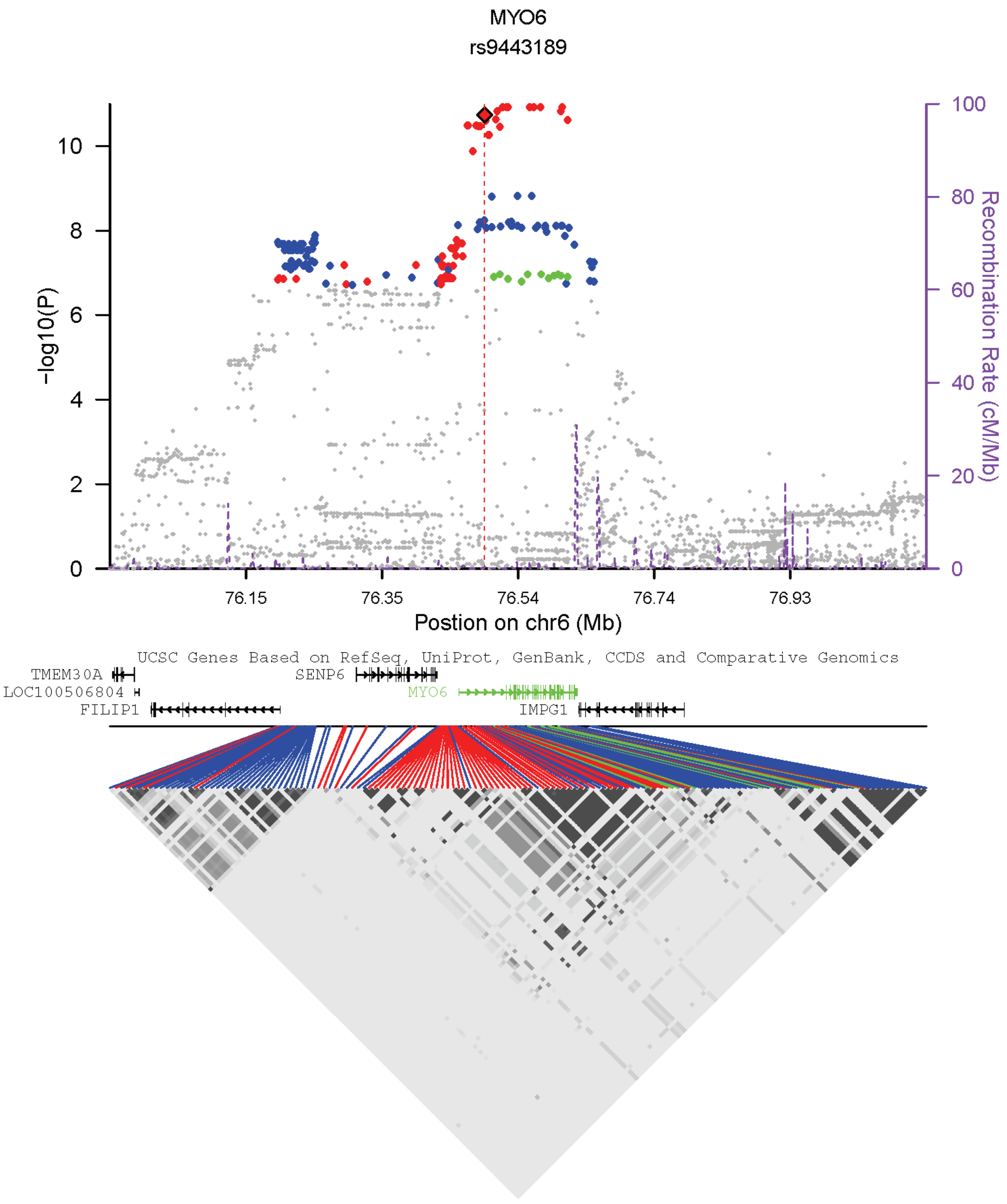

rsID	pos	minor	major	MAF	Beta	p.value	adj.pvalue	rs9443189	imputed	Result Type
rs11752498	76199880	T	A	0.48	−0.056	1.88e−08	0.000377	<0.2	1	Flanking
rs9443189	76495882	G	A	0.16	0.093	1.84e−11	0.906406	1.000	1	PC Risk
rs3798439	76566734	T	G	0.15	0.094	1.23e−11	−	0.968	1	Peak
rs699186	76625079	C	T	0.37	−0.056	2.16e−08	0.000533	<0.2	0	Flanking

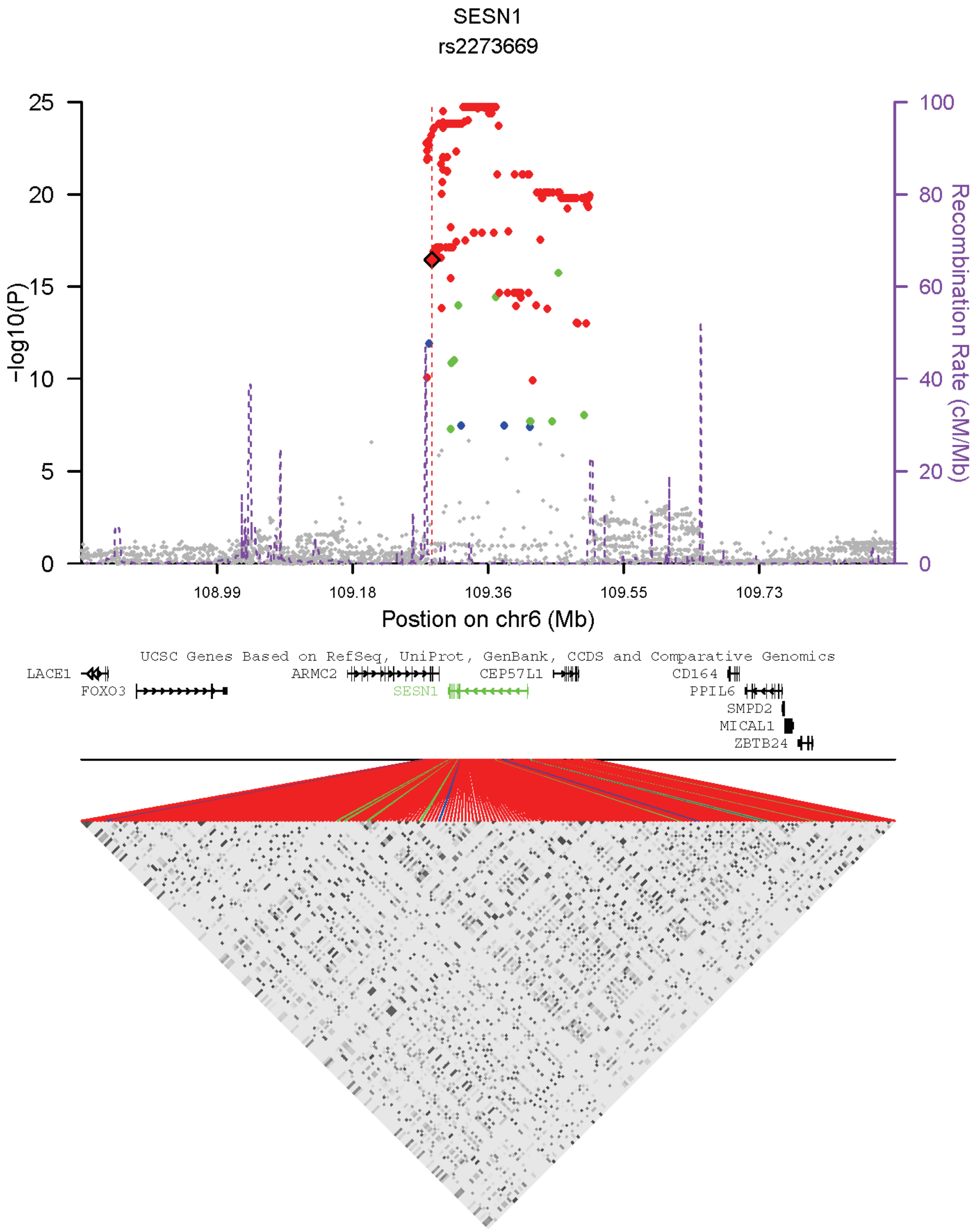

rsID	pos	minor	major	MAF	Beta	p.value	adj.pvalue	rs2273669	imputed	Result Type
rs12193858	109277908	T	A	0.12	0.204	1.71e−23	0.833	0.742	1	Flanking
rs2273669	109285189	G	A	0.16	0.157	3.45e−17	0.234	1.000	0	PC Risk
rs112871784	109362245	T	C	0.13	0.202	1.89e−25	–	0.774	1	Peak
rs59358669	109500192	A	G	0.11	0.199	1.10e−20	0.953	0.594	1	Flanking

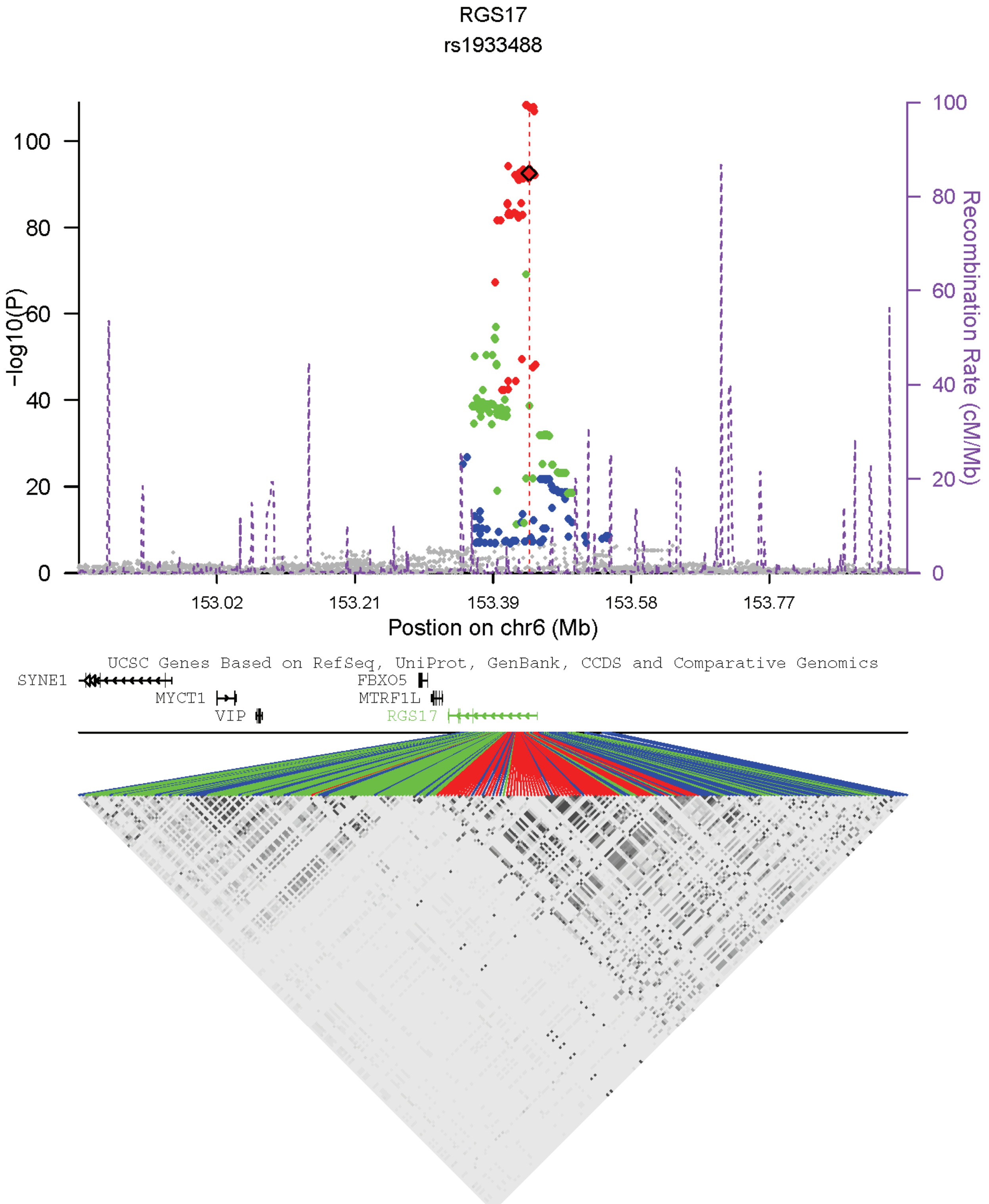

rsID	pos	minor	major	MAF	Beta	p.value	adj.pvalue	rs1933488	imputed	Result Type
rs7774354	153352474	C	A	0.12	−0.684	6.14e−26	4.96e−02	<0.2	0	Flanking
rs1281962	153431376	G	C	0.45	0.597	3.11e−50	1.93e−09	0.640	1	ReverseBeta
rs113645266	153437735	C	T	0.32	−0.837	4.40e−109	−	0.635	1	Peak
rs1933488	153441079	G	A	0.44	−0.738	2.62e−93	2.55e−18	1.000	0	PC Risk
rs315408	153549673	A	G	0.14	−0.378	2.88e−09	3.88e−01	<0.2	0	Flanking

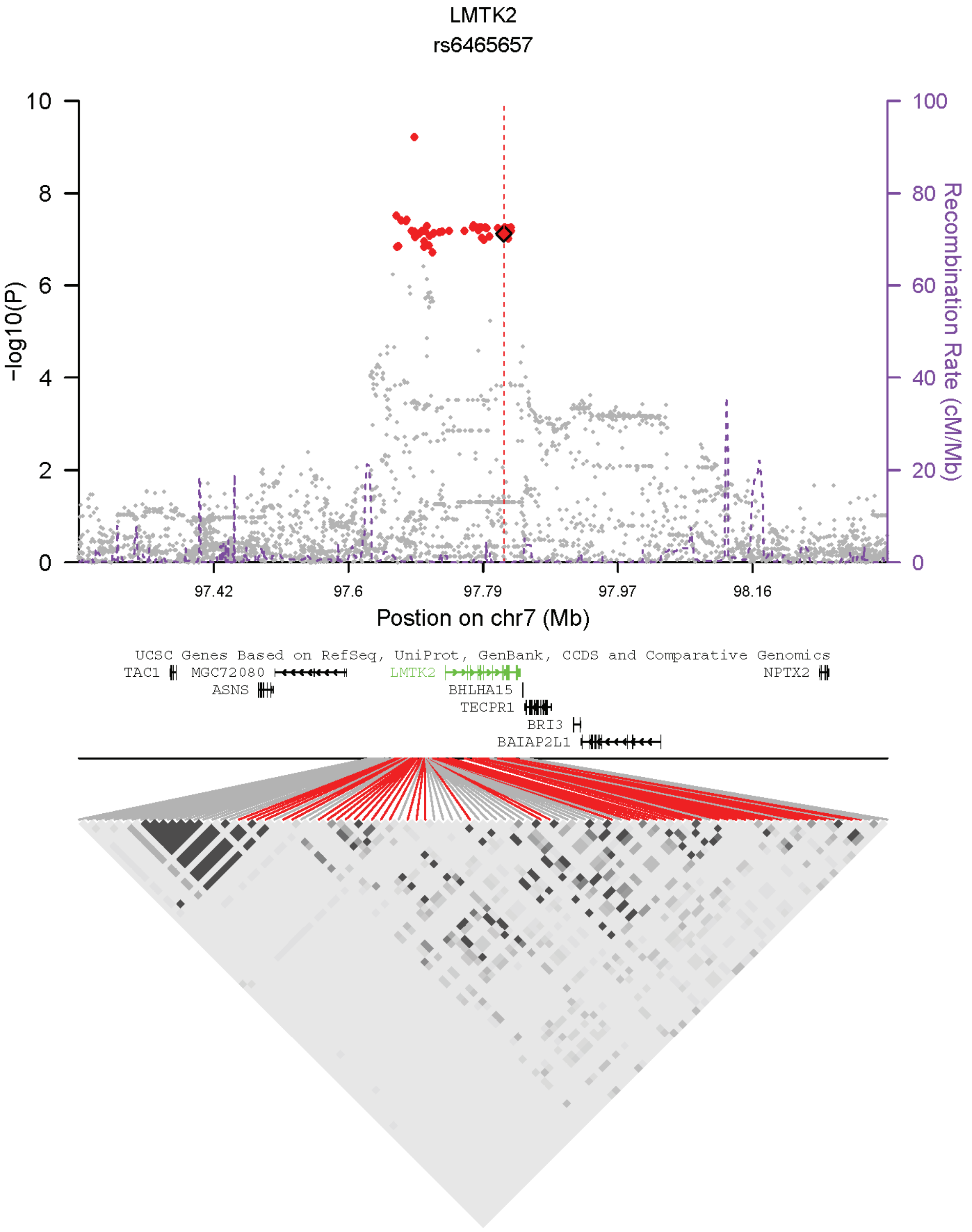

rsID	pos	minor	major	MAF	Beta	p.value	adj.pvalue	rs6465657	imputed	Result Type
rs847577	97694858	A	T	0.39	0.057	6.08e−10	−	0.677	1	Peak
rs6465657	97816327	C	T	0.47	0.045	7.56e−08	0.621	1.000	0	PC Risk
rs2132276	97826340	G	A	0.47	0.045	5.58e−08	0.559	0.991	1	Flanking

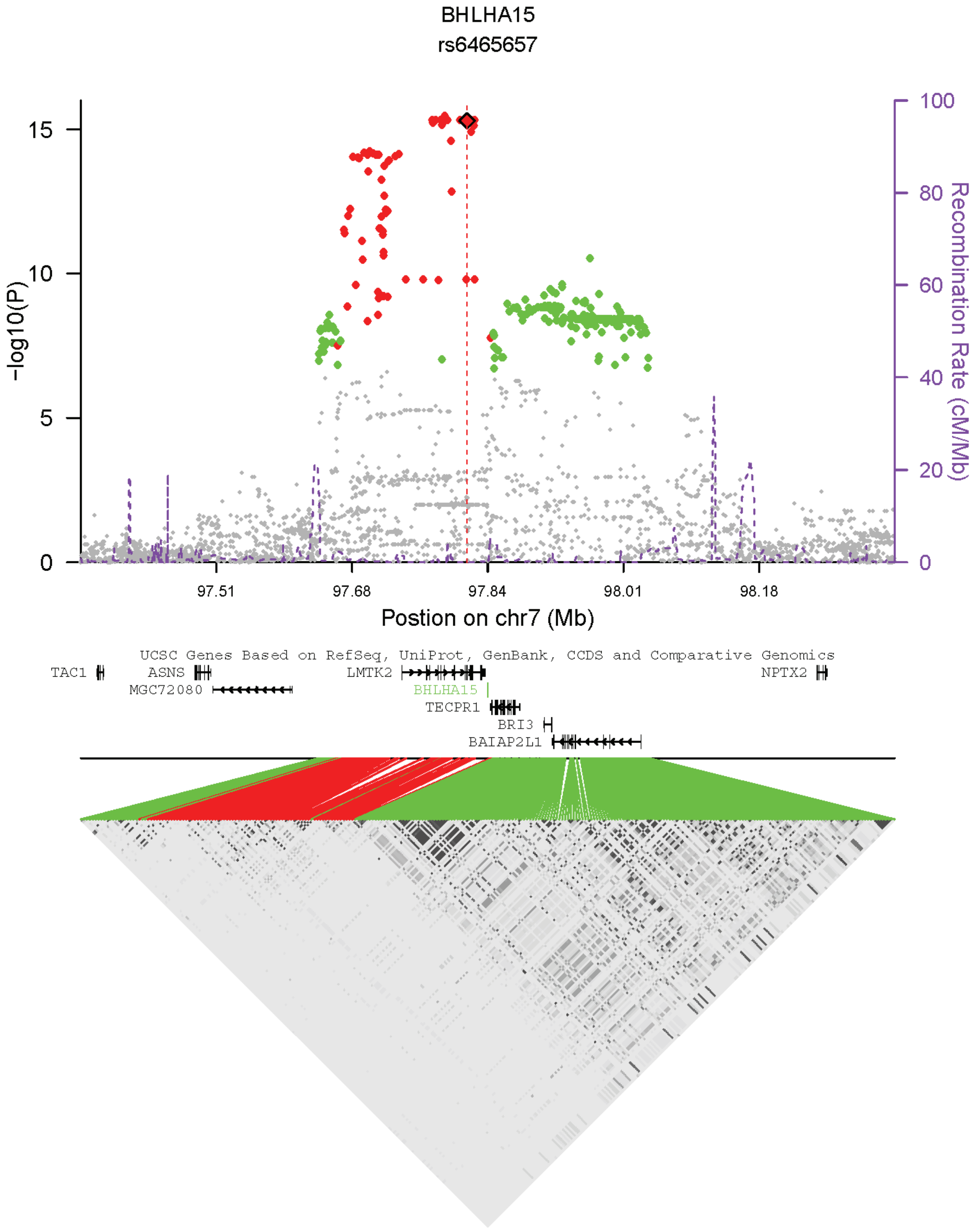

rsID	pos	minor	major	MAF	Beta	p.value	adj.pvalue	rs6465657	imputed	Result Type
rs1180776	97636476	A	G	0.42	−0.175	1.02e−08	0.435	0.442	1	Flanking
rs6958572	97789351	A	G	0.48	0.239	3.52e−16	−	0.946	0	Peak
rs6465657	97816327	C	T	0.47	0.236	5.08e−16	0.431	1.000	0	PC Risk
rs11496115	98037034	A	G	0.49	−0.177	1.15e−08	0.623	0.422	1	Flanking

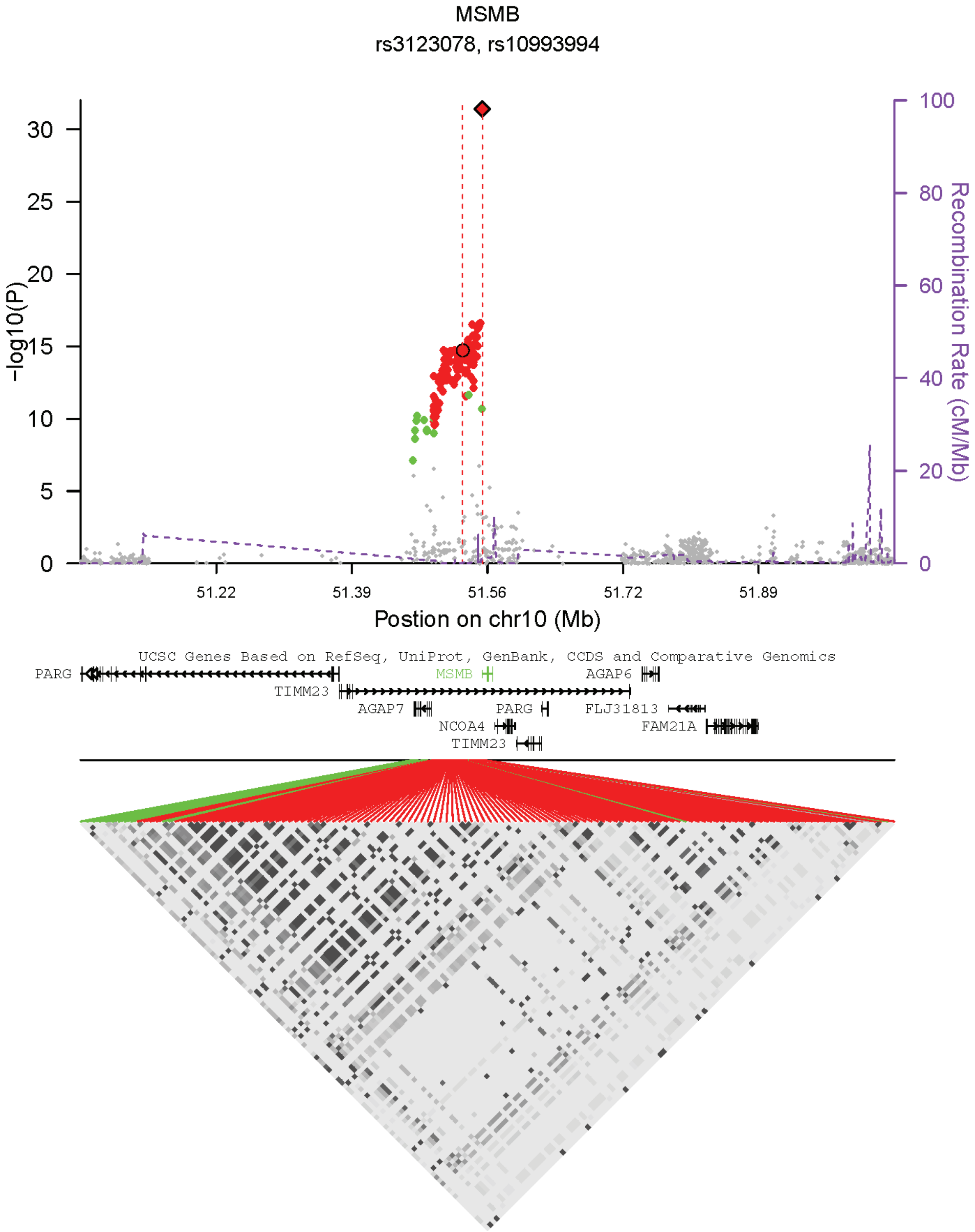

rsID	pos	minor	major	MAF	Beta	p.value	adj.pvalue	rs3123078	rs10993994	imputed	Result Type
chr10:51465650	51465650	C	T	0.46	−0.172	2.50e−09	2.33e−01	0.432	0.383	1	Flanking
rs3123078	51524971	C	T	0.48	−0.190	1.81e−15	8.25e−07	1.000	0.788	0	PC Risk
rs10763536	51535801	A	G	0.49	0.189	1.74e−14	2.86e−06	0.935	0.752	1	ReverseBeta
rs10993994	51549496	T	C	0.42	−0.273	4.06e−32	−	0.788	1.000	0	Peak

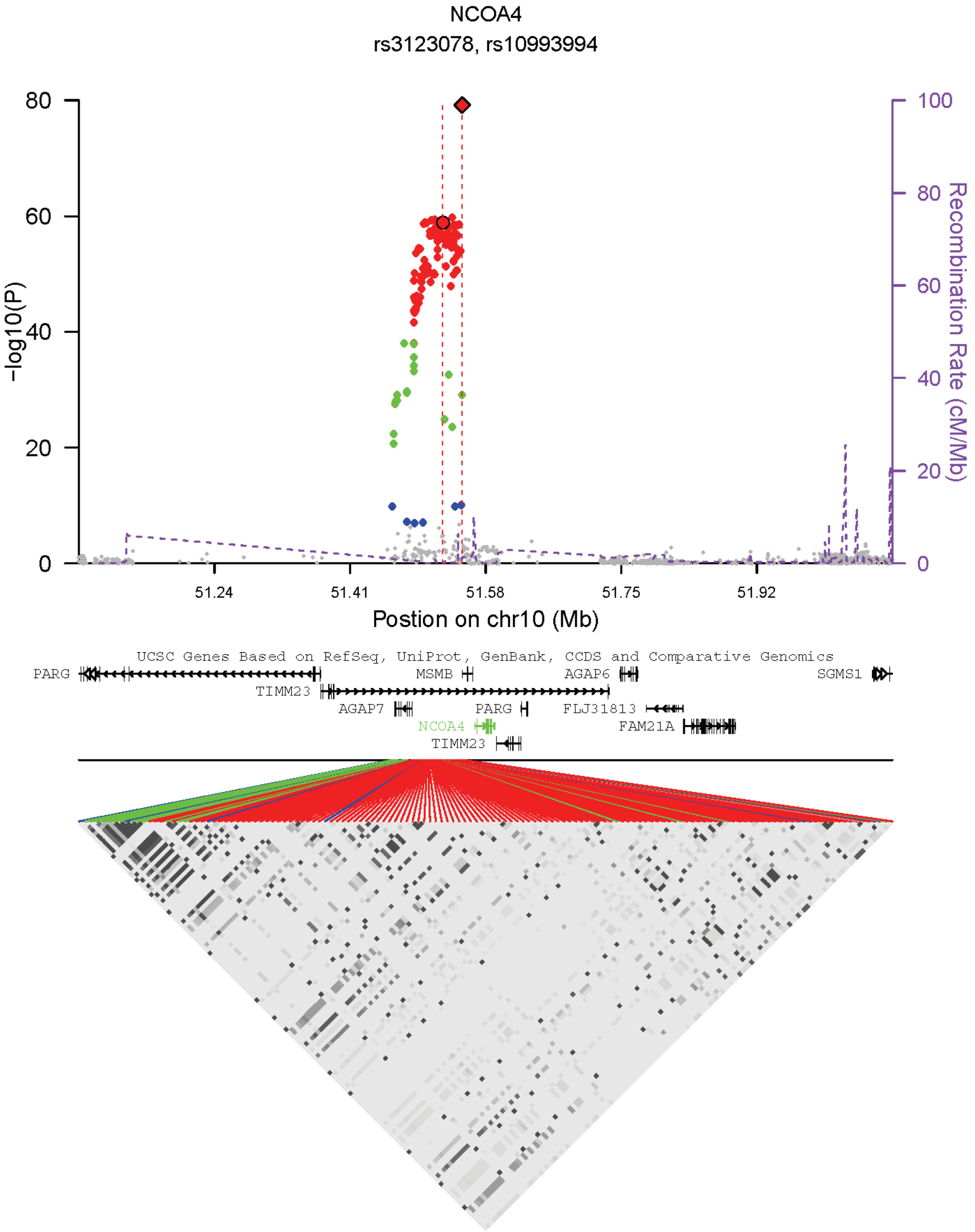

rsID	pos	minor	major	MAF	Beta	p.value	adj.pvalue	rs3123078	rs10993994	imputed	Result Type
rs79489567	51461808	A	G	0.09	0.340	1.54e−10	0.0541	<0.2	<0.2	1	Flanking
rs3123078	51524971	C	T	0.48	0.307	1.06e−59	0.3452	1.000	0.788	0	PC Risk
rs10763536	51535801	A	G	0.49	−0.309	2.74e−56	0.3898	0.935	0.752	1	ReverseBeta
rs10993994	51549496	T	C	0.42	0.344	6.83e−80	–	0.788	1.000	0	Peak

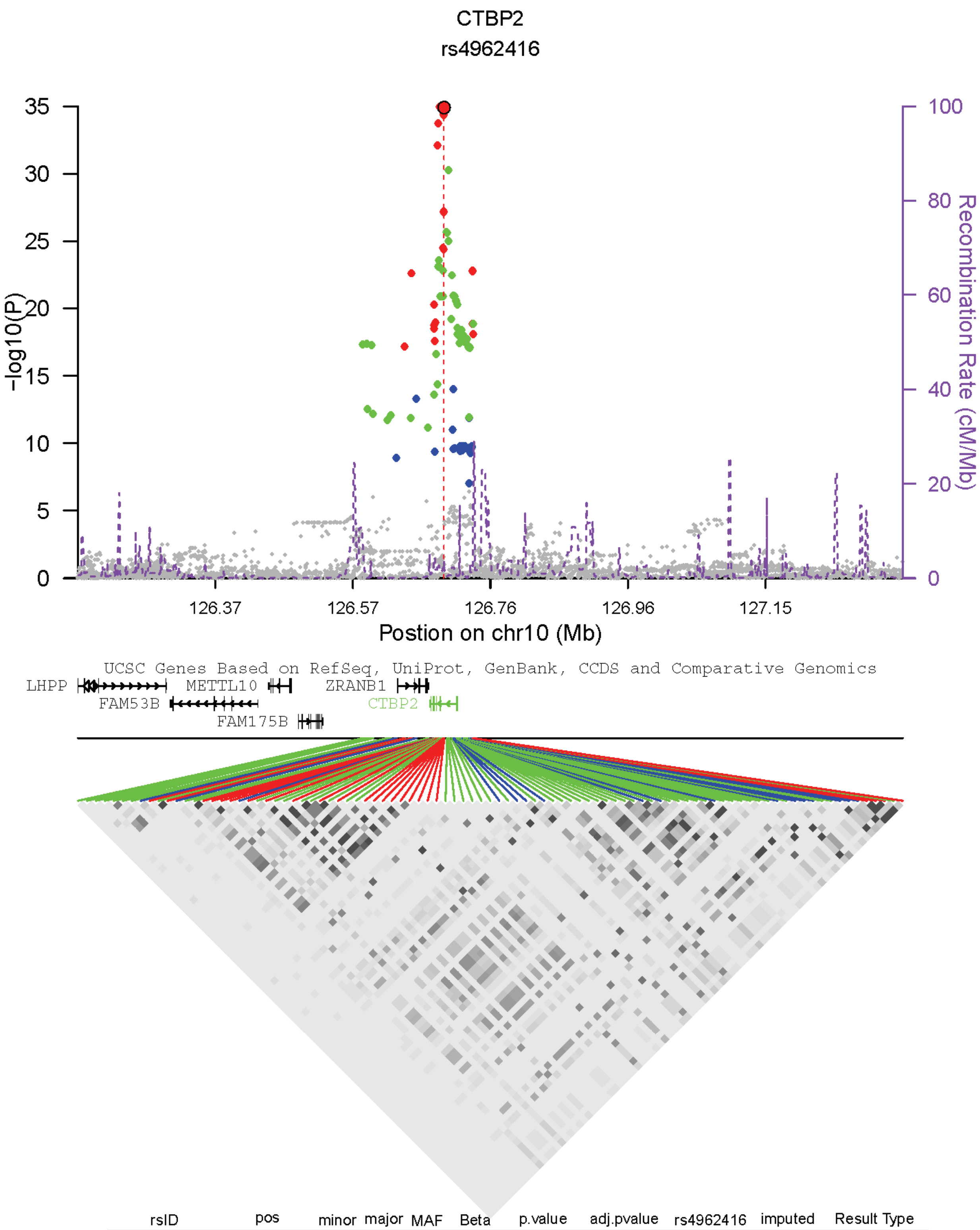

rsID	pos	minor	major	MAF	Beta	p.value	adj.pvalue	rs4962416	imputed	Result Type
rs35186116	126582160	G	A	0.27	0.109	5.10e−18	0.66191	0.482	1	Flanking
rs4962720	126696840	T	G	0.27	0.137	1.19e−35	0.55803	1.000	0	Peak
rs4962416	126696872	C	T	0.27	0.137	1.19e−35	−	1.000	0	Peak
rs4109292	126710654	A	G	0.51	−0.099	1.25e−21	0.00212	0.373	1	ReverseBeta
kgp21878233	126738637	C	A	0.37	0.094	8.17e−19	0.37272	0.519	1	Flanking

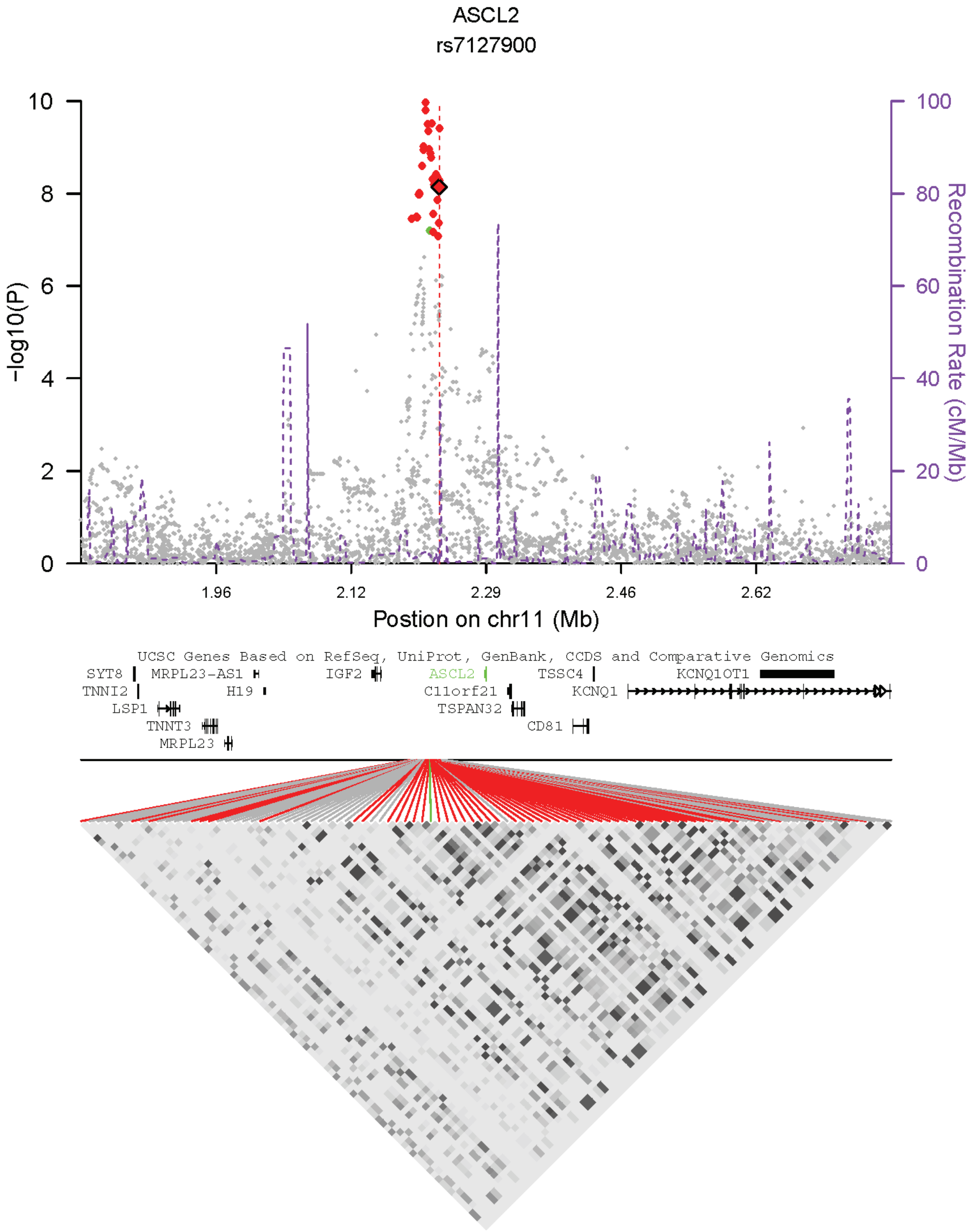

rsID	pos	minor	major	MAF	Beta	p.value	adj.pvalue	rs7127900	imputed	Result Type
rs146569428	2199686	A	G	0.25	0.165	3.52e−08	0.650	0.612	1	Flanking
rs12419367	2217280	A	G	0.25	0.192	1.07e−10	–	0.718	0	Peak
rs7127900	2233574	A	G	0.23	0.180	7.26e−09	0.519	1.000	0	PC Risk
rs10840606	2234690	G	A	0.21	0.197	3.93e−10	0.263	0.872	0	Flanking

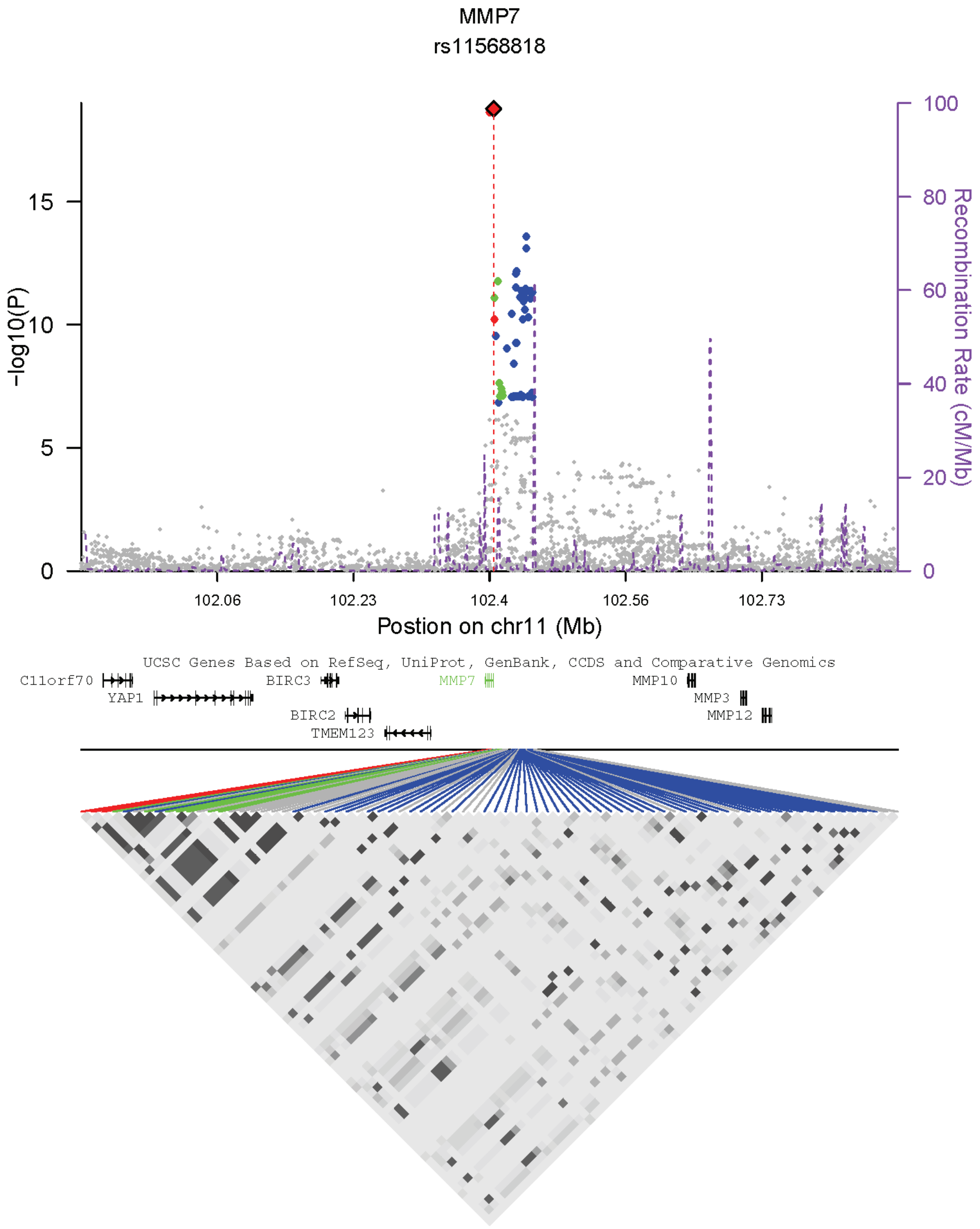

rsID	pos	minor	major	MAF	Beta	p.value	adj.pvalue	rs11568818	imputed	Result Type
rs12285347	102396607	C	T	0.46	−0.564	2.52e−19	7.72e−01	0.997	0	Flanking
rs11568818	102401661	C	T	0.46	−0.568	1.70e−19	−	1.000	0	Peak
rs1940054	102449951	A	C	0.47	0.409	4.85e−12	8.04e−13	<0.2	1	Flanking

COL2A1

rs80130819, rs731236

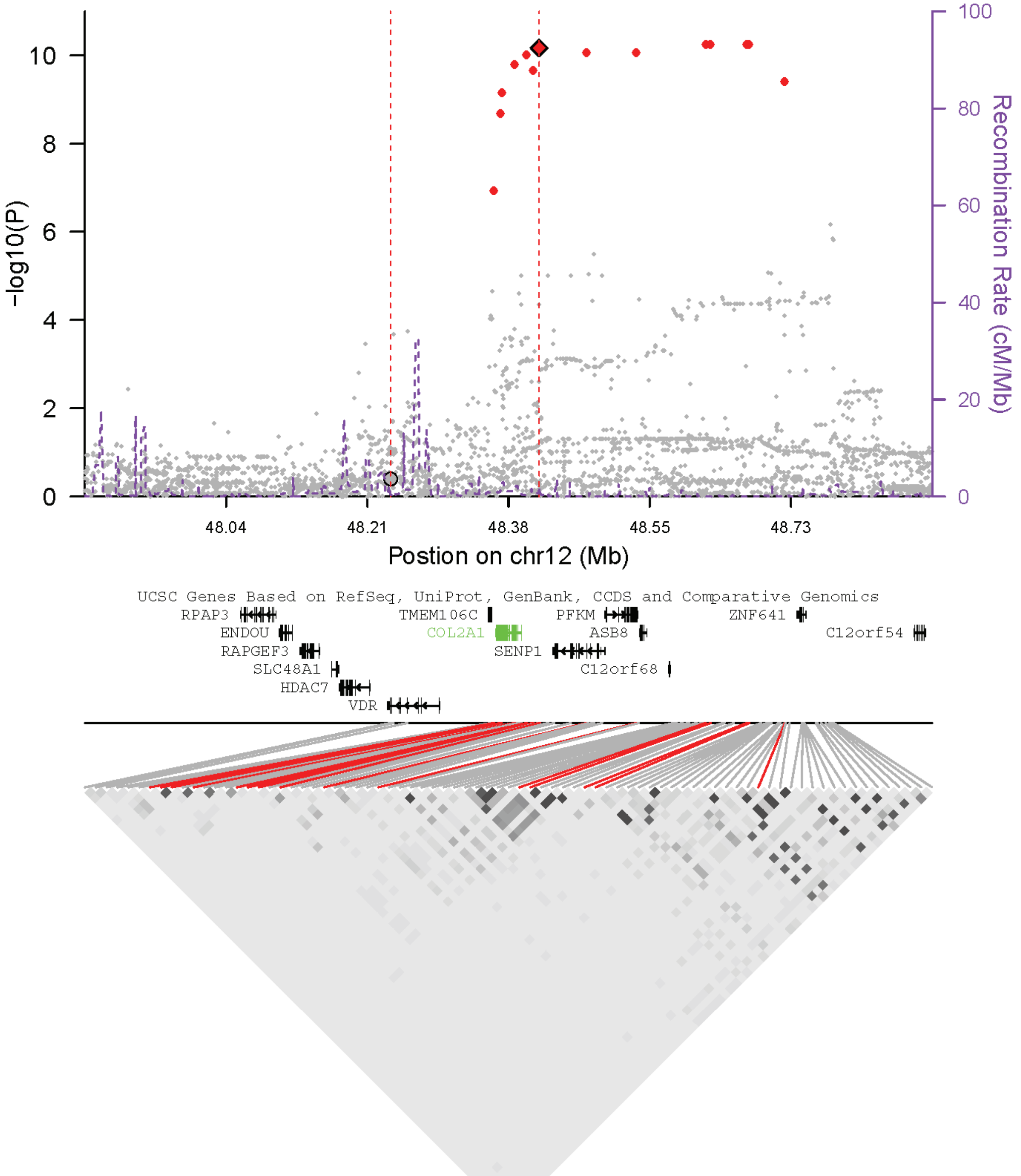

rsID	pos	minor	major	MAF	Beta	p.value	adj.pvalue	rs731236	rs80130819	imputed	Result Type
rs731236	48238757	G	A	0.39	0.078	3.90e−01	0.5148	1.000	<0.2	0	PC Risk
rs7968852	48259741	A	G	0.47	0.247	7.40e−03	0.0313	0.533	<0.2	1	MaxLD
rs4760607	48365265	G	T	0.07	0.955	1.17e−07	0.6509	<0.2	0.633	0	Flanking
rs80130819	48419618	C	A	0.07	1.176	6.73e−11	0.4487	<0.2	1.000	1	PC Risk
rs2087343	48675783	G	A	0.07	1.178	5.62e−11	–	<0.2	0.927	0	Peak
rs2450995	48719172	T	C	0.08	1.072	3.86e−10	0.5567	<0.2	0.787	1	Flanking

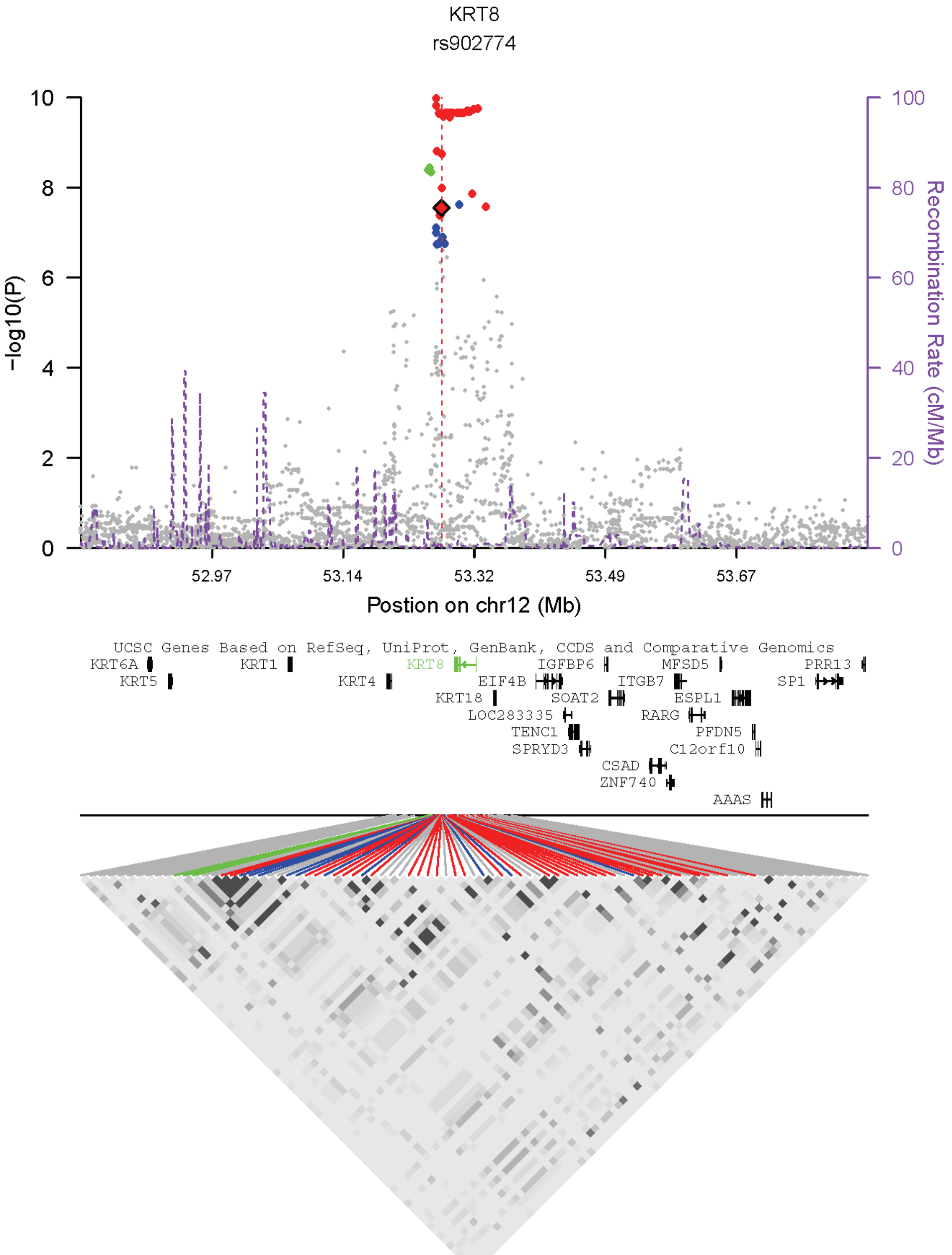

rsID	pos	minor	major	MAF	Beta	p.value	adj.pvalue	rs902774	imputed	Result Type
rs58017462	53256863	A	G	0.09	−0.135	4.07e−09	0.085229	0.482	1	Flanking
rs73106451	53266739	A	C	0.14	−0.119	1.06e−10	–	0.782	1	Peak
rs902774	53273904	A	G	0.17	−0.098	2.78e−08	0.769273	1.000	0	PC Risk
rs4919703	53297594	C	G	0.36	0.075	2.41e−08	0.000142	<0.2	1	ReverseBeta
rs117412892	53333309	A	G	0.15	−0.109	2.65e−08	0.896989	0.559	1	Flanking

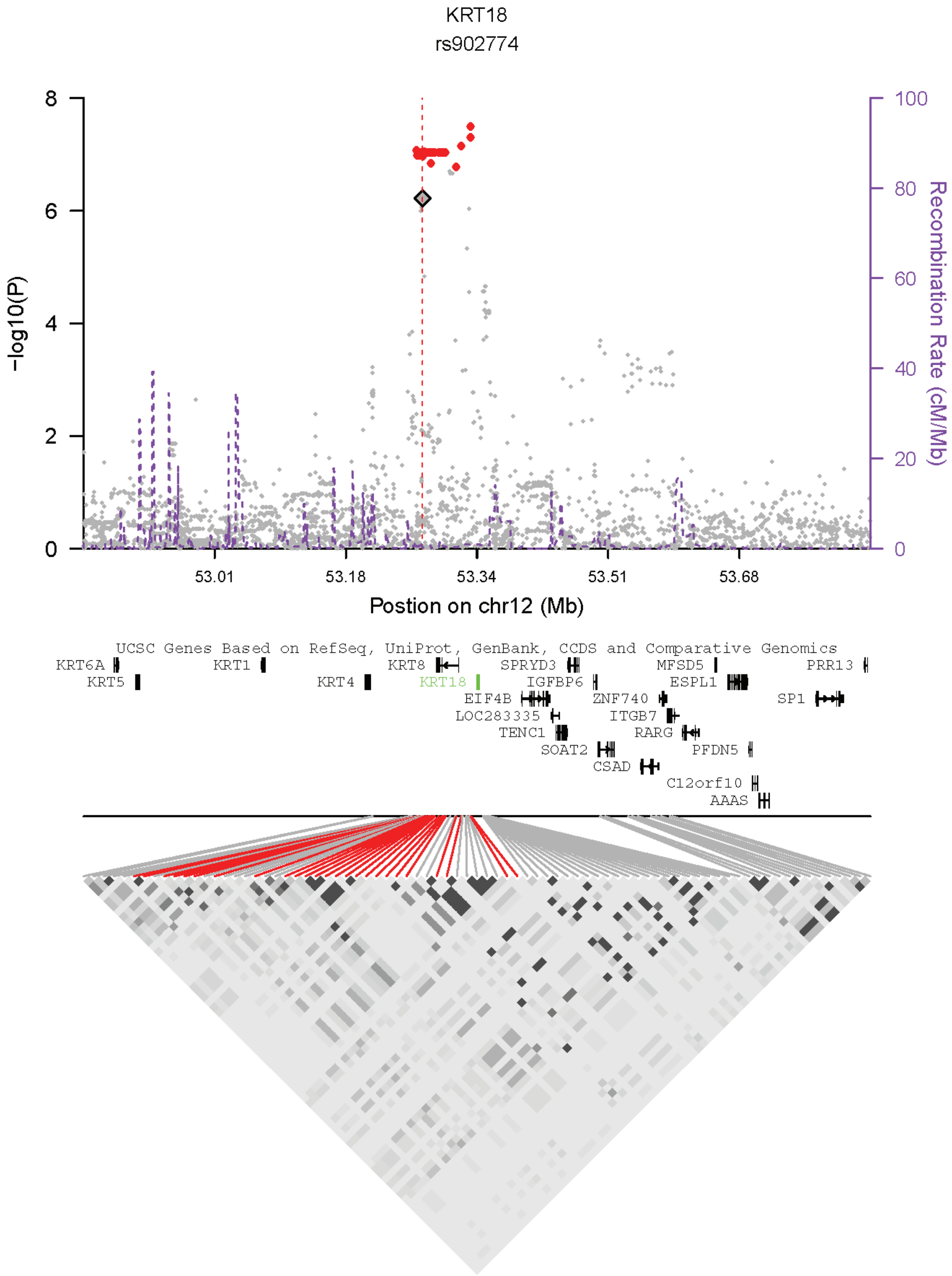

rsID	pos	minor	major	MAF	Beta	p.value	adj.pvalue	rs902774	imputed	Result Type
rs73106451	53266739	A	C	0.14	−0.066	8.42e−08	0.0268	0.782	1	Flanking
rs902774	53273904	A	G	0.17	−0.059	6.04e−07	0.1816	1.000	0	PC Risk
rs12302939	53335216	G	C	0.22	−0.058	3.21e−08	–	0.547	1	Peak
rs11170341	53335563	C	T	0.22	−0.056	5.01e−08	0.3290	0.539	0	Flanking

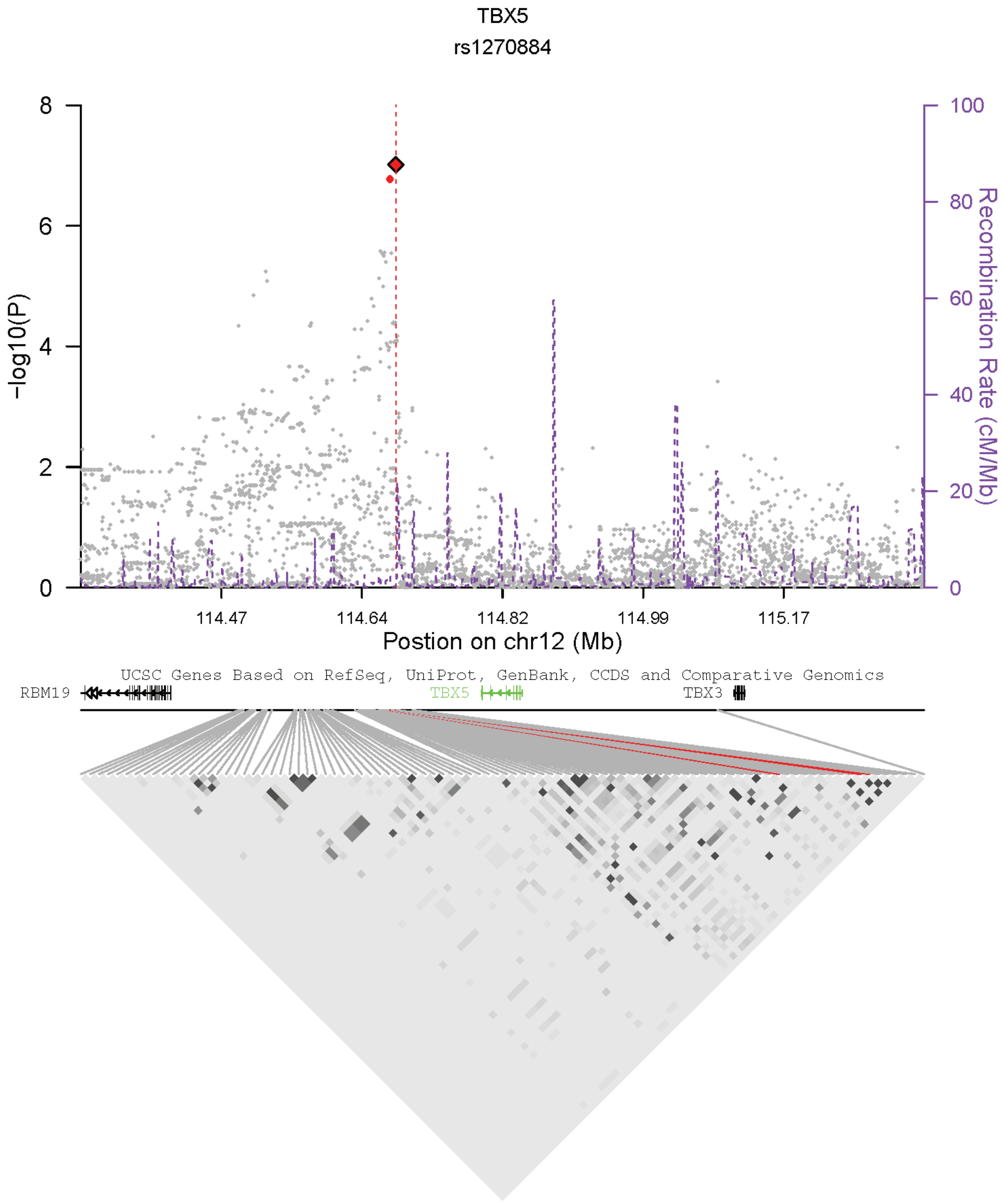

rsID	pos	minor	major	MAF	Beta	p.value	adj.pvalue	rs1270884	imputed	Result Type
rs2555013	114678318	C	T	0.48	0.180	1.68e−07	0.528	0.991	1	Flanking
rs1270884	114685571	G	A	0.48	0.183	9.68e−08	0.890	1.000	1	PC Risk
rs2555004	114686645	A	G	0.48	0.182	9.38e−08	−	1.000	0	Peak

C14orf39

rs7153648

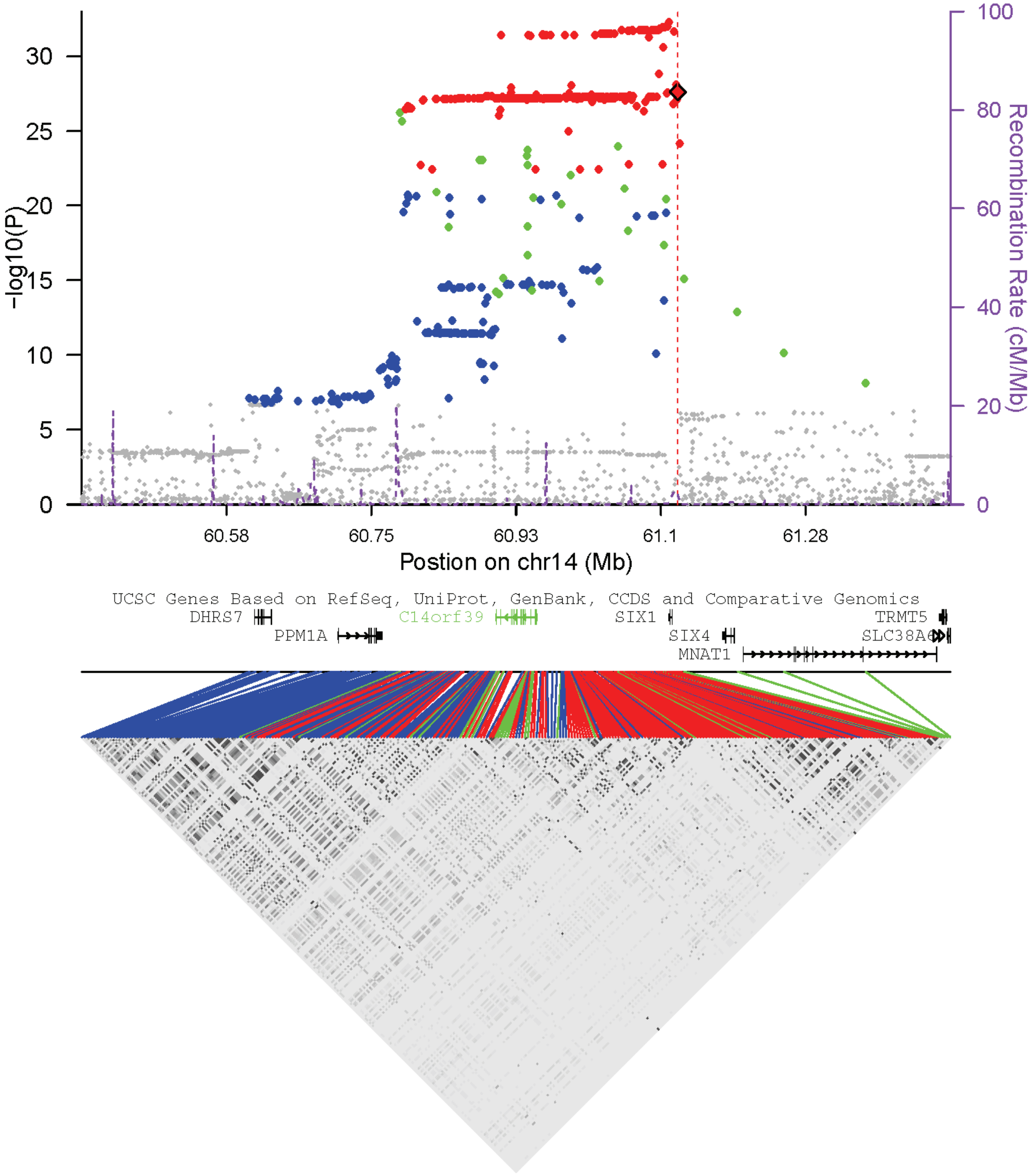

rsID	pos	minor	major	MAF	Beta	p.value	adj.pvalue	rs7153648	imputed	Result Type
rs216501	60640388	T	C	0.13	−0.346	2.63e−08	0.0615	<0.2	0	Flanking
rs10144415	61112667	G	C	0.10	−0.792	5.30e−33	−	0.778	1	Peak
rs7153648	61122526	C	G	0.09	−0.783	2.45e−28	0.3088	1.000	1	PC Risk
rs8004224	61349255	A	C	0.03	−0.720	8.16e−09	0.9517	0.309	1	Flanking

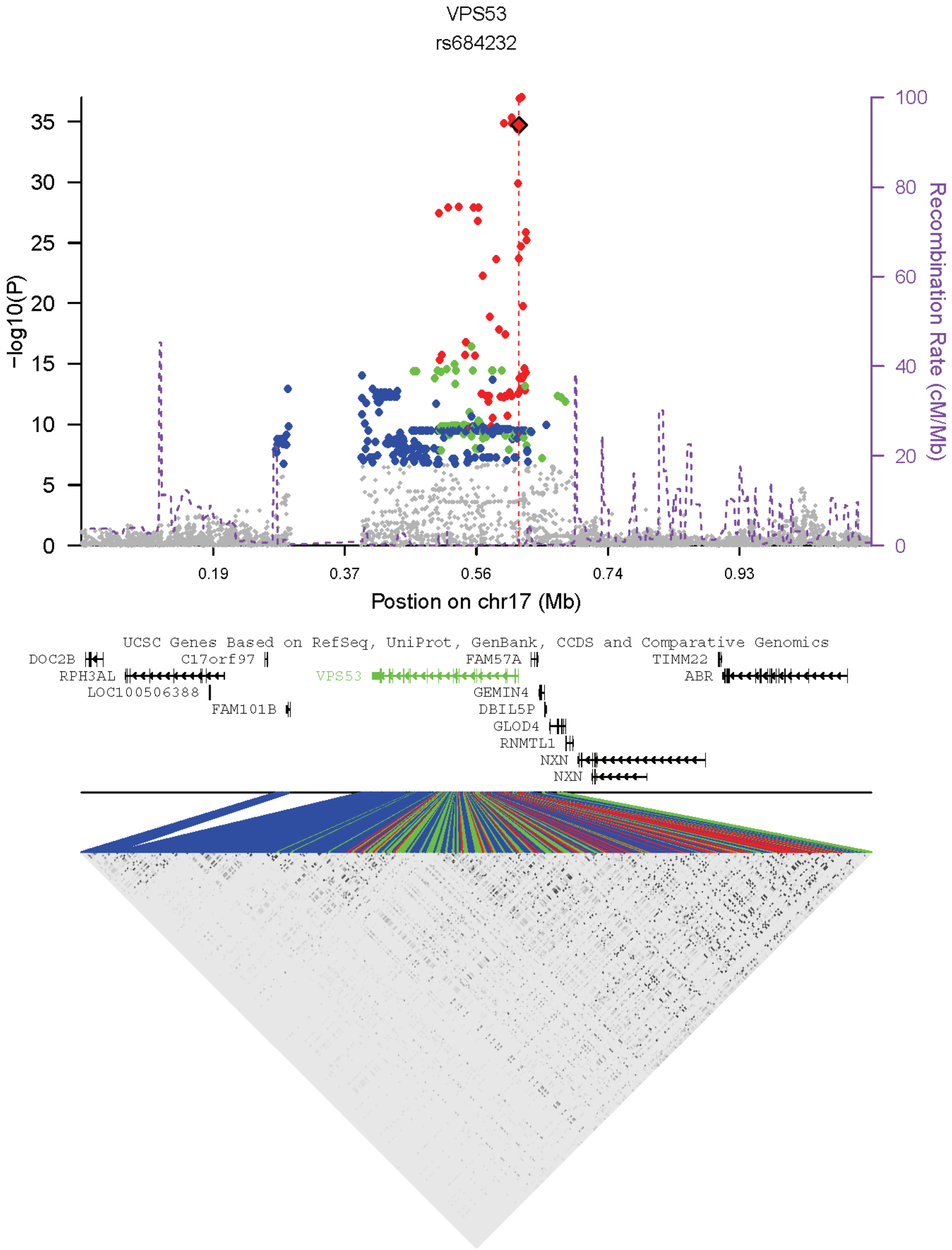

rsID	pos	minor	major	MAF	Beta	p.value	adj.pvalue	rs684232	imputed	Result Type
rs182175045	276230	T	G	0.03	0.140	1.24e−08	3.38e−08	<0.2	1	Flanking
rs684232	618965	C	T	0.39	−0.100	1.92e−35	6.48e−01	1.000	0	PC Risk
rs437948	623356	G	C	0.40	−0.103	1.04e−37	–	0.914	1	Peak
rs2273454	685640	T	G	0.22	−0.069	1.42e−12	5.03e−01	0.302	0	Flanking

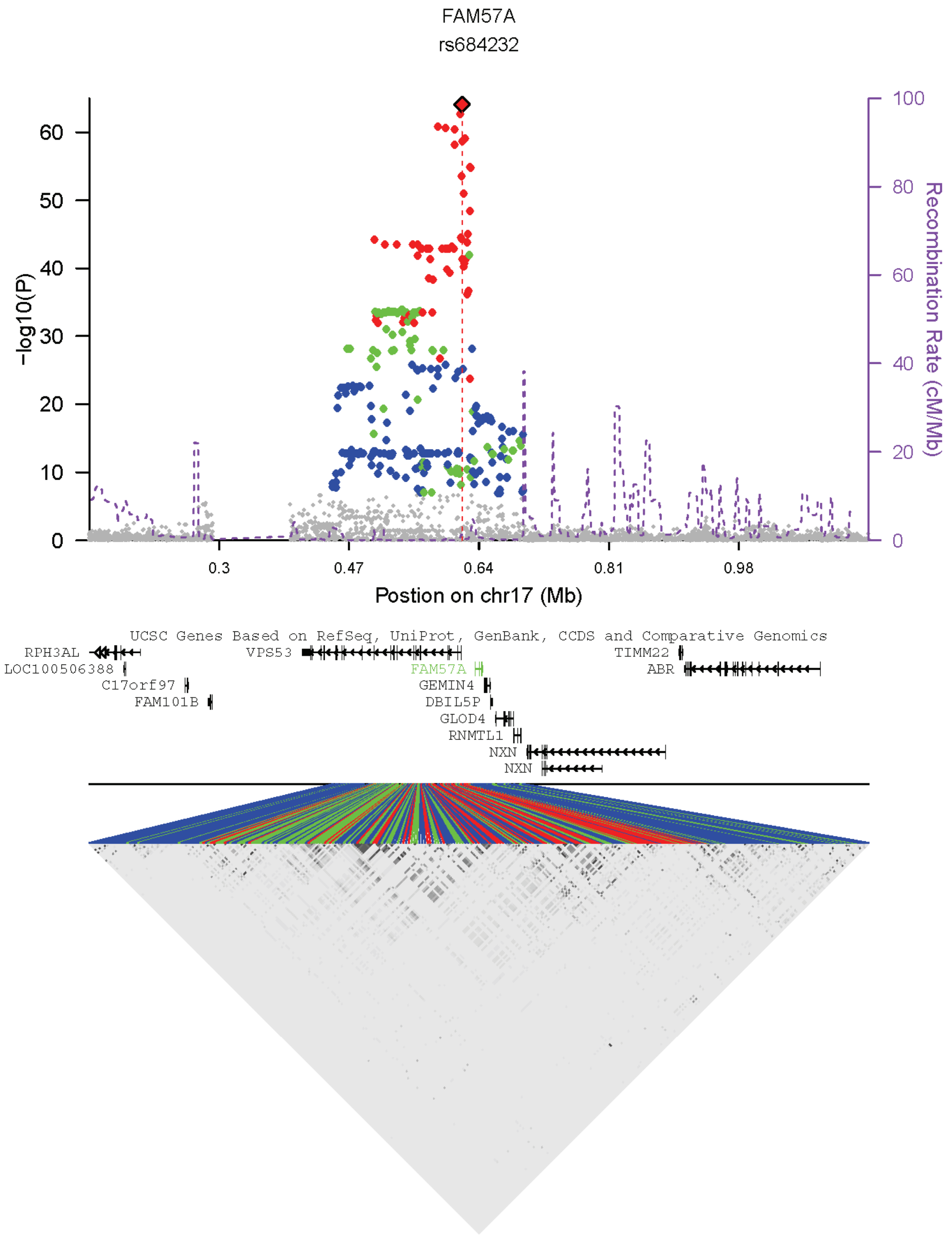

rsID	pos	minor	major	MAF	Beta	p.value	adj.pvalue	rs684232	imputed	Result Type
rs4968072	451732	G	A	0.35	−0.112	1.38e−08	4.56e−01	<0.2	1	Flanking
rs684232	618965	C	T	0.39	−0.240	8.16e−65	6.84e−03	1.000	0	PC Risk
rs461251	619162	G	A	0.41	−0.240	3.22e−65	–	0.917	1	Peak
rs72810267	698814	A	G	0.25	0.166	3.09e−16	1.35e−08	<0.2	1	Flanking

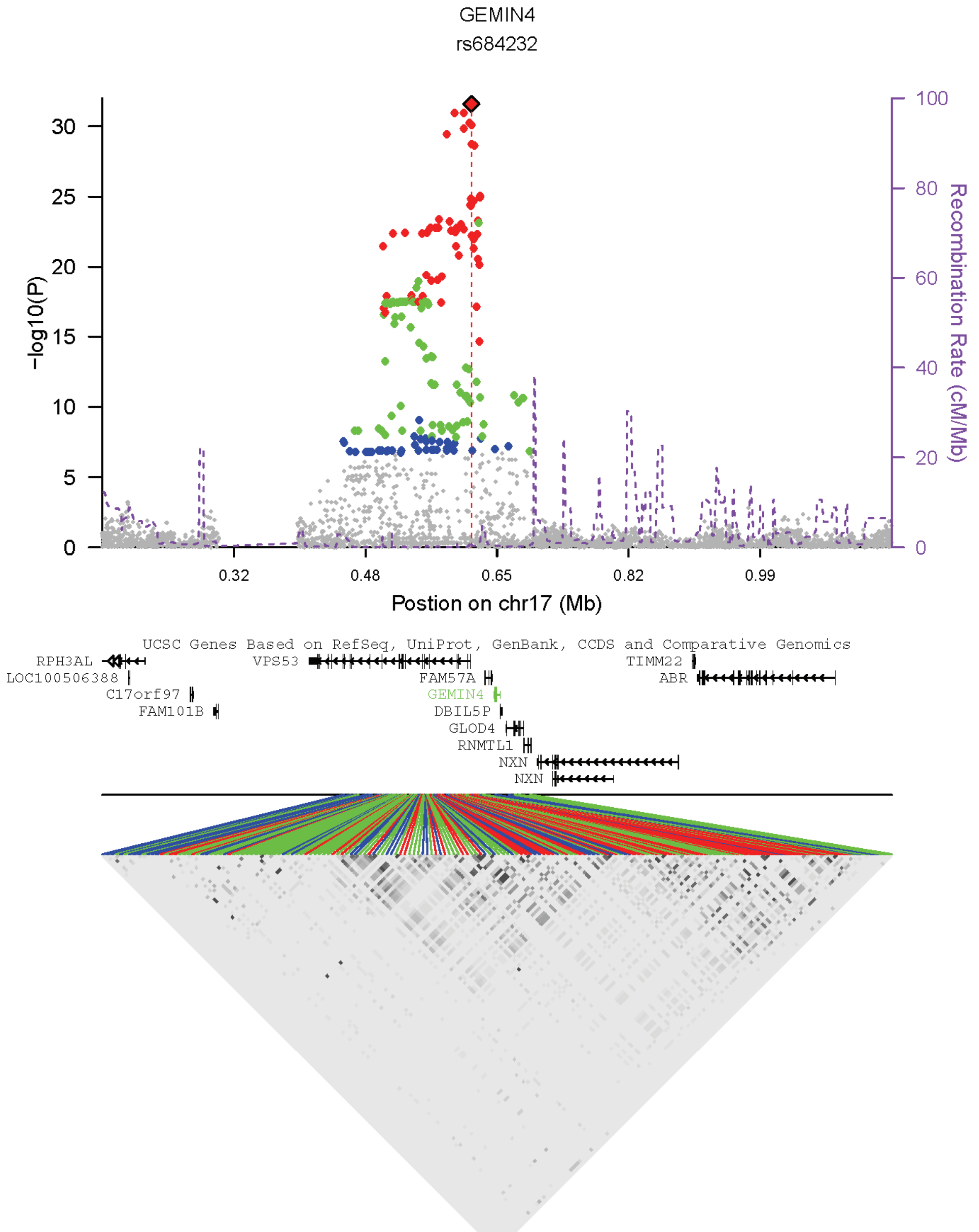

rsID	pos	minor	major	MAF	Beta	p.value	adj.pvalue	rs684232	imputed	Result Type
rs4968165	470314	A	C	0.18	−0.070	5.08e−09	0.2438	0.365	1	Flanking
rs2474694	618039	A	G	0.39	−0.111	1.89e−32	–	1.000	1	Peak
rs2955626	618100	C	G	0.49	0.094	4.31e−25	0.0337	0.636	1	ReverseBeta
rs684232	618965	C	T	0.39	−0.111	2.57e−32	0.3981	1.000	0	PC Risk
rs2273454	685640	T	G	0.22	−0.075	2.57e−11	0.6763	0.302	0	Flanking

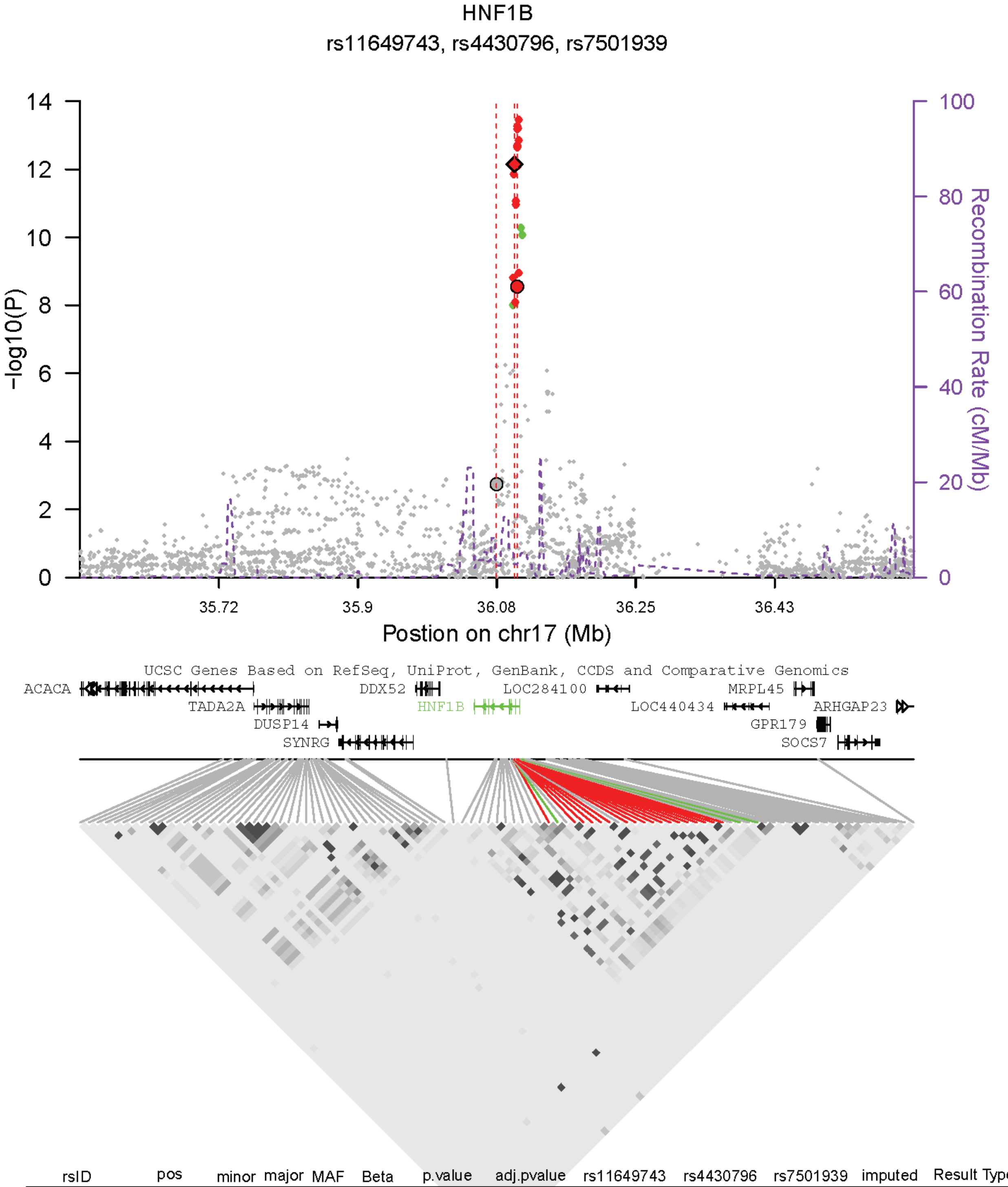

rsID	pos	minor	major	MAF	Beta	p.value	adj.pvalue	rs11649743	rs4430796	rs7501939	imputed	Result Type
rs11649743	36074979	A	G	0.21	−0.084	1.77e−03	0.000774	1.000	<0.2	<0.2	0	PC Risk
rs7223387	36082473	G	T	0.28	−0.079	1.22e−03	0.005402	0.633	<0.2	<0.2	0	MaxLD
rs2005705	36096300	A	G	0.41	−0.131	1.61e−09	0.120599	<0.2	0.809	0.519	1	Flanking
rs4430796	36098040	G	A	0.43	−0.154	7.18e−13	0.014737	<0.2	1.000	0.669	0	PC Risk
rs7501939	36101156	T	C	0.35	−0.131	2.78e−09	0.164378	<0.2	0.669	1.000	0	PC Risk
rs11263763	36103565	G	A	0.43	−0.162	1.42e−13	0.015050	<0.2	0.892	0.706	1	MaxLD
rs12453443	36104121	G	C	0.46	0.169	3.55e−14	−	<0.2	0.530	0.420	1	Peak
rs67143603	36108231	C	T	0.37	0.154	8.74e−11	0.124766	<0.2	0.268	0.222	1	Flanking

PPP1R14A
rs8102476

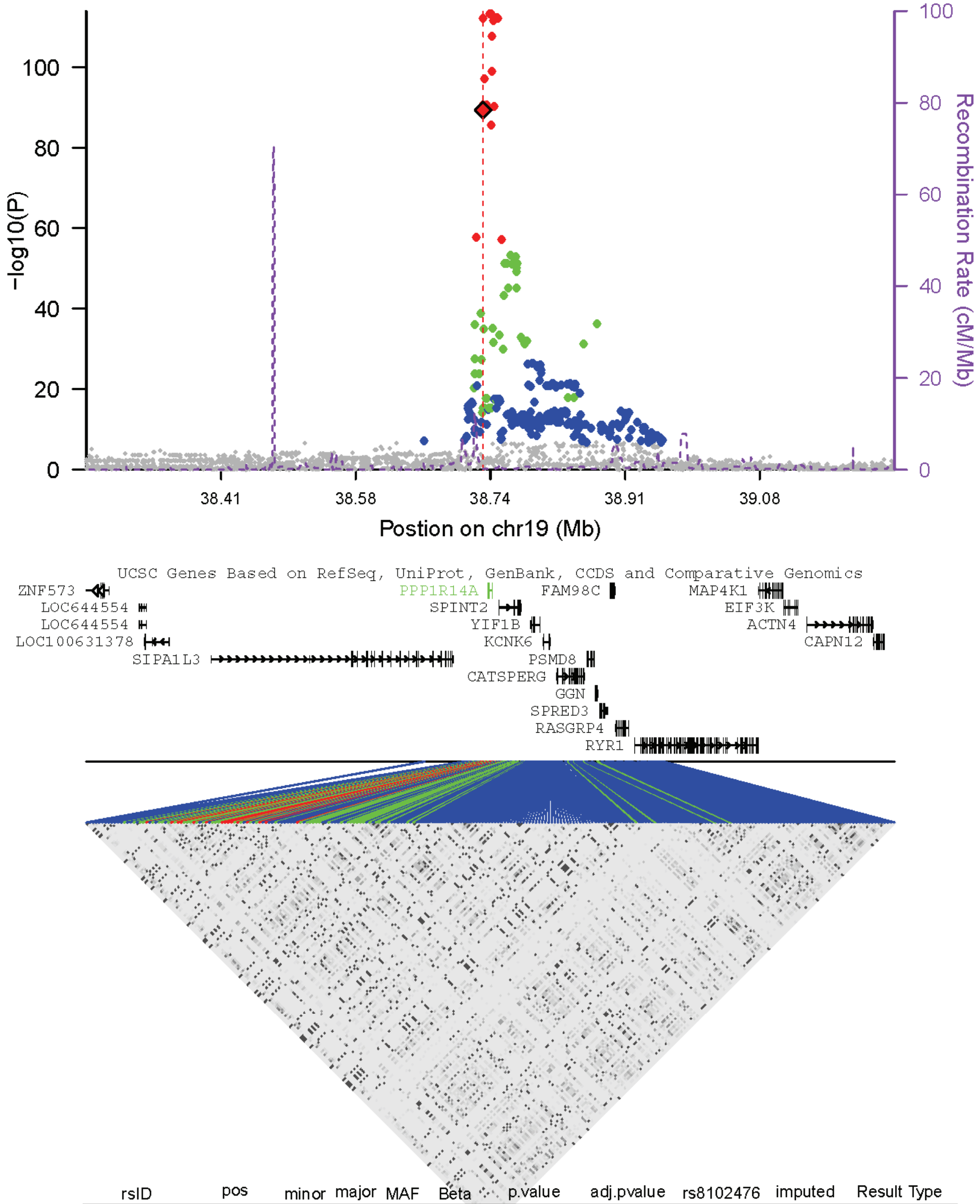

rsID	pos	minor	major	MAF	Beta	p.value	adj.pvalue	rs8102476	imputed	Result Type
rs12978169	38715814	A	G	0.27	−0.149	8.34e−09	0.457	<0.2	1	Flanking
rs4803899	38727640	A	T	0.49	0.328	2.52e−58	0.218	0.588	1	ReverseBeta
rs8102476	38735613	T	C	0.43	−0.372	4.05e−90	0.928	1.000	0	PC Risk
rs12976534	38743962	G	A	0.47	−0.400	5.85e−114	0.688	0.858	0	Peak
rs12610267	38744733	G	A	0.47	−0.400	5.85e−114	−	0.855	0	Peak
rs7259574	38956176	A	G	0.35	−0.142	1.52e−08	0.938	<0.2	1	Flanking

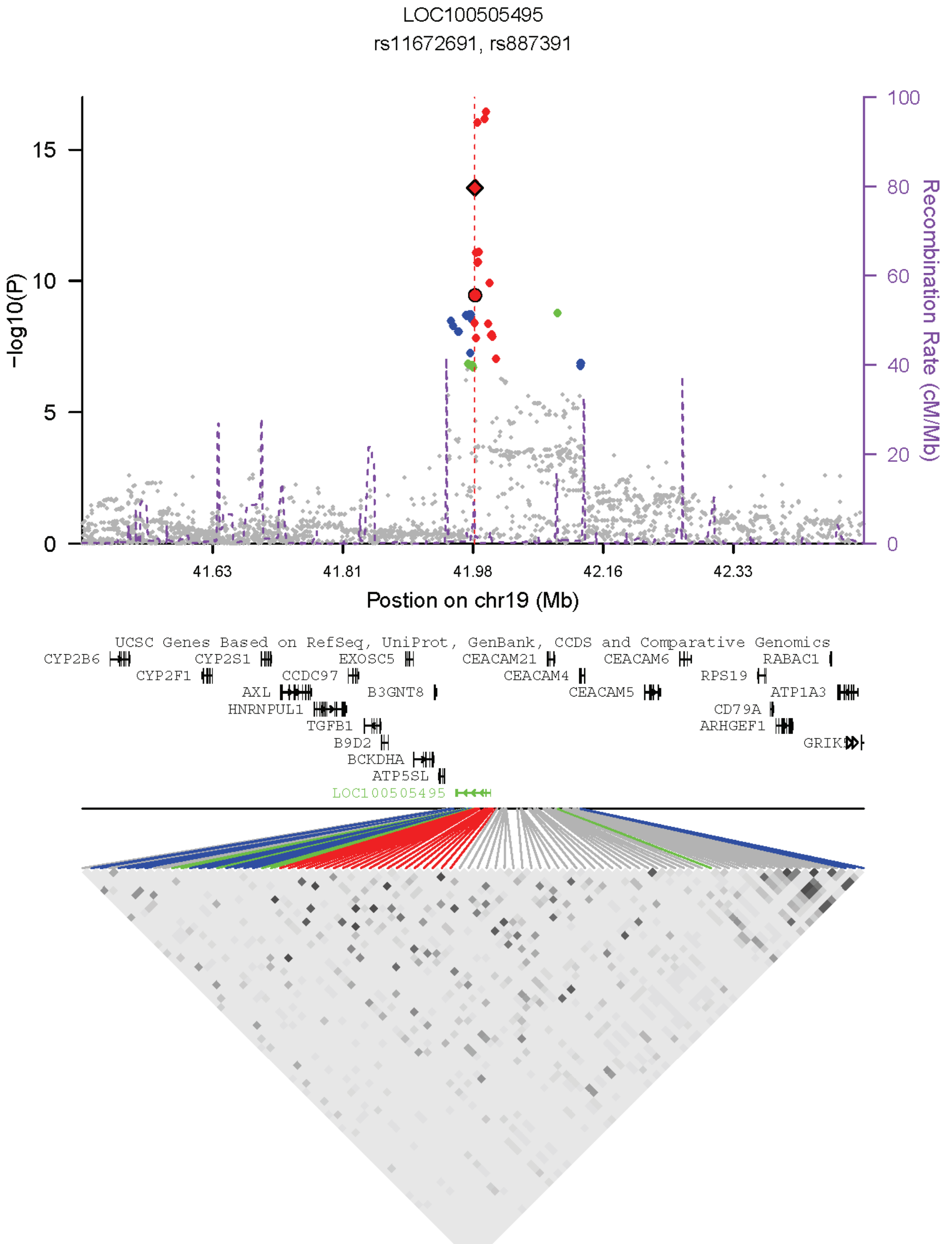

rsID	pos	minor	major	MAF	Beta	p.value	adj.pvalue	rs11672691	rs887391	imputed	Result Type
rs2316973	41953928	G	T	0.05	−0.271	3.49e−09	0.00187	<0.2	<0.2	0	Flanking
rs11672691	41985587	A	G	0.25	−0.173	2.86e−14	0.76263	1.000	0.860	0	PC Risk
rs887391	41985624	C	T	0.22	−0.150	3.46e−10	0.46068	0.860	1.000	1	PC Risk
rs2191139	42001210	T	C	0.23	−0.198	3.64e−17	−	0.804	0.667	0	Peak
rs731055	42096416	C	T	0.17	−0.166	1.68e−09	0.87113	0.382	0.284	1	Flanking

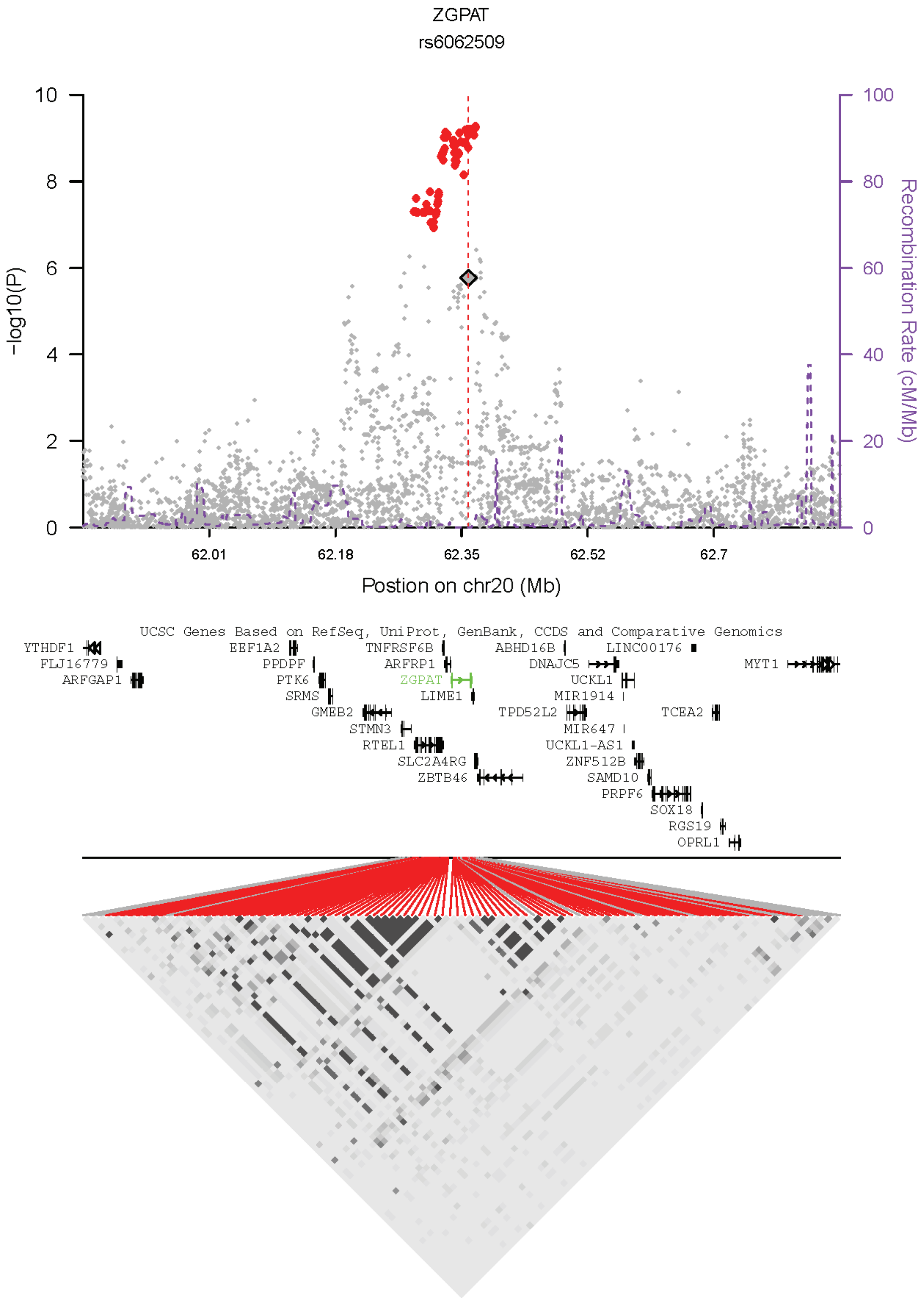

rsID	pos	minor	major	MAF	Beta	p.value	adj.pvalue	rs6062509	imputed	Result Type
rs3761124	62288752	T	C	0.24	−0.039	5.04e−08	0.794	0.539	0	Flanking
rs6062509	62362563	G	T	0.31	−0.033	1.70e−06	0.294	1.000	1	PC Risk
rs2253829	62373079	C	G	0.25	−0.045	5.46e−10	–	0.736	1	Peak

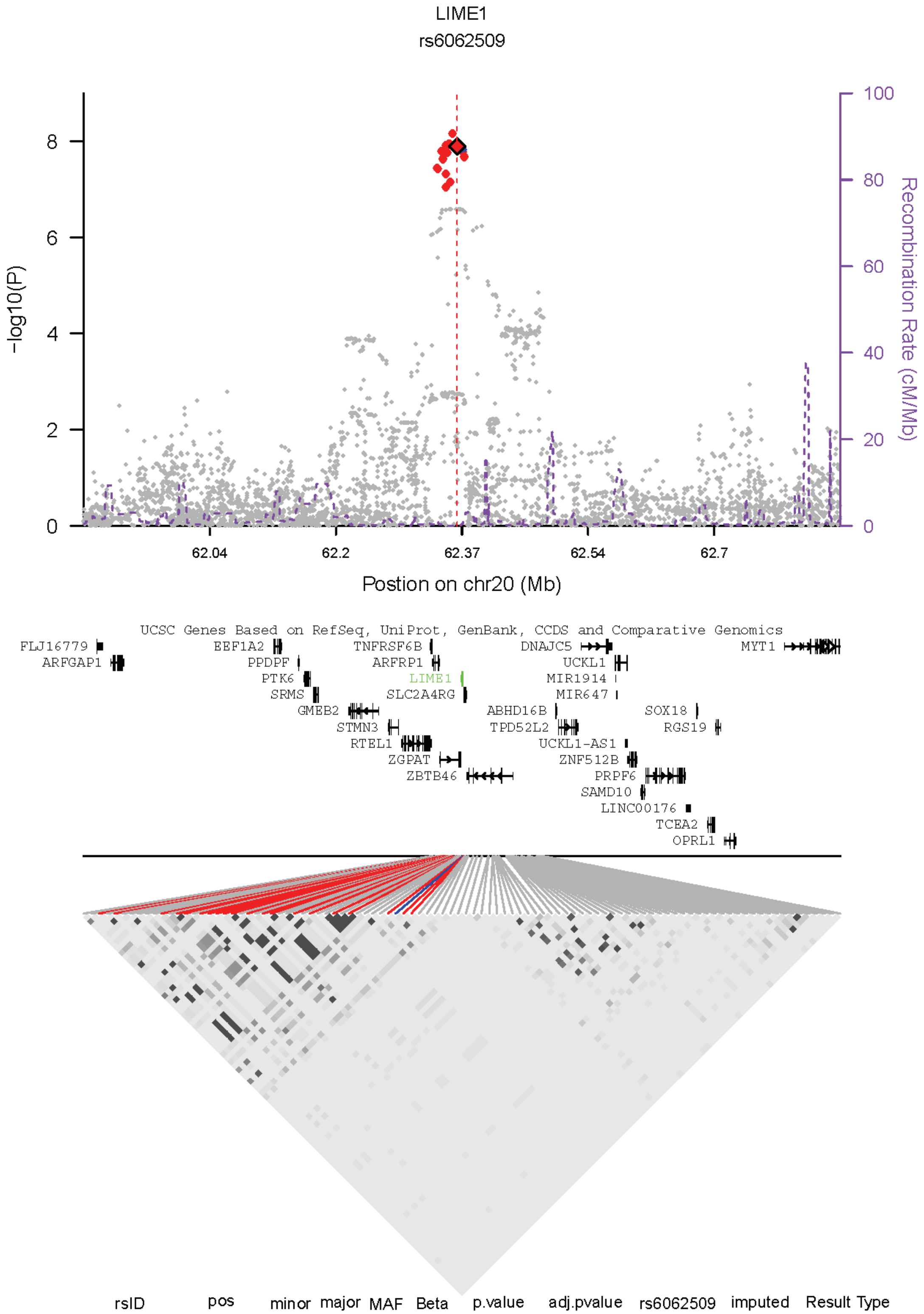

rsID	pos	minor	major	MAF	Beta	p.value	adj.pvalue	rs6062509	imputed	Result Type
rs6062497	62336258	C	T	0.32	0.081	3.61e−08	0.770	0.934	1	Flanking
rs6011058	62356627	C	T	0.31	0.086	7.01e−09	−	0.984	0	Peak
rs6062509	62362563	G	T	0.31	0.084	1.28e−08	0.507	1.000	1	PC Risk
rs4809221	62372706	G	A	0.31	0.083	2.17e−08	0.344	0.996	1	Flanking

GPR143

rs2405942

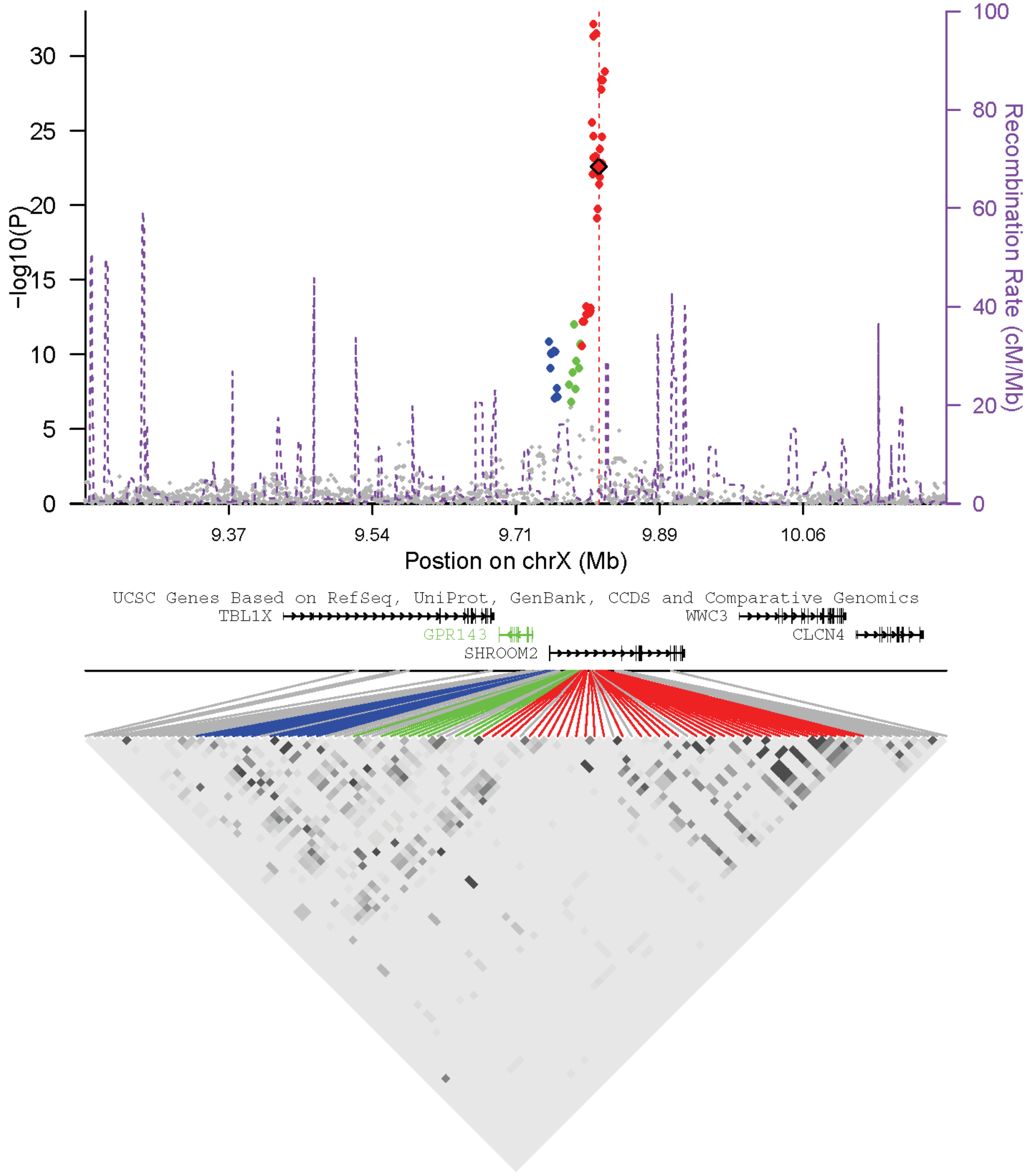

rsID	pos	minor	major	MAF	Beta	p.value	adj.pvalue	rs2405942	imputed	Result Type
rs111660911	9754135	C	A	0.17	−0.127	1.51e−11	1.19e−14	<0.2	1	Flanking
rs4830661	9807693	A	G	0.27	0.170	7.39e−33	–	0.767	0	Peak
rs2405942	9814135	G	A	0.23	0.154	2.50e−23	7.80e−01	1.000	0	PC Risk
rs5934705	9821399	T	C	0.31	0.156	1.17e−29	1.98e−01	0.678	0	Flanking

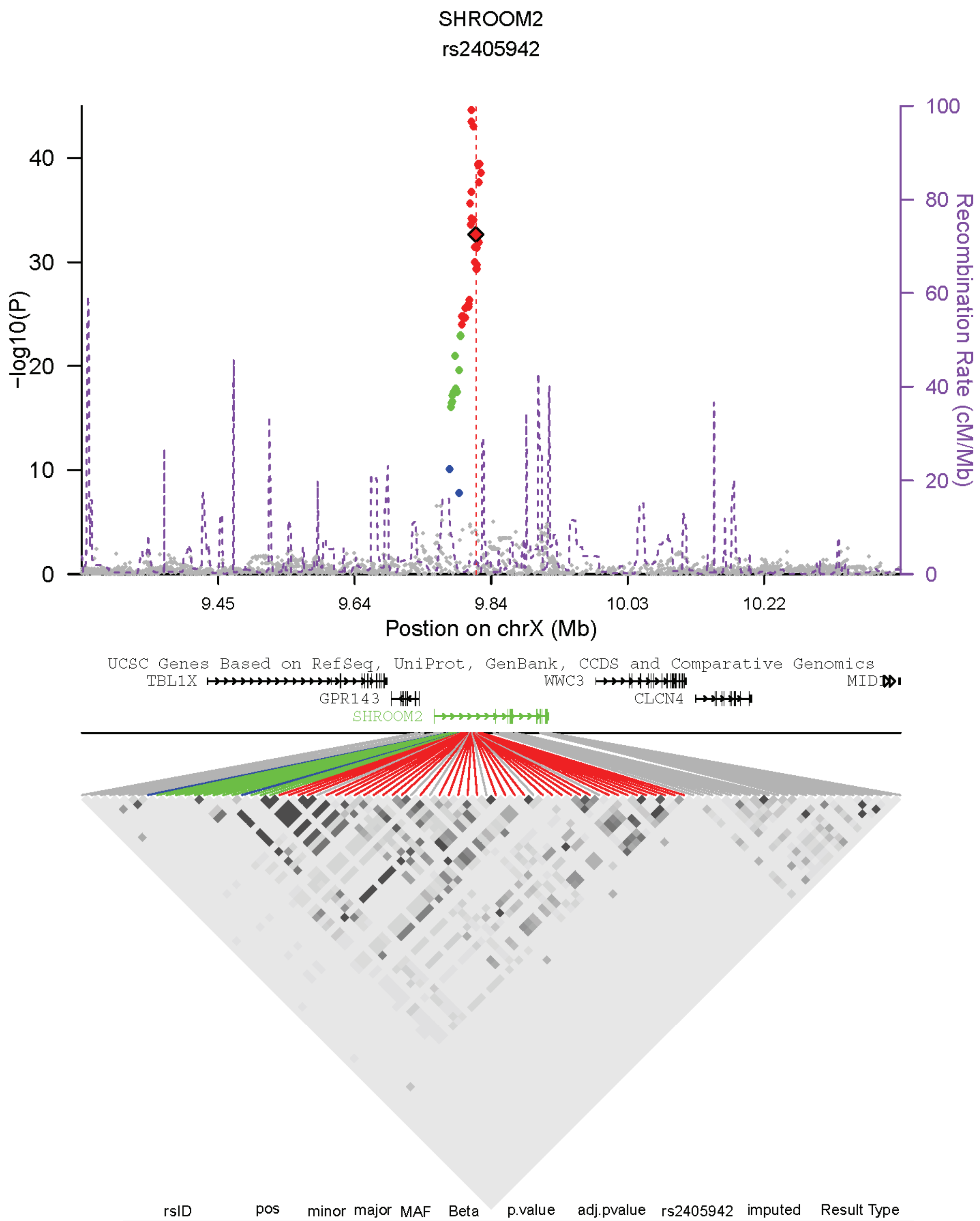

rsID	pos	minor	major	MAF	Beta	p.value	adj.pvalue	rs2405942	imputed	Result Type
rs55743966	9776909	A	T	0.38	0.060	8.41e−11	0.0881	<0.2	0	Flanking
rs116074556	9790986	G	A	0.15	−0.090	1.80e−08	0.0357	<0.2	1	ReverseBeta
rs4830661	9807693	A	G	0.27	0.133	2.62e−45	–	0.767	0	Peak
rs2405942	9814135	G	A	0.23	0.124	2.19e−33	0.6824	1.000	0	PC Risk
rs5934705	9821399	T	C	0.31	0.121	2.71e−39	0.2854	0.678	0	Flanking

NUDT11

rs1327301, rs5945572, rs5945619

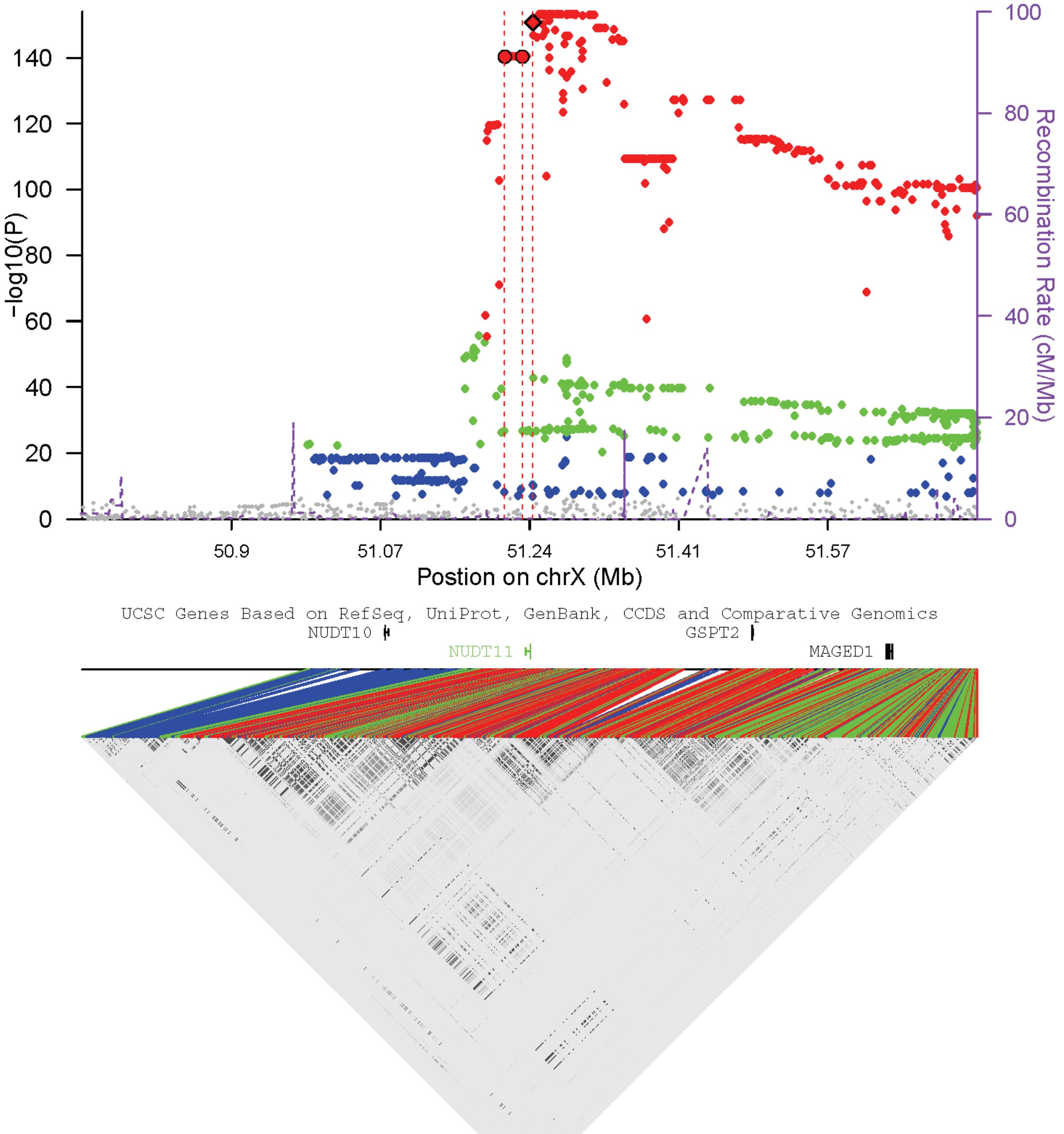

rsID	pos	minor	major	MAF	Beta	p.value	adj.pvalue	rs1327301	rs5945572	rs5945619	imputed	Result Type
rs187324800	50989678	T	C	0.19	0.310	2.90e−23	0.167	0.210	0.210	0.205	1	Flanking
rs1327301	51210057	T	C	0.44	0.460	3.67e−141	0.527	1.000	1.000	0.928	1	PC Risk
rs5945572	51229683	A	G	0.44	0.460	3.43e−141	0.521	1.000	1.000	0.928	0	PC Risk
rs5945619	51241672	C	T	0.45	0.468	2.10e−151	0.593	0.928	0.928	1.000	1	PC Risk
rs2384958	51249397	T	A	0.45	0.469	6.37e−154	–	0.934	0.934	0.994	1	Peak
rs141135935	51256992	G	A	0.46	−0.454	1.07e−104	0.115	0.713	0.713	0.748	1	ReverseBeta
rs190928294	52097394	A	G	0.08	0.290	5.07e−09	0.653	<0.2	<0.2	<0.2	1	Flanking

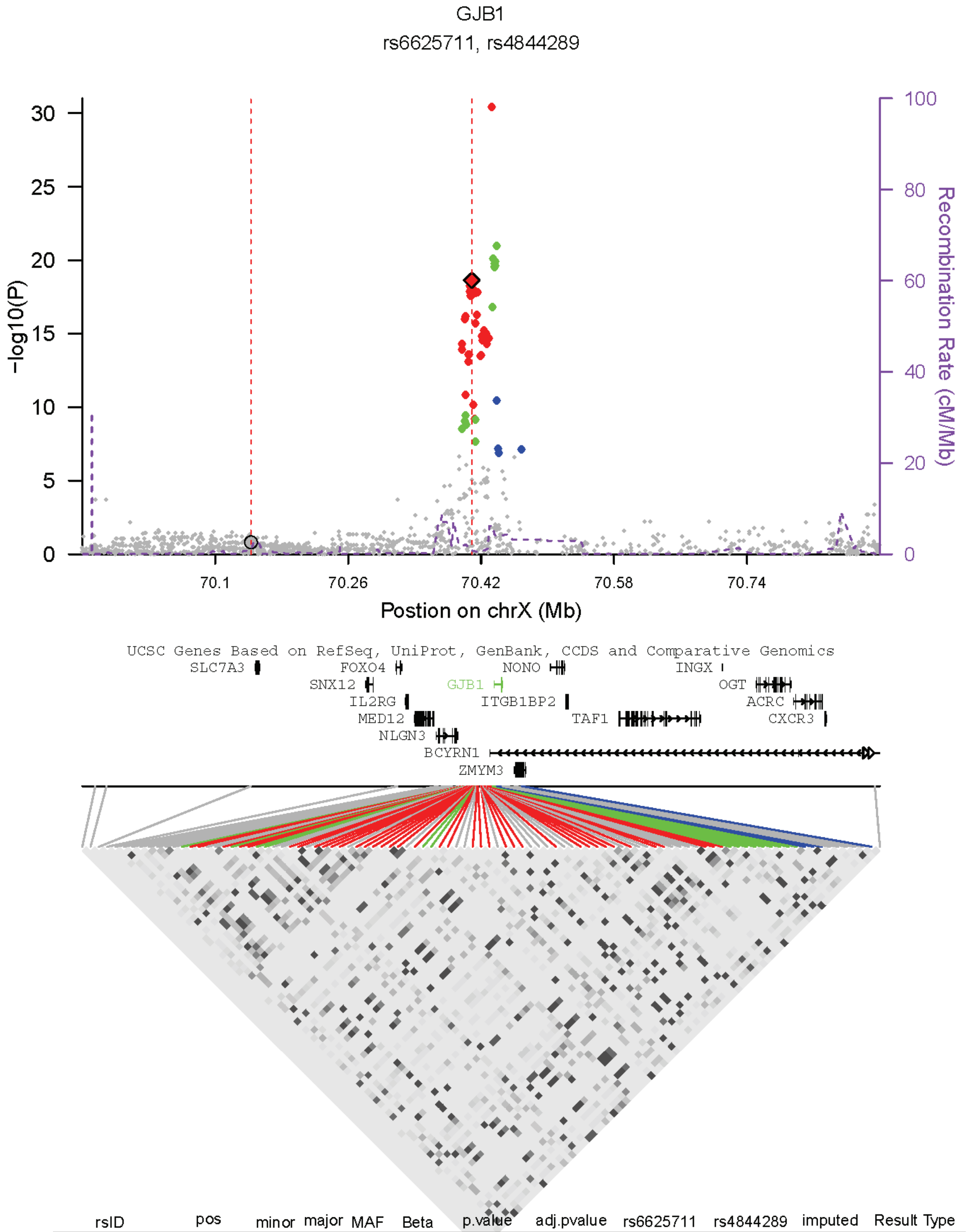

rsID	pos	minor	major	MAF	Beta	p.value	adj.pvalue	rs6625711	rs4844289	imputed	Result Type
rs6525442	70117463	G	A	0.49	−0.042	3.11e−02	0.1462	0.722	<0.2	1	MaxLD
rs6625711	70139850	A	T	0.42	0.030	1.46e−01	0.3088	1.000	<0.2	1	PC Risk
rs56232551	70396389	T	C	0.18	−0.143	3.26e−09	0.1159	<0.2	0.327	1	Flanking
rs4844289	70407983	G	A	0.39	−0.167	2.45e−19	0.1946	<0.2	1.000	1	PC Risk
rs747181	70432708	A	G	0.31	−0.221	4.01e−31	–	<0.2	0.512	0	Peak
rs184587106	70438659	A	C	0.09	−0.225	3.63e−11	0.0497	<0.2	<0.2	1	Flanking

Supplementary Figure 3 - Regional association plots for each of the genes placed in Group 2

Regional association plots display eQTL results for the target gene listed in the title and all SNPs in the target gene region. All PC risk-SNPs in the region are listed in the second line of the title. The x-axis shows the chromosomal position of the SNPs (with expressed genes in the region displayed below) and the y-axis is the –log10(p-value) obtained by regressing normalized expression levels for the target gene on the number of minor alleles of each SNP genotype adjusted for histologic characteristics and 14 expression principal components. The PC risk-SNP position is indicated by a dotted red vertical line with the eQTL result displayed as a diamond. Colored points show all Bonferroni significant results with the color defined by LD between the SNP and the PC risk-SNP (LD r^2>0.5 red, between 0.2 – 0.5 green, and ≤0.2 blue). If the region contains multiple PC risk-SNPs, the points are colored based on LD with the PC risk-SNP that is in highest LD with the eQTL peak associated SNP. The eQTL result for this PC risk-SNP is displayed as diamond, the data points for all of the other PC risk-SNPs are displayed as an open circle.

The table below the LD plot shows results for the peak-SNP (Result Type = Peak) and the most upstream and downstream SNPs (Result Type = Flanking) surpassing the Bonferroni significance threshold (P < 1.96E-07 for primary analysis and P < 3.02E-08 for second stage) as well as the top result for each PC risk SNP (Result Type = PC Risk) and, if a SNP in LD (r^2 > 0.5) with the PC risk SNP has a stronger association than the PC risk SNP (i.e., lower p-value), then the LD SNP with the strongest association is also presented (Result Type = MaxLD). Finally, if all of these SNPs have a single direction of effect, e.g., all have positive beta coefficients thus increasing gene expression, and if any Bonferroni significant results were

observed in the opposite direction, then the strongest result among the opposite direction SNPs is also presented (Result Type = ReverseBeta).

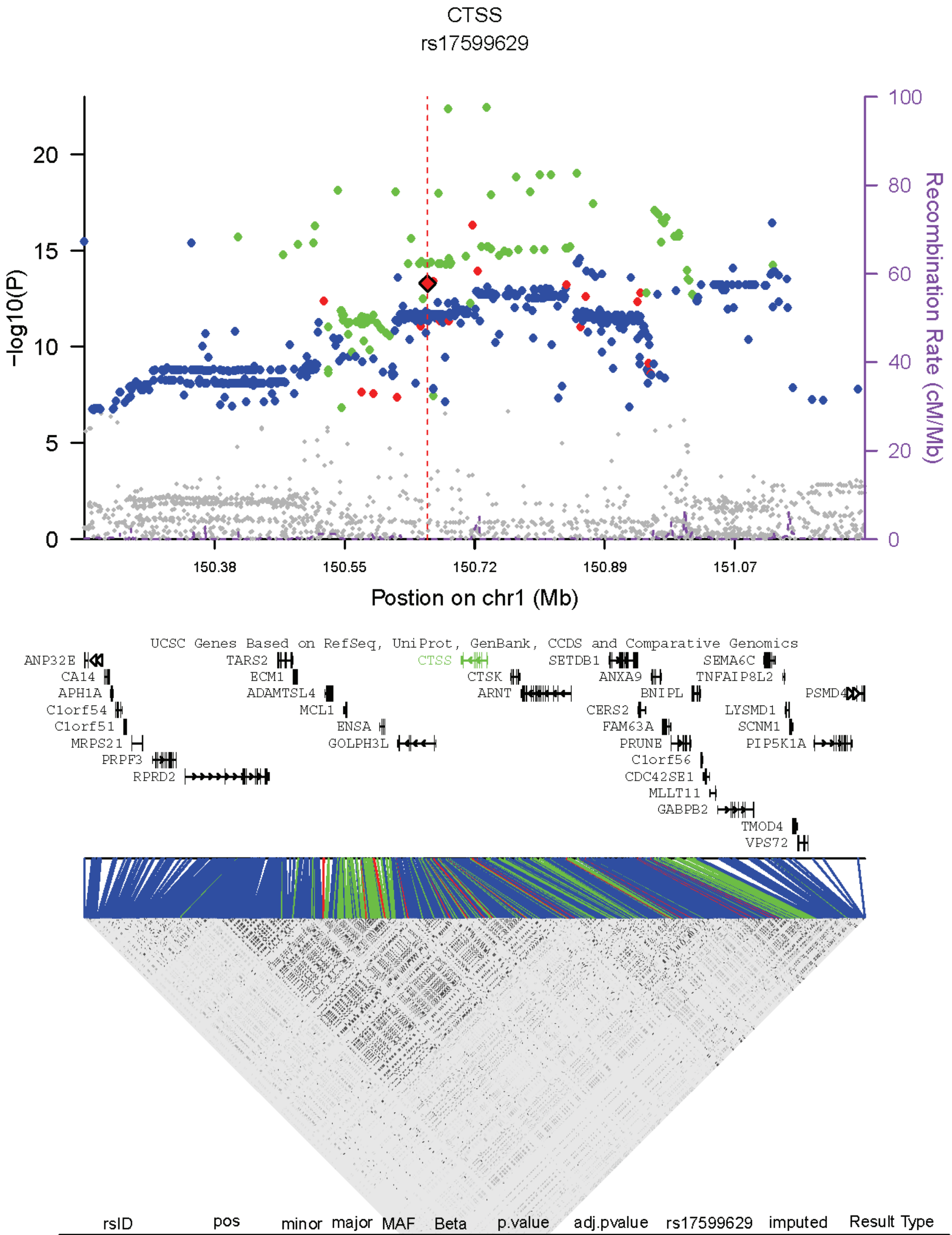

rsID	pos	minor	major	MAF	Beta	p.value	adj.pvalue	rs17599629	imputed	Result Type
rs143192984	149758064	A	G	0.08	−0.207	3.43e−09	3.07e−02	<0.2	1	Flanking
rs17599629	150658287	G	A	0.25	−0.143	5.18e−14	1.87e−02	1.000	0	PC Risk
rs75056606	150718602	G	T	0.18	−0.174	4.94e−17	3.58e−02	0.636	0	MaxLD
rs12568757	150729793	G	A	0.42	0.130	6.02e−16	1.25e−10	0.206	0	ReverseBeta
rs41271951	150737220	G	A	0.11	−0.252	3.70e−23	−	0.345	1	Peak
rs78685532	150738197	C	T	0.42	0.130	6.02e−16	1.25e−10	0.206	0	ReverseBeta
rs7534124	150739073	C	T	0.42	0.130	6.02e−16	1.25e−10	0.206	1	ReverseBeta
rs34834099	151502427	T	C	0.07	−0.198	9.37e−09	1.20e−02	<0.2	1	Flanking

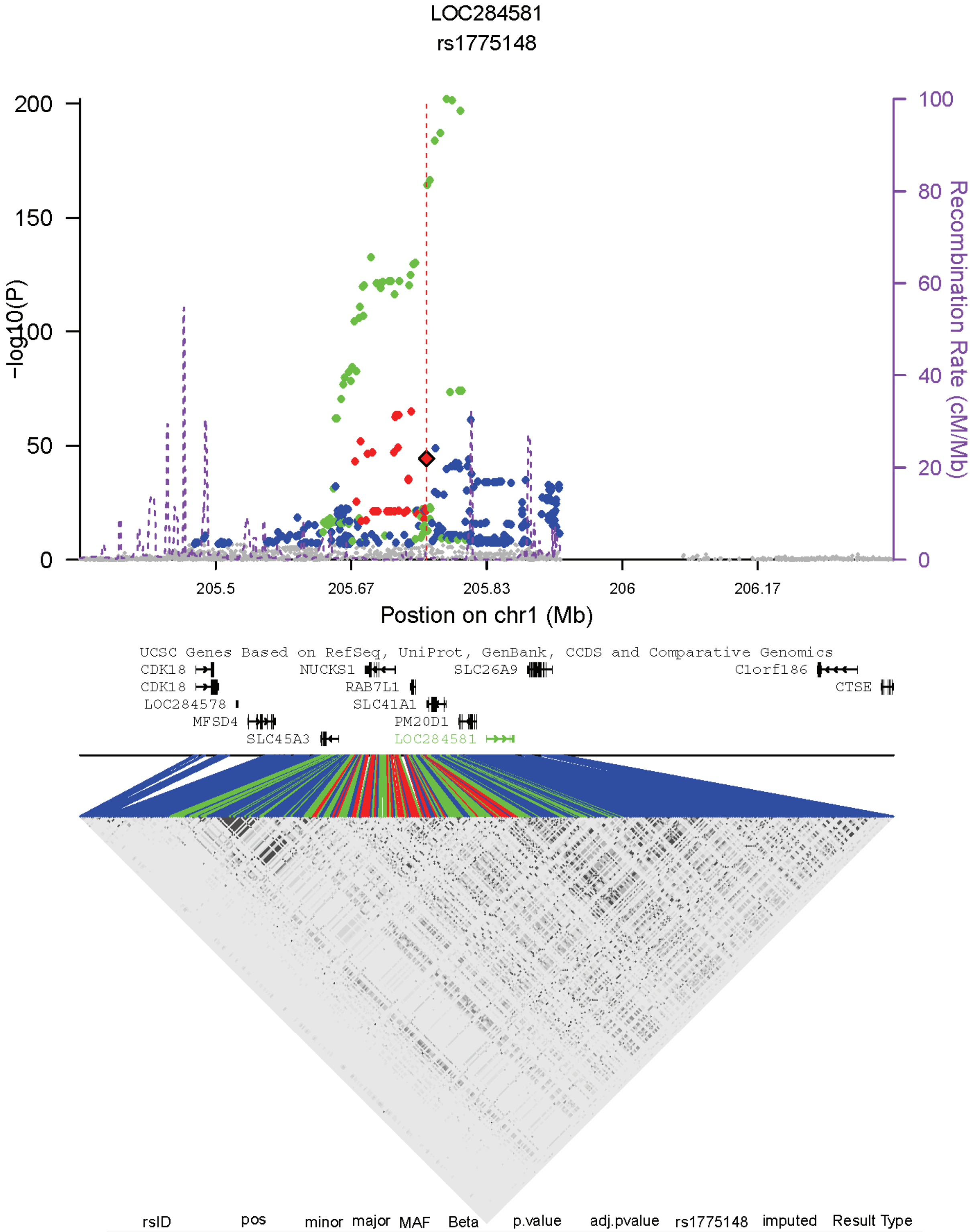

rsID	pos	minor	major	MAF	Beta	p.value	adj.pvalue	rs1775148	imputed	Result Type
rs77694338	205473772	C	T	0.27	0.573	2.41e−08	0.82382	<0.2	1	Flanking
rs708723	205739266	C	T	0.49	−1.261	1.62e−65	0.58505	0.678	0	MaxLD
rs1775148	205757824	C	T	0.43	−1.126	4.77e−45	0.57327	1.000	1	PC Risk
rs9438393	205782718	G	A	0.38	1.735	1.72e−202	−	0.363	0	Peak
rs7413698	205921859	G	A	0.45	−0.589	3.60e−12	0.00878	<0.2	0	Flanking

GDF7
rs13385191

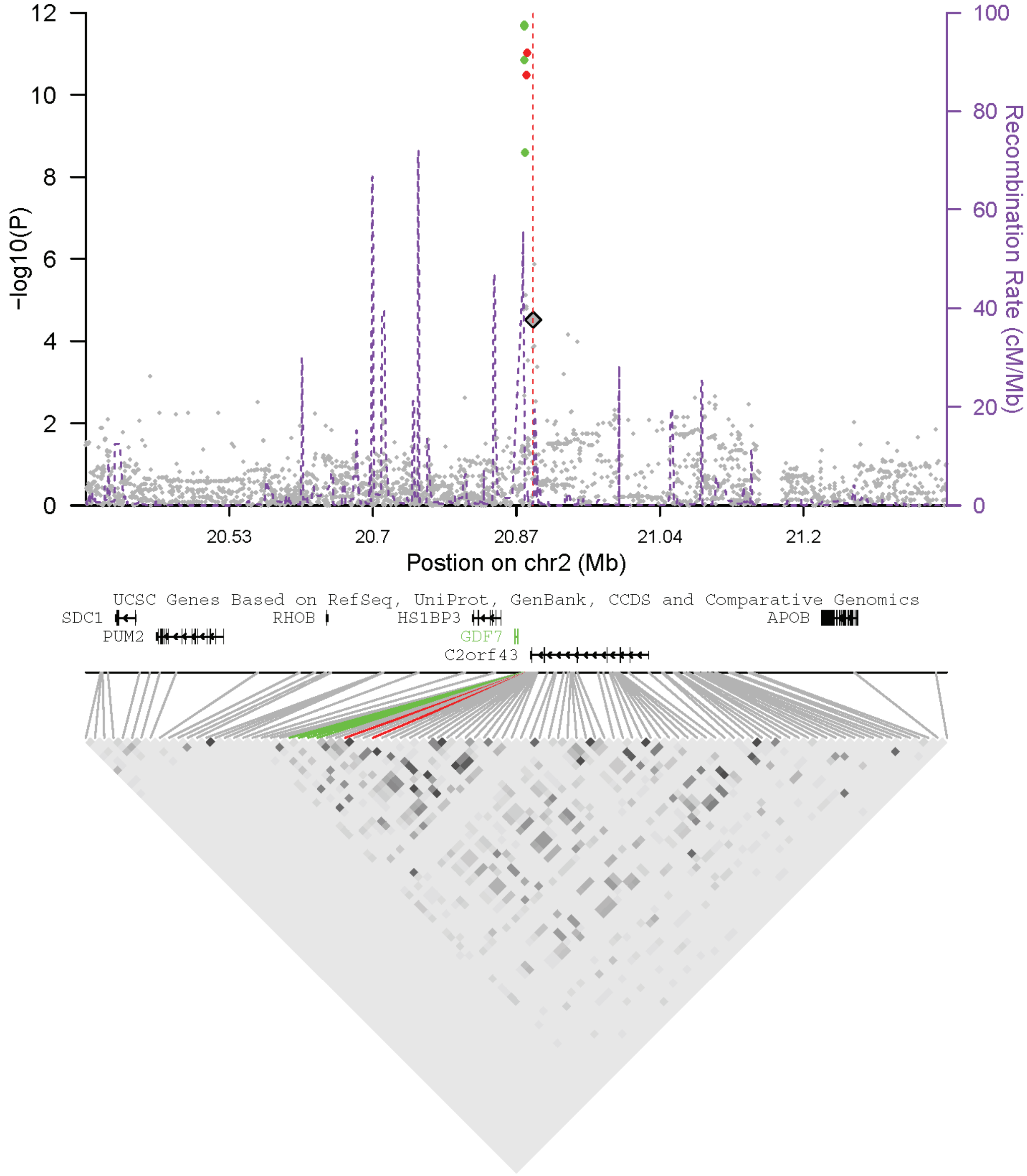

rsID	pos	minor	major	MAF	Beta	p.value	adj.pvalue	rs13385191	imputed	Result Type
rs9306894	20878105	G	A	0.37	−0.181	2.12e−12	0.531	0.479	1	Flanking
rs9306895	20878153	C	T	0.37	−0.181	2.03e−12	−	0.479	1	Peak
rs2289081	20881840	C	G	0.36	−0.173	9.42e−12	0.594	0.547	1	MaxLD
rs13385191	20888265	G	A	0.24	−0.120	3.07e−05	0.417	1.000	1	PC Risk

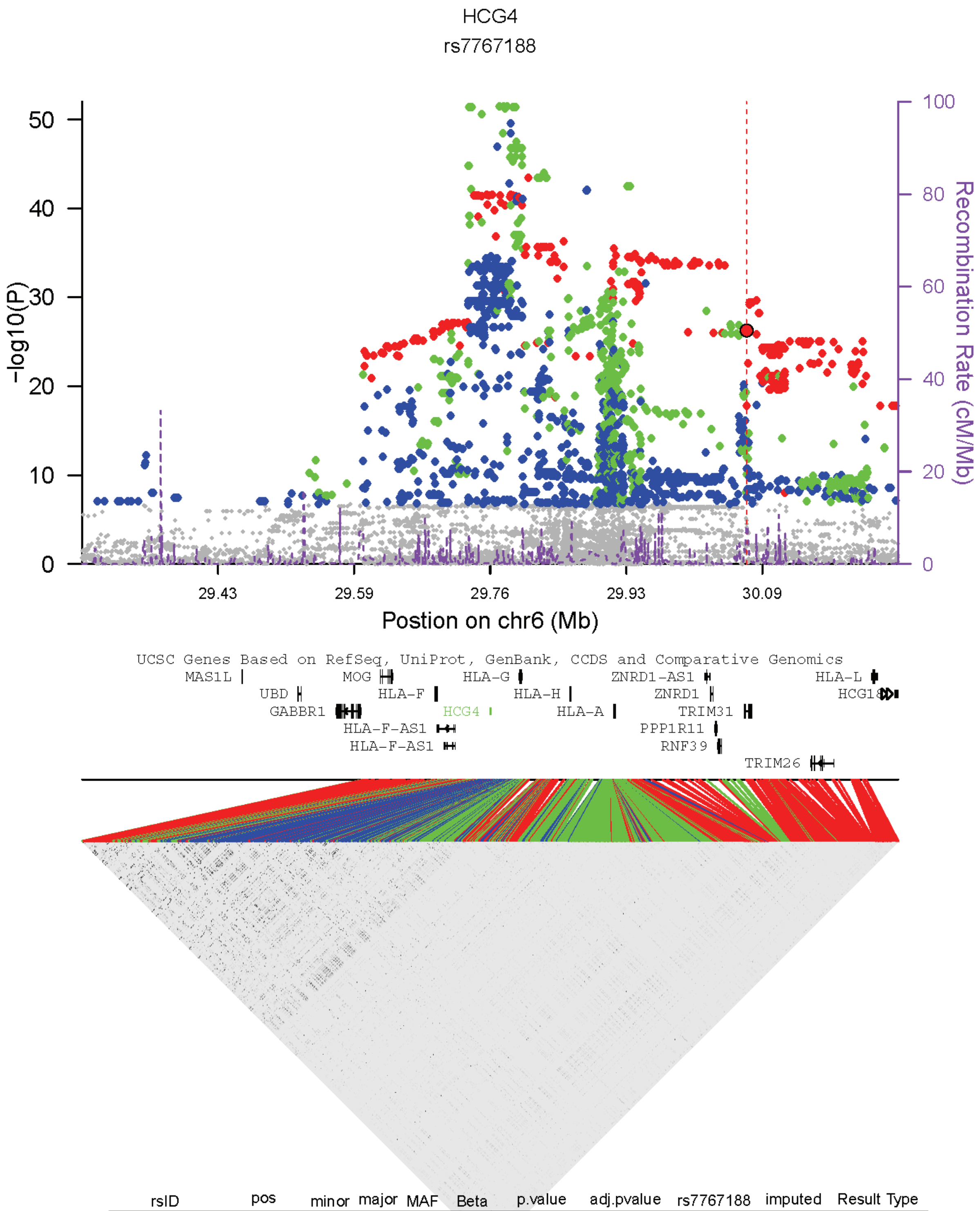

rsID	pos	minor	major	MAF	Beta	p.value	adj.pvalue	rs7767188	imputed	Result Type
rs116556815	28714044	G	C	0.15	−0.261	1.60e−08	0.032818	<0.2	1	Flanking
rs114651782	29771298	T	A	0.38	0.476	3.73e−52	−	0.458	1	Peak
rs2735030	29775950	C	A	0.38	0.476	3.73e−52	0.218861	0.458	0	Peak
rs114865932	29781011	T	A	0.38	0.476	3.73e−52	0.218861	0.458	1	Peak
rs148465275	29806786	T	A	0.34	0.457	3.66e−44	0.549773	0.510	1	MaxLD
rs7767188	30073776	A	G	0.24	0.404	5.28e−27	0.078225	1.000	0	PC Risk
rs148642004	30838043	C	G	0.19	−0.240	2.45e−08	0.000527	<0.2	1	Flanking

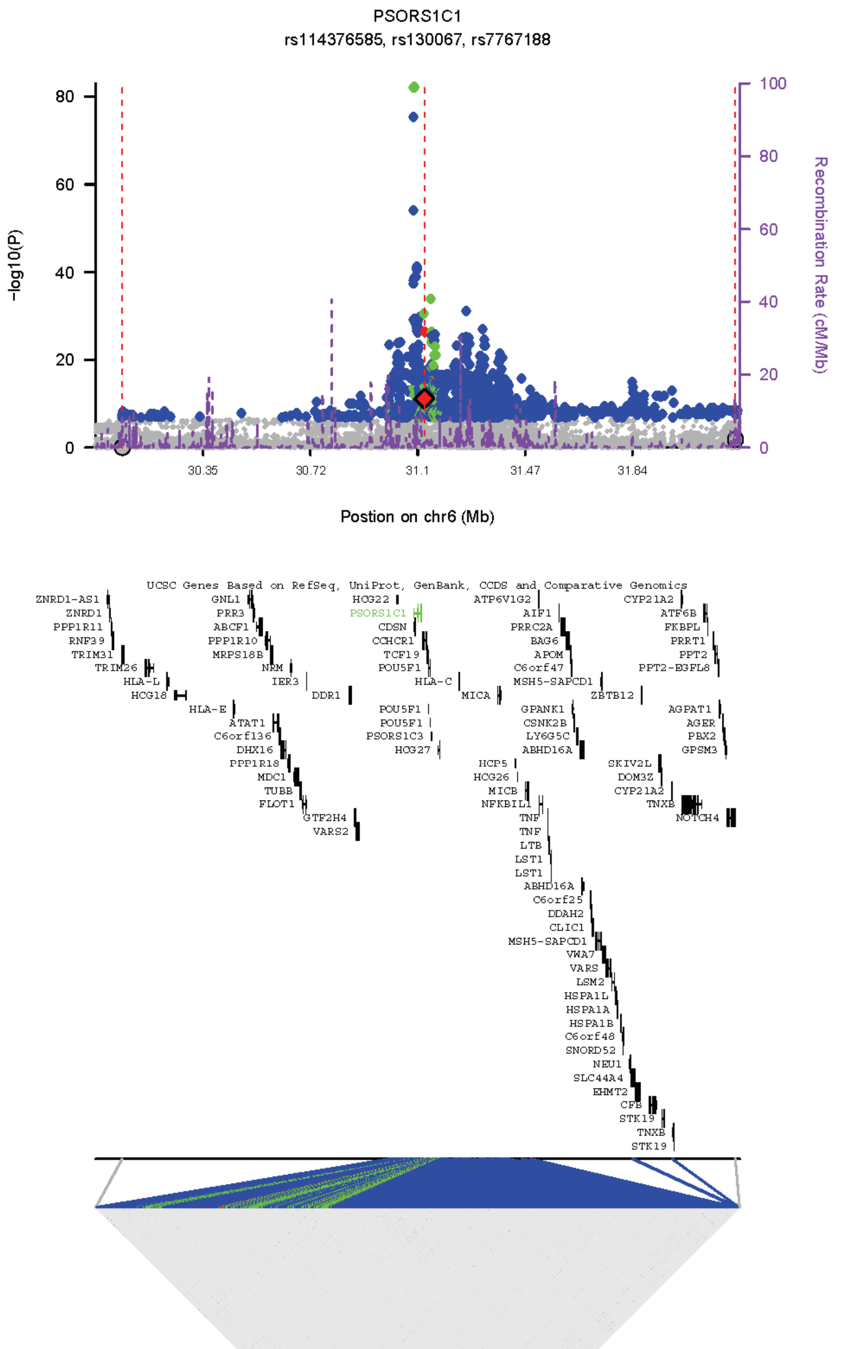

rsID	pos	minor	major	MAF	Beta	p.value	adj.pvalue	rs7767188	rs130067	rs114376585	imputed	Result Type
rs7767188	30073776	A	G	0.24	0.024	7.28e−01	0.039248	1.000	<0.2	<0.2	0	PC Risk
rs116104370	30076660	A	G	0.15	0.440	6.52e−09	0.000233	<0.2	<0.2	<0.2	0	Flanking
rs2022065	30121460	T	C	0.34	0.116	5.59e−02	0.912315	0.506	<0.2	<0.2	0	MaxLD
rs3130987	31085641	T	C	0.49	0.952	8.24e−83	–	<0.2	0.245	<0.2	1	Peak
rs130067	31118511	G	T	0.21	0.481	7.55e−12	0.001884	<0.2	1.000	<0.2	0	PC Risk
rs1265109	31119589	T	G	0.28	0.670	6.56e−27	0.044088	<0.2	0.658	<0.2	0	MaxLD
rs114376585	32192331	A	G	0.38	−0.149	1.05e−02	0.508667	<0.2	<0.2	1.000	0	PC Risk
rs116522847	32197219	G	T	0.45	0.227	6.10e−05	0.806020	<0.2	<0.2	0.516	1	MaxLD
rs142377682	32202086	T	G	0.15	−0.468	4.63e−09	0.171923	<0.2	<0.2	0.252	1	Flanking

PSORS1C3

rs114376585, rs130067, rs7767188

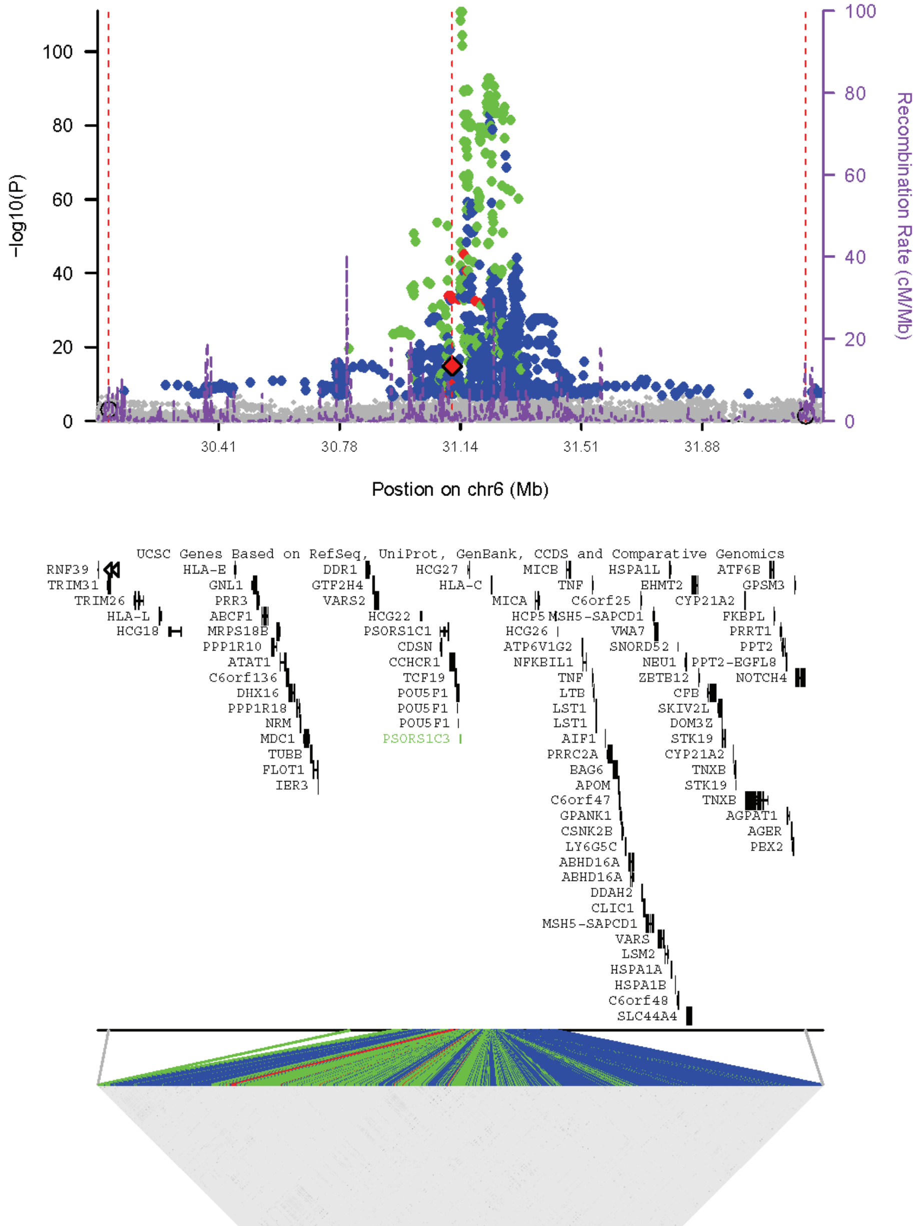

rsID	pos	minor	major	MAF	Beta	p.value	adj.pvalue	rs7767188	rs130067	rs114376585	imputed	Result Type
rs7767188	30073776	A	G	0.24	0.342	6.32e−04	6.61e−01	1.000	<0.2	<0.2	0	PC Risk
rs9261438	30089280	T	C	0.32	0.445	4.35e−07	4.70e−01	0.554	<0.2	<0.2	0	MaxLD
rs116109250	30090337	A	G	0.32	0.445	4.35e−07	4.70e−01	0.554	<0.2	<0.2	1	MaxLD
rs116609891	30122978	A	G	0.03	1.902	7.09e−09	2.25e−01	<0.2	<0.2	<0.2	1	Flanking
rs1265095	31106643	A	G	0.49	−0.818	3.67e−23	1.33e−01	<0.2	<0.2	<0.2	0	ReverseBeta
rs130067	31118511	G	T	0.21	0.814	1.36e−15	5.32e−06	<0.2	1.000	<0.2	0	PC Risk
rs115680823	31150873	C	T	0.22	1.814	1.86e−111	–	<0.2	0.320	<0.2	1	Peak
rs144721865	31156041	T	C	0.28	1.224	1.23e−45	1.38e−03	<0.2	0.637	<0.2	1	MaxLD
rs114376585	32192331	A	G	0.38	0.186	2.93e−02	5.42e−01	<0.2	<0.2	1.000	0	PC Risk
rs9267853	32195251	T	C	0.38	0.189	2.71e−02	5.56e−01	<0.2	<0.2	0.997	0	MaxLD
rs2022539	32239558	C	A	0.39	0.486	2.27e−08	1.44e−02	<0.2	<0.2	<0.2	0	Flanking

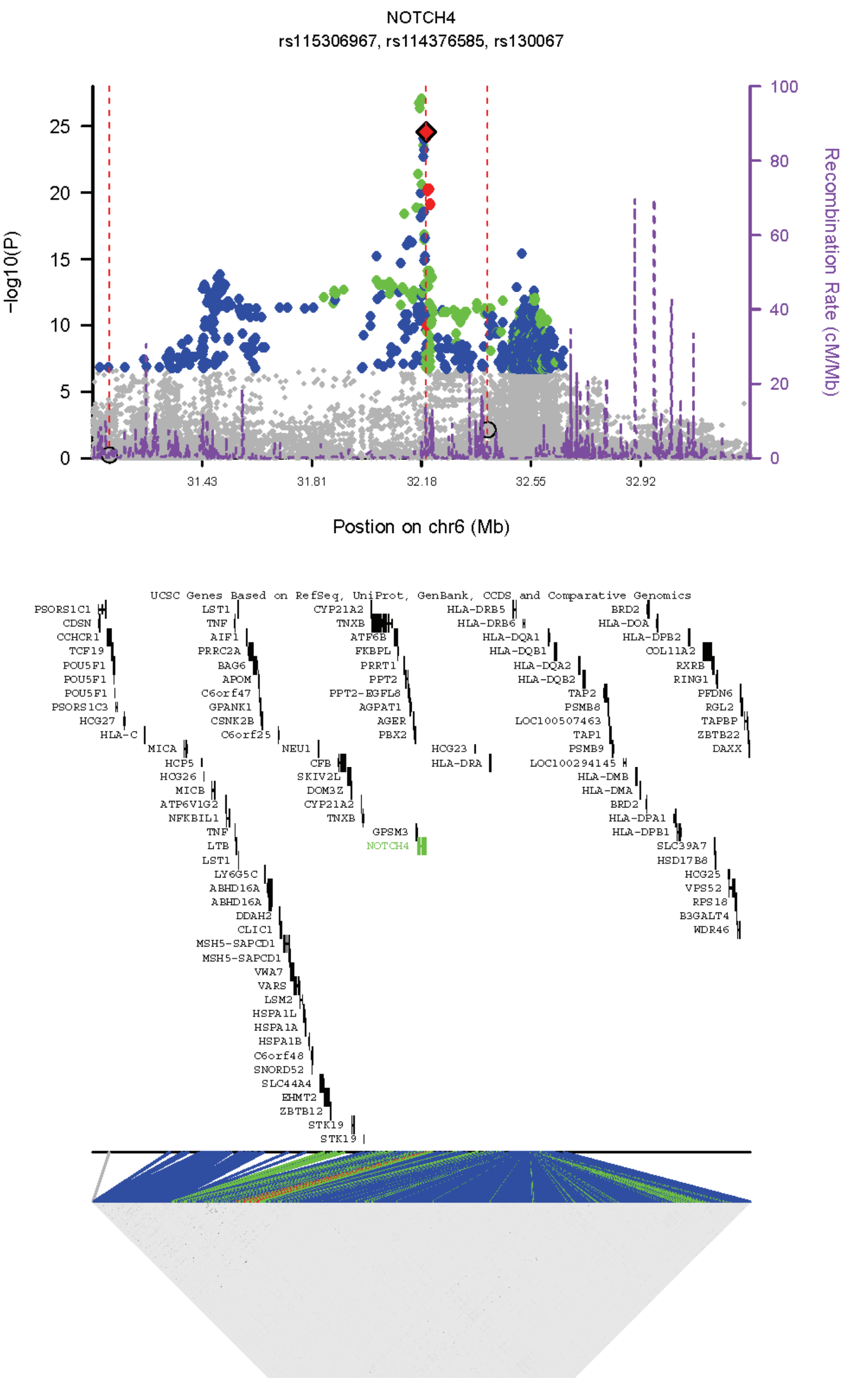

rsID	pos	minor	major	MAF	Beta	p.value	adj.pvalue	rs130067	rs114376585	rs115306967	imputed	Result Type
rs1265093	31107187	A	G	0.29	0.027	1.25e−01	1.87e−01	0.639	<0.2	<0.2	0	MaxLD
rs130067	31118511	G	T	0.21	0.012	5.38e−01	3.97e−01	1.000	<0.2	<0.2	0	PC Risk
rs113635367	31313378	A	G	0.19	0.111	2.72e−08	3.89e−03	<0.2	<0.2	<0.2	1	Flanking
rs138007308	32178773	T	C	0.50	0.160	8.75e−28	–	<0.2	0.244	<0.2	1	Peak
rs114376585	32192331	A	G	0.38	0.157	2.69e−25	1.11e−10	<0.2	1.000	<0.2	0	PC Risk
rs115306967	32400939	C	G	0.28	−0.048	6.74e−03	7.66e−01	<0.2	<0.2	1.000	1	PC Risk
rs3135392	32409242	A	C	0.39	−0.080	6.79e−07	2.16e−01	<0.2	<0.2	0.522	0	MaxLD
rs3129718	32659838	T	C	0.15	0.125	8.14e−09	6.38e−02	<0.2	<0.2	<0.2	0	Flanking

SLC22A2
rs651164, rs9364554

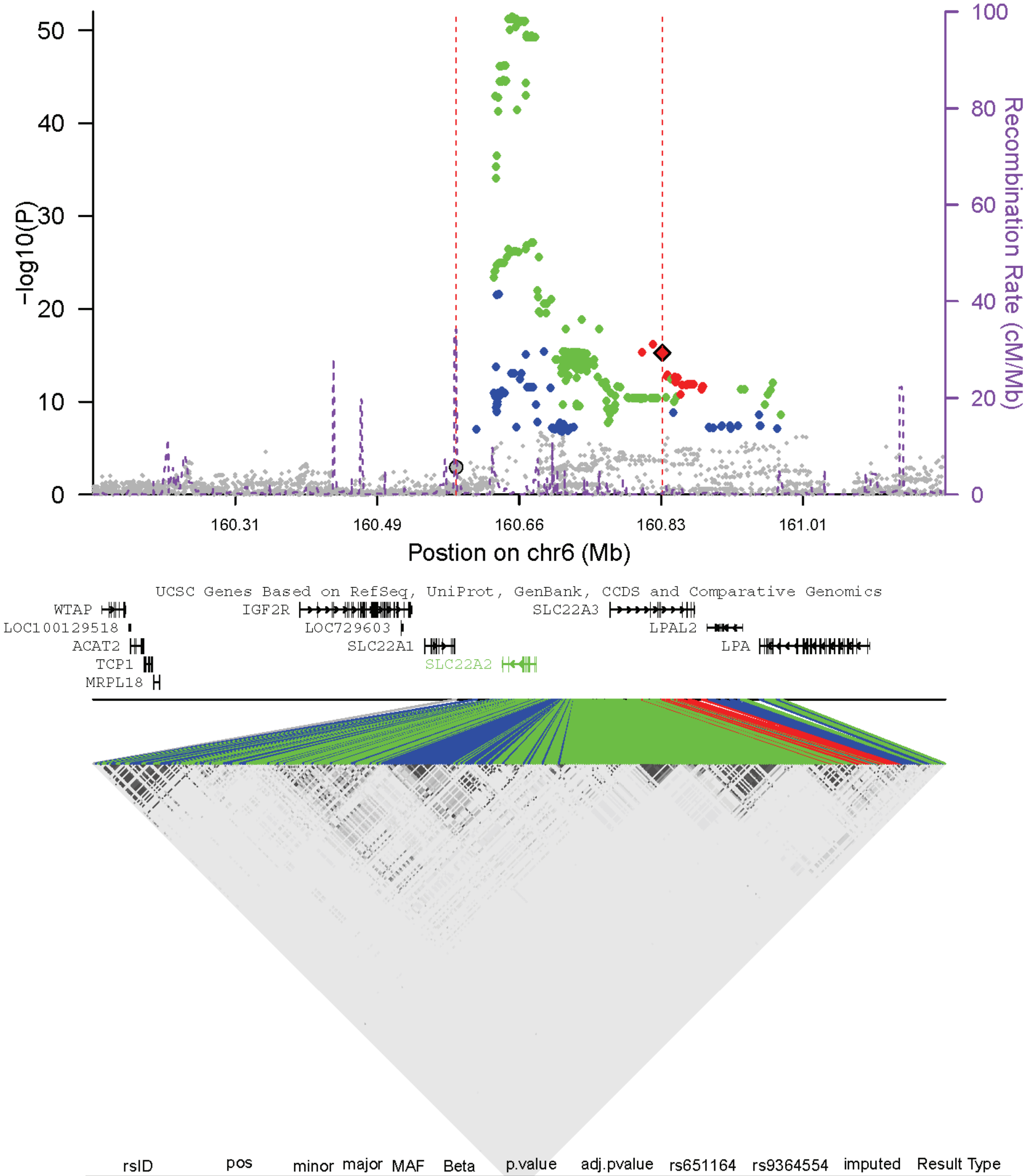

rsID	pos	minor	major	MAF	Beta	p.value	adj.pvalue	rs651164	rs9364554	imputed	Result Type
rs651164	160581374	A	G	0.31	0.170	1.02e−03	0.885	1.000	<0.2	1	PC Risk
rs4123902	160628053	T	A	0.12	−0.605	4.73e−24	0.250	<0.2	0.221	1	Flanking
rs582158	160649864	C	A	0.24	−0.673	4.03e−52	–	<0.2	0.274	1	Peak
rs3105751	160822675	G	A	0.31	−0.362	7.33e−17	0.247	<0.2	0.975	1	MaxLD
rs9364554	160833664	T	C	0.32	−0.347	5.51e−16	0.303	<0.2	1.000	0	PC Risk
rs12210186	160978997	G	A	0.13	−0.377	2.49e−09	0.188	<0.2	0.232	1	Flanking

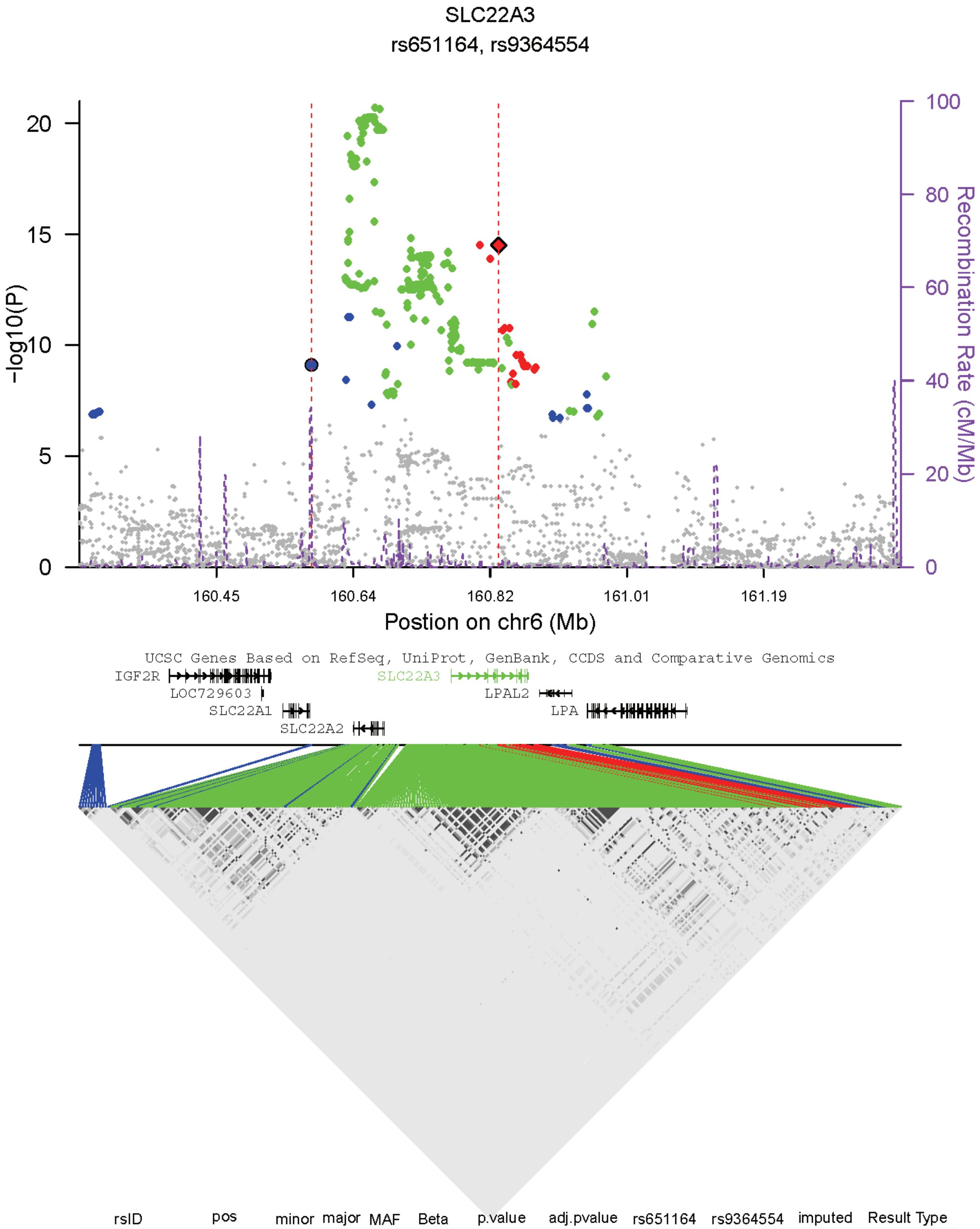

rsID	pos	minor	major	MAF	Beta	p.value	adj.pvalue	rs651164	rs9364554	imputed	Result Type
rs651164	160581374	A	G	0.31	0.302	7.58e−10	2.08e−05	1.000	<0.2	1	PC Risk
rs316021	160668041	C	T	0.24	−0.435	2.03e−21	–	<0.2	0.280	0	Peak
rs9364554	160833664	T	C	0.32	−0.325	3.06e−15	3.36e−04	<0.2	1.000	0	PC Risk
rs1112444	160835192	A	C	0.32	−0.325	2.89e−15	3.29e−04	<0.2	1.000	1	MaxLD
rs12210186	160978997	G	A	0.13	−0.361	2.71e−09	5.08e−03	<0.2	0.232	1	Flanking

AGAP7
rs3123078, rs10993994

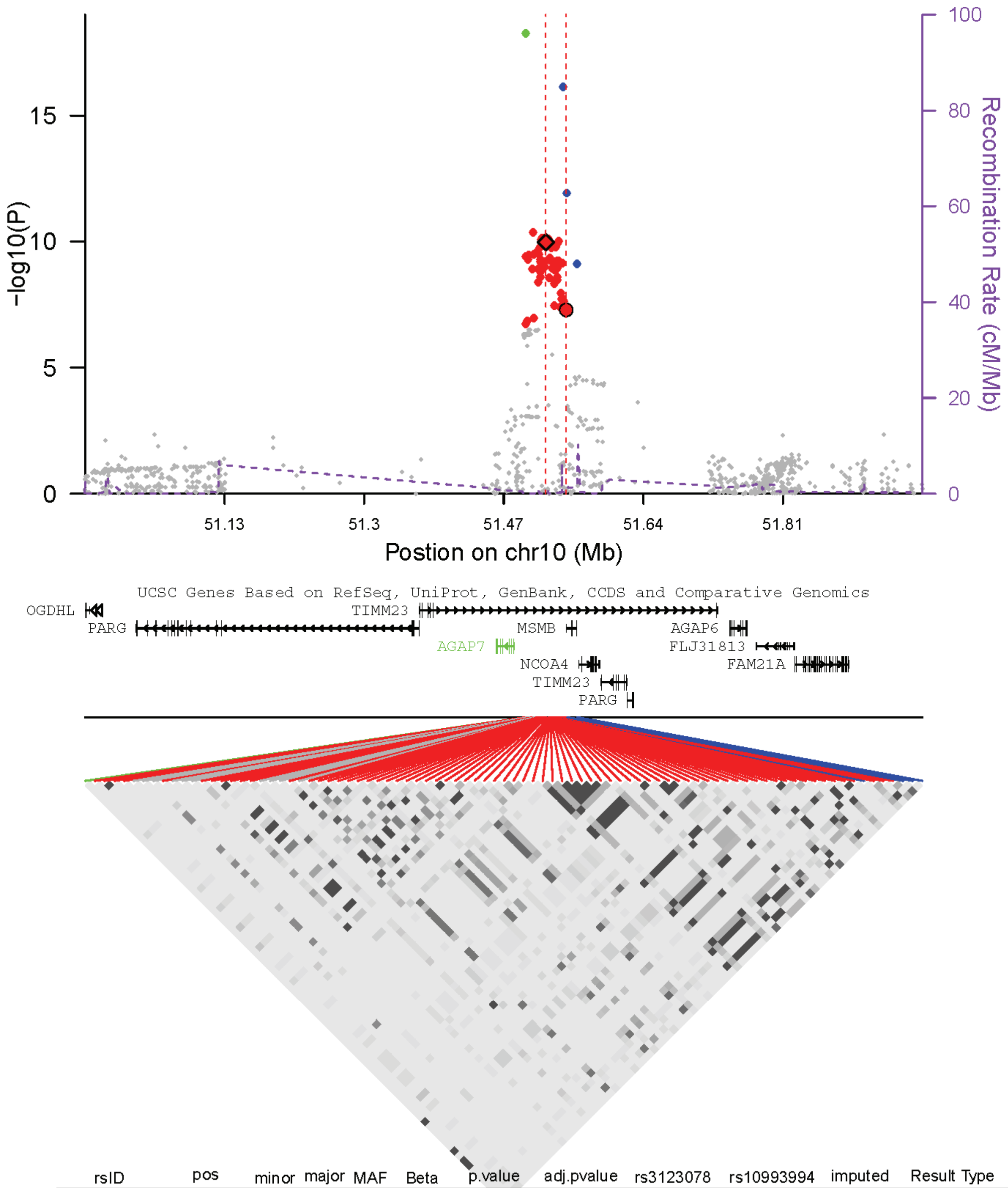

rsID	pos	minor	major	MAF	Beta	p.value	adj.pvalue	rs3123078	rs10993994	imputed	Result Type
rs12777595	51500625	C	G	0.15	0.478	5.70e−19	–	0.219	<0.2	1	Peak
rs61847060	51510203	A	G	0.46	0.252	4.36e−11	0.00410	0.939	0.743	1	MaxLD
rs3123078	51524971	C	T	0.48	0.242	1.05e−10	0.00440	1.000	0.788	0	PC Risk
rs28416634	51539271	A	G	0.49	−0.232	9.22e−10	0.00991	0.905	0.727	1	ReverseBeta
rs10993994	51549496	T	C	0.42	0.207	4.99e−08	0.04381	0.788	1.000	0	PC Risk
rs78267136	51563352	T	C	0.08	0.397	7.66e−10	0.15179	<0.2	<0.2	1	Flanking

C10orf32
rs3850699

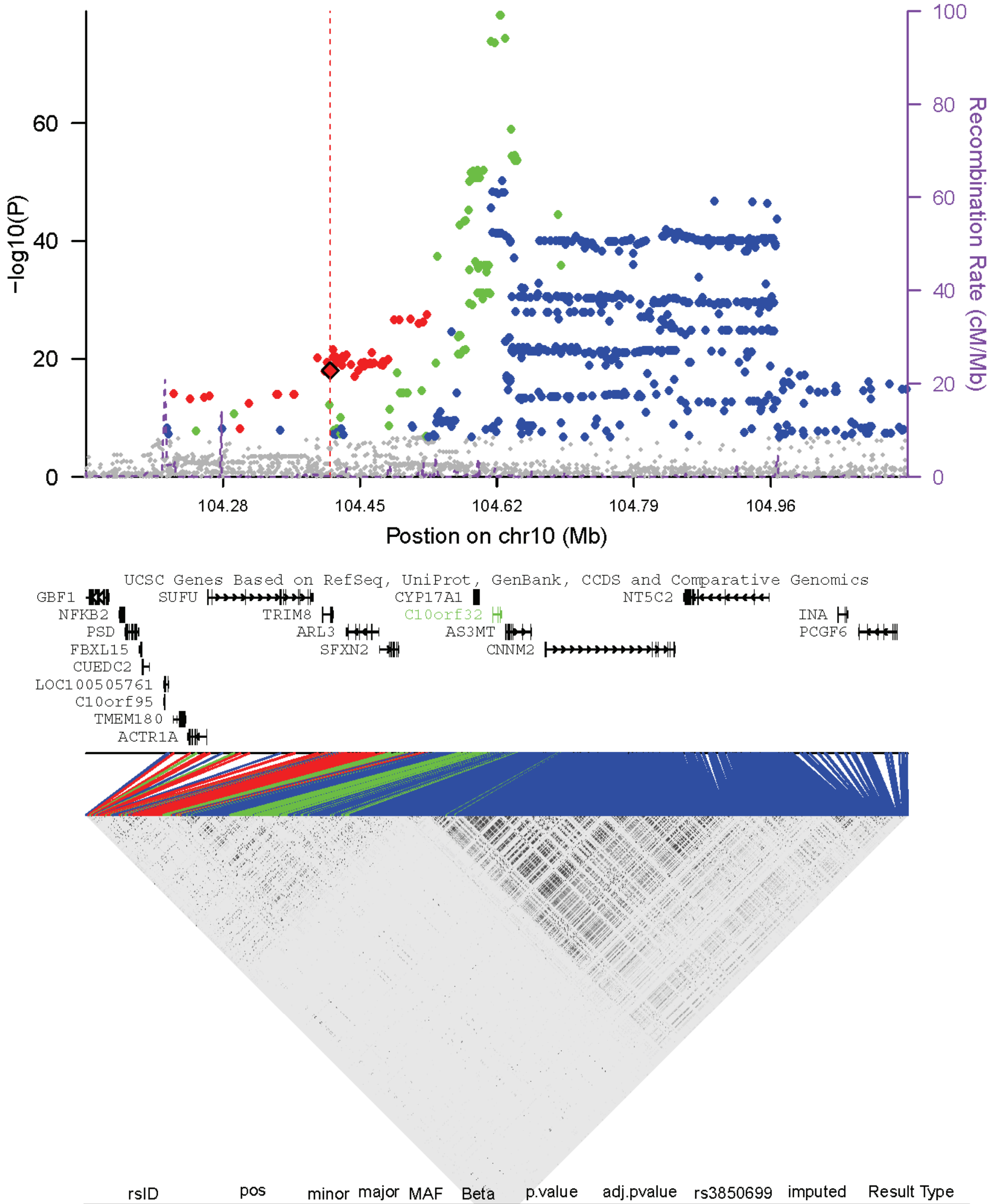

rsID	pos	minor	major	MAF	Beta	p.value	adj.pvalue	rs3850699	imputed	Result Type
rs150098322	104214364	C	T	0.09	−0.104	6.26e−09	0.872	<0.2	1	Flanking
rs3850699	104414221	G	A	0.27	−0.094	9.75e−19	0.694	1.000	0	PC Risk
rs12776506	104532828	G	A	0.29	−0.107	2.98e−28	0.788	0.603	1	MaxLD
rs9527	104623578	T	C	0.22	−0.174	4.58e−79	–	0.288	0	Peak
rs7090337	104775483	A	G	0.42	0.074	4.25e−15	0.739	<0.2	1	ReverseBeta
rs2863733	105244565	G	A	0.30	−0.060	1.43e−08	0.856	<0.2	0	Flanking

TUBA1B
rs10875943

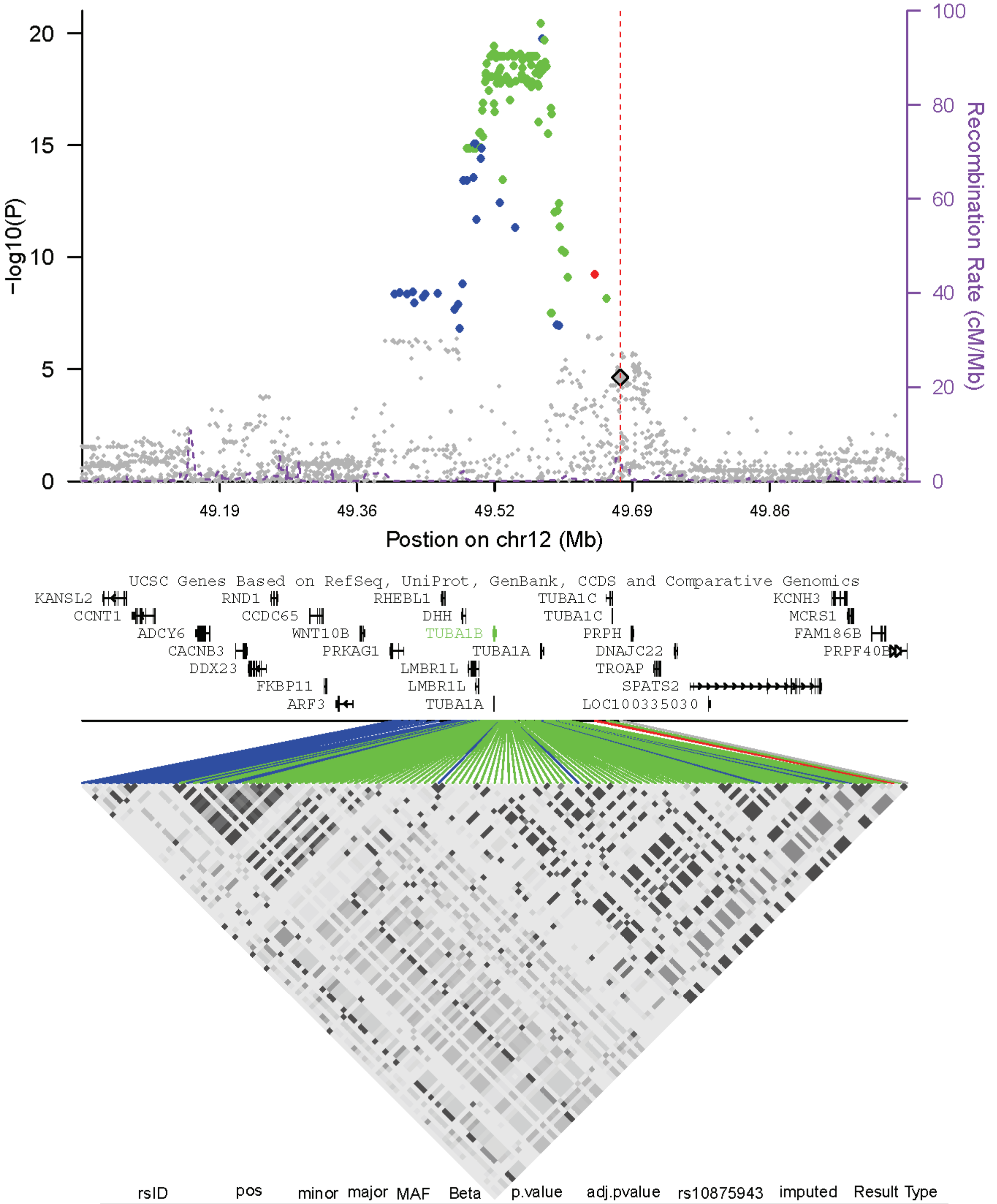

rsID	pos	minor	major	MAF	Beta	p.value	adj.pvalue	rs10875943	imputed	Result Type
rs10875910	49402393	C	G	0.39	−0.072	4.64e−09	0.8800	<0.2	1	Flanking
rs1056875	49580180	C	T	0.34	−0.125	3.79e−21	–	0.216	1	Peak
rs11168936	49645240	C	T	0.38	−0.079	6.13e−10	0.0818	0.507	0	MaxLD
rs10875940	49659756	A	C	0.35	−0.087	7.31e−09	0.1228	0.307	1	Flanking
rs10875943	49676010	C	T	0.30	−0.058	2.36e−05	0.6458	1.000	1	PC Risk

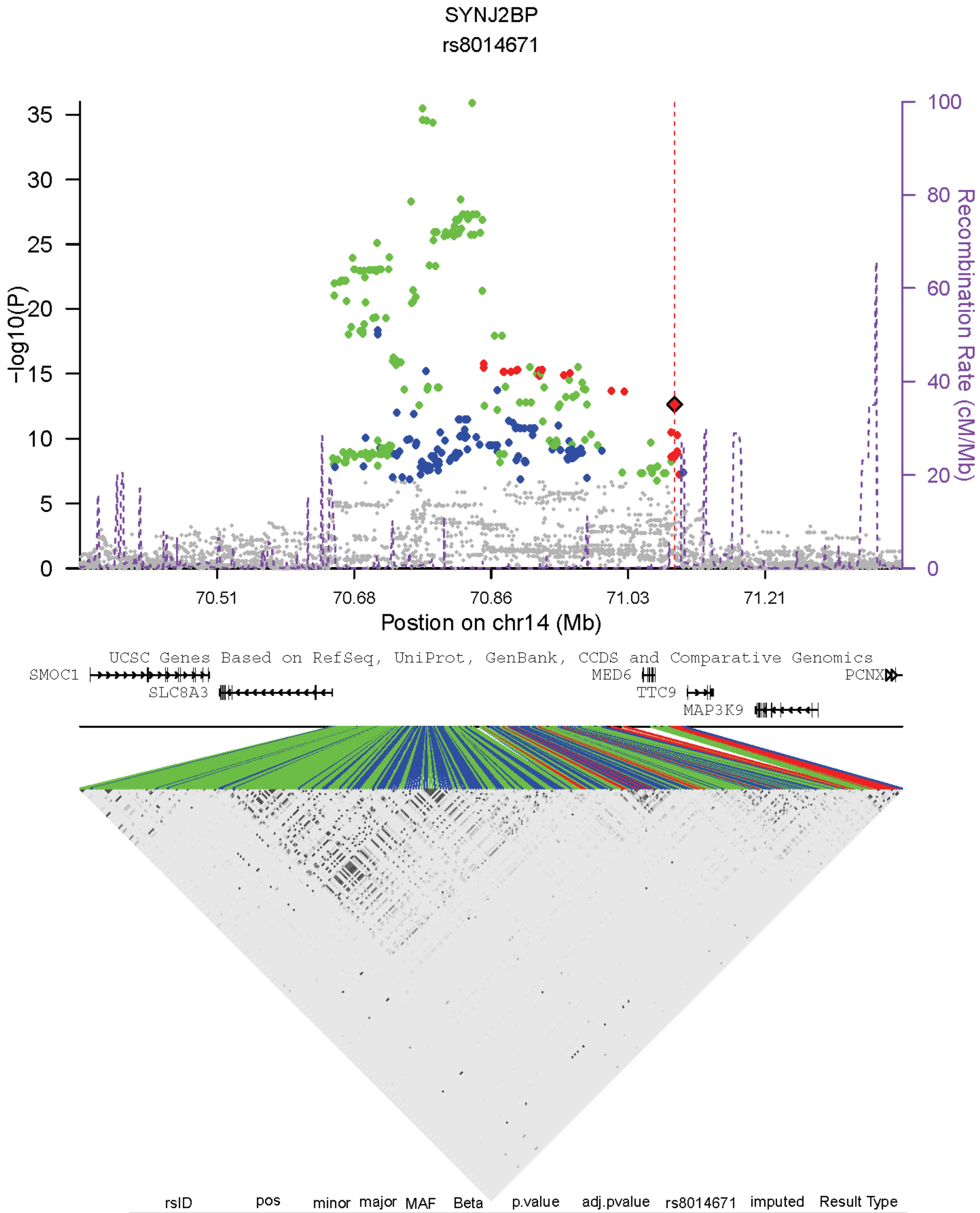

rsID	pos	minor	major	MAF	Beta	p.value	adj.pvalue	rs8014671	imputed	Result Type
rs12882693	70656838	G	A	0.32	−0.041	3.54e−09	0.485	0.259	1	Flanking
rs1044527	70833819	G	C	0.45	0.082	1.26e−36	−	0.434	1	Peak
rs11626414	70848680	A	G	0.44	0.055	1.67e−16	0.273	0.509	1	MaxLD
rs8014671	71092256	A	G	0.41	−0.050	2.34e−13	0.701	1.000	0	PC Risk
rs7145863	71098523	G	A	0.29	−0.040	6.30e−08	0.833	0.555	1	Flanking

MARVELD3
rs12051443

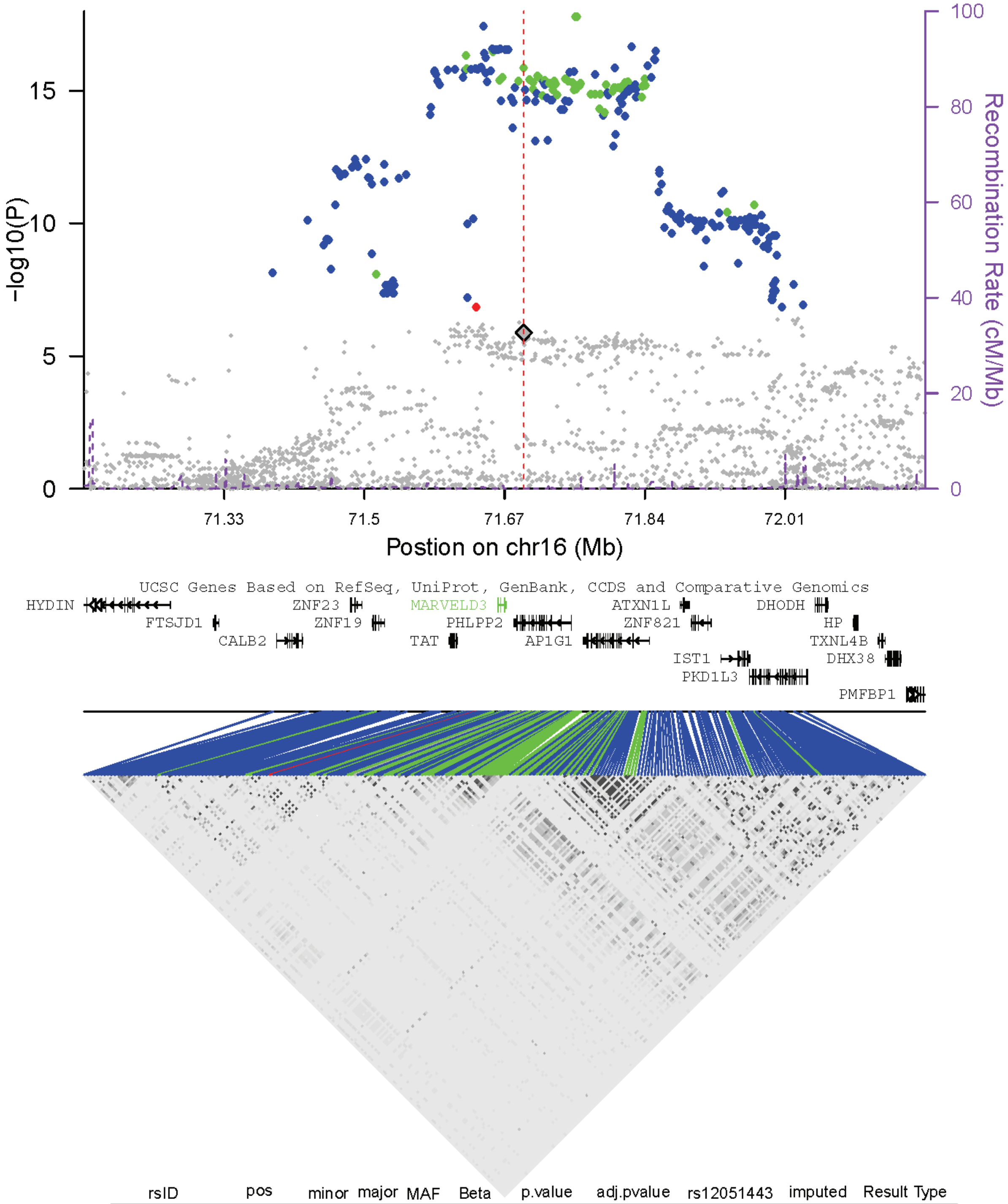

rsID	pos	minor	major	MAF	Beta	p.value	adj.pvalue	rs12051443	imputed	Result Type
rs73588051	71388627	G	A	0.11	0.100	7.28e−09	0.00482	<0.2	1	Flanking
rs9940142	71633873	G	A	0.38	−0.059	1.43e−07	0.66085	0.791	1	MaxLD
rs12051443	71691329	A	G	0.34	−0.054	1.32e−06	0.67773	1.000	1	PC Risk
rs11639902	71755852	C	T	0.37	0.095	1.65e−18	–	0.283	0	Peak
rs36114735	72017683	T	C	0.13	0.091	2.08e−08	0.01166	<0.2	1	Flanking

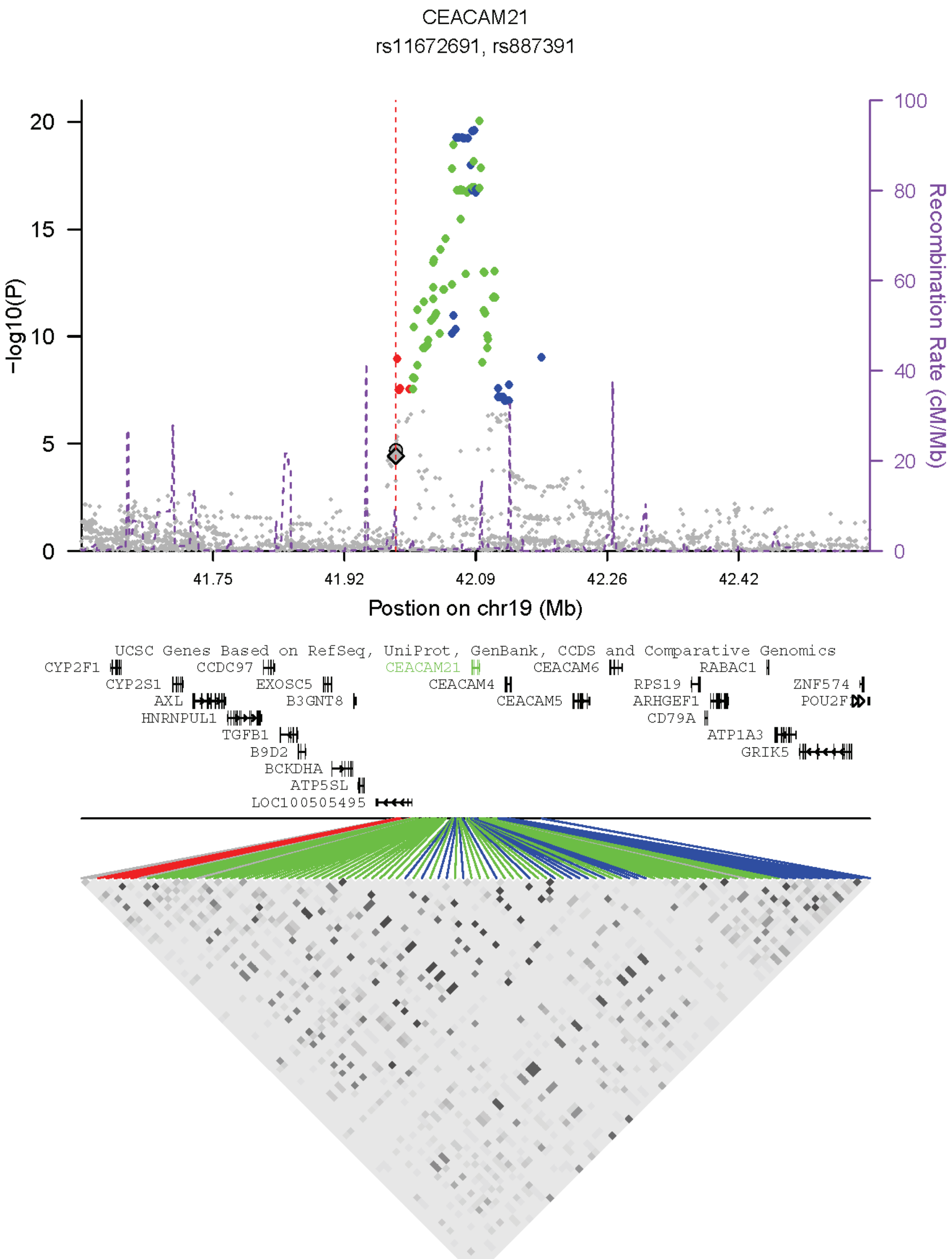

rsID	pos	minor	major	MAF	Beta	p.value	adj.pvalue	rs11672691	rs887391	imputed	Result Type
rs11672691	41985587	A	G	0.25	−0.148	1.97e−05	0.6040	1.000	0.860	0	PC Risk
rs887391	41985624	C	T	0.22	−0.148	3.73e−05	0.7097	0.860	1.000	1	PC Risk
rs8107333	41987873	A	G	0.18	−0.245	1.10e−09	0.5151	0.578	0.679	1	MaxLD
rs3745936	42092815	T	A	0.19	−0.339	9.18e−21	−	<0.2	0.248	1	Peak
rs4803498	42172442	T	G	0.06	−0.389	9.99e−10	0.0135	<0.2	<0.2	1	Flanking

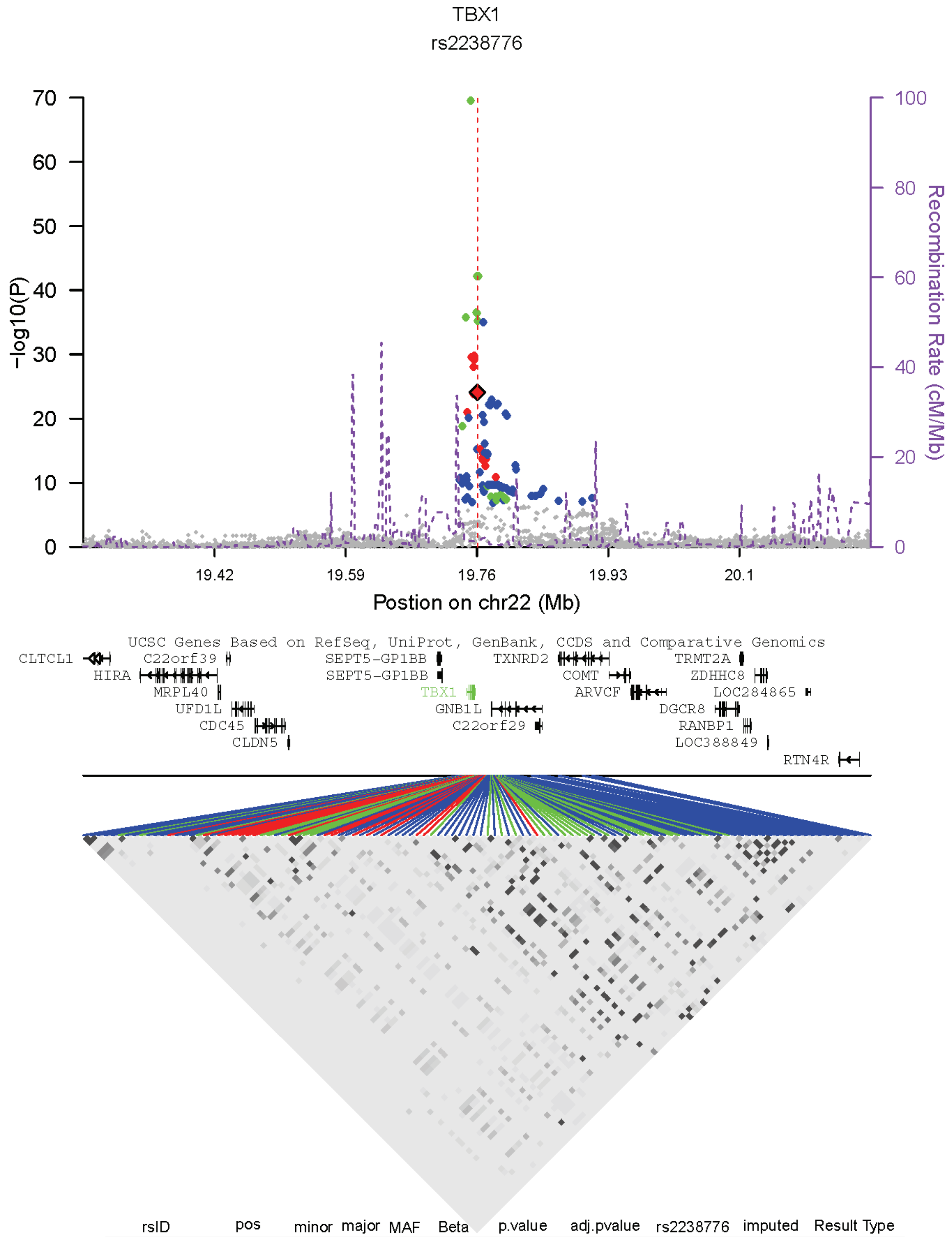

rsID	pos	minor	major	MAF	Beta	p.value	adj.pvalue	rs2238776	imputed	Result Type
rs747226	19735162	T	C	0.45	−0.302	4.02e−11	0.3576	<0.2	1	Flanking
rs1978060	19749525	A	G	0.36	0.749	3.39e−70	–	0.330	1	Peak
rs72646967	19754091	C	A	0.21	0.653	1.95e−30	0.0341	0.896	1	MaxLD
rs2238776	19757892	A	G	0.19	0.587	8.96e−25	0.0532	1.000	1	PC Risk
rs11089318	19907920	T	G	0.15	0.375	2.30e−08	0.1551	<0.2	1	Flanking

SLC7A3

rs6625711, rs4844289

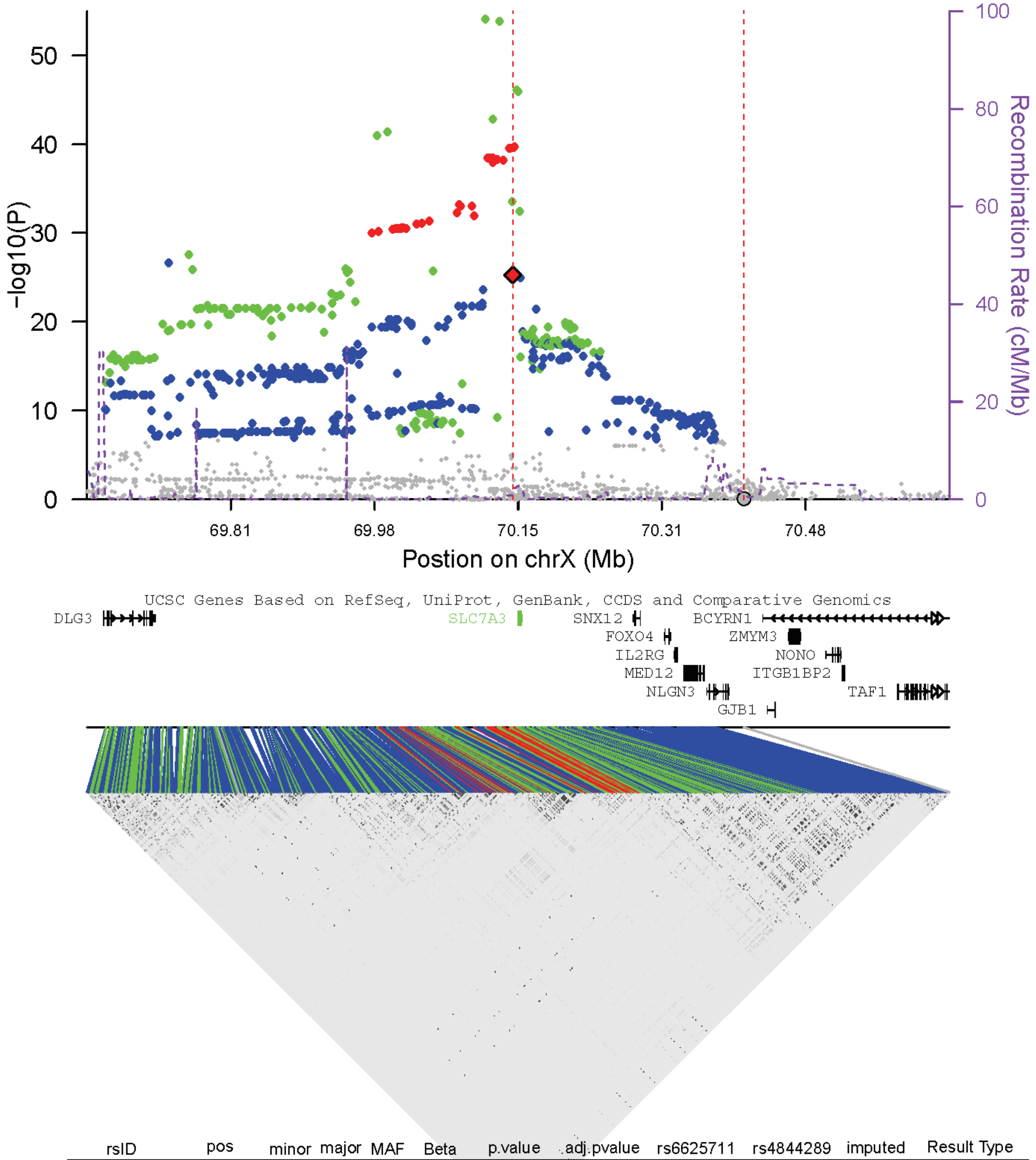

rsID	pos	minor	major	MAF	Beta	p.value	adj.pvalue	rs6625711	rs4844289	imputed	Result Type
rs62609681	69667815	C	T	0.25	0.256	7.35e−11	0.88819	<0.2	<0.2	1	Flanking
rs4617319	70108247	C	A	0.36	0.498	7.85e−55	−	0.367	<0.2	0	Peak
rs6525442	70117463	G	A	0.49	−0.427	1.17e−38	0.00232	0.722	<0.2	1	ReverseBeta
rs6625711	70139850	A	T	0.42	0.377	5.20e−26	0.00192	1.000	<0.2	1	PC Risk
rs5937025	70142030	C	T	0.48	0.424	1.88e−40	0.00224	0.759	<0.2	1	MaxLD
rs4844286	70371574	G	T	0.44	0.204	5.43e−09	0.98319	<0.2	<0.2	1	Flanking
rs7066438	70399819	T	A	0.31	0.071	5.65e−02	0.11992	<0.2	0.653	1	MaxLD
rs4844289	70407983	G	A	0.39	0.012	7.41e−01	0.25106	<0.2	1.000	1	PC Risk

Supplementary Figure 4 - Regional association plots for each of the genes placed in Group 3

Regional association plots display eQTL results for the target gene listed in the title and all SNPs in the target gene region. All PC risk-SNPs in the region are listed in the second line of the title. The x-axis shows the chromosomal position of the SNPs (with expressed genes in the region displayed below) and the y-axis is the –log10(p-value) obtained by regressing normalized expression levels for the target gene on the number of minor alleles of each SNP genotype adjusted for histologic characteristics and 14 expression principal components. The PC risk-SNP position is indicated by a dotted red vertical line with the eQTL result displayed as a diamond. Colored points show all Bonferroni significant results with the color defined by LD between the SNP and the PC risk-SNP (LD r^2>0.5 red, between 0.2 – 0.5 green, and ≤0.2 blue). If the region contains multiple PC risk-SNPs, the points are colored based on LD with the PC risk-SNP that is in highest LD with the eQTL peak associated SNP. The eQTL result for this PC risk-SNP is displayed as diamond, the data points for all of the other PC risk-SNPs are displayed as an open circle.

The table below the LD plot shows results for the peak-SNP (Result Type = Peak) and the most upstream and downstream SNPs (Result Type = Flanking) surpassing the Bonferroni significance threshold (P < 1.96E-07 for primary analysis and P < 3.02E-08 for second stage) as well as the top result for each PC risk SNP (Result Type = PC Risk) and, if a SNP in LD (r^2 > 0.5) with the PC risk SNP has a stronger association than the PC risk SNP (i.e., lower p-value), then the LD SNP with the strongest association is also presented (Result Type = MaxLD). Finally, if all of these SNPs have a single direction of effect, e.g., all have positive beta coefficients thus increasing gene expression, and if any Bonferroni significant results were

observed in the opposite direction, then the strongest result among the opposite direction SNPs is also presented (Result Type = ReverseBeta).

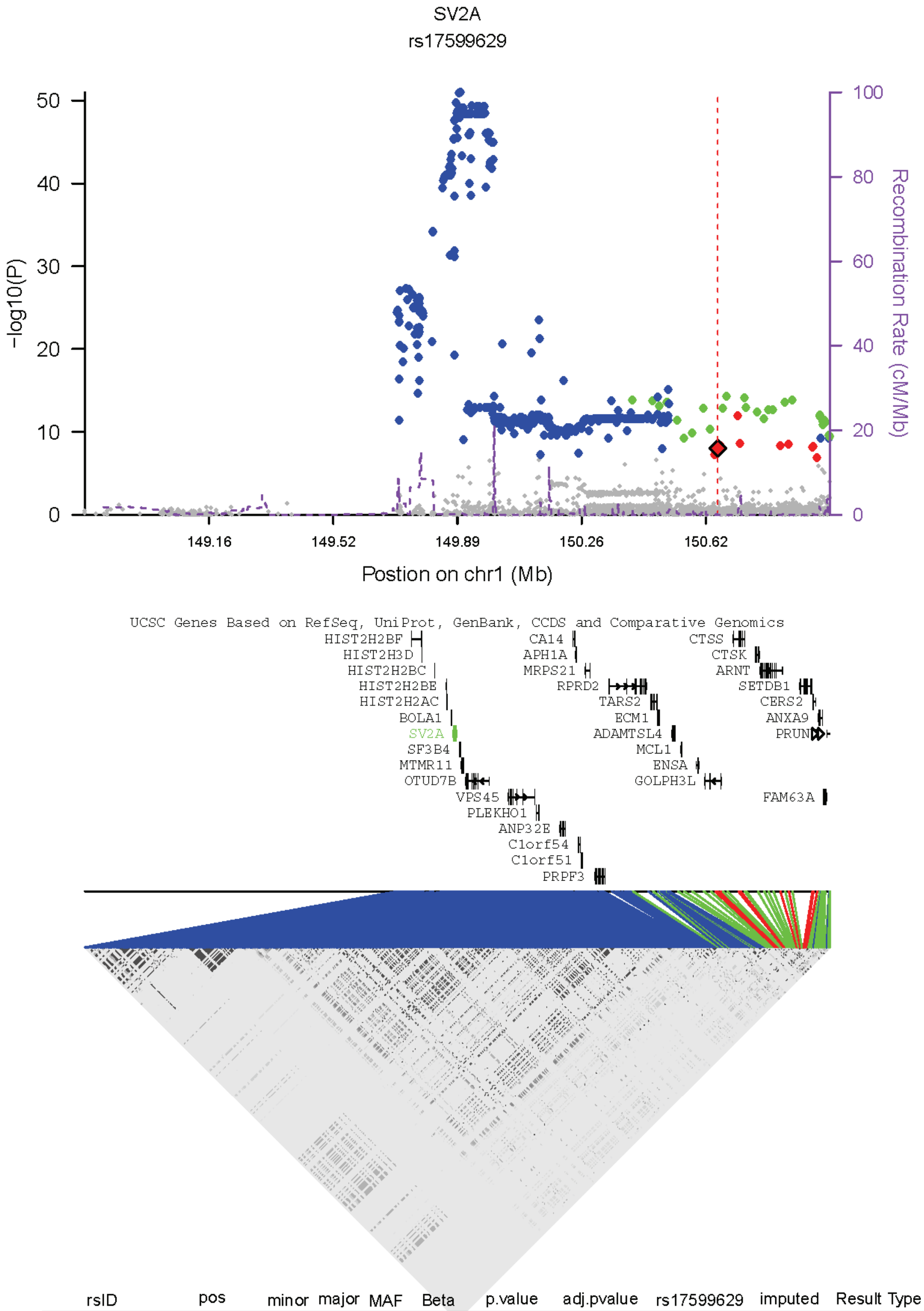

rsID	pos	minor	major	MAF	Beta	p.value	adj.pvalue	rs17599629	imputed	Result Type
rs147317935	149712677	A	G	0.09	−0.368	4.38e−25	0.16810	<0.2	1	Flanking
rs16836630	149898951	C	G	0.07	−0.446	1.09e−51	−	<0.2	1	Peak
rs17599629	150658287	G	A	0.25	−0.116	9.51e−09	0.04221	1.000	0	PC Risk
rs75056606	150718602	G	T	0.18	−0.156	1.10e−12	0.00215	0.636	0	MaxLD
rs4418603	150989274	G	A	0.09	−0.184	4.05e−10	0.27179	0.220	1	Flanking

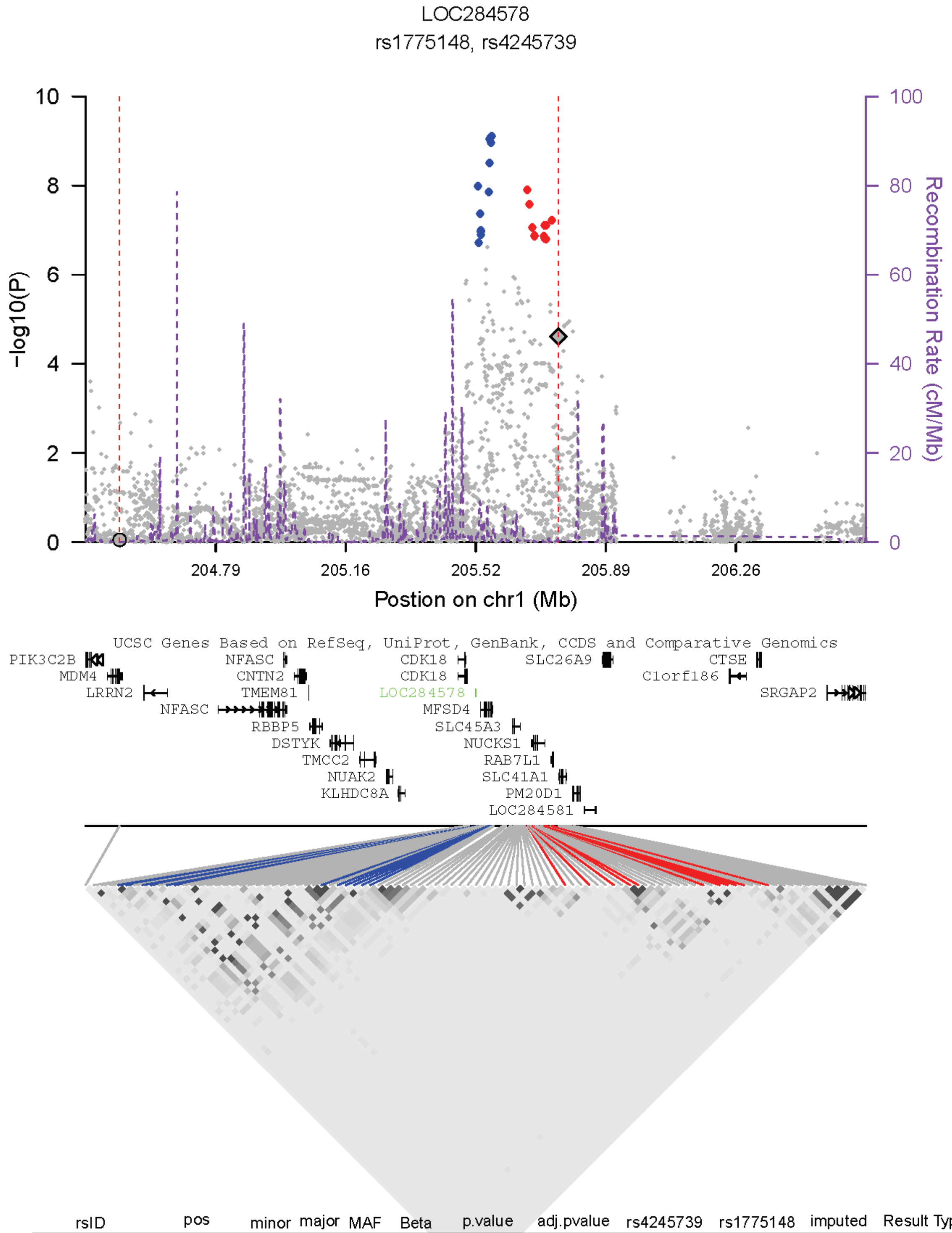

rsID	pos	minor	major	MAF	Beta	p.value	adj.pvalue	rs4245739	rs1775148	imputed	Result Type
rs4245739	204518842	C	A	0.26	0.008	8.74e−01	6.43e−01	1.000	<0.2	0	PC Risk
rs5010868	204550366	A	T	0.34	0.044	3.95e−01	2.78e−01	0.617	<0.2	1	MaxLD
rs149649212	205532538	G	T	0.12	0.423	1.03e−08	7.48e−03	<0.2	<0.2	1	Flanking
rs12041432	205570770	A	G	0.13	0.399	8.03e−10	–	<0.2	<0.2	1	Peak
rs6673687	205670369	T	A	0.43	−0.251	1.25e−08	1.26e−06	<0.2	0.545	1	MaxLD
rs708723	205739266	C	T	0.49	−0.234	6.12e−08	6.12e−07	<0.2	0.678	0	Flanking
rs1775148	205757824	C	T	0.43	−0.189	2.45e−05	5.15e−05	<0.2	1.000	1	PC Risk

ITGA6

rs12621278

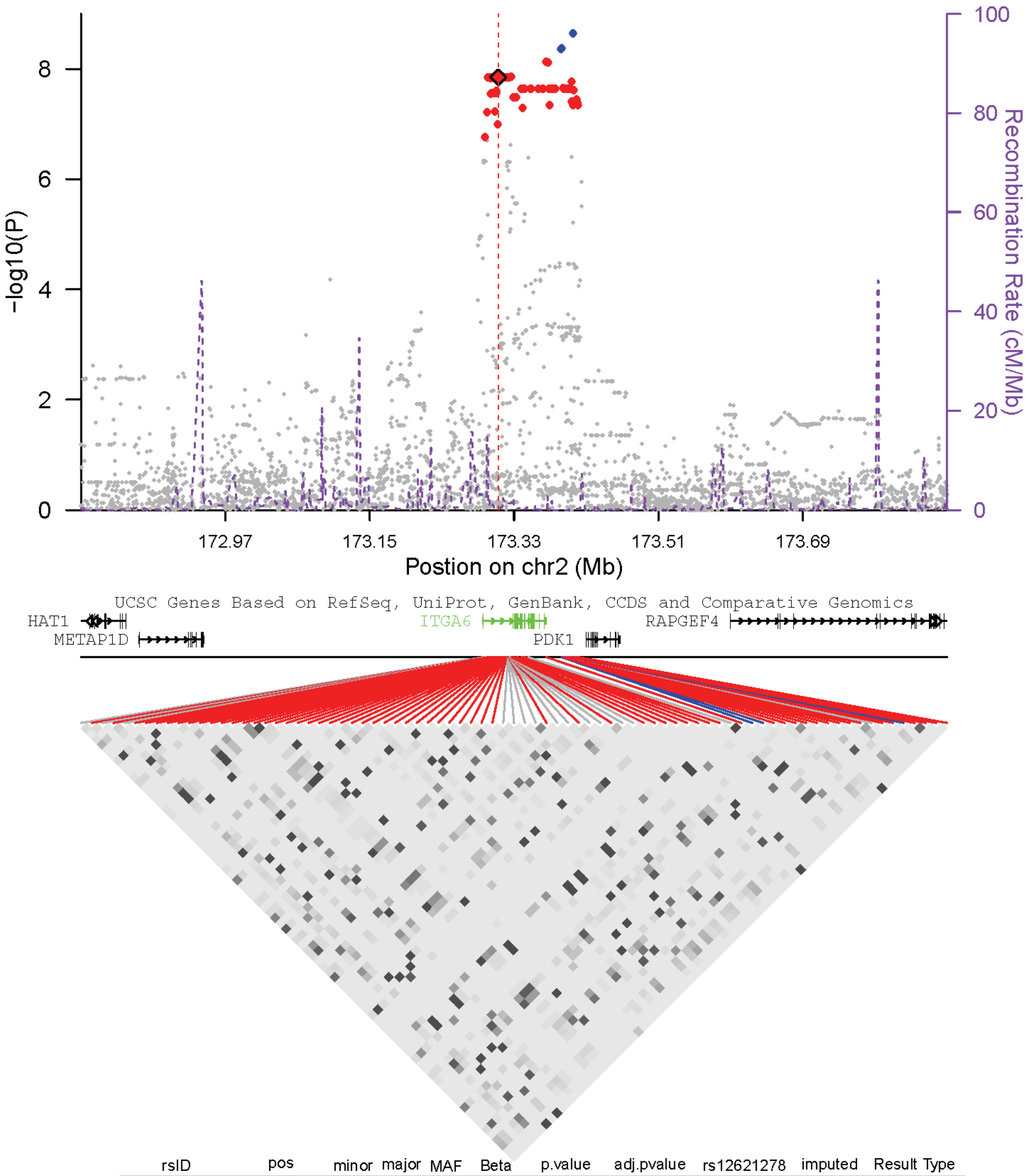

rsID	pos	minor	major	MAF	Beta	p.value	adj.pvalue	rs12621278	imputed	Result Type
rs12053442	173295405	C	G	0.05	−0.128	1.75e−07	0.000568	0.868	1	Flanking
rs12621278	173311553	G	A	0.05	−0.136	1.42e−08	0.000167	1.000	1	PC Risk
rs16860643	173371278	G	A	0.05	−0.138	7.39e−09	0.000122	0.885	0	MaxLD
rs7591769	173404886	A	G	0.22	−0.072	2.26e−09	−	<0.2	1	Peak
rs115160117	173411004	C	G	0.05	−0.138	4.52e−08	0.000517	0.901	1	Flanking

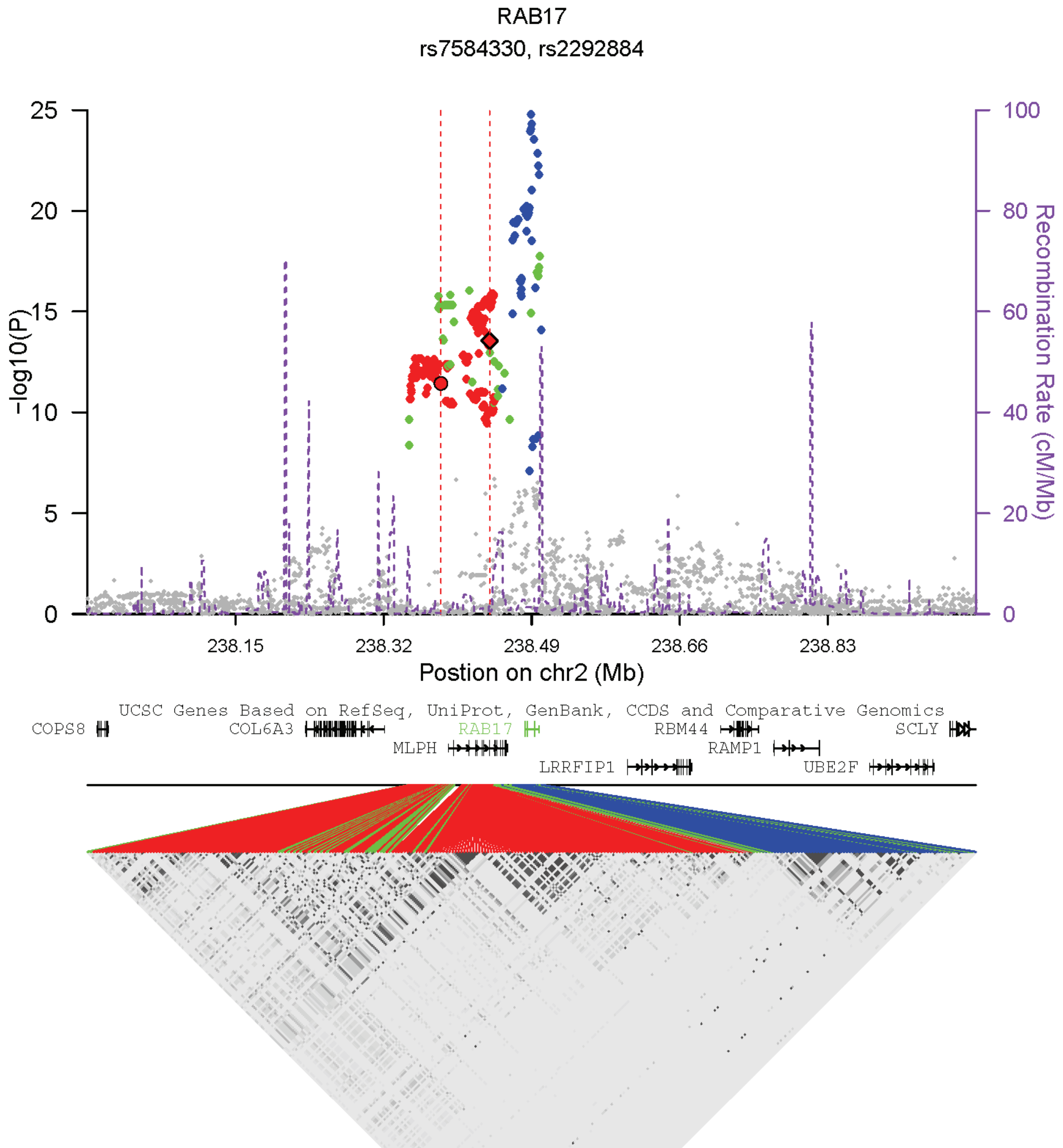

rsID	pos	minor	major	MAF	Beta	p.value	adj.pvalue	rs7584330	rs2292884	imputed	Result Type
rs12471077	238351337	T	C	0.14	−0.213	4.35e−09	2.04e−02	0.345	0.288	0	Flanking
rs7584330	238387228	G	A	0.28	−0.192	3.54e−12	3.77e−04	1.000	0.690	0	PC Risk
rs79618460	238419959	C	T	0.16	−0.276	9.21e−17	2.21e−04	0.515	0.481	1	MaxLD
rs2292884	238443226	G	A	0.27	−0.204	2.84e−14	7.70e−05	0.690	1.000	0	PC Risk
rs74003103	238446699	A	G	0.17	−0.268	1.32e−16	7.87e−04	0.503	0.551	1	MaxLD
rs74003104	238446823	T	C	0.17	−0.268	1.32e−16	7.87e−04	0.503	0.551	1	MaxLD
rs74003105	238446851	C	T	0.17	−0.268	1.32e−16	7.87e−04	0.503	0.551	0	MaxLD
rs74003106	238447004	G	A	0.17	−0.268	1.32e−16	7.87e−04	0.503	0.551	0	MaxLD
rs13404216	238491258	A	C	0.36	−0.271	1.67e−25	−	<0.2	<0.2	1	Peak
rs113771323	238491714	T	C	0.09	0.428	5.22e−25	2.60e−21	<0.2	<0.2	1	ReverseBeta
rs10929236	238502591	G	A	0.32	−0.208	8.50e−15	1.96e−06	<0.2	<0.2	0	Flanking

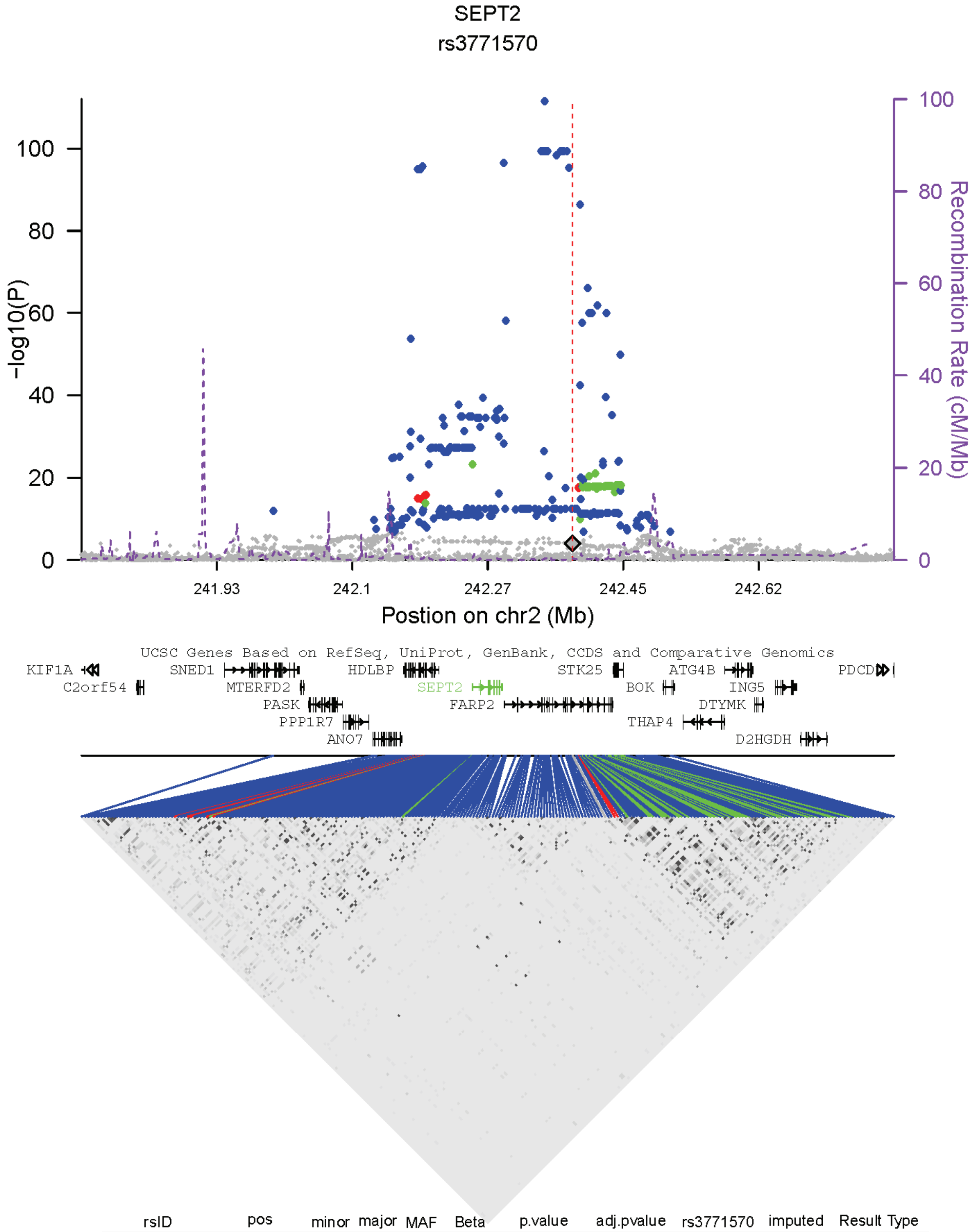

rsID	pos	minor	major	MAF	Beta	p.value	adj.pvalue	rs3771570	imputed	Result Type
rs79100040	242000648	A	G	0.09	0.102	1.42e−12	4.04e−01	<0.2	0	Flanking
rs115271170	242347614	T	C	0.10	0.248	3.33e−112	–	<0.2	1	Peak
rs3771570	242382864	T	C	0.16	−0.044	9.12e−05	5.73e−02	1.000	1	PC Risk
rs3771578	242391212	T	C	0.26	0.080	2.18e−18	5.65e−02	0.507	1	MaxLD
rs10201714	242488000	A	G	0.45	−0.050	7.18e−09	9.67e−06	<0.2	1	Flanking

WDR52
rs7611694

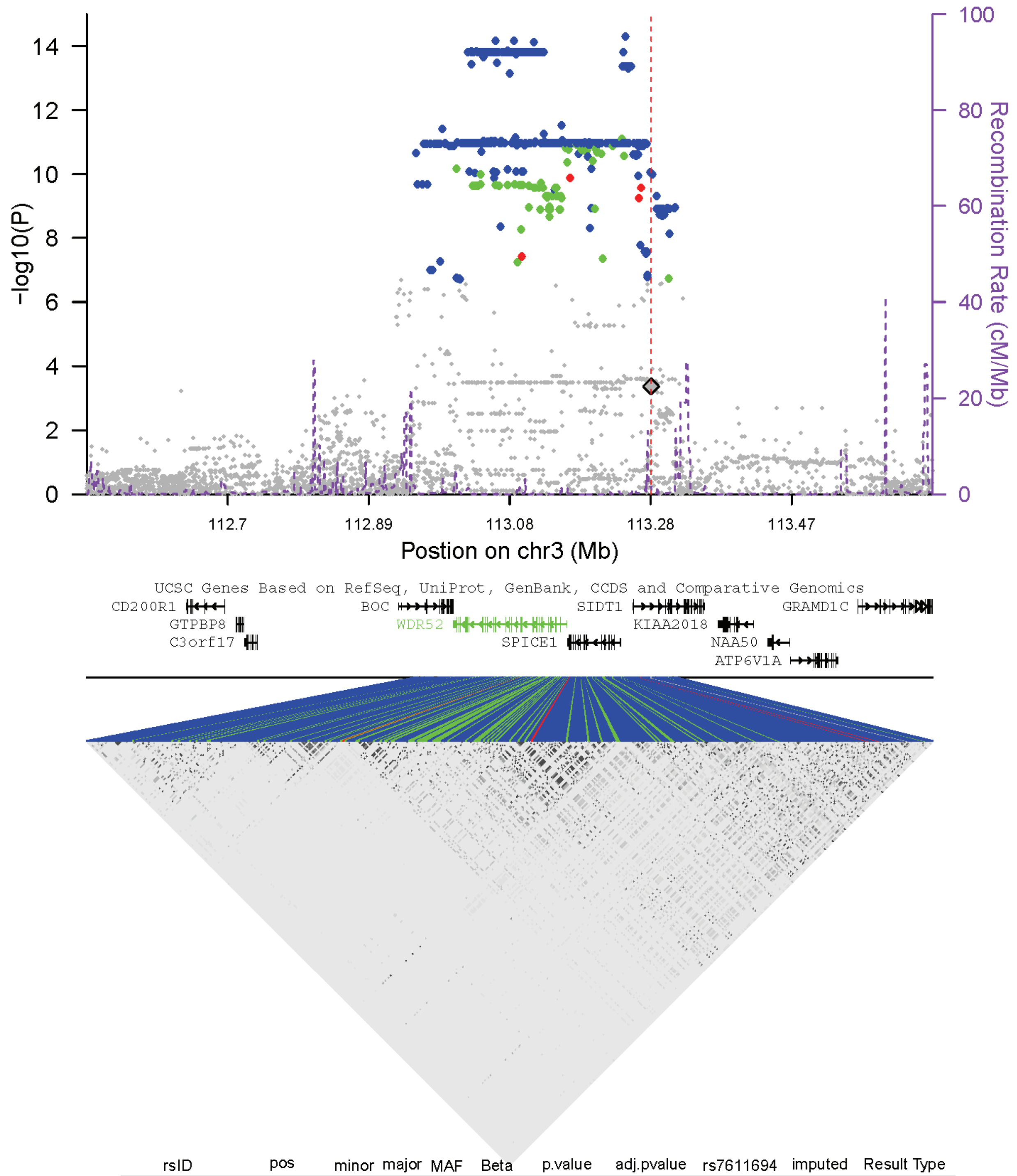

rsID	pos	minor	major	MAF	Beta	p.value	adj.pvalue	rs7611694	imputed	Result Type
rs116425520	112955254	T	C	0.03	−0.226	2.25e−11	2.00e−02	<0.2	1	Flanking
rs2129430	113165773	C	G	0.43	−0.074	1.30e−10	6.08e−06	0.508	1	MaxLD
rs2007579	113241623	A	G	0.05	−0.198	5.09e−15	−	<0.2	1	Peak
rs7611694	113275624	C	A	0.40	−0.041	4.28e−04	2.29e−03	1.000	0	PC Risk
rs138921562	113308528	A	G	0.03	−0.206	1.16e−09	1.85e−01	<0.2	1	Flanking

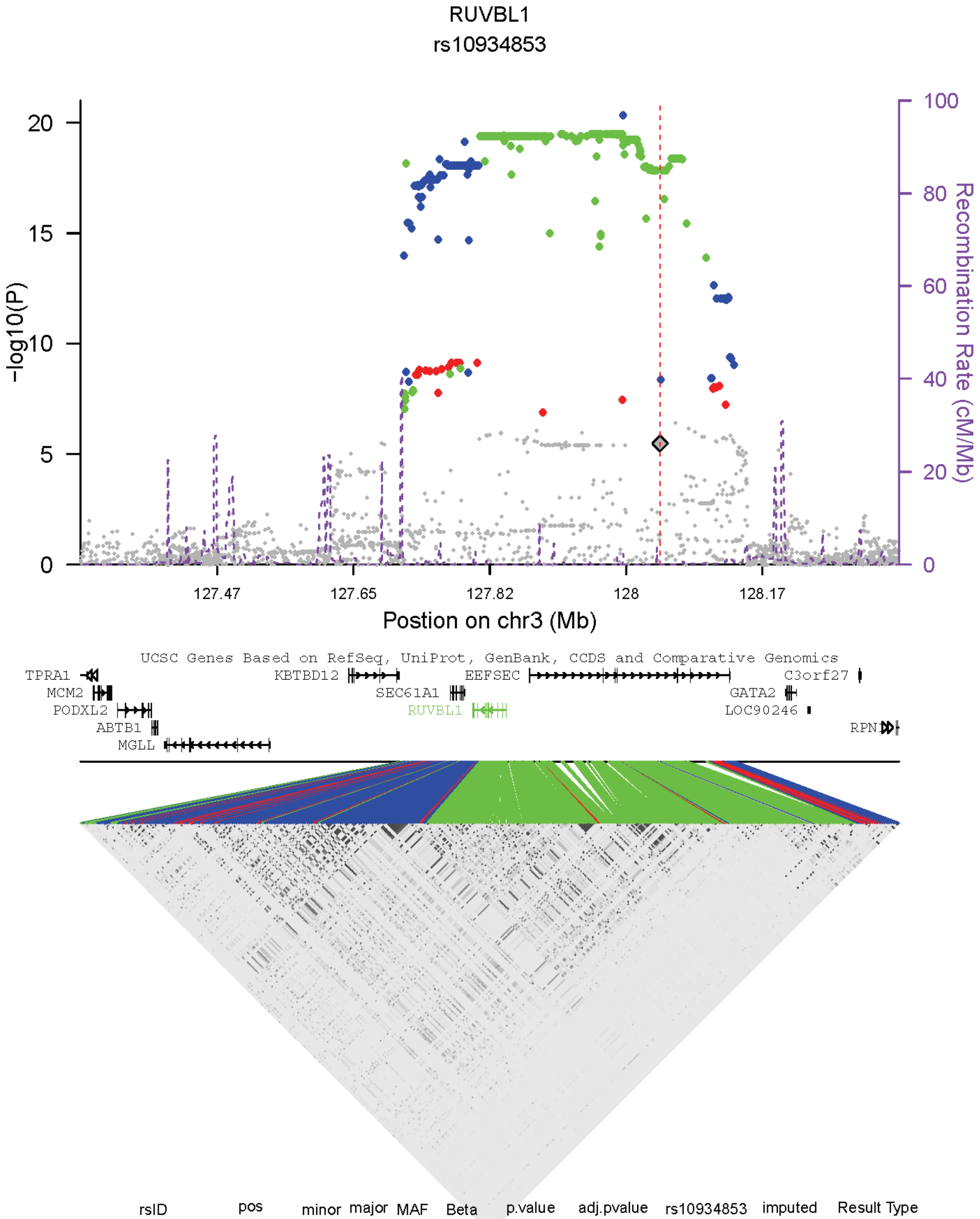

rsID	pos	minor	major	MAF	Beta	p.value	adj.pvalue	rs10934853	imputed	Result Type
rs9839080	127711997	T	C	0.44	0.047	2.03e−08	0.3595	0.474	1	Flanking
rs74501258	127771159	A	G	0.45	−0.055	2.48e−09	0.0687	0.350	1	ReverseBeta
rs2075402	127784262	C	T	0.43	0.051	7.69e−10	0.2208	0.530	1	MaxLD
rs2811415	127991527	A	G	0.17	0.099	4.60e−21	−	<0.2	0	Peak
rs10934853	128038373	A	C	0.30	0.042	3.32e−06	0.7554	1.000	0	PC Risk
rs56299192	128133006	T	A	0.09	0.092	9.82e−10	0.1569	<0.2	1	Flanking

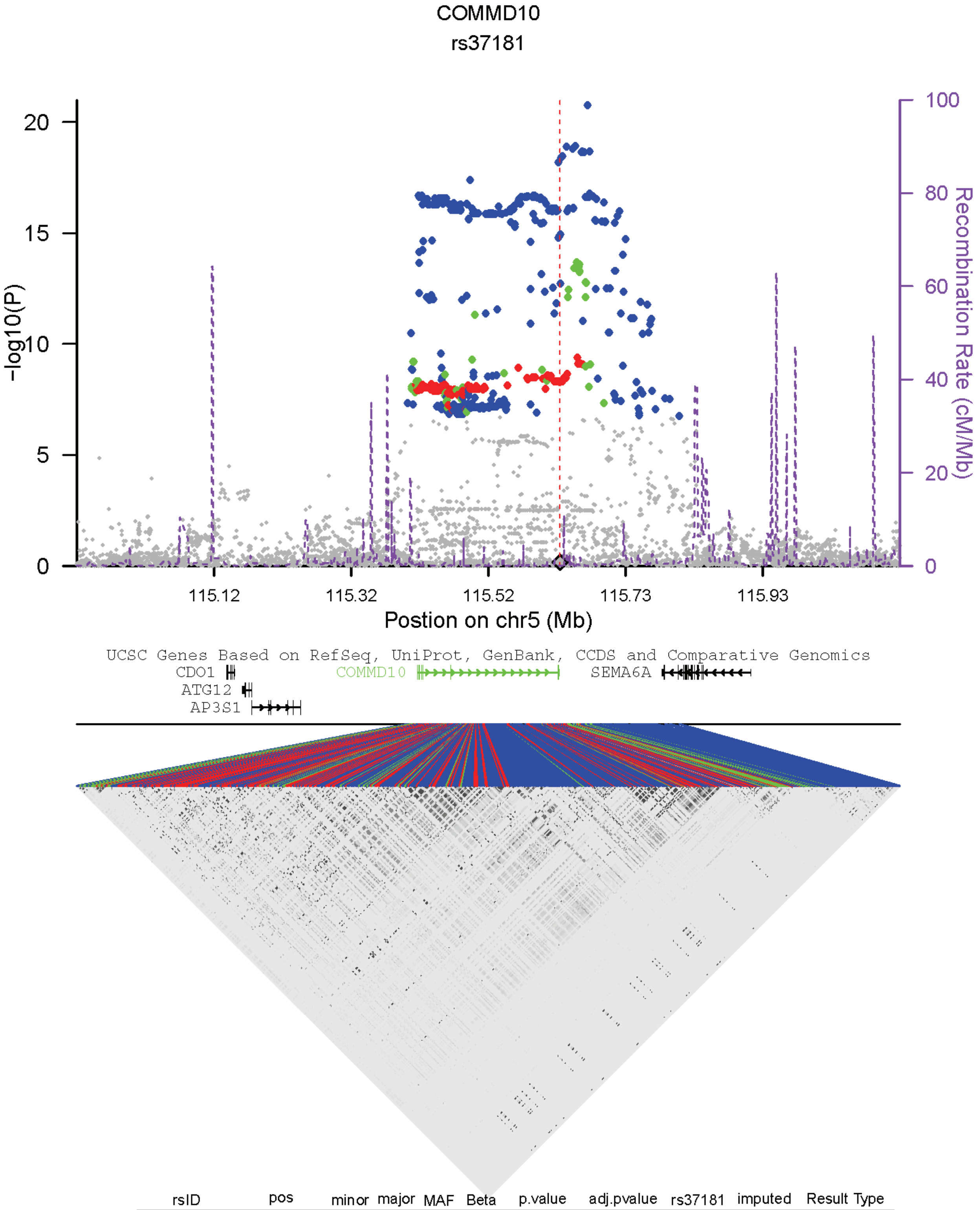

rsID	pos	minor	major	MAF	Beta	p.value	adj.pvalue	rs37181	imputed	Result Type
rs72804805	115411582	C	G	0.15	0.069	3.30e−11	0.00248	<0.2	0	Flanking
rs37181	115630004	G	T	0.22	0.004	6.79e−01	0.00743	1.000	1	PC Risk
rs785373	115655585	C	G	0.36	0.048	4.31e−10	0.33446	0.507	1	MaxLD
rs804152	115670932	C	T	0.23	0.080	1.82e−21	–	<0.2	0	Peak
rs56153002	115784061	C	G	0.09	0.066	2.83e−08	0.05587	<0.2	0	Flanking

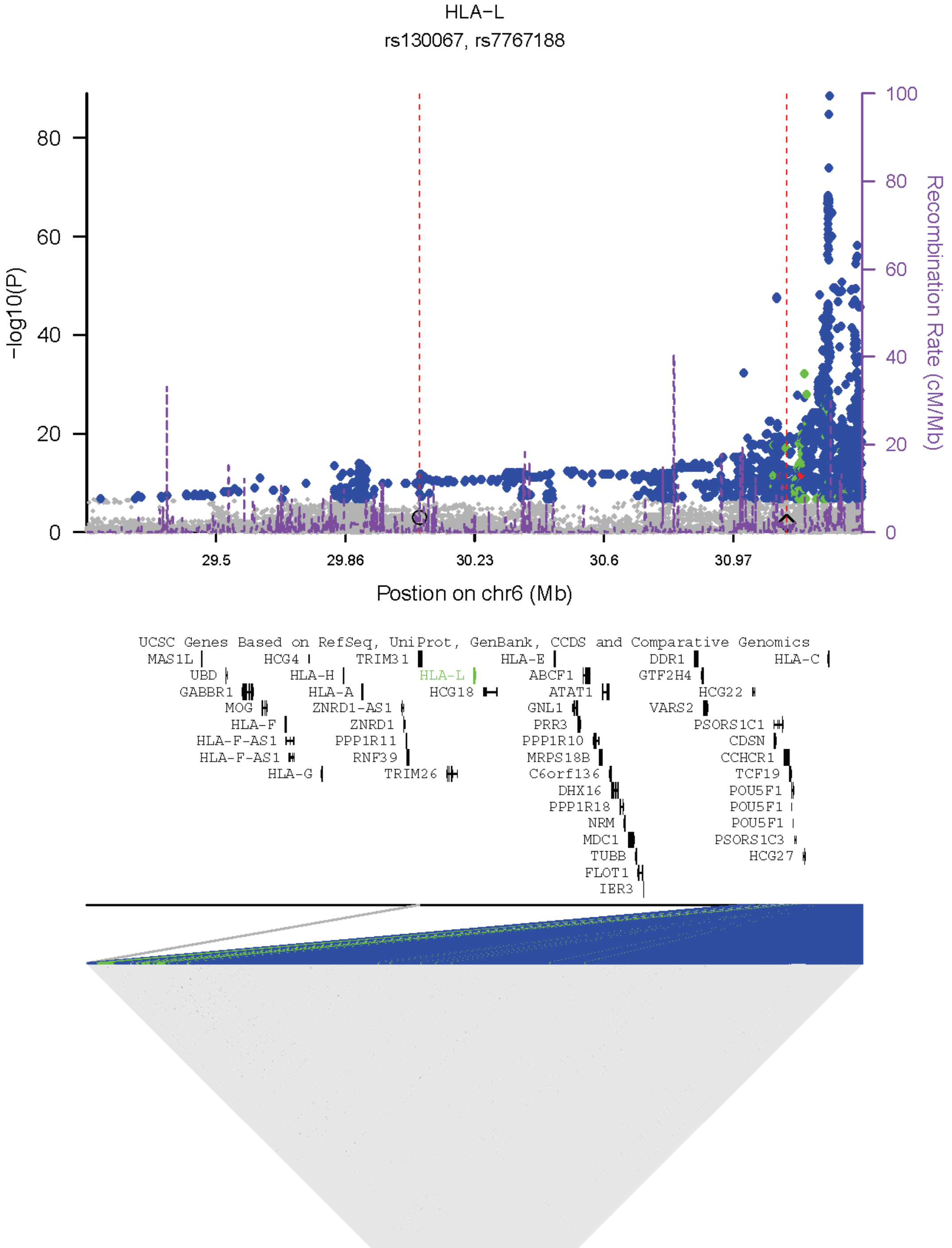

rsID	pos	minor	major	MAF	Beta	p.value	adj.pvalue	rs7767188	rs130067	imputed	Result Type
rs147080558	29413003	T	C	0.09	−0.416	1.75e−08	0.06307	<0.2	<0.2	1	Flanking
rs3823374	29944148	A	C	0.36	0.219	5.93e−07	0.03724	0.518	<0.2	0	MaxLD
rs3823375	29944158	C	T	0.36	0.219	5.93e−07	0.03724	0.518	<0.2	0	MaxLD
rs7767188	30073776	A	G	0.24	0.165	8.24e−04	0.04196	1.000	<0.2	0	PC Risk
rs130067	31118511	G	T	0.21	0.126	1.51e−02	0.00719	<0.2	1.000	0	PC Risk
rs139078838	31164511	T	C	0.31	0.322	2.39e−12	0.41570	<0.2	0.557	1	MaxLD
rs115212502	31242151	C	T	0.43	−0.691	4.01e−89	−	<0.2	<0.2	1	Peak
rs114949789	31334601	T	C	0.18	−0.354	5.64e−11	0.21759	<0.2	<0.2	1	Flanking

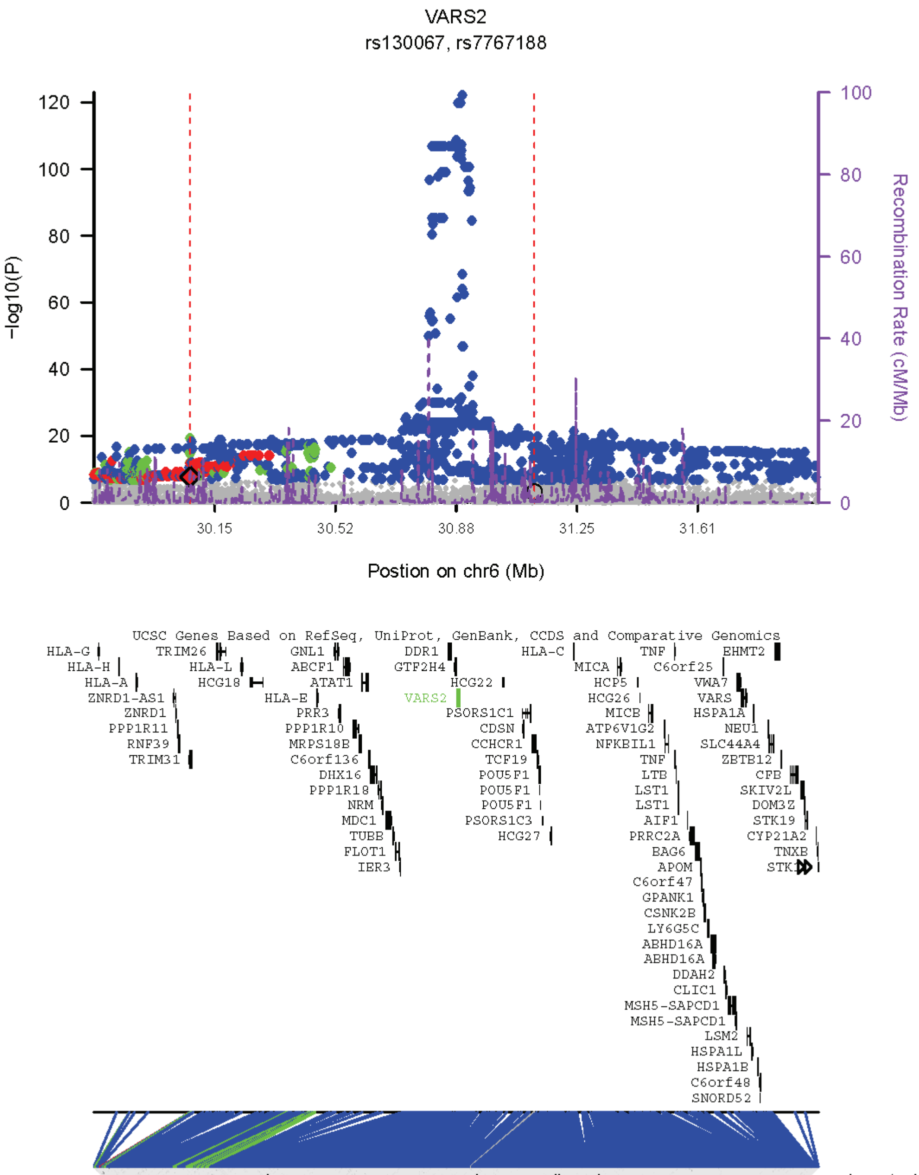

rsID	pos	minor	major	MAF	Beta	p.value	adj.pvalue	rs7767188	rs130067	imputed	Result Type
rs116078832	29784503	G	A	0.35	0.096	4.87e−09	0.122360	0.517	<0.2	1	Flanking
rs7767188	30073776	A	G	0.24	0.104	1.67e−08	0.000301	1.000	<0.2	0	PC Risk
rs9261639	30237859	A	G	0.30	0.125	1.04e−14	0.000010	0.624	<0.2	0	MaxLD
rs115591439	30252836	T	A	0.30	0.125	1.04e−14	0.000010	0.624	<0.2	1	MaxLD
rs147212564	30254678	A	G	0.30	0.125	1.04e−14	0.000010	0.624	<0.2	1	MaxLD
rs115368375	30257497	G	C	0.30	0.125	1.04e−14	0.000010	0.624	<0.2	1	MaxLD
rs261946	30271334	G	A	0.30	0.125	1.04e−14	0.000010	0.624	<0.2	0	MaxLD
rs115267644	30272459	A	G	0.30	0.125	1.04e−14	0.000010	0.624	<0.2	1	MaxLD
rs116555278	30285650	A	T	0.30	0.125	1.04e−14	0.000010	0.624	<0.2	1	MaxLD
rs2844750	30313268	A	G	0.30	0.125	1.04e−14	0.000010	0.624	<0.2	0	MaxLD
rs2844749	30314116	A	G	0.30	0.125	1.04e−14	0.000010	0.624	<0.2	0	MaxLD
rs114078037	30315011	A	T	0.30	0.125	1.04e−14	0.000010	0.624	<0.2	1	MaxLD
rs2844651	30900664	T	C	0.46	−0.288	8.36e−123	−	<0.2	<0.2	0	Peak
rs1265093	31107187	A	G	0.29	0.103	3.42e−09	0.015398	<0.2	0.639	0	MaxLD
rs130067	31118511	G	T	0.21	0.068	4.55e−04	0.779739	<0.2	1.000	0	PC Risk
rs149411182	31973863	T	C	0.47	0.090	2.06e−08	0.210808	<0.2	<0.2	1	Flanking

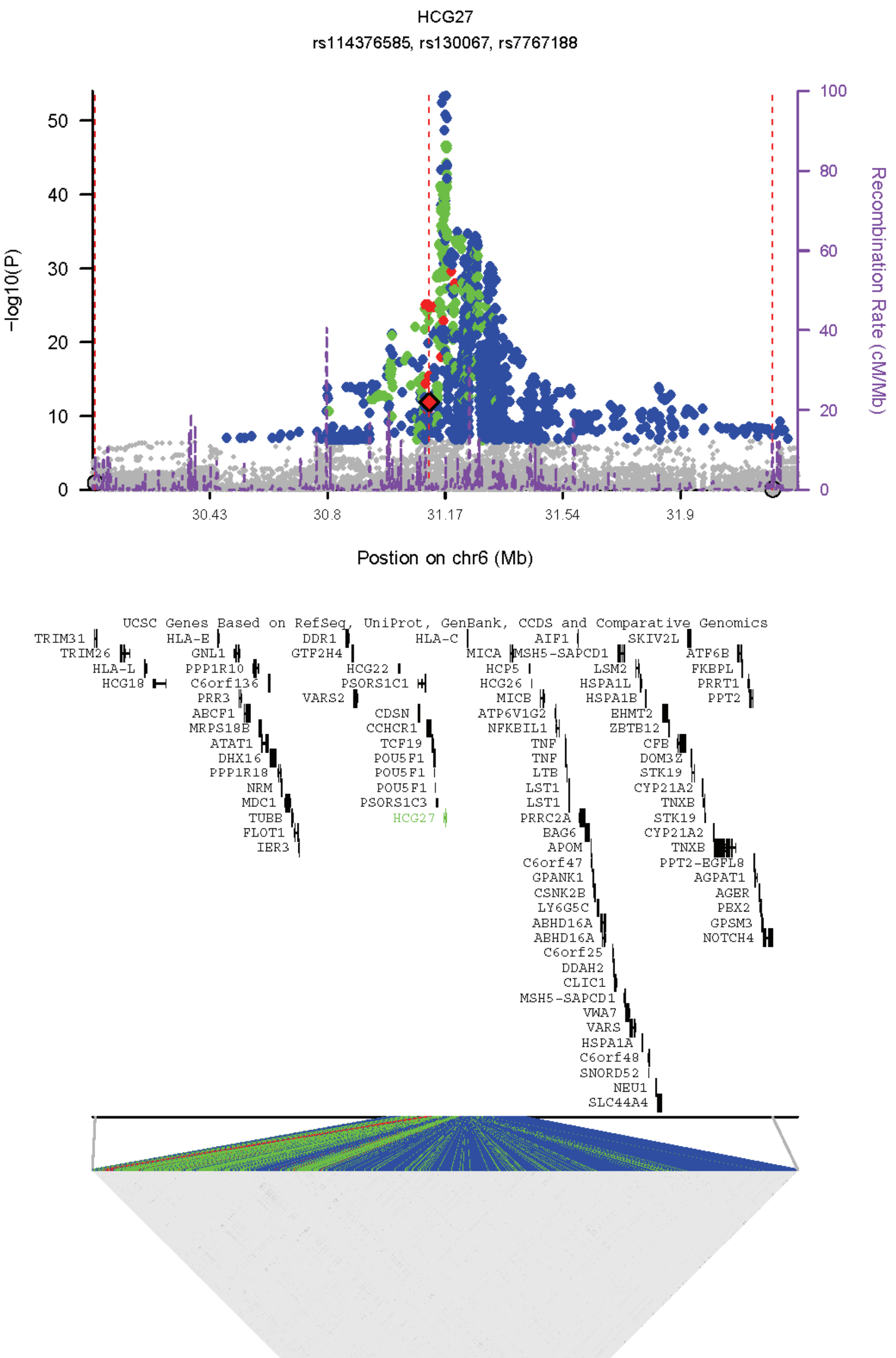

rsID	pos	minor	major	MAF	Beta	p.value	adj.pvalue	rs7767188	rs130067	rs114376585	imputed	Result Type
rs7767188	30073776	A	G	0.24	0.063	9.87e−02	3.51e−02	1.000	<0.2	<0.2	0	PC Risk
rs183830067	30217185	T	A	0.31	0.149	3.19e−05	4.54e−04	0.514	<0.2	<0.2	1	MaxLD
rs114637387	30711805	C	G	0.10	−0.302	1.60e−08	2.11e−03	<0.2	<0.2	<0.2	1	Flanking
rs130067	31118511	G	T	0.21	0.278	1.36e−12	2.32e−04	<0.2	1.000	<0.2	0	PC Risk
rs6930065	31173669	C	T	0.34	0.457	4.41e−54	–	<0.2	<0.2	<0.2	0	Peak
rs145076668	31188436	T	C	0.13	0.526	2.74e−30	9.42e−08	<0.2	0.565	<0.2	1	MaxLD
rs114376585	32192331	A	G	0.38	−0.012	7.05e−01	6.50e−01	<0.2	<0.2	1.000	0	PC Risk
rs116522847	32197219	G	T	0.45	0.030	3.43e−01	3.06e−01	<0.2	<0.2	0.516	1	MaxLD
rs140250697	32230354	A	G	0.37	−0.187	9.01e−09	1.87e−02	<0.2	<0.2	<0.2	1	Flanking

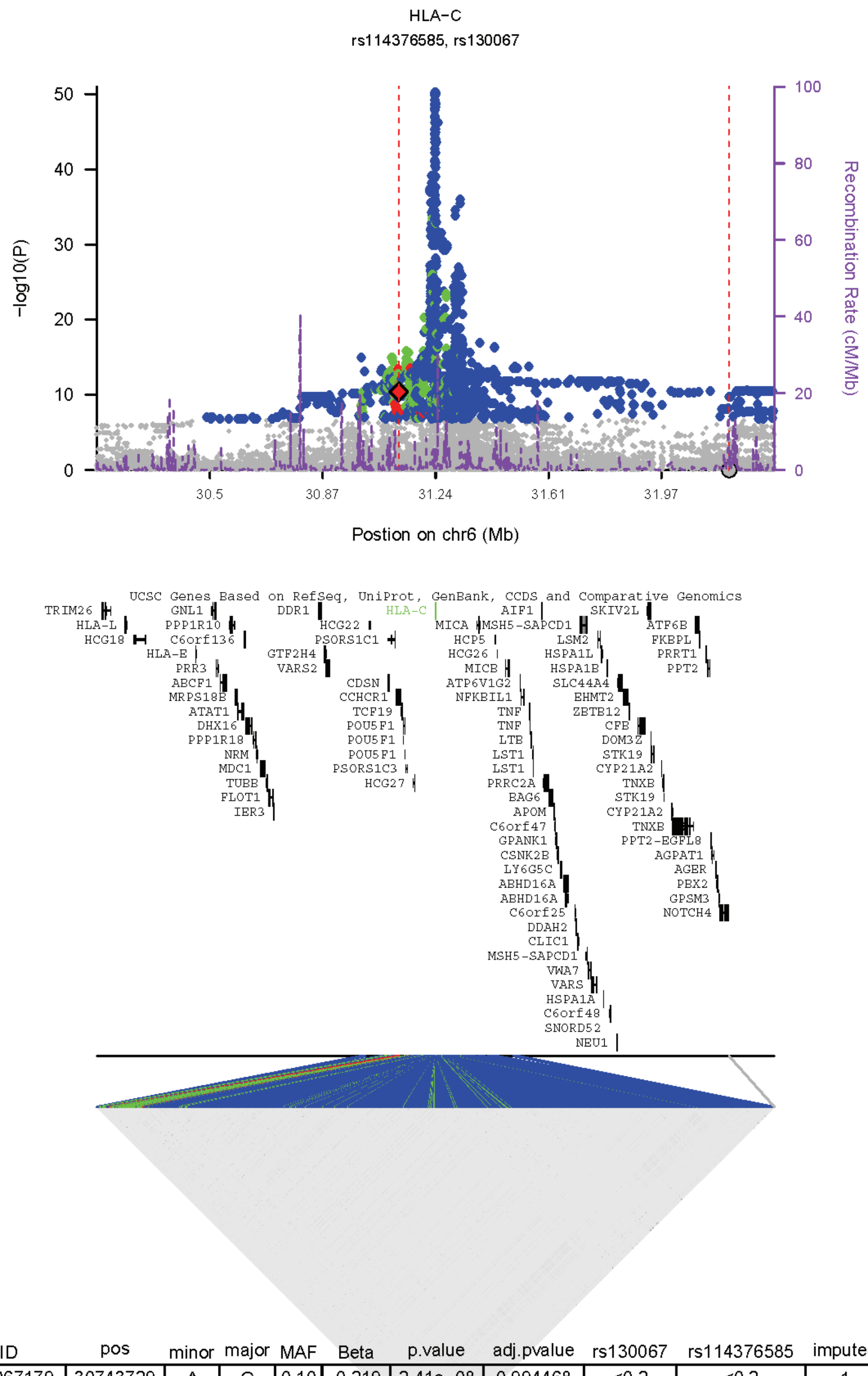

rsID	pos	minor	major	MAF	Beta	p.value	adj.pvalue	rs130067	rs114376585	imputed	Result Type
rs149067179	30743729	A	G	0.10	0.219	2.41e−08	0.994468	<0.2	<0.2	1	Flanking
rs130067	31118511	G	T	0.21	−0.196	4.29e−11	0.000263	1.000	<0.2	0	PC Risk
rs144721865	31156041	T	C	0.28	−0.204	3.09e−14	0.000590	0.637	<0.2	1	MaxLD
rs78848968	31240010	G	A	0.32	0.343	5.67e−51	−	<0.2	<0.2	0	Peak
rs114376585	32192331	A	G	0.38	−0.003	9.06e−01	0.089796	<0.2	1.000	0	PC Risk
rs116522847	32197219	G	T	0.45	−0.091	1.44e−04	0.931817	<0.2	0.516	1	MaxLD
rs3129939	32336766	G	A	0.17	0.187	1.67e−08	0.054610	<0.2	<0.2	0	Flanking

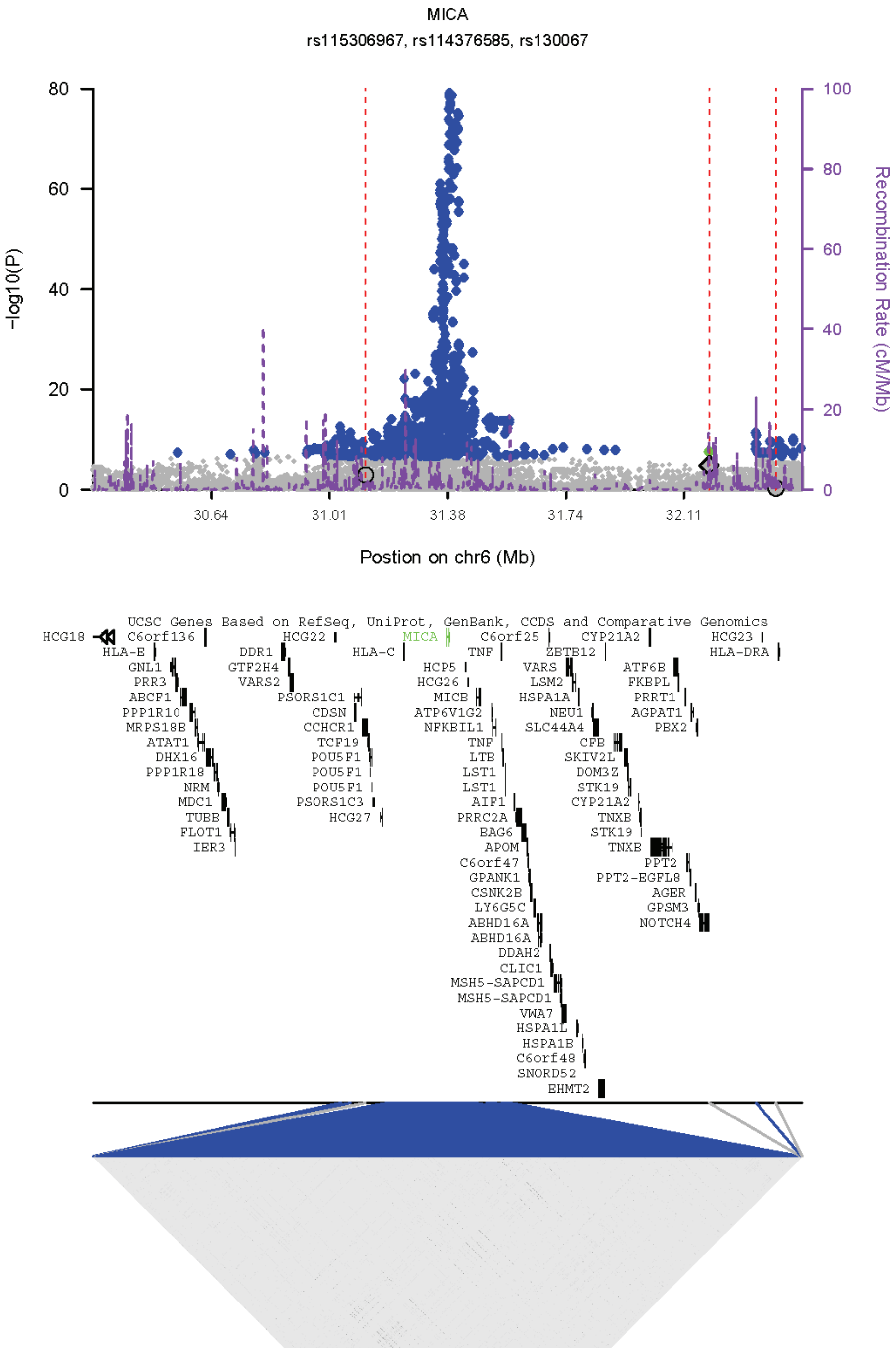

rsID	pos	minor	major	MAF	Beta	p.value	adj.pvalue	rs130067	rs114376585	rs115306967	imputed	Result Type
rs1264372	30769726	T	C	0.25	0.138	1.44e−08	0.000455	<0.2	<0.2	<0.2	0	Flanking
rs130067	31118511	G	T	0.21	0.085	1.02e−03	0.088742	1.000	<0.2	<0.2	0	PC Risk
rs115683835	31221299	A	G	0.13	0.180	7.25e−09	0.002950	0.553	<0.2	<0.2	1	MaxLD
rs148355290	31383304	A	G	0.27	−0.374	9.13e−80	–	<0.2	<0.2	<0.2	1	Peak
rs114376585	32192331	A	G	0.38	0.090	1.48e−05	0.119371	<0.2	1.000	<0.2	0	PC Risk
rs116522847	32197219	G	T	0.45	−0.103	3.39e−07	0.002029	<0.2	0.516	<0.2	1	MaxLD
rs743862	32381939	C	T	0.33	−0.060	4.15e−03	0.769695	<0.2	<0.2	0.532	0	MaxLD
rs115306967	32400939	C	G	0.28	−0.019	4.12e−01	0.220896	<0.2	<0.2	1.000	1	PC Risk
rs112941517	32480174	C	A	0.35	−0.151	6.69e−09	0.034710	<0.2	<0.2	<0.2	1	Flanking

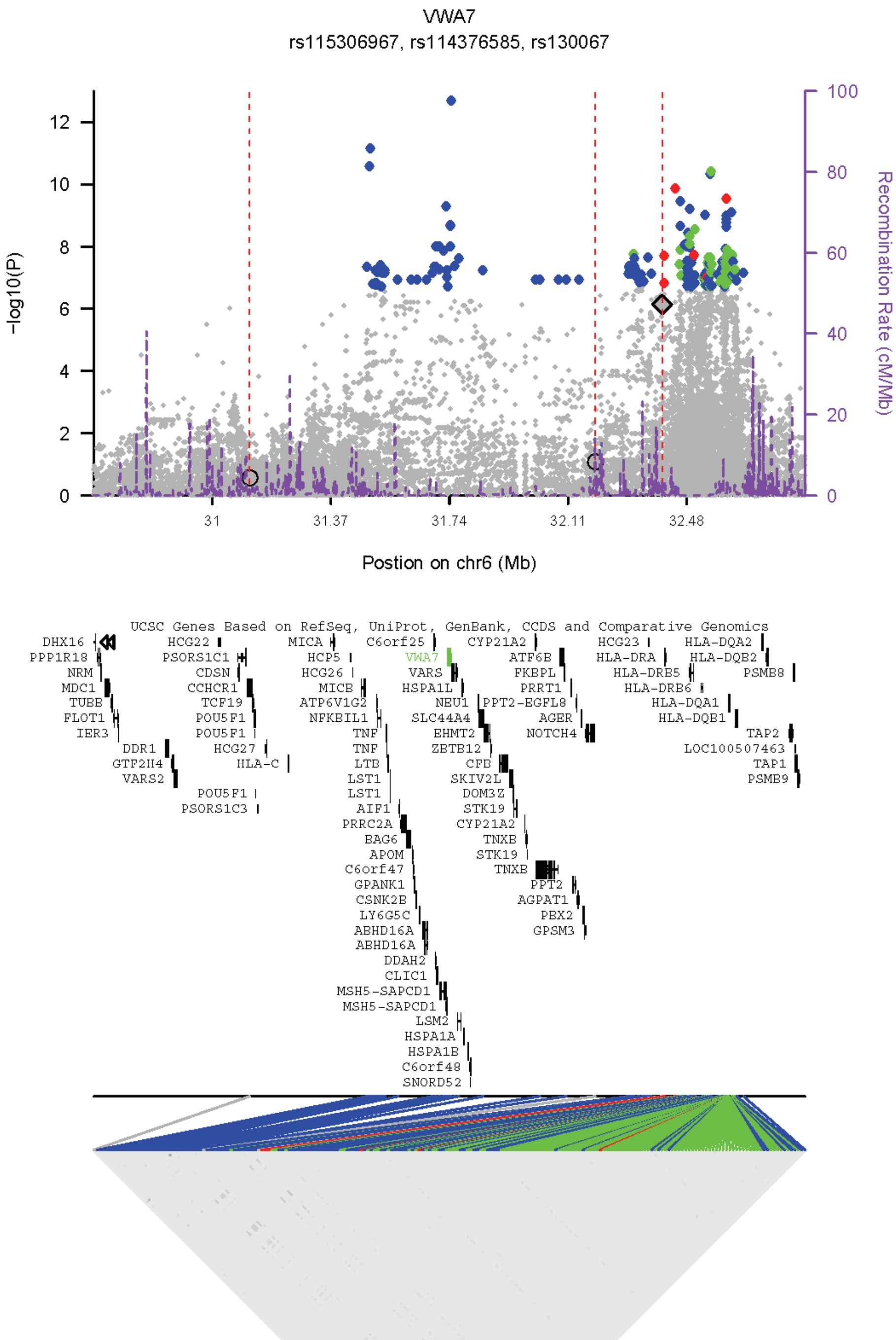

rsID	pos	minor	major	MAF	Beta	p.value	adj.pvalue	rs130067	rs114376585	rs115306967	imputed	Result Type
rs130067	31118511	G	T	0.21	0.035	2.61e−01	2.30e−01	1.000	<0.2	<0.2	0	PC Risk
rs145076668	31188436	T	C	0.13	0.102	7.70e−03	3.19e−02	0.565	<0.2	<0.2	1	MaxLD
rs141114485	31492157	G	C	0.35	−0.170	2.70e−11	2.73e−05	<0.2	<0.2	<0.2	1	Flanking
rs2736426	31745284	C	T	0.48	0.182	2.02e−13	–	<0.2	<0.2	<0.2	0	Peak
rs114376585	32192331	A	G	0.38	0.044	8.00e−02	7.67e−01	<0.2	1.000	<0.2	0	PC Risk
rs141507970	32206103	C	G	0.37	0.059	2.12e−02	3.59e−01	<0.2	0.627	<0.2	1	MaxLD
rs115306967	32400939	C	G	0.28	−0.139	7.30e−07	3.07e−04	<0.2	<0.2	1.000	1	PC Risk
rs143466021	32443223	A	G	0.35	−0.176	1.35e−10	2.45e−06	<0.2	<0.2	0.578	1	MaxLD
rs28374650	32623367	T	C	0.25	−0.163	2.99e−08	8.99e−06	<0.2	<0.2	<0.2	1	Flanking

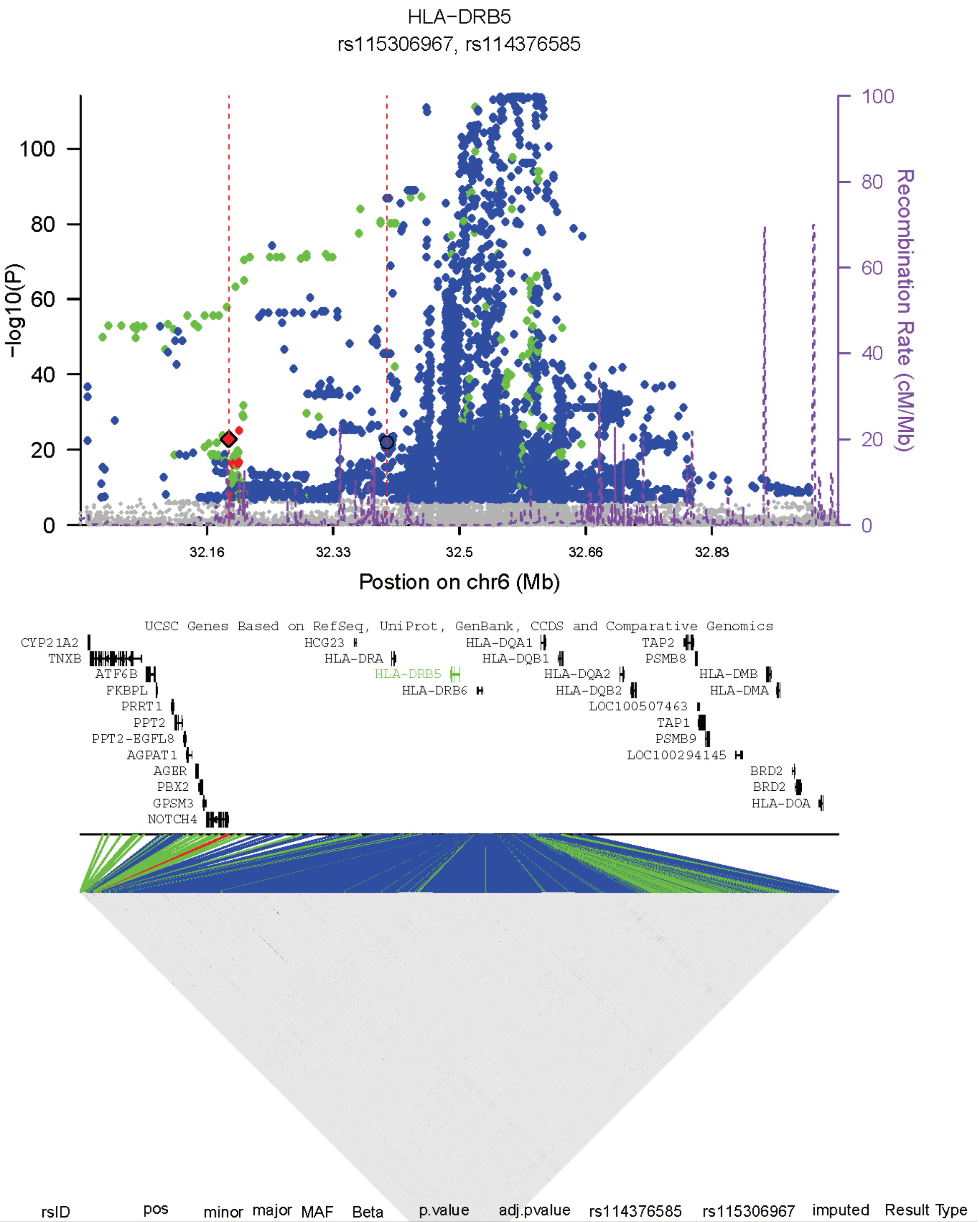

rsID	pos	minor	major	MAF	Beta	p.value	adj.pvalue	rs114376585	rs115306967	imputed	Result Type
rs2516450	31387157	C	T	0.15	0.523	1.80e−21	8.88e−01	<0.2	<0.2	0	Flanking
rs114376585	32192331	A	G	0.38	0.399	1.54e−23	5.33e−08	1.000	<0.2	0	PC Risk
rs141507970	32206103	C	G	0.37	0.419	1.11e−25	1.15e−06	0.627	<0.2	1	MaxLD
rs115306967	32400939	C	G	0.28	−0.440	8.52e−23	3.41e−18	<0.2	1.000	1	PC Risk
rs143466021	32443223	A	G	0.35	−0.556	1.56e−39	9.91e−31	<0.2	0.578	1	MaxLD
rs9271085	32576152	T	C	0.17	0.988	1.67e−114	−	<0.2	<0.2	1	Peak
rs114014593	33045966	A	G	0.40	−0.234	1.33e−08	1.89e−03	<0.2	<0.2	1	Flanking

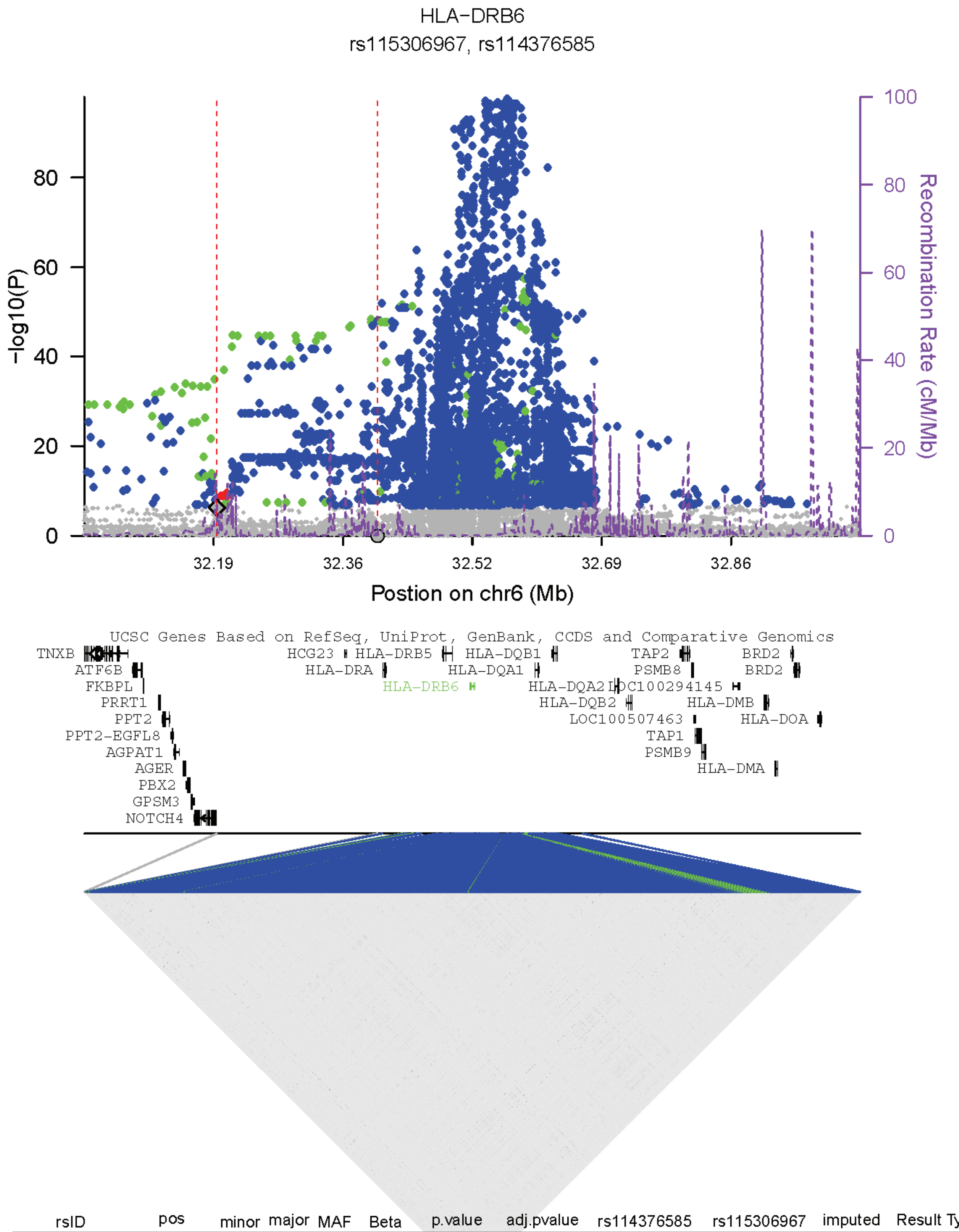

rsID	pos	minor	major	MAF	Beta	p.value	adj.pvalue	rs114376585	rs115306967	imputed	Result Type
rs2596471	31428911	A	G	0.18	−0.767	2.06e−10	0.1400	<0.2	<0.2	0	Flanking
rs114376585	32192331	A	G	0.38	−0.466	5.69e−07	0.0409	1.000	<0.2	0	PC Risk
rs141507970	32206103	C	G	0.37	−0.586	2.88e−10	0.2843	0.627	<0.2	1	MaxLD
rs115306967	32400939	C	G	0.28	0.008	9.41e−01	0.0990	<0.2	1.000	1	PC Risk
rs3129887	32410691	A	G	0.17	0.527	3.07e−05	0.6536	<0.2	0.507	0	MaxLD
rs9270804	32569422	A	T	0.28	−1.842	3.31e−98	–	<0.2	<0.2	1	Peak
rs114383918	32936607	C	A	0.08	−1.008	1.30e−08	0.1255	<0.2	<0.2	1	Flanking

HLA−DRB1
rs115306967, rs114376585

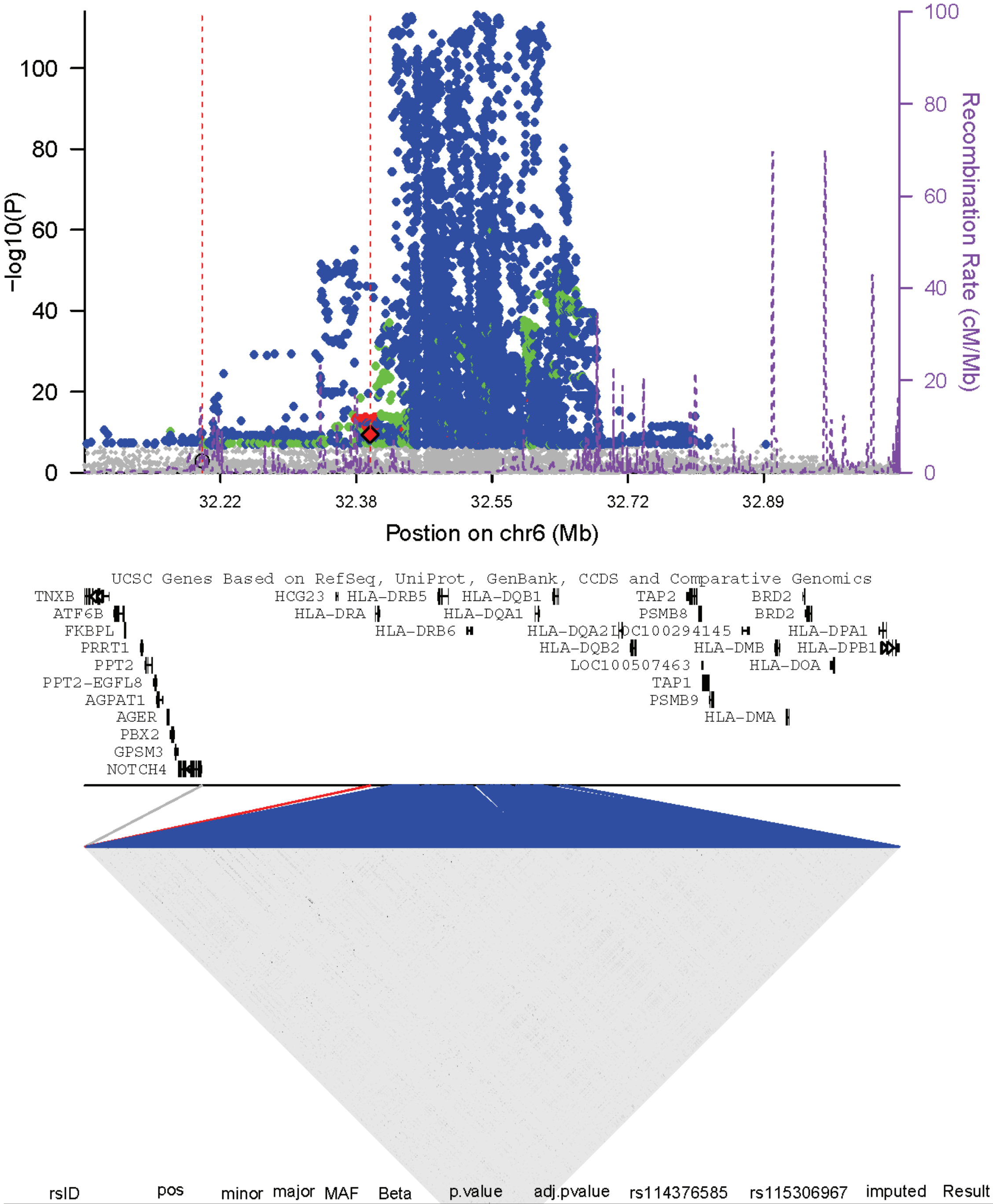

rsID	pos	minor	major	MAF	Beta	p.value	adj.pvalue	rs114376585	rs115306967	imputed	Result Type
rs2253051	31462002	C	G	0.13	−0.206	2.55e−08	0.846	<0.2	<0.2	1	Flanking
rs114376585	32192331	A	G	0.38	−0.081	1.01e−03	0.972	1.000	<0.2	0	PC Risk
rs141507970	32206103	C	G	0.37	−0.092	1.90e−04	0.117	0.627	<0.2	1	MaxLD
rs115306967	32400939	C	G	0.28	0.169	4.22e−10	0.669	<0.2	1.000	1	PC Risk
rs146320947	32534206	A	G	0.35	−0.455	7.06e−114	−	<0.2	<0.2	1	Peak
rs114143157	32601019	A	G	0.34	0.227	1.96e−18	0.609	<0.2	0.544	1	MaxLD
rs2071536	32821447	T	C	0.12	−0.214	3.83e−09	0.506	<0.2	<0.2	1	Flanking

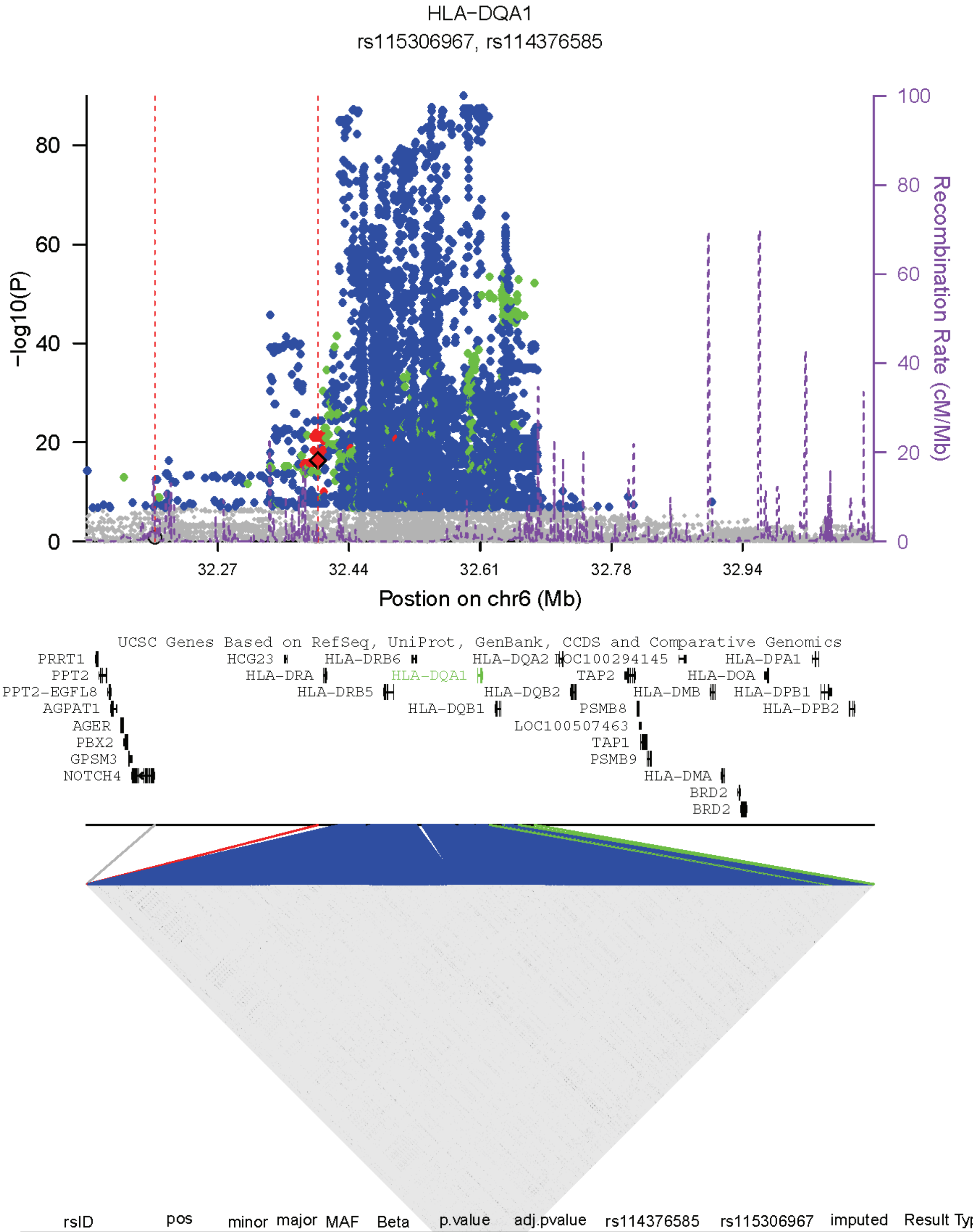

rsID	pos	minor	major	MAF	Beta	p.value	adj.pvalue	rs114376585	rs115306967	imputed	Result Type
rs112247058	31606376	C	T	0.51	−0.164	2.14e−08	0.020389	<0.2	<0.2	1	Flanking
rs114376585	32192331	A	G	0.38	−0.047	1.16e−01	0.525691	1.000	<0.2	0	PC Risk
rs141507970	32206103	C	G	0.37	−0.070	1.99e−02	0.560389	0.627	<0.2	1	MaxLD
rs115306967	32400939	C	G	0.28	0.271	4.14e−17	0.006047	<0.2	1.000	1	PC Risk
rs28383313	32587117	G	C	0.33	−0.509	1.19e−90	−	<0.2	<0.2	1	Peak
rs114143157	32601019	A	G	0.34	0.347	5.38e−30	0.001486	<0.2	0.544	1	MaxLD
rs2071556	32904601	G	T	0.37	−0.180	9.82e−09	0.000656	<0.2	<0.2	0	Flanking

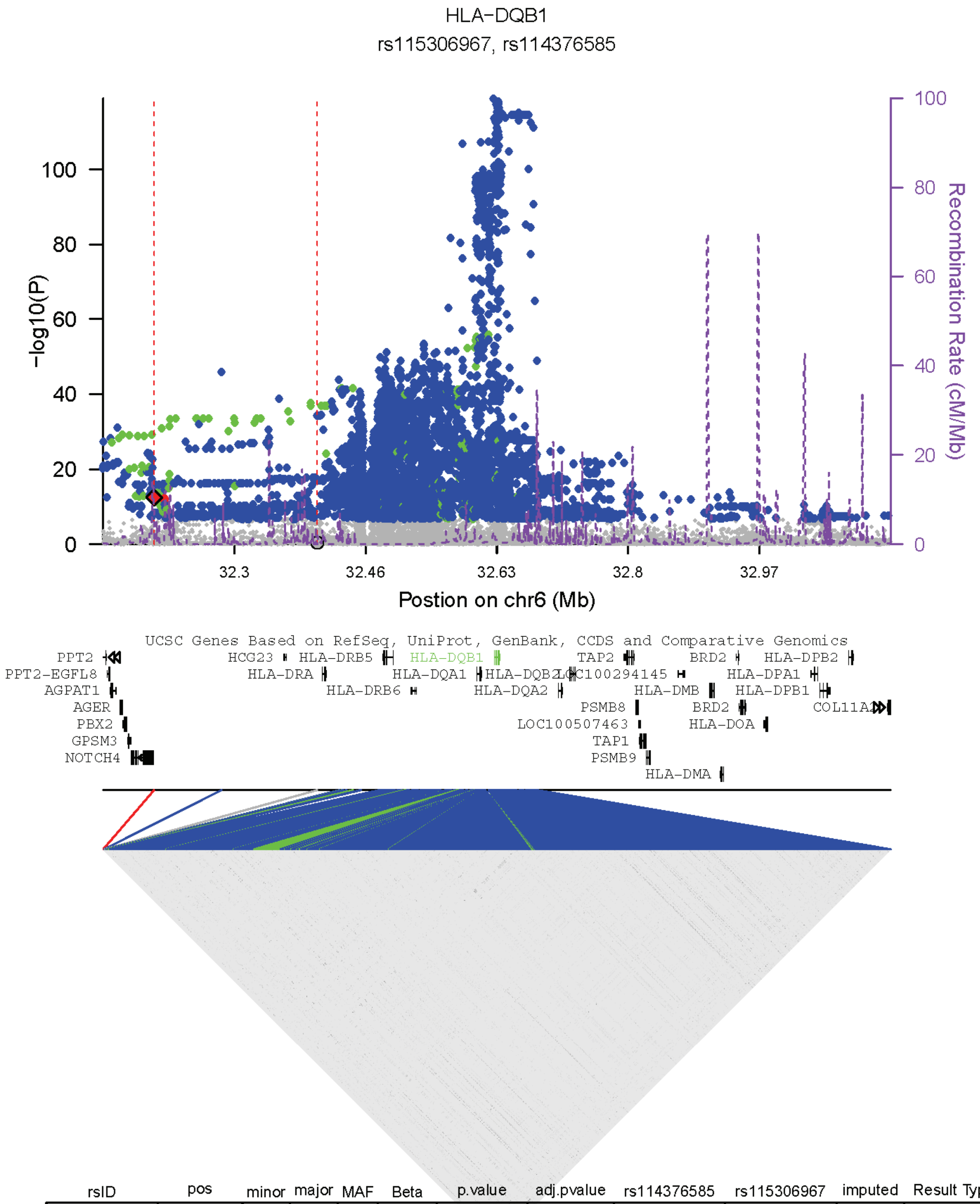

rsID	pos	minor	major	MAF	Beta	p.value	adj.pvalue	rs114376585	rs115306967	imputed	Result Type
rs2857709	31532814	A	G	0.16	0.564	1.44e−19	1.01e−01	<0.2	<0.2	0	Flanking
rs114376585	32192331	A	G	0.38	0.340	3.56e−13	3.23e−08	1.000	<0.2	0	PC Risk
rs9267853	32195251	T	C	0.38	0.342	2.65e−13	4.43e−08	0.997	<0.2	0	MaxLD
rs115306967	32400939	C	G	0.28	−0.055	3.10e−01	7.44e−01	<0.2	1.000	1	PC Risk
rs9269349	32540517	G	A	0.26	−0.242	6.98e−05	2.65e−01	<0.2	0.514	1	MaxLD
rs9273394	32627082	A	G	0.28	0.938	1.83e−119	−	<0.2	<0.2	1	Peak
rs114554250	33196570	A	G	0.04	−0.616	2.86e−08	1.07e−07	<0.2	<0.2	1	Flanking

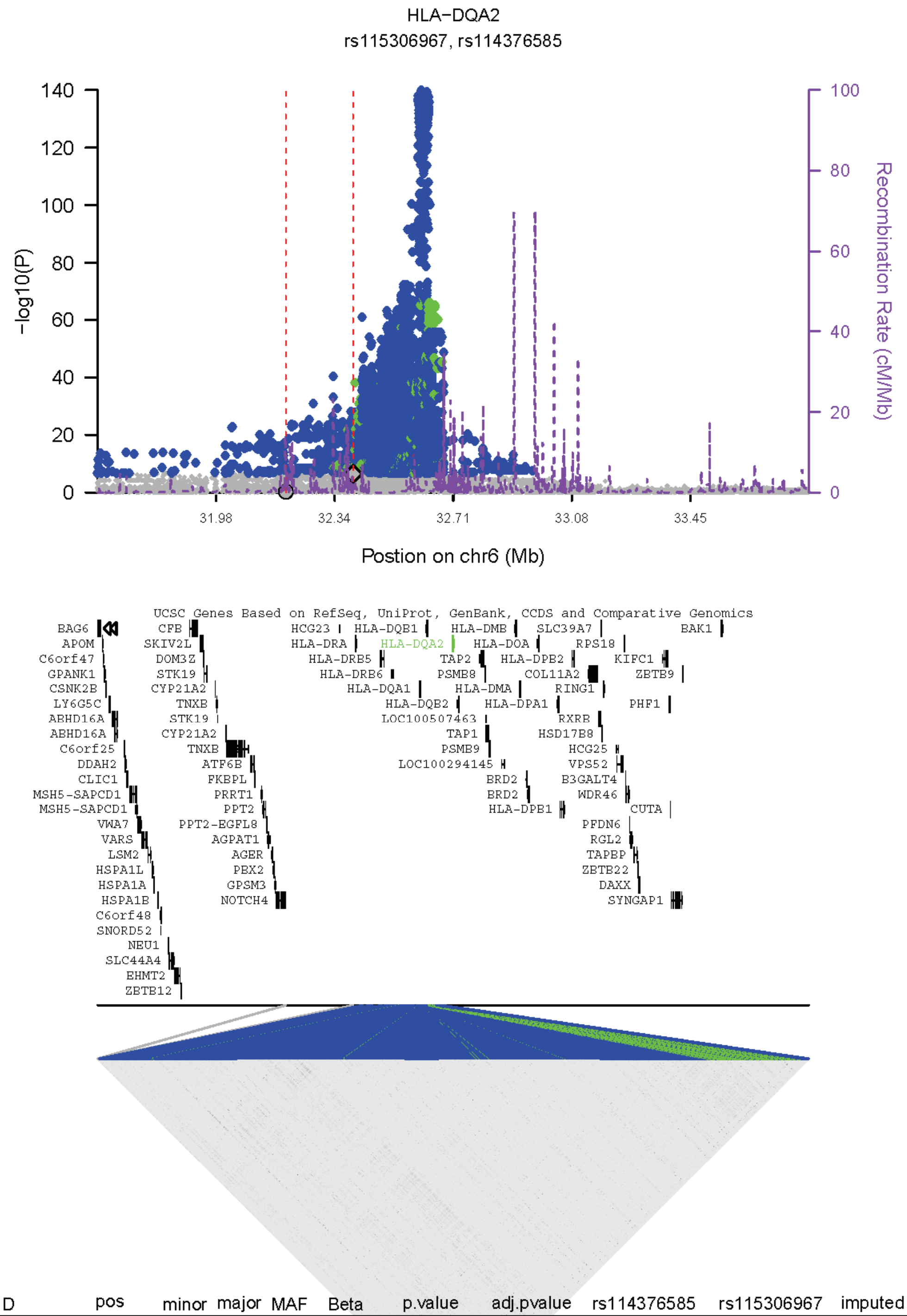

rsID	pos	minor	major	MAF	Beta	p.value	adj.pvalue	rs114376585	rs115306967	imputed	Result Type
rs114558910	31611158	T	C	0.20	−0.872	3.58e−12	2.52e−01	<0.2	<0.2	1	Flanking
rs114376585	32192331	A	G	0.38	−0.094	3.70e−01	2.55e−02	1.000	<0.2	0	PC Risk
rs141507970	32206103	C	G	0.37	−0.374	3.53e−04	4.86e−01	0.627	<0.2	1	MaxLD
rs115306967	32400939	C	G	0.28	−0.589	3.42e−07	2.18e−01	<0.2	1.000	1	PC Risk
rs114143157	32601019	A	G	0.34	−0.812	2.37e−13	8.55e−01	<0.2	0.544	1	MaxLD
rs6927022	32612397	G	A	0.47	1.683	2.01e−86	6.80e−09	<0.2	<0.2	1	ReverseBeta
rs9273123	32612733	G	A	0.41	−2.037	1.89e−140	−	<0.2	<0.2	1	Peak
rs115660687	32938199	A	G	0.06	−1.167	1.23e−08	7.45e−03	<0.2	<0.2	1	Flanking

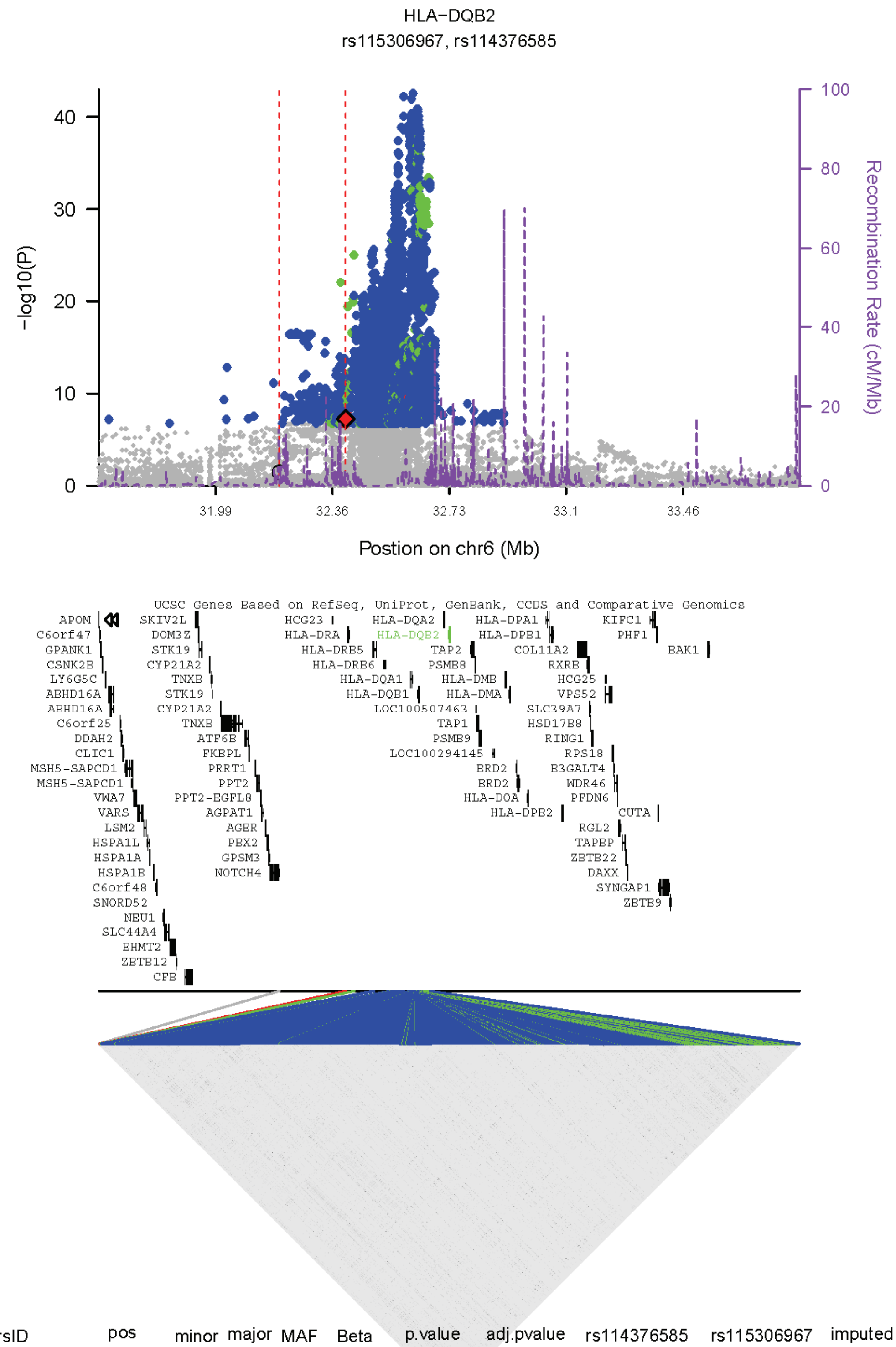

rsID	pos	minor	major	MAF	Beta	p.value	adj.pvalue	rs114376585	rs115306967	imputed	Result Type
rs115521560	32019382	C	A	0.07	0.496	4.65e−10	1.47e−05	<0.2	<0.2	0	Flanking
rs114376585	32192331	A	G	0.38	0.091	3.09e−02	8.28e−09	1.000	<0.2	0	PC Risk
rs115306967	32400939	C	G	0.28	−0.252	5.80e−08	2.30e−02	<0.2	1.000	1	PC Risk
rs114143157	32601019	A	G	0.34	−0.283	3.60e−10	4.60e−03	<0.2	0.544	1	MaxLD
rs17612669	32615816	G	A	0.35	−0.566	2.82e−43	−	<0.2	<0.2	1	Peak
rs154978	32899296	C	T	0.46	−0.240	1.67e−08	5.56e−04	<0.2	<0.2	0	Flanking

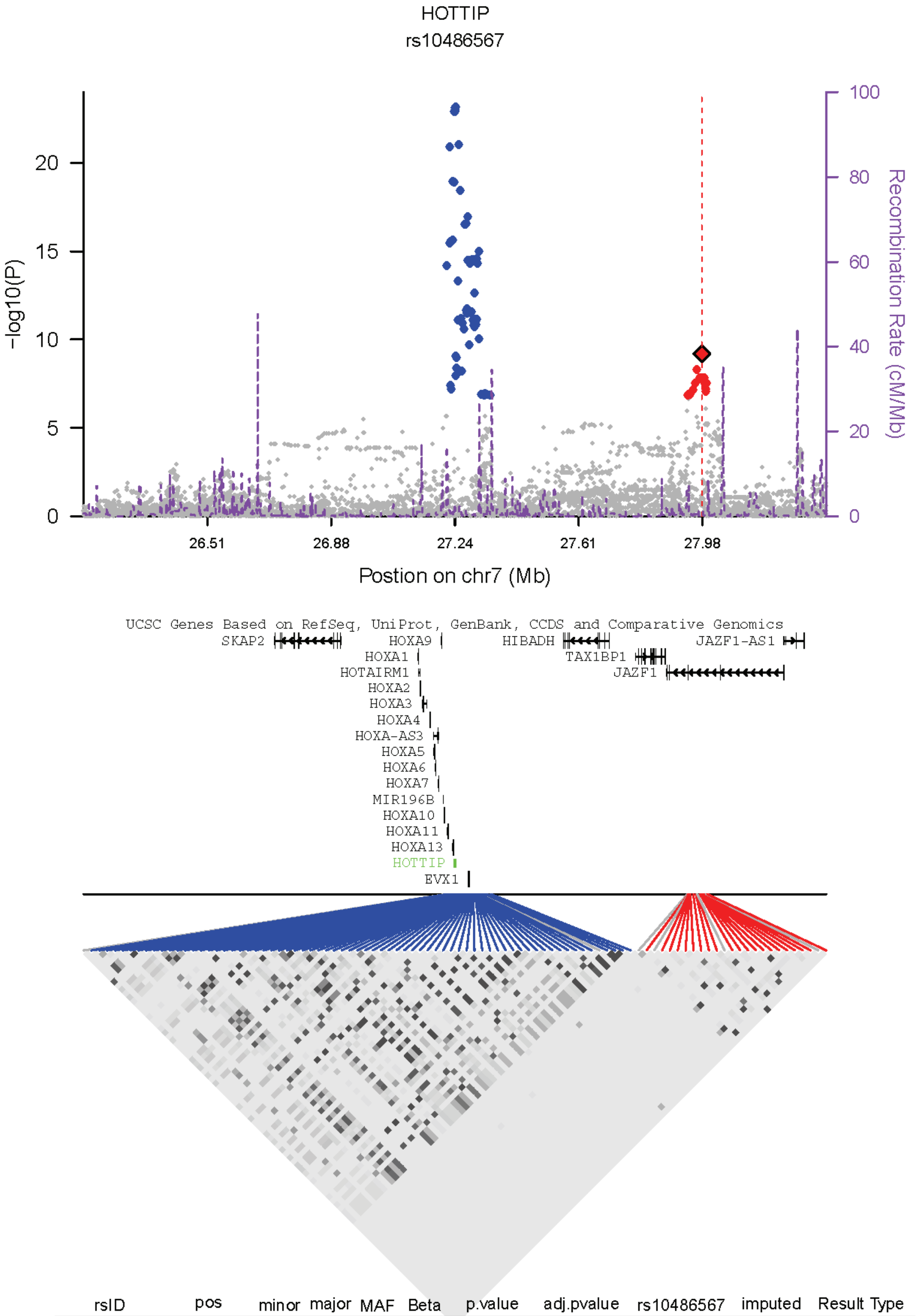

rsID	pos	minor	major	MAF	Beta	p.value	adj.pvalue	rs10486567	imputed	Result Type
rs6461992	27220831	A	G	0.08	0.314	6.42e−15	4.56e−01	<0.2	0	Flanking
rs3735533	27245893	T	C	0.07	0.423	7.14e−24	–	<0.2	1	Peak
rs10486567	27976563	A	G	0.23	0.161	6.44e−10	5.37e−11	1.000	0	PC Risk
rs7808935	27977363	C	T	0.23	0.161	6.17e−10	5.18e−11	1.000	1	MaxLD
rs10807843	27992390	C	T	0.21	0.150	3.09e−08	3.02e−10	0.910	1	Flanking

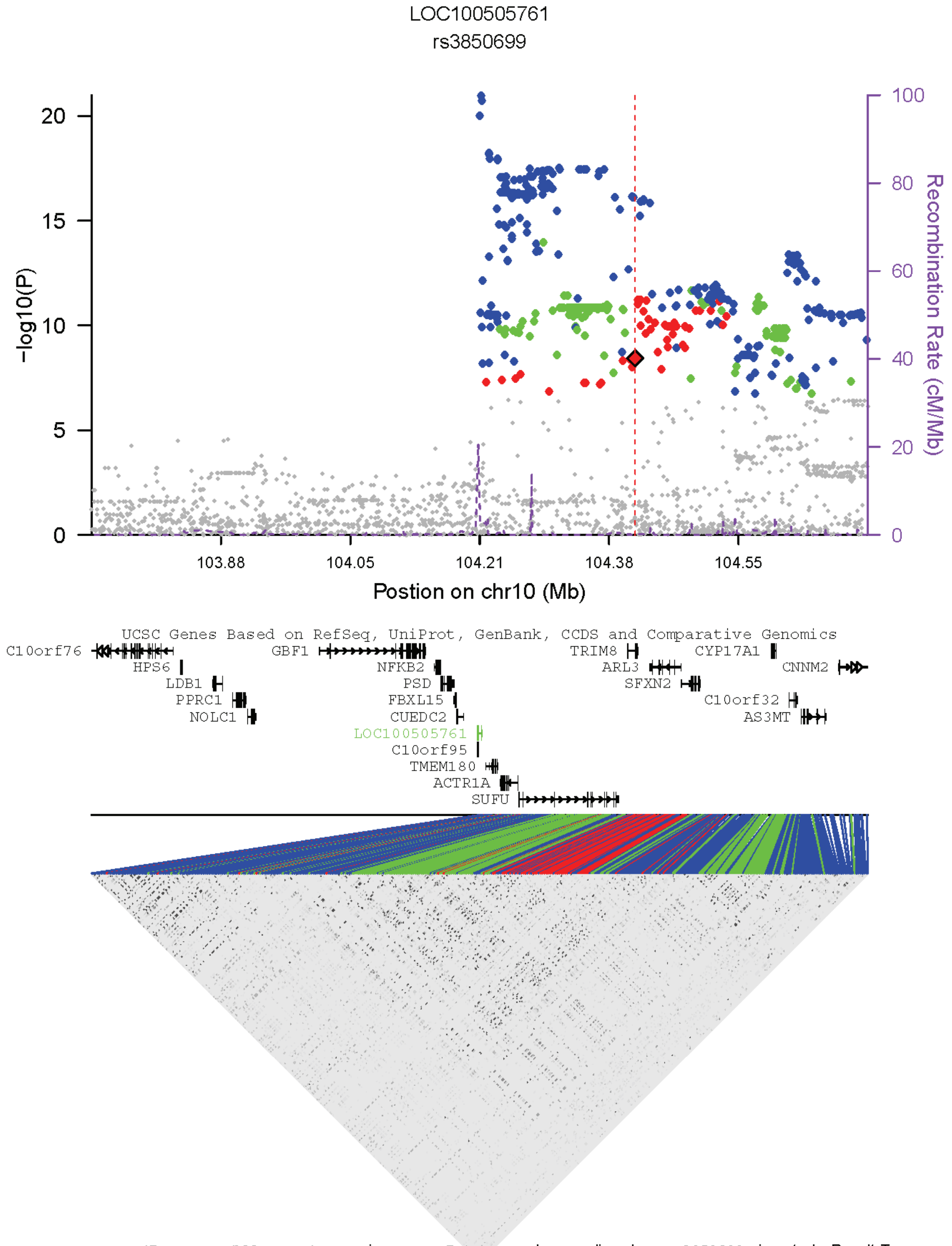

rsID	pos	minor	major	MAF	Beta	p.value	adj.pvalue	rs3850699	imputed	Result Type
rs11191291	104213502	T	C	0.23	0.132	9.78e−21	3.27e−02	<0.2	0	Flanking
rs1064072	104215617	G	C	0.28	0.126	1.12e−21	−	<0.2	1	Peak
rs3850699	104414221	G	A	0.27	−0.083	3.72e−09	4.87e−09	1.000	0	PC Risk
rs12773833	104418350	T	C	0.29	−0.093	6.28e−12	7.69e−11	0.914	1	MaxLD
rs4307650	104959852	A	C	0.38	−0.080	9.19e−11	1.28e−06	<0.2	1	Flanking

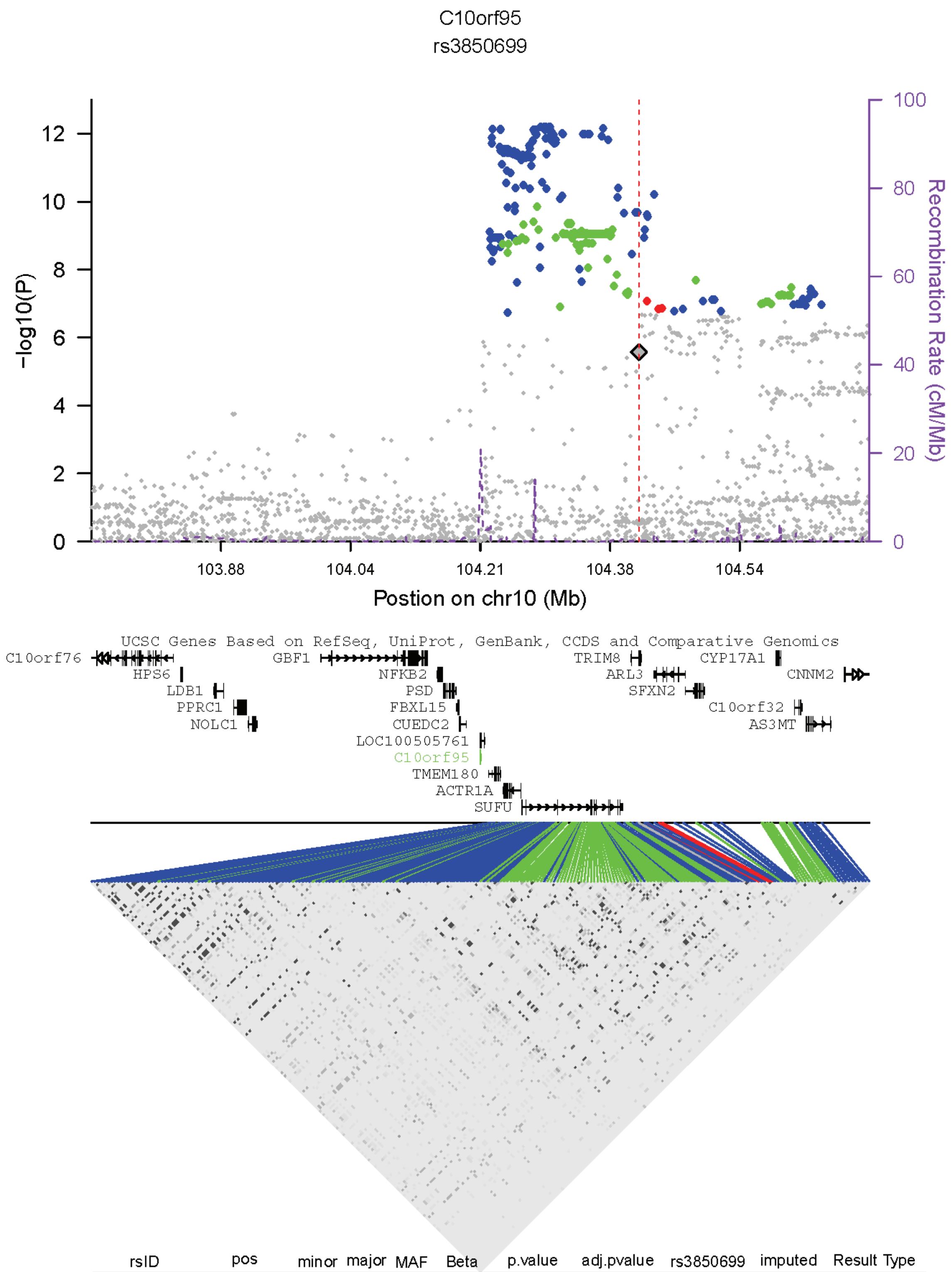

rsID	pos	minor	major	MAF	Beta	p.value	adj.pvalue	rs3850699	imputed	Result Type
rs12570201	104222963	T	C	0.49	0.136	7.91e−10	0.18534	<0.2	1	Flanking
rs7896841	104294665	T	C	0.36	0.164	6.43e−13	–	<0.2	1	Peak
rs3850699	104414221	G	A	0.27	−0.116	2.70e−06	0.02622	1.000	0	PC Risk
rs7916092	104425471	C	T	0.30	−0.128	8.62e−08	0.00771	0.900	1	MaxLD
rs2265309	104487871	T	C	0.46	−0.121	2.11e−08	0.05104	0.311	1	Flanking

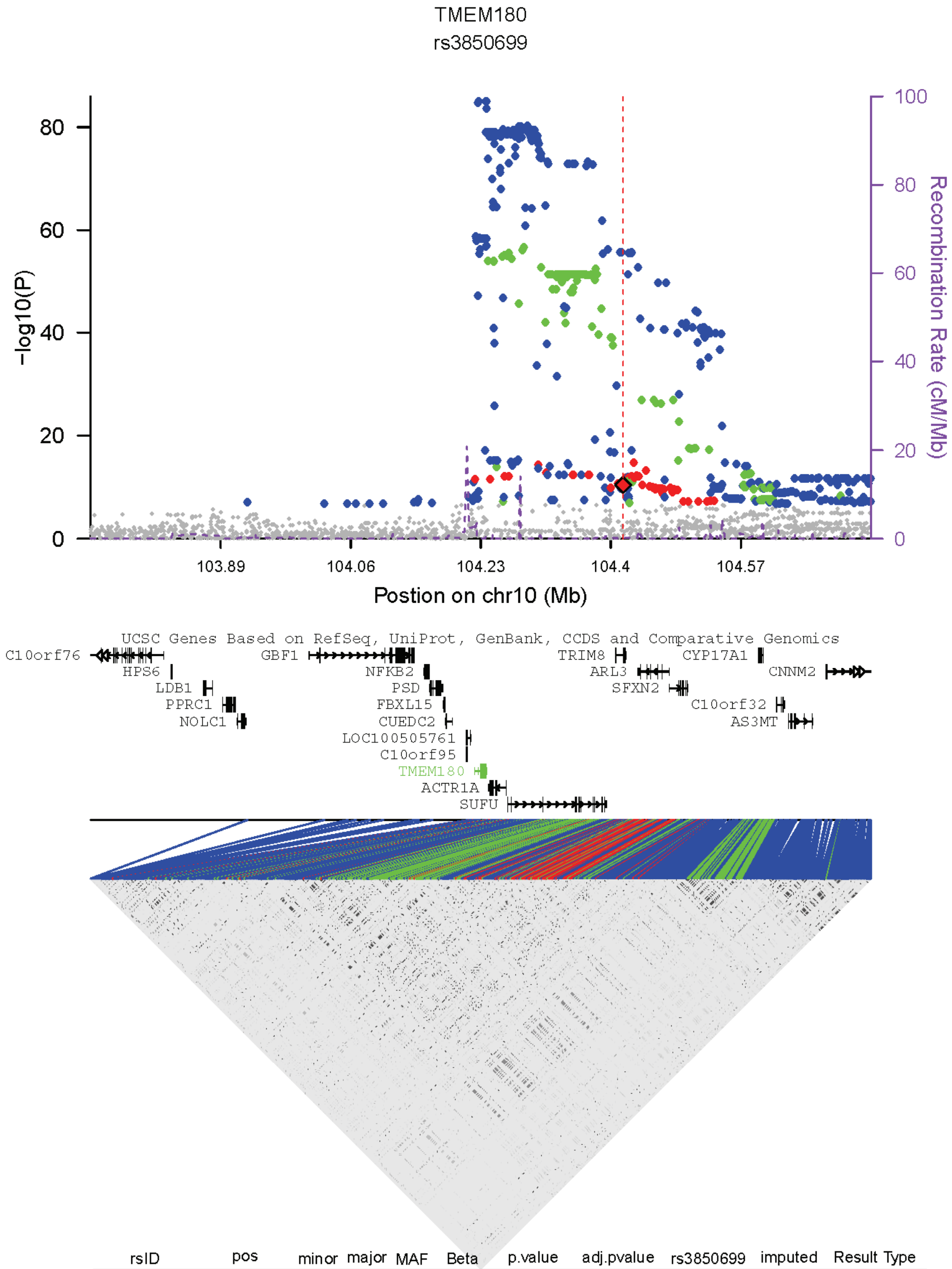

rsID	pos	minor	major	MAF	Beta	p.value	adj.pvalue	rs3850699	imputed	Result Type
rs12360330	104148355	T	C	0.14	−0.195	2.68e−08	0.07075	<0.2	1	Flanking
rs61873704	104236929	C	T	0.37	0.413	9.37e−86	–	<0.2	1	Peak
rs3850699	104414221	G	A	0.27	−0.179	4.00e−11	0.21752	1.000	0	PC Risk
rs12262998	104428716	T	C	0.30	−0.207	1.63e−15	0.08981	0.823	1	MaxLD
rs4311992	105120649	A	G	0.23	0.155	2.45e−08	0.00075	<0.2	1	Flanking

SFXN2
rs3850699

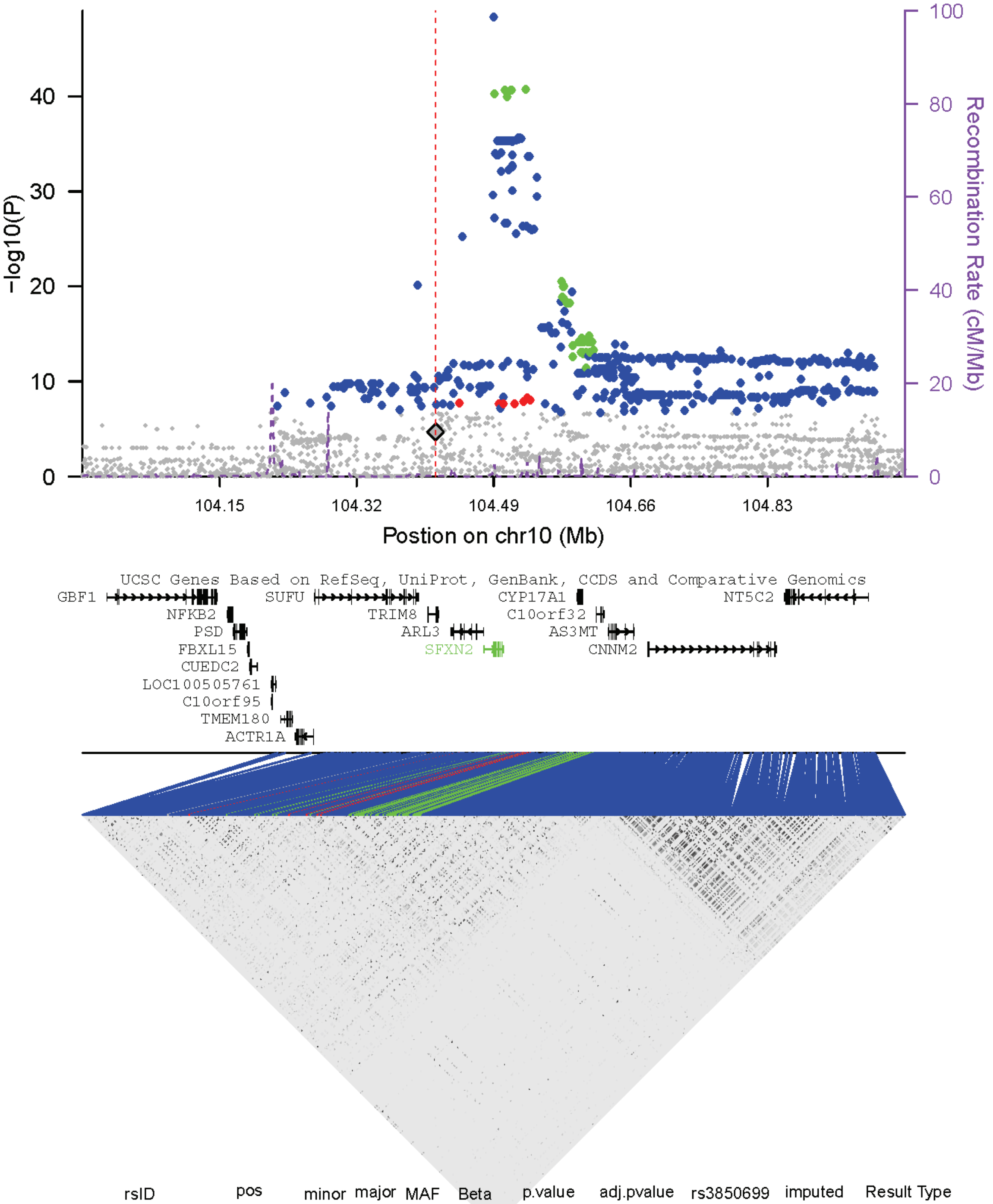

rsID	pos	minor	major	MAF	Beta	p.value	adj.pvalue	rs3850699	imputed	Result Type
rs118028082	104226834	A	G	0.07	−0.184	1.54e−09	5.64e−01	<0.2	1	Flanking
rs3850699	104414221	G	A	0.27	−0.076	2.02e−05	5.10e−27	1.000	0	PC Risk
rs2902548	104487382	T	C	0.17	−0.273	5.22e−49	–	<0.2	0	Peak
rs7918080	104517826	C	G	0.22	0.221	2.56e−36	1.20e−35	<0.2	1	ReverseBeta
rs11191394	104528509	C	A	0.28	−0.098	6.04e−09	2.89e−41	0.607	1	MaxLD
rs113278154	104960464	T	C	0.09	−0.161	1.23e−09	3.43e−01	<0.2	1	Flanking

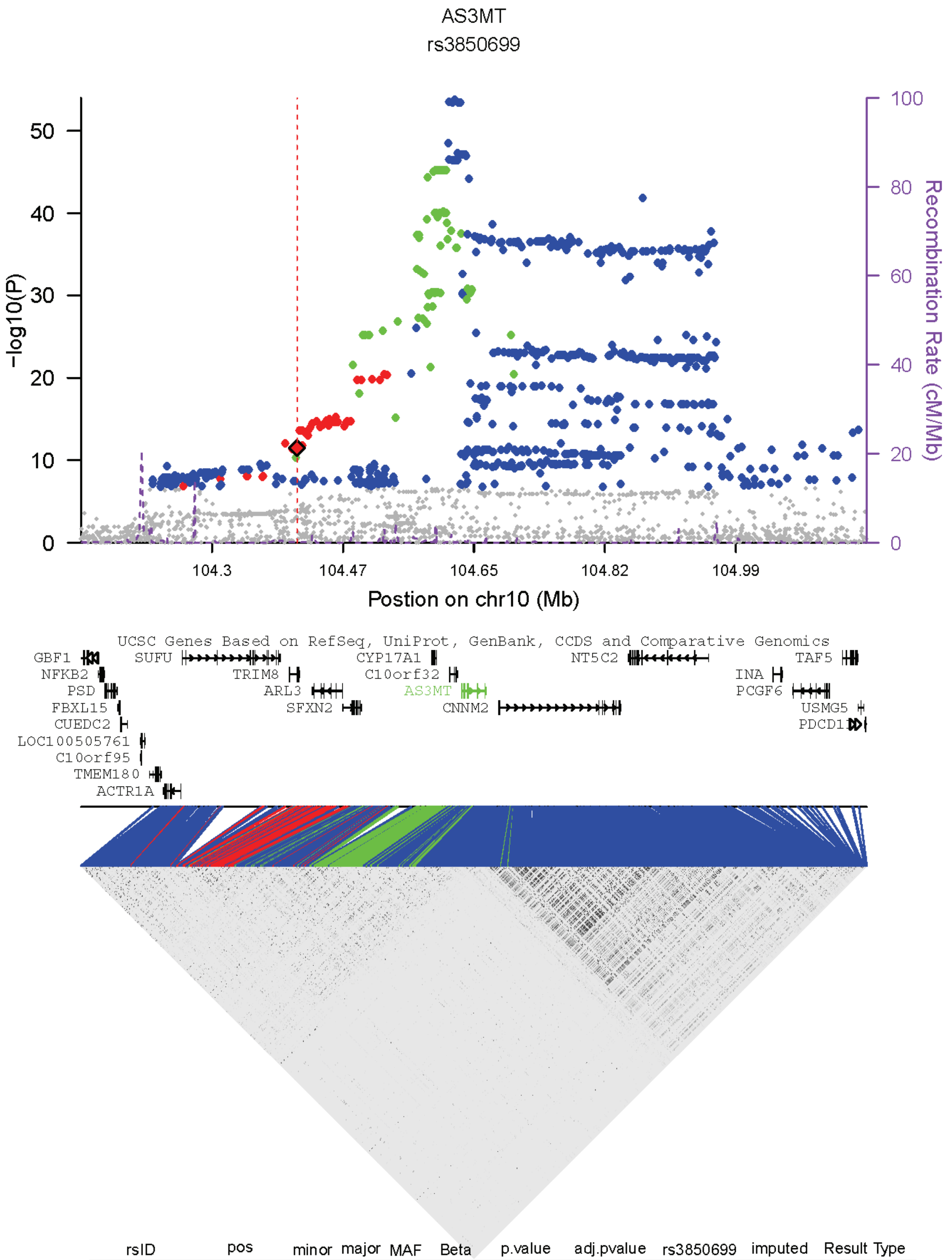

rsID	pos	minor	major	MAF	Beta	p.value	adj.pvalue	rs3850699	imputed	Result Type
rs11191294	104225837	G	T	0.37	0.196	2.50e−08	0.390	<0.2	1	Flanking
rs3850699	104414221	G	A	0.27	−0.260	3.05e−12	0.309	1.000	0	PC Risk
rs11191394	104528509	C	A	0.28	−0.331	3.77e−21	0.127	0.607	1	MaxLD
rs12241517	104621068	G	A	0.33	−0.471	1.75e−54	–	<0.2	1	Peak
rs3740478	105205356	T	C	0.06	−0.390	1.86e−08	0.832	<0.2	0	Flanking

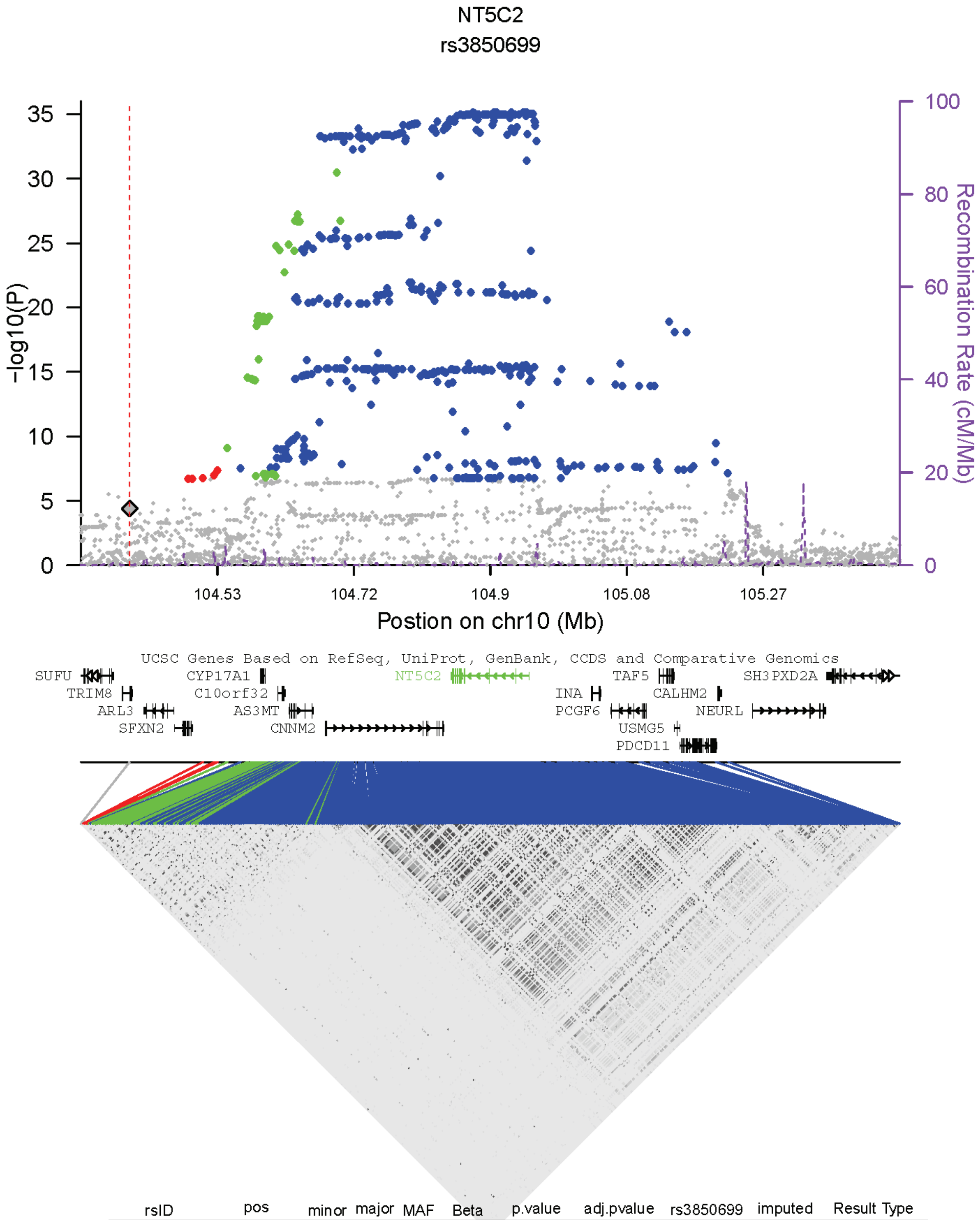

rsID	pos	minor	major	MAF	Beta	p.value	adj.pvalue	rs3850699	imputed	Result Type
rs3850699	104414221	G	A	0.27	−0.032	4.00e−05	0.3763	1.000	0	PC Risk
rs11191381	104493444	T	C	0.29	−0.038	1.95e−07	0.2684	0.602	0	Flanking
rs12776506	104532828	G	A	0.29	−0.040	4.71e−08	0.3554	0.603	1	MaxLD
rs7902218	104740210	A	G	0.47	0.048	3.83e−13	0.8366	<0.2	1	ReverseBeta
rs2066322	104909339	G	T	0.29	−0.084	8.12e−36	−	<0.2	1	Peak
rs3740478	105205356	T	C	0.06	−0.089	3.67e−10	0.0604	<0.2	0	Flanking

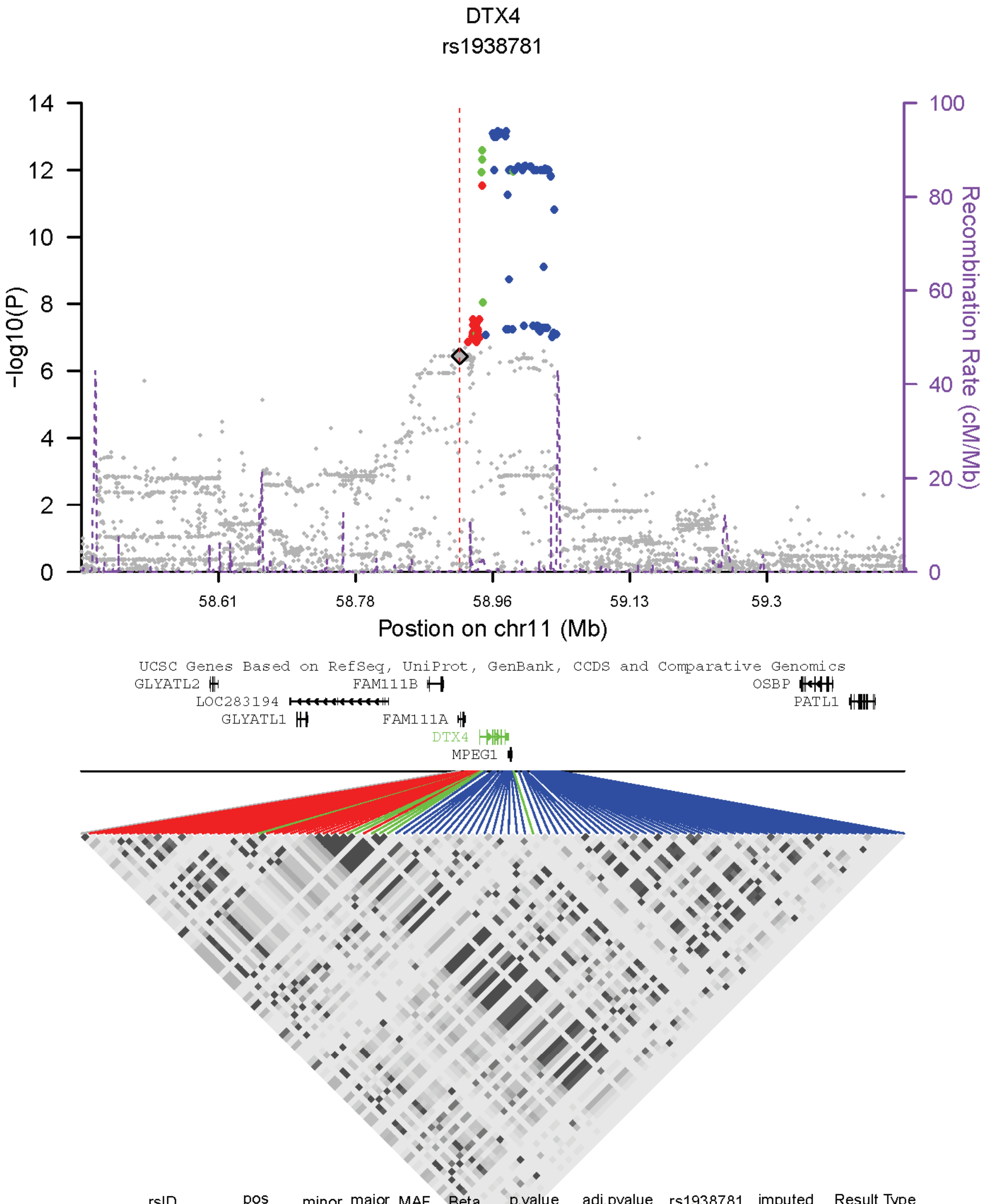

rsID	pos	minor	major	MAF	Beta	p.value	adj.pvalue	rs1938781	imputed	Result Type
rs1938781	58915110	G	A	0.22	0.103	3.64e−07	1.16e−06	1.000	1	PC Risk
rs17153415	58925860	G	A	0.21	0.107	1.42e−07	6.06e−07	0.947	1	Flanking
rs7934971	58943442	T	G	0.32	0.122	2.97e−12	5.06e−11	0.504	1	MaxLD
rs12284698	58962967	C	T	0.02	0.493	7.14e−14	–	<0.2	0	Peak
rs75112843	58973623	G	A	0.02	0.493	7.14e−14	8.92e−01	<0.2	0	Peak
rs12791481	58977372	G	T	0.47	−0.105	1.89e−09	1.53e−07	<0.2	1	ReverseBeta
rs11229905	59034323	G	A	0.01	0.500	1.56e−11	7.00e−01	<0.2	1	Flanking

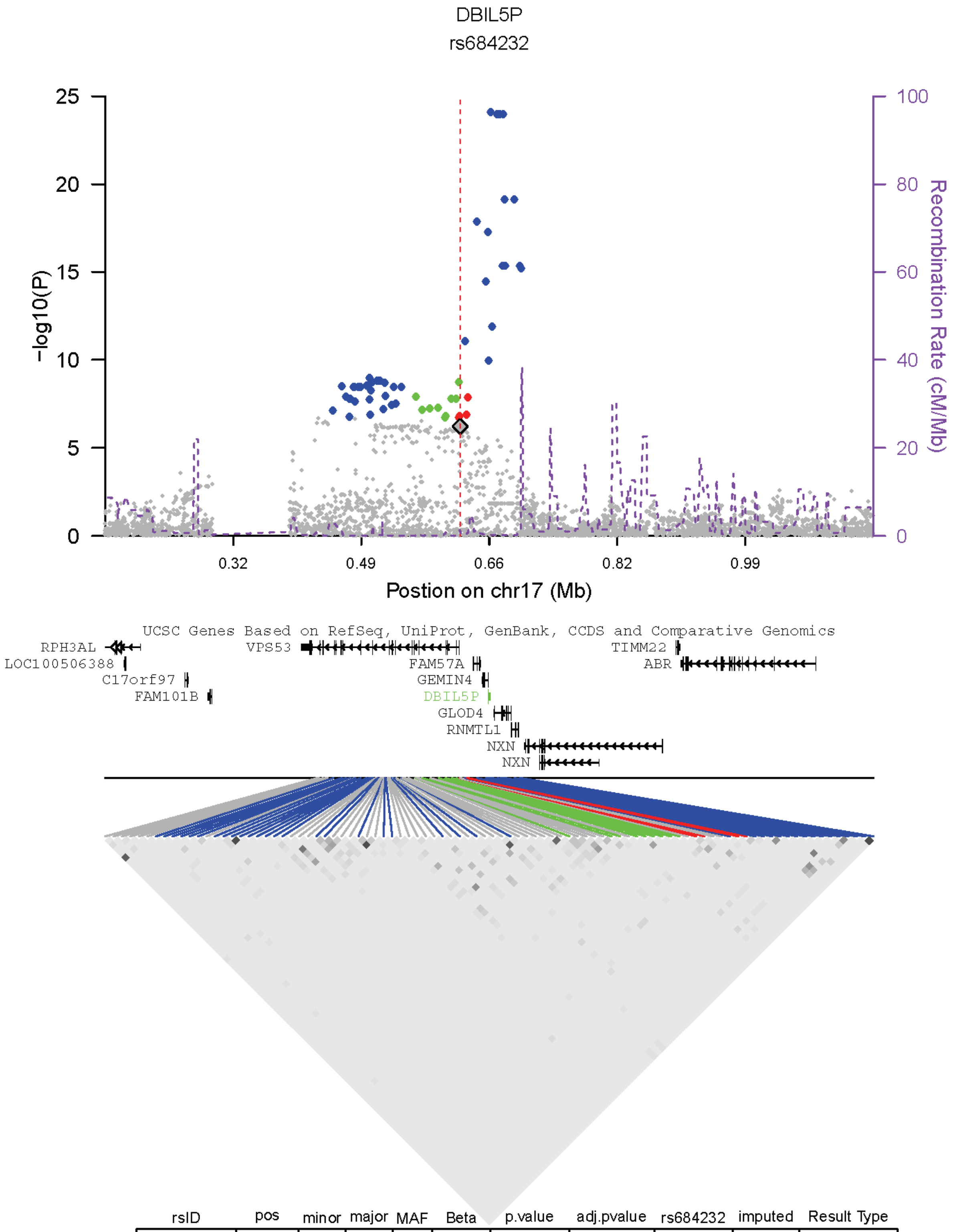

rsID	pos	minor	major	MAF	Beta	p.value	adj.pvalue	rs684232	imputed	Result Type
rs12948505	464962	T	C	0.18	0.205	3.35e−09	2.69e−09	<0.2	1	Flanking
rs684232	618965	C	T	0.39	−0.144	5.95e−07	1.27e−05	1.000	0	PC Risk
rs2740360	629309	T	C	0.46	0.167	1.34e−08	1.00e−04	0.518	1	MaxLD
rs2750006	659371	A	G	0.03	0.761	8.46e−25	−	<0.2	1	Peak
rs75504469	698635	T	C	0.02	0.833	6.65e−16	7.76e−01	<0.2	1	Flanking

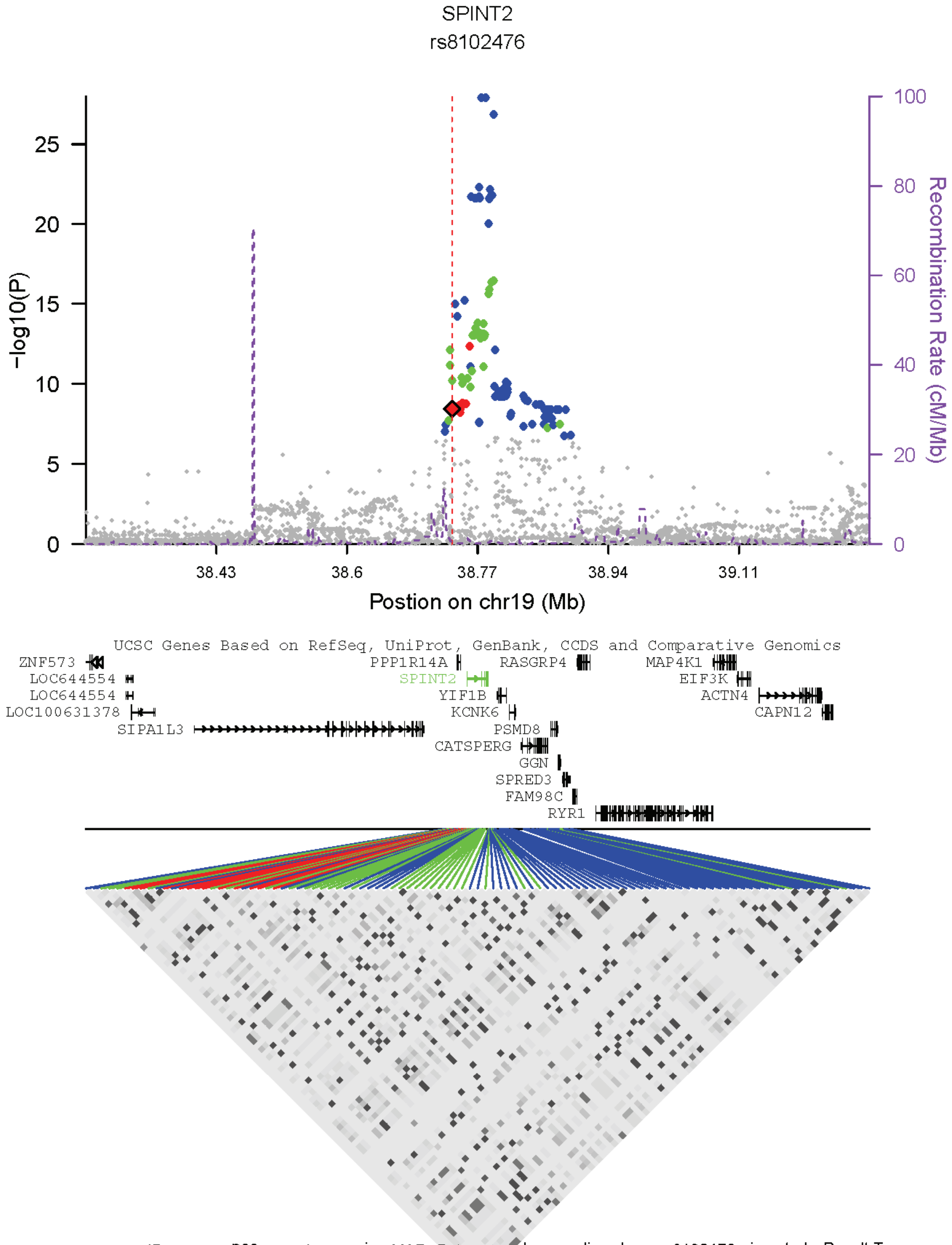

rsID	pos	minor	major	MAF	Beta	p.value	adj.pvalue	rs8102476	imputed	Result Type
rs7246060	38731662	A	G	0.33	0.059	2.04e−08	0.020337	0.231	1	Flanking
rs8102476	38735613	T	C	0.43	0.058	3.56e−09	0.040741	1.000	0	PC Risk
rs3786877	38759180	C	T	0.41	0.072	4.68e−13	0.000645	0.563	0	MaxLD
rs2060244	38774622	T	C	0.10	0.165	1.31e−28	–	<0.2	1	Peak
rs3745949	38779912	G	A	0.10	0.165	1.31e−28	0.744940	<0.2	1	Peak
rs79210197	38884909	T	A	0.04	0.157	4.12e−09	0.535215	<0.2	0	Flanking

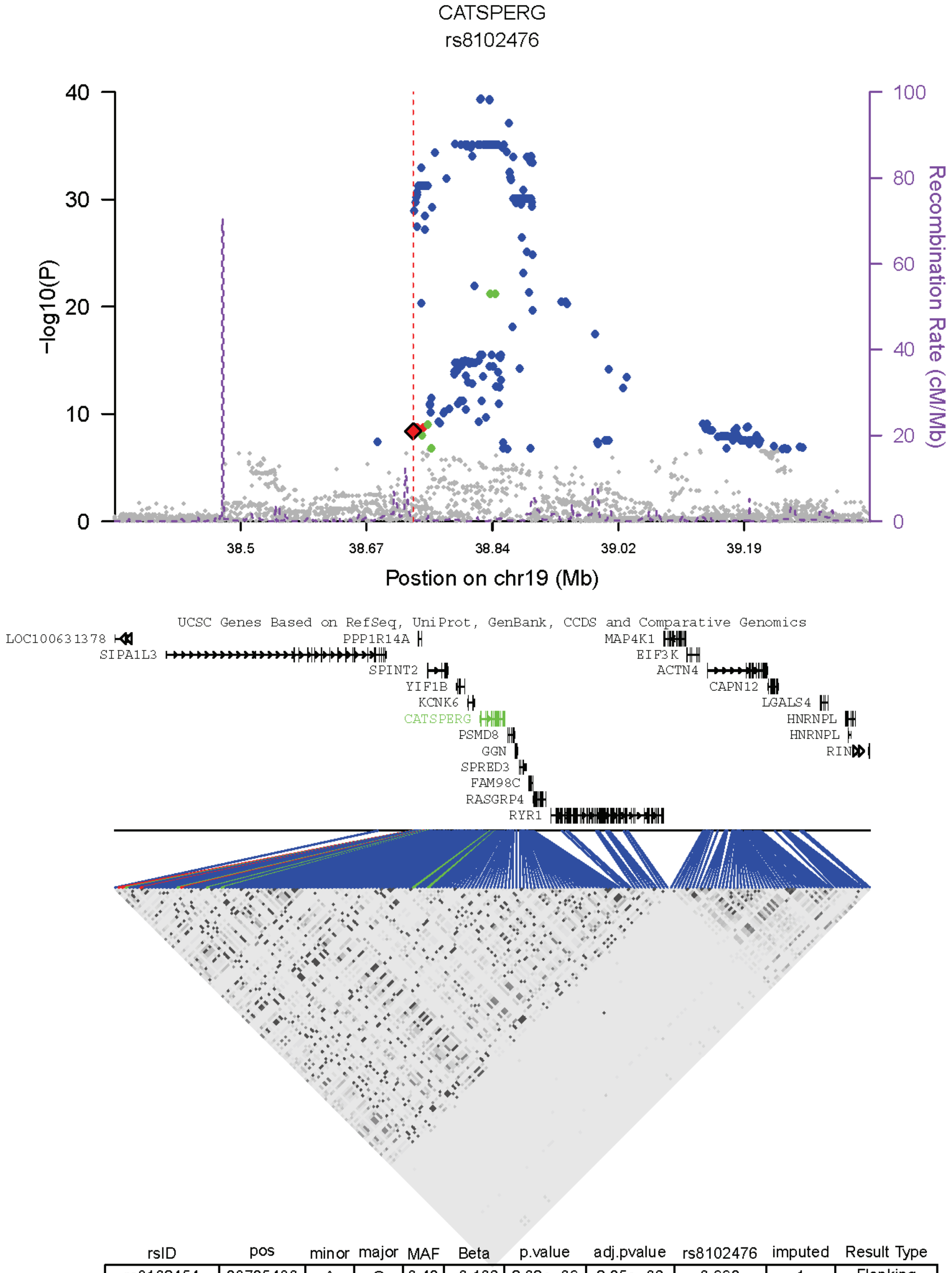

rsID	pos	minor	major	MAF	Beta	p.value	adj.pvalue	rs8102476	imputed	Result Type
rs8102454	38735480	A	G	0.43	−0.106	2.02e−09	2.35e−06	0.993	1	Flanking
rs8102476	38735613	T	C	0.43	−0.105	4.01e−09	2.01e−06	1.000	0	PC Risk
rs12981216	38740925	T	C	0.43	−0.106	1.99e−09	2.39e−06	0.987	1	MaxLD
rs113370094	38828509	C	A	0.03	0.614	4.56e−40	−	<0.2	1	Peak
rs62119116	39210673	T	C	0.06	0.200	2.98e−08	2.52e−02	<0.2	1	Flanking

TMPRSS2
rs1041449

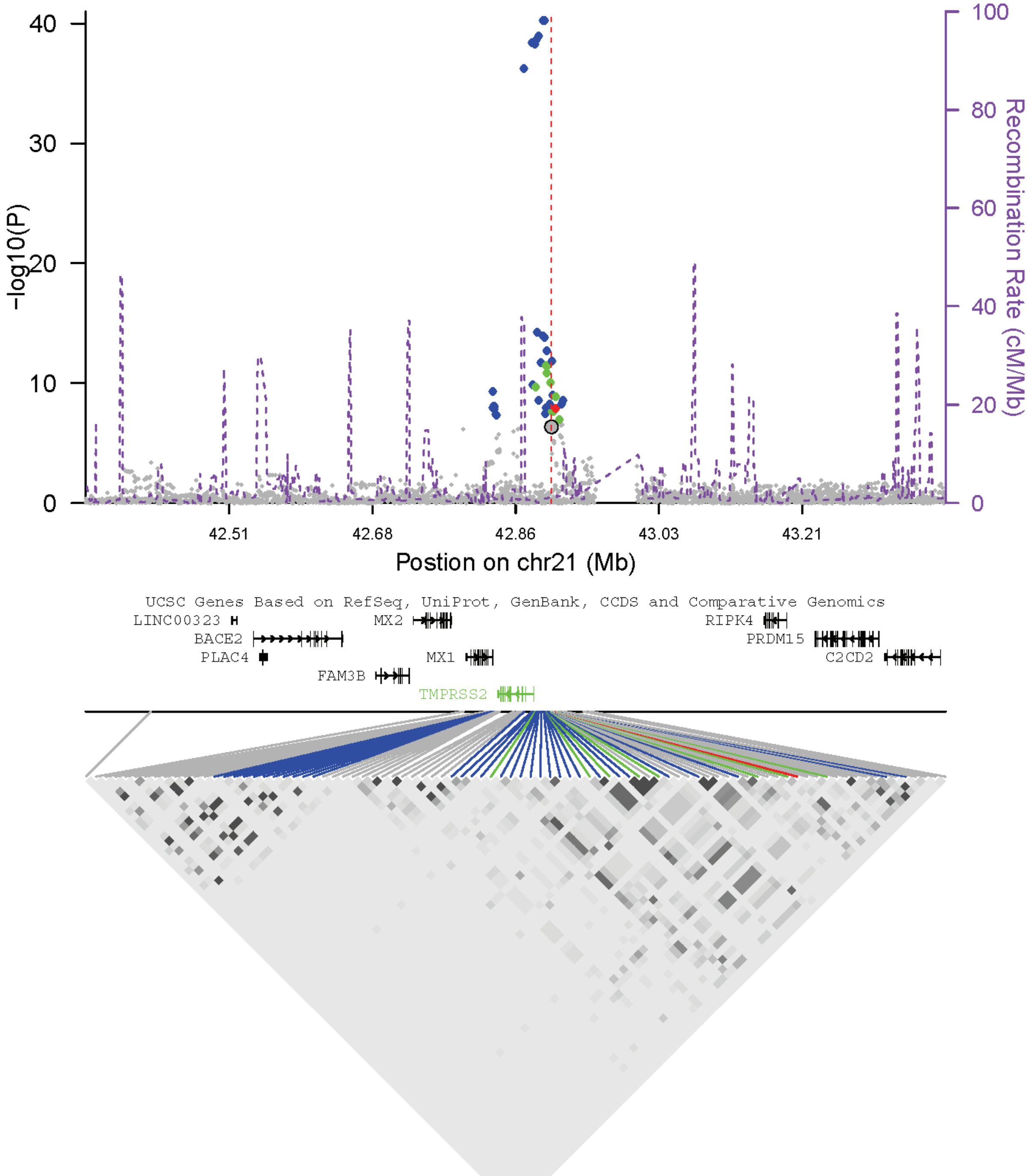

rsID	pos	minor	major	MAF	Beta	p.value	adj.pvalue	rs1041449	imputed	Result Type
rs1557370	42830690	G	A	0.07	−0.260	5.40e−10	0.000341	<0.2	0	Flanking
rs6517673	42892056	A	G	0.08	−0.490	5.59e−41	–	<0.2	0	Peak
rs8134657	42893908	A	G	0.08	−0.490	5.59e−41	0.961031	<0.2	0	Peak
rs1041449	42901421	G	A	0.47	0.106	4.08e−07	0.075963	1.000	0	PC Risk
rs9979125	42907168	T	C	0.48	0.120	1.32e−08	0.025171	0.897	1	MaxLD
rs78831106	42915890	A	C	0.02	−0.553	2.99e−09	0.949788	<0.2	1	Flanking

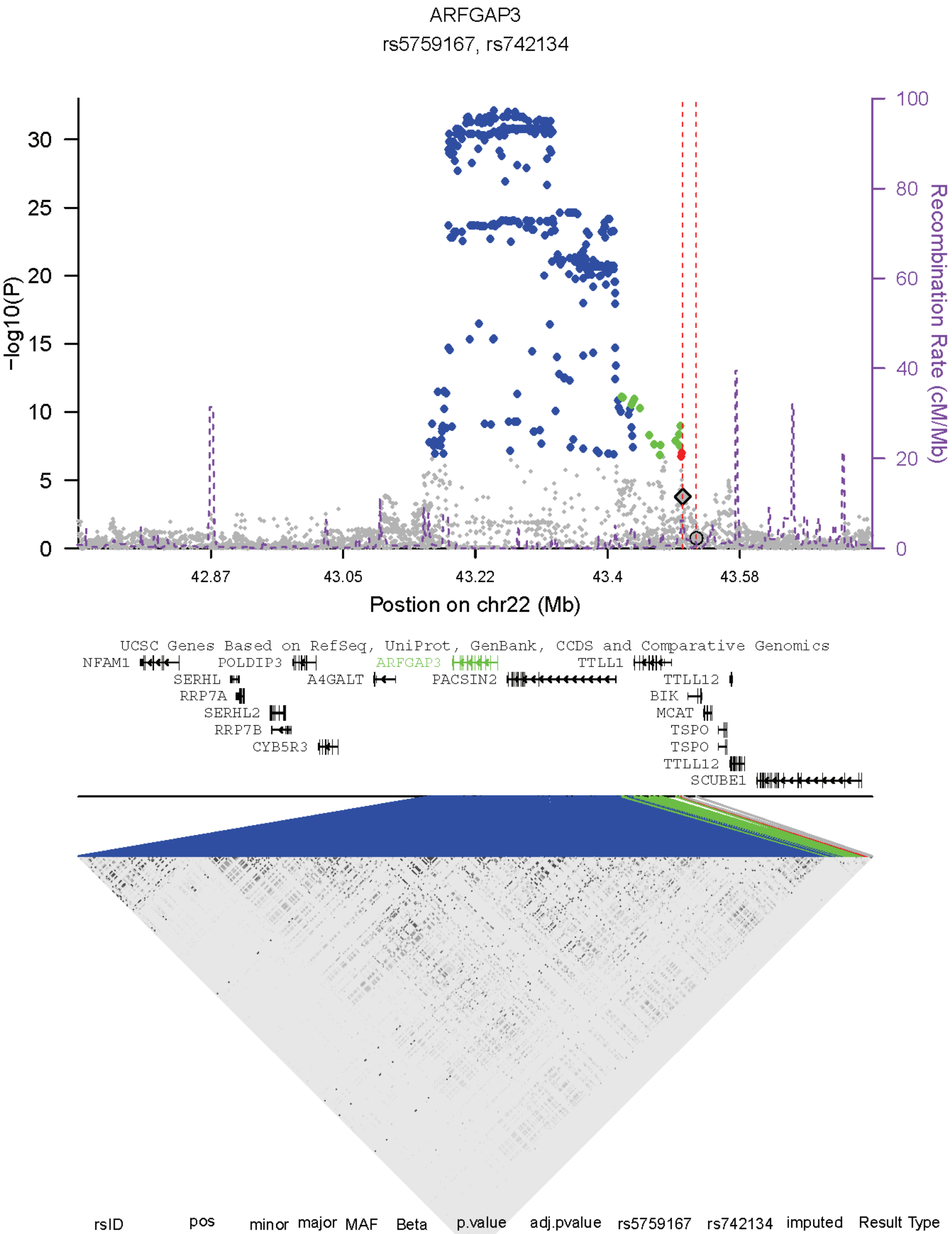

rsID	pos	minor	major	MAF	Beta	p.value	adj.pvalue	rs5759167	rs742134	imputed	Result Type
rs13058545	43162106	C	G	0.41	−0.060	1.64e−08	0.276	<0.2	<0.2	1	Flanking
rs2899364	43248411	G	T	0.43	0.120	7.87e−33	−	<0.2	<0.2	1	Peak
rs9612008	43499144	A	G	0.45	−0.054	1.02e−07	0.101	0.665	<0.2	1	MaxLD
rs5759167	43500212	T	G	0.49	−0.038	1.61e−04	0.187	1.000	<0.2	0	PC Risk
rs742134	43518275	A	G	0.11	0.024	1.66e−01	0.939	<0.2	1.000	1	PC Risk
rs5759182	43539651	G	A	0.11	0.029	8.61e−02	0.774	<0.2	0.950	0	MaxLD

Supplementary Figure 5 - Distributions of the minimal size of eQTL peak

For each of the 88 significant target genes, the minimal size of the eQTL peak was determined by taking the distance between the endpoints of the highest cluster of significant SNPs. The distribution is plotted with Kb of the peak on the y-axis and sorted by size. The inset plot displays all regions with size < 50Kb.

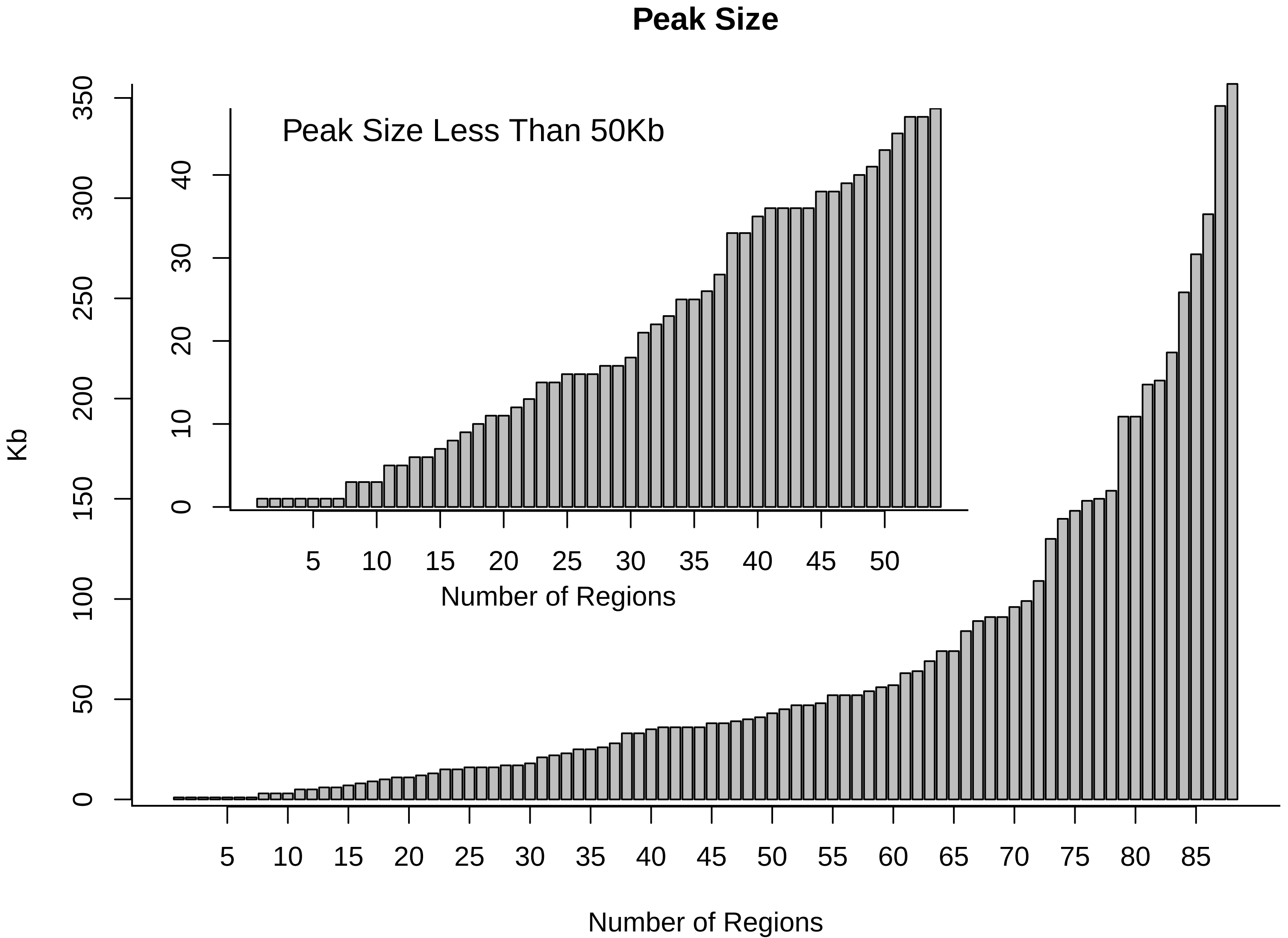
Peak Size
Kb
Number of Regions
Peak Size Less Than 50Kb
Number of Regions
0
50
100
150
200
250
300
350
5
10
15
20
25
30
35
40
45
50
55
60
65
70
75
80
85
0
10
20
30
40
5
10
15
20
25
30
35
40
45
50

Supplementary Figure 6 - Mapping of candidate regulatory domains and candidate causative SNPS responsible for *CTBP2* gene dysregulation

Panel A shows the regional association plot for *CTBP2* over a 400Kb region. The peak eQTL region explored further (results shown in panels A and C) is approximately 15 kb and is outlined by a red box. The characteristics of the plot are described in figure 1.

Panel B shows the UCSC genome browser for the 15Kb peak eQTL region (as outlined in panel B), providing a number of tracks used to map elements important for gene regulatory domains as described in Hazelett et. al.[1] The 24 peak eQTL SNPs are shown in the first track as reference points (see table for identification).

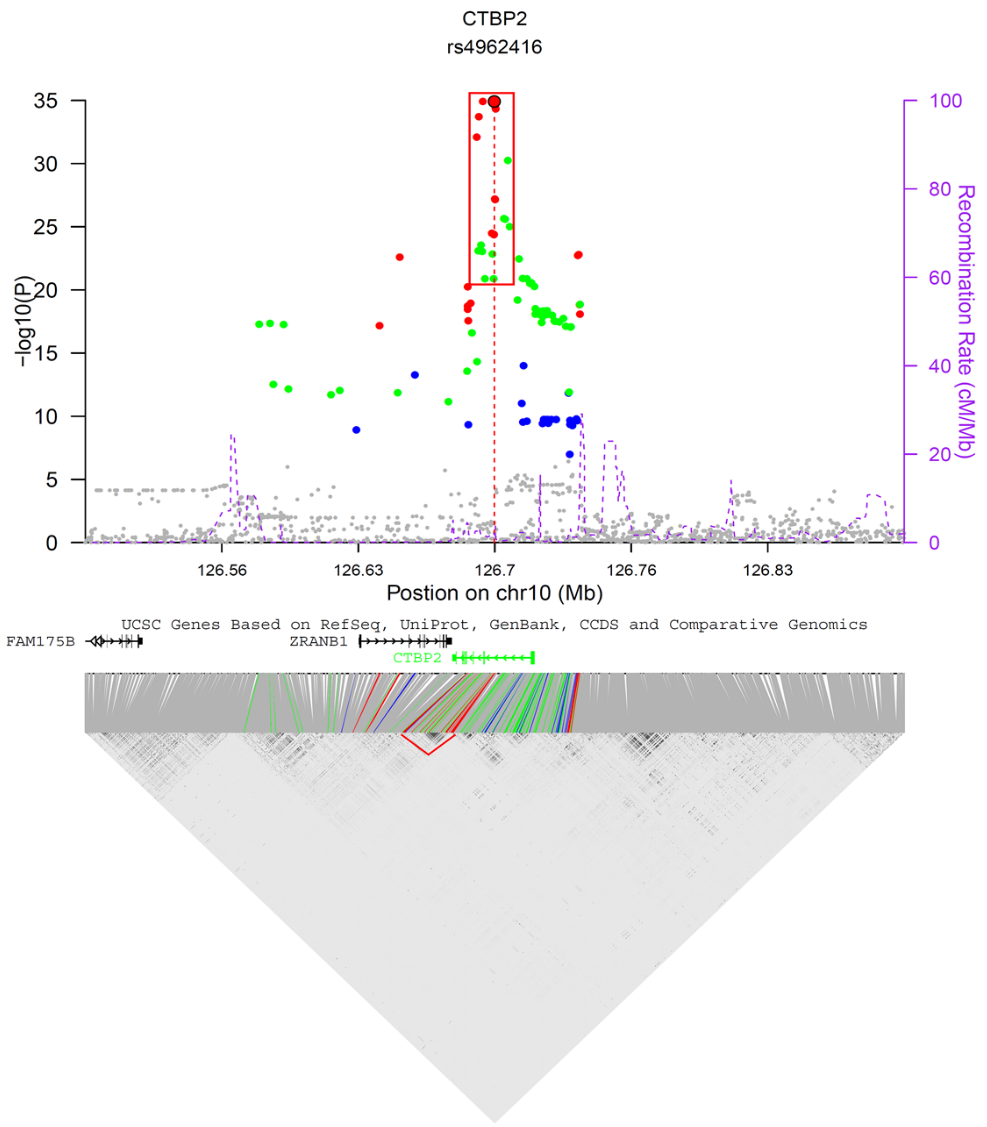

CTBP2
rs4962416
−log10(P)
Recombination Rate (cM/Mb)
Postion on chr10 (Mb)
126.56
126.63
126.7
126.76
126.83
UCSC Genes Based on RefSeq, UniProt, GenBank, CCDS and Comparative Genomics
FAM175B
ZRANB1
CTBP2

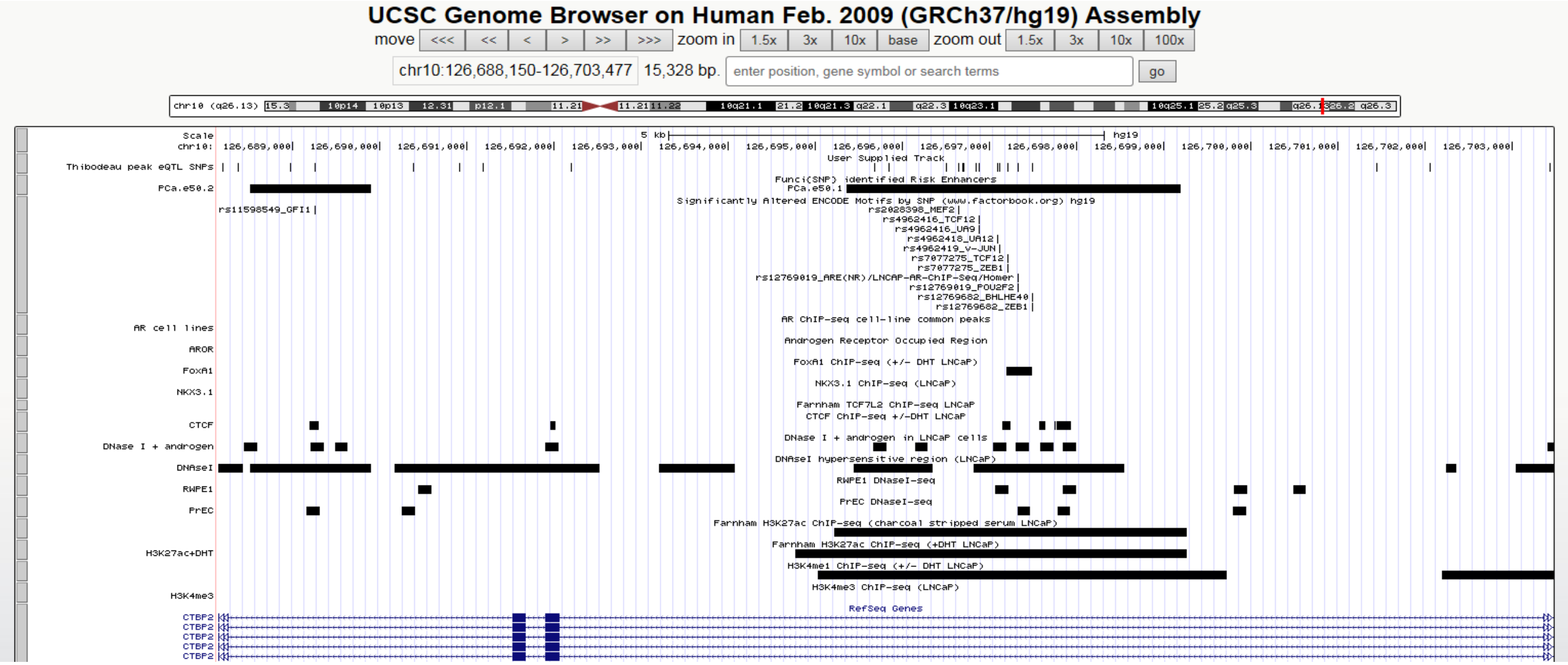
UCSC Genome Browser on Human Feb. 2009 (GRCh37/hg19) Assembly
move <<< << < > >> >>> zoom in 1.5x 3x 10x base zoom out 1.5x 3x 10x 100x
chr10:126,688,150-126,703,477 15,328 bp.
enter position, gene symbol or search terms
go
Scale
chr10:
Thibodeau peak eQTL SNPs
PCa.e50.2
User Supplied Track
Func(SNP) identified Risk Enhancers
PCa.e50.1
Significantly Altered ENCODE Motifs by SNP (www.factorbook.org) hg19
rs11598549_GFI1
rs2028398_MEF2
rs4962416_TCF12
rs4962416_UA9
rs4962418_UA12
rs4962419_v-JUN
rs7077275_TCF12
rs7077275_ZEB1
rs12769019_ARE(NR)/LNCAP-AR-ChIP-Seq/Homer
rs12769019_POU2F2
rs12769682_BHLHE40
rs12769682_ZEB1
AR cell lines
AR ChIP-seq cell-line common peaks
AROR
Androgen Receptor Occupied Region
FoxA1
FoxA1 ChIP-seq (+/- DHT LNCaP)
NKX3.1
NKX3.1 ChIP-seq (LNCaP)
Farnham TCF7L2 ChIP-seq LNCaP
CTCF
CTCF ChIP-seq +/-DHT LNCaP
DNase I + androgen
DNase I + androgen in LNCaP cells
DNaseI
DNaseI hypersensitive region (LNCaP)
RWPE1
RWPE1 DNaseI-seq
PrEC
PrEC DNaseI-seq
Farnham H3K27ac ChIP-seq (charcoal stripped serum LNCaP)
H3K27ac+DHT
Farnham H3K27ac ChIP-seq (+DHT LNCaP)
H3K4me1 ChIP-seq (+/- DHT LNCaP)
H3K4me3
H3K4me3 ChIP-seq (LNCaP)
RefSeq Genes
CTBP2
CTBP2
CTBP2
CTBP2
CTBP2

Supplementary Figure 7 - Mapping of candidate regulatory domains and candidate causative SNPS responsible for *ASCL2A* gene dysregulation

Panel A shows the regional association plot for *ASCL2* over a 180Kb region. The peak eQTL region explored further (panels A and C) is approximately 35 kb and is outlined by a red box. The characteristics of the plot are described in figure 1.

Panel B shows the UCSC genome browser for the 35Kb peak eQTL region (as outlined in panel B), providing a number of tracks used to map elements important for gene regulatory domains as described in Hazelett et. al.[1] The 54 peak eQTL SNPs are shown in the first track as reference points (see table for identification).

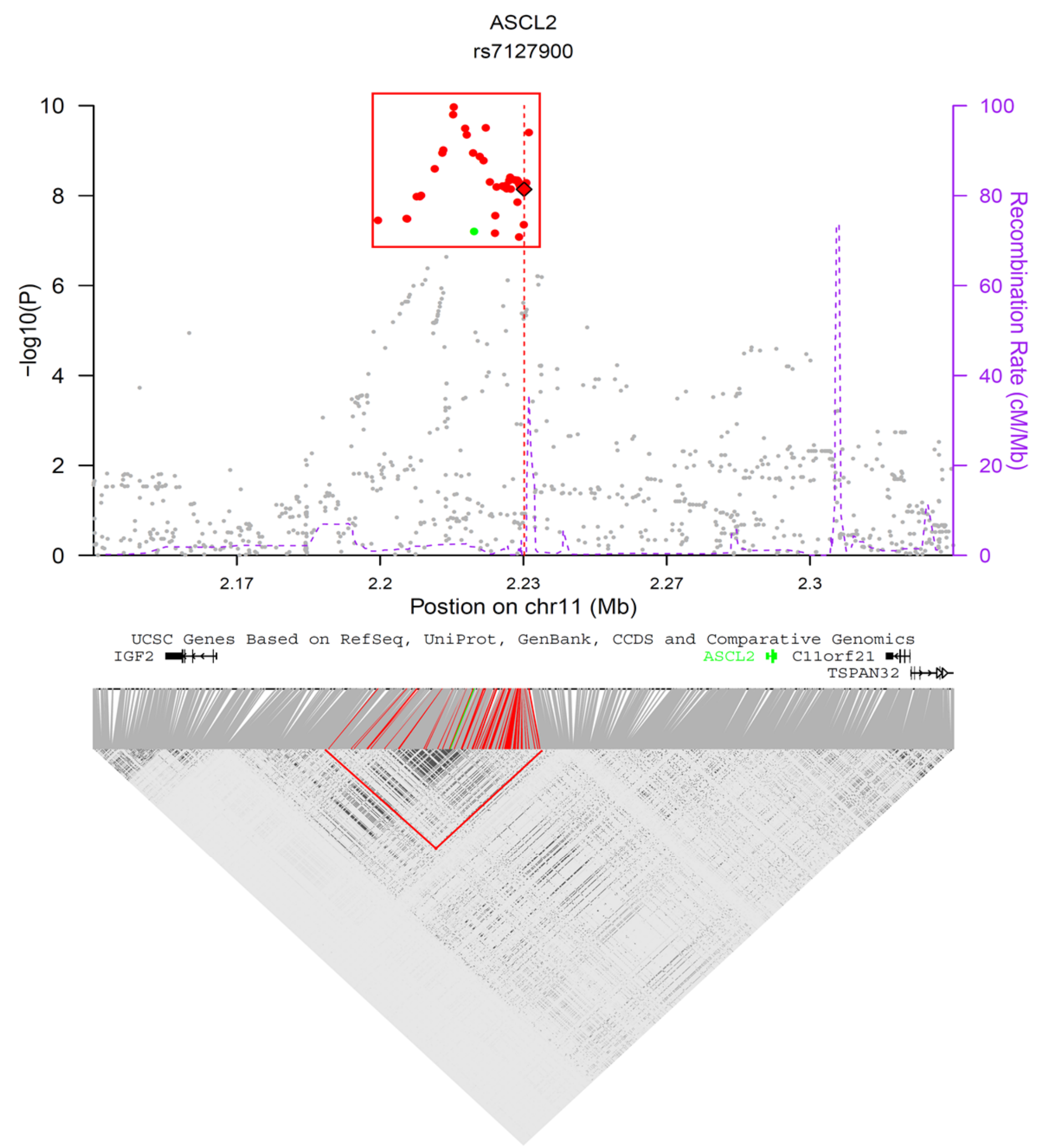
ASCL2
rs7127900
-log10(P)
Recombination Rate (cM/Mb)
Postion on chr11 (Mb)
2.17
2.2
2.23
2.27
2.3
UCSC Genes Based on RefSeq, UniProt, GenBank, CCDS and Comparative Genomics
IGF2
ASCL2
C11orf21
TSPAN32

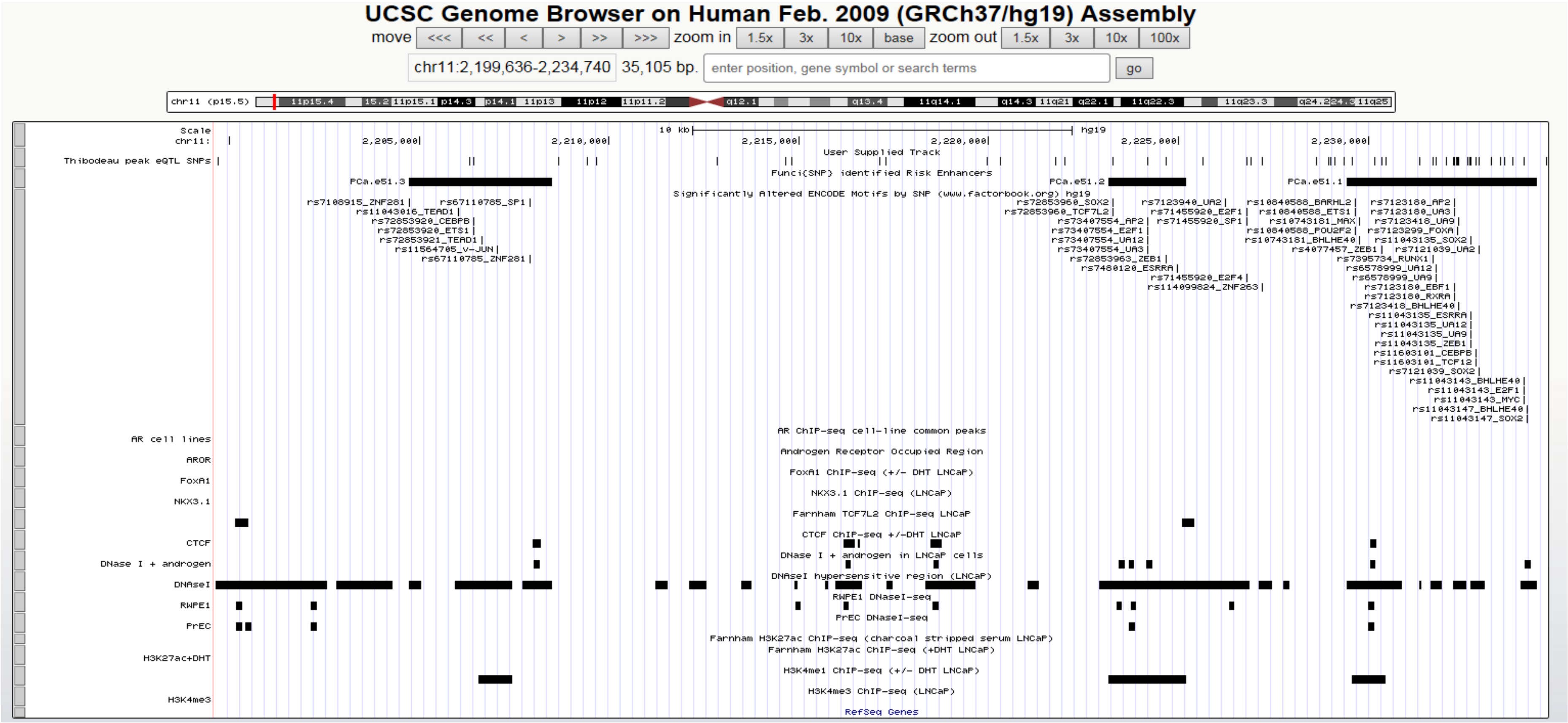
UCSC Genome Browser on Human Feb. 2009 (GRCh37/hg19) Assembly
move <<< << < > >> >>> zoom in 1.5x 3x 10x base zoom out 1.5x 3x 10x 100x
chr11:2,199,636-2,234,740 35,105 bp.
enter position, gene symbol or search terms
go
chr11 (p15.5)
Scale
chr11:
10 kb
hg19
2,205,000
2,210,000
2,215,000
2,220,000
2,225,000
2,230,000
User Supplied Track
Thibodeau peak eQTL SNPs
Funci{SNP} identified Risk Enhancers
PCa.e51.3
PCa.e51.2
PCa.e51.1
Significantly Altered ENCODE Motifs by SNP (www.factorbook.org) hg19
rs7108915_ZNF281
rs67110785_SP1
rs11043016_TEAD1
rs72853920_CEBPB
rs72853920_ETS1
rs72853921_TEAD1
rs11564705_v-JUN
rs67110785_ZNF281
rs72853960_SOX2
rs72853960_TCF7L2
rs73407554_AP2
rs73407554_E2F1
rs73407554_UA12
rs73407554_UA3
rs72853963_ZEB1
rs7480120_ESRRA
rs7123940_UA2
rs71455920_E2F1
rs71455920_SP1
rs71455920_E2F4
rs114099824_ZNF263
rs10840588_BARHL2
rs10840588_ETS1
rs10743181_MAX
rs10840588_POU2F2
rs10743181_BHLHE40
rs4077457_ZEB1
rs7123180_AP2
rs7123180_UA3
rs7123418_UA9
rs7123299_FOXA
rs11043135_SOX2
rs7121039_UA2
rs7395734_RUNX1
rs6578999_UA12
rs6578999_UA9
rs7123180_EBF1
rs7123180_RXRA
rs7123418_BHLHE40
rs11043135_ESRRA
rs11043135_UA12
rs11043135_UA9
rs11043135_ZEB1
rs11603101_CEBPB
rs11603101_TCF12
rs7121039_SOX2
rs11043143_BHLHE40
rs11043143_E2F1
rs11043143_MYC
rs11043147_BHLHE40
rs11043147_SOX2
AR cell lines
AR ChIP-seq cell-line common peaks
AROR
Androgen Receptor Occupied Region
FoxA1
FoxA1 ChIP-seq (+/- DHT LNCaP)
NKX3.1
NKX3.1 ChIP-seq (LNCaP)
Farnham TCF7L2 ChIP-seq LNCaP
CTCF
CTCF ChIP-seq +/-DHT LNCaP
DNase I + androgen
DNase I + androgen in LNCaP cells
DNAseI
DNAseI hypersensitive region (LNCaP)
RWPE1
RWPE1 DNaseI-seq
PrEC
PrEC DNaseI-seq
Farnham H3K27ac ChIP-seq (charcoal stripped serum LNCaP)
Farnham H3K27ac ChIP-seq (+DHT LNCaP)
H3K27ac+DHT
H3K4me1 ChIP-seq (+/- DHT LNCaP)
H3K4me3 ChIP-seq (LNCaP)
H3K4me3
RefSeq Genes

Supplementary Figure 8 - Summary of published eQTL studies used for comparison

Venn diagram depicting the overlap of the positive associations found in each study. The most common genes (found in 3 or more studies) are listed.

	Expression platform	Genotyping Platform	# samples	Tissue	# QWAS SNPs	Genes tested
**Mayo**	Rnaseq	Illumina 2.5 M	471	normal	147	(+/- 1 Mb)
**Xu**[4]	RNAseq	Aff 6.0	50	tumor	51	(+/- 1 Mb)
**Grisanzio**[2]	NanoString nCounter	Sequenom	407 / 255	normal / tumor	12	(+/- 0.5 Mb)
**Li**[3]	RNAseq	Affy 6.0	145	tumor	69	(+/- 0.5 Mb)

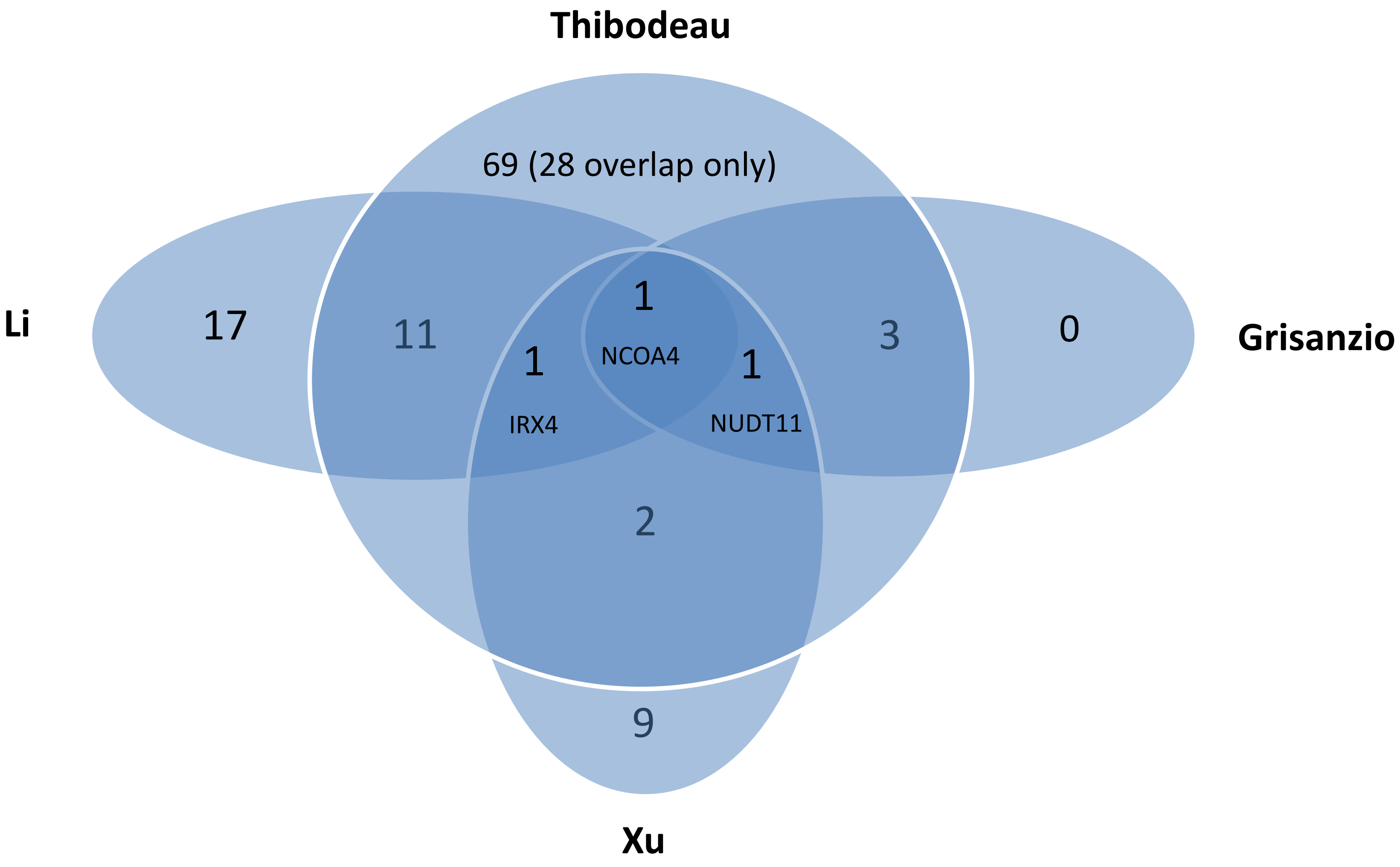

Supplementary References

1. Hazelett, D.J. *et al.* Comprehensive functional annotation of 77 prostate cancer risk loci. *PLoS genetics.* **10**, e1004102 (2014).
2. Grisanzio, C. *et al.* Genetic and functional analyses implicate the NUDT11, HNF1B, and SLC22A3 genes in prostate cancer pathogenesis. *Proc. Natl. Acad. Sci. U. S. A.* **109**, 11252-7 (2012).
3. Li, Q. *et al.* Expression QTL-based analyses reveal candidate causal genes and loci across five tumor types. *Hum. Mol. Genet.* **23**, 5294-302 (2014).
4. Xu, X. *et al.* Variants at IRX4 as prostate cancer expression quantitative trait loci. *Eur. J. Hum. Genet.* **22**, 558-63 (2014).
